# A meta-analysis of global fungal distribution reveals climate-driven patterns

**DOI:** 10.1038/s41467-019-13164-8

**Published:** 2019-11-13

**Authors:** Tomáš Větrovský, Petr Kohout, Martin Kopecký, Antonin Machac, Matěj Man, Barbara Doreen Bahnmann, Vendula Brabcová, Jinlyung Choi, Lenka Meszárošová, Zander Rainier Human, Clémentine Lepinay, Salvador Lladó, Rubén López-Mondéjar, Tijana Martinović, Tereza Mašínová, Daniel Morais, Diana Navrátilová, Iñaki Odriozola, Martina Štursová, Karel Švec, Vojtěch Tláskal, Michaela Urbanová, Joe Wan, Lucia Žifčáková, Adina Howe, Joshua Ladau, Kabir Gabriel Peay, David Storch, Jan Wild, Petr Baldrian

**Affiliations:** 1 Laboratory of Environmental Microbiology, Institute of Microbiology of the Czech Academy of Sciences, Vídeňská 1083, 14220 Praha 4, Czech Republic; 2 Faculty of Science, Charles University, Albertov 6, 12844 Praha 2, Czech Republic; 3 Institute of Botany of the Czech Academy of Sciences, Zámek 1, 25243 Průhonice, Czech Republic; 4 Faculty of Forestry and Wood Sciences, Czech University of Life Sciences Prague, Kamýcká 129, 16521 Praha 6, Czech Republic; 5 Center for Theoretical Study, Charles University and the Czech Academy of Sciences, Jilská 1, 11000 Praha 1, Czech Republic; 6 Center for Macroecology, Evolution and Climate, Natural History Museum of Denmark, University of Copenhagen, DK-2100 Copenhagen, Denmark; 7 Biodiversity Research Centre, University of British Columbia, 2212 Main Mall, Vancouver, V6T 1Z4 Canada; 8 Department of Agricultural and Biosystems Engineering, Iowa State University, 1201 Sukup Hall, Ames, IA 50011 USA; 9 Department of Biology, Stanford University, Stanford, CA 94305 USA; 10 Gladstone Institutes, San Francisco, CA 94158 USA

**Keywords:** Biodiversity, Climate-change ecology, Microbial ecology, Fungal ecology

## Abstract

The evolutionary and environmental factors that shape fungal biogeography are incompletely understood. Here, we assemble a large dataset consisting of previously generated mycobiome data linked to specific geographical locations across the world. We use this dataset to describe the distribution of fungal taxa and to look for correlations with different environmental factors such as climate, soil and vegetation variables. Our meta-study identifies climate as an important driver of different aspects of fungal biogeography, including the global distribution of common fungi as well as the composition and diversity of fungal communities. In our analysis, fungal diversity is concentrated at high latitudes, in contrast with the opposite pattern previously shown for plants and other organisms. Mycorrhizal fungi appear to have narrower climatic tolerances than pathogenic fungi. We speculate that climate change could affect ecosystem functioning because of the narrow climatic tolerances of key fungal taxa.

## Abstract (web summary)

The authors assemble and analyse previously generated mycobiome data linked to geographical locations across the world. They describe the distribution of fungal taxa and show that climate is an important driver of fungal biogeography and that fungal diversity appears to be concentrated at high latitudes.

## Introduction

Fungi are eukaryotic microorganisms that play fundamental roles in regulating key ecosystem processes. As major decomposers of organic matter as well as mutualists or pathogens of plants, soil fungi significantly influence plant primary production, carbon mineralisation and sequestration, and act as crucial regulators of the soil carbon balance, which is one of the greatest topics of human security in this century^1–3^. Moreover, the activities of diverse soil communities markedly contribute to the production of clean water, food and air and the suppression of disease-causing soil organisms. As such, soil biodiversity is increasingly recognised to provide services critical to food safety and human health^4^.

Considering the magnitude of ongoing global change, it is of high importance to determine how climate and other environmental factors affect the diversity and distribution of fungal communities^5^. Fungal symbionts can benefit plants by ameliorating abiotic stressors associated with climate change, such as heat and drought^6,7^, but the distribution of these symbionts may be driven by mechanisms other than climate^8,9^. However, because the geographic distribution and environmental preferences of nearly all fungi remain unknown^10^, their current status and the future threats to their existence are difficult to assess^11,12^. Due to the importance of plant–fungal interactions, the ability to predict shifts in fungal distributions could help to understand or predict ecosystem-level changes.

Climatic^13,14^ and edaphic variables^15^ as well as vegetation features^3^ were recently reported to influence the composition of fungal communities. Moreover, fungal diversity has been reported to respond to climate factors in drylands^16^. It is less clear whether the global fungal biodiversity patterns correspond with higher diversities at low latitudes that were previously demonstrated for terrestrial macroorganisms and bacteria^2,17^. The distribution of plant and animal species also demonstrates strong division into biogeographical regions^18^. Although some biogeographic patterns were also observed for bacteria^19^, other data show that dominant microbes are widespread^20^ and may thus exist in all areas of suitable environments.

In addition to the climate, soil characteristics or vegetation, fungal diversity and community composition might also be shaped by other factors, such as dispersal limitations of certain taxa^21,22^, although transcontinental dispersal of fungi has also been documented^23,24^. Similarly, fungal community surveys have shown a mixed degree of regional endemism^10,25^ and identified groups with rather cosmopolitan distributions^26,27^.

So far, only few studies focused on fungal distribution and diversity on global scale^10,28,29^. Importantly, these single survey studies focused on limited number of biomes^10,28^ or narrower taxonomical group within the Fungal kingdom^29^. Here, we have undertaken a comprehensive meta-study of data published on the composition of soil fungal communities. This approach enabled us to re-analyse multiple datasets from different biogeographical regions and biomes and compile a large dataset of fungal taxa distribution worldwide. We particularly aimed to (i) identify the most common fungal taxa worldwide (fungal taxa which occurred in more than 5% of samples), (ii) describe how the global distributions of fungal hypothesised species and fungal ecological groups are constrained by climate and other important environmental factors and (iii) assess global patterns of fungal diversity.

## Results and discussion

### Global fungal biogeography and its drivers

**Fig. 1:**
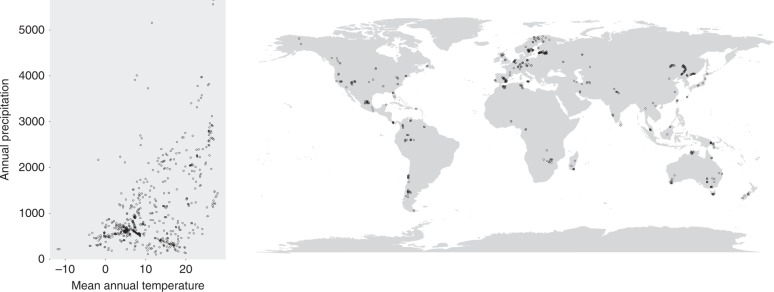
Mean annual temperature and annual precipitation of the analysed samples and their geographic distributions

Our meta-study is based on high-throughput sequence data from 3084 soil samples, which represents a large extant fungal community dataset that spans a wide range of climatic conditions (Fig. 1, Supplementary Table 1) and originate from 36 independent studies. Most of the samples were from Europe, followed by North America, Asia, South America, Australia and Africa. The samples were overwhelmingly from forests, followed by deserts, grasslands, shrublands, woodlands, croplands and tundra. Compared to previous large-scale studies focused on fungal diversity^10,16,30^, our analysis spans the widest spectra of vegetation types.

After obtaining the raw sequence data from each study, the sequences that passed quality check were binned by species hypotheses (SHs), which are the molecular taxa that represent fungal species proxies. The SH concept relies on molecular keys, reference sequences and voucher material specified by taxonomists and enables precise communication and delimitation of traditionally classified species as well as environmental sequences^31^. The assignment of environmental sequences to such a defined dataset enables future references to the results, which is impossible to achieve by de novo sequence classification. On average, 52.7 ± 19.5% (SD) of the sequences per sample were assigned to an existing SH. Of the most frequently recorded SHs found in >5% of all samples (469 SH, “top taxa”), 70% belonged to Ascomycota, 18% to Basidiomycota and 9% to Mucoromycota; one SH belonged to Cryptomycota (Supplementary Data 1). Out of the 258 top taxa with assigned ecology, 144 were saprotrophs, 29 were plant pathogens, 16 were endophytes, 38 were ectomycorrhizal fungi, 10 were animal pathogens, 17 were wood saprotrophs, 3 were ericoid mycorrhizal fungi and 1 was mycoparasite.

**Fig. 2:**
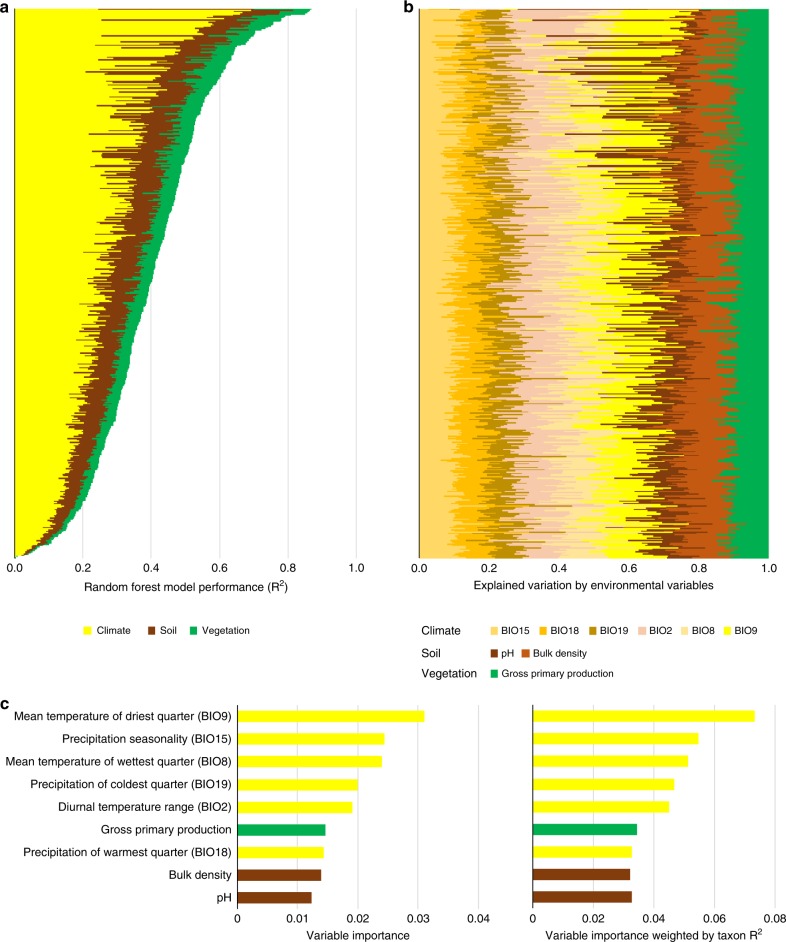
Environmental variables explaining the global distribution of the most frequent fungal taxa. **a** Random forest model performance for 457 fungal taxa where out-of-bag *R*^2^ > 0. **b** Contribution of climatic, soil and vegetation variable categories to the variation explained by the complete random forest model for each fungal taxon. **c** Importance of individual environmental variables across models for all fungal taxa showing raw variable importance and variable importance weighted by out-of-bag *R*^2^ for each taxon

Random forest models separately calculated for each of the SH (Supplementary Software 1) indicated that environmental variables significantly constrained the geographic distribution of 457 of the 469 top taxa (97%), representing all fungal ecological groups (e.g., saprotrophs, mycorrhizae and pathogens). The mean model performance was 40% of the explained variance, while the maximum performance was 86.9%; for 414 of the top taxa, the random forest performance exceeded 20% (Fig. 2a). Of the environmental factors, 65.5% of the variability was explained by climate factors, 23.7% was explained by variables related to soil and 11.2% was explained by vegetation variables. Among the climate variables, more of the explained variation was associated with temperature and its variation (38.7%) than with precipitation (26.8%). Among the soil characteristics, bulk density (13.8%) contributed more than soil pH (9.5%; Fig. 2b). Across all top taxa, the random forest models were most sensitive to mean temperature of driest quarter and precipitation seasonality (Fig. 2c). However, it should be acknowledged that the climate, soil, as well as vegetation variables were obtained from global databases and although the quality and application of such data in research is rapidly increasing, our results might be affected by resolution of this type of data. Considering that temperature increase and changes in precipitation seasonality are frequently predicted under future climate change^32^, our models predict that these may be the predominant drivers of changing fungal species distributions in the future. Because the factors that constrained the geographic distributions differed significantly among taxa (Fig. 2b), the idiosyncratic responses of species to climate change will likely have a considerable effect on composition of fungal communities in the future. Similarly, random forest models indicated that climate significantly constrained the geographic distribution of most of the fungal families, although among 50 most abundant fungal families, significant predictions were obtained for a smaller share than for fungal top taxa (74 and 97%) and the explained variation was in average lower—14 and 40% (Supplementary Figs. 1 and 2). This is likely due to the variation of ecological requirements including the nutritional modes among species within fungal families^33^.

**Fig. 3:**
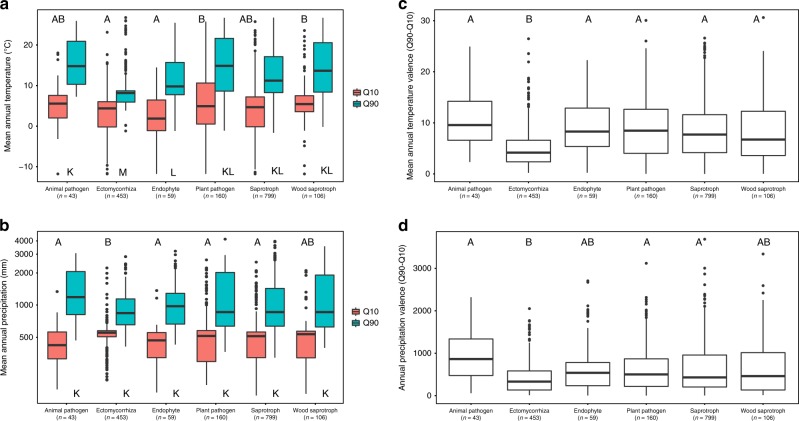
Climatic determinants of ecological guilds of fungi. **a** The first and ninth deciles of sample mean annual temperature and **b** annual precipitation for SHs belonging to selected ecological guilds with occurrence in >10 samples. **c** Ecological variance of mean annual temperature and **d** annual precipitation of these SHs expressed as the difference between the ninth and tenth deciles. The boxes marked with different letters are significantly different at the *p* < 0.05 according to Kruskal–Wallis with post hoc Nemenyi tests. In boxplots, middle line represents median, upper and bottom horizontal lines represent third and first quartile, whiskers represent maximum and minimum values below the upper and lower fence and points represent outliers

When the fungal SHs belonging to various ecological guilds were compared, ectomycorrhizal fungi were found to have cooler upper temperature limit than those of other guilds (Fig. 3a, b). The temperature valence of ectomycorrhizal fungi was 4.2 °C on average, which was lower than that in other guilds (6.7–9.6 °C), and their precipitation valence (330 mm) was narrower than that in all other guilds (430–860 mm) except endophytes and wood saprotrophs due to the relative low occurrence of ectomycorrhizal fungi in samples with low precipitation (Fig. 3c, d). The narrower climate niche of ectomycorrhizal fungi indicates that these mutualistic plant symbionts might have to respond to climate change challenges by shifting their phenology, distribution range or physiology. This largely differs from the expected responses of plant pathogens which seem to have much broader climate niches. Climate also influenced the relative abundances of fungal guilds across the globe, and guilds such as ericoid mycorrhizal fungi were nearly absent from particular regions of climate space (Supplementary Fig. 3). Our results are in line with recently demonstrated patterns observed on a small-scale^34^. It should be, however, noted that these results were obtained with 1620 fungal SHs, those where sufficient numbers of observations were available. Although these SHs represent the majority of observations across the whole dataset, more data will be needed in the future to prove whether the trends hold for other members of the respective guilds.

The current paradigm suggests that regional fungal assemblages are characterised by frequent endemic species occurrences^1^, and the distribution of these endemics is constrained by dispersal limitations^22^. The dataset of this study does not allow us to address the question of endemism directly, however, most of the top SH were cosmopolitan species, with 29% found on all six continents, 20% found on five continents and 14% found on four continents. Only 8.5% of top taxa were constrained to a single continent (Supplementary Figs. 4 and 5). Of the 3943 frequent SHs that occurred in 10 or more samples, the share of taxa constrained to one continent was also low (23%), while 48% of SHs occurred on three or more continents and 8% occurred on all continents (Supplementary Fig. 4). In a global survey of arbuscular mycorrhizal fungi, endemism was also rare and limited to 7% of taxa found on only one continent while 34% of taxa occurred on all sampled continents and contrasted with the high frequency of endemism displayed by plants^27^. Although mostly cosmopolitan, top taxa were frequently associated with samples from sites with similar climates (Supplementary Fig. 5).

**Fig. 4:**
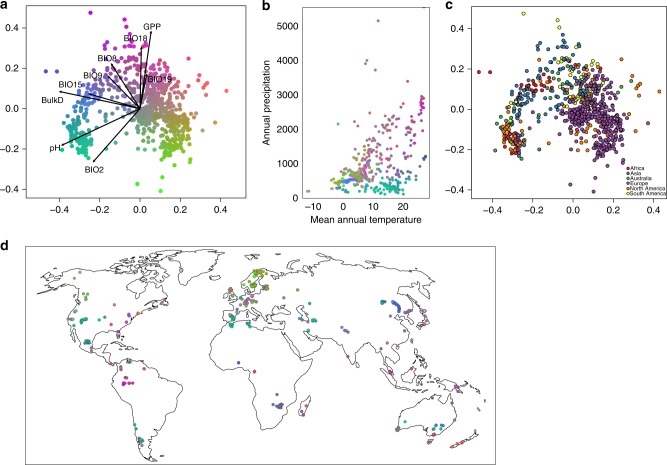
Variation in community composition of fungal SH is driven by climate, but only weakly structured by dispersal. **a** Three-dimensional non-metric multidimensional scaling of fungal community composition based on Bray–Curtis dissimilarities (stress = 0.13). The figure shows the first two ordination axes, but the third ordination axis is captured through the rgb colour coding of the samples. All environmental variables used in the random forest models are passively projected onto the ordination space. **b** Mean annual temperatures and annual precipitation of all analysed samples coloured as in panel (**a**). **c** The same ordination as in (**a**), but with samples coloured according to the continents, suggests relatively weak geographic patterns in the data. **d** Geographic distribution of the samples. The samples are coloured as in panel a; the more similar the colour is, the more similar the community composition of the samples

The overall compositions of the fungal communities showed strong association with environmental variables, while separate clustering by continents was less clear (Fig. 4a, c). The limited share of potentially endemic species among top taxa may indicate that for the most common fungal species, environmental variables represent more important drivers of community composition than local speciation and dispersal barriers (Fig. 4b, d). This is also well documented by high similarity of fungal communities from similar environments, but different continents (e.g., tropical rain forests in South America, Africa and Southeast Asia). In contrast to macroorganisms such as mammals and plants^18^, the distribution of dominant fungi seems to be less constrained by geographic barriers, probably due to better dispersal abilities. Given that there are also a number of cases where endemism and dispersal limitations have been documented for populations and higher taxonomic groups^1,35^ and considering that the share of endemic species is likely higher for less common fungal taxa, future work should attempt to reconcile these observations and our findings with respect to frequently observed taxa.

### Global patterns of fungal diversity

**Fig. 5:**
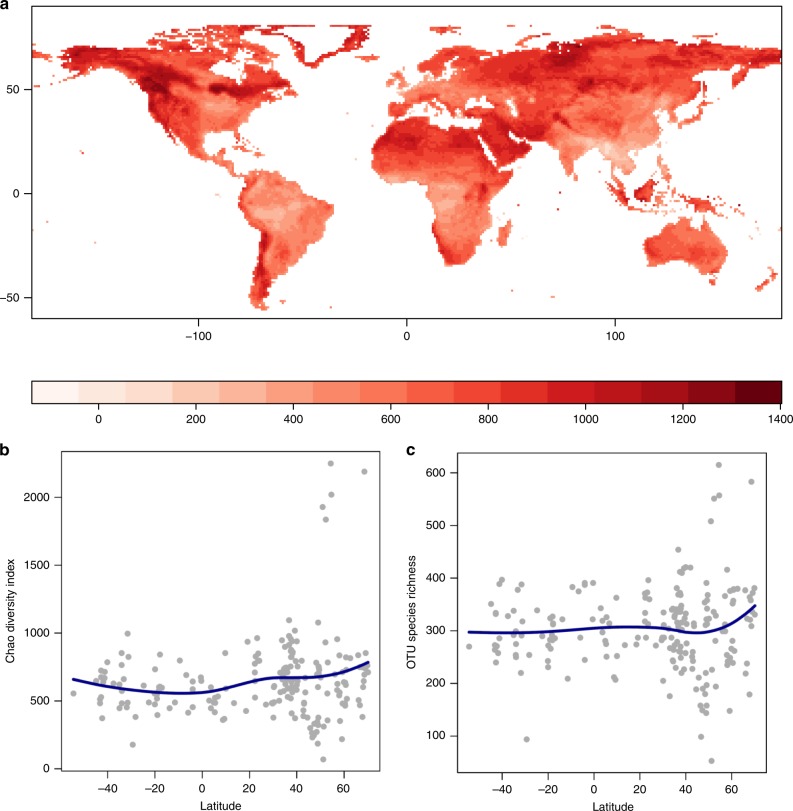
Inferred patterns of fungal species diversity predicted by the best-subset GLM. **a** The Chao index of the OTU diversity projection (model *R*^2^ = 17.2%); **b** latitudinal gradient fitted by the means of the non-parametric smoothing of the Chao index; **c** latitudinal gradient fitted by the means of the non-parametric smoothing of the OTU richness

We used multiple statistical setups, including random forests, best-subset regression and non-parametric smoothing, applied to whole fungal communities classified into operational taxonomic units (OTUs). We evaluated the diversity of these communities in terms of richness (number of species) and when accounting for abundance data (Chao’s index). Both diversity metrics across the different setups consistently revealed relatively low diversity of fungi in the tropics. In contrast, temperate regions showed significant variation in fungal diversity, with most diverse regions concentrated towards high latitudes (Fig. 5a–c, Supplementary Figs. 6 and 7). Tropical diversity and the diversity across the Southern Hemisphere appeared comparatively low, except for several putative hotspots (Fig. 5a). While the results across different methods often diverged in details (e.g., diversity hotspots based on richness and Chao’s index), they did not provide any compelling support for high diversity of fungi in the tropics, which contrasts dramatically with the well-known patterns for plants, arthropods, vertebrates^2^ and some bacteria^17^. Furthermore, fungal diversity showed modest negative relation to temperature, which corroborates our finding of high diversity at cold latitudes (GLM *R*^2^ ≈15%, Random Forest *R*^2^ ≈25%). While these results will need to be further refined and revisited as more data, especially from the currently undersampled regions, become available, present comparisons strongly indicate surprisingly low diversity of fungi in the tropics.

Our meta-study indicates that the global distribution of fungi is shaped by multiple environmental factors but climate is the primary one for the most common fungal species. This finding underscores how profound the effects of ongoing climate change may be to both ecosystem functioning and food safety. For example, beneficial ectomycorrhizal fungi show narrow climatic niches compared to plant pathogens. The effects of climate on fungal distribution can thus seriously disrupt plant productivity. Further work, such as observations of fungal community composition changes over time, is needed to extend the current results in order to assess the responses of fungal communities to changing climates. Our results pave the way for such further research and identify climate as a principal driver of the biogeographic patterns in fungi, which seem to differ dramatically from the patterns known across other eukaryotes and bacteria.

## Methods

### Data selection

We explored papers that used high-throughput sequencing for the analysis of fungal communities that were published by 2016; in total, we explored 479 papers. The following selection criteria were used for the inclusion of samples (and, consequently, studies) into the dataset: (1) samples came from soils (including litter) that were not subject to experimental treatment; (2) the precise geographic location of each sample was recorded (GPS coordinates); (3) the whole fungal community was subject to amplicon sequencing (studies using group-specific primers were excluded); (4) the internal transcribed spacer regions (ITS1, ITS2 or both) were subject to amplification; (5) sequencing data (either in fasta or fastq format) were publicly available or provided by the authors of the study upon request, and the sequences were unambiguously assigned to samples and (6) the samples could be assigned to biomes according to the Environment Ontology (http://www.ontobee.org/ontology/ENVO). In total, 67 publications contained samples that matched our criteria (Supplementary Table 1); however, for 31 of the publications, we were not able to obtain sequencing data (the data were neither public nor provided by the authors upon request). PRISMA Flow Diagram^36^ reporting literature search and selection process is provided in Supplementary Fig. 8.

### Processing of sequencing data

In total, 66,865,554 sequences were obtained from 36 studies covering 3370 soil samples after trimming sequences with mean quality phred scores below 20. Each sequence was labelled using the combination of a sample ID and sequence ID, and the full ITS1 or ITS2 fungal region was extracted using Perl script ITSx v1.0.11^37^. ITS extraction resulted in a total of 3,228,494 full ITS1 and 22,966,153 full ITS2 sequences. For each original sequence, either ITS1 or ITS2 was selected based on quality. The extracted ITS sequences were classified according to the representative sequence of the closest UNITE species hypothesis (SH) using BLASTN, considering the SH created using a 98.5% similarity threshold (general release 22.8.2016^31^). The best BLASTN hit results were filtered with the following thresholds: *e*-value < 10e^−50^, sequence similarity ≥98.5%. The filtering resulted in 11,375 536 sequences assigned to SHs originating from 3084 soil samples. Poorly preserved samples that were dominated by moulds (relative abundance of sequences belonging to >20% Mortierellaceae, >20% Mucoraceae or >5% Trichocomaceae) were removed as previously recommended^10^ since high mould dominance typically results from growth after sampling and does not correspond to the abundance of mould in soil.

For the analysis of fungal diversity, all fungal sequences with full ITS2 sequences were clustered into OTUs at 98.5% similarity using VSEARCH^38^ instead of assigning the sequences to SHs. Representative sequence of each OTU was selected for BLASTn searches against the International Nucleotide Sequence Databases Collaboration and UNITE databases. We considered e-values of BLASTn search results < e^−50^ reliable to assign sequences to the fungal kingdom^10^. This was done because the share of sequences assigned to existing SHs differed among samples and could have affected the results (fungal sequences not assigned to SHs represent equally valid fungal taxa). Samples were randomly subsampled to 1000 sequences, and samples with fewer sequences were not considered.

### Sample descriptions

Sample metadata were collected from the published papers and/or public repositories where they were submitted by the authors. In some cases, metadata were obtained from the authors of individual studies upon request. The samples were assigned to continents, and all sites were categorised into biomes following the classification of Environment Ontology to a maximum achievable depth for each sample.

### Environmental data for samples

To explore main environmental drivers of soil fungal distribution at the global scale, we analysed climate, soil and vegetation data for each site. We extracted bioclimatic variables from the global CHELSA^39^ and WorldClim 2^40^ databases for each sample. Since the results based on CHELSA and WorldClim 2 were comparable, we decided to use the CHELSA for final analyses, because precipitation patterns are better captured in the CHELSA dataset^39^. We explored all 19 bioclimatic variables derived from the monthly temperature and precipitation data, which represent different aspects of the climate like long-term averages (e.g., mean annual temperature and annual precipitation), seasonality (e.g., annual range in temperature and precipitation) and climatic extremes (e.g., temperature of the coldest and warmest month and precipitation of the wettest and driest quarters).

To explore soil drivers of fungal distribution, we extracted four soil variables relevant for soil fungal distribution from SoilGrids^41^. Because vast majority of studies focused on soil fungal communities in top soil layers, we extracted following variables for the first 5 cm of soil horizon: soil pH, cation exchange capacity, bulk density and organic carbon content.

Besides that, we used two remote sensing proxies to explore vegetation productivity, both derived from MODIS satellite data. Enhanced vegetation index (EVI) is an improved version of the widely used NDVI, based on monthly MODIS data available in 1 km resolution and covers the period 2001–2012^42^. Gross primary productivity (GPP) is based on 8-day MODIS composite data available in 500 m resolution and covers period 2000–2018^43^.

### Biogeography of fungal taxa

A table of SH sequence counts in each sample was produced and inspected. Singleton sequences belonging to highly abundant SHs were often present in multiple samples located far from the remaining observations, and their occurrences often reflected the sampling plans of individual studies. This result indicated a high risk that singleton observations are often due to technical cross-contamination during sampling or sample processing, and we thus decided to remove all local singleton occurrences of all SHs from the dataset before analysis. The relative abundances of the SHs were calculated as the share of sequences classified to each SH divided by the total number of all classified sequences. Besides that, SHs were also grouped into fungal families.

Global distribution and relative abundance maps of fungal SHs as well as fungal families were created using custom made scripts with ggplot2, ggmap, maptools, sh, raster, rgdal and maps packages in the R statistical computing environment (R Core Team, 2018, v. 3.3.2). In addition, relative abundances of SH were also plotted against mean annual temperature and annual precipitation.

### Assignment and analysis of fungal ecological guilds

SHs were classified using FUNGuild^44^ into the following ecological guilds: animal pathogens, arbuscular mycorrhizal, ectomycorrhizal, endophytes, ericoid mycorrhizal, foliar epiphyte, lichenicolous, lichenized, mycoparasites, plant pathogens, saprotrophs and wood saprotrophs. Where necessary, assignments were corrected based on the literature (e.g., all *Acephala* spp. except *A. macrosclerotiorum* were considered endophytes; *Meliniomyces variabilis* was considered an ericoid mycorrhizal fungus; all *Gomphidius* spp. were considered mycoparasites). To analyse whether fungal guilds differ in the climatic ranges and climatic niche breadths of their members, all SHs with defined ecology and that occurred in >10 samples were analysed for guilds with >50 SHs (animal pathogens, ectomycorrhizal, endophytes, plant pathogens, saprotrophs and wood saprotrophs). For these 1620 SHs, we calculated the first and the ninth deciles of the mean annual temperature and precipitation as well as the niche breadths (ninth decile–1st decile). Non-parametric Kruskal–Wallis tests with Nemenyi post hoc comparisons were used to calculate the differences in climatic niche breadths between the 1st and the 9th deciles among the selected fungal guilds (PMCMR package in R). To counteract the problem with multiple comparisons, we used Bonferroni correction to calculate adjusted *p* values.

Considering that the distribution of ectomycorrhizal fungi (unlike saprotrophs and other fungal groups) is tightly associated with the presence of host plants, we also reanalysed the difference in climatic niche breadth among fungal guilds using a dataset limited to those samples, where the relative abundance of ectomycorrhizal fungi reached more than 5%. The results obtained using this limited dataset were similar to those reported for the full dataset containing all samples, which indicates that sample selection did not affect our results.

### Environmental drivers of distribution of fungal taxa

To reduce the effects of sampling depth on the analysis of environmental drivers of fungal distribution, only samples with at least 1000 sequences classified to SH (1578 samples in total) were selected. Because some samples represent the same sites with the same climate (for example individual samples from different soil depths collected at one site), we aggregated all samples to individual sites with unique geographic coordinates. This aggregation resulted in a final dataset of 742 sites with unique geographic coordinates.

To reduce collinearity among predictors, we reduced the initial set of 25 environmental variables to 9 variables with variation inflation factor (VIF) below 5. This final set included six bioclimatic variables (BIO2—diurnal temperature range; BIO8—mean temperature of wettest quarter; BIO9—mean temperature of driest quarter; BIO15—precipitation seasonality; BIO18—precipitation of warmest quarter and BIO19—precipitation of coldest quarter), two soil variables (soil pH and bulk density) and one productivity variable (GPP).

To explore the environmental drivers of distributions of fungal SHs and families, we modelled occurrence of each SH or family by Random Forest^45^. We used Random Forest implementation in R package *extendedForest*^45^, fitted 500 trees to each taxon and used random subset of one-third of the environmental predictors as candidates for each split of the tree. The overall predictive ability of the forest for each SH or family was calculated as the average proportion of out-of-bag data variance explained by the fitted forest.

In Random Forest, the predictor relative importance is quantified as the decrease in performance when each predictor is randomly permuted but other predictors are not modified^45^. To express variable importance across all modelled SHs or families, the relative importance of each predictor was calculated as a sum of predictor relative importances of all Random Forests for individual taxa weighted by Random Forest predictive ability (out-of-bag *R*^2^) for that taxa^46^. To allow comparison of our approach with approach based on AUC, we calculated pseudo-AUC for each taxon modelled in our study with ROCR package for R^47^.

### Analysis of community composition

To explore the main compositional gradients and relate them to environmental variables and geography, we ordinated the aggregated samples through non-metric multidimensional scaling (NMDS). In this analysis, we were interested in general pattern of compositional variation among sites, therefore, we analysed the whole dataset of 12,223 non-singleton SHs occurring on the 742 aggregated sites. As a dissimilarity index, we used the Bray–Curtis index calculated on Hellinger-transformed SH abundances. We calculated the global NMDS in three dimensions with the metaMDS function in the *vegan* R package, which implements weak treatment of ties^48^. Finally, we used the envfit function in the *vegan* R package to project all environmental variables used in Random Forest modelling onto NMDS compositional space.

### Global diversity of fungi

To explore the geographic patterns of fungal diversity, we assigned the sampled localities to 1 × 1 degree grid cells covering the globe. Grid-based rather than locality-based analyses can be used to standardise the geographic scale of the analysis, which facilitates cross-region comparisons and limits false presences in the data^49^. The grid-based approach is broadly favoured in biogeographic analyses for its suitability for large-scale comparisons (e.g., comparing richness across regions spanning tropical, temperate, and boreal climatic regimes, vegetation and soil types). Such comparisons would be hardly interpretable and computationally tractable under the site-based approach because of the issues of defining the geographic scale of a site, resolution of environmental data, gaps in large-scale environmental data, etc. Only comparable and sufficiently sampled grid cells containing OTUs unambiguously identified as fungi entered subsequent analyses, ensuring that tropical as well as temperate regions across multiple biogeographic realms (e.g., Afrotropics, Neotropics, Nearctic and Palaearctic^50^) are represented by high-quality occurrence data that are necessary to evaluate global diversity patterns. In cells containing multiple samples, one sample was randomly selected from the study that covered most cells.

Multiple approaches were used to evaluate the patterns of global diversity. First, we used non-parametric smoothing to investigate the changes in species diversity (number of OTUs) with latitude using a second-degree polynomial function fitted locally to 75% of the data points. Different parametrizations yielded qualitatively similar results. Second, we investigated the changes in species diversity across different regions of the world using environment-based generalised linear models (GLMs). Specifically, we characterised the environment within each 1 × 1 degree grid cell using bioclimatic variables BIO1-BIO19 as well as soil (pH, organic carbon content, bulk density and cation exchange capacity) and vegetation productivity data (GPP and EVI). Together, these variables are broadly considered to capture environmental conditions relevant to fungi, and have been shown to efficiently predict the patterns of regional diversity in microorganisms^10,51,52^. To identify the most parsimonious model of fungal diversity, we evaluated a set of candidate GLMs that contained different combinations of variables using an exhaustive search algorithm in the *leaps* R package^53^, but we limited the maximum size of the model to 10 variables to ensure tractability. In total, 18 million models were evaluated. The best model was selected based on the adjusted *R*^2^, AIC, and BIC values, and the best model was then used to predict the fungal diversity across all grid cells covering the globe. Namely, we predicted the diversity of species and the upper and lower bounds of the diversity (95% prediction interval). Third, we evaluated the global patterns of species diversity using random forest. Random forest is a machine learning algorithm that makes few statistical assumptions (e.g., compared to regression models) while maximising the predictive power and mitigating the issue of possible overfitting^45^. Random forest was implemented in R (package *randomForest*)^54^. Each tree was fitted based on a random sample of two-third of the observations (“in-bag”), and each tree split was based on a different random subset of one-third of the predictors, while the results were cross-validated against the remaining observations (“out-of-bag”), which is in line with standard protocols^45,54^.

The three statistical approaches, i.e., non-parametric smoothing, best-subset regressions and random forest, were applied to the OTU dataset to predict the species richness (=OTU richness) and Chao index^55^ in each 1 × 1 degree grid cell. Unlike species richness, the Chao index evaluates the regional diversity while taking into account the possible differences in the relative prevalence of rare and highly abundant species across grid cells. The same estimates were also calculated with the SH dataset, giving results comparable to the global biodiversity patterns based on OTU approach.

### Reporting summary

Further information on research design is available in the Nature Research Reporting Summary linked to this article.

## Supplementary information

Supplementary Information

Description of Additional Supplementary Files

Supplementary Data 1

Supplementary Software 1

Reporting Summary

## Data Availability

All data are publicly available online (see Supplementary Table 1 for details).

## Code availability

R-script is provided in Supplementary Software 1.
