# Supplementary material for: A meta-analysis of global fungal distribution reveals climate-driven patterns
Source: Nat Commun. 2019 Nov 13;10:5142. doi: 10.1038/s41467-019-13164-8 (PMC6853883; doi:10.1038/s41467-019-13164-8)
Supplement: Supplementary file 1 — Supplementary Information [file 41467_2019_13164_MOESM1_ESM.pdf]

Supplementary Information

A meta-analysis of global fungal distribution reveals climate-driven patterns

Tomáš Větrovský[1]*, Petr Kohout[1,2]*, Martin Kopecký[3,4], Antonin Macháč[2,5,6,7], Matěj Man[3], Barbara Doreen Bahnmann[1], Vendula Brabcová[1], Jinlyung Choi[8], Lenka Meszárošová[1], Zander Rainier Human[1], Clémentine Lepinay[1], Salvador Lladó[1], Rubén López-Mondéjar[1], Tijana Martinović[1], Tereza Mašínová[1], Daniel Morais[1], Diana Navrátilová[1], Iñaki Odriozola[1], Martina Štursová[1], Karel Švec[1], Vojtěch Tláskal[1], Michaela Urbanová[1], Joe Wan[9], Lucia Žifčáková[1], Adina Chuang Howe[8], Joshua Ladau[10], Kabir Gabriel Peay[9], David Storch[5,6], Jan Wild[3], Petr Baldrian[1]

[1]Laboratory of Environmental Microbiology, Institute of Microbiology of the Czech Academy of Sciences, Vídeňská 1083, 14220 Praha 4, Czech Republic
[2]Faculty of Science, Charles University, Albertov 6, 12844 Prague, Czech Republic
[3]Institute of Botany of the Czech Academy of Sciences, Zámek 1, 25243 Průhonice, Czech Republic
[4]Faculty of Forestry and Wood Sciences, Czech University of Life Sciences Prague, Kamýcká 129, 16521 Praha 6, Czech Republic
[5]Center for Theoretical Study, Charles University and the Czech Academy of Sciences, Jilská 1, 11000 Praha 1, Czech Republic
[6]Center for Macroecology, Evolution and Climate, Natural History Museum of Denmark, University of Copenhagen, DK-2100 Copenhagen, Denmark
[7]Biodiversity Research Centre, University of British Columbia, 2212 Main Mall, Vancouver V6T 1Z4, Canada
[8]Department of Agricultural and Biosystems Engineering, Iowa State University, 1201 Sukup Hall, Ames IA 50011, USA
[9]Department of Biology, Stanford University, Stanford CA 94305, USA
[10]Gladstone Institutes, San Francisco, CA 94158, USA

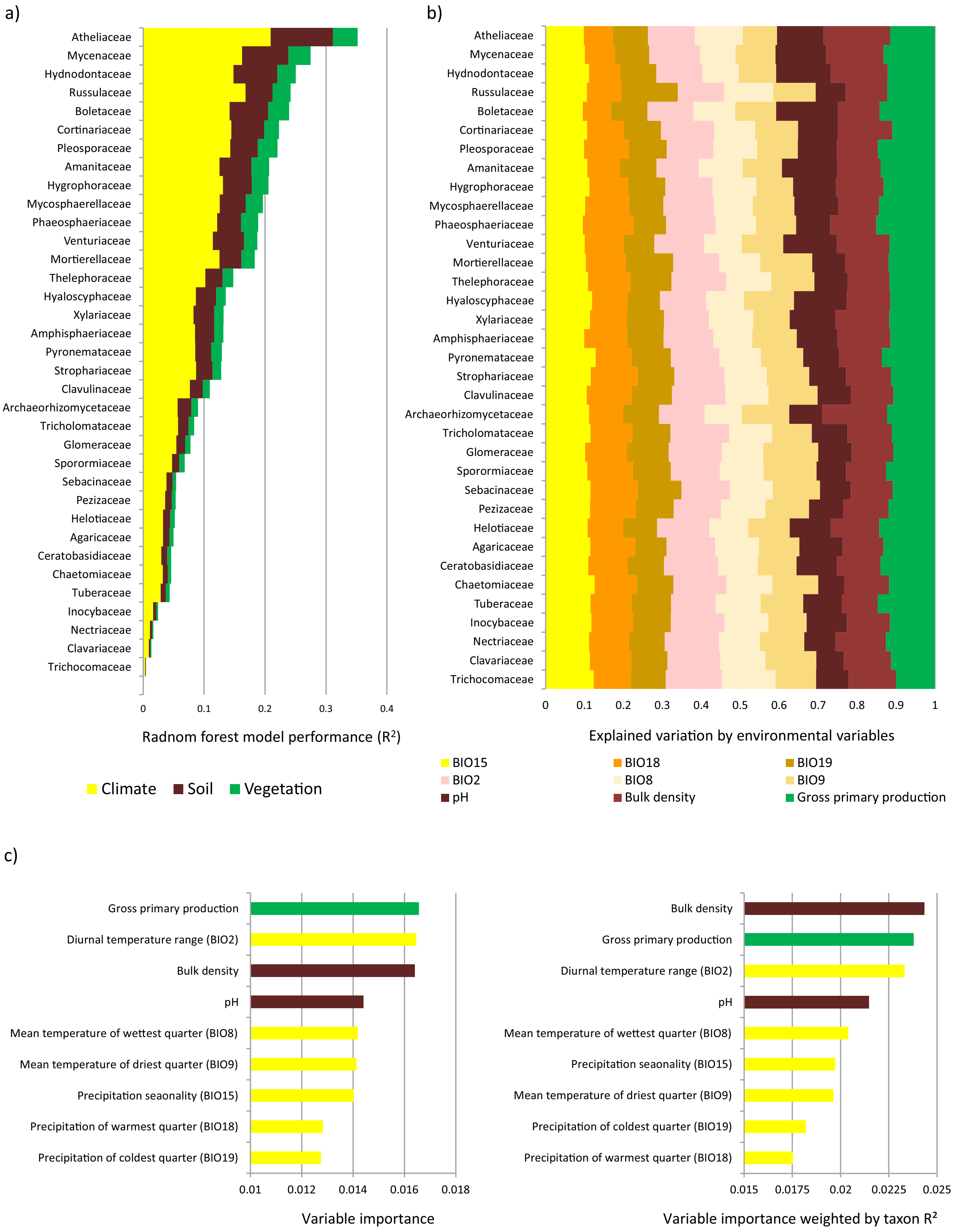

a)
Atheliaceae
Mycenaceae
Hydnodontaceae
Russulaceae
Boletaceae
Cortinariaceae
Pleosporaceae
Amanitaceae
Hygrophoraceae
Mycosphaerellaceae
Phaeosphaeriaceae
Venturiaceae
Mortierellaceae
Thelephoraceae
Hyaloscyphaceae
Xylariaceae
Amphisphaeriaceae
Pyronemataceae
Strophariaceae
Clavulinaceae
Archaeorhizomycetaceae
Tricholomataceae
Glomeraceae
Sporormiaceae
Sebacinaceae
Pezizaceae
Helotiaceae
Agaricaceae
Ceratobasidiaceae
Chaetomiaceae
Tuberaceae
Inocybaceae
Nectriaceae
Clavariaceae
Trichocomaceae
0
0.1
0.2
0.3
0.4
Radnom forest model performance (R^2)
Climate
Soil
Vegetation
b)
Atheliaceae
Mycenaceae
Hydnodontaceae
Russulaceae
Boletaceae
Cortinariaceae
Pleosporaceae
Amanitaceae
Hygrophoraceae
Mycosphaerellaceae
Phaeosphaeriaceae
Venturiaceae
Mortierellaceae
Thelephoraceae
Hyaloscyphaceae
Xylariaceae
Amphisphaeriaceae
Pyronemataceae
Strophariaceae
Clavulinaceae
Archaeorhizomycetaceae
Tricholomataceae
Glomeraceae
Sporormiaceae
Sebacinaceae
Pezizaceae
Helotiaceae
Agaricaceae
Ceratobasidiaceae
Chaetomiaceae
Tuberaceae
Inocybaceae
Nectriaceae
Clavariaceae
Trichocomaceae
0
0.1
0.2
0.3
0.4
0.5
0.6
0.7
0.8
0.9
1
Explained variation by environmental variables
BIO15
BIO18
BIO19
BIO2
BIO8
BIO9
pH
Bulk density
Gross primary production
c)
Gross primary production
Diurnal temperature range (BIO2)
Bulk density
pH
Mean temperature of wettest quarter (BIO8)
Mean temperature of driest quarter (BIO9)
Precipitation seaonality (BIO15)
Precipitation of warmest quarter (BIO18)
Precipitation of coldest quarter (BIO19)
0.01
0.012
0.014
0.016
0.018
Variable importance
Bulk density
Gross primary production
Diurnal temperature range (BIO2)
pH
Mean temperature of wettest quarter (BIO8)
Precipitation seaonality (BIO15)
Mean temperature of driest quarter (BIO9)
Precipitation of coldest quarter (BIO19)
Precipitation of warmest quarter (BIO18)
0.015
0.0175
0.02
0.0225
0.025
Variable importance weighted by taxon R^2

Supplementary Figure 1: Environmental variables explaining the global distribution of the most frequent fungal families. **a,** Random forest model performance for 35 most frequent fungal families where $R^2 > 0$. **b,** Contribution of climatic, soil and vegetation variable categories to the variation explained by the complete random forest model for each fungal family. **c,** Importance of individual environmental variables across models for most frequent fungal families showing raw variable importance and variable importance weighted by out-of-bag R^2 for each taxon.

Agaricaceae

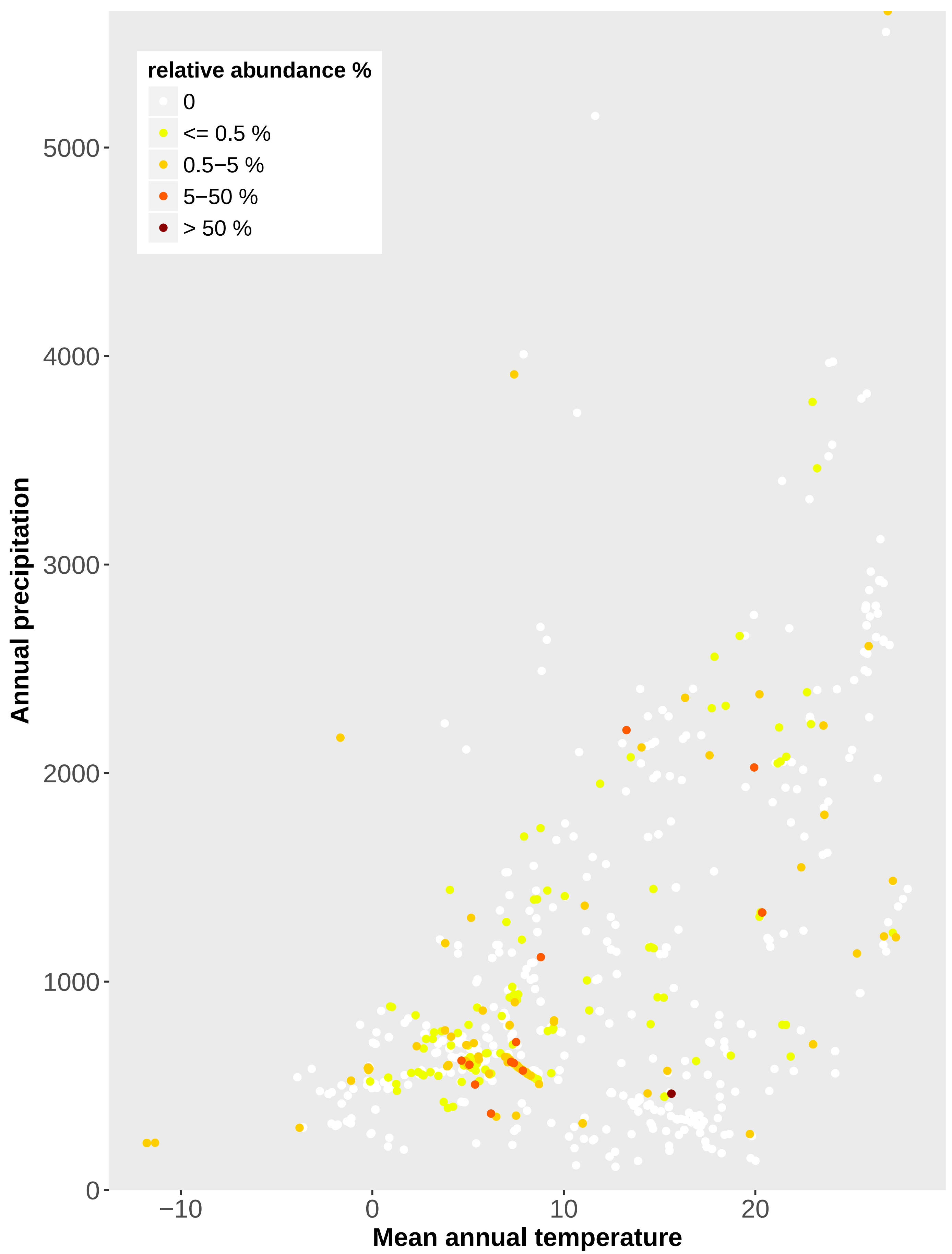

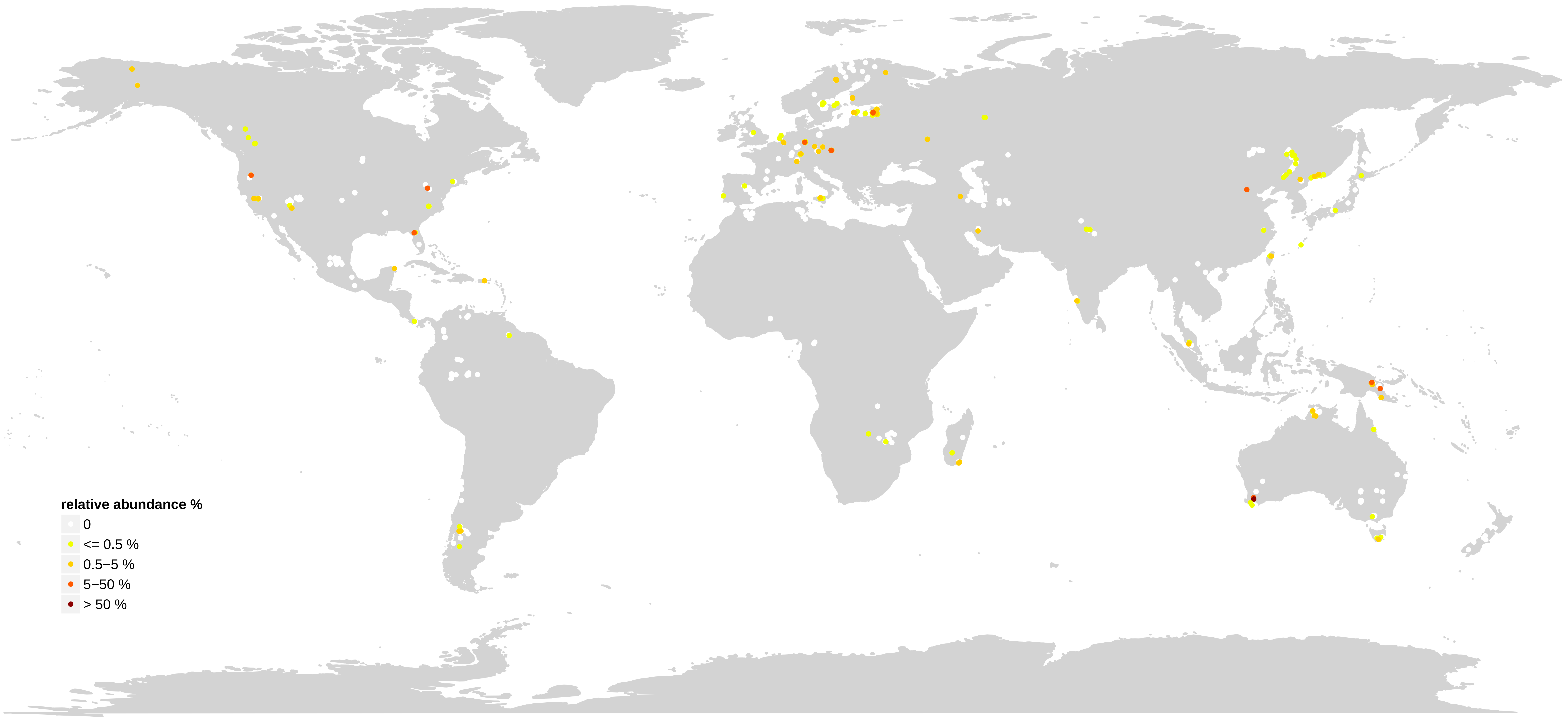

Amanitaceae

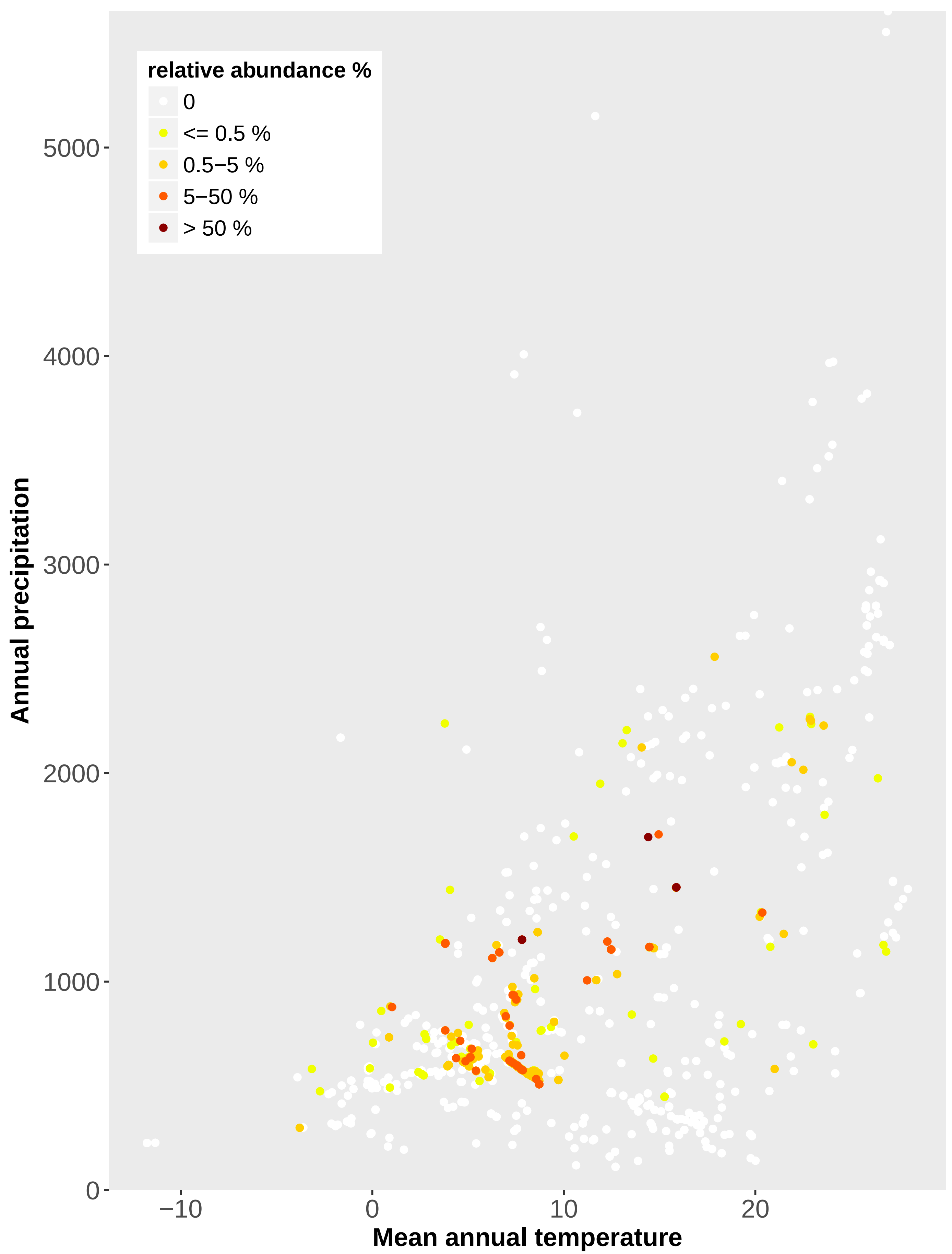

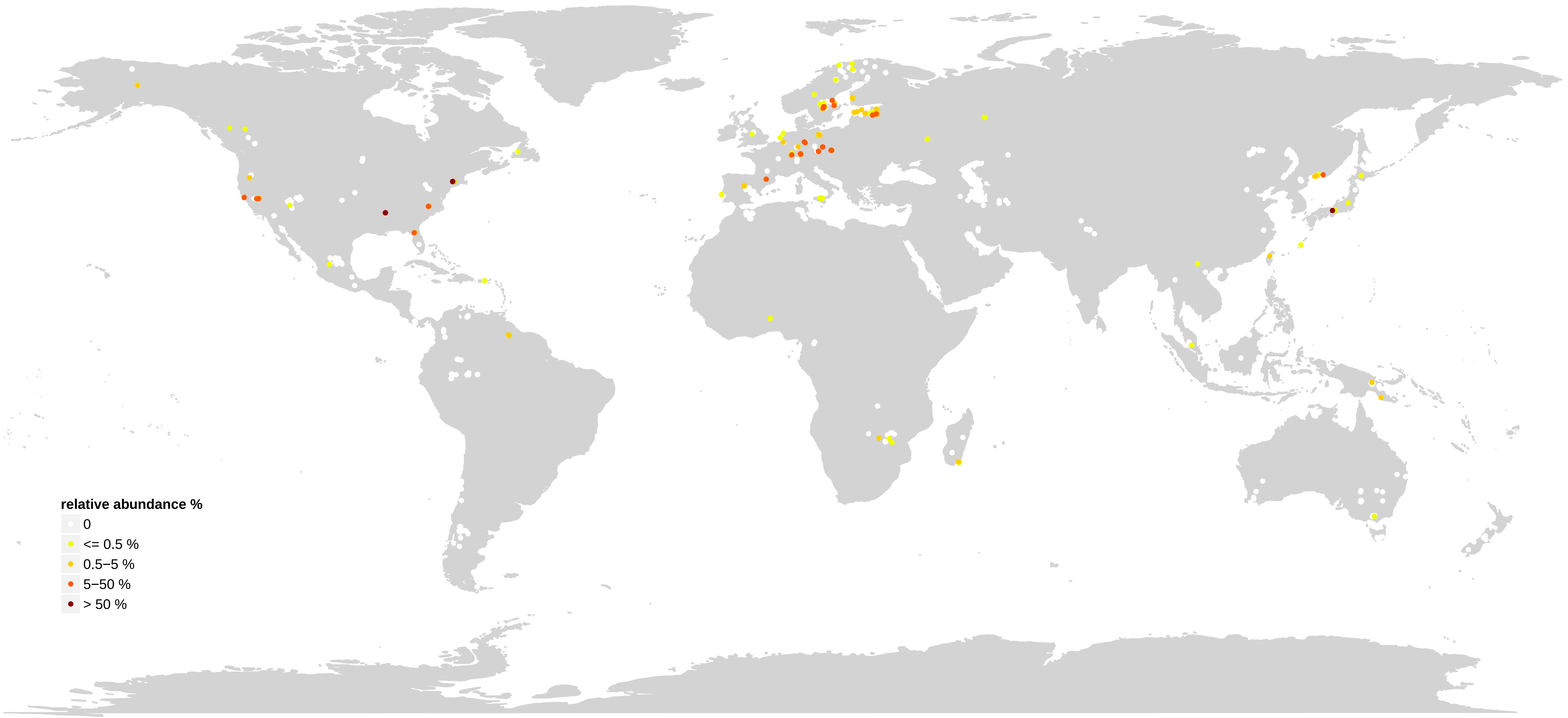

Amphisphaeriaceae

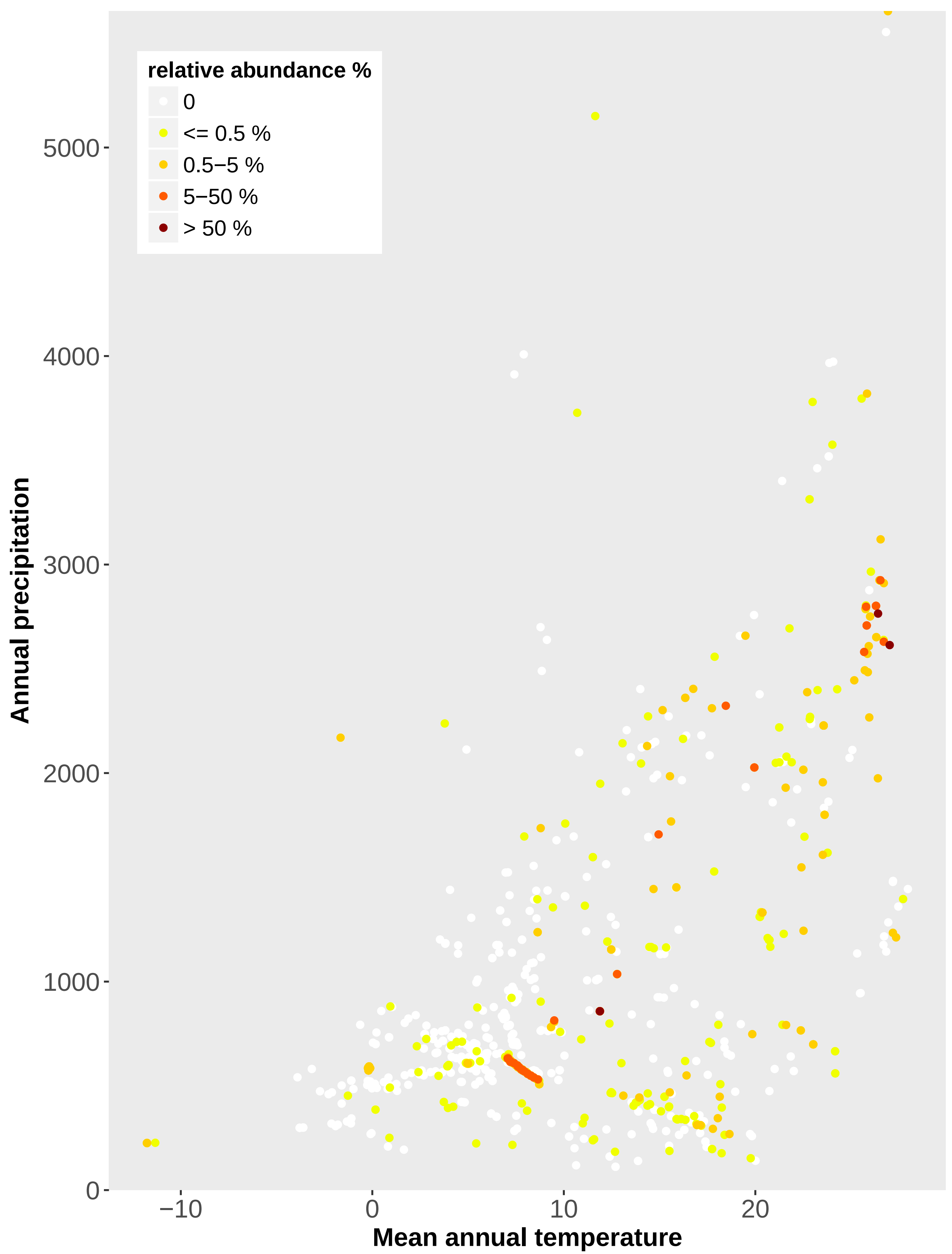

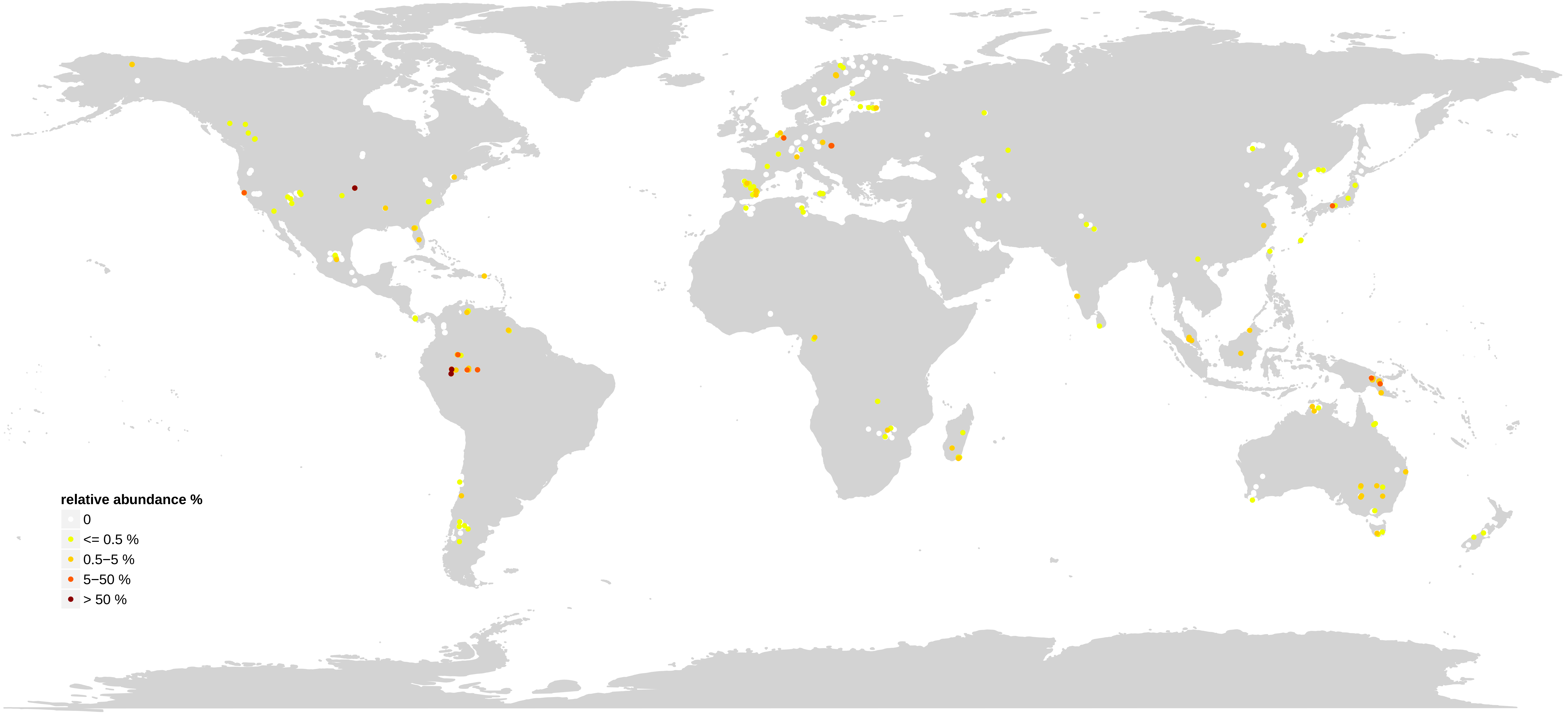

Archaeorhizomycetaceae

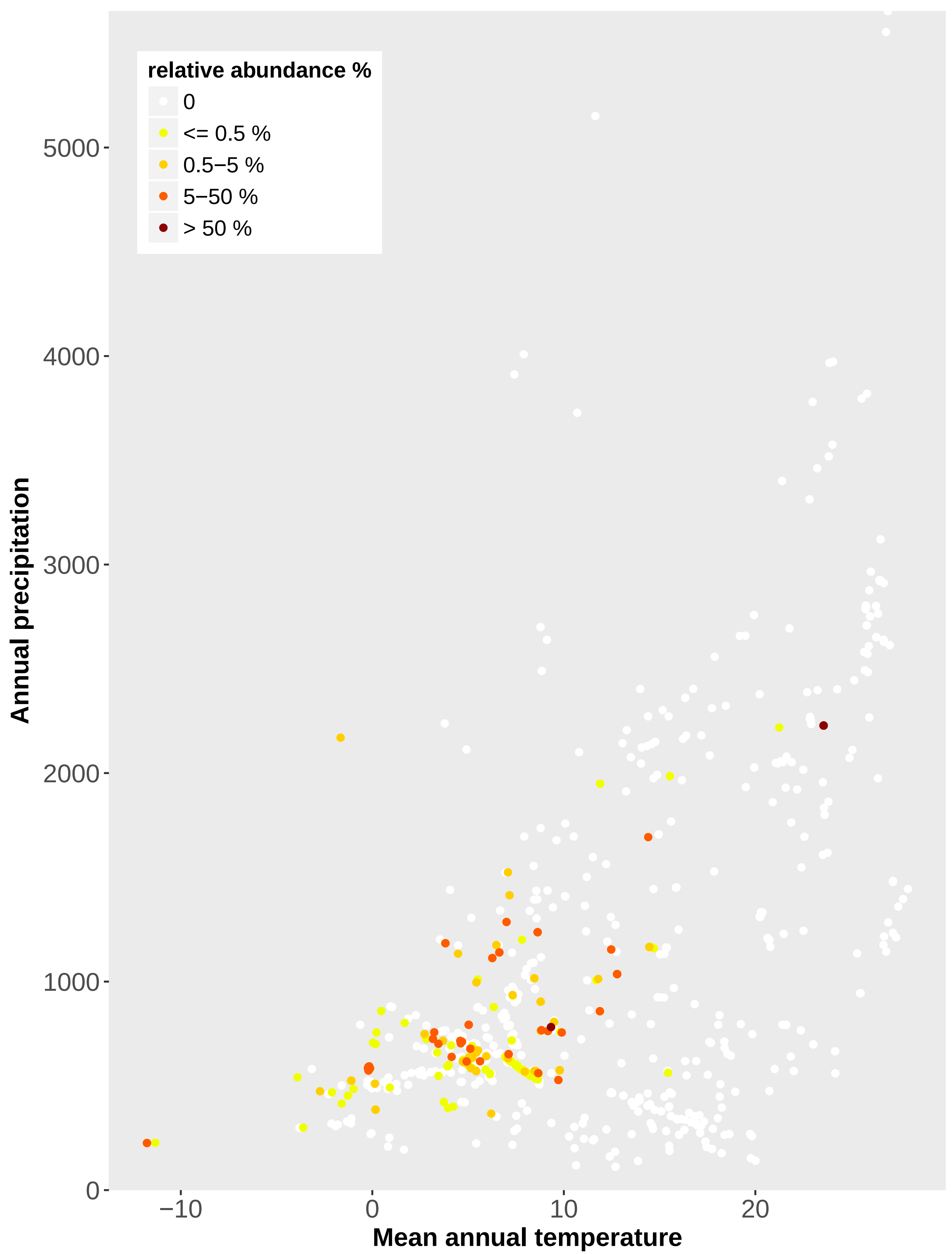

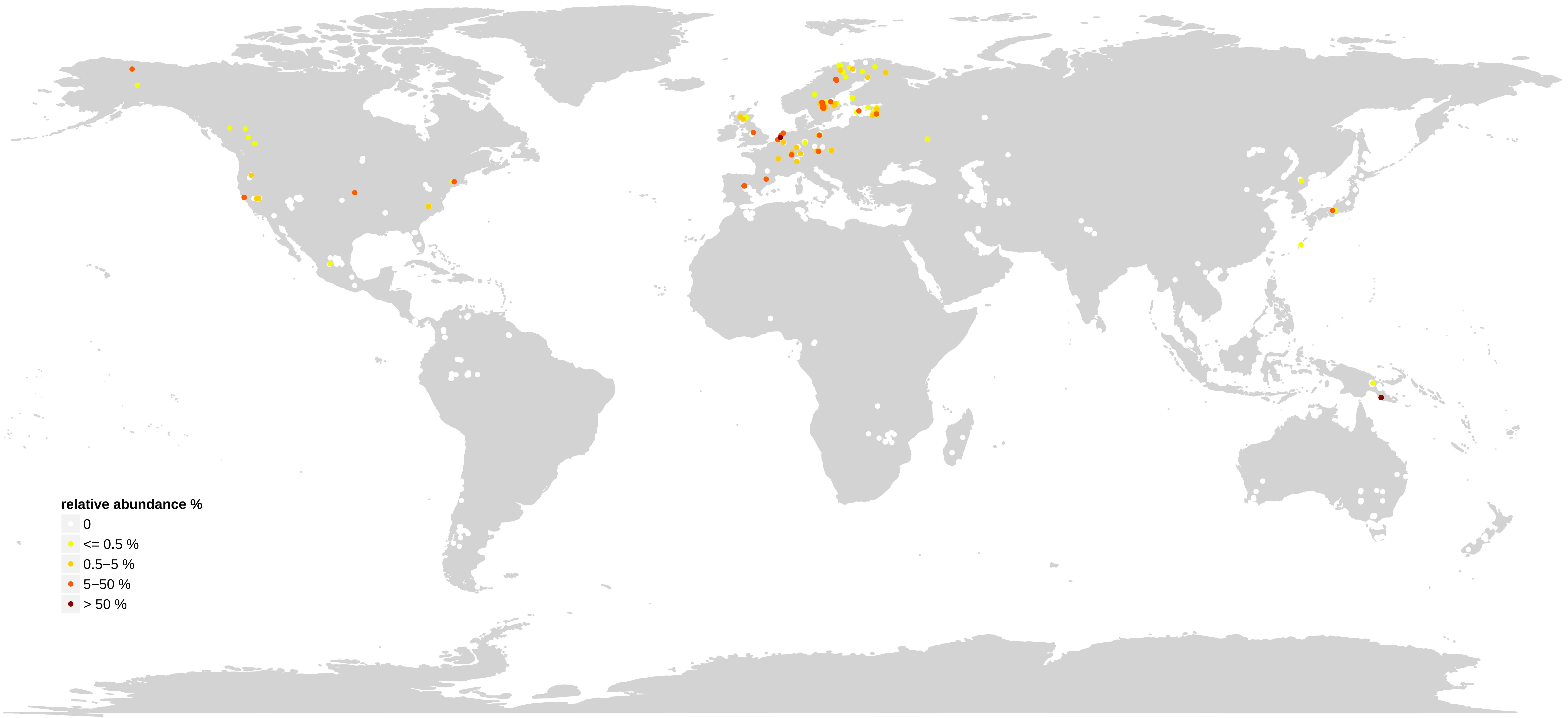

Atheliaceae

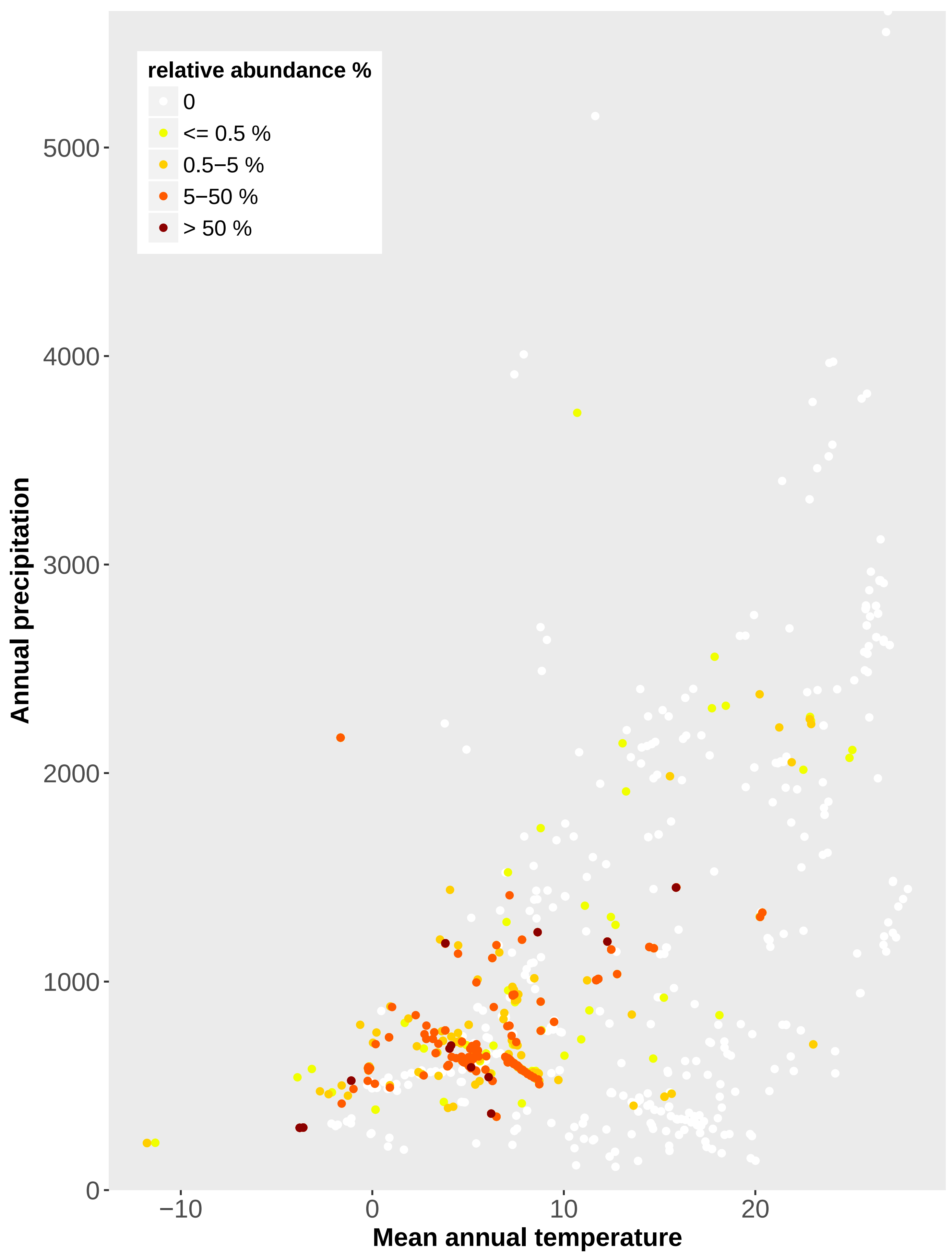

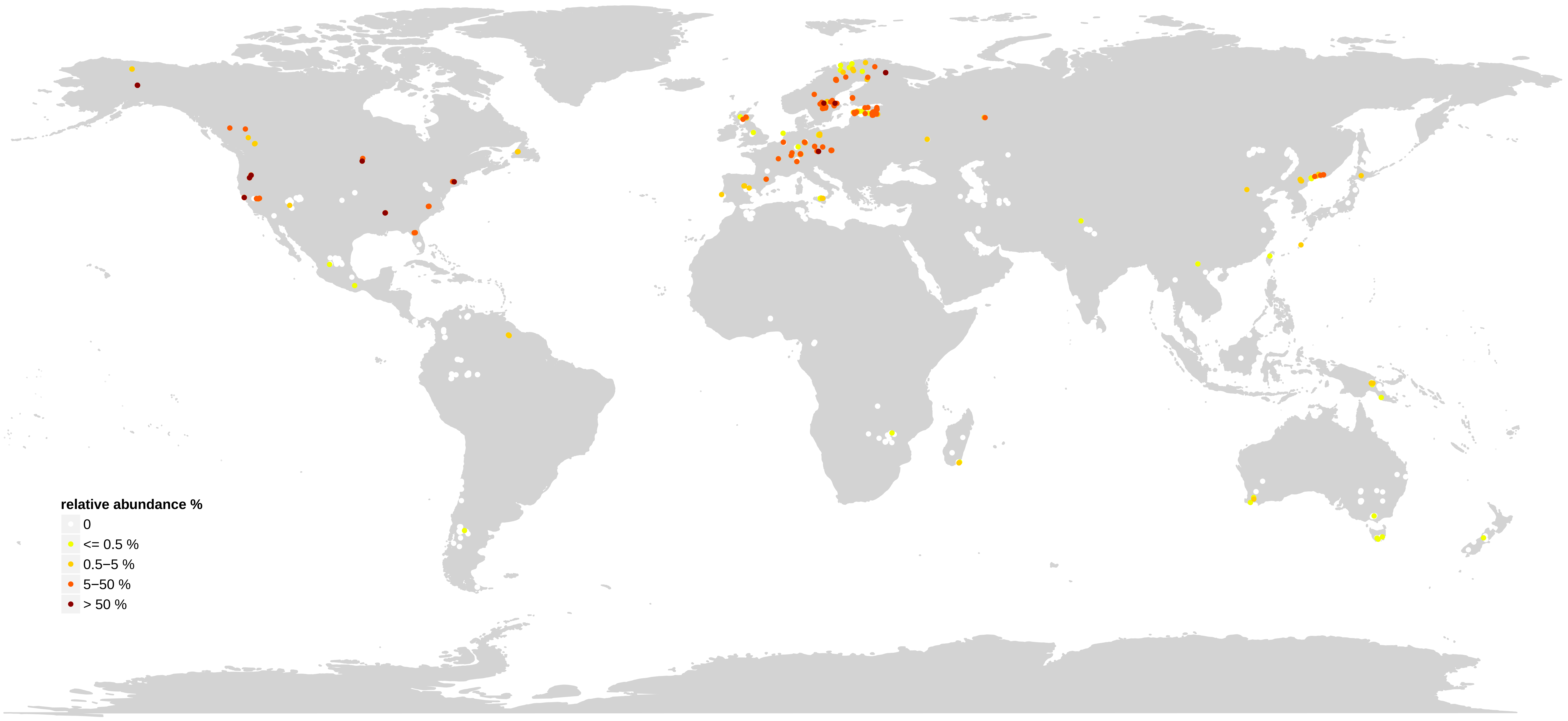

Boletaceae

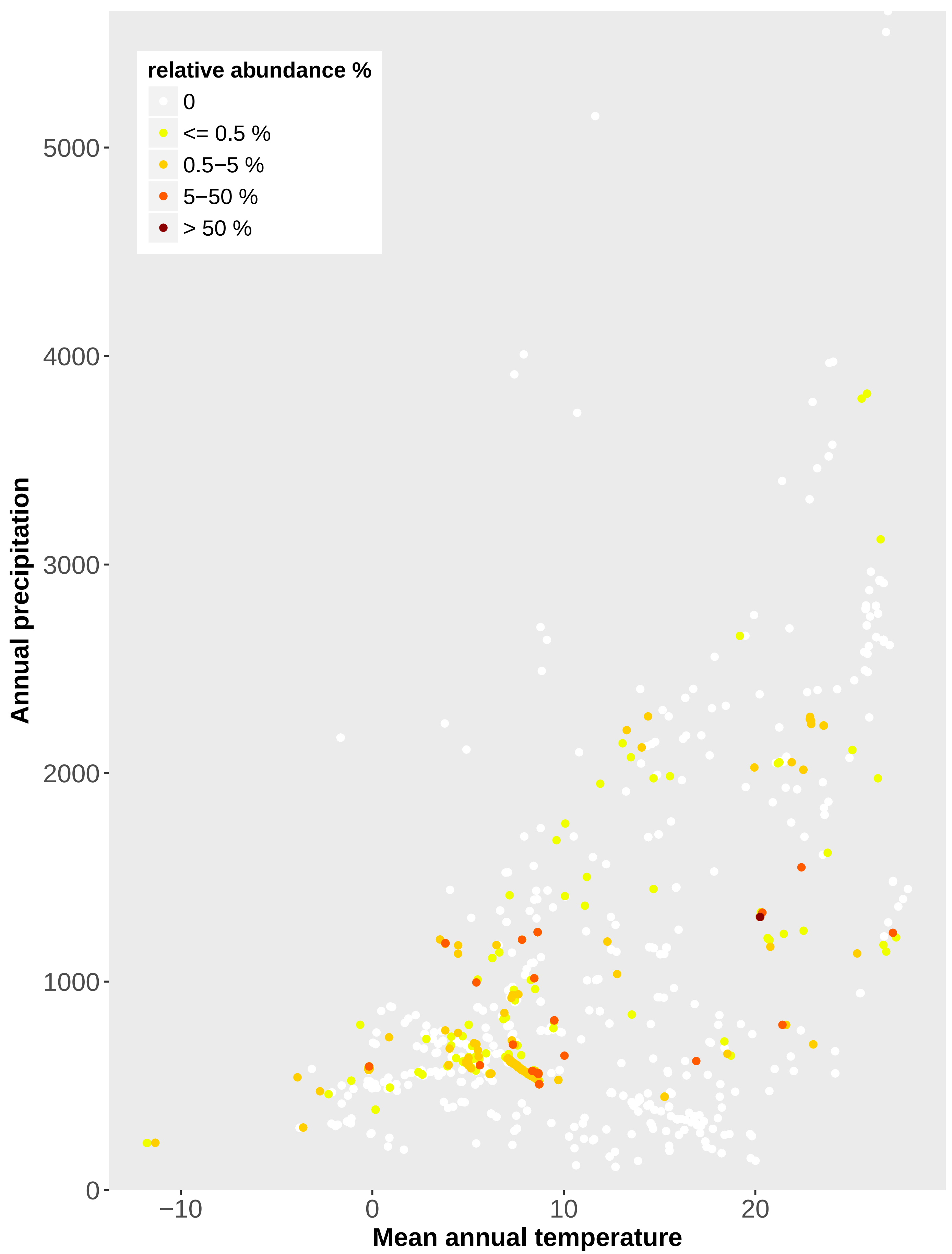

relative abundance %
0
<= 0.5 %
0.5−5 %
5−50 %
> 50 %
Annual precipitation
0
1000
2000
3000
4000
5000
Mean annual temperature
−10
0
10
20

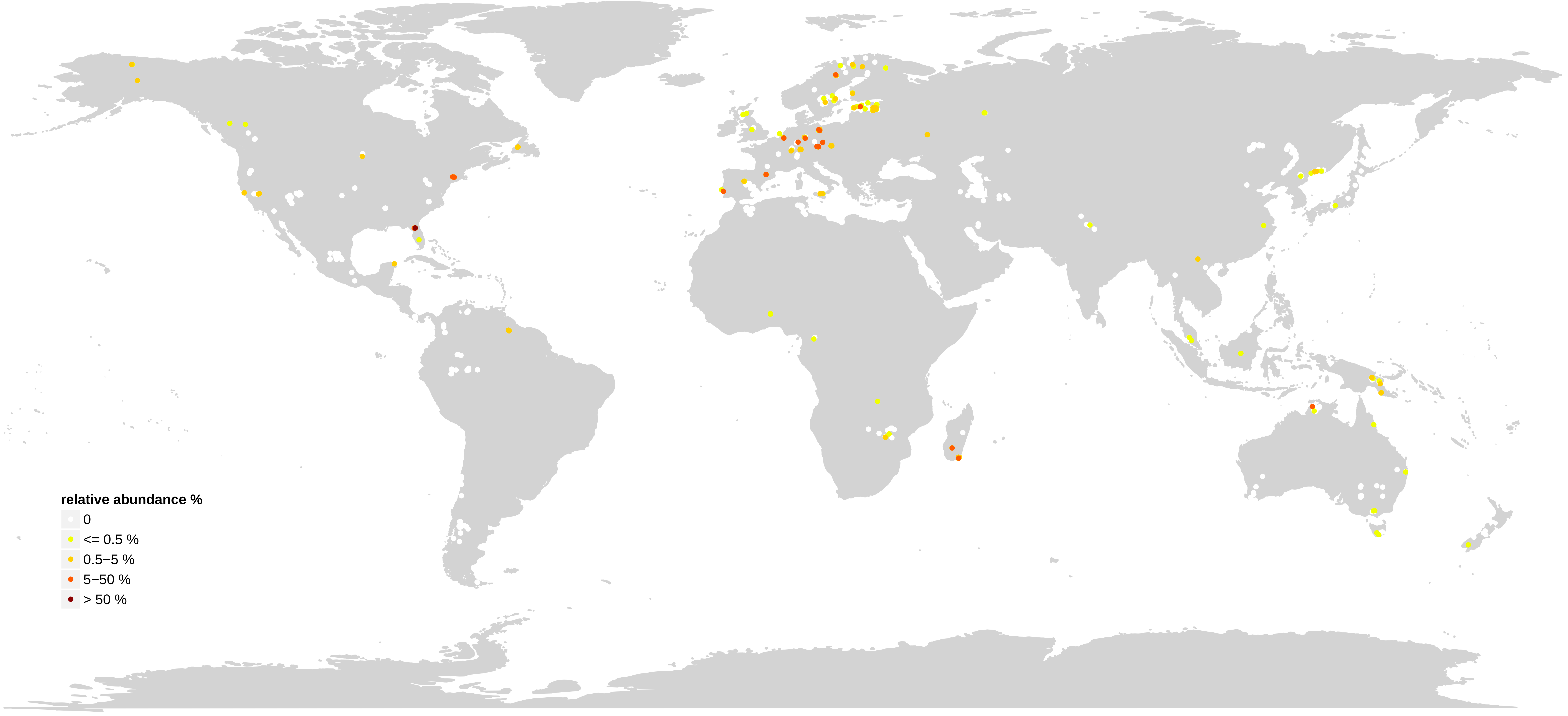

relative abundance %
0
<= 0.5 %
0.5−5 %
5−50 %
> 50 %

Ceratobasidiaceae

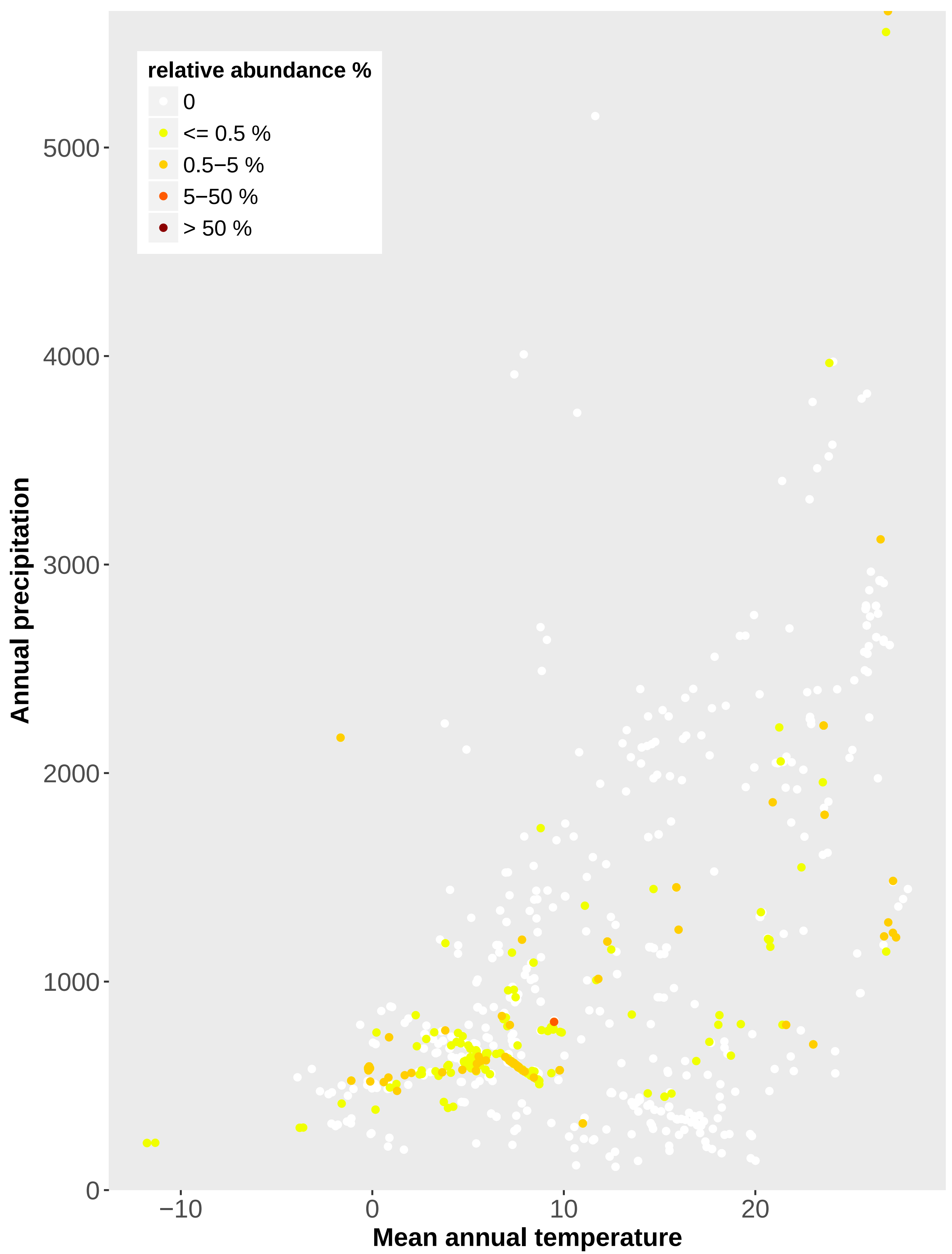

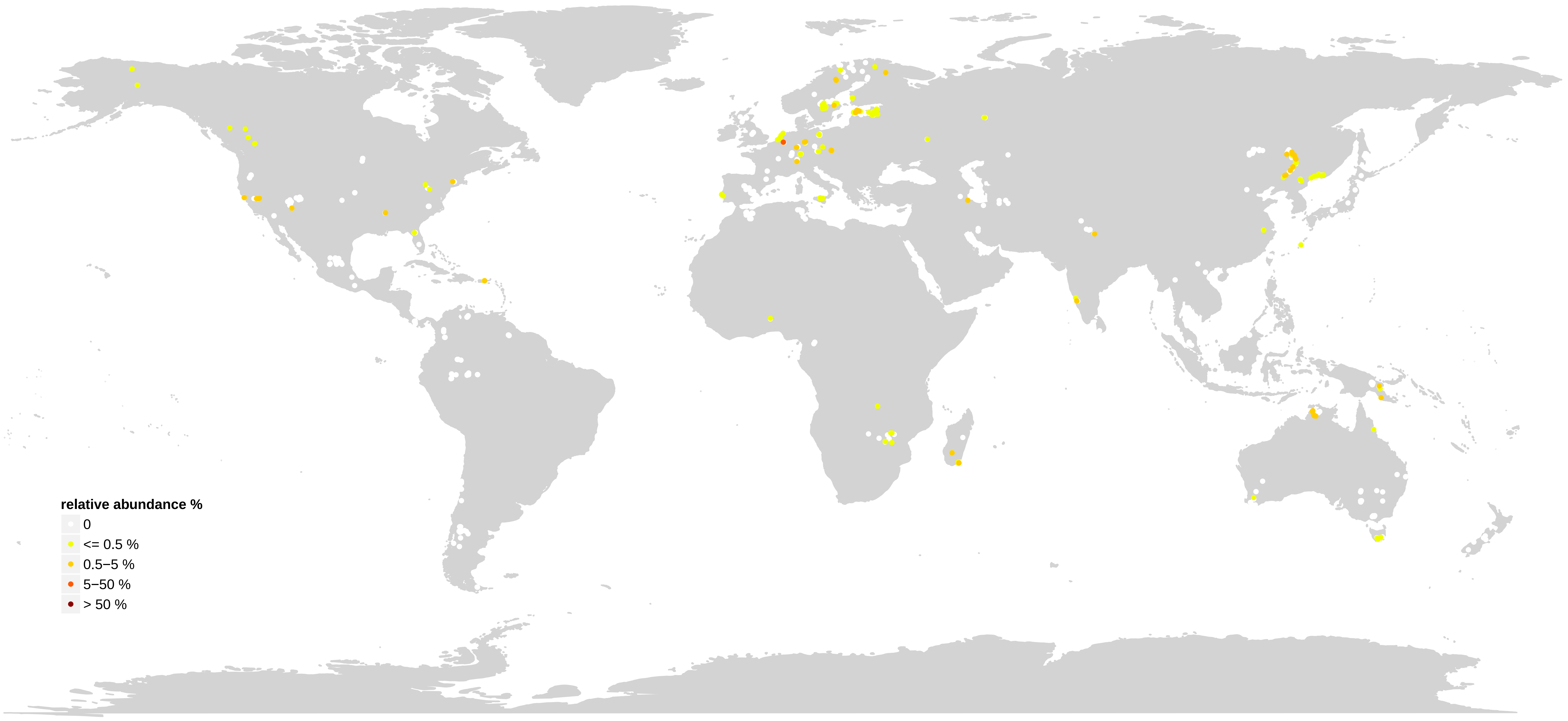

Chaetomiaceae

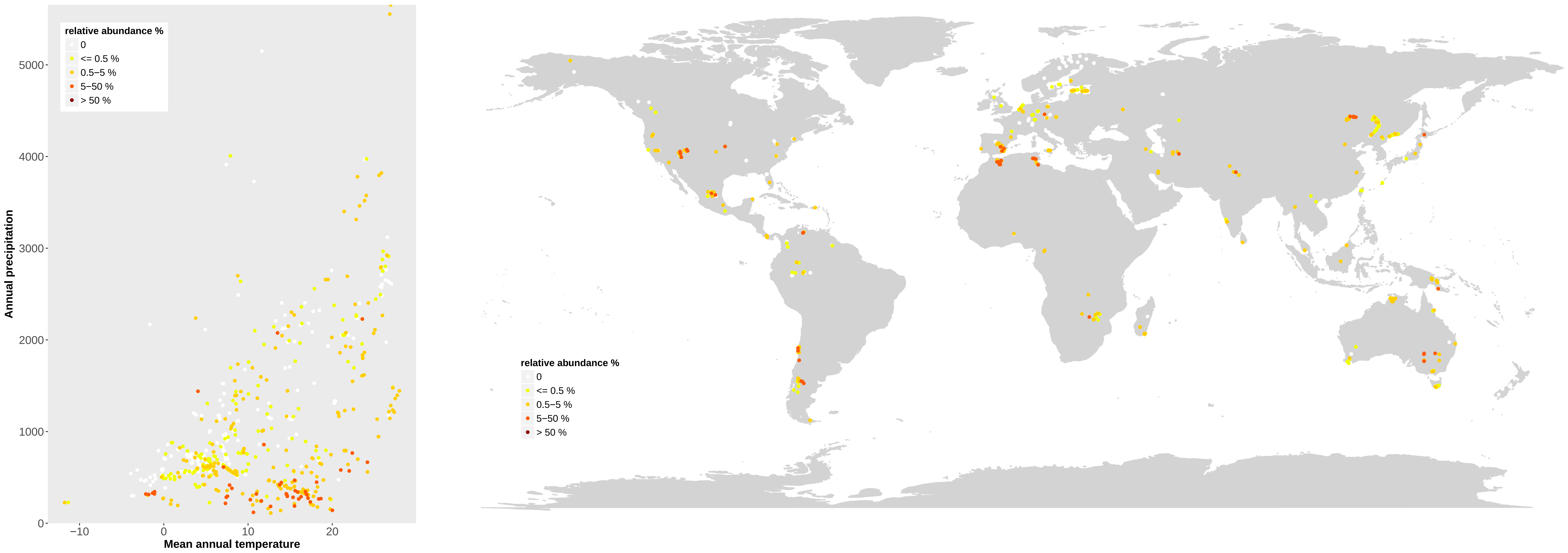

Clavariaceae

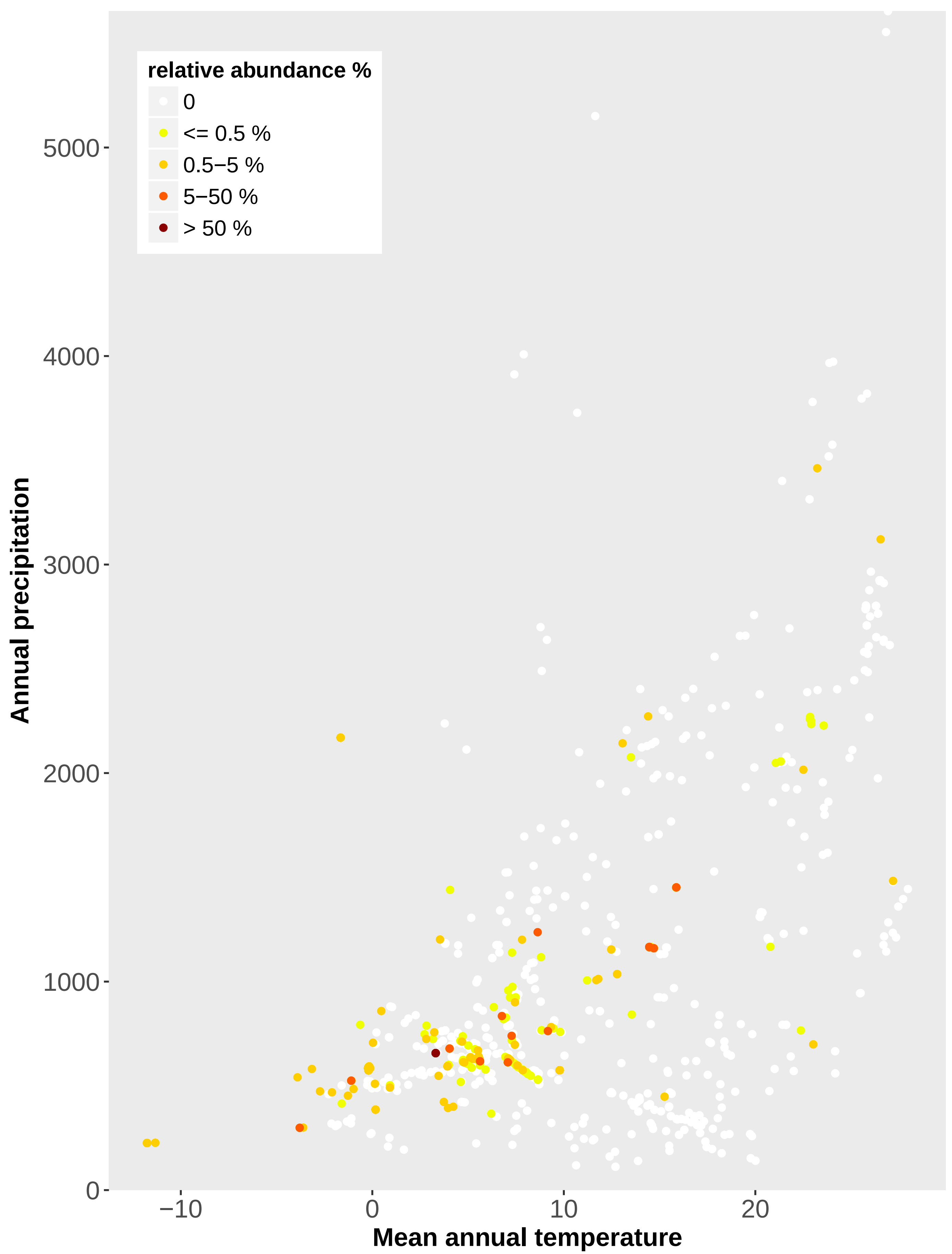

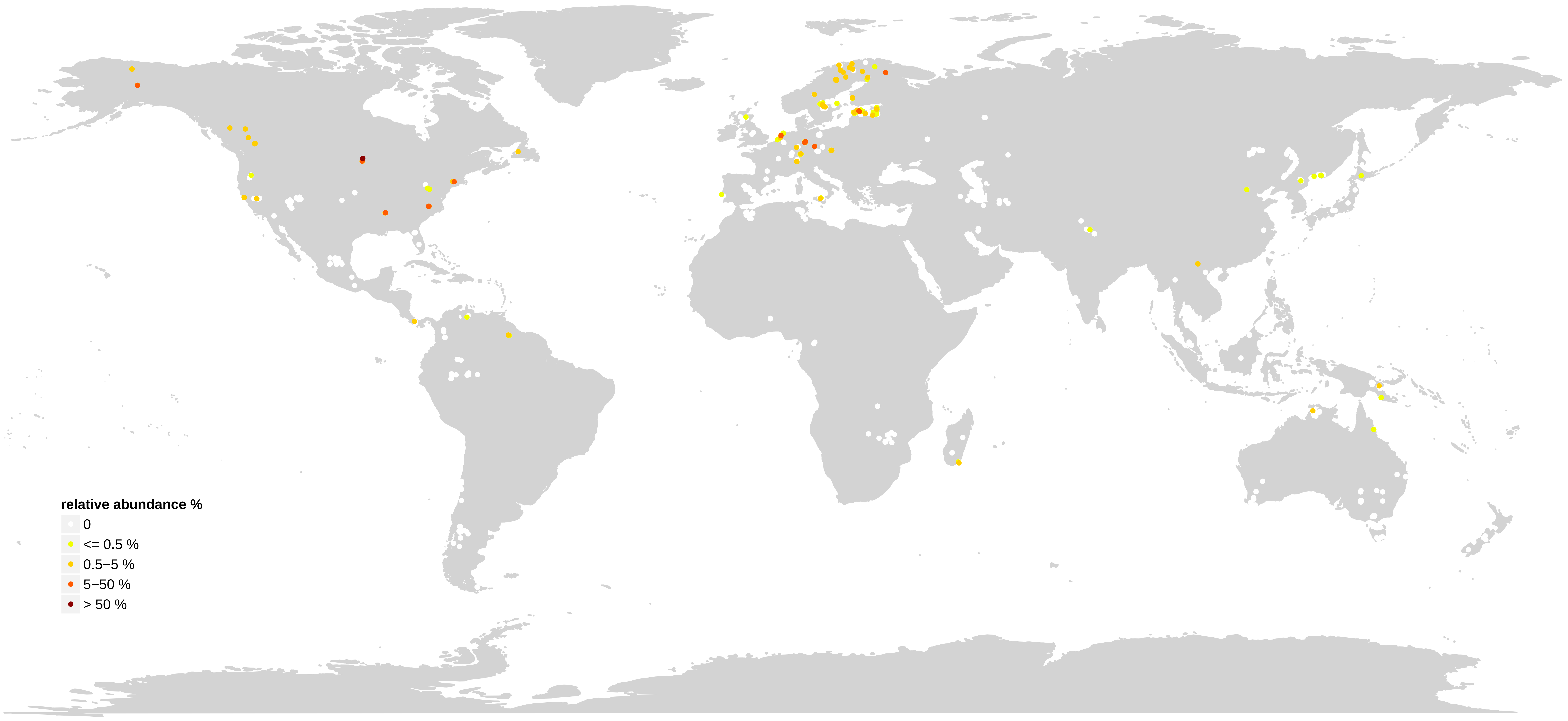

Clavulinaceae

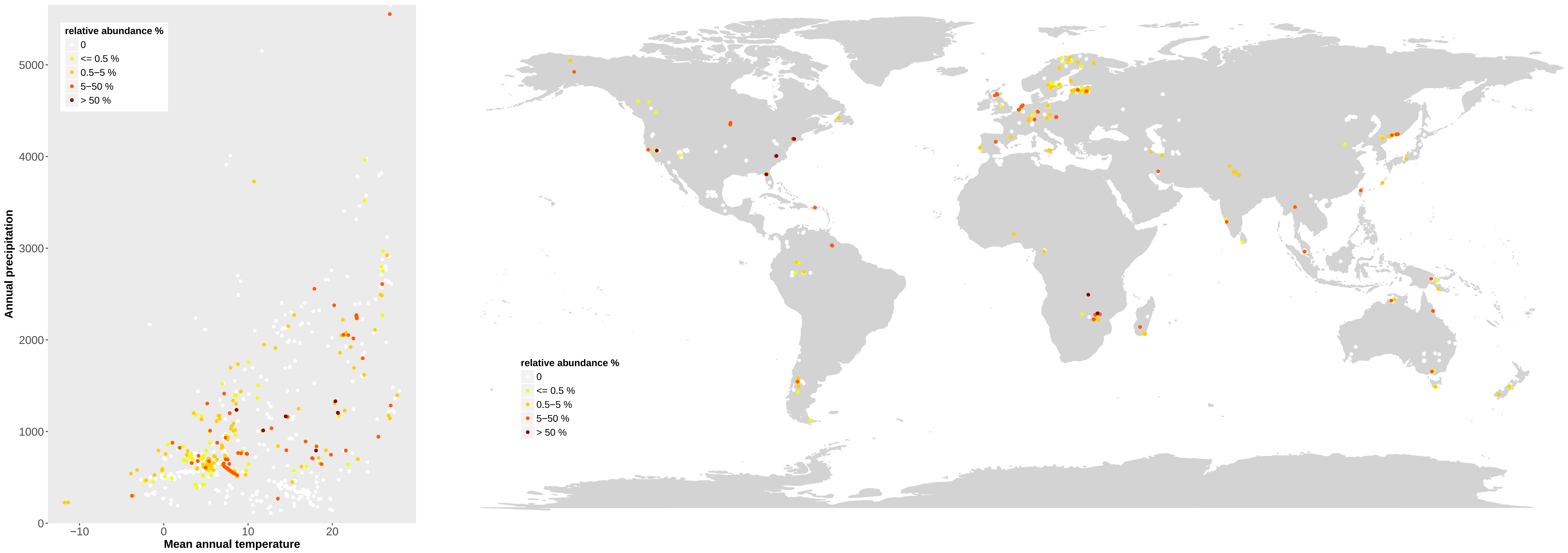

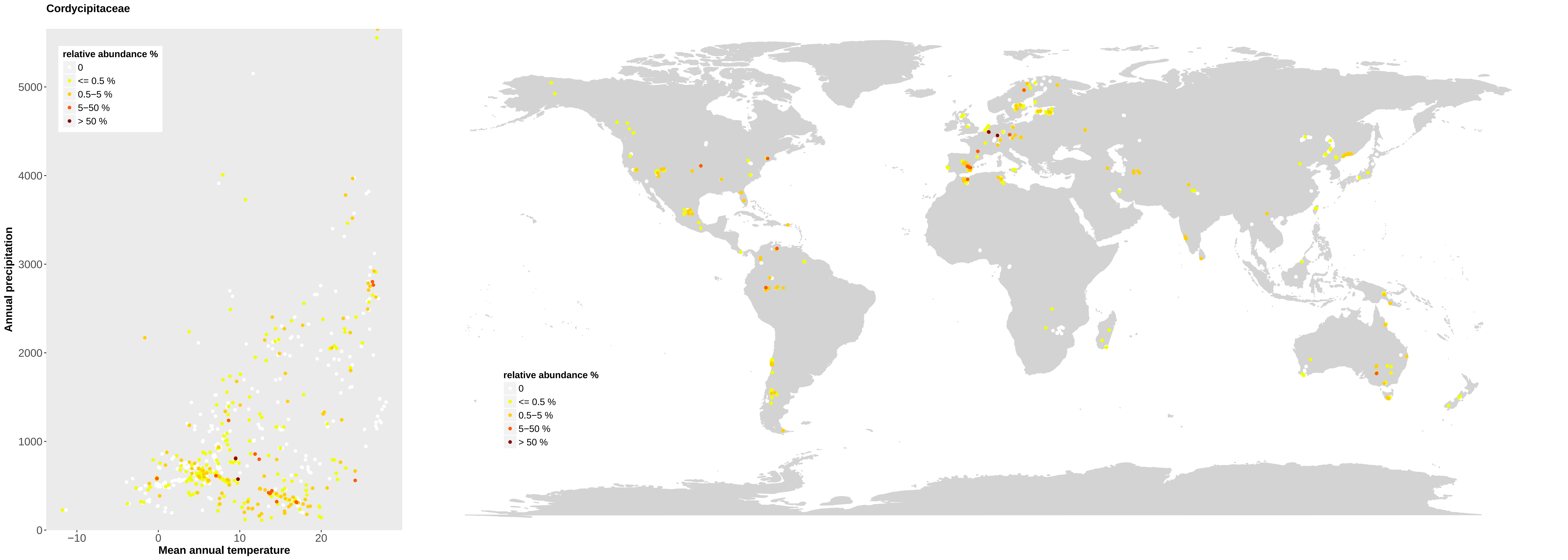
Cordycipitaceae
relative abundance %
0
<= 0.5 %
0.5–5 %
5–50 %
> 50 %
Annual precipitation
0
1000
2000
3000
4000
5000
−10
0
10
20
Mean annual temperature
relative abundance %
0
<= 0.5 %
0.5–5 %
5–50 %
> 50 %

Cortinariaceae

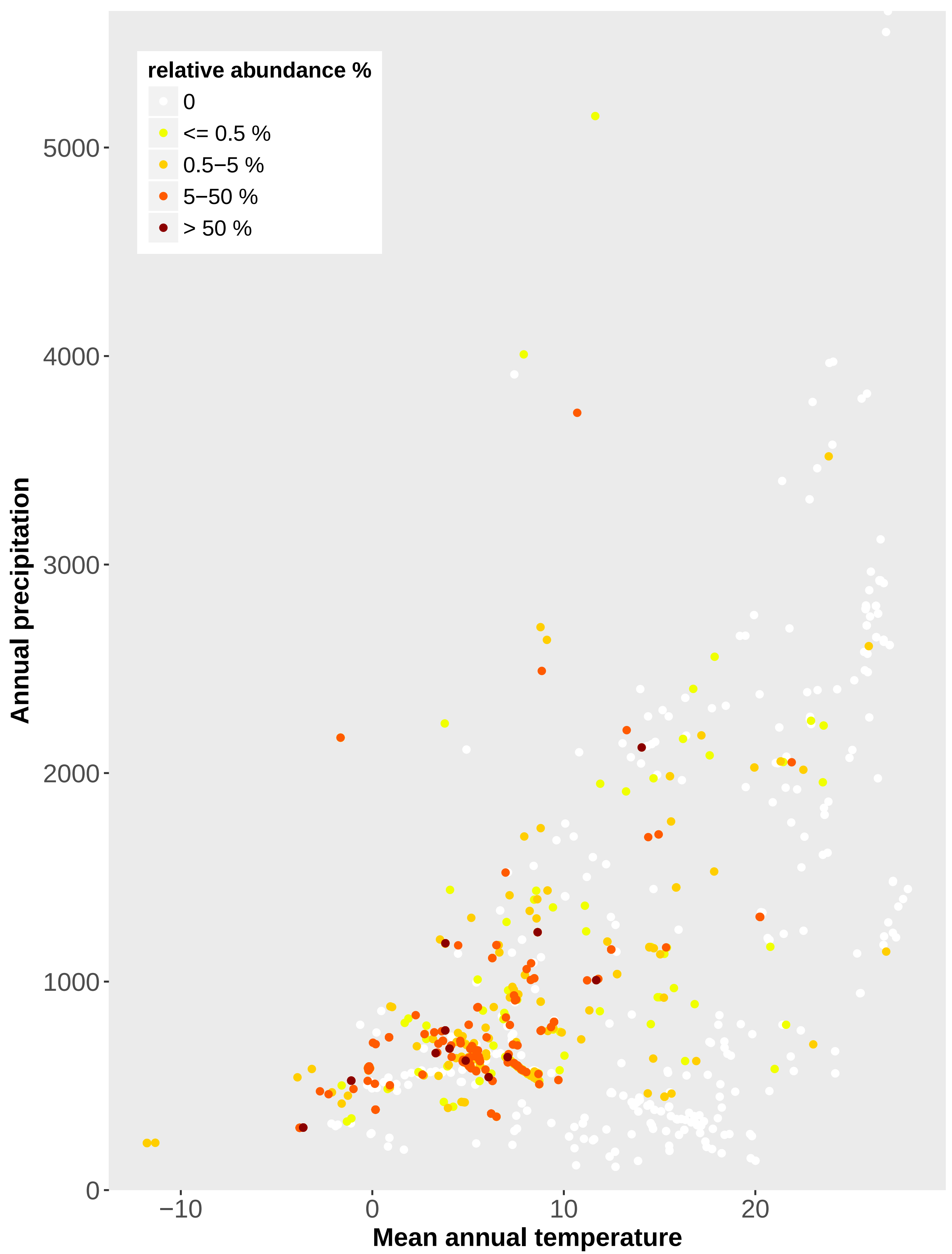

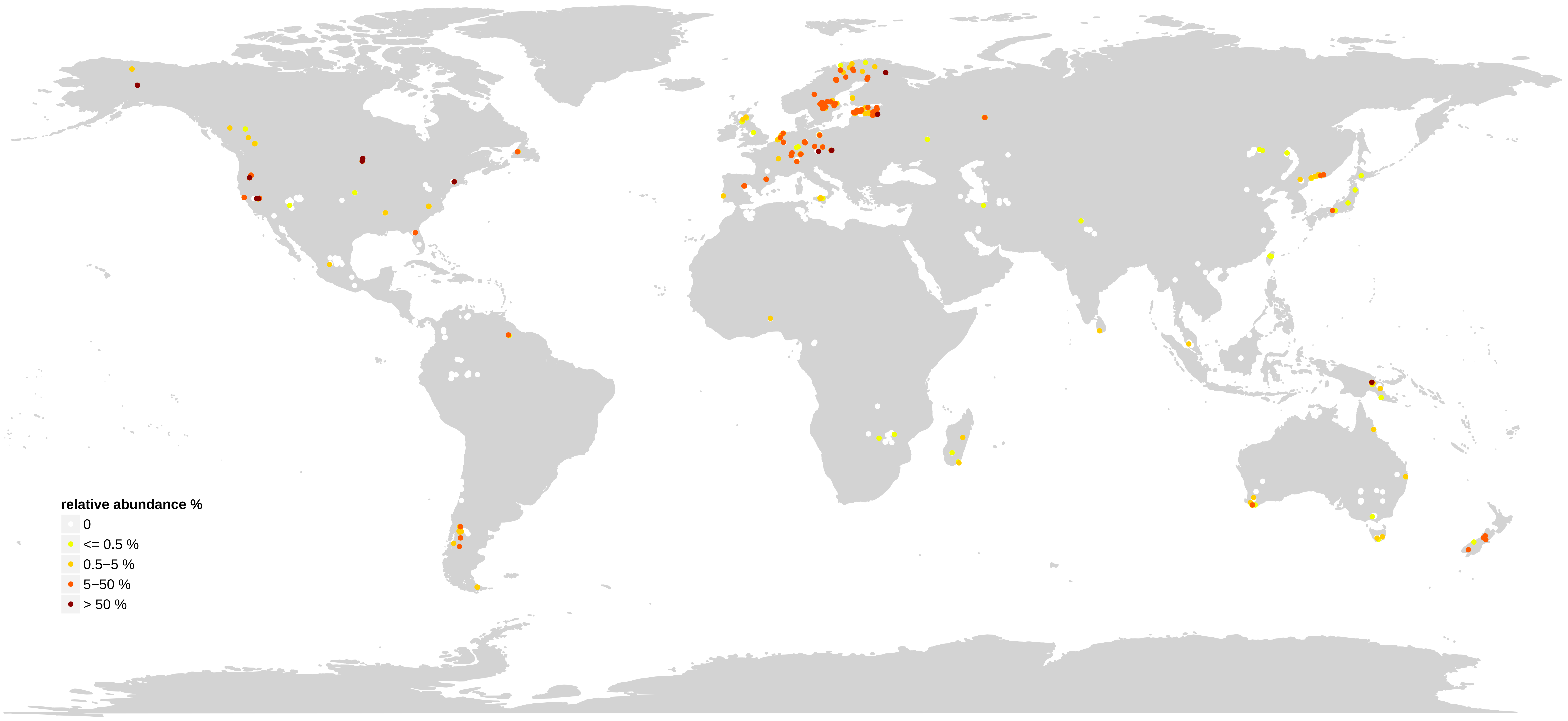

Entolomataceae

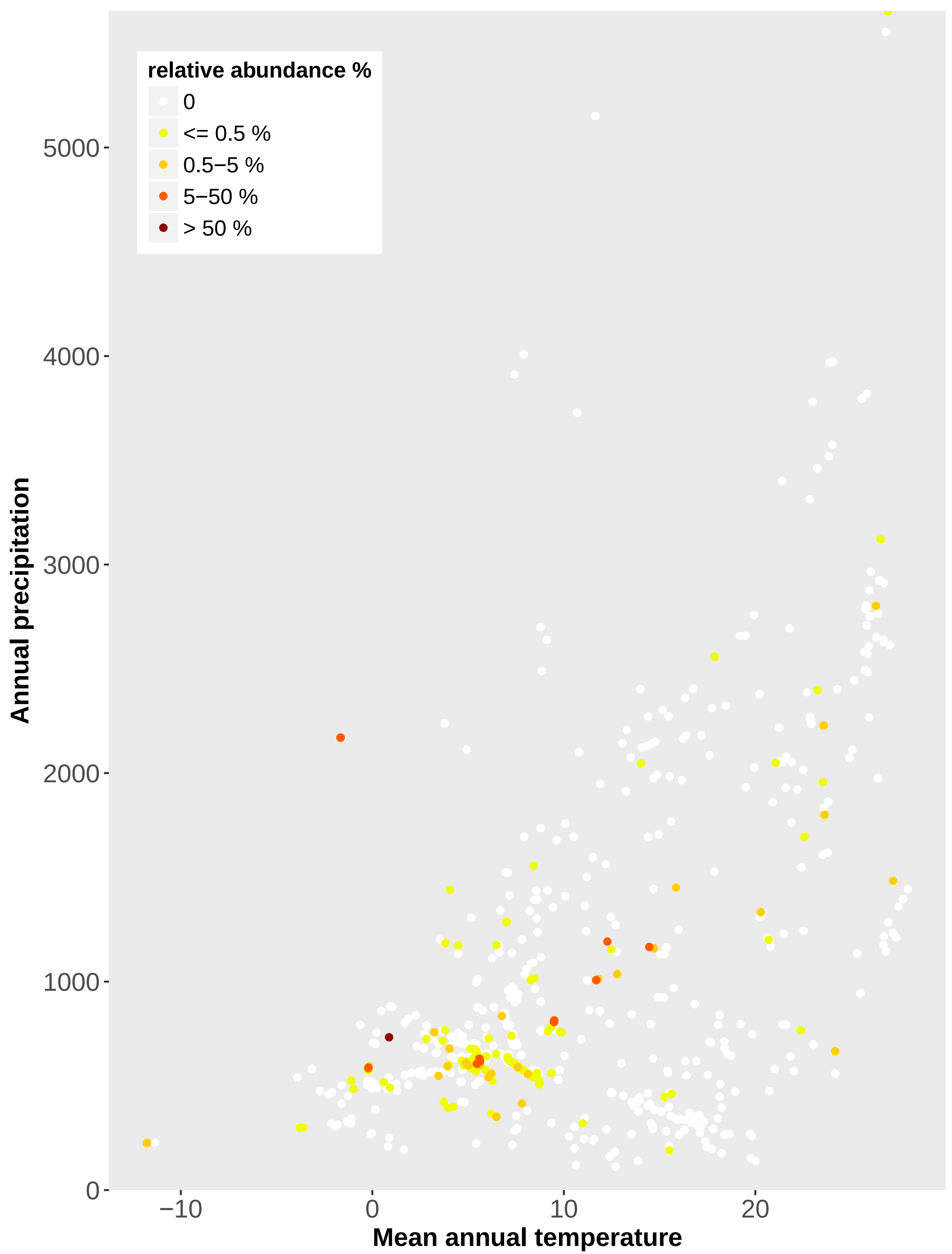

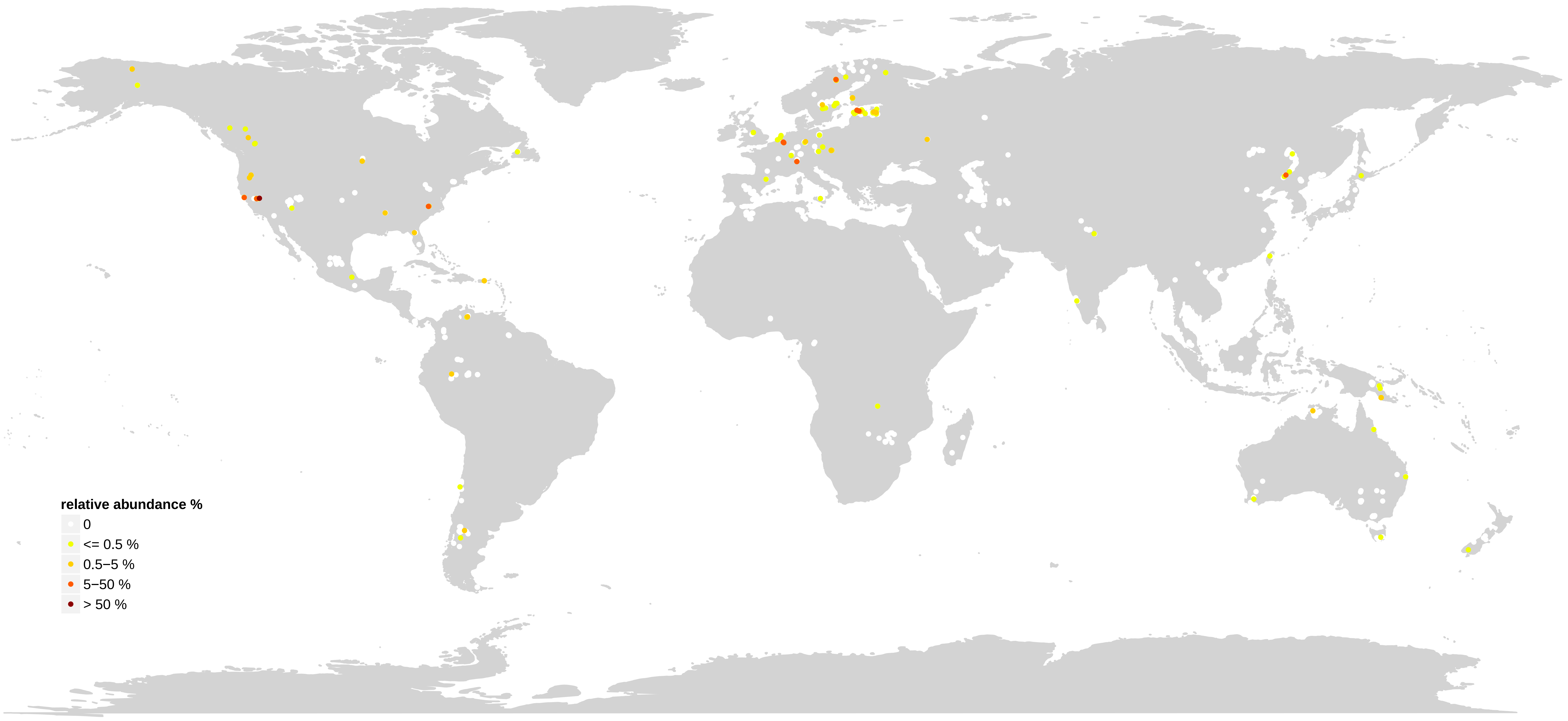

Glomeraceae

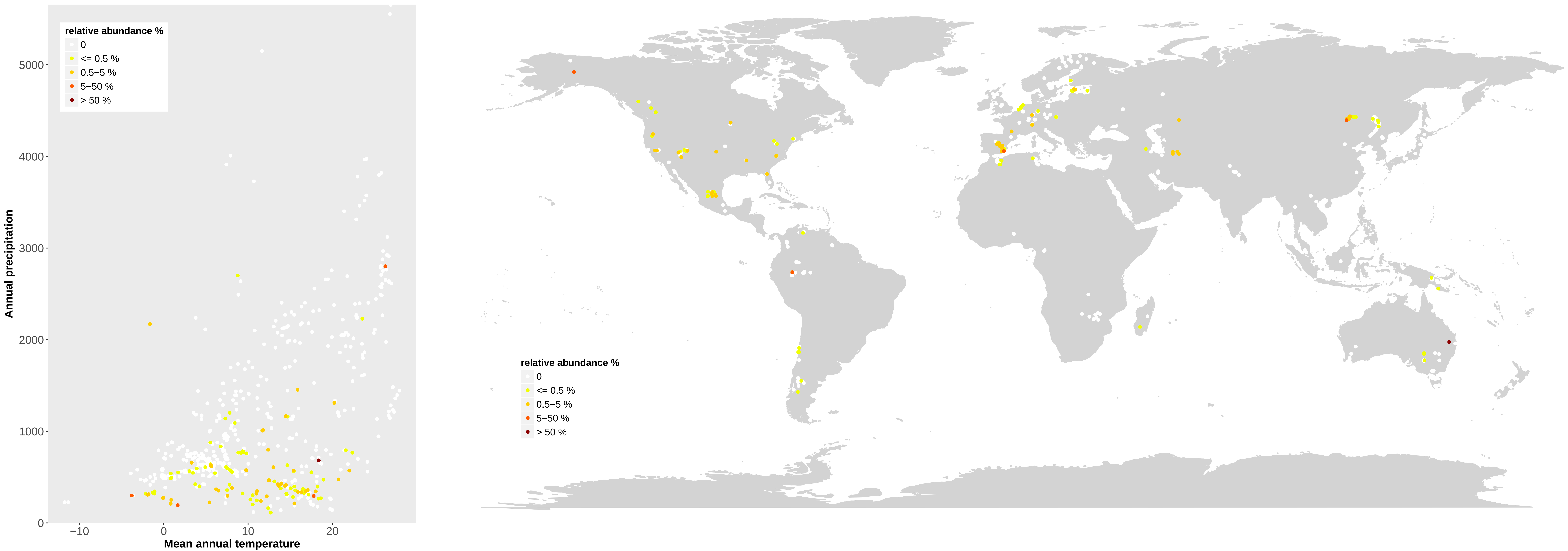

Helotiaceae

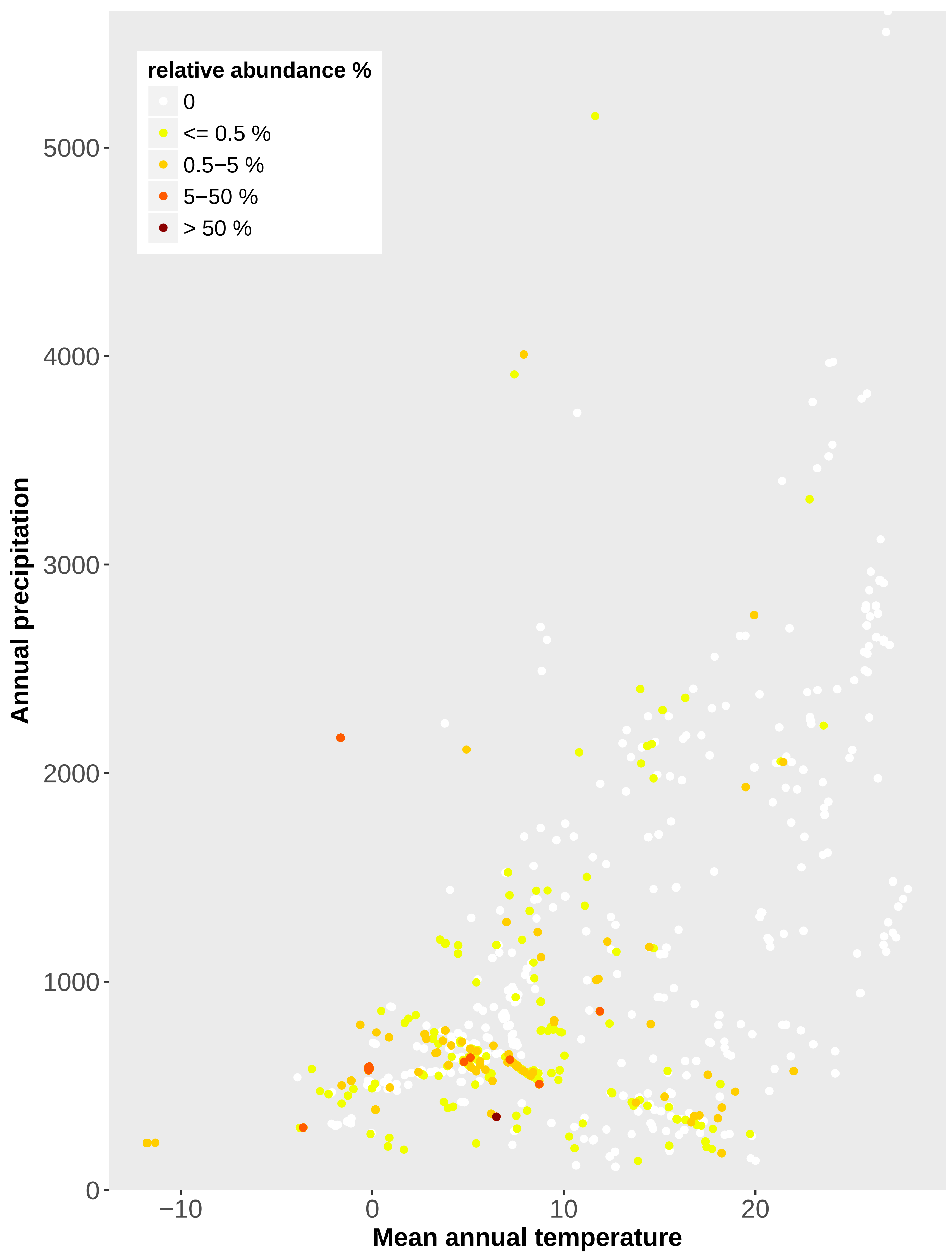

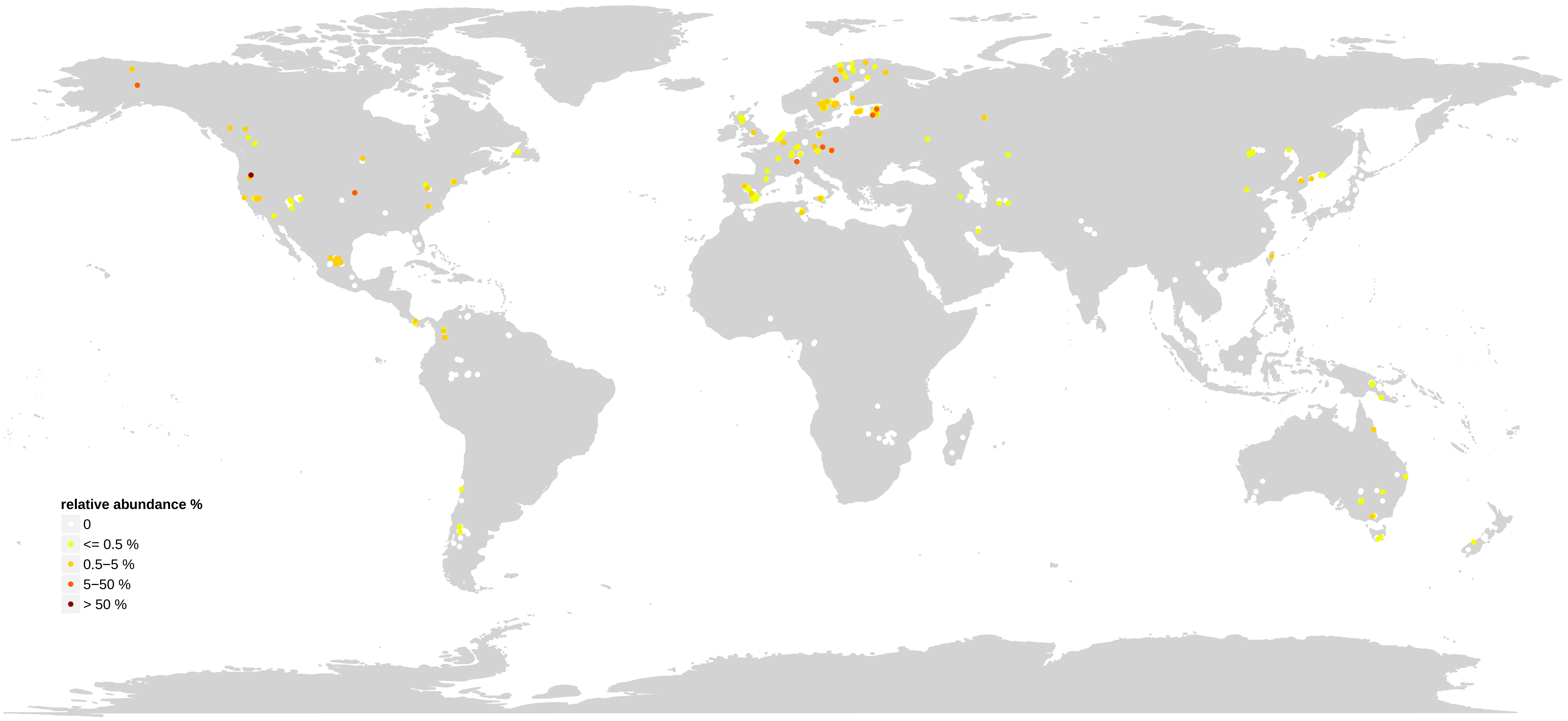

Helotiales_fam_Incertae_sedis

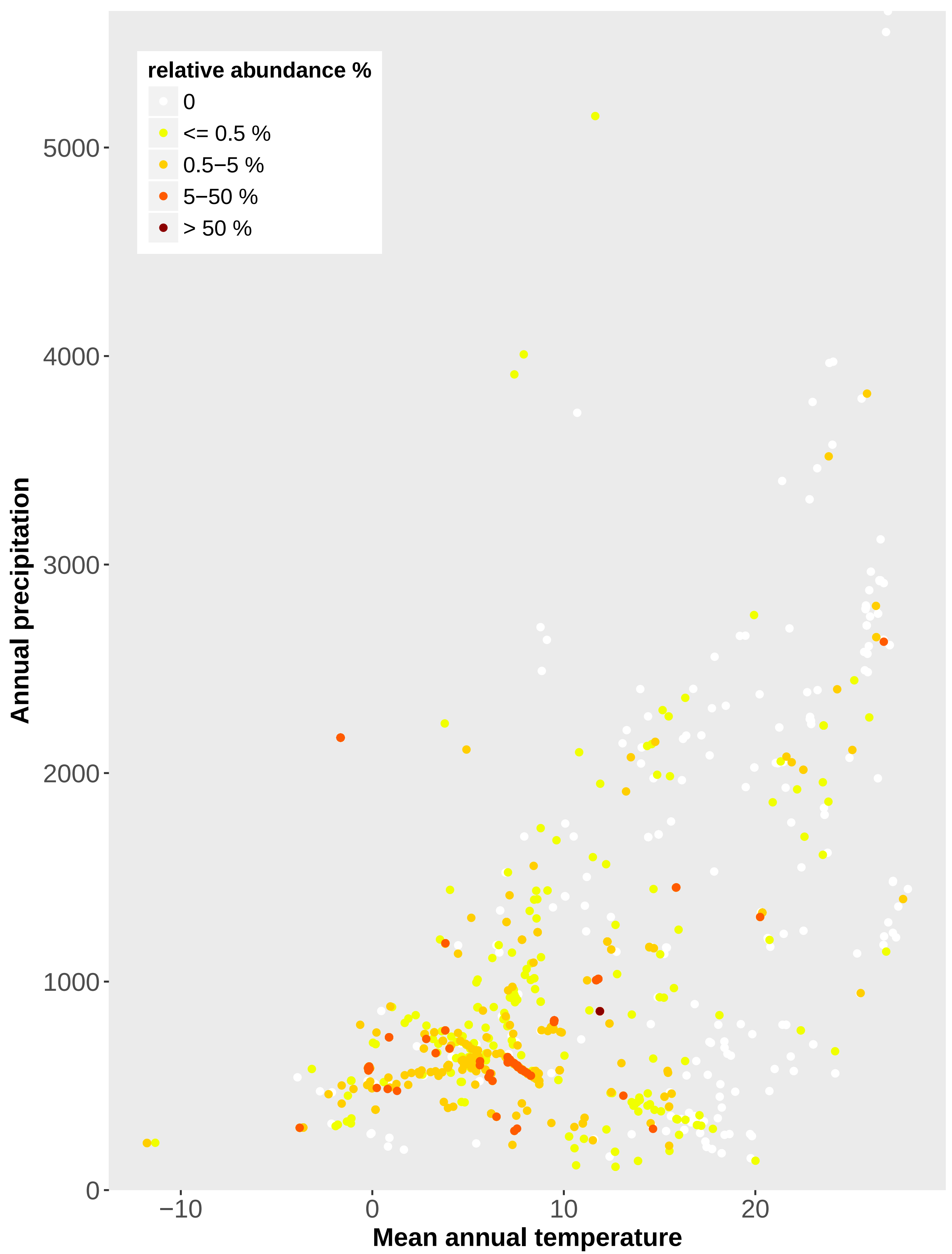

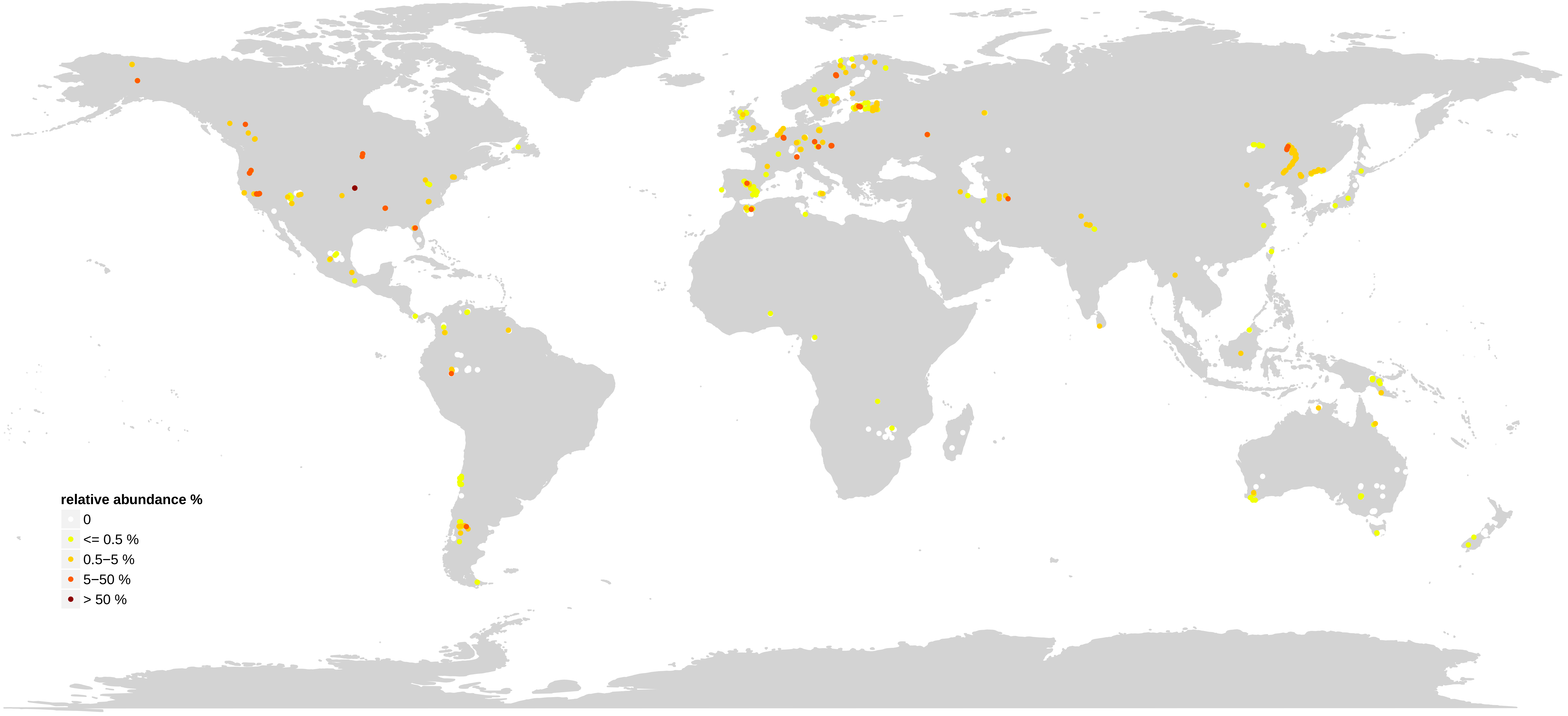

Herpotrichiellaceae

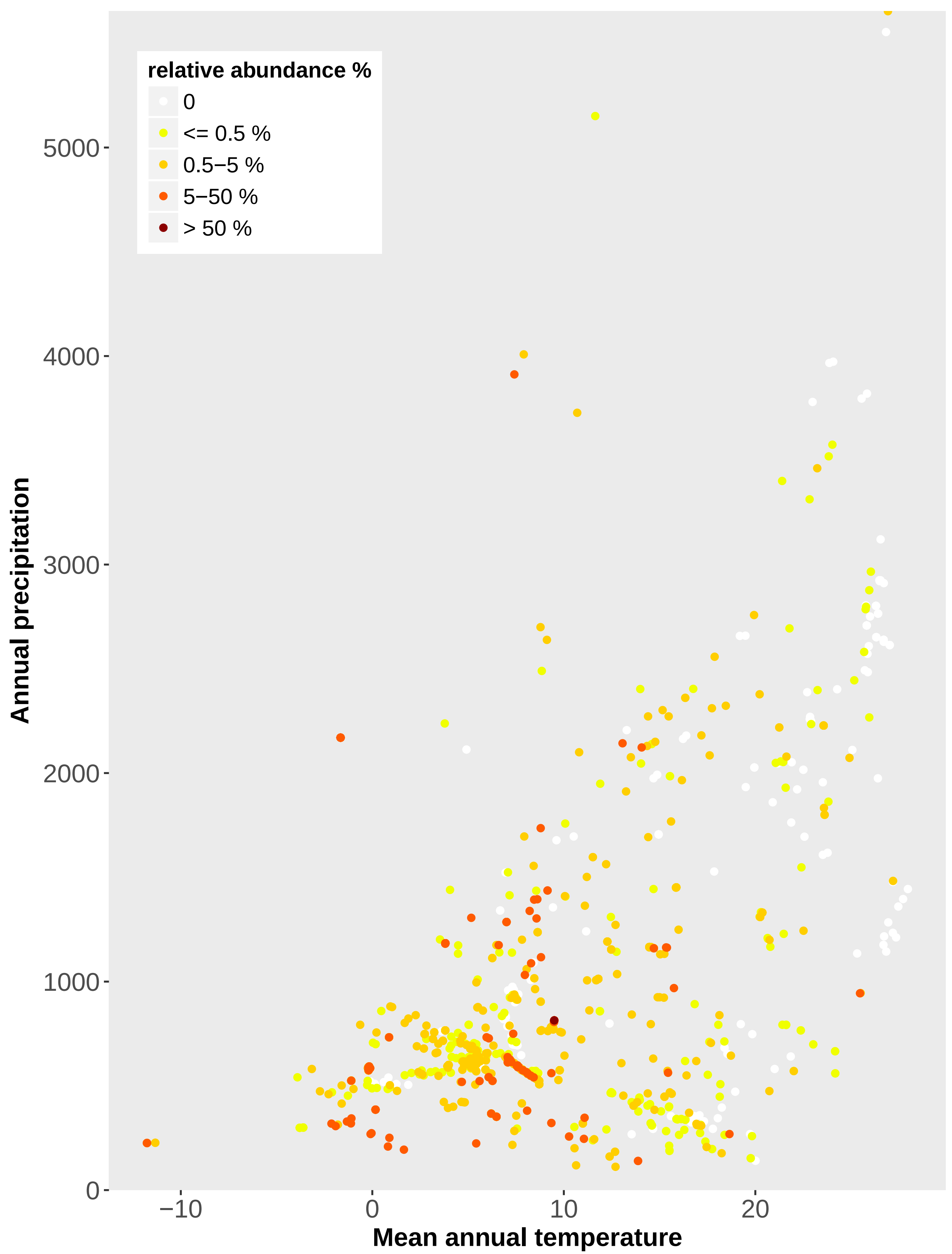

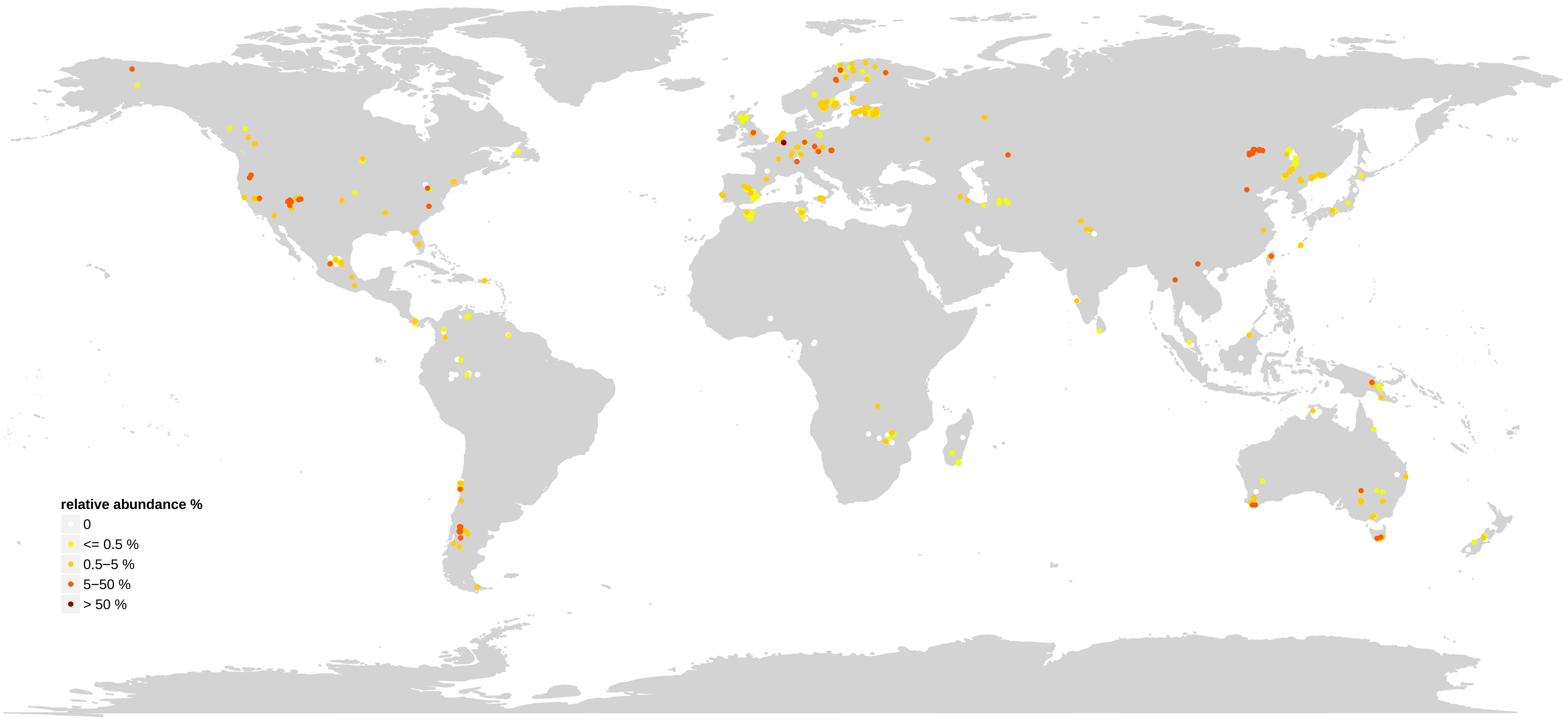

Hyaloscyphaceae

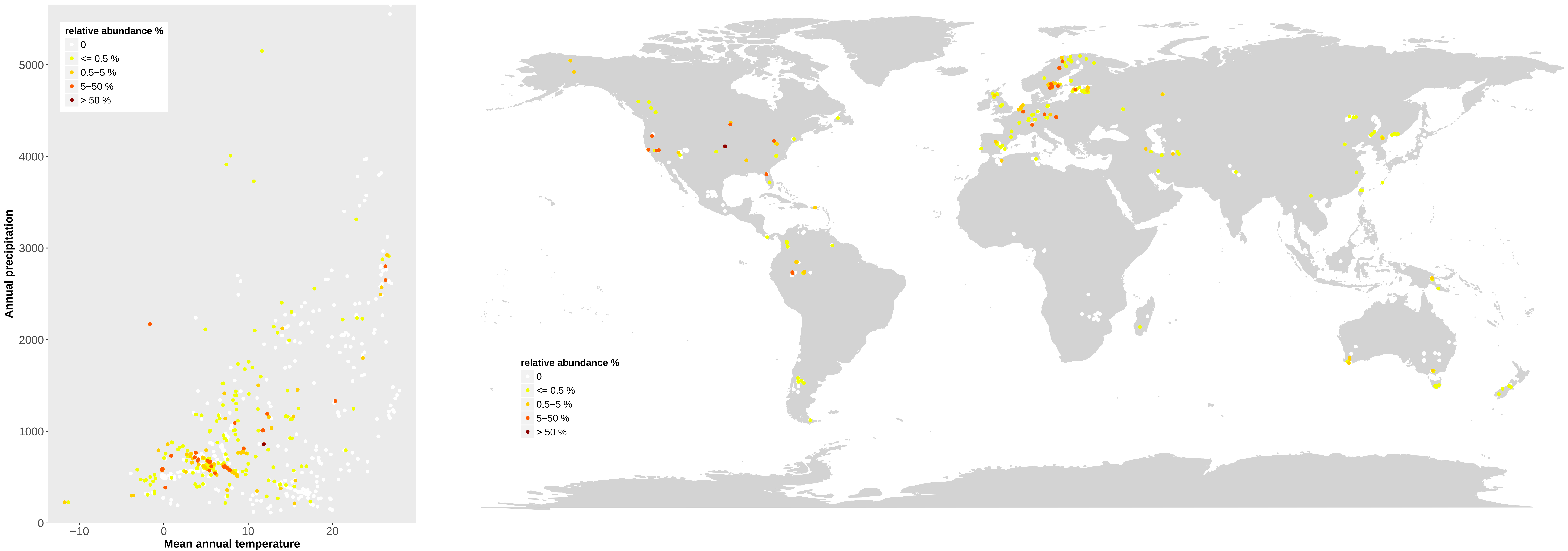

Hydnodontaceae

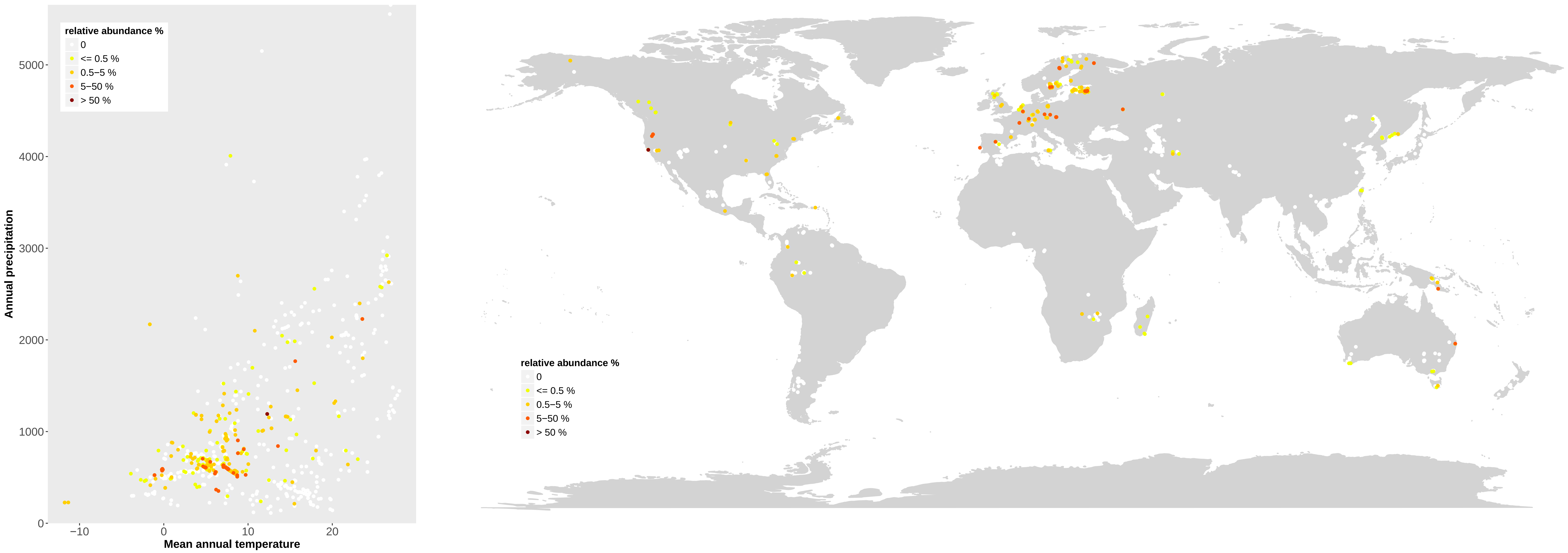

Hygrophoraceae

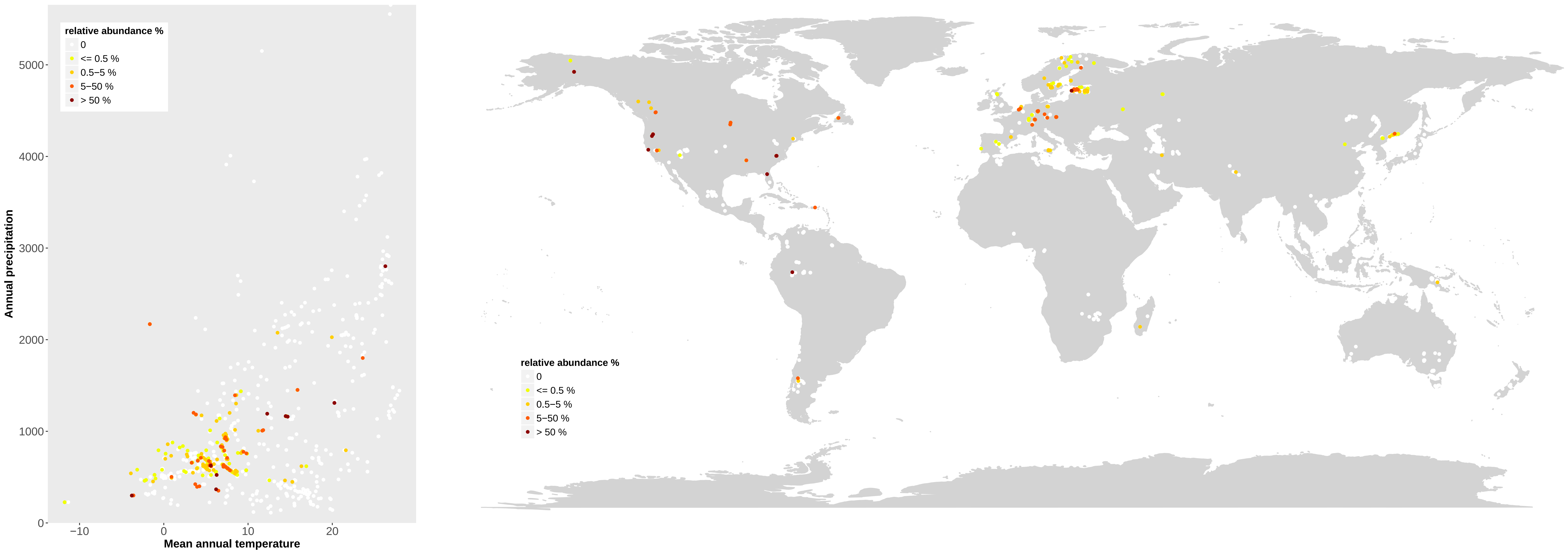

Hypocreaceae

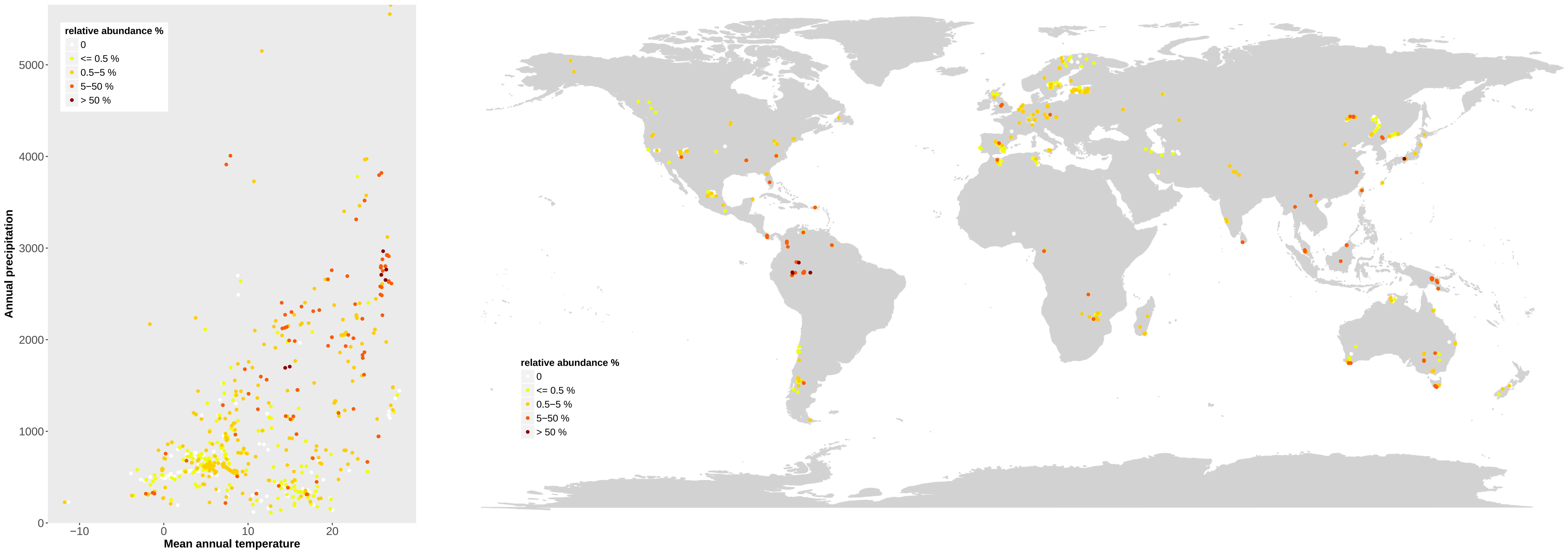

Hypocreales_fam_Incertae_sedis

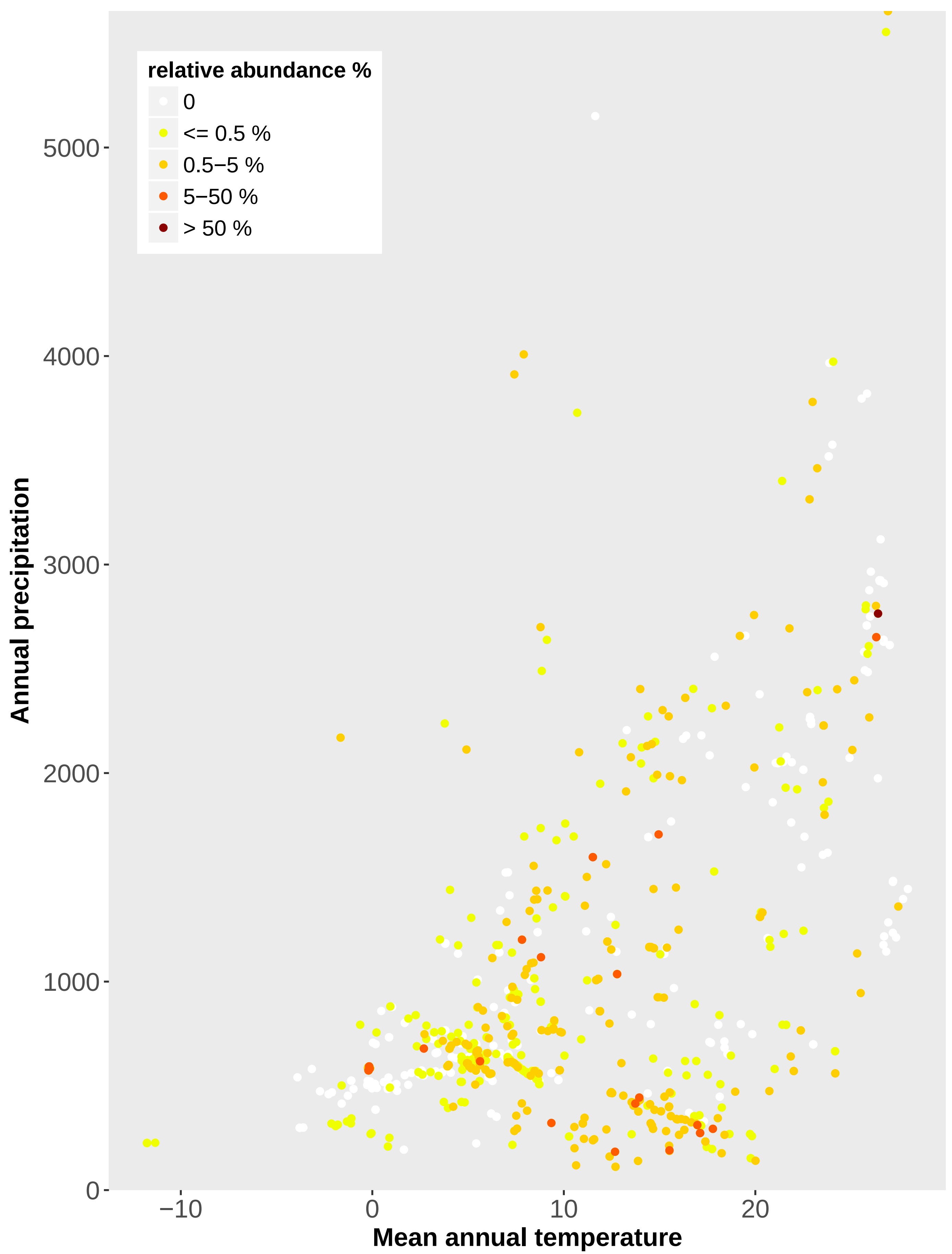

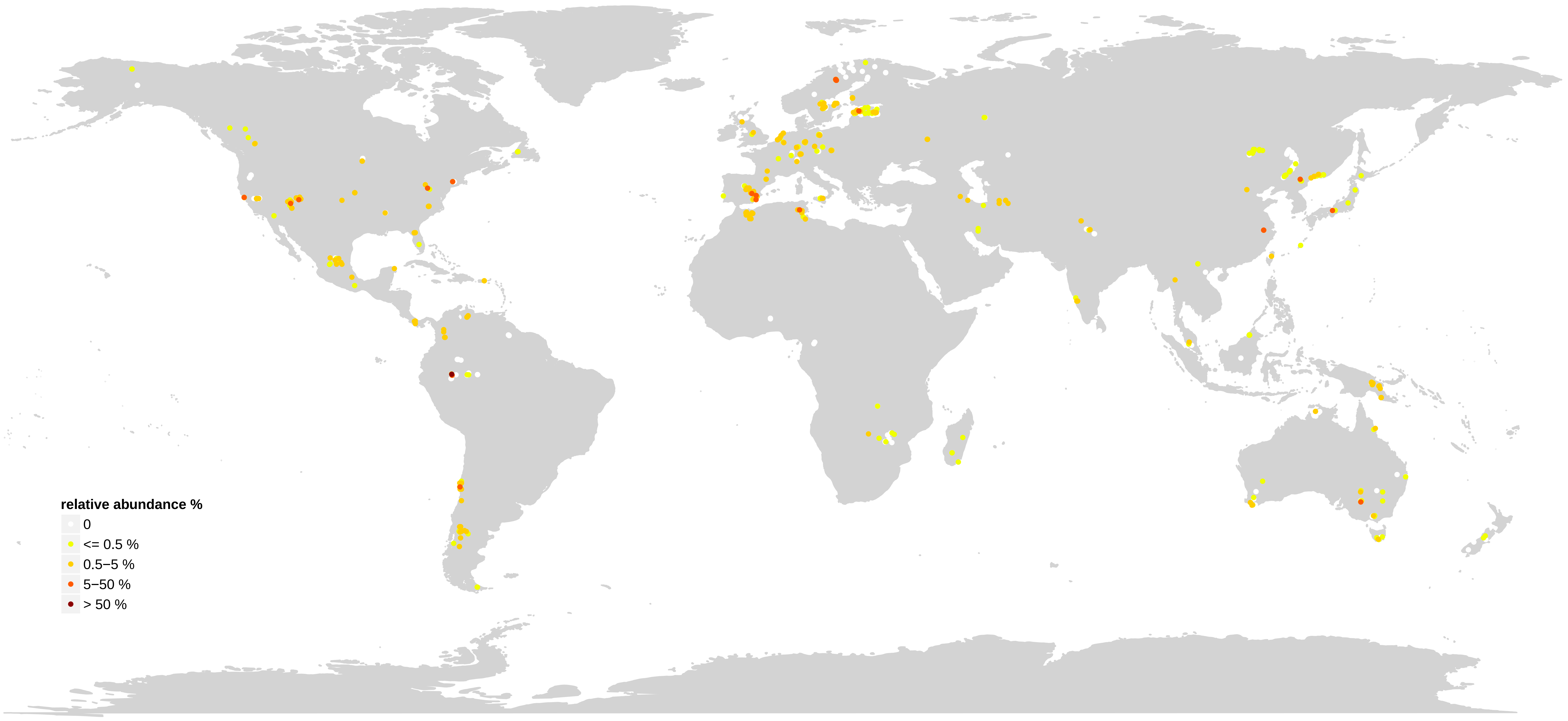

Inocybaceae

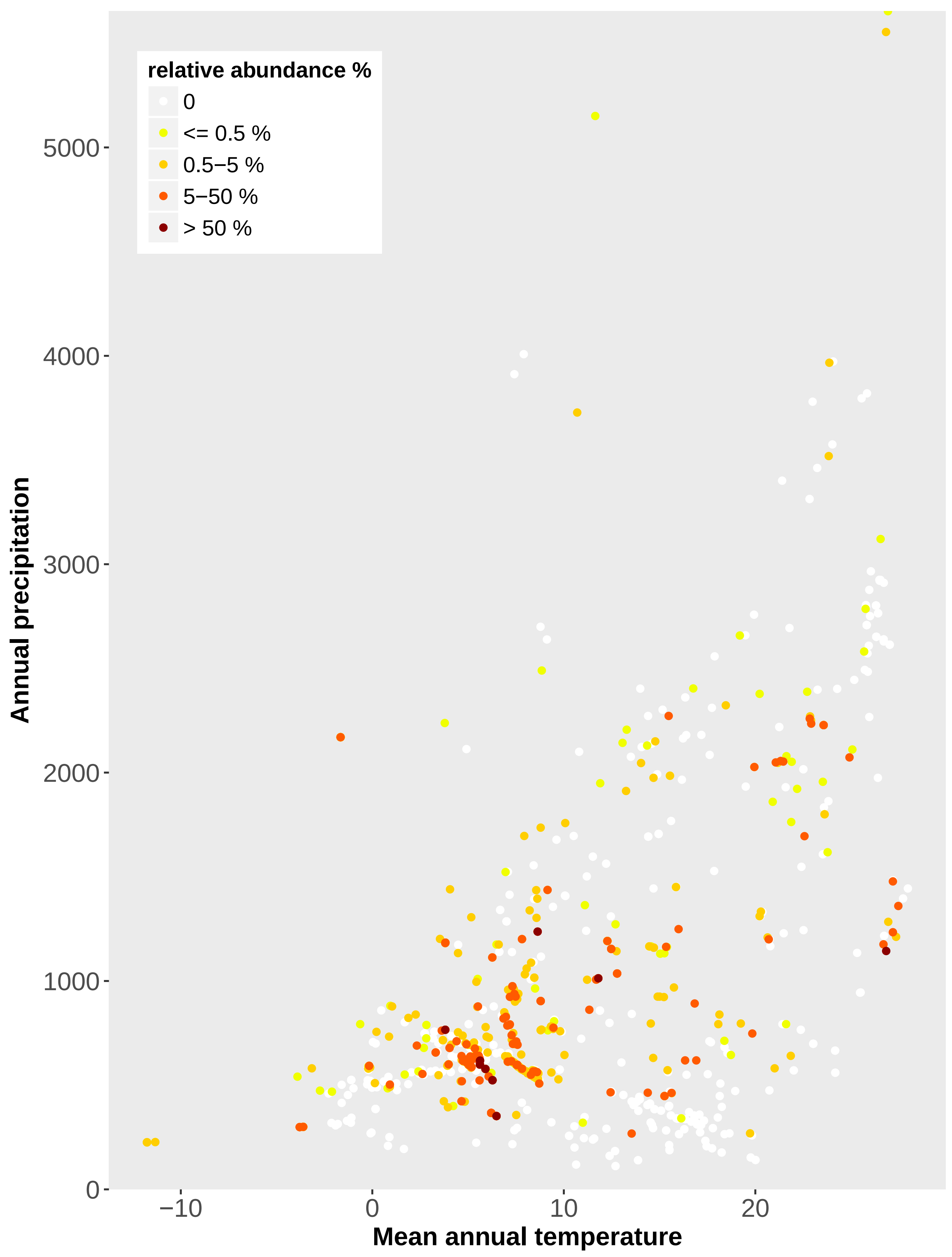

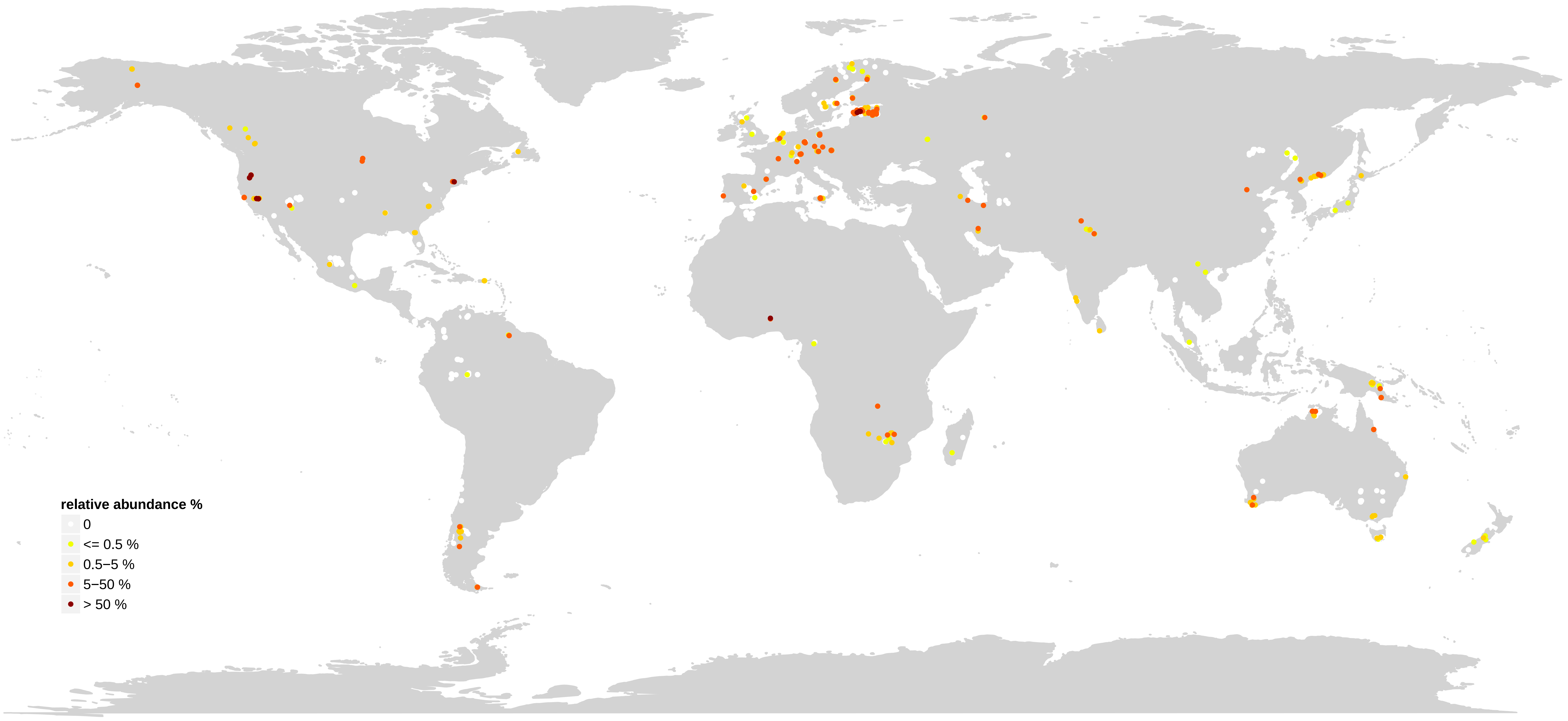

Lasiosphaeriaceae

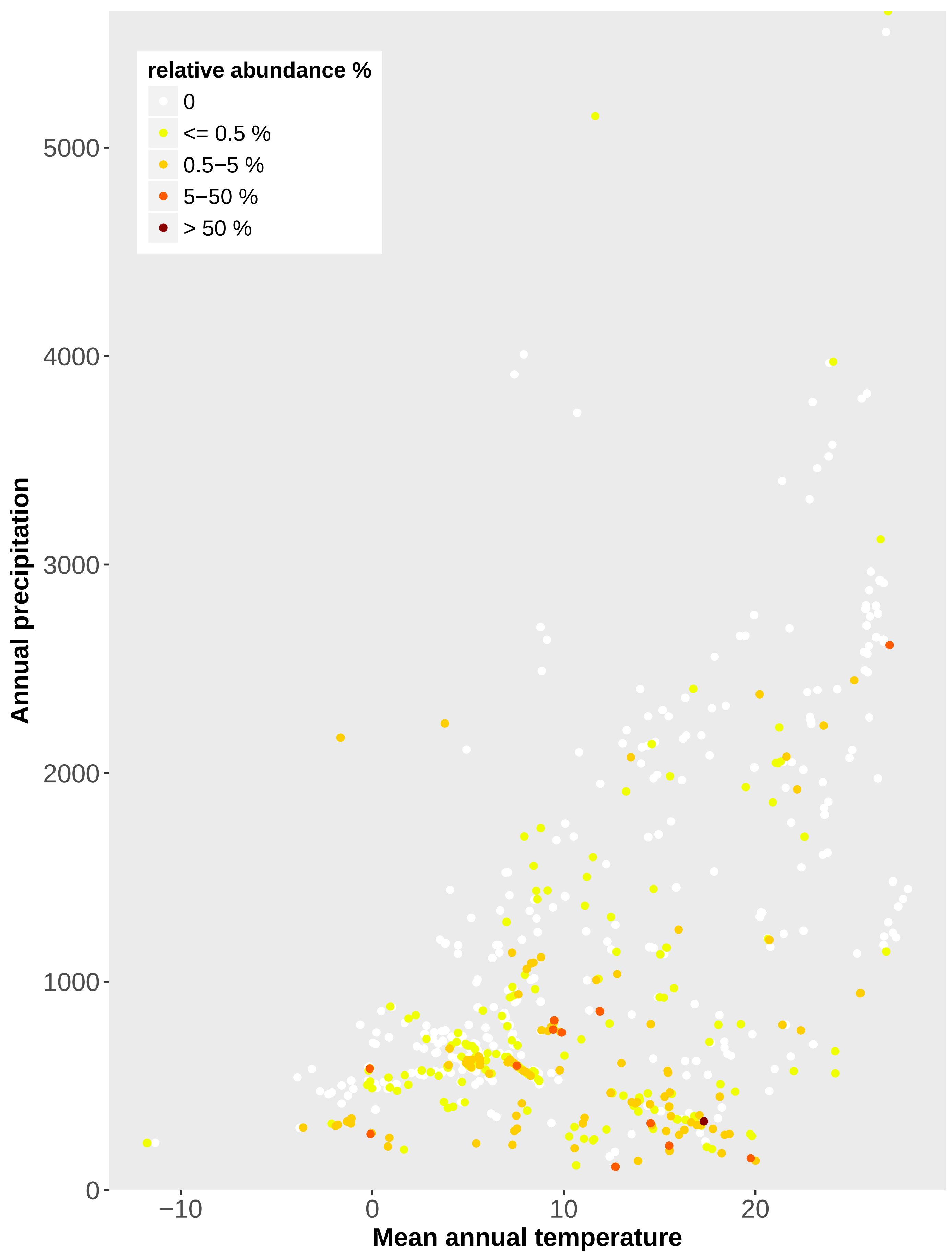

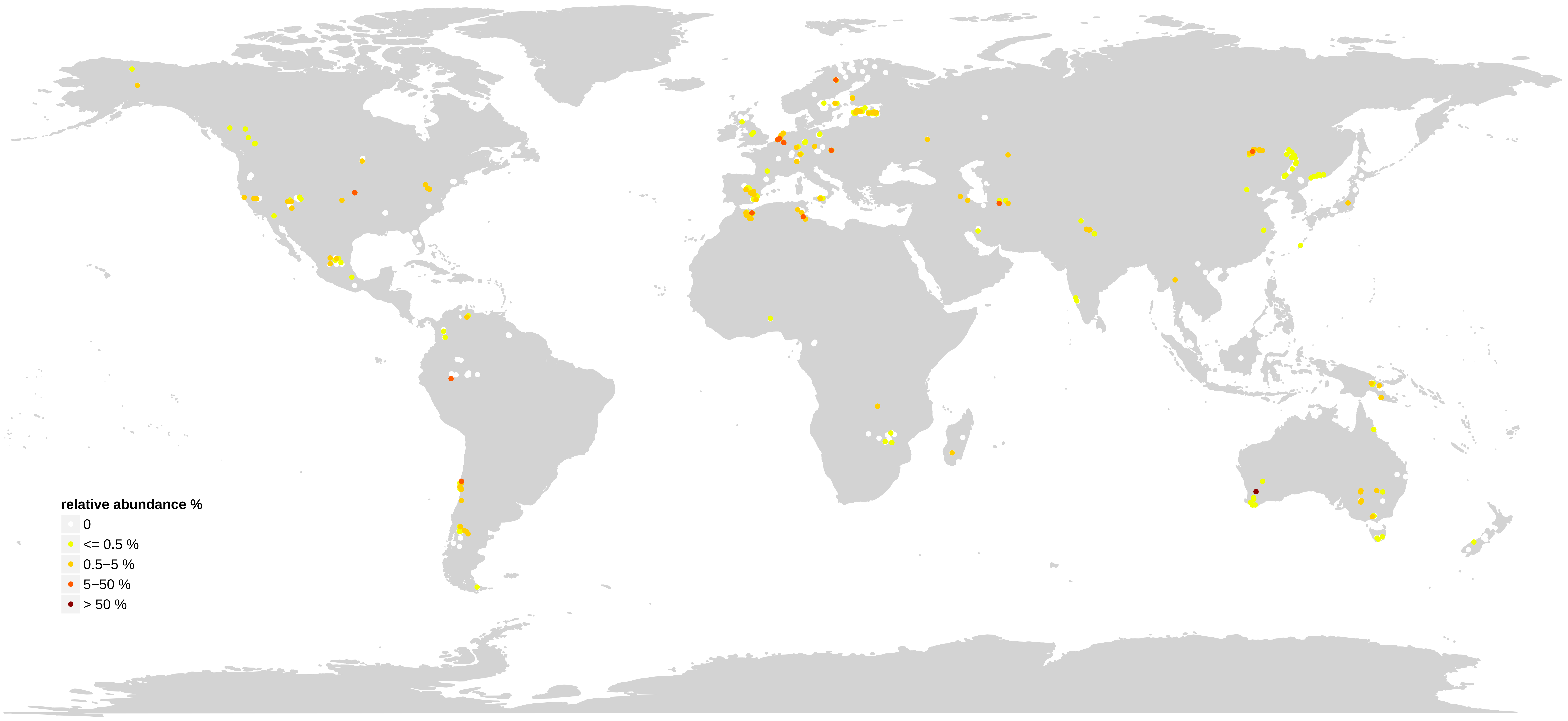

Mortierellaceae

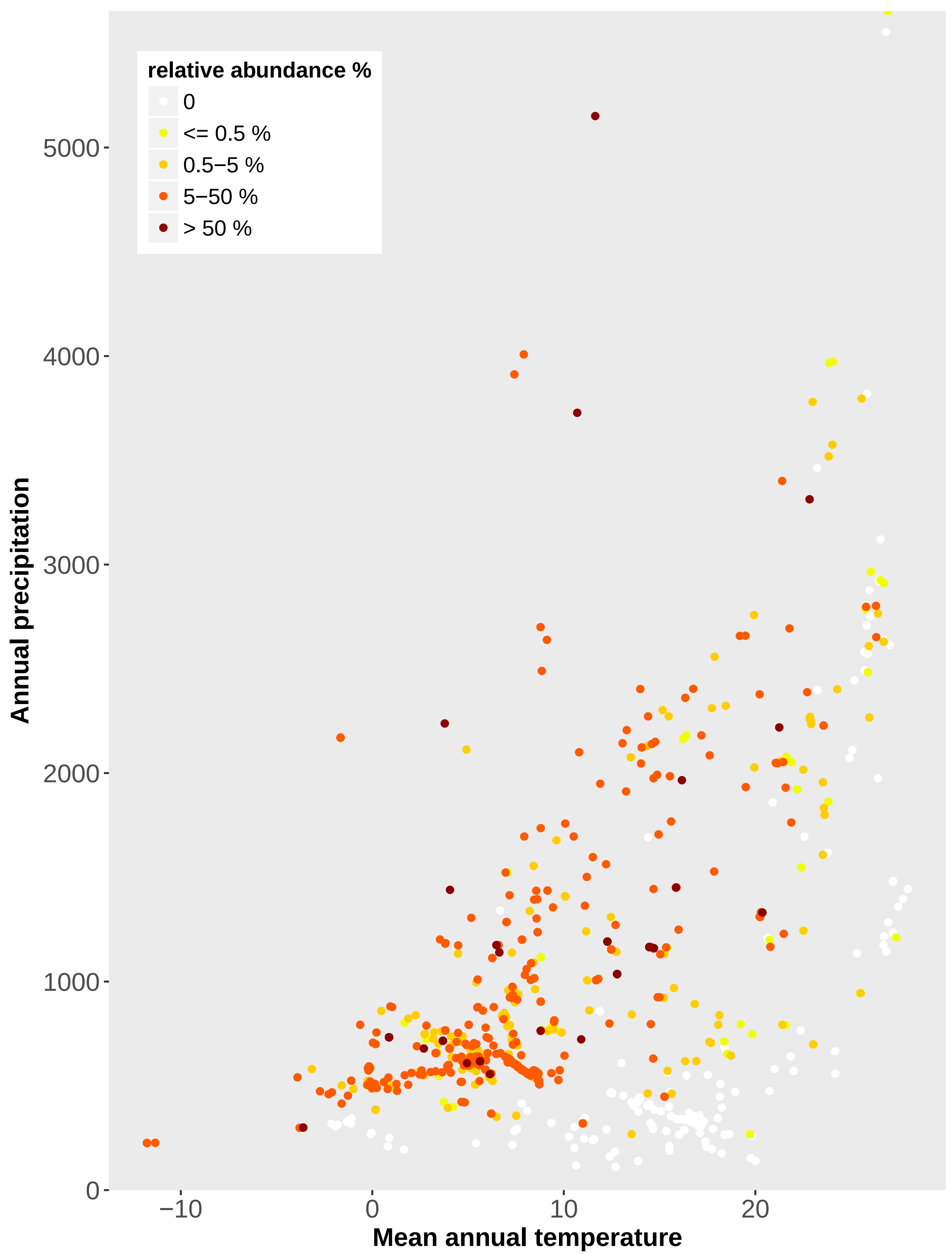

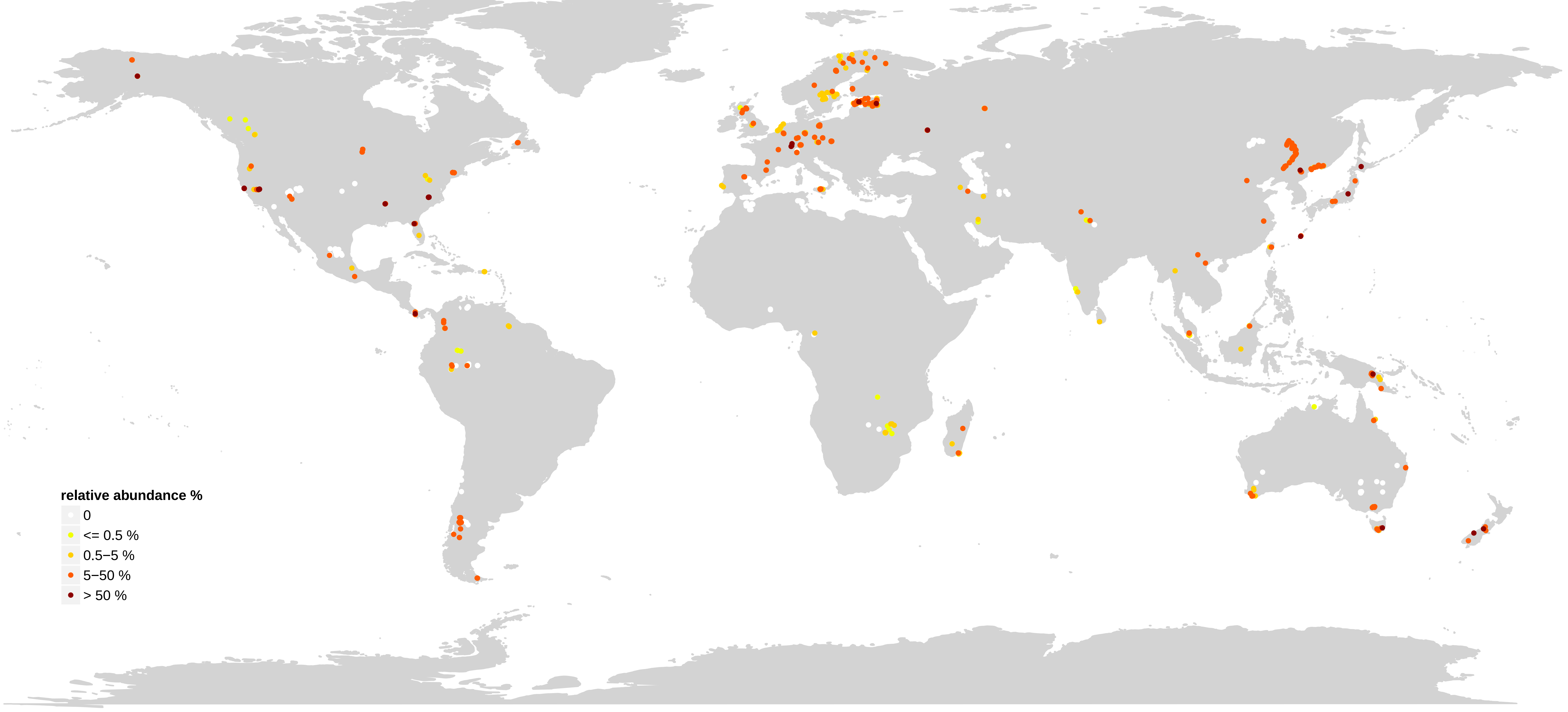

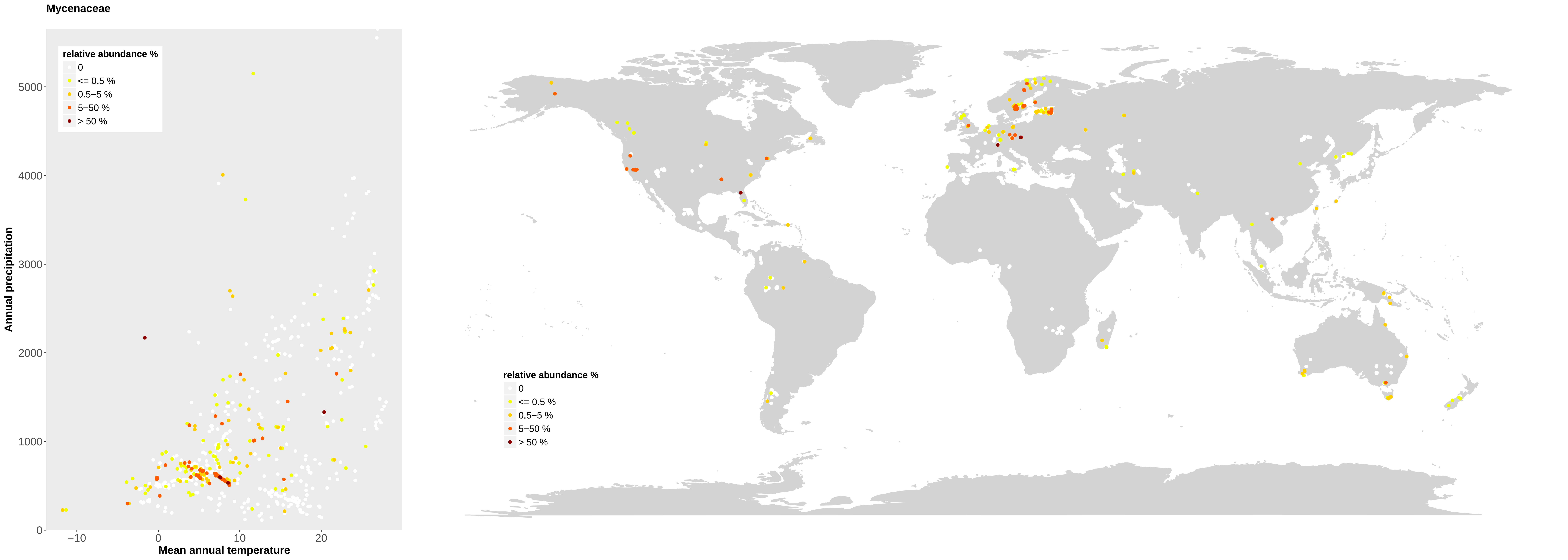
Mycenaceae
relative abundance %
0
<= 0.5 %
0.5–5 %
5–50 %
> 50 %
5000
4000
3000
2000
1000
0
−10
0
10
20
Annual precipitation
Mean annual temperature
relative abundance %
0
<= 0.5 %
0.5–5 %
5–50 %
> 50 %

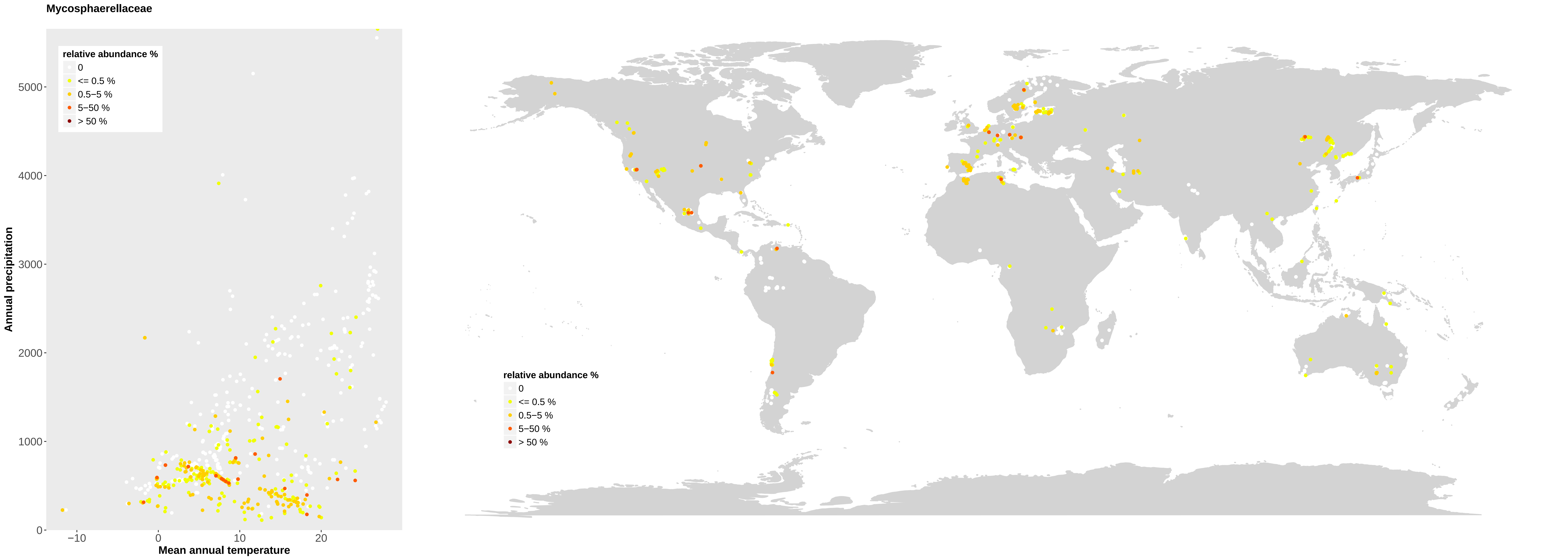
Mycosphaerellaceae
relative abundance %
0
<= 0.5 %
0.5–5 %
5–50 %
> 50 %
Annual precipitation
Mean annual temperature
0
1000
2000
3000
4000
5000
−10
0
10
20
relative abundance %
0
<= 0.5 %
0.5–5 %
5–50 %
> 50 %

Nectriaceae

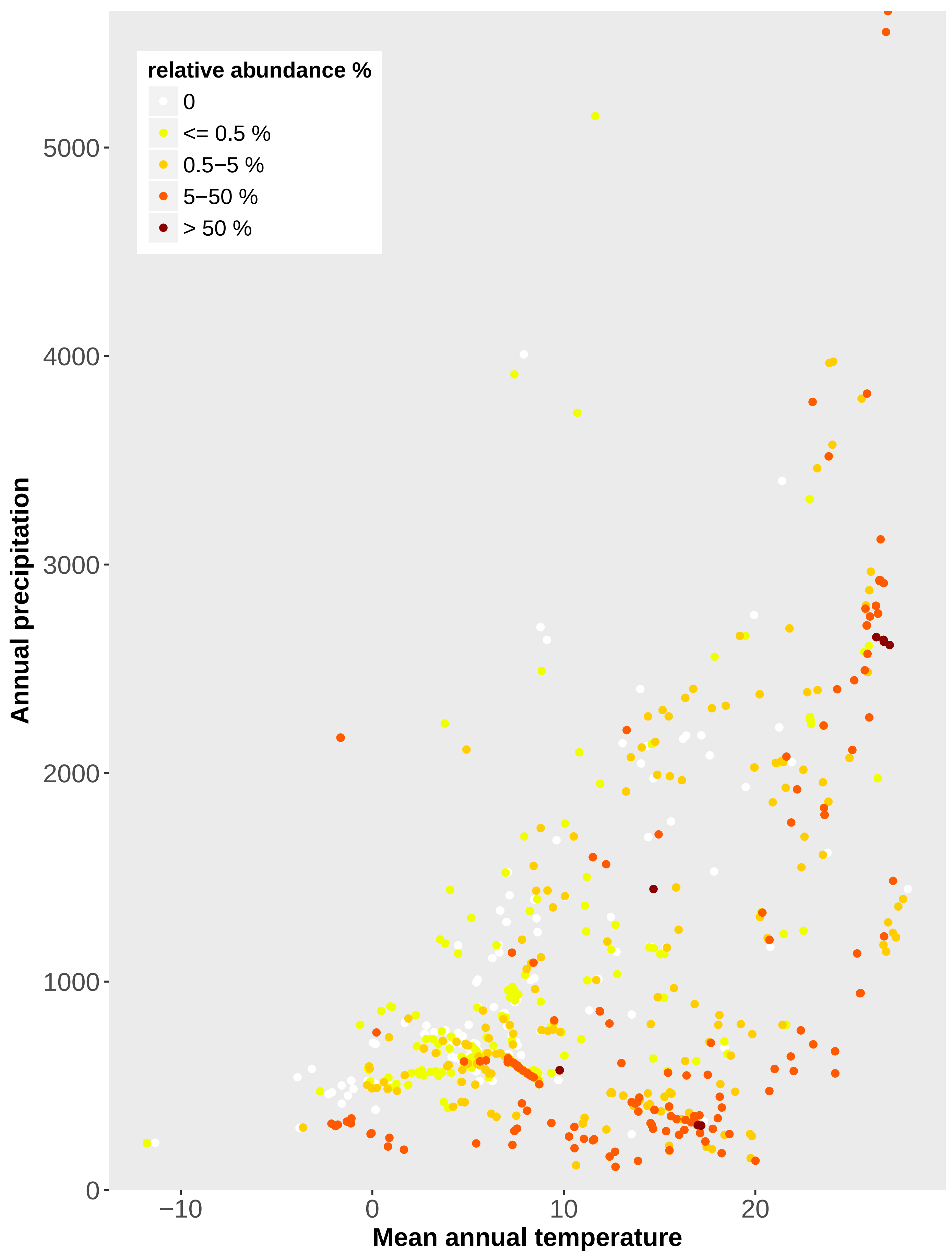

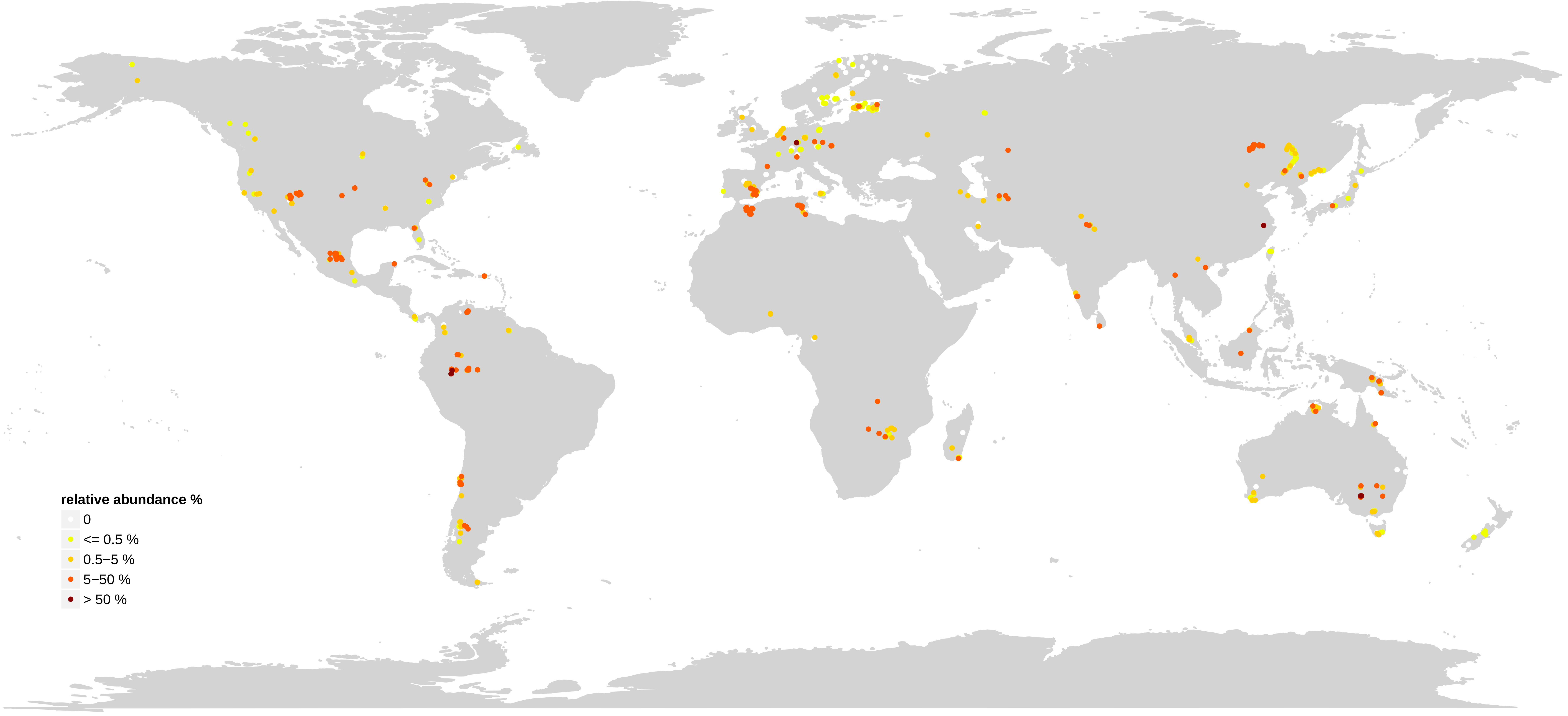

Pezizaceae

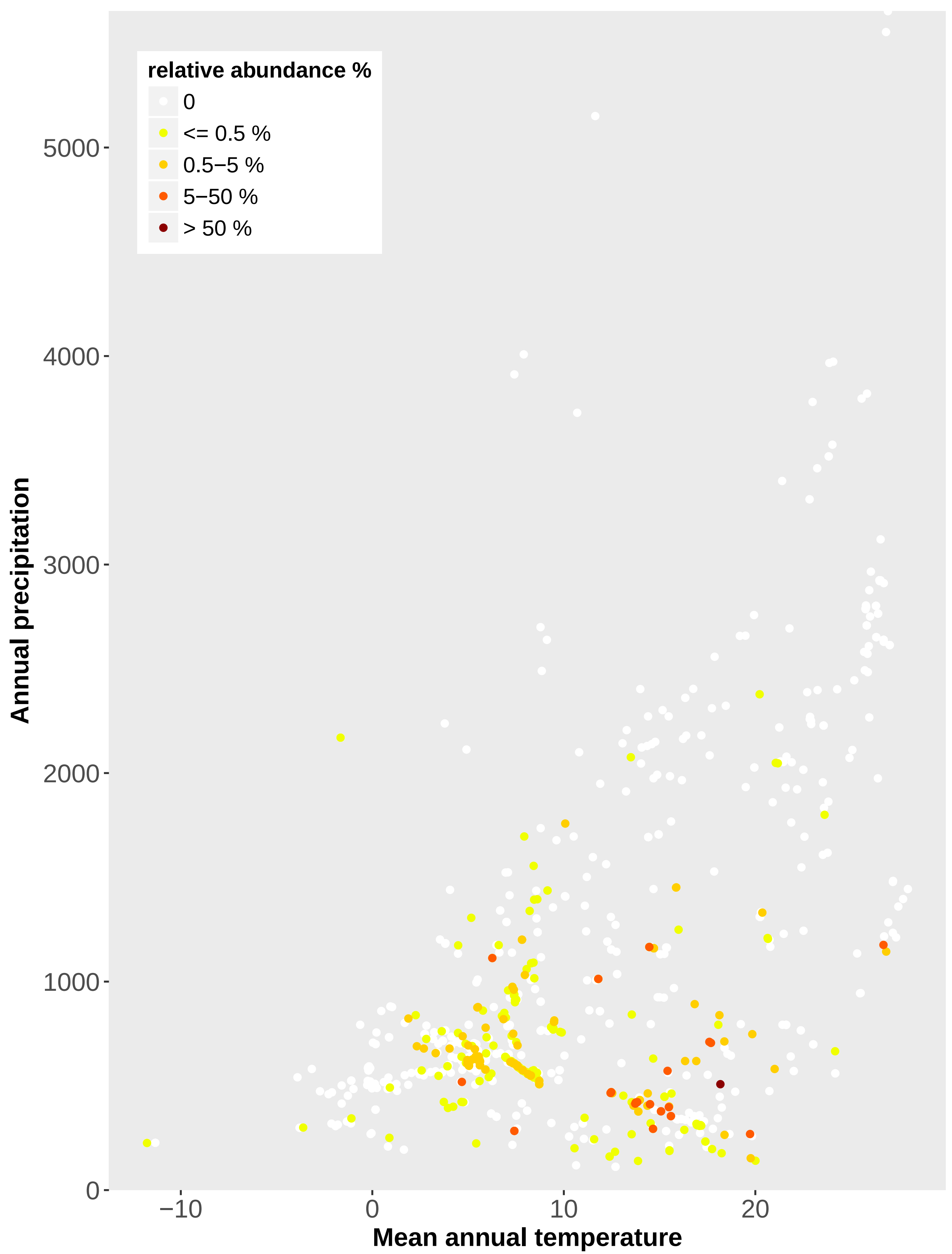
relative abundance %
0
<= 0.5 %
0.5–5 %
5–50 %
> 50 %
Annual precipitation
5000
4000
3000
2000
1000
0
−10
0
10
20
Mean annual temperature

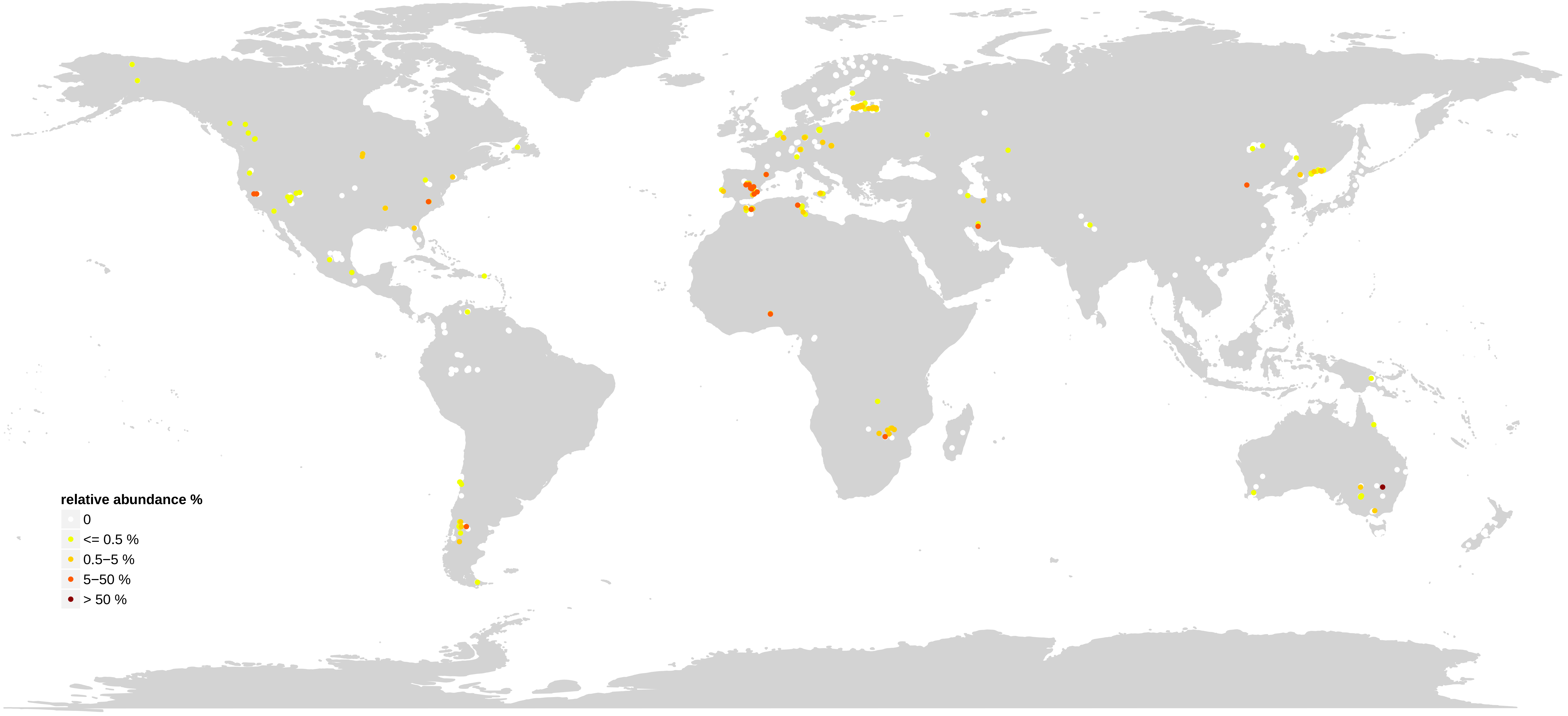
relative abundance %
0
<= 0.5 %
0.5–5 %
5–50 %
> 50 %

Pezizomycotina_fam_Incertae_sedis

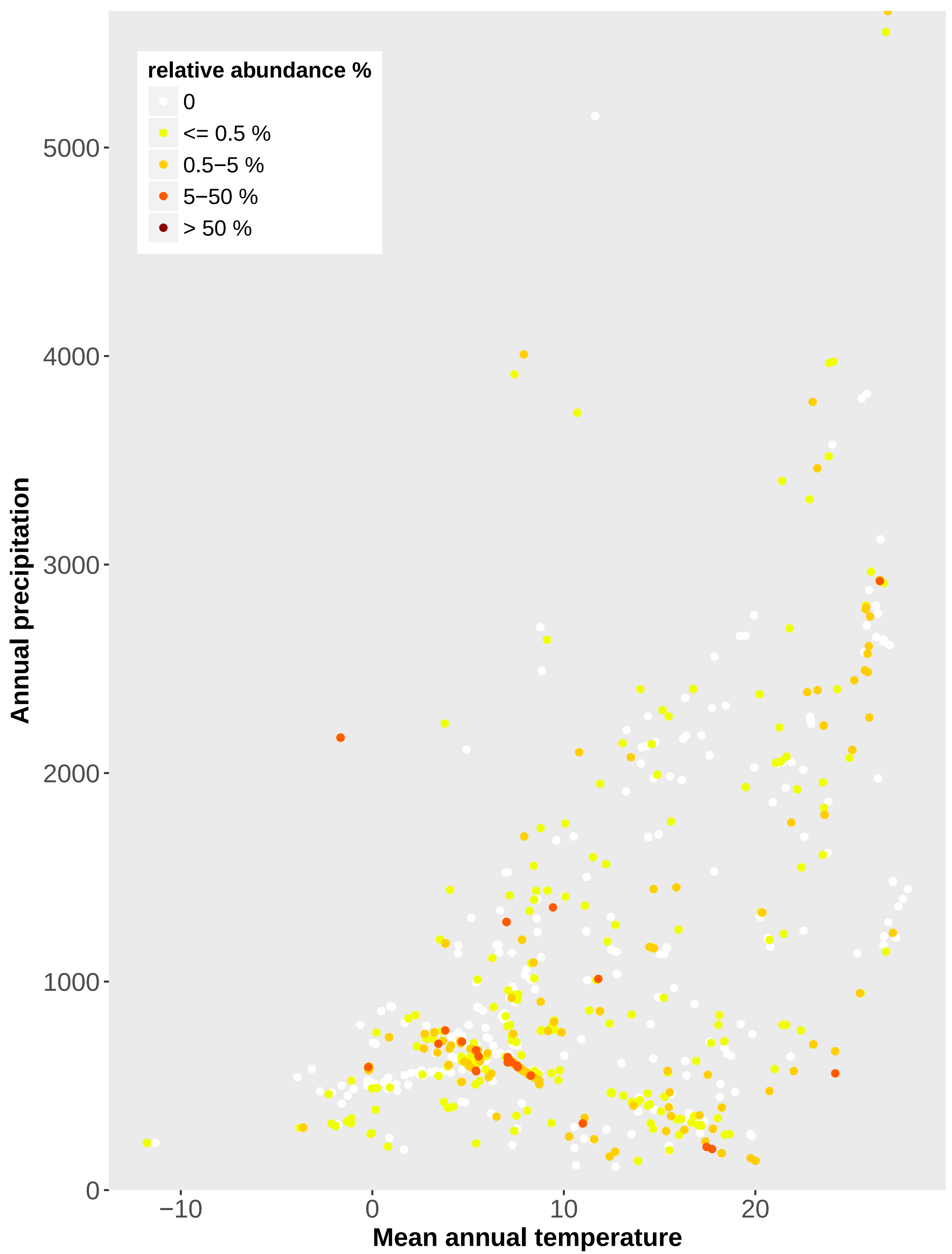

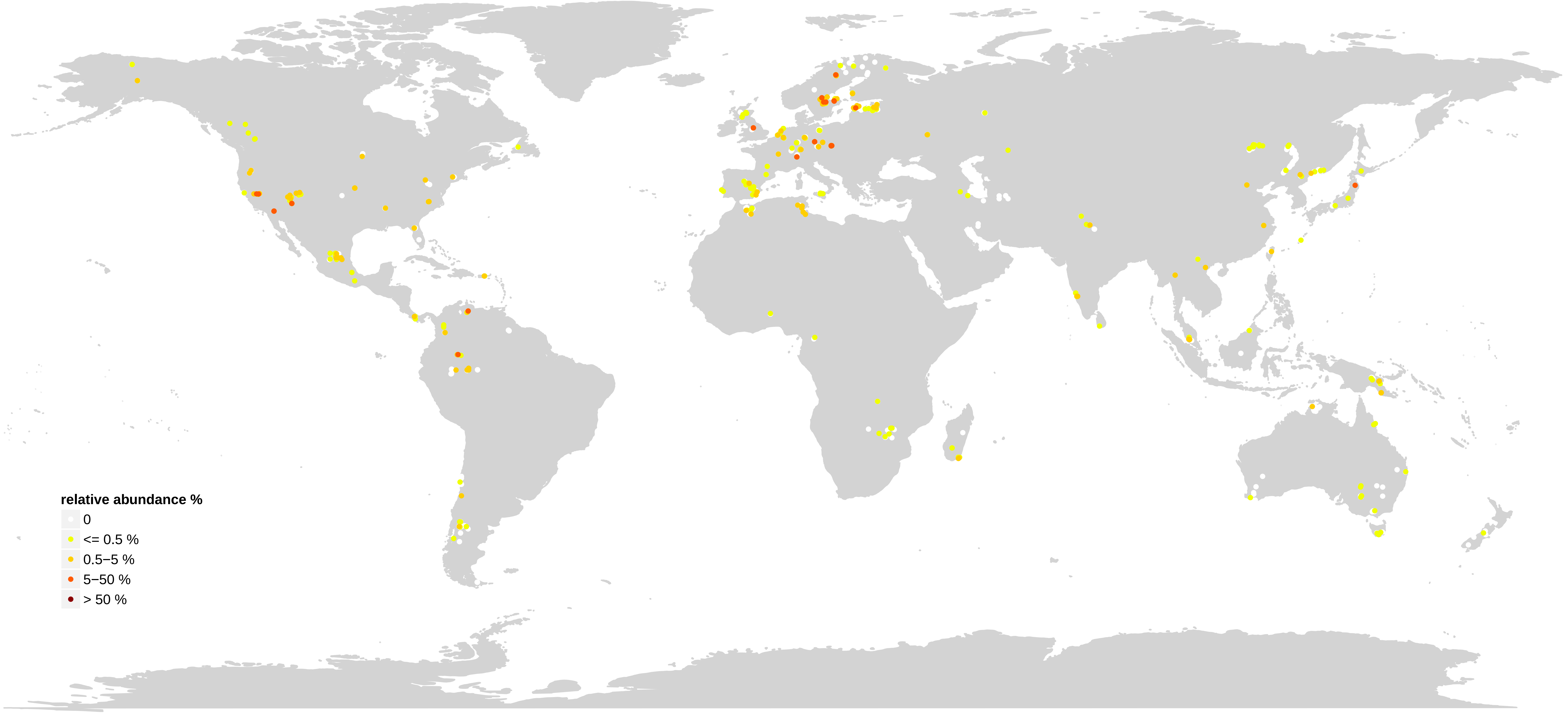

Phaeosphaeriaceae

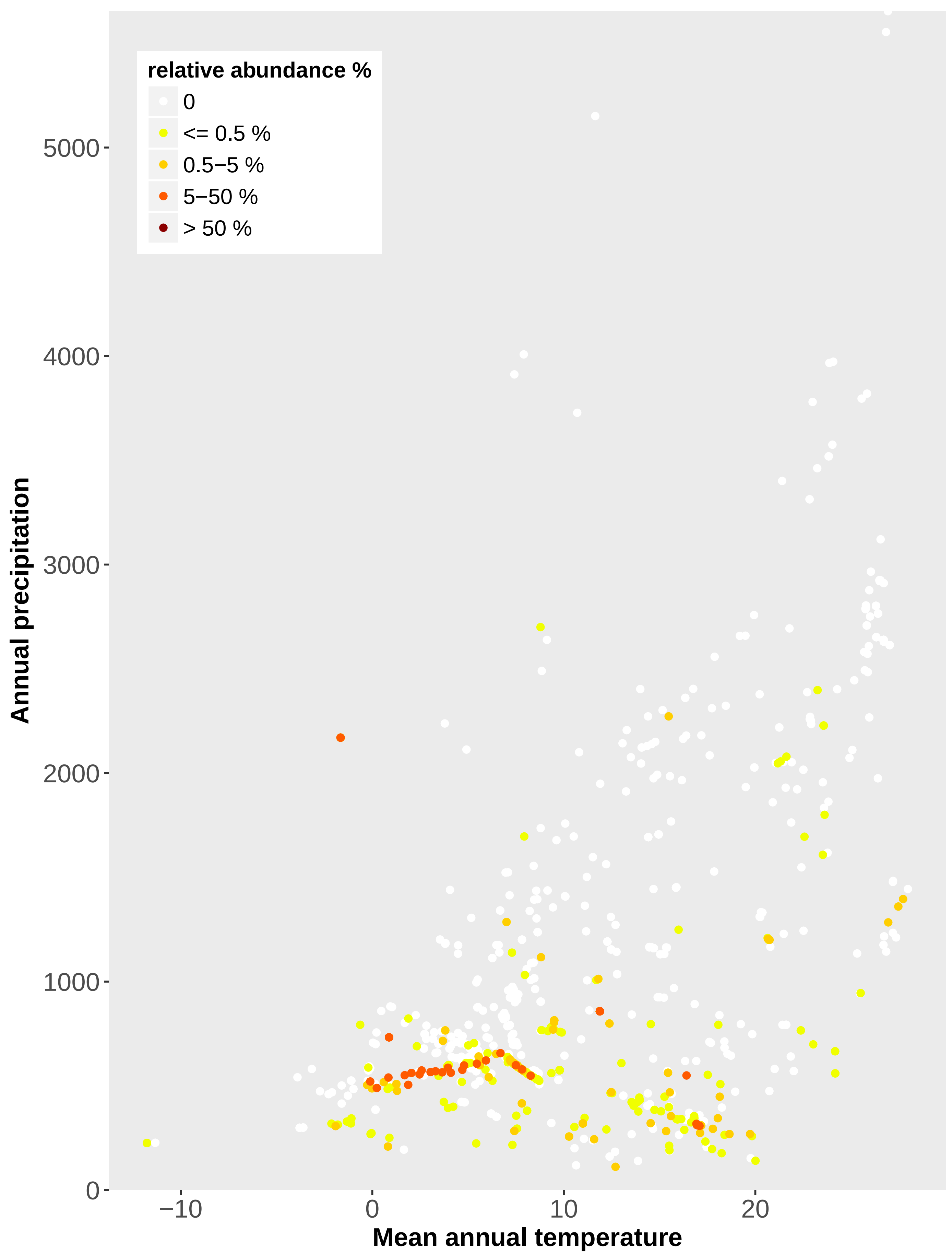

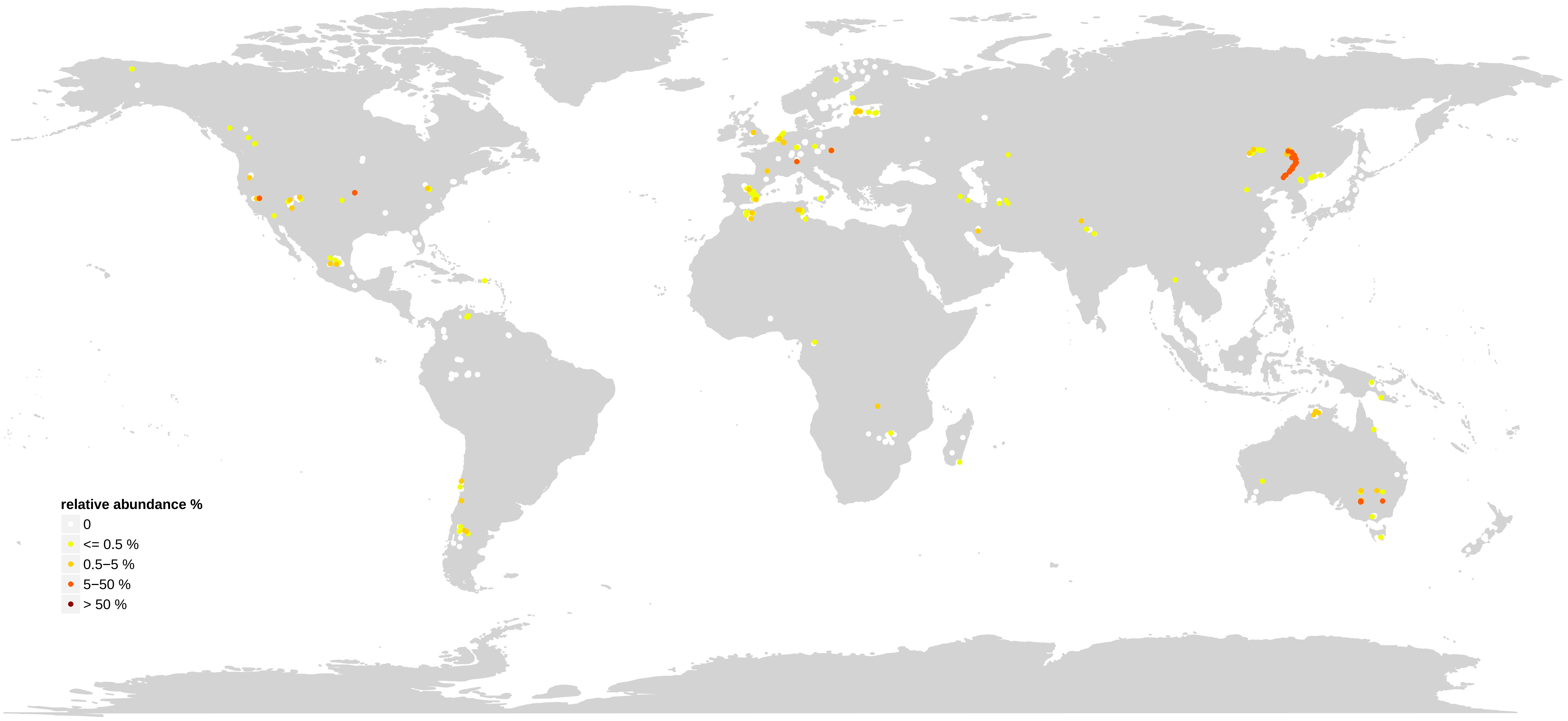

Pleosporaceae

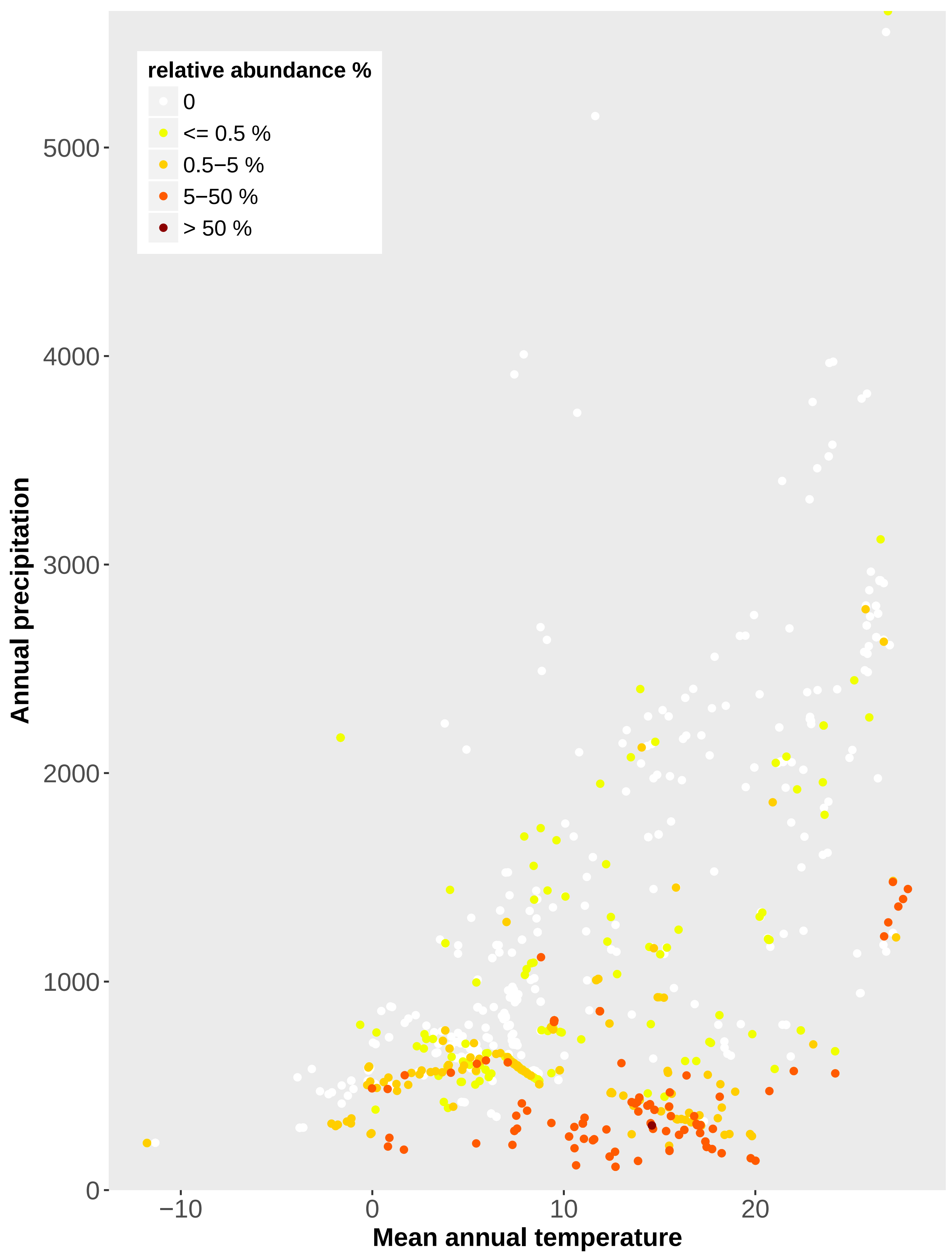

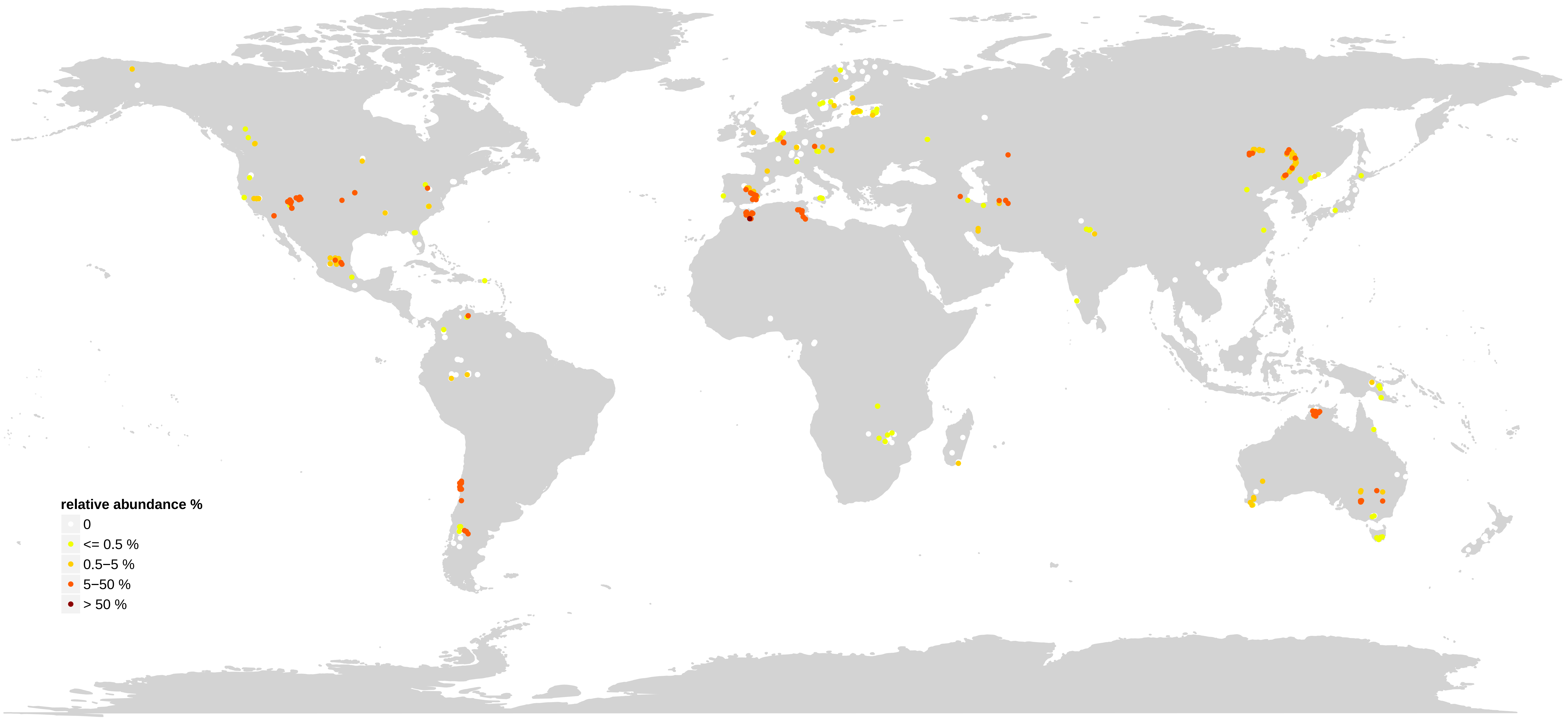

Pleosporales_fam_Incertae_sedis

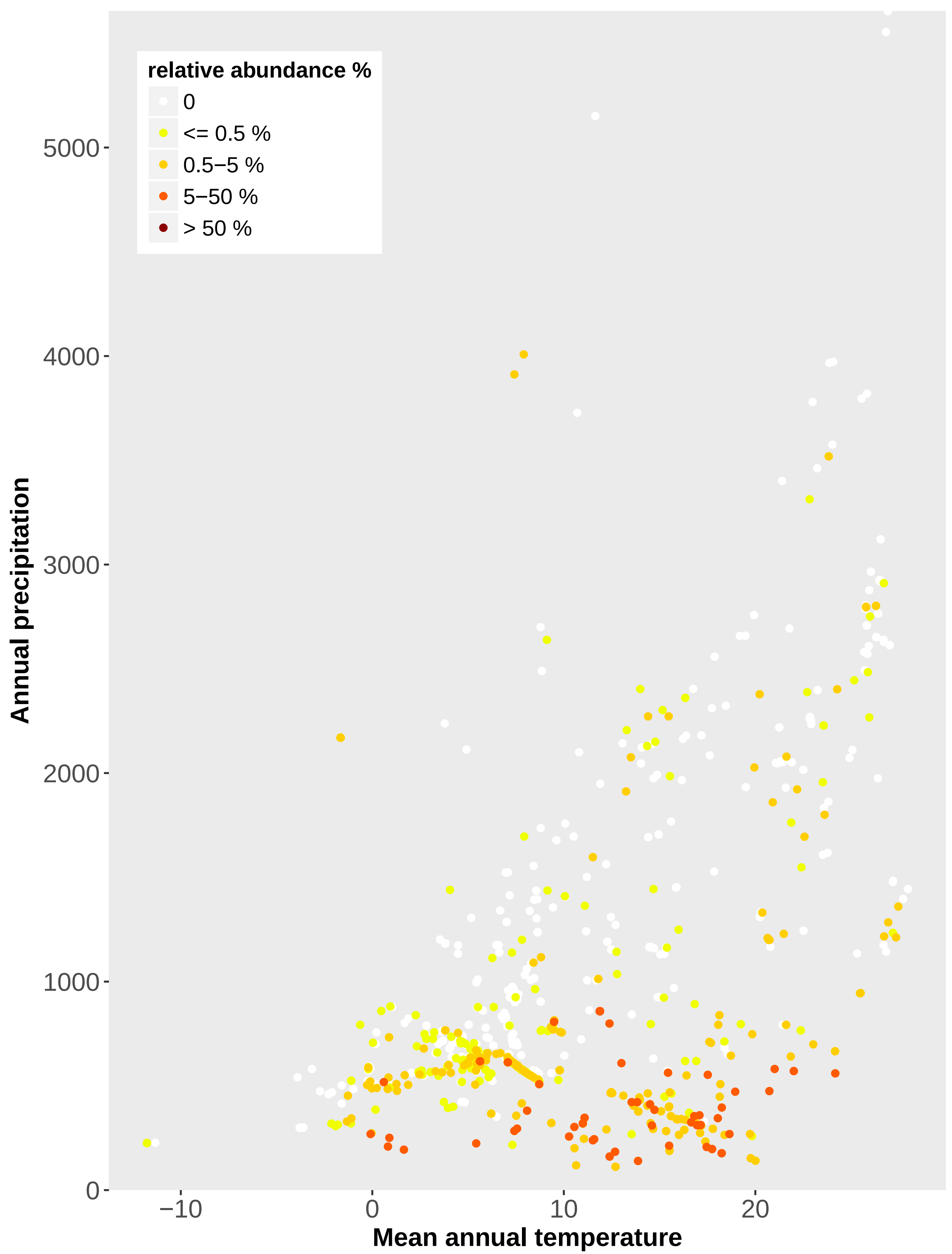

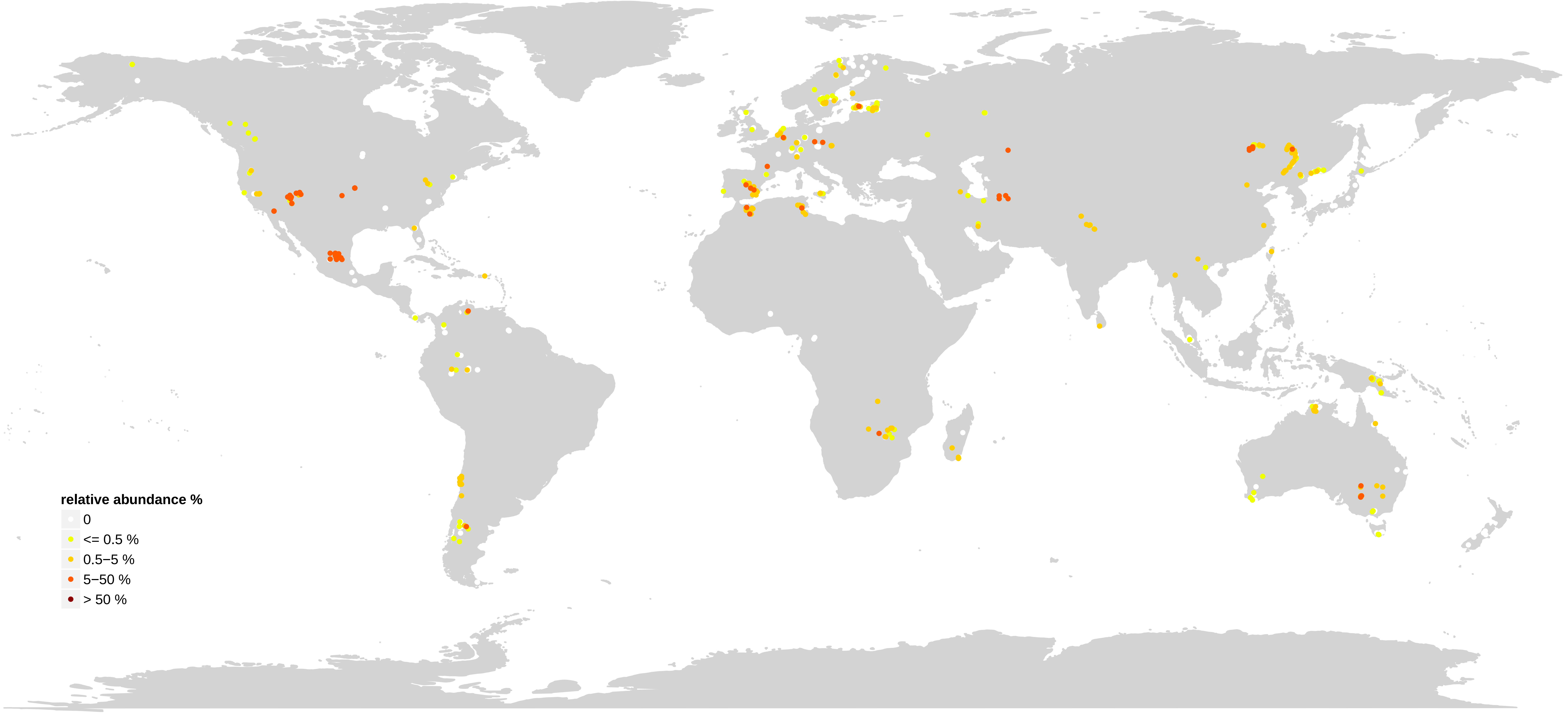

Psathyrellaceae

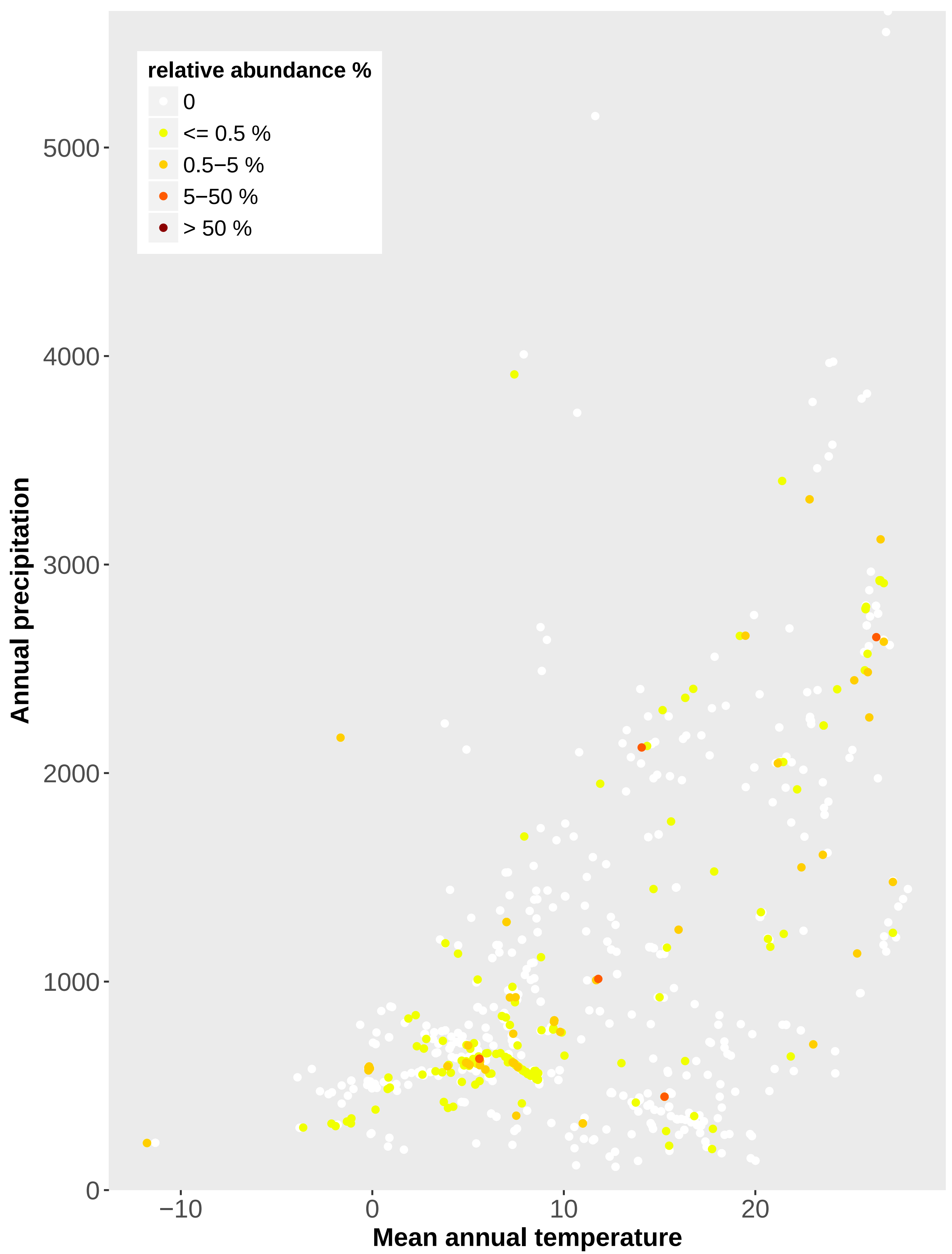

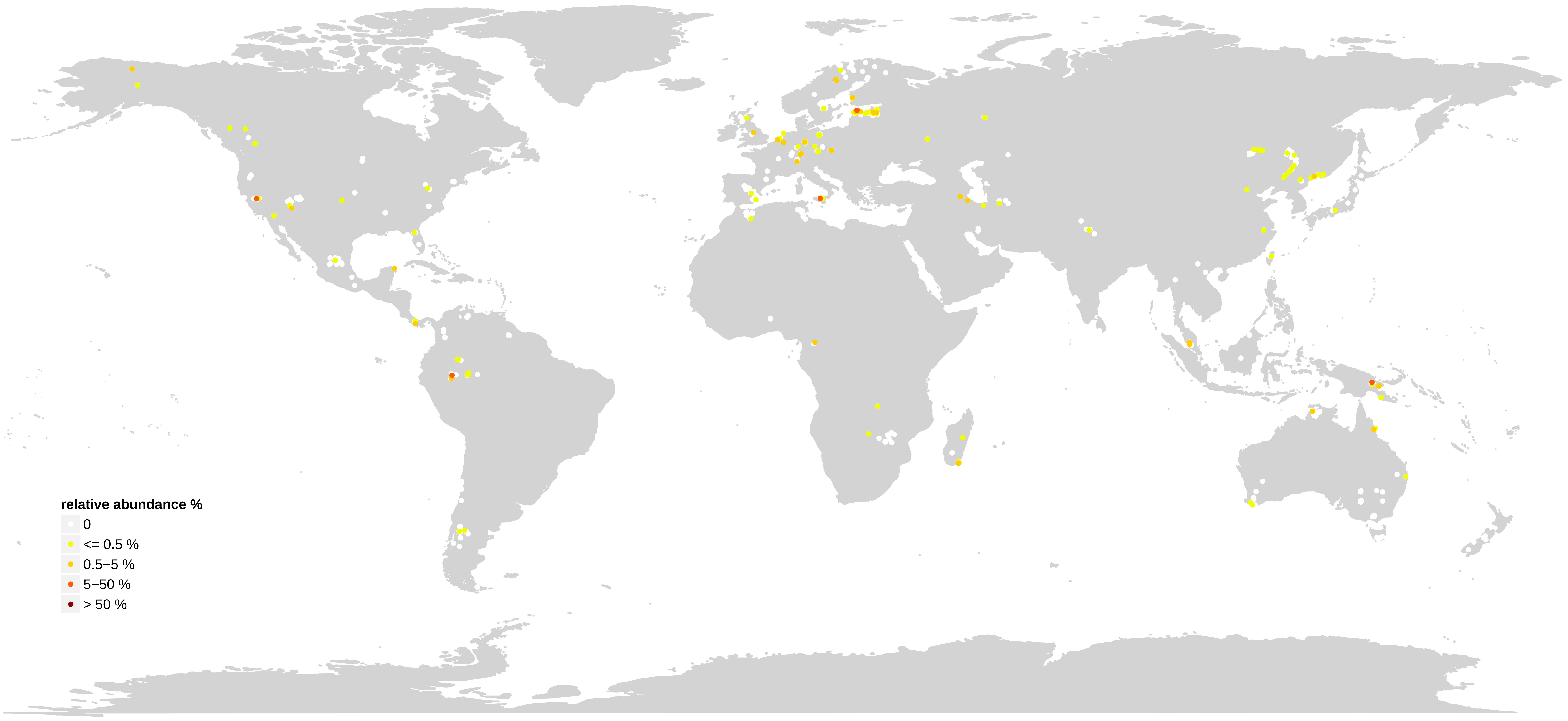

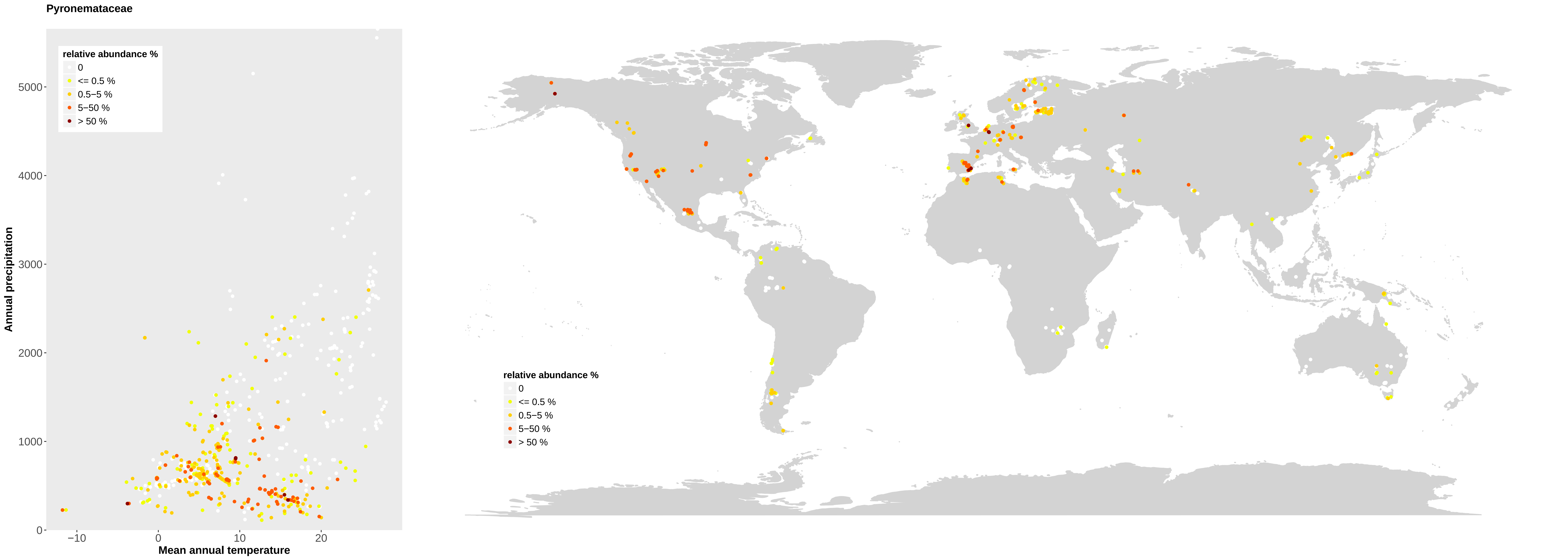

Pyronemataceae
relative abundance %
0
<= 0.5 %
0.5–5 %
5–50 %
> 50 %
5000
4000
3000
2000
1000
0
Annual precipitation
−10
0
10
20
Mean annual temperature
relative abundance %
0
<= 0.5 %
0.5–5 %
5–50 %
> 50 %

Russulaceae

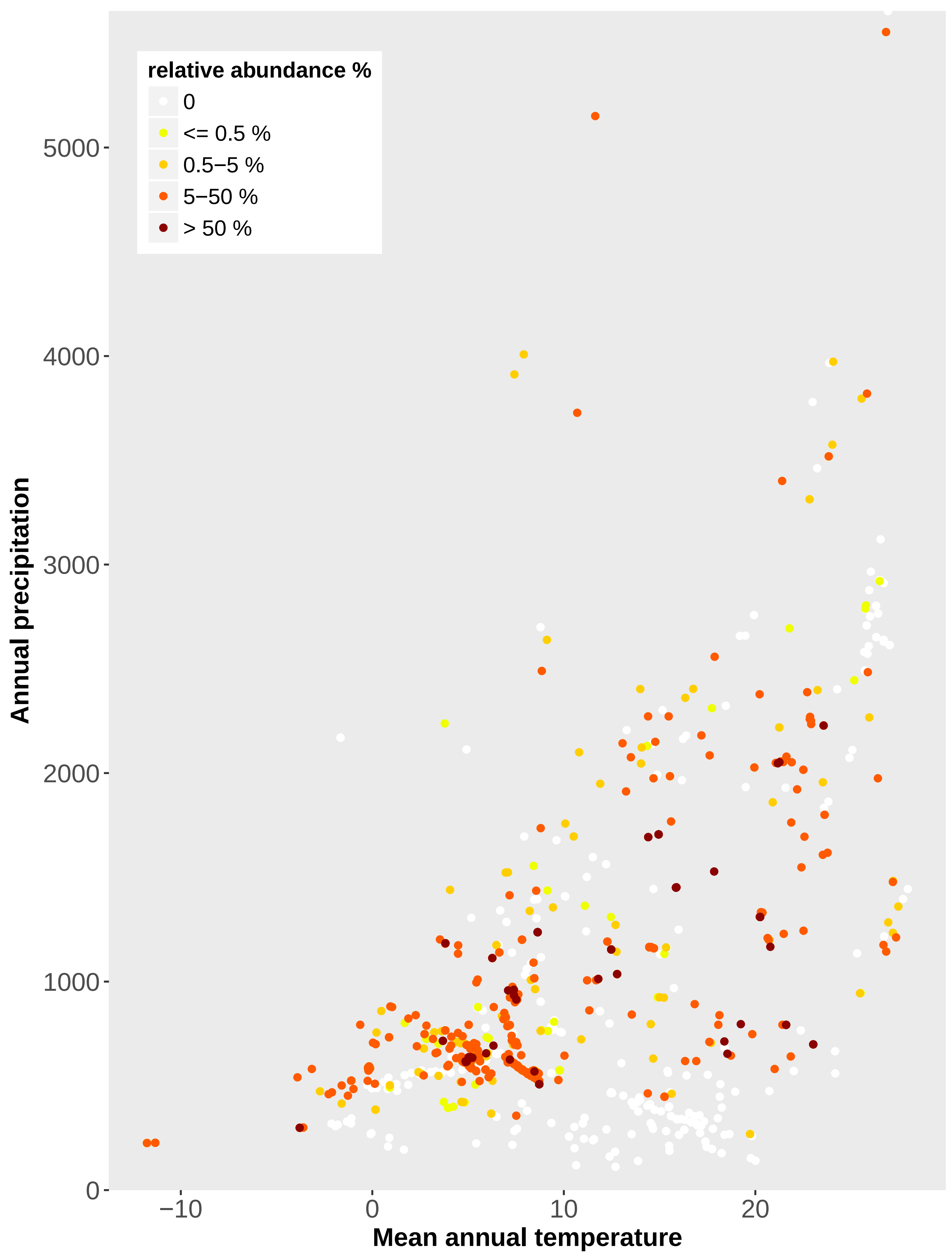

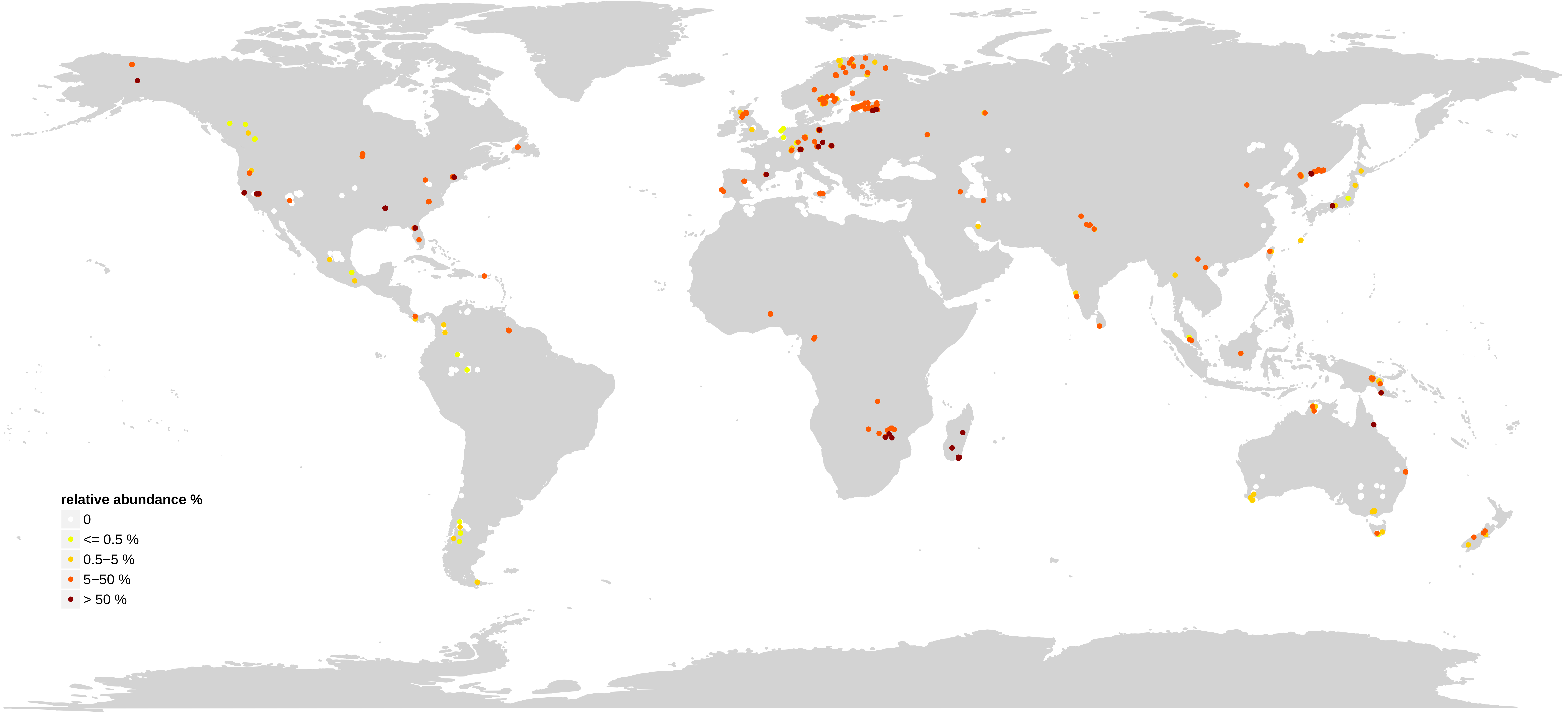

Sebacinaceae

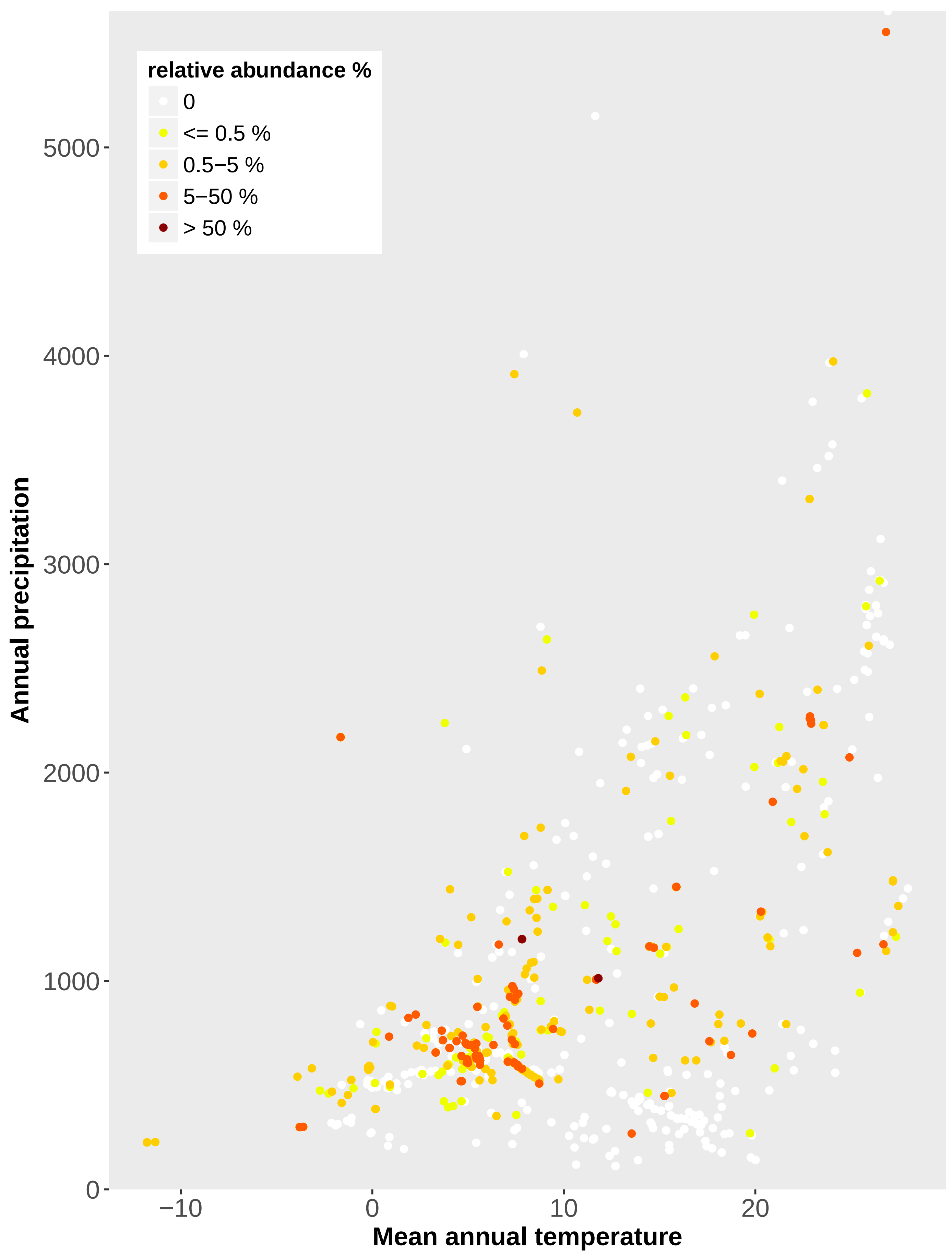

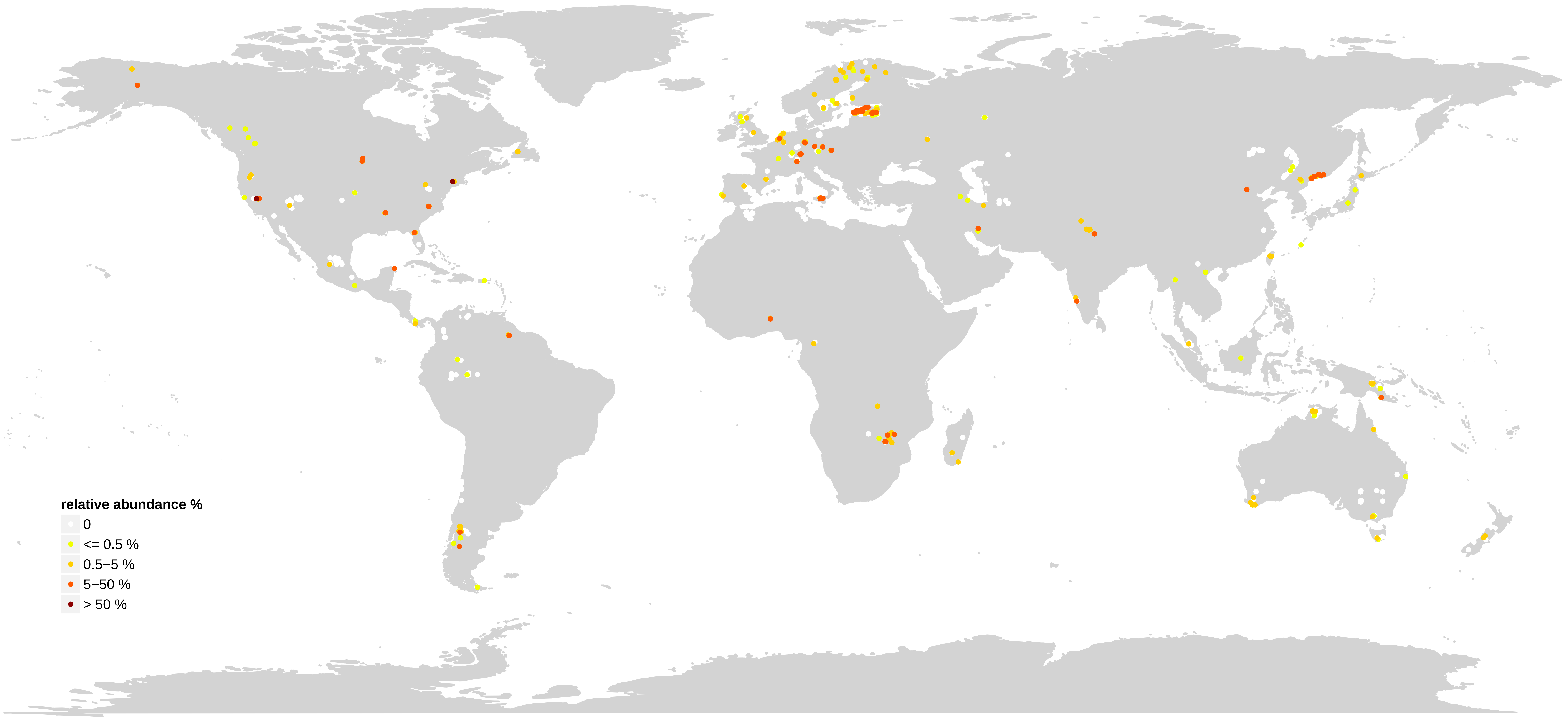

Sporormiaceae

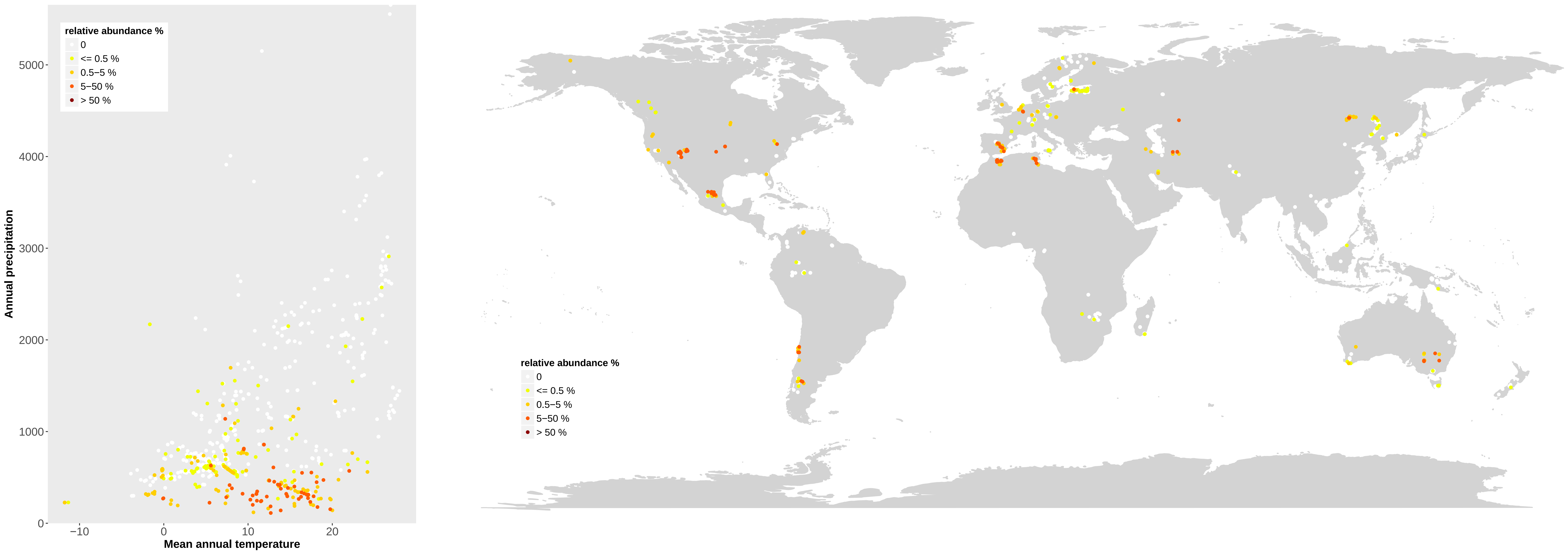

Stropharlaceae

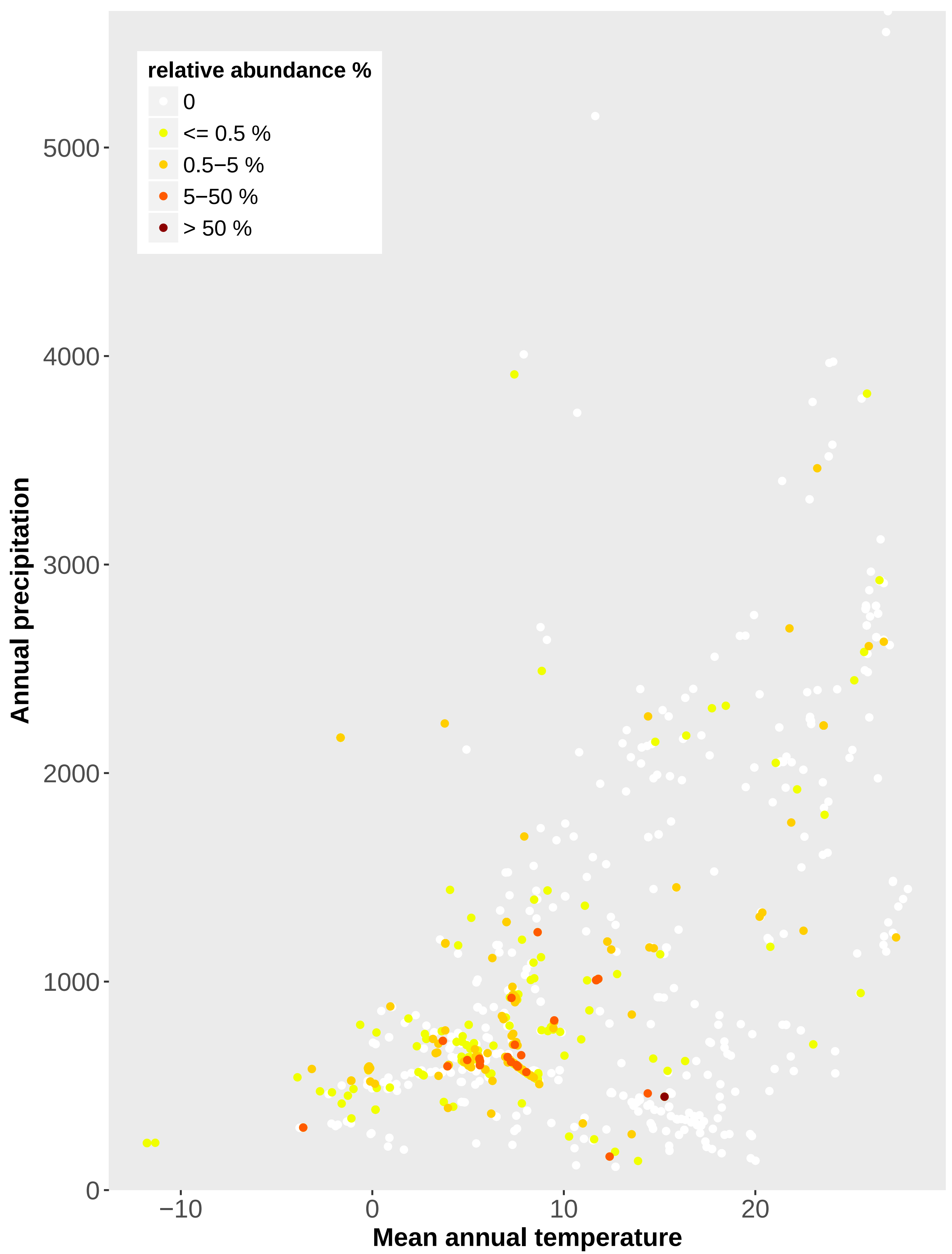

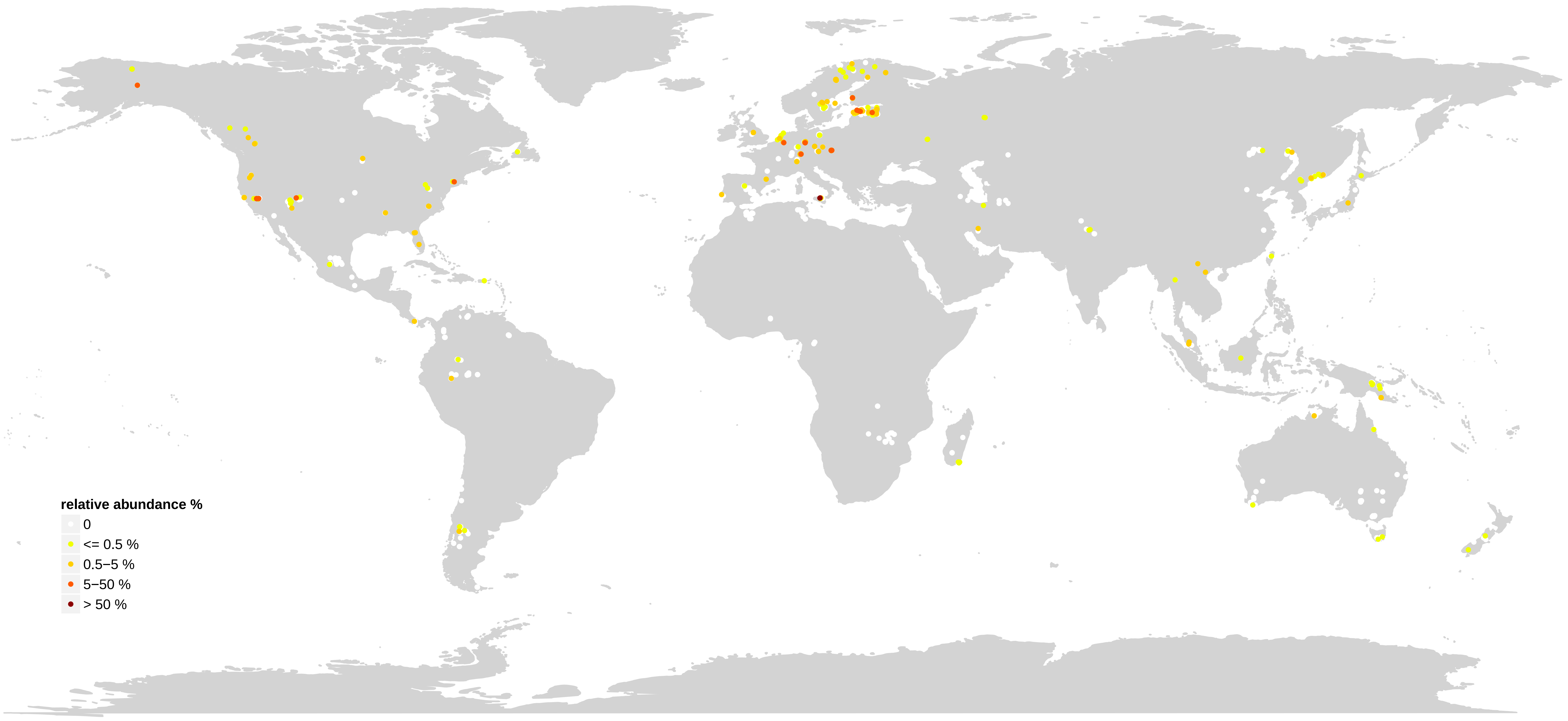

Thelephoraceae

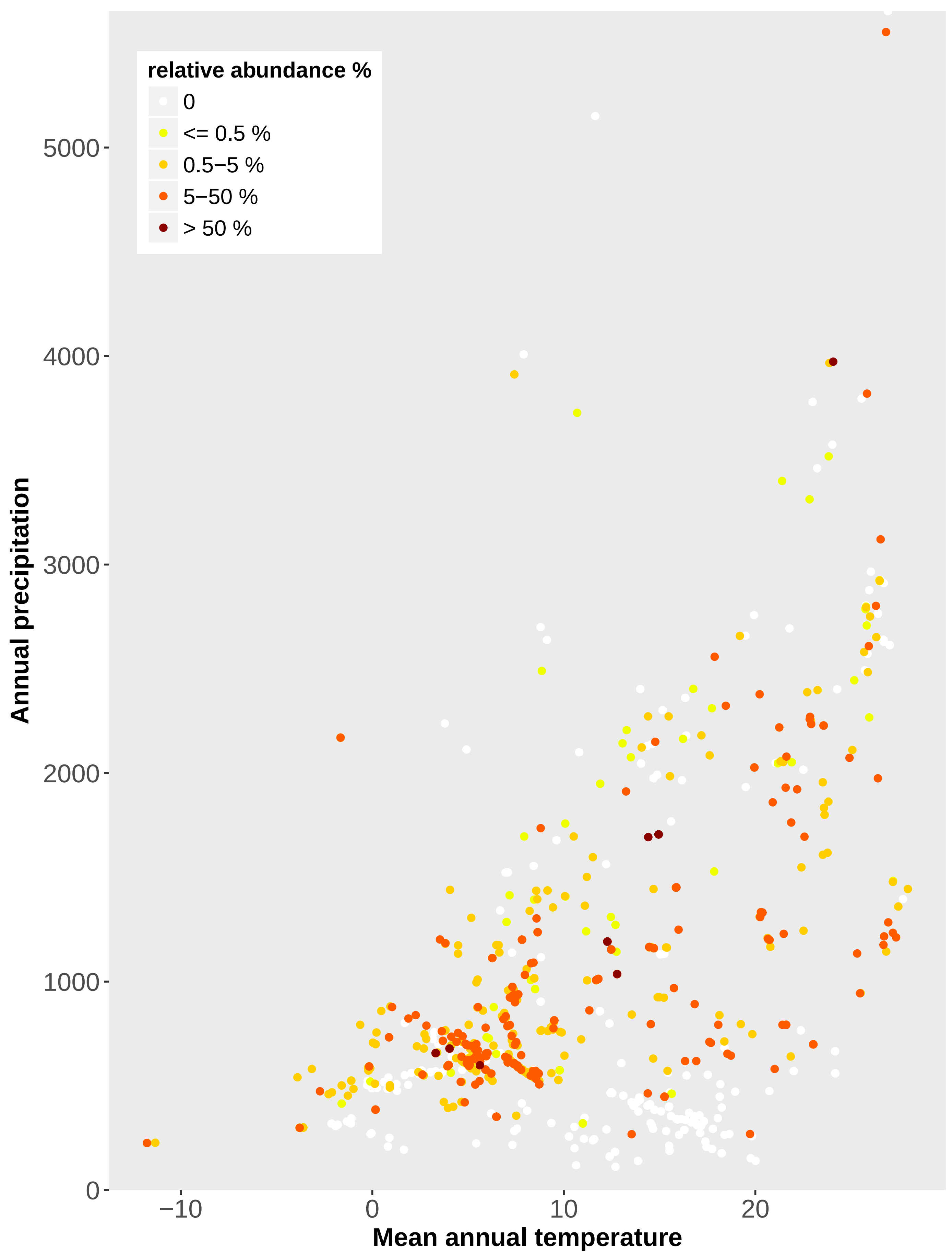

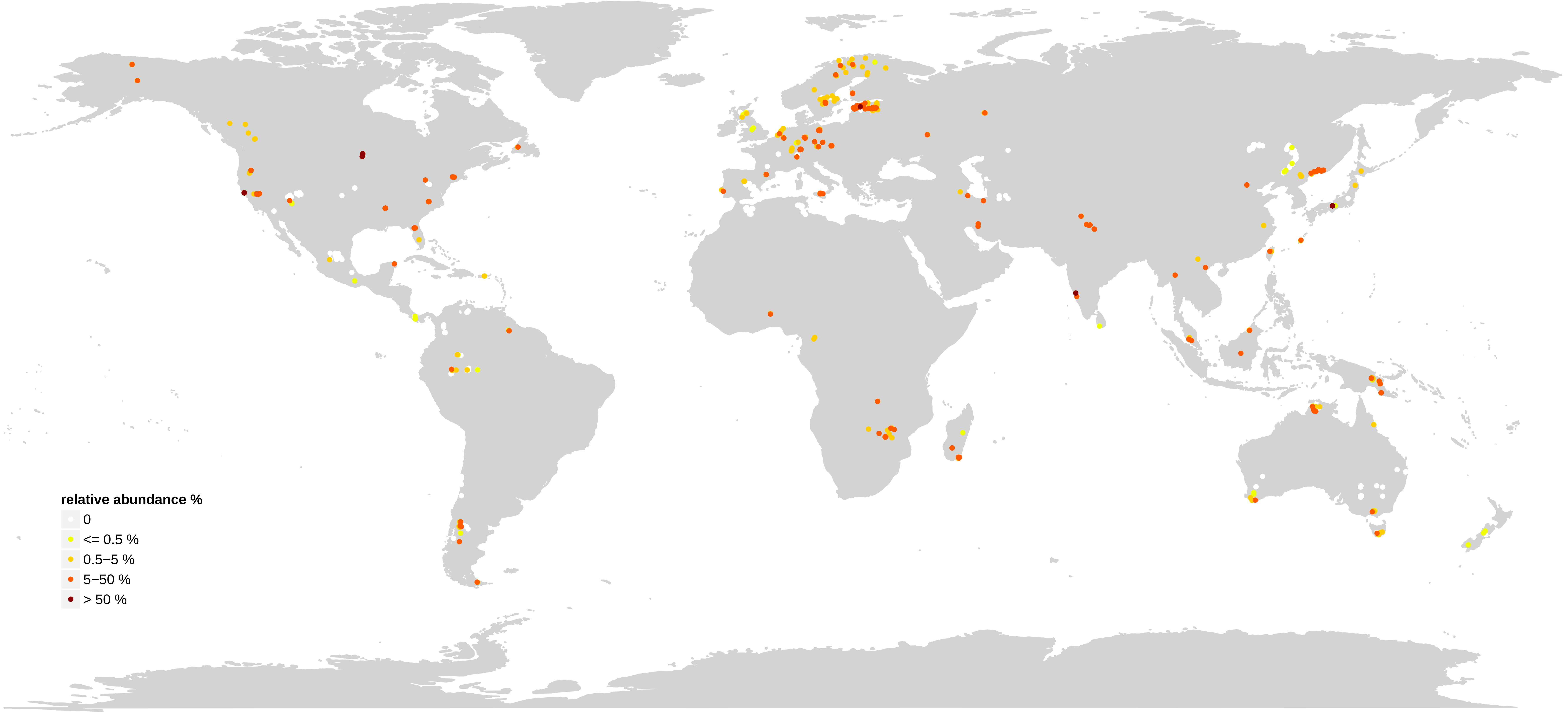

Tremellales_fam_Incertae_sedis

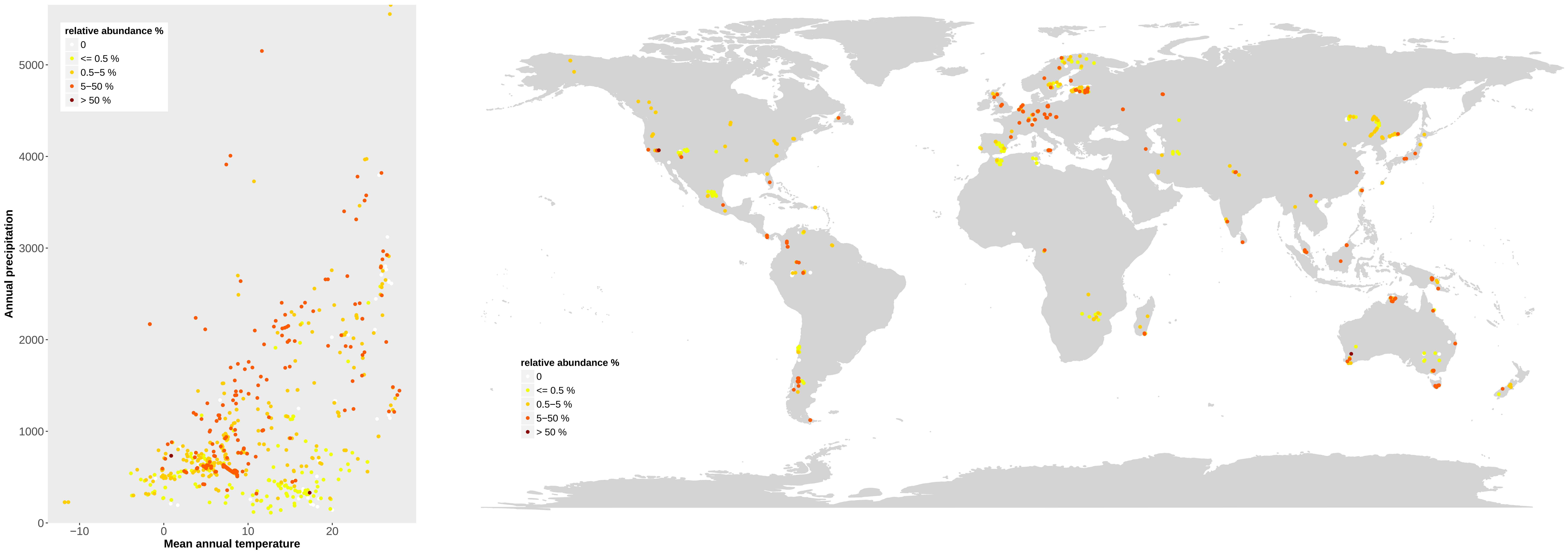

Trichocomaceae

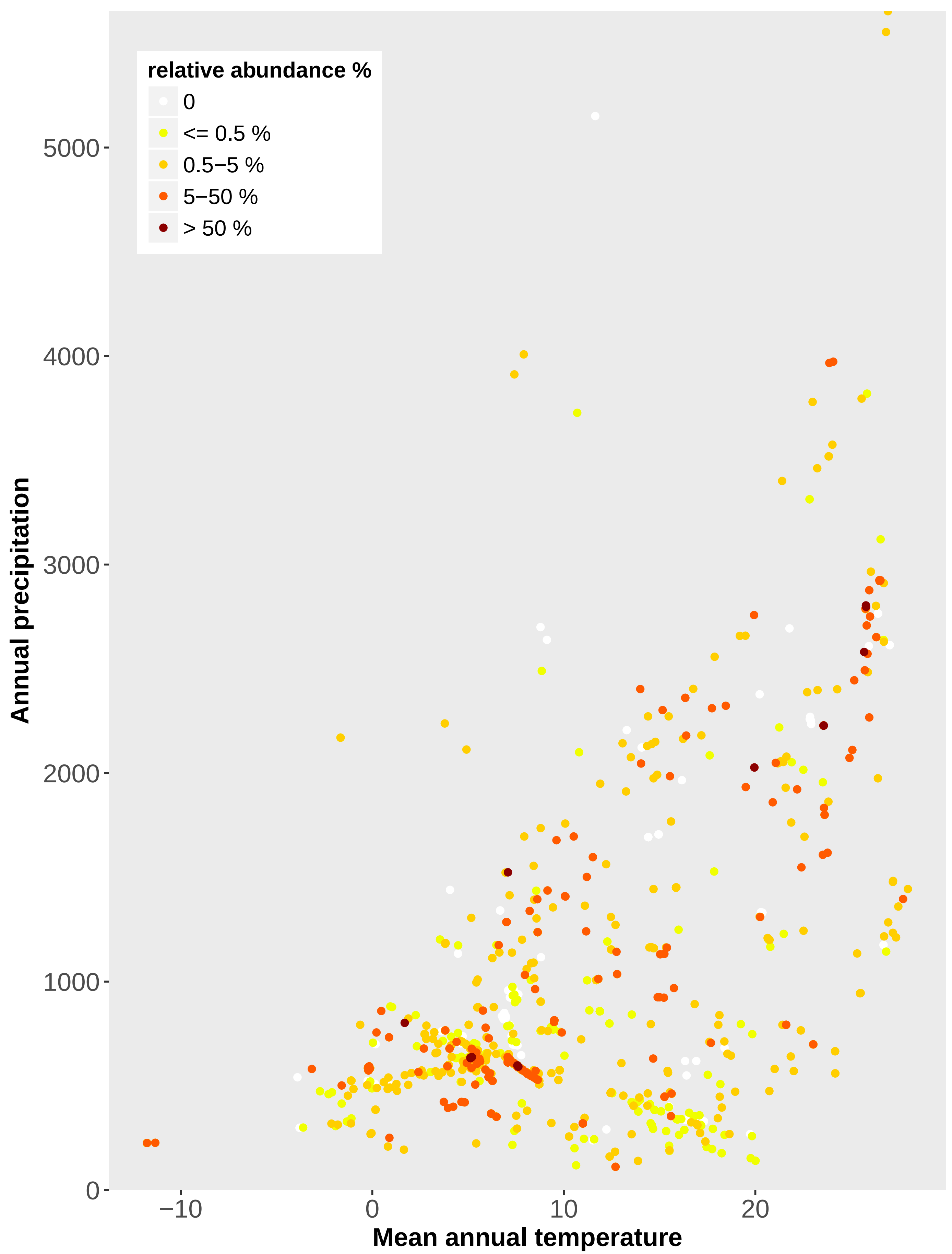

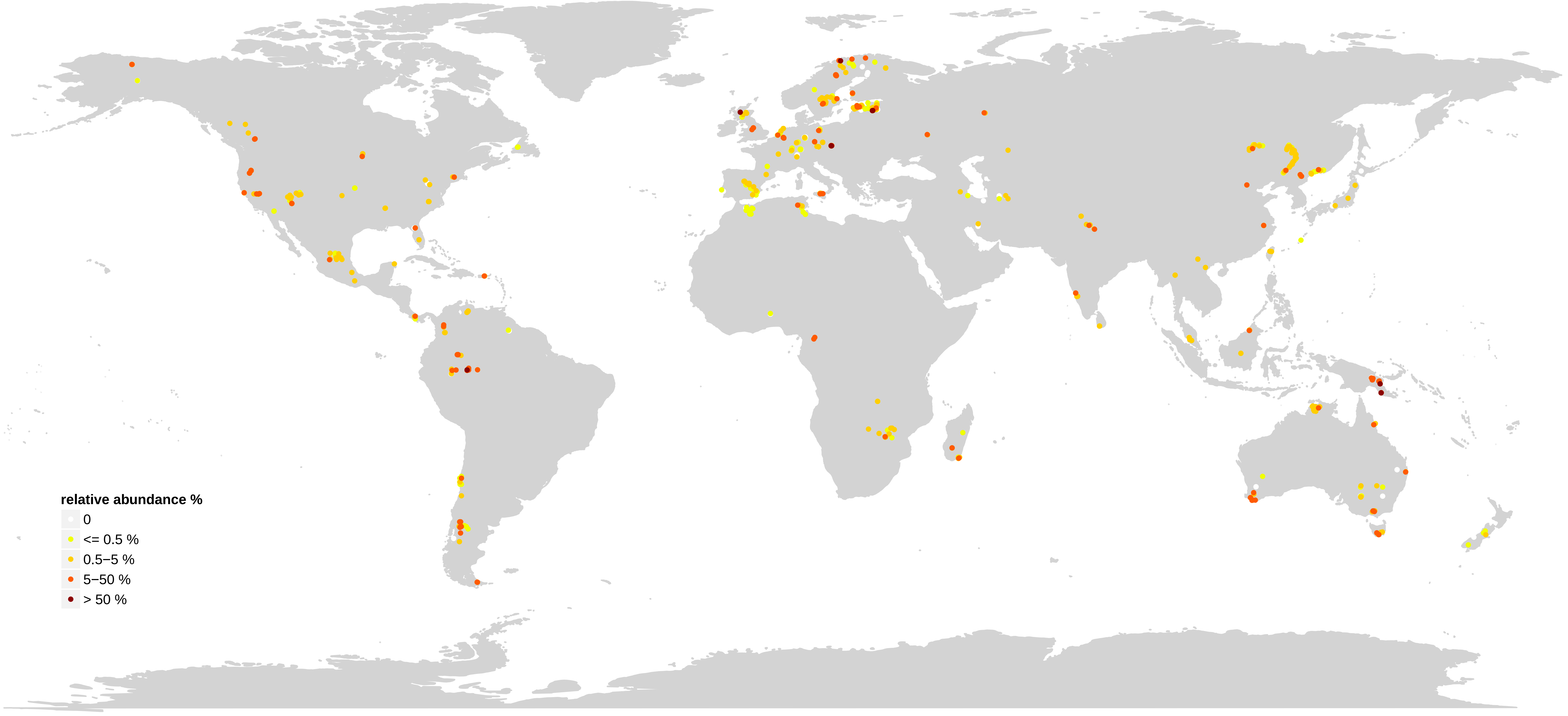

Tricholomataceae

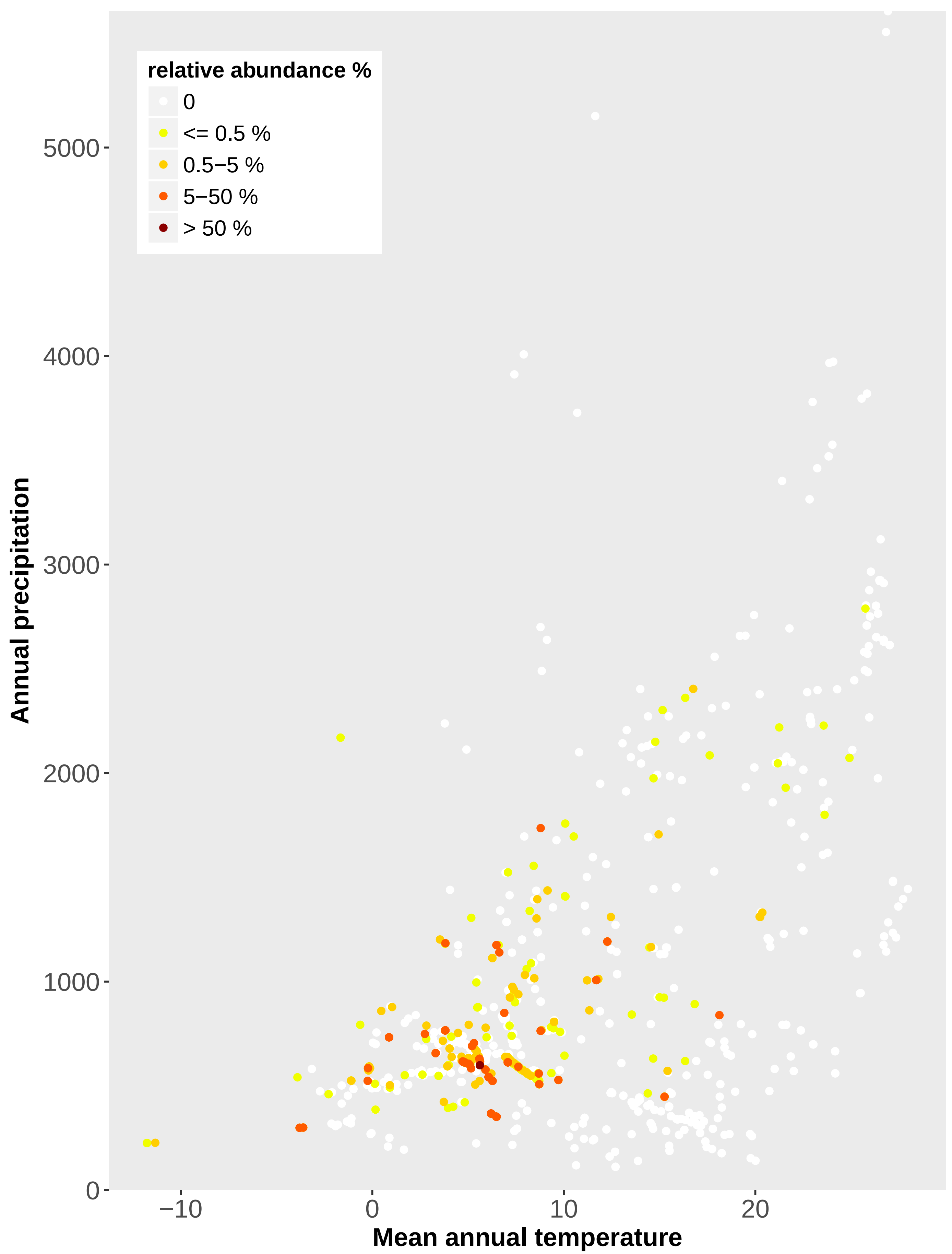

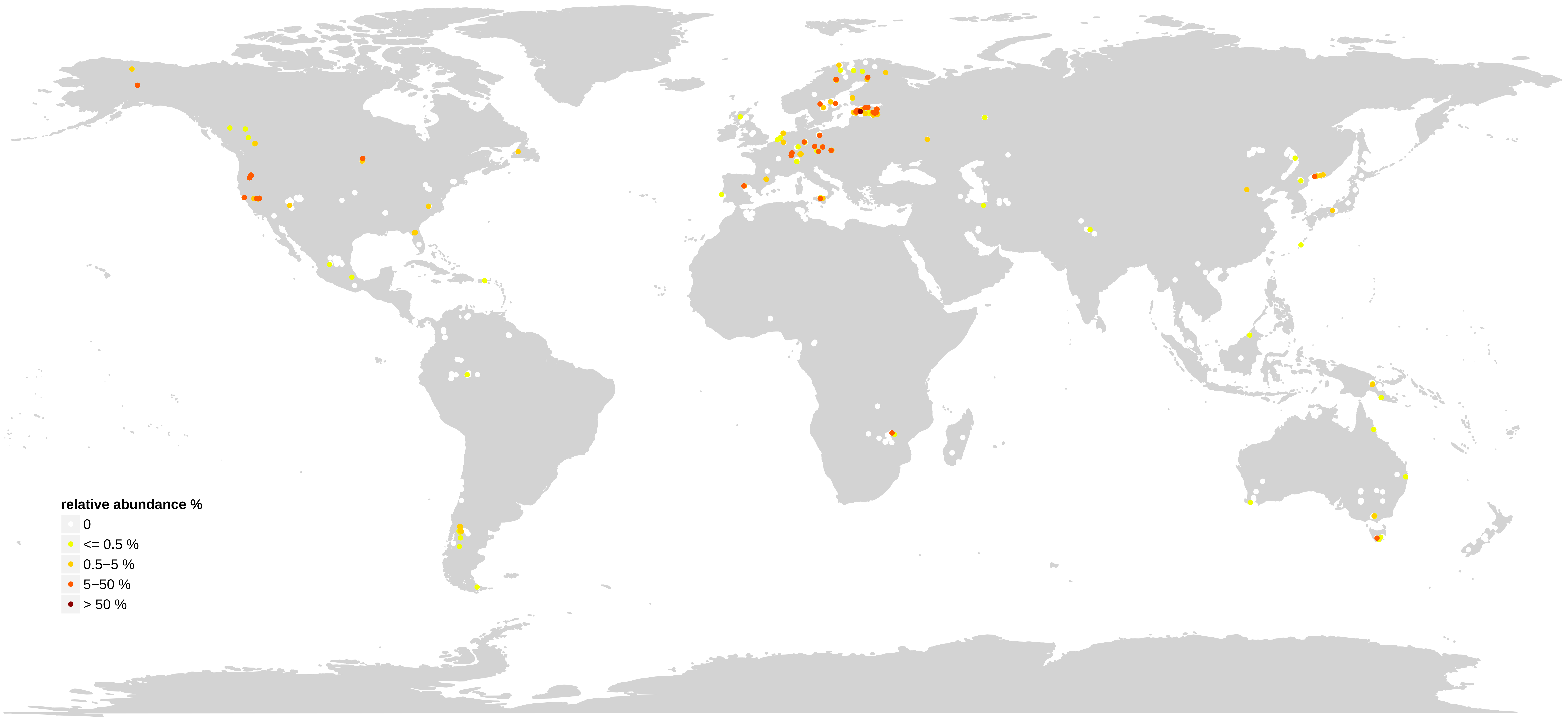

Tuberaceae

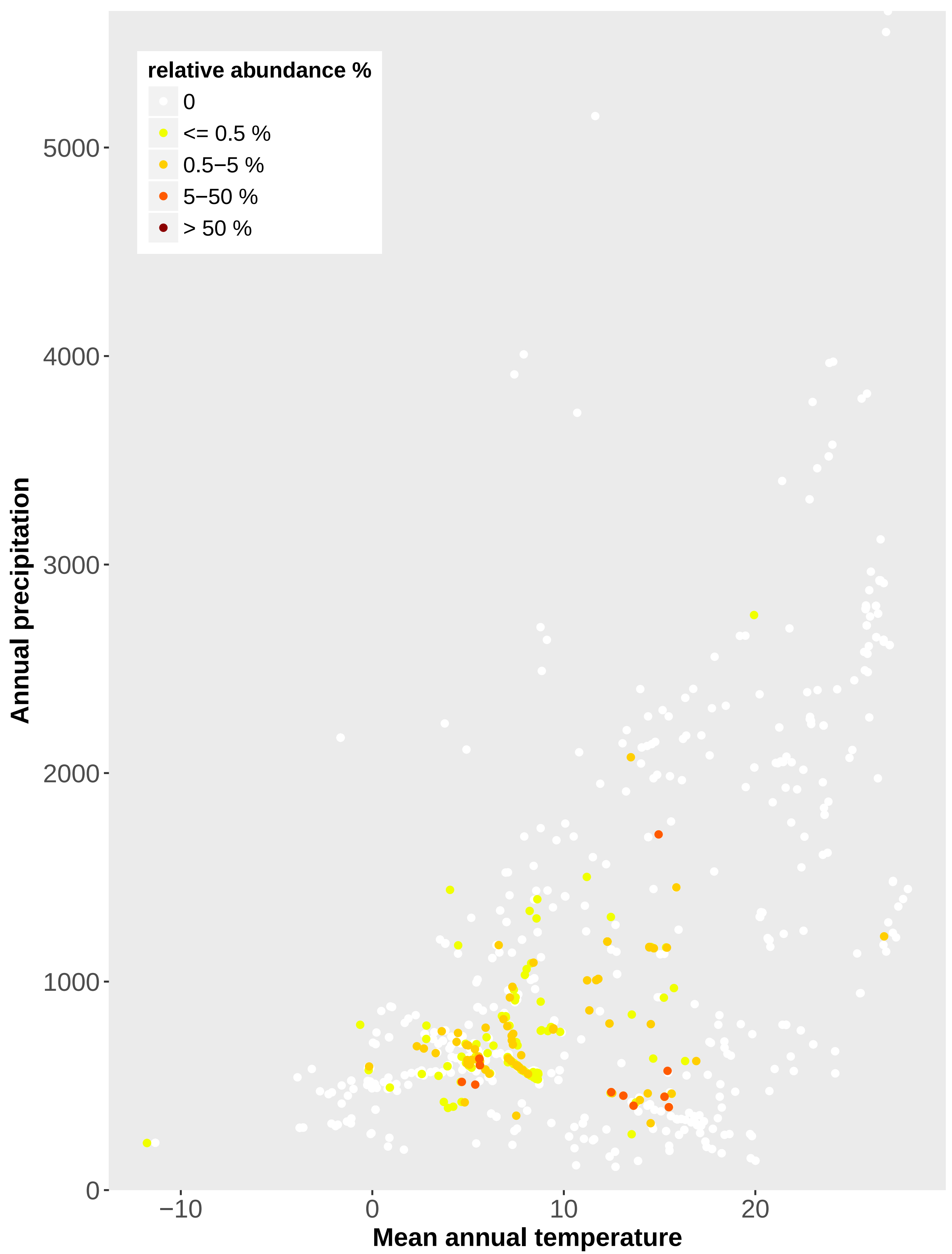
relative abundance %
0
<= 0.5 %
0.5−5 %
5−50 %
> 50 %
Annual precipitation
5000
4000
3000
2000
1000
0
−10
0
10
20
Mean annual temperature

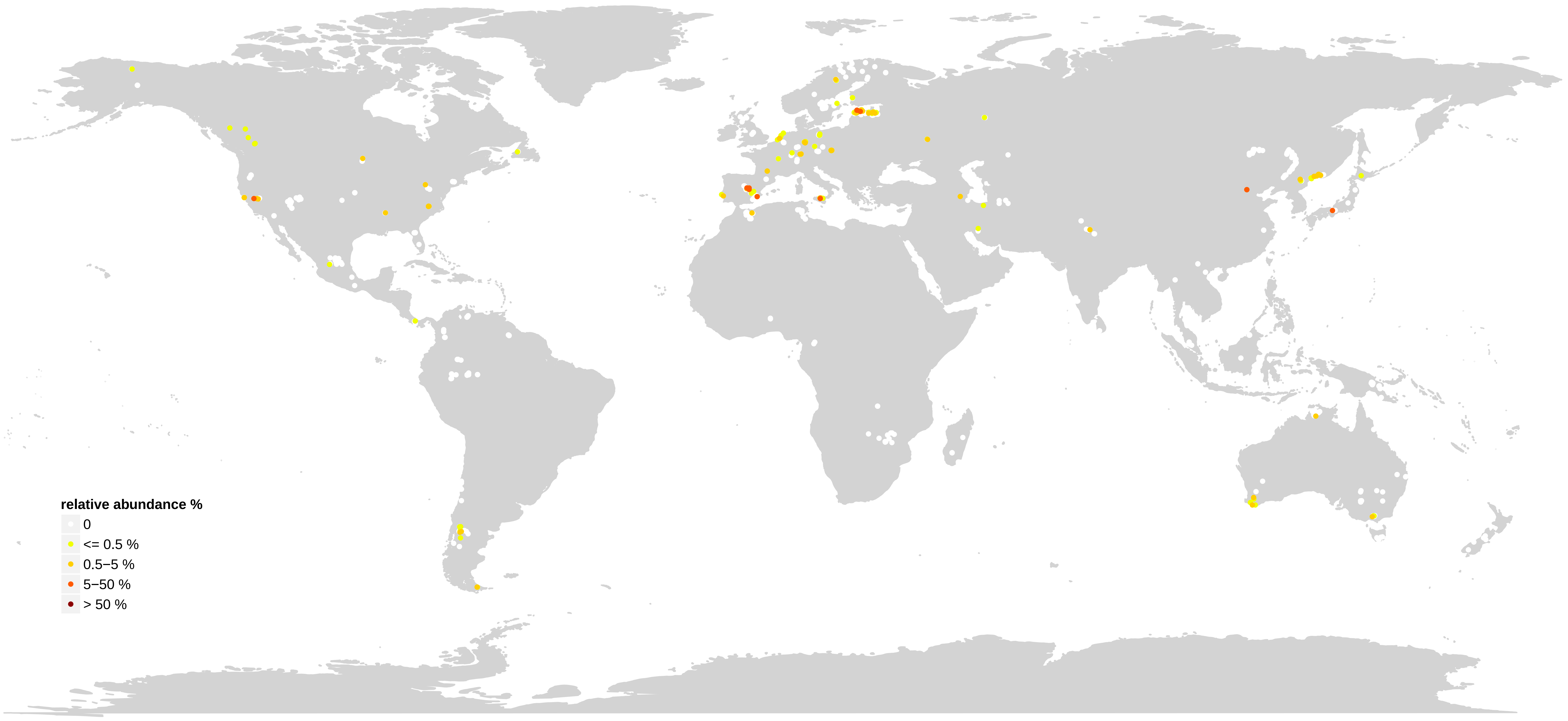
relative abundance %
0
<= 0.5 %
0.5−5 %
5−50 %
> 50 %

Venturiaceae

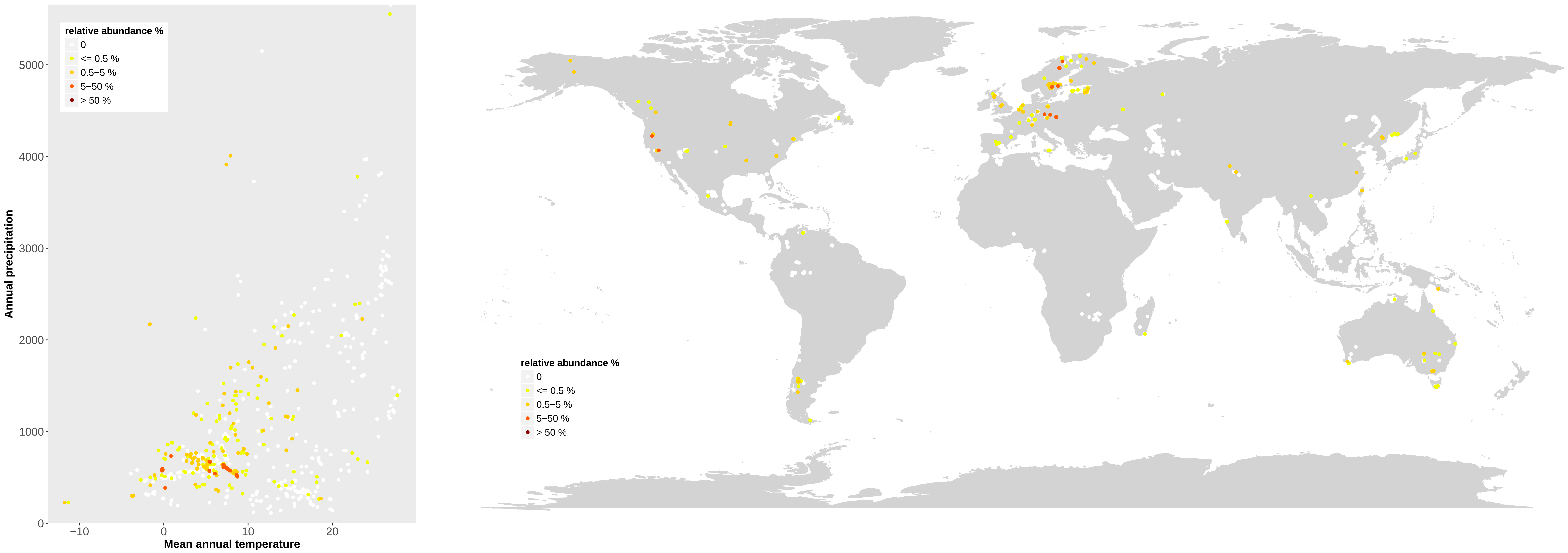

Xylariaceae

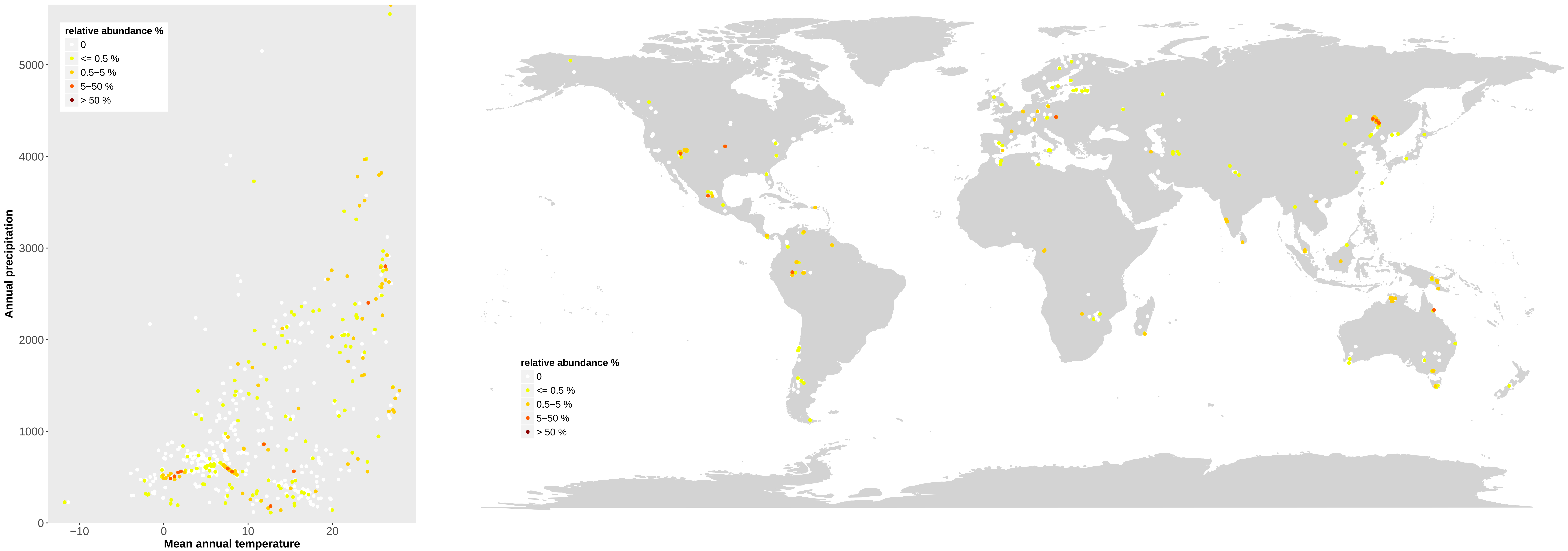

Supplementary Figure 2: Geographic distribution and sequence abundances of the fifty most common fungal families with mean annual temperatures/annual precipitation for the corresponding samples.

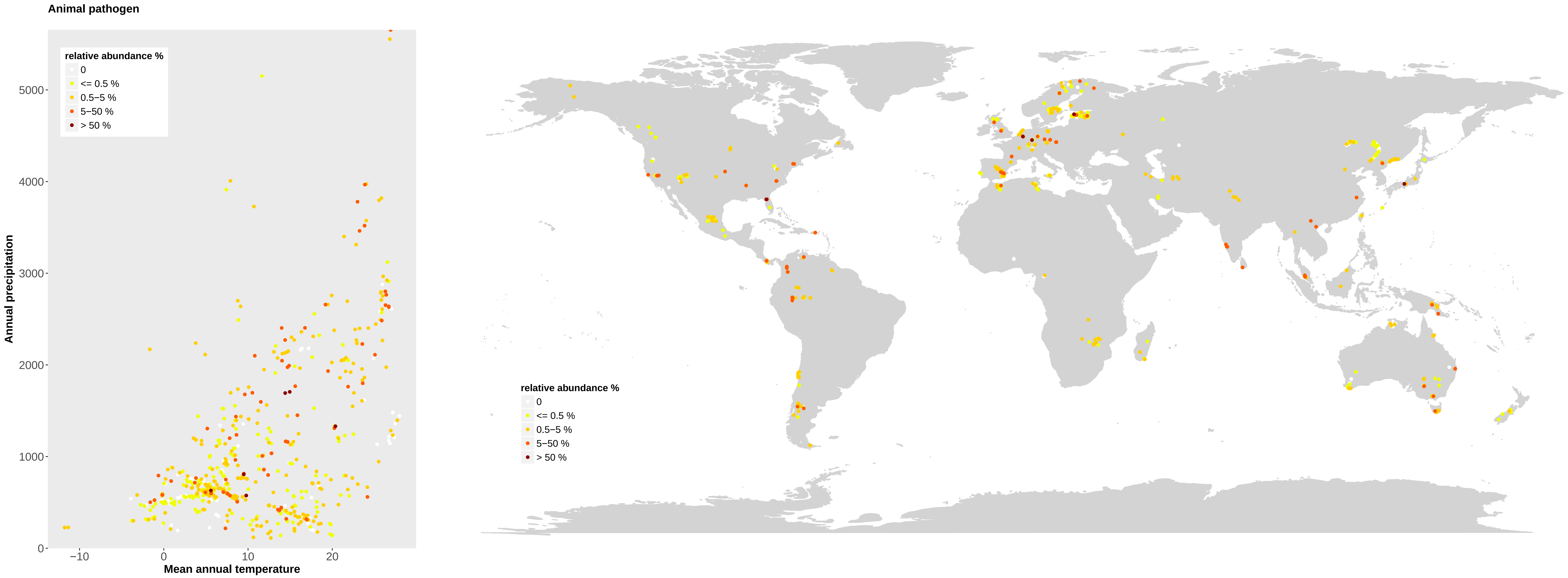
Animal pathogen
relative abundance %
0
<= 0.5 %
0.5–5 %
5–50 %
> 50 %
5000
4000
3000
2000
1000
0
−10
0
10
20
Annual precipitation
Mean annual temperature
relative abundance %
0
<= 0.5 %
0.5–5 %
5–50 %
> 50 %

Arbuscular Mycorrhizal

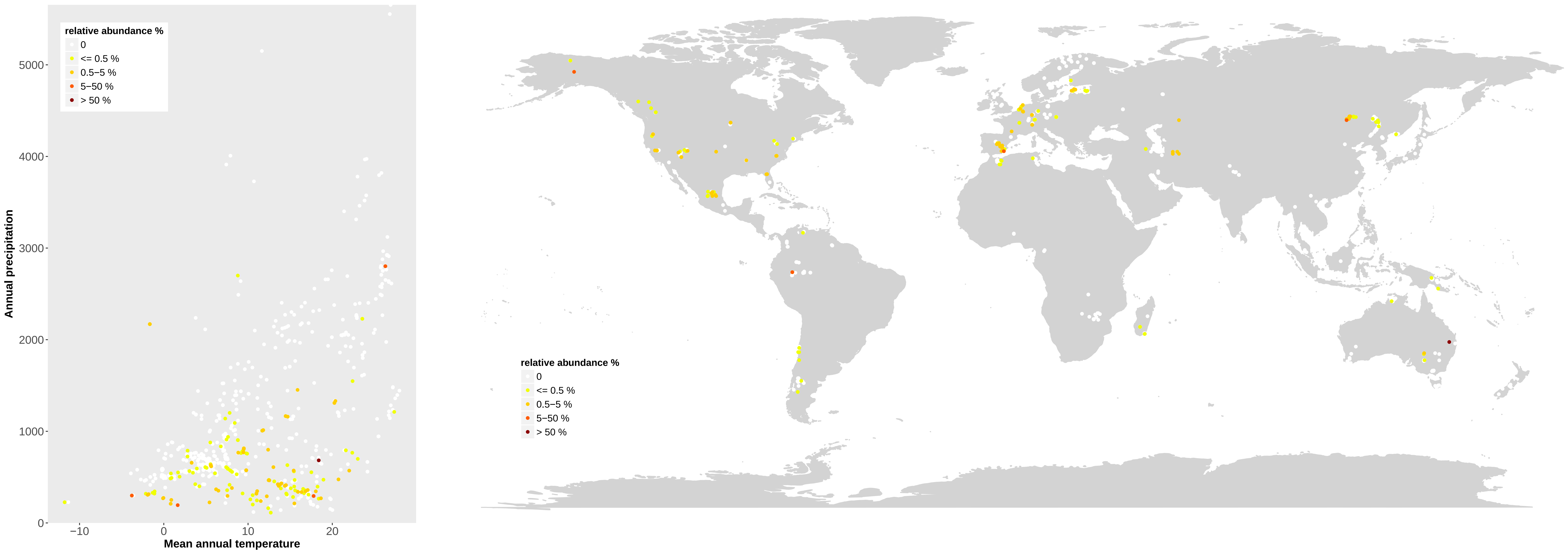

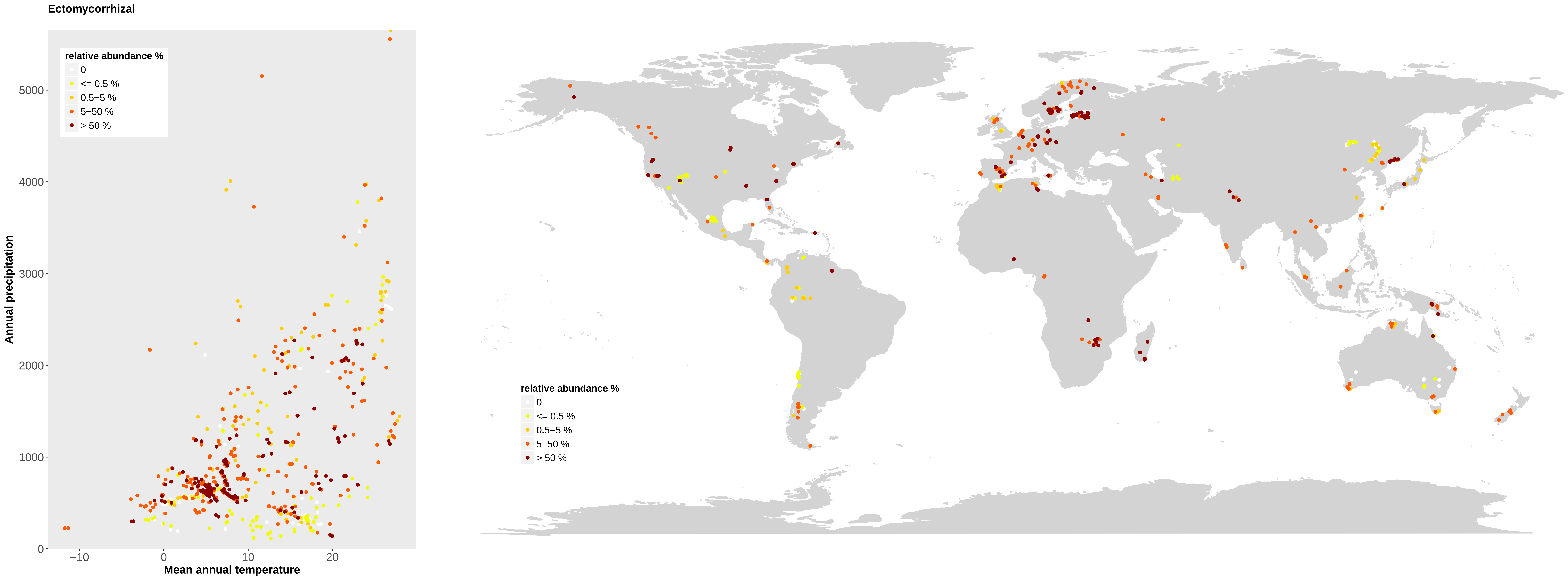
Ectomycorrhizal
relative abundance %
0
<= 0.5 %
0.5–5 %
5–50 %
> 50 %
Annual precipitation
0
1000
2000
3000
4000
5000
−10
0
10
20
Mean annual temperature
relative abundance %
0
<= 0.5 %
0.5–5 %
5–50 %
> 50 %

Endophyte

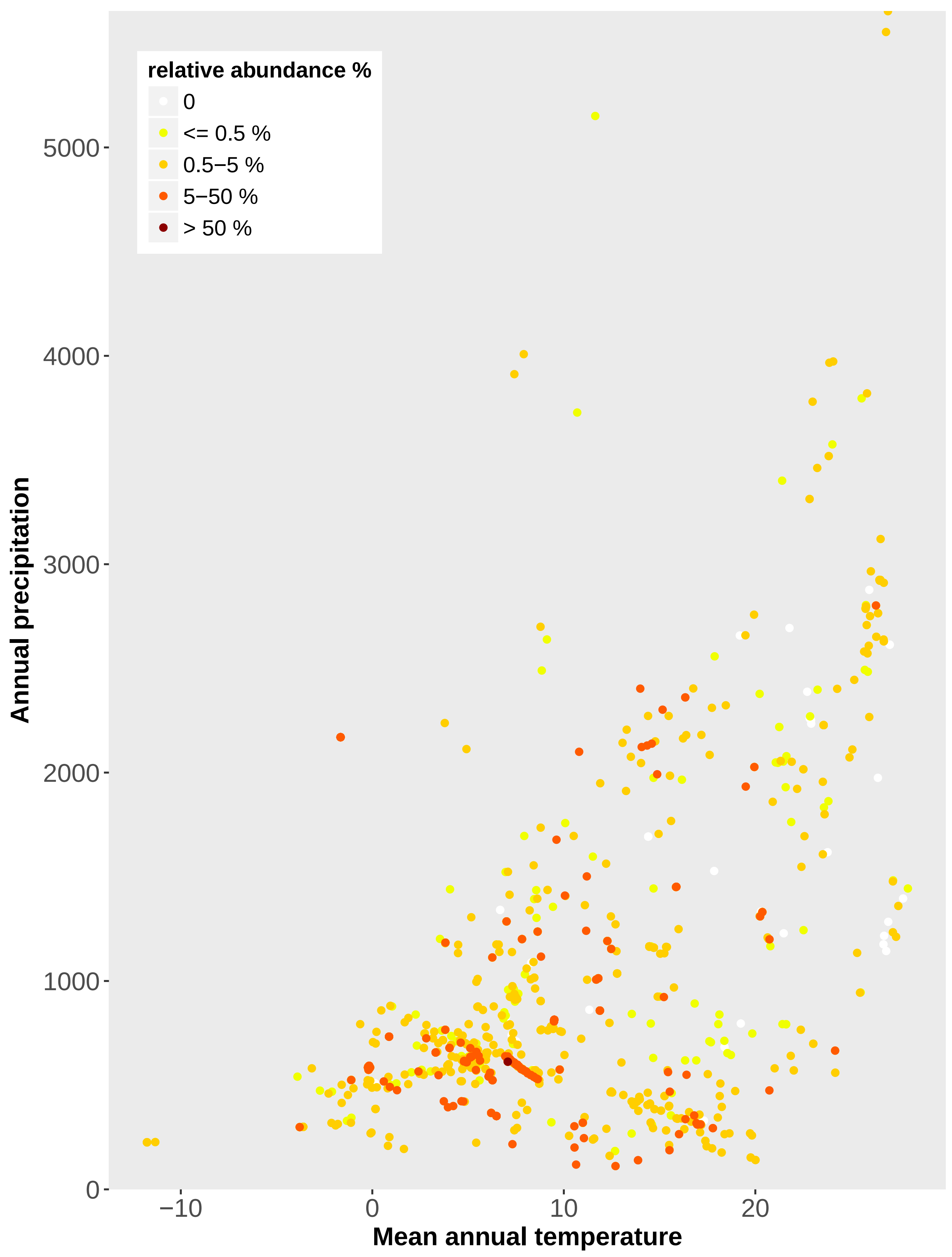
relative abundance %
0
<= 0.5 %
0.5–5 %
5–50 %
> 50 %
Annual precipitation
Mean annual temperature
0
1000
2000
3000
4000
5000
−10
0
10
20

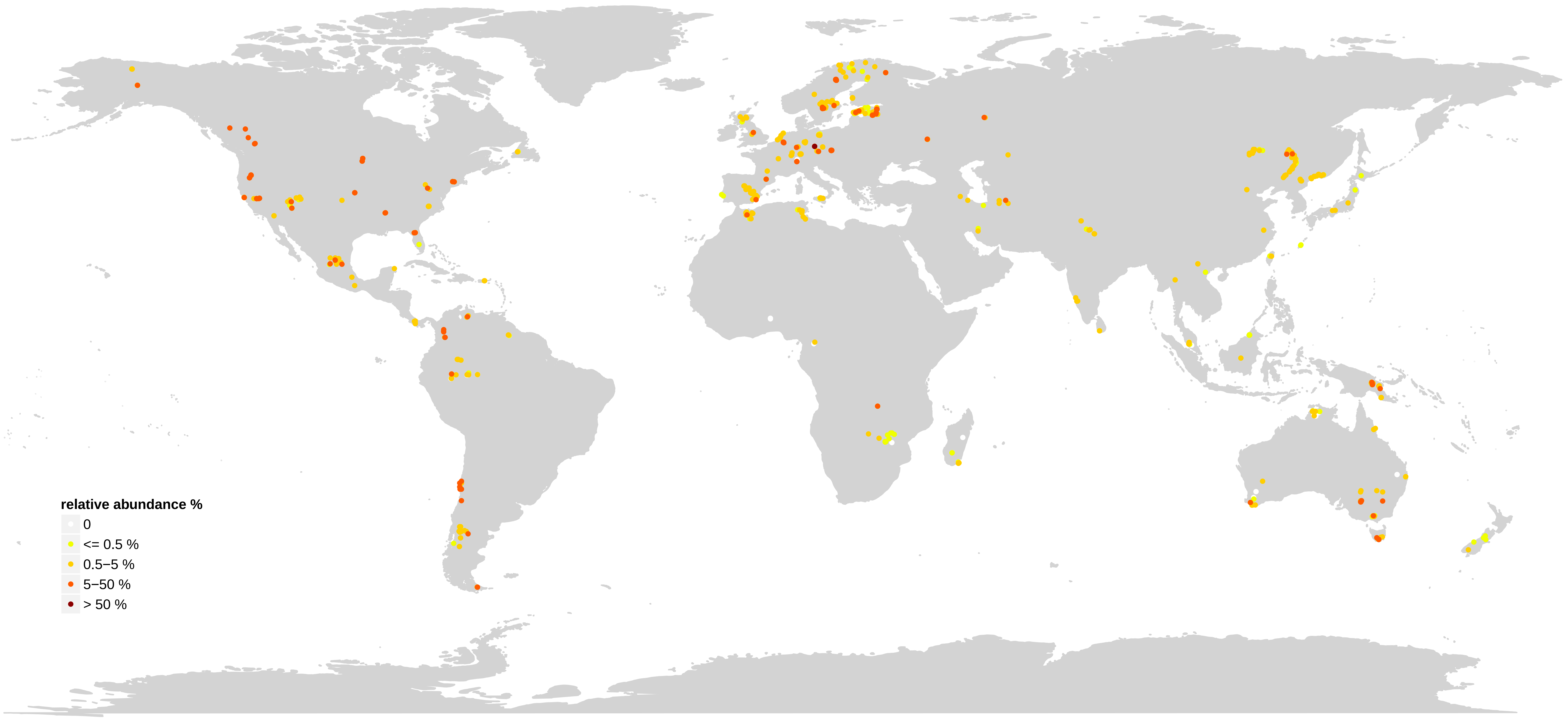
relative abundance %
0
<= 0.5 %
0.5–5 %
5–50 %
> 50 %

Ericoid Mycorrhizal

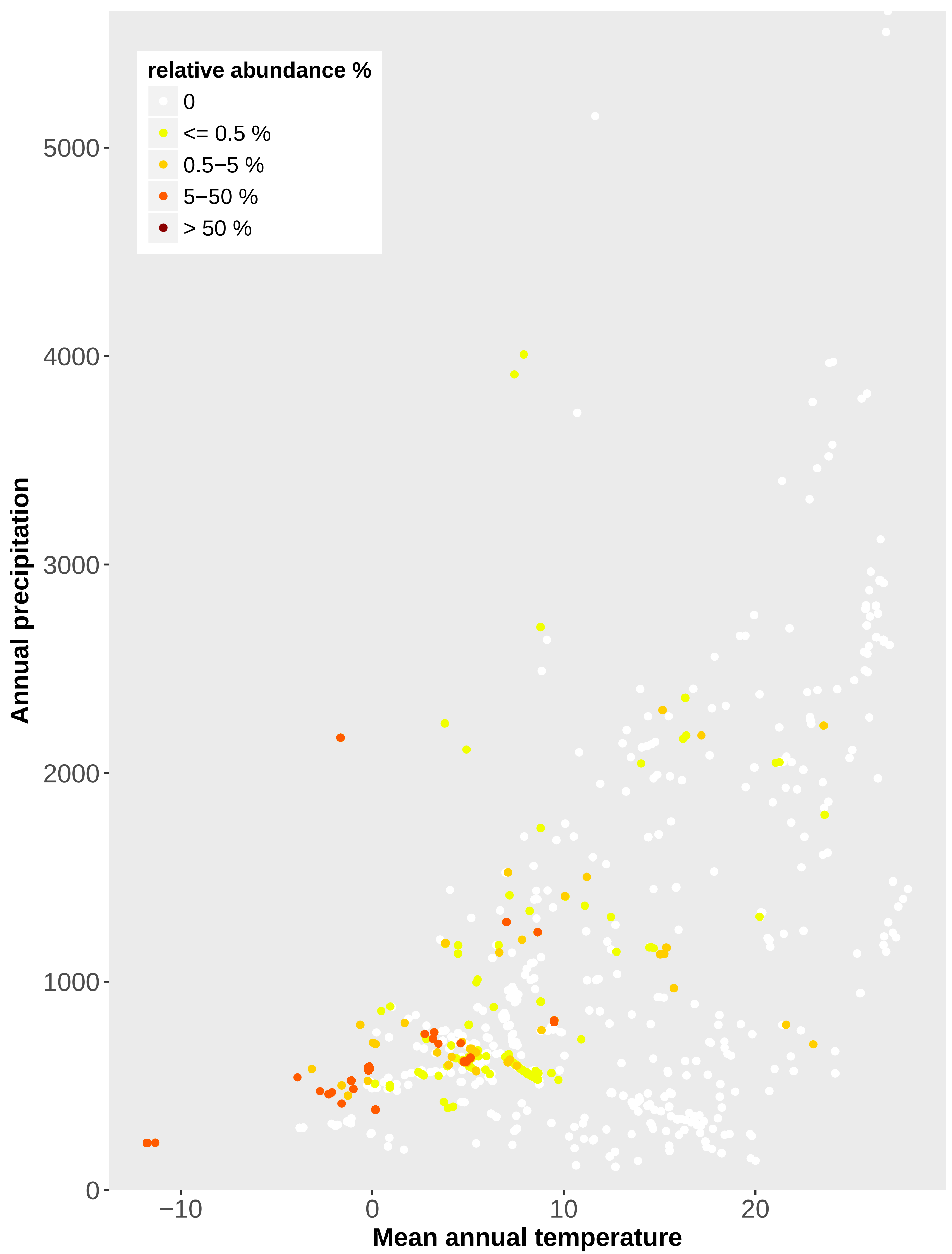

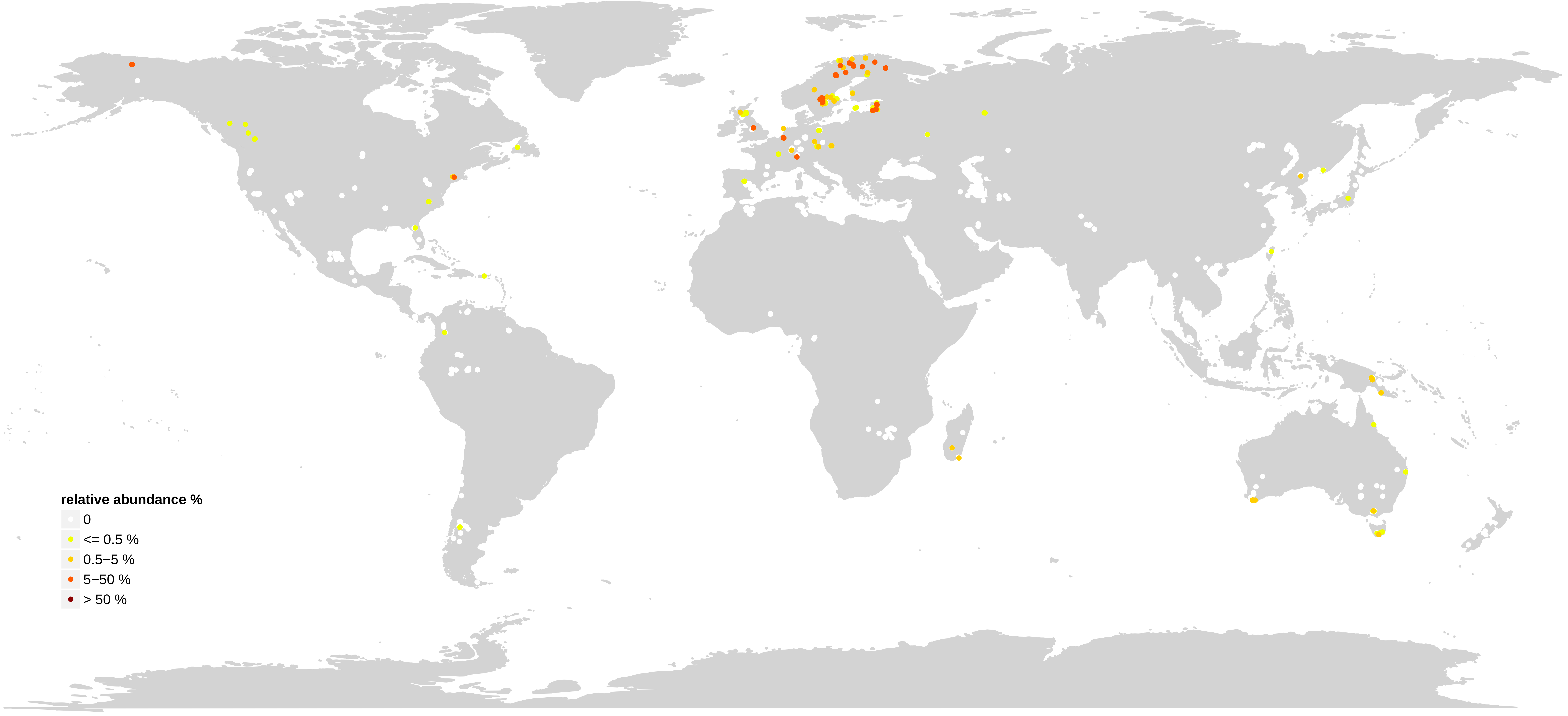

Foliar Epiphyte

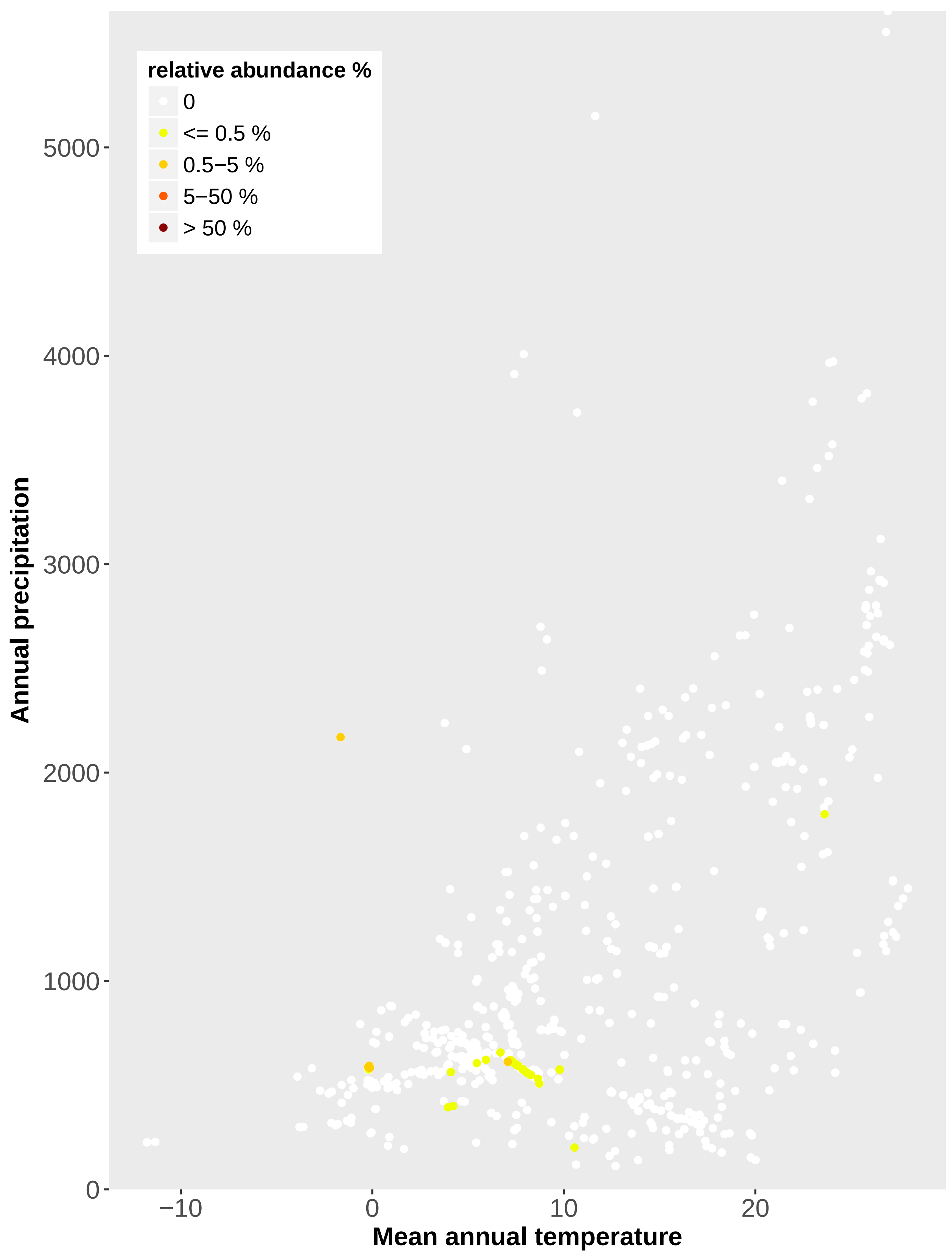

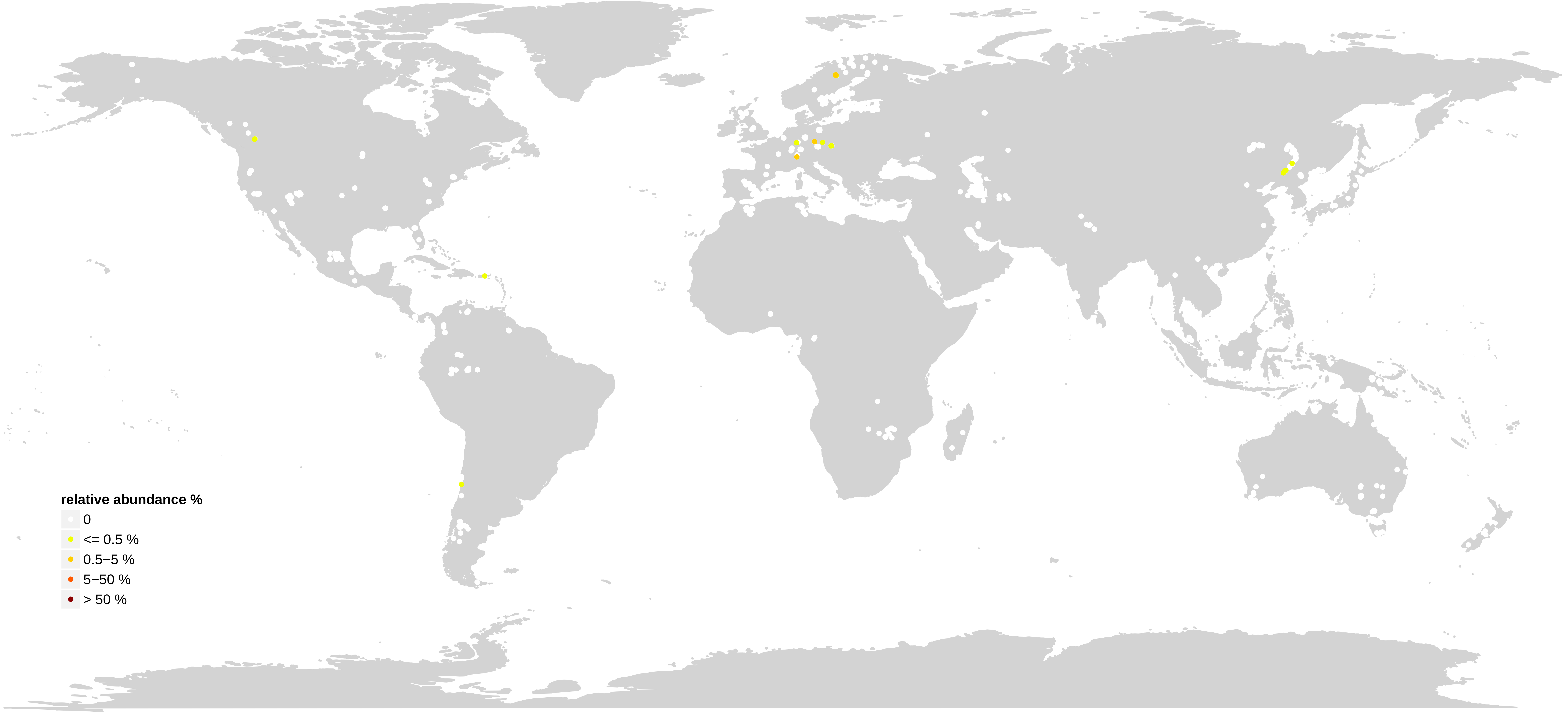

Lichenicolous

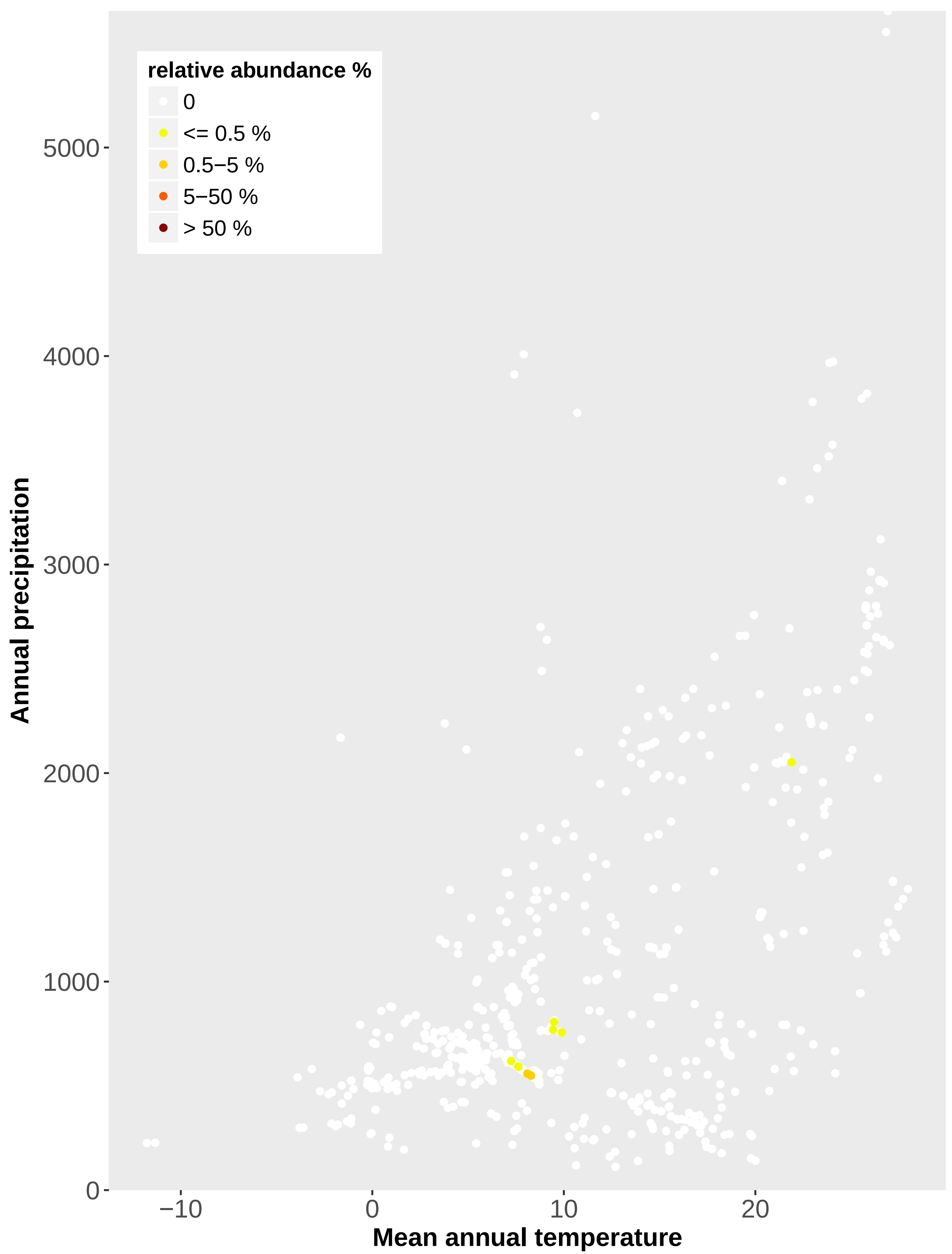

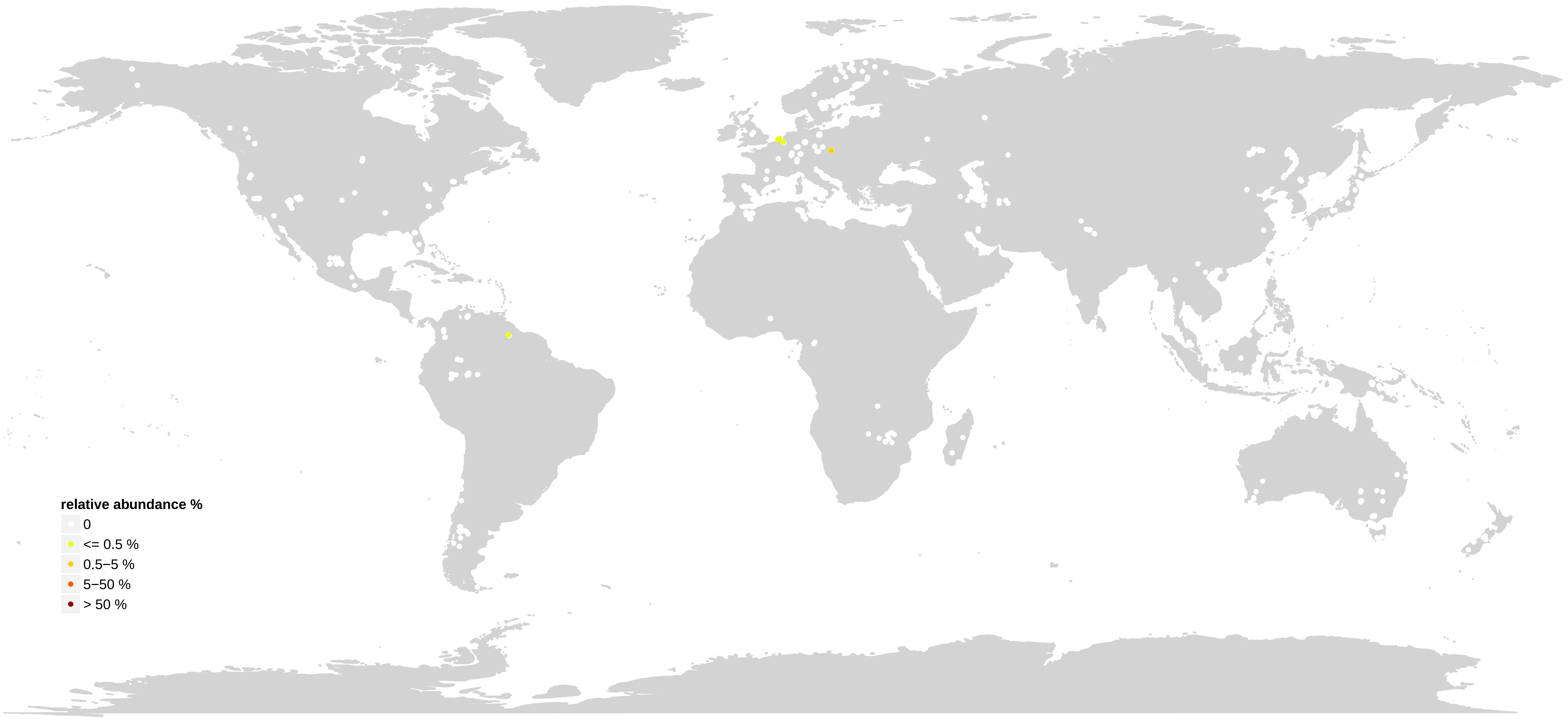

Lichenized

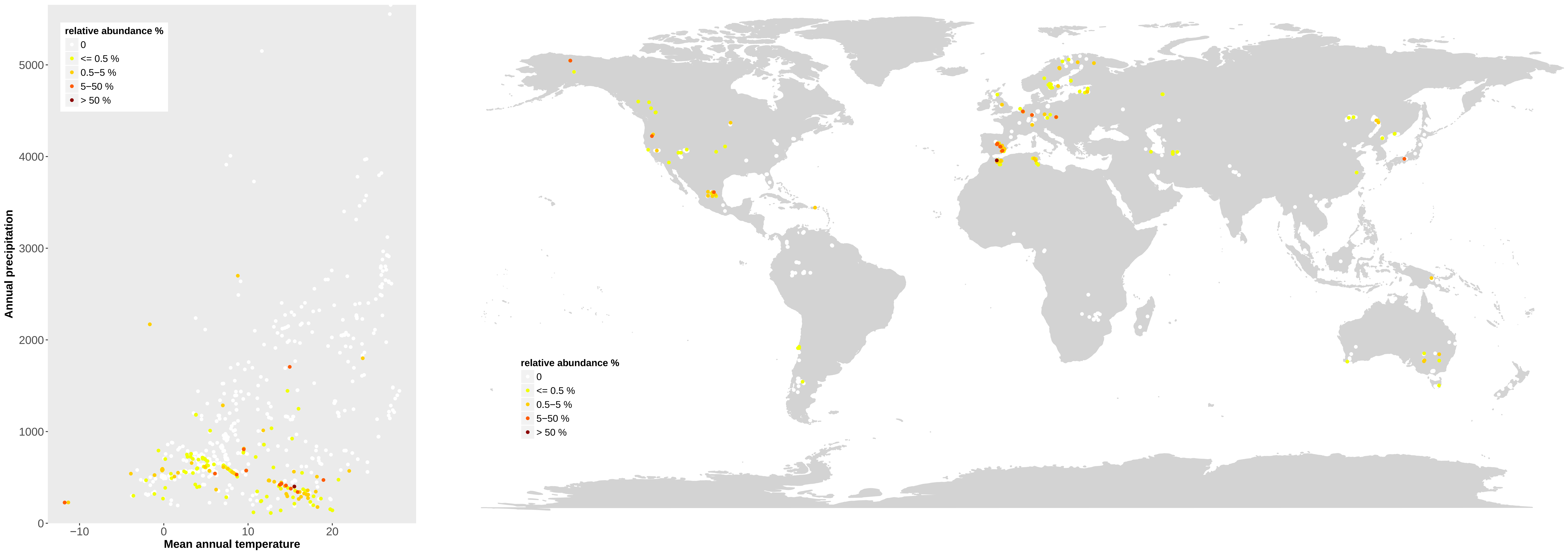
relative abundance %
0
<= 0.5 %
0.5–5 %
5–50 %
> 50 %
Annual precipitation
0
1000
2000
3000
4000
5000
−10
0
10
20
Mean annual temperature
relative abundance %
0
<= 0.5 %
0.5–5 %
5–50 %
> 50 %

Mycoparasite

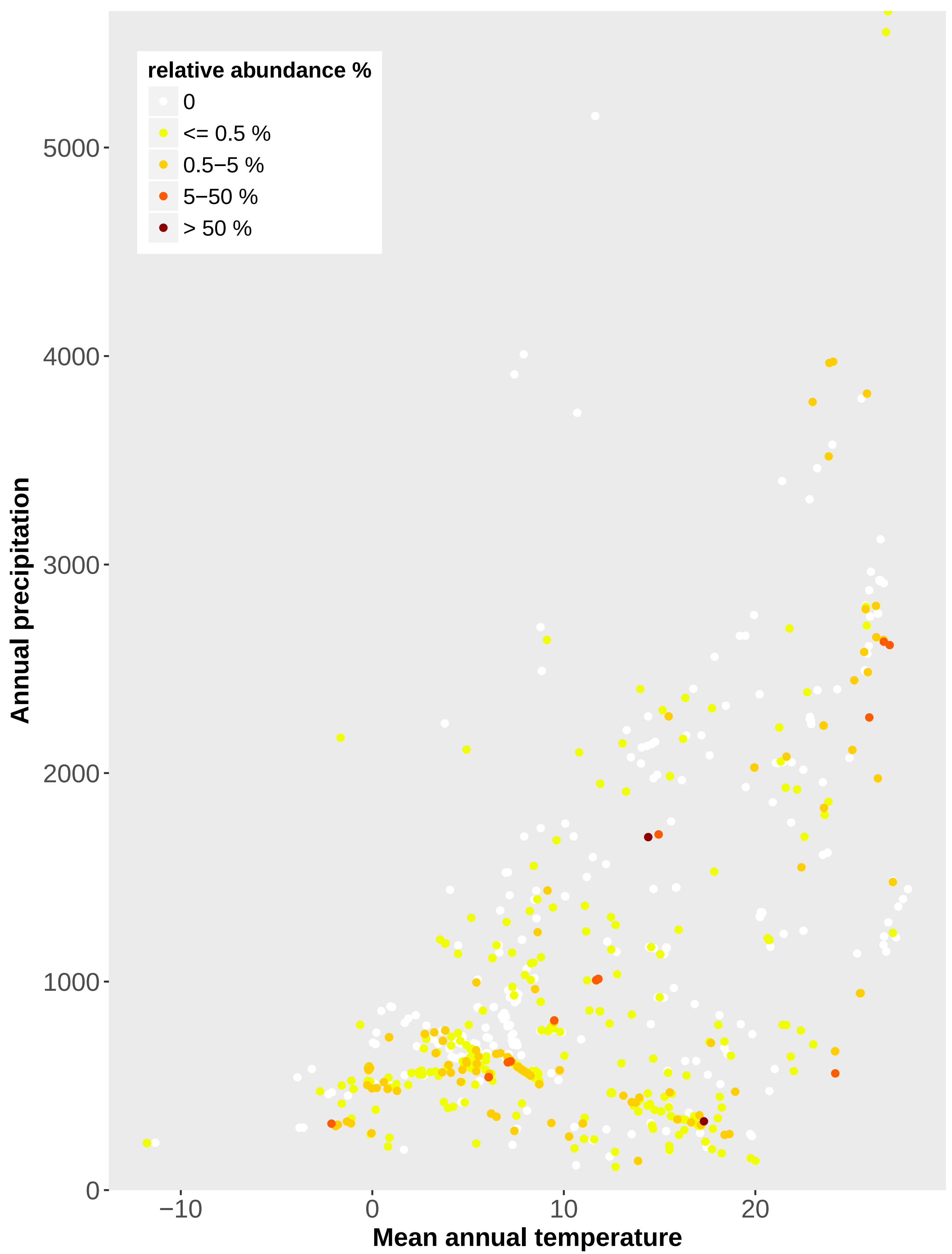

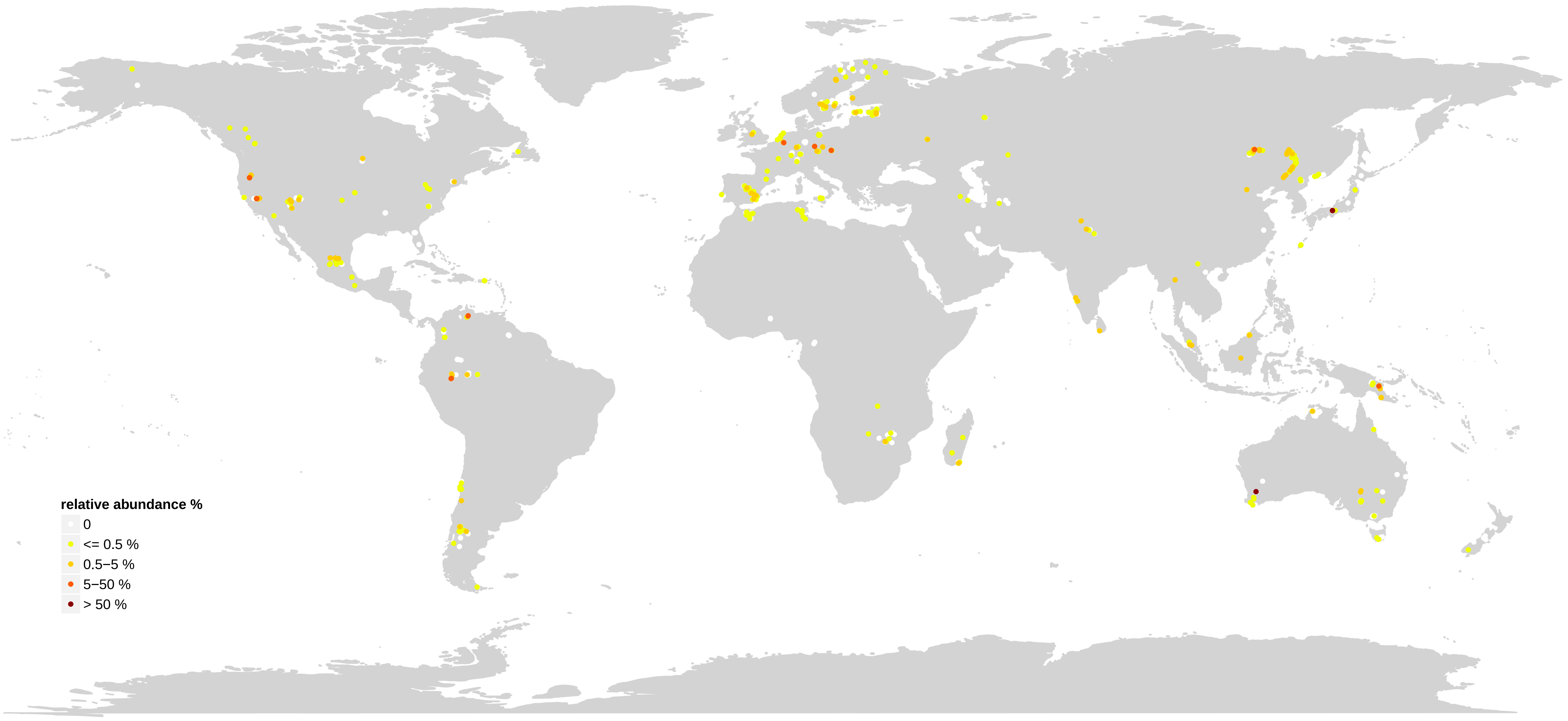

Plant Pathogen

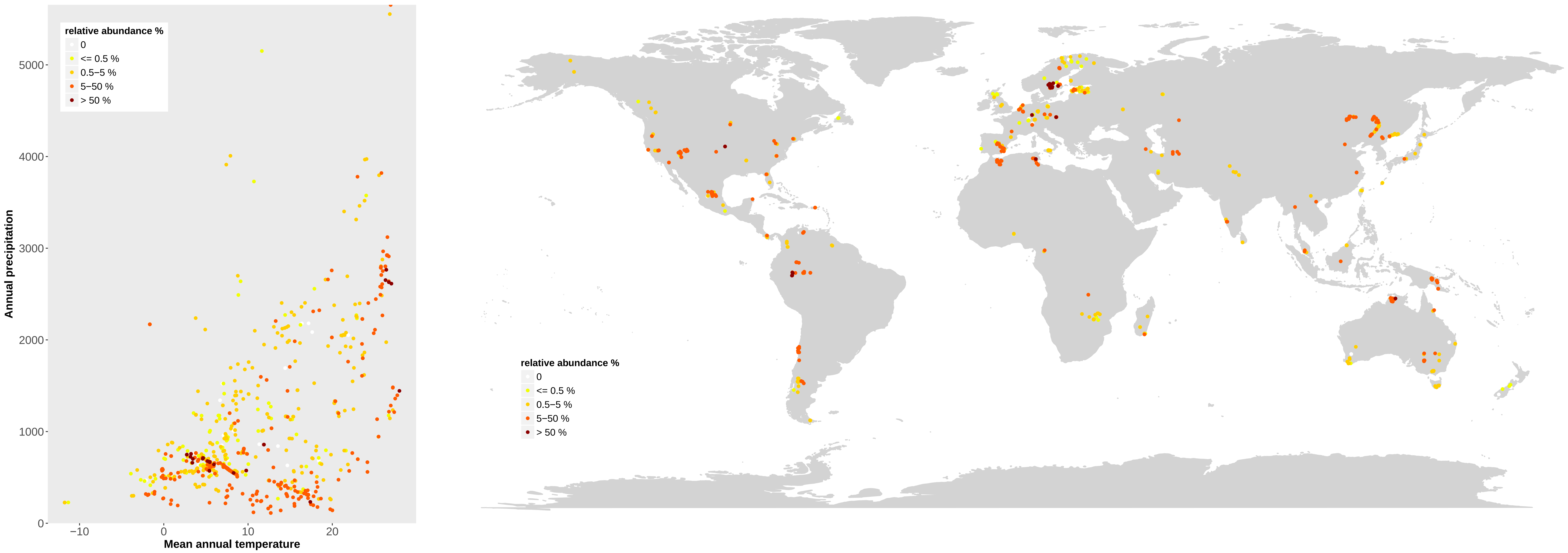

Saprotroph

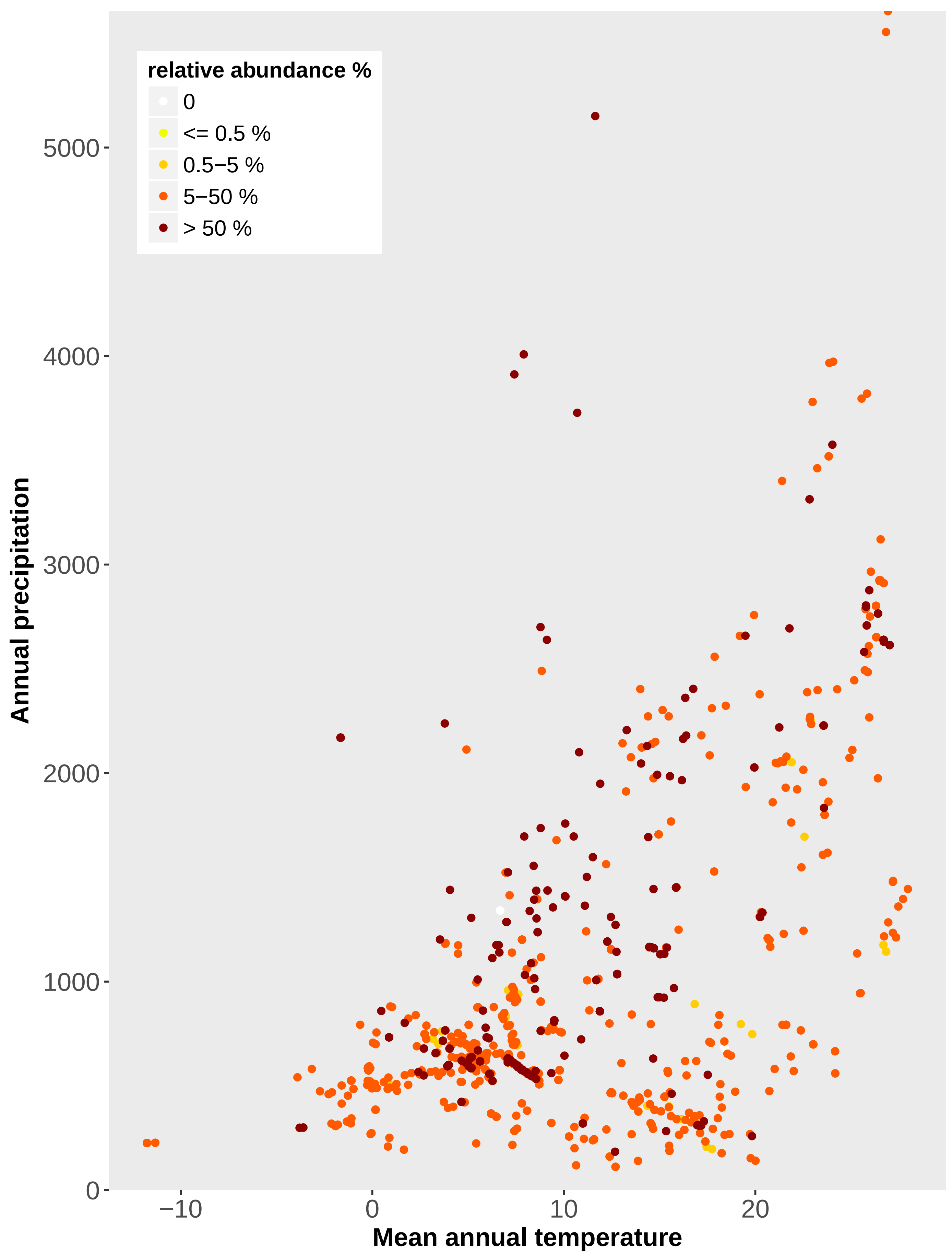
relative abundance %
0
<= 0.5 %
0.5−5 %
5−50 %
> 50 %
Annual precipitation
Mean annual temperature
0
1000
2000
3000
4000
5000
−10
0
10
20

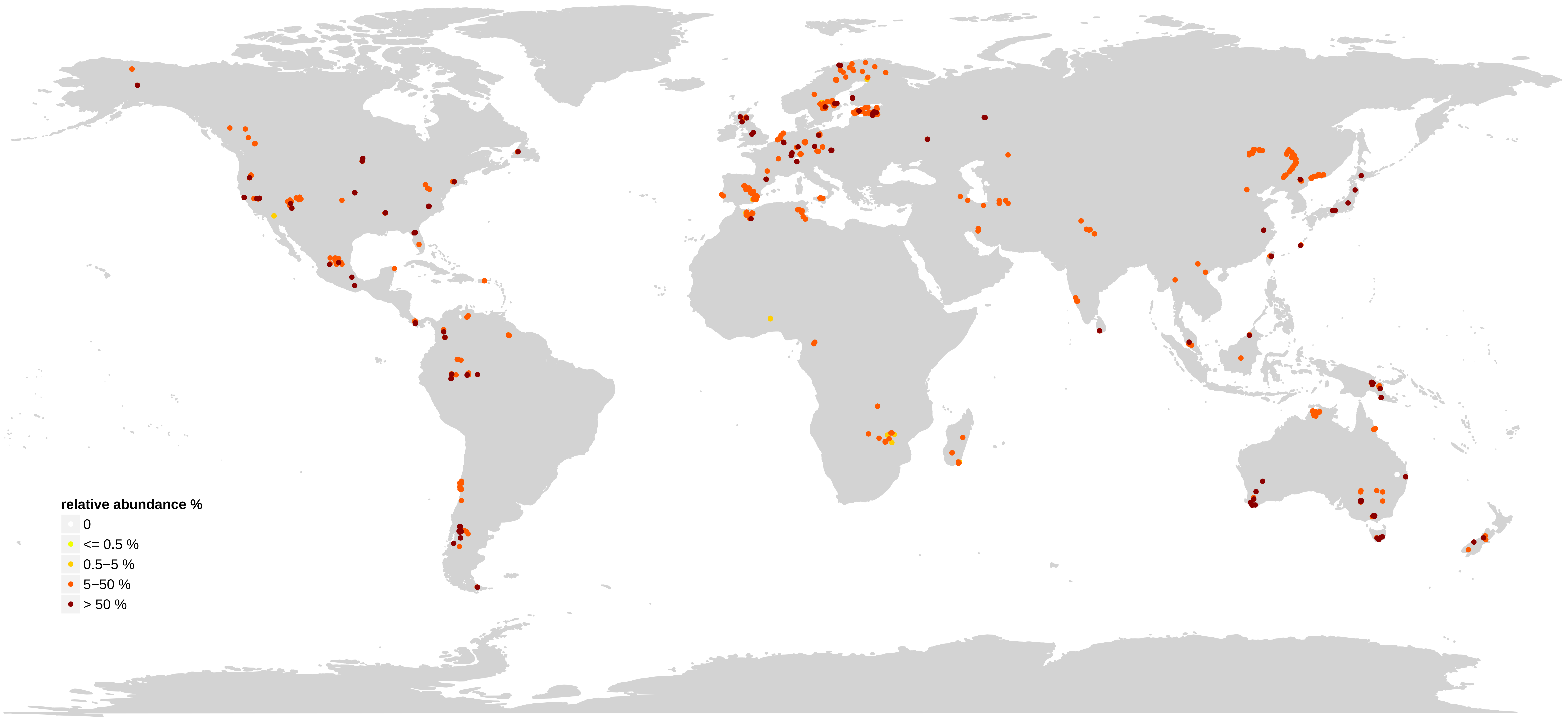
relative abundance %
0
<= 0.5 %
0.5−5 %
5−50 %
> 50 %

Wood Saprotroph

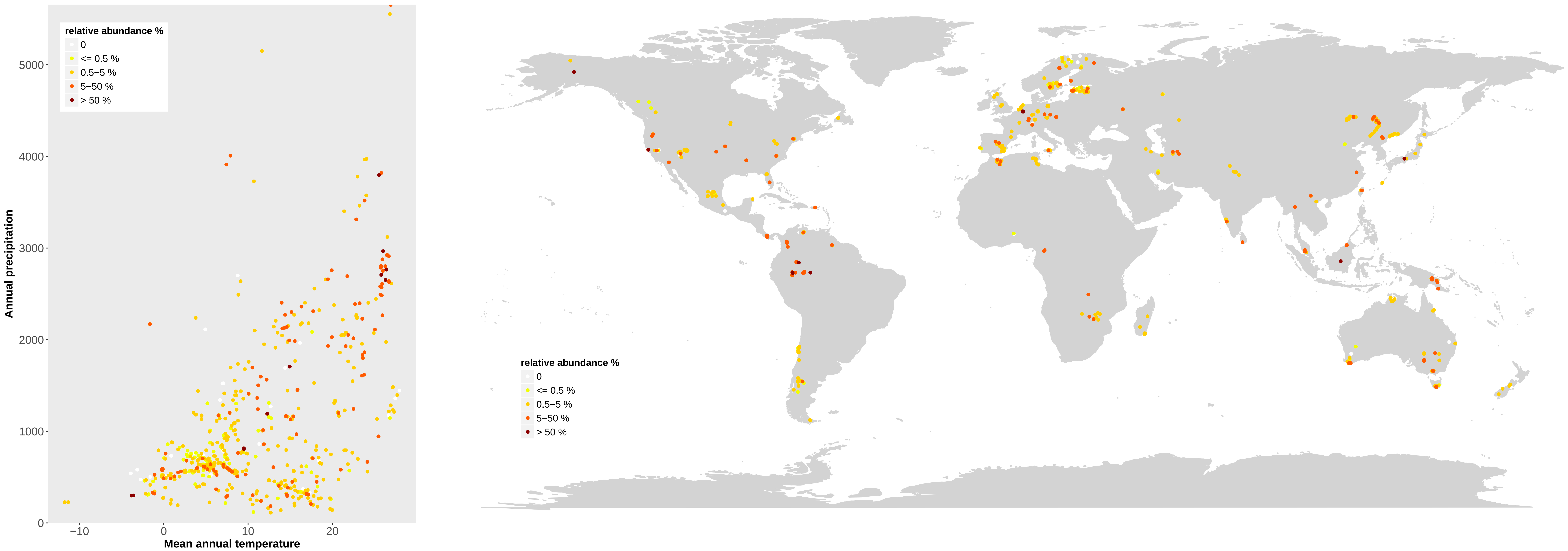

Supplementary Figure 3: Relative abundances of the sequences of ecological guilds of fungi in samples across the range of mean annual temperatures/annual precipitation and geographic distribution of samples.

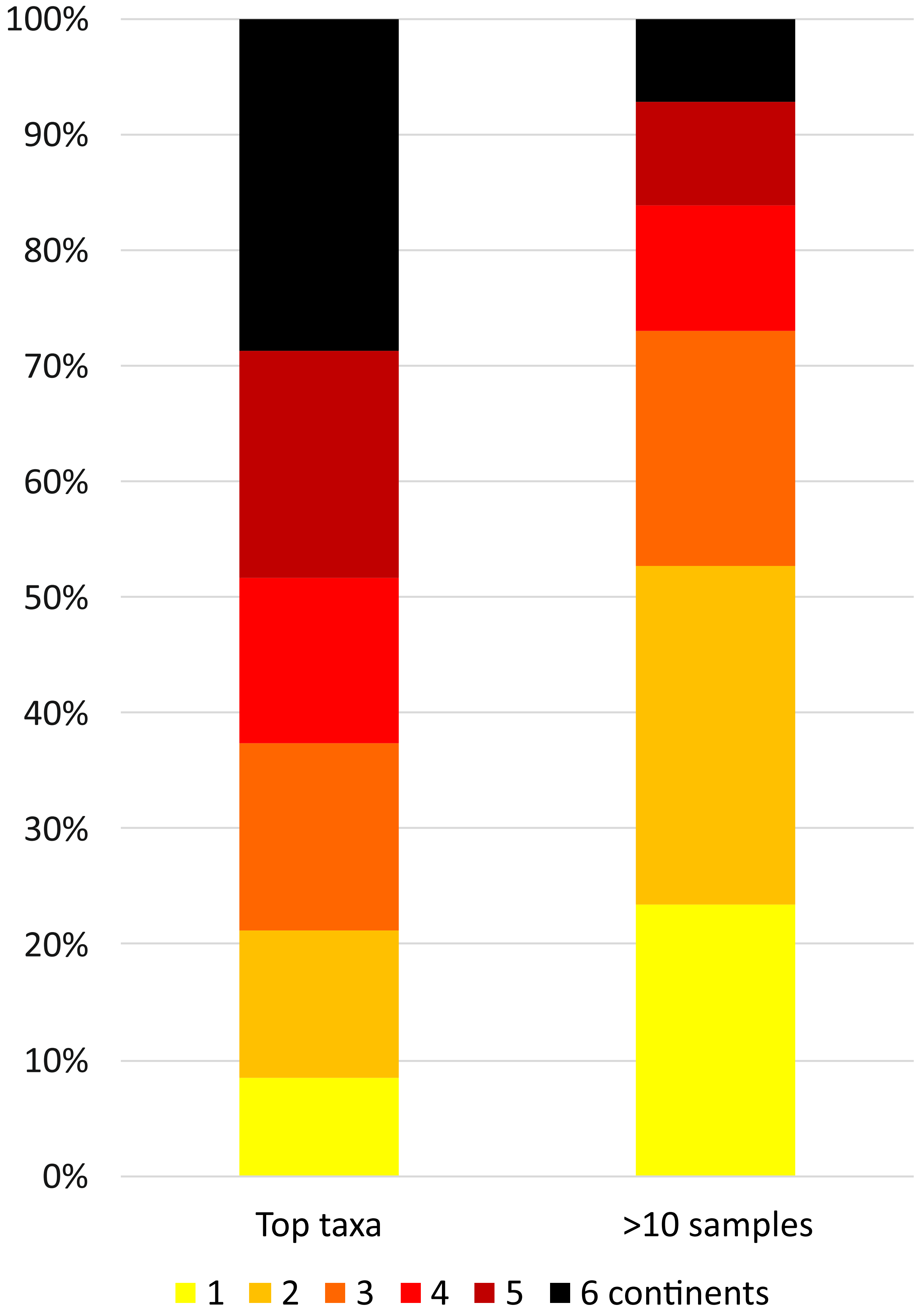

100%
90%
80%
70%
60%
50%
40%
30%
20%
10%
0%
Top taxa
>10 samples
1
2
3
4
5
6 continents

Supplementary Figure 4: Occurrence of the most common fungal species hypotheses (SHs) and all fungal taxa present in 10 or more samples across continents.

SH216786 Pleosporaceae sp

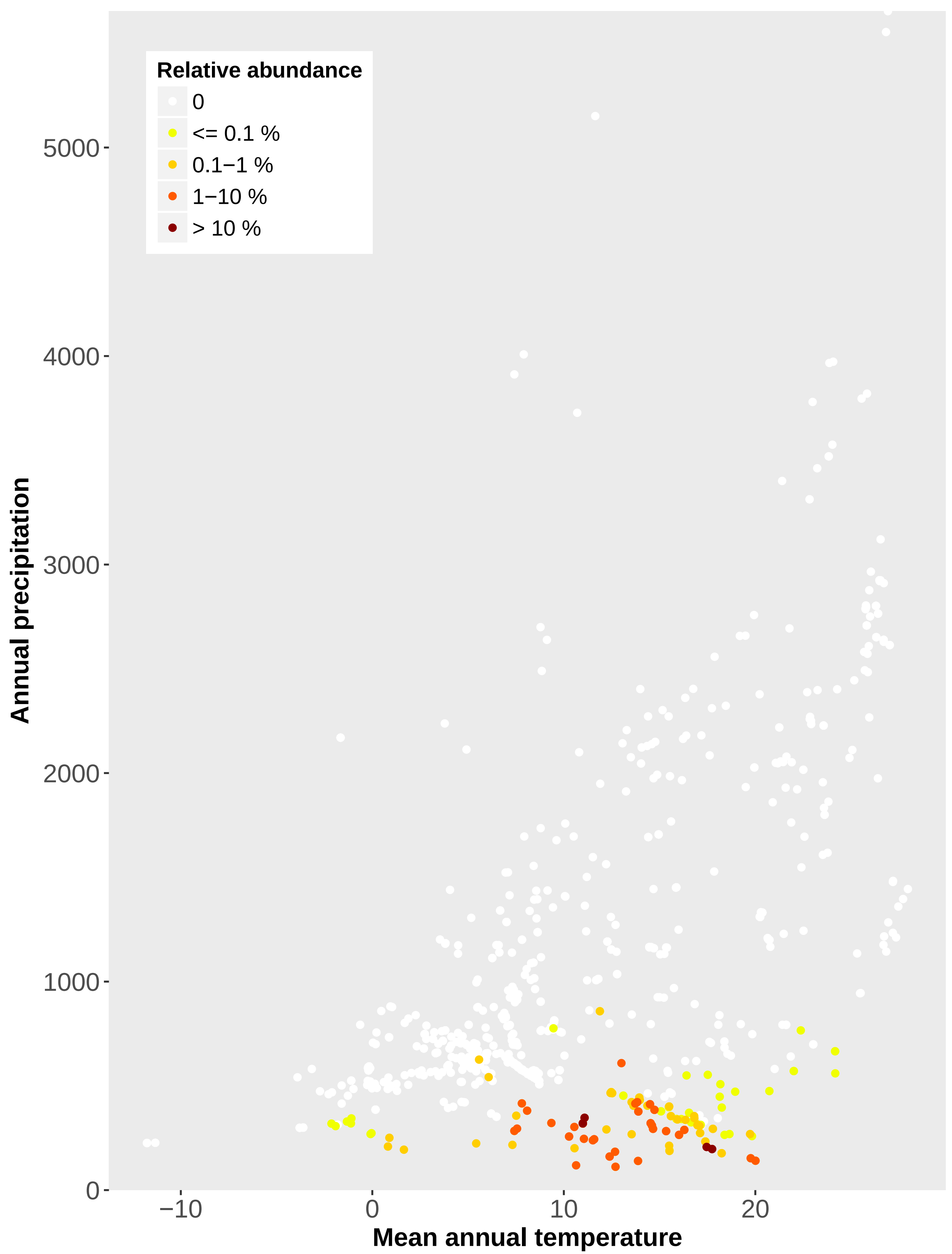

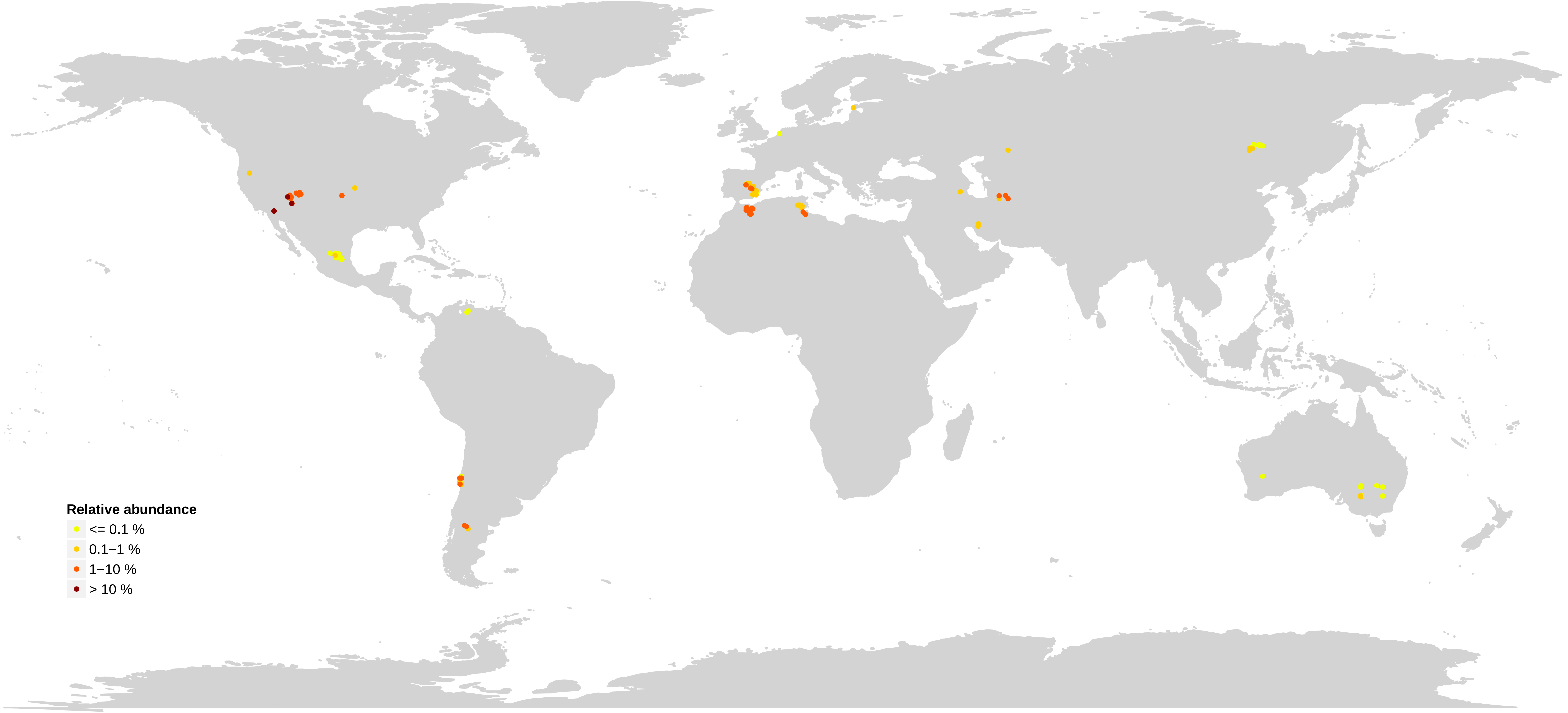

SH196779 Mortierella humilis

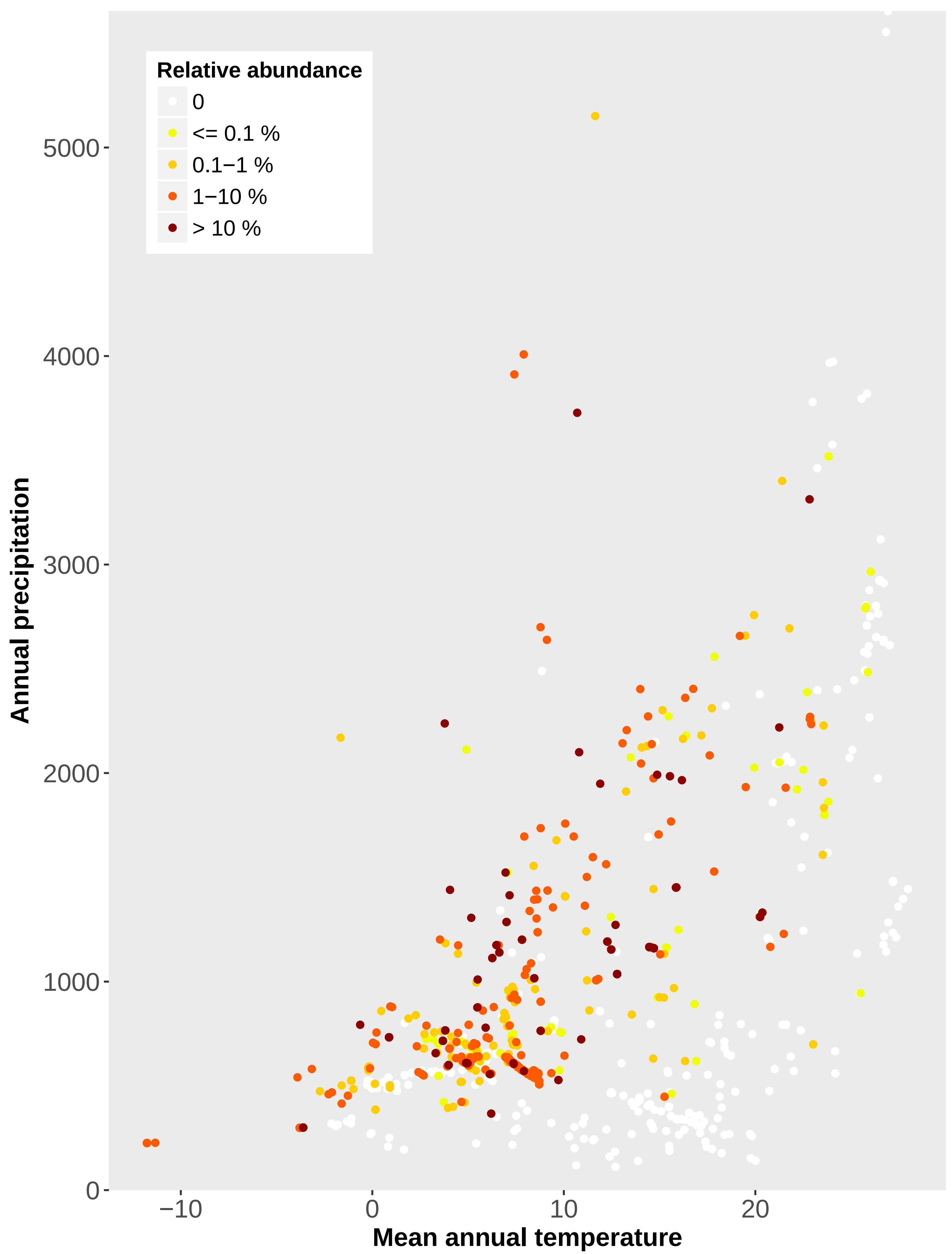

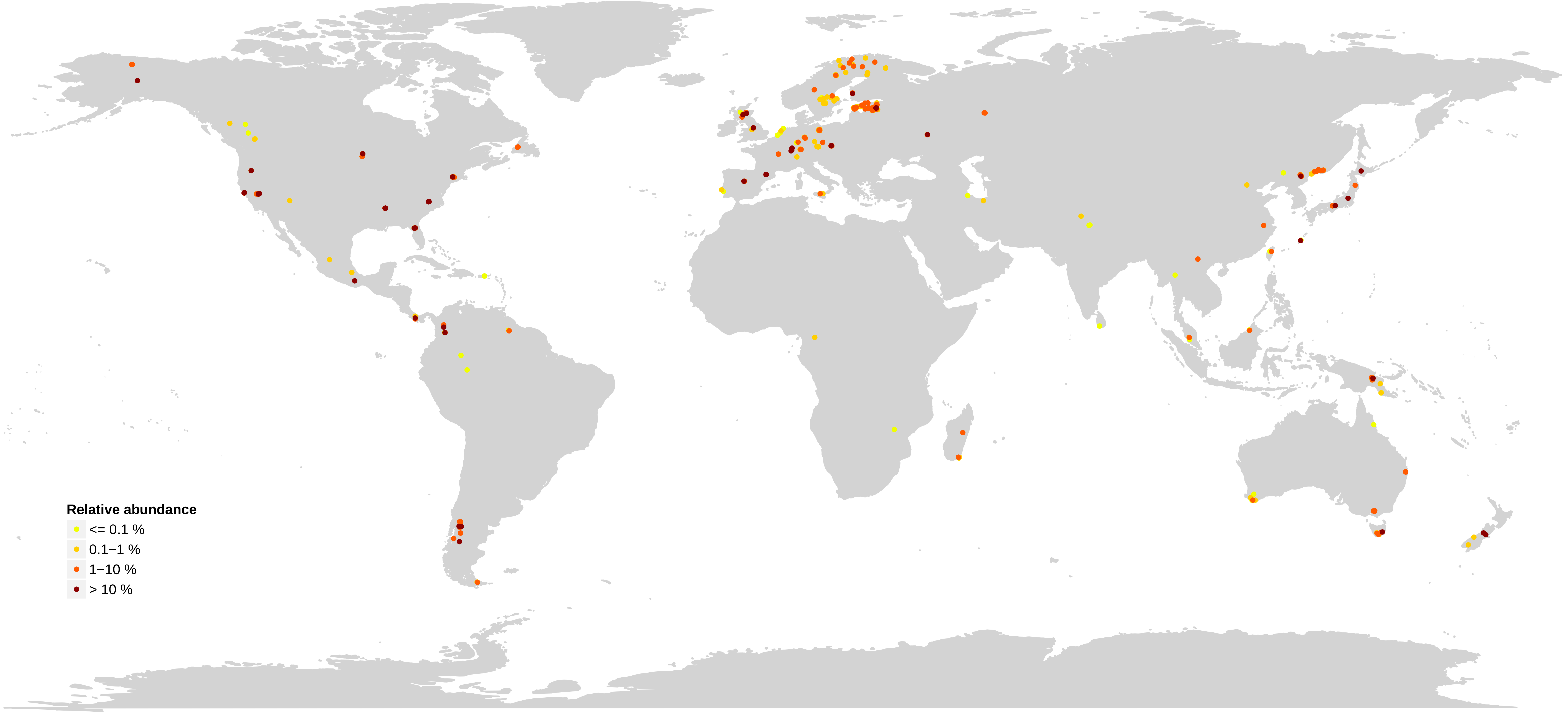

SH216785 Ulocladium chartarum

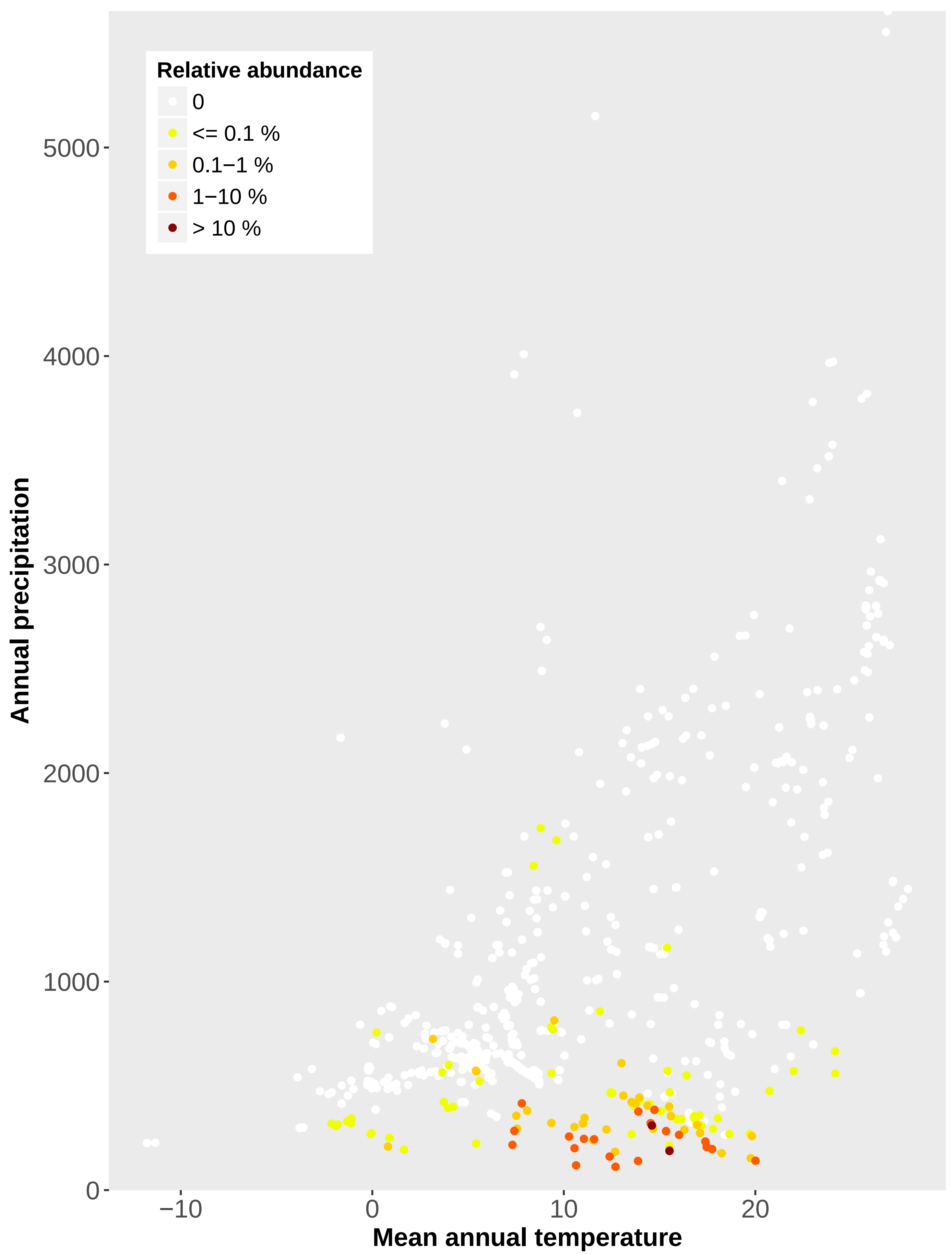

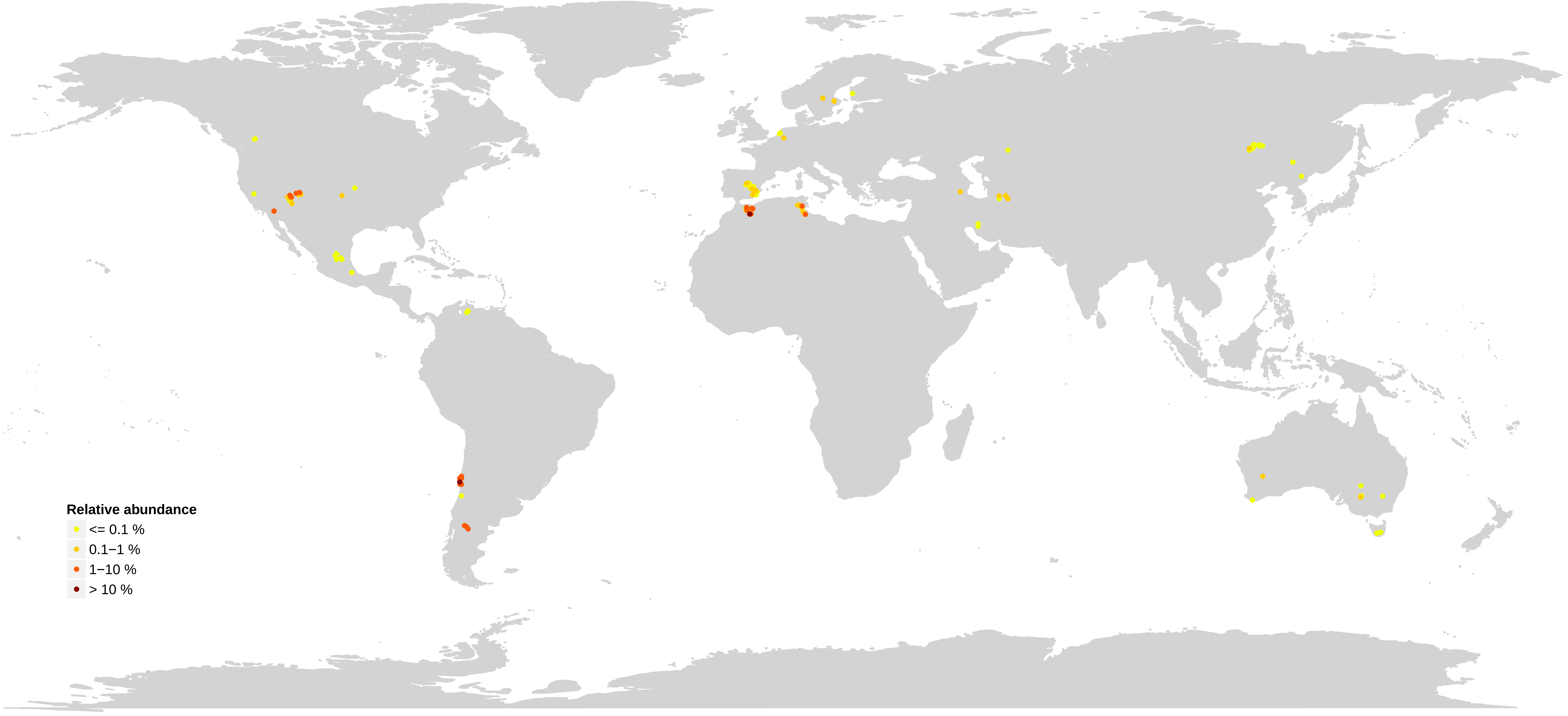

SH216990 Oidiodendron chlamydosporicum

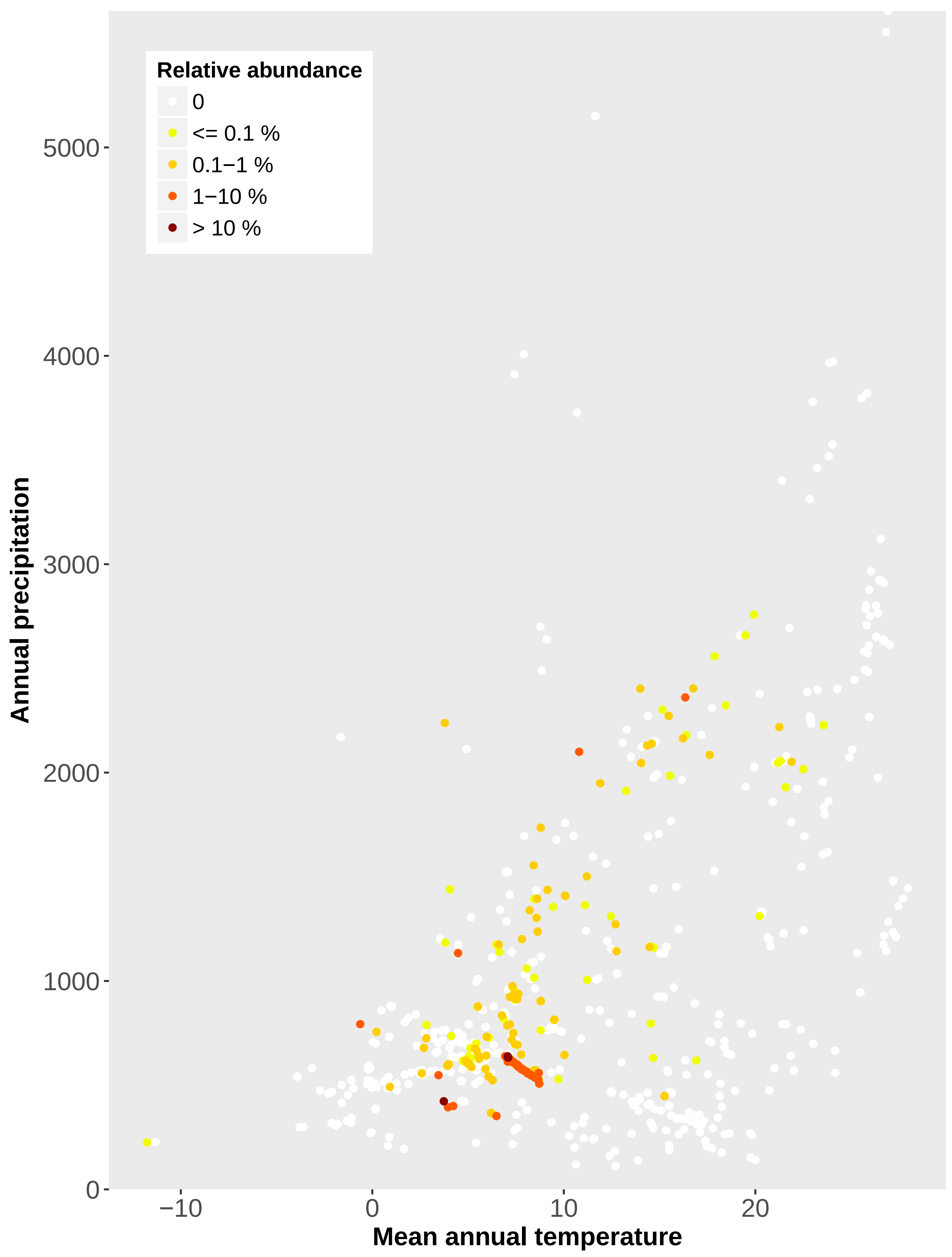

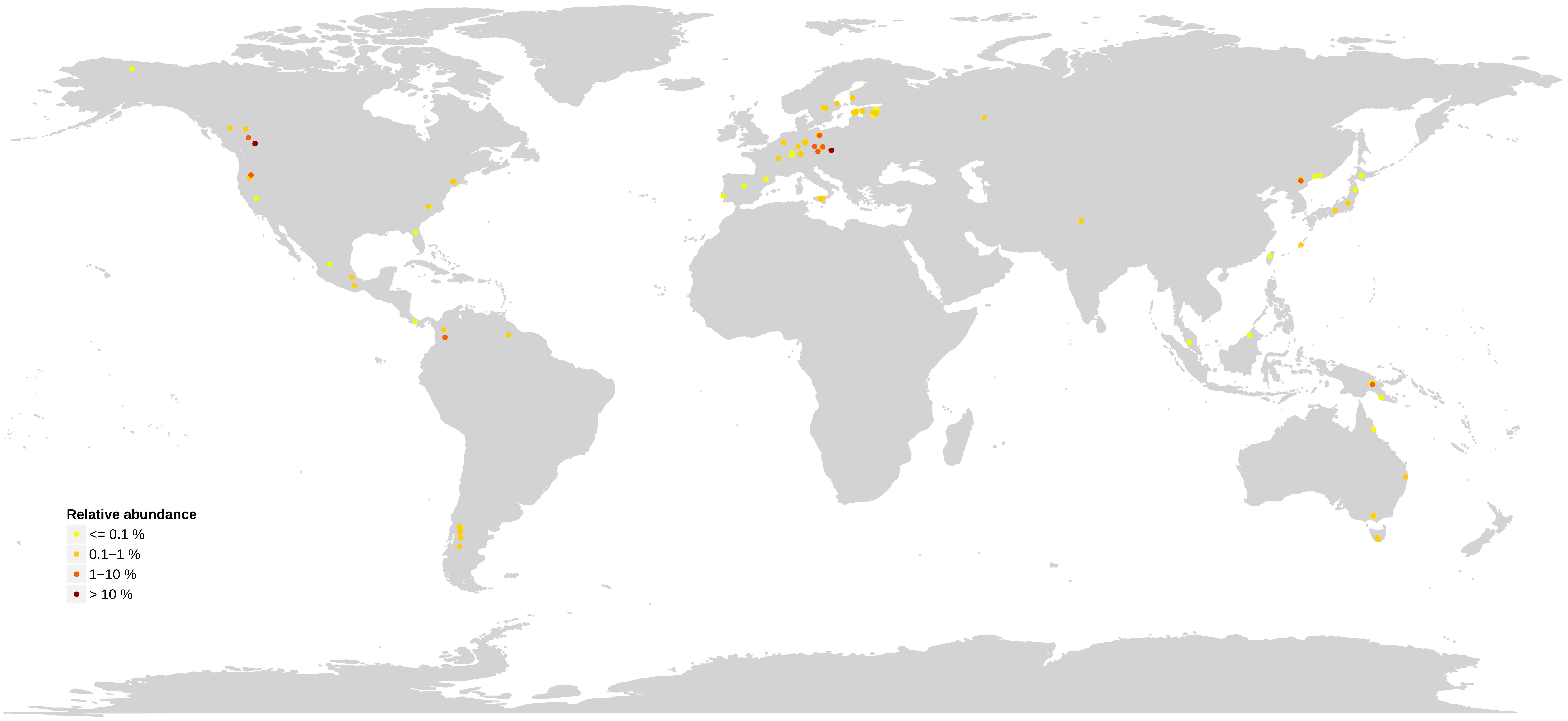

SH220702 Nectriaceae sp

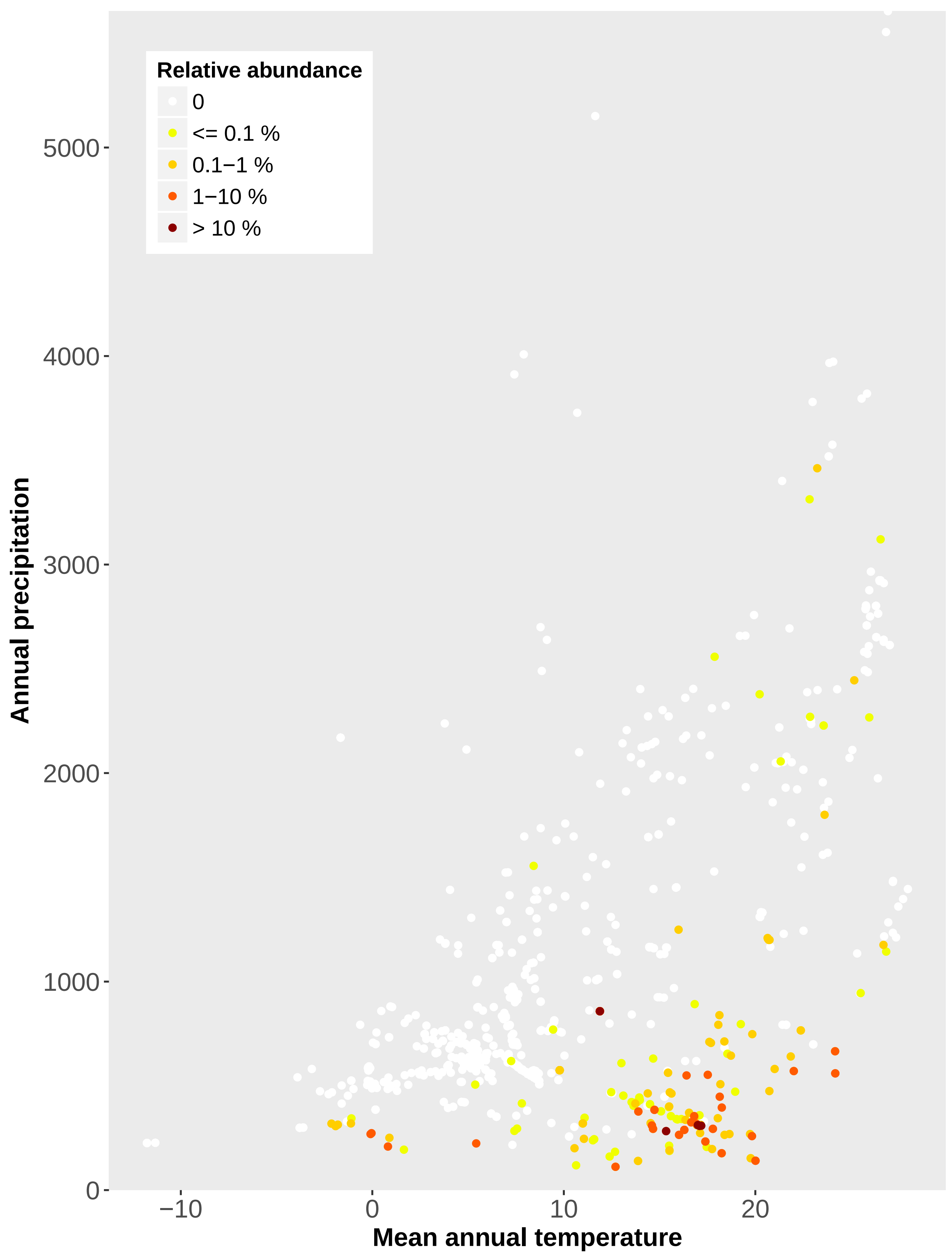

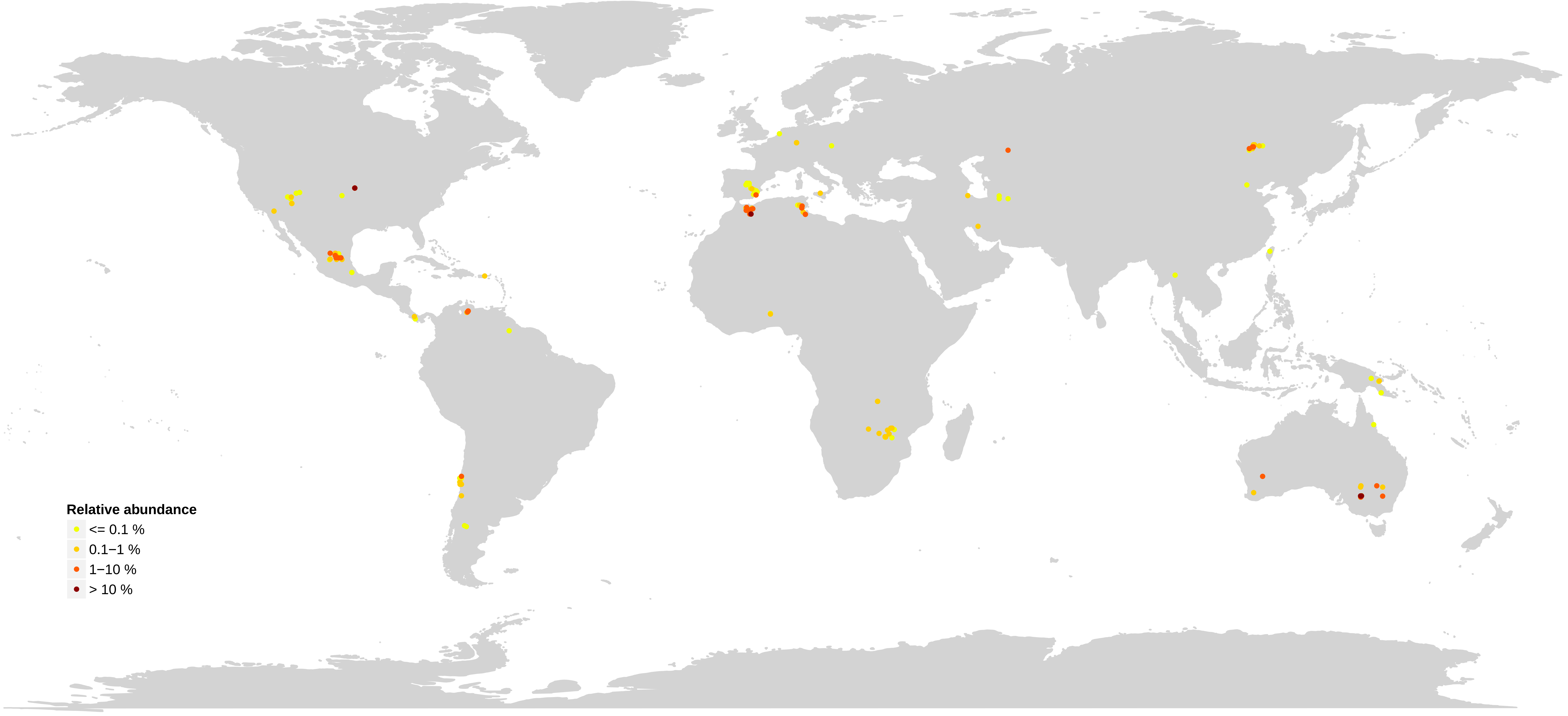

SH196824 Piloderma sphaerosporum

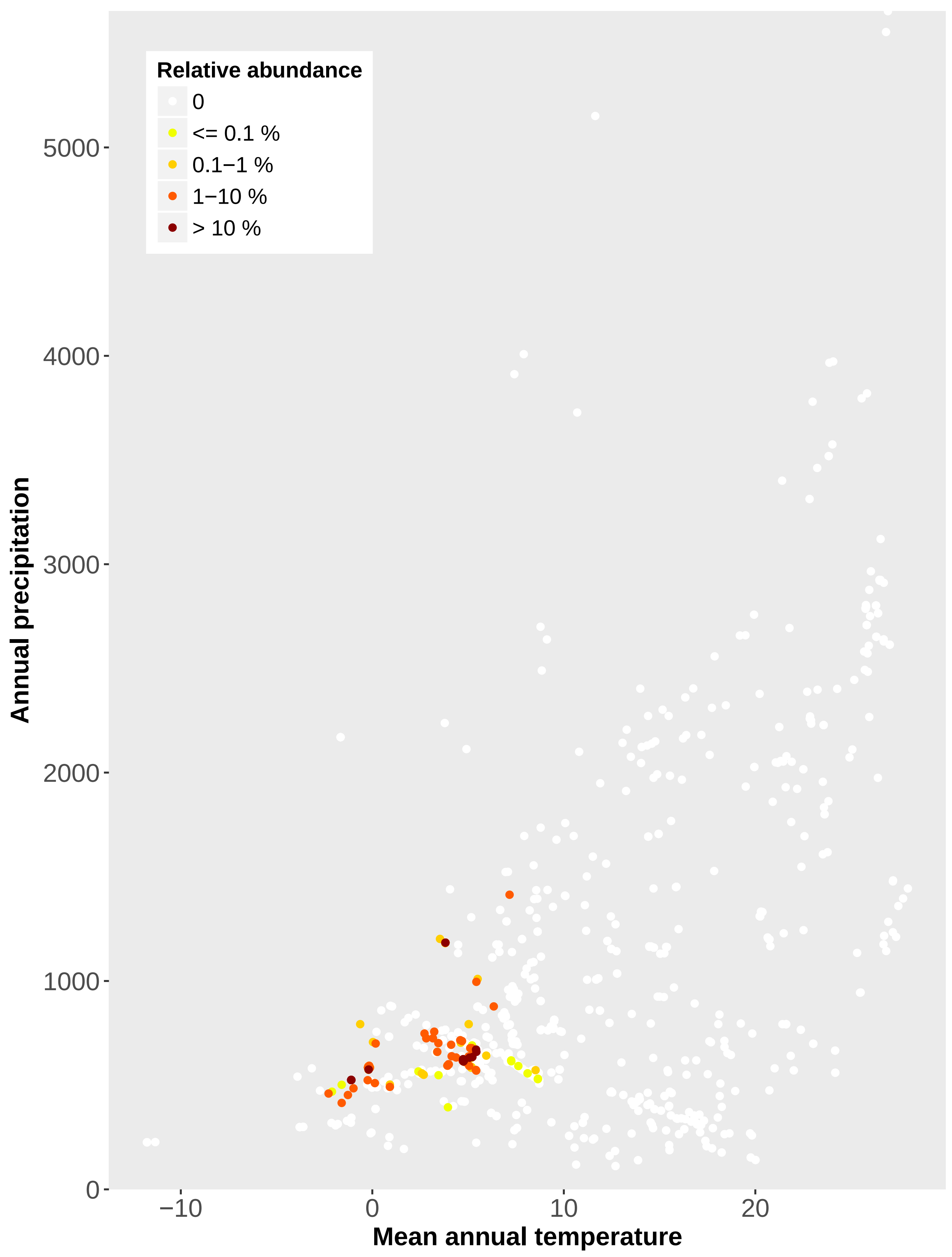

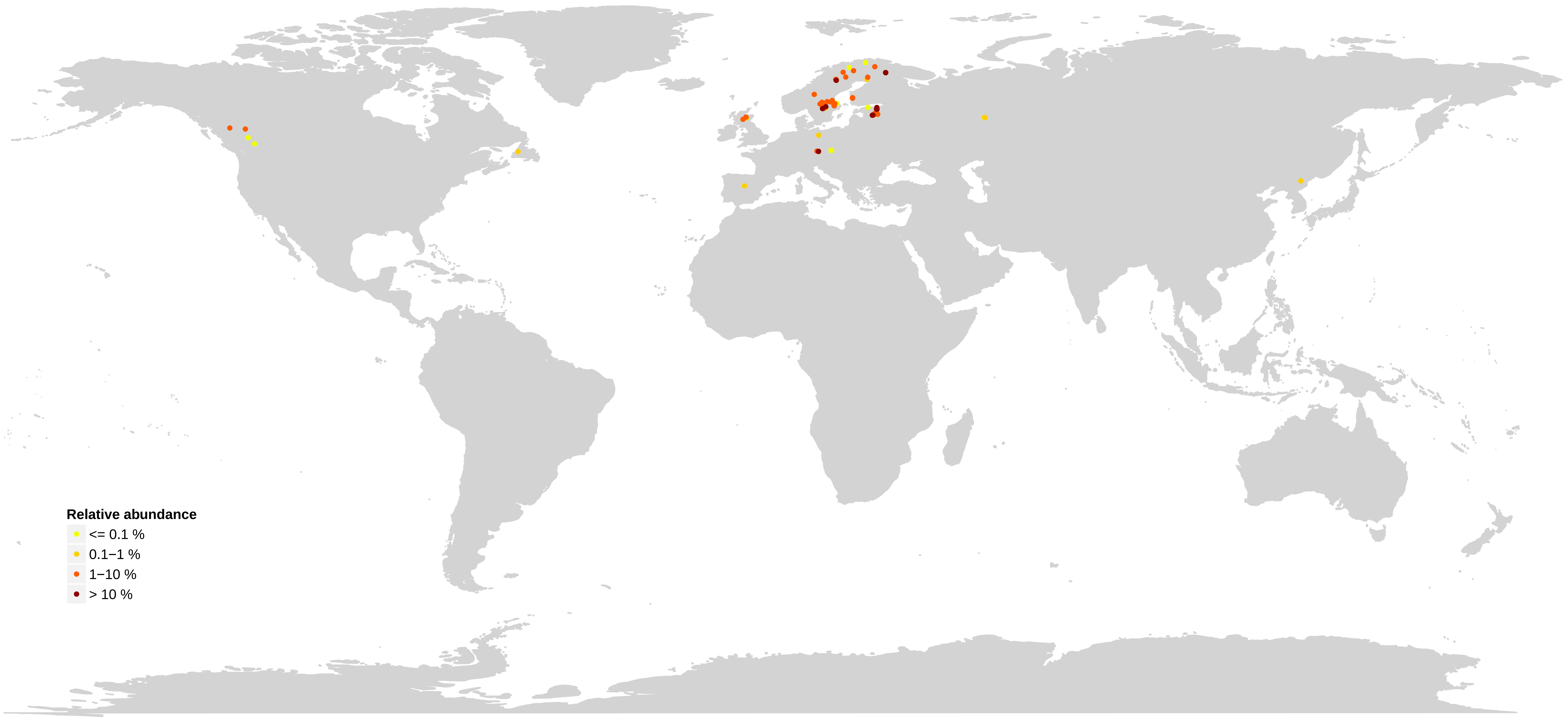

SH190017 Cryptococcus terricola

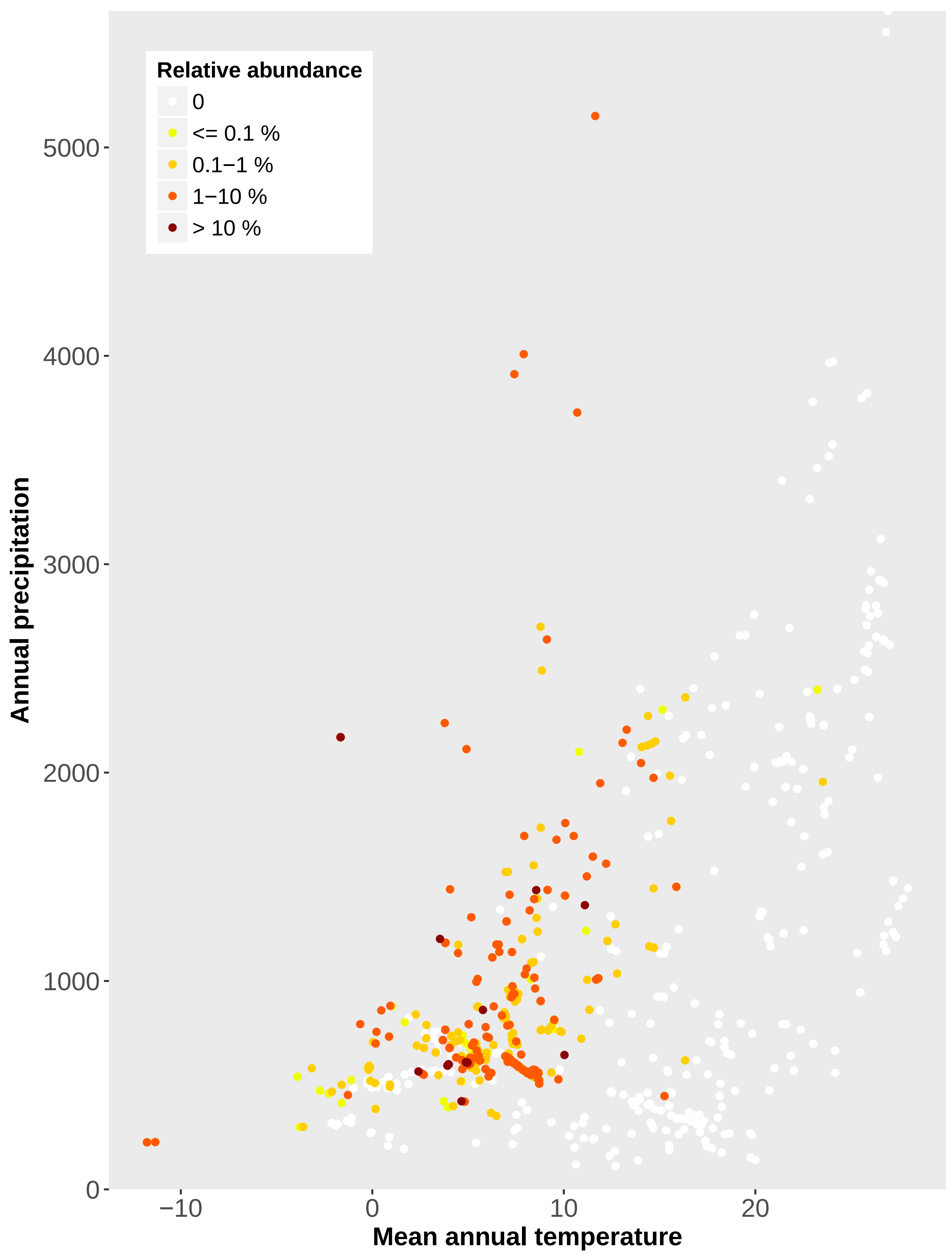

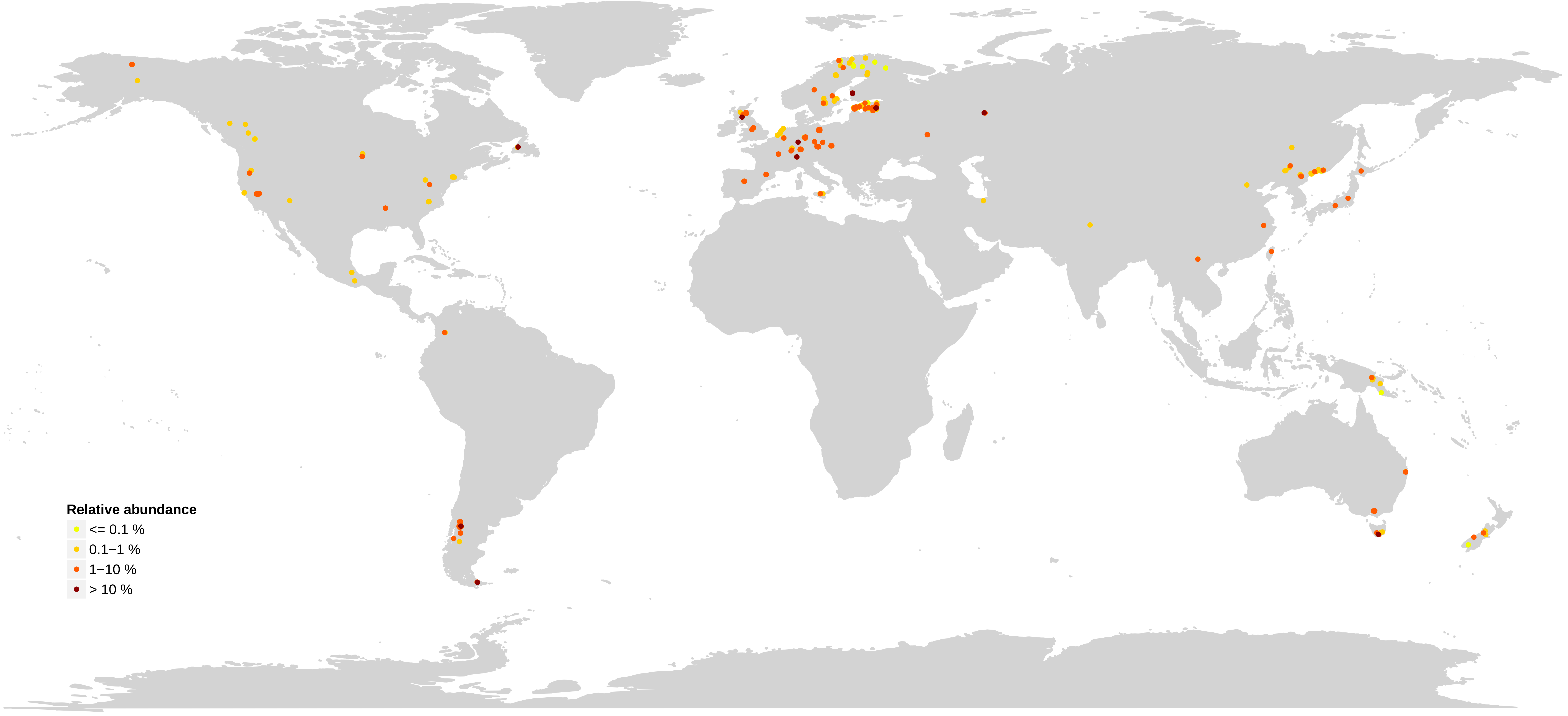

SH182152 Ascomycota sp

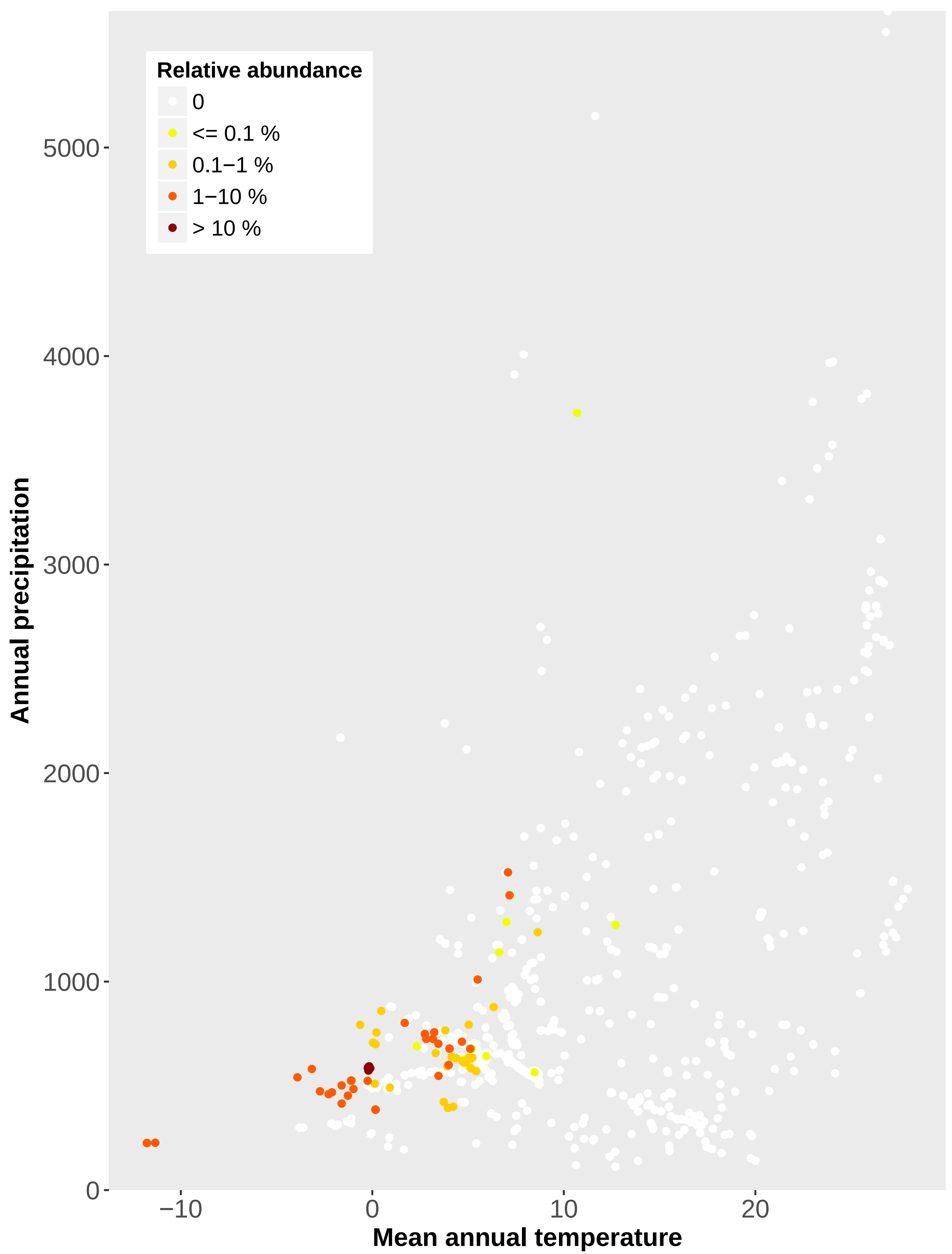

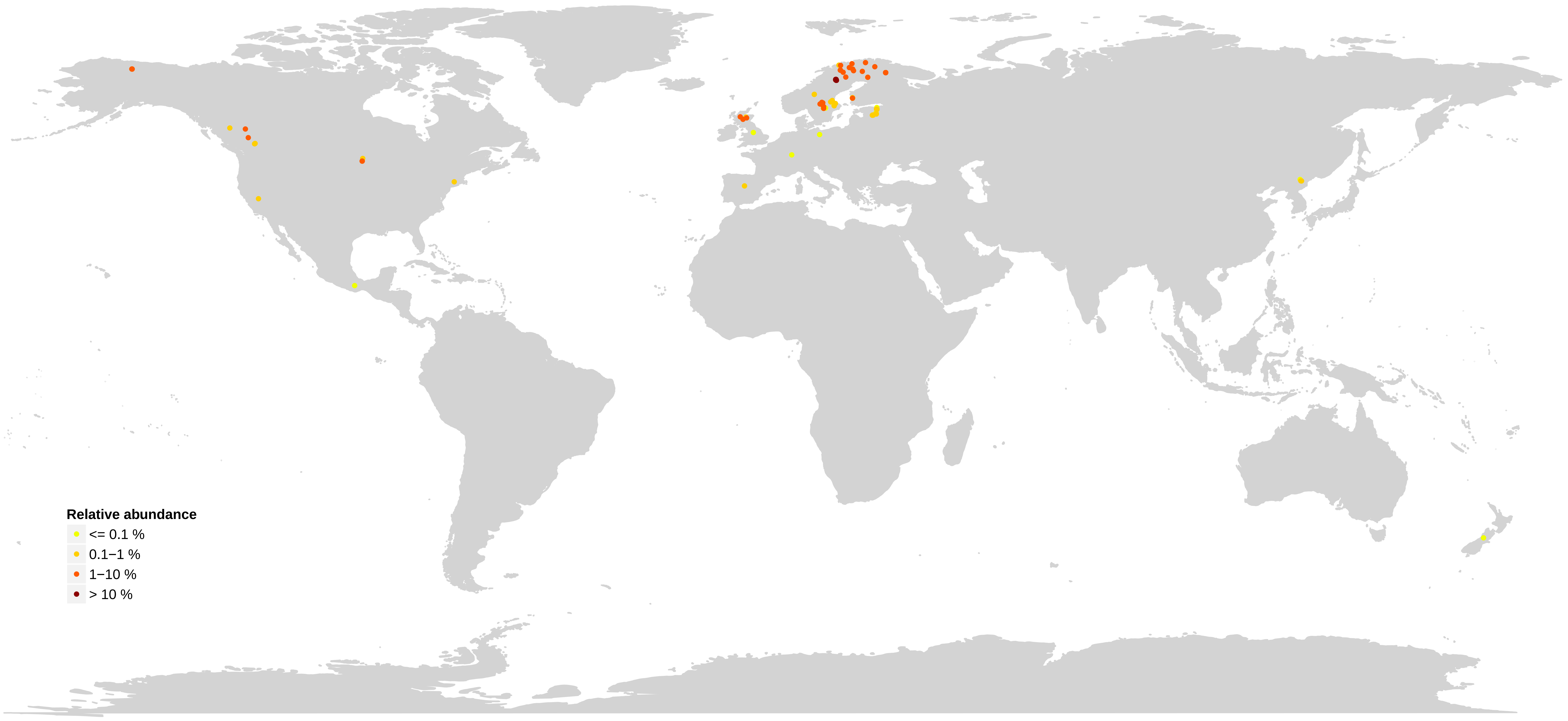

SH245191 Macroventuria anomochaeta

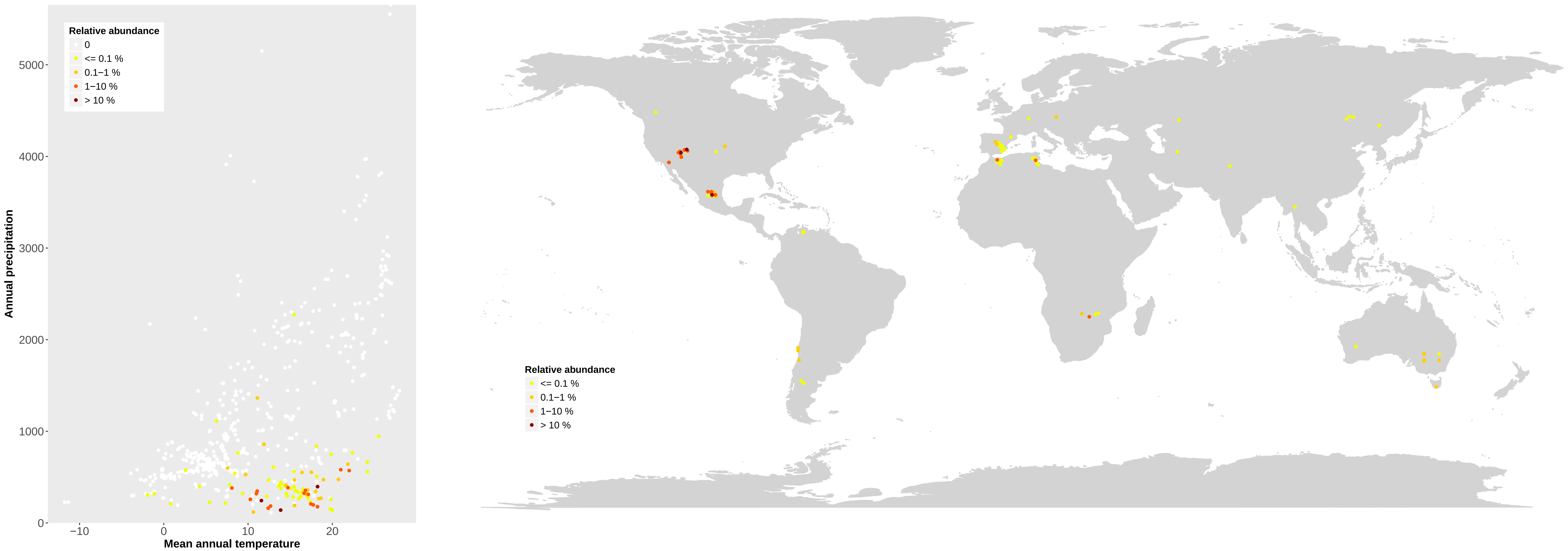

SH214266 Pezoloma ericae

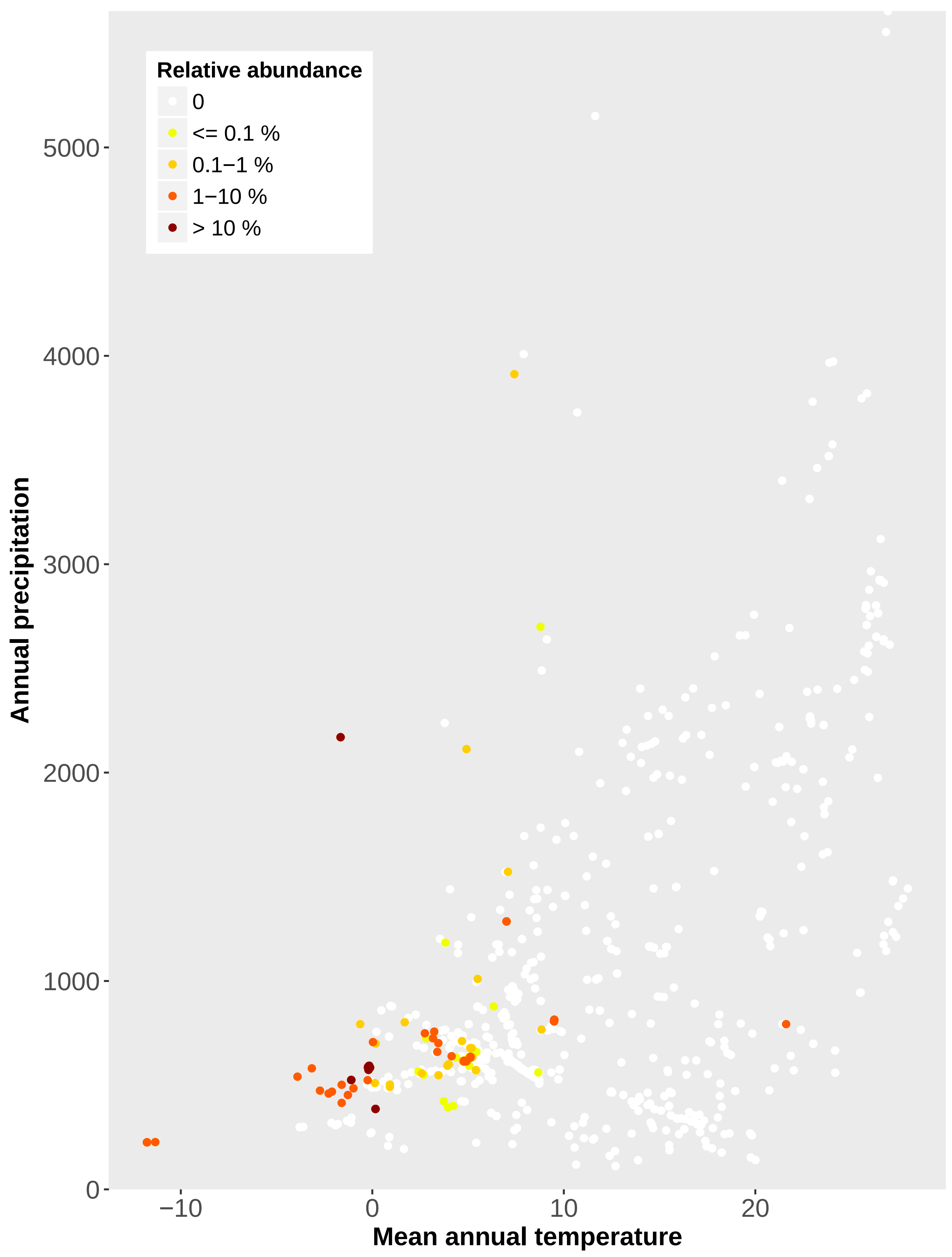

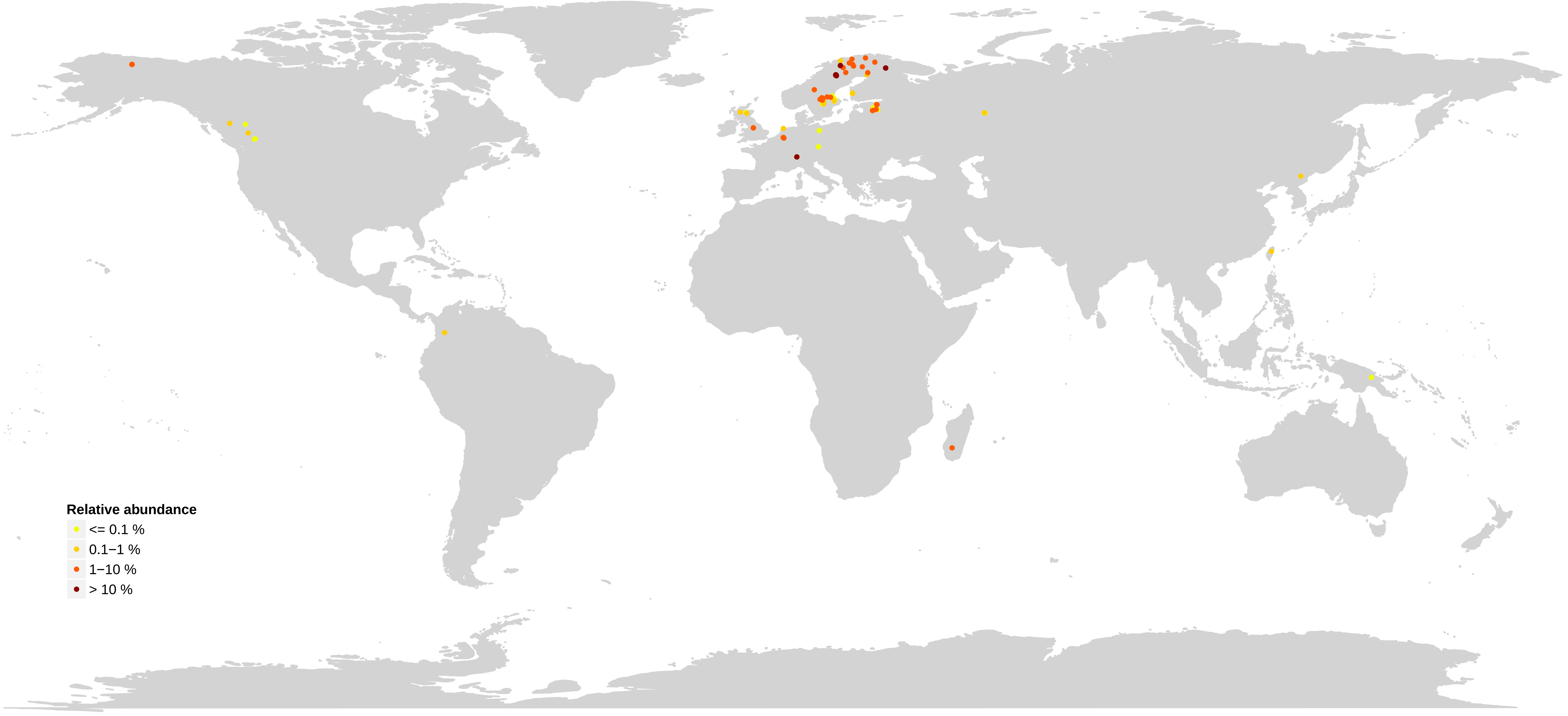

SH213880 Gibberella tricincta

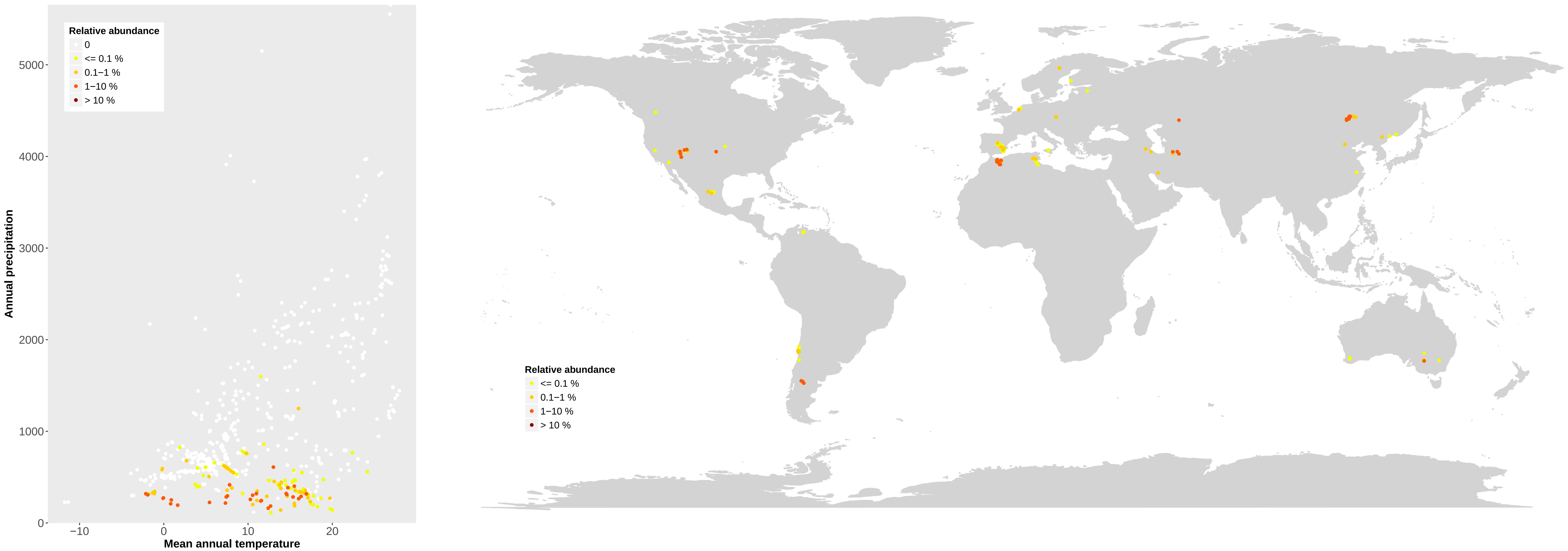

SH182978 Ascomycota sp

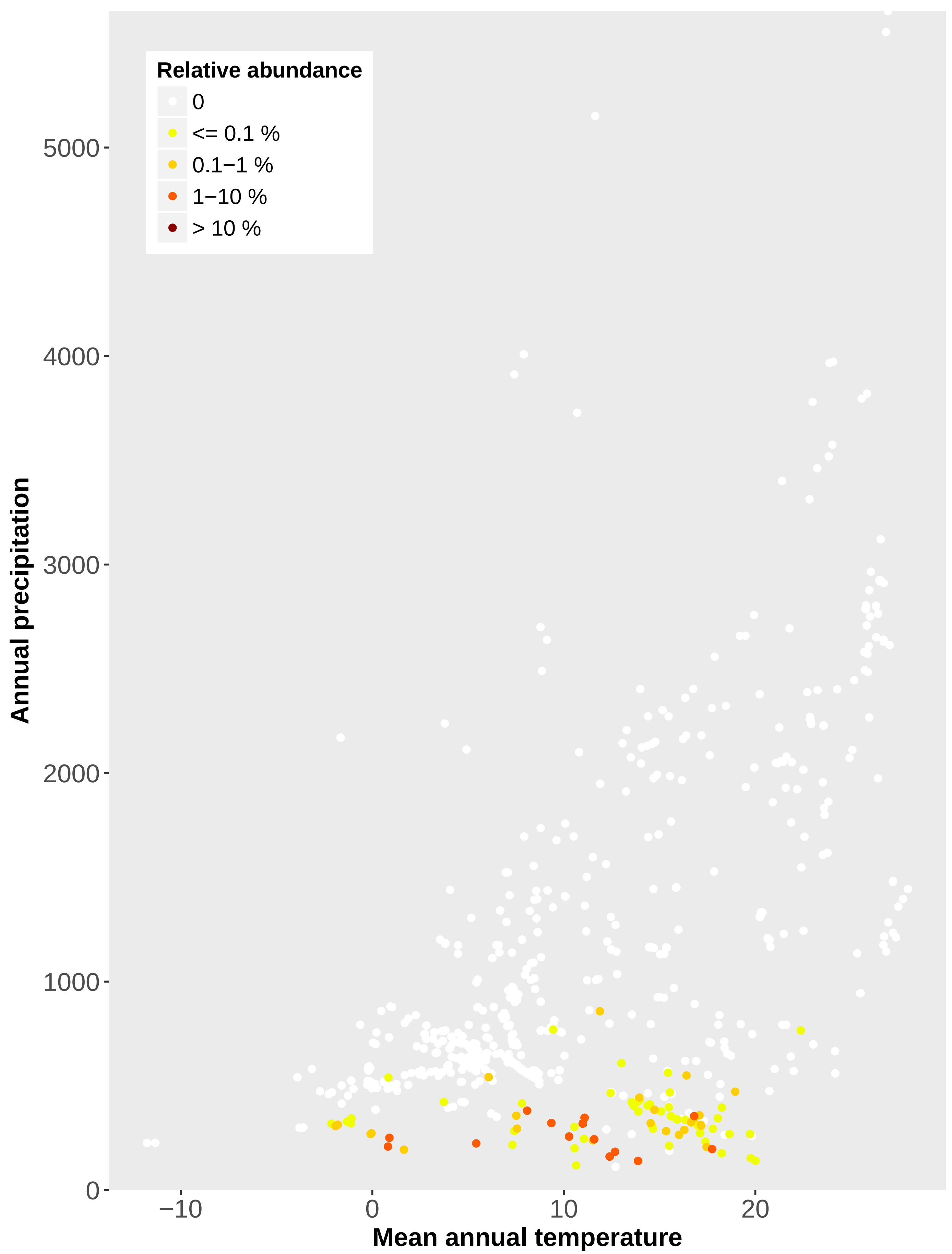

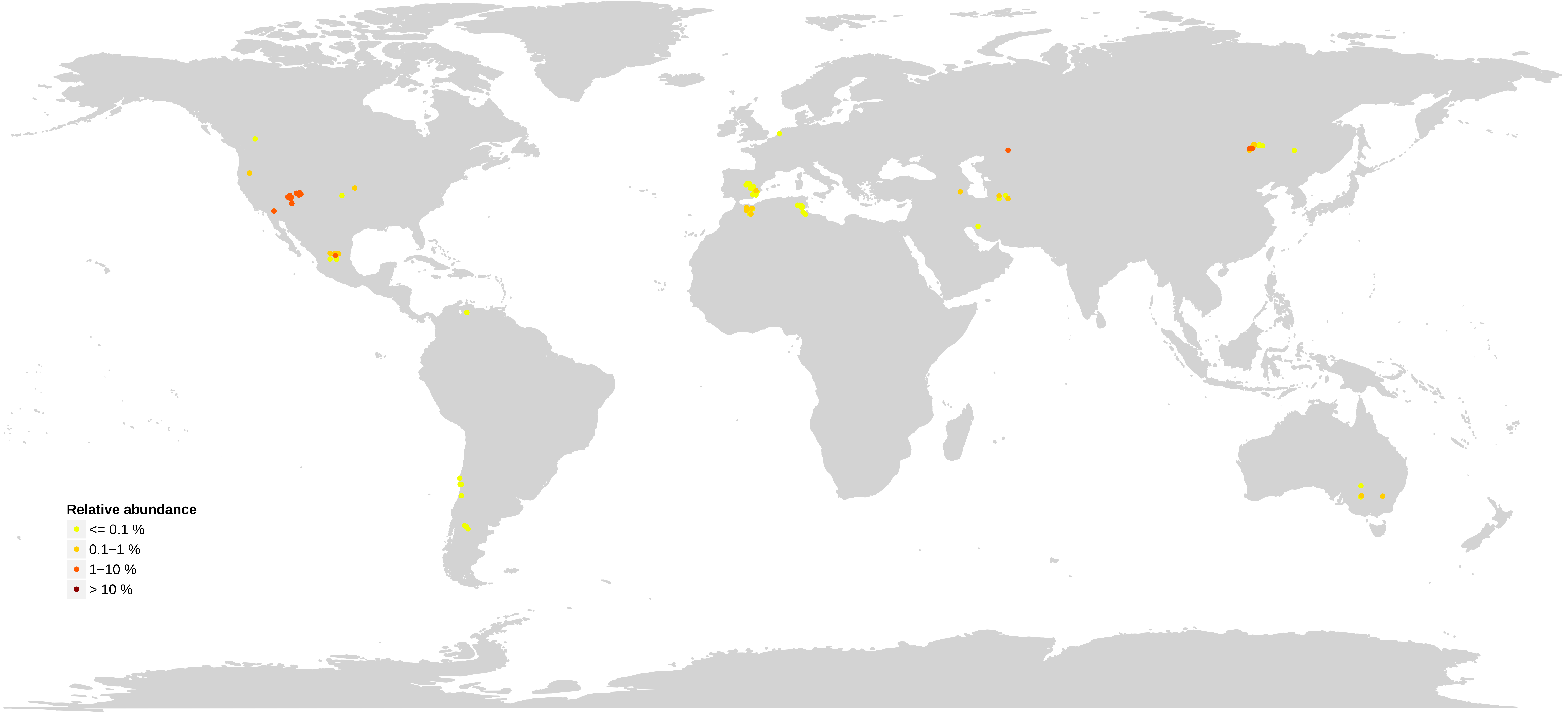

SH422672 Cladosporium exasperatum

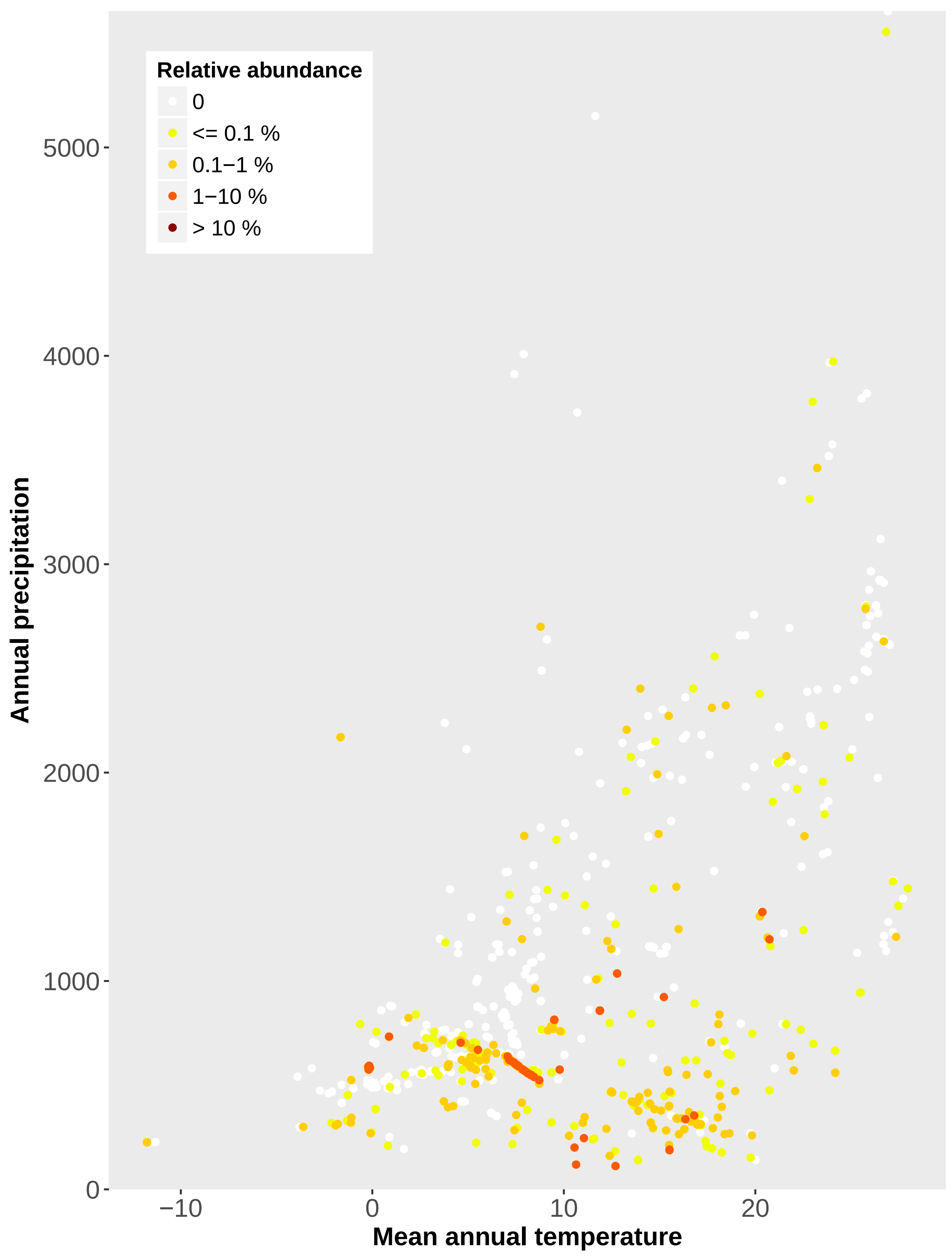

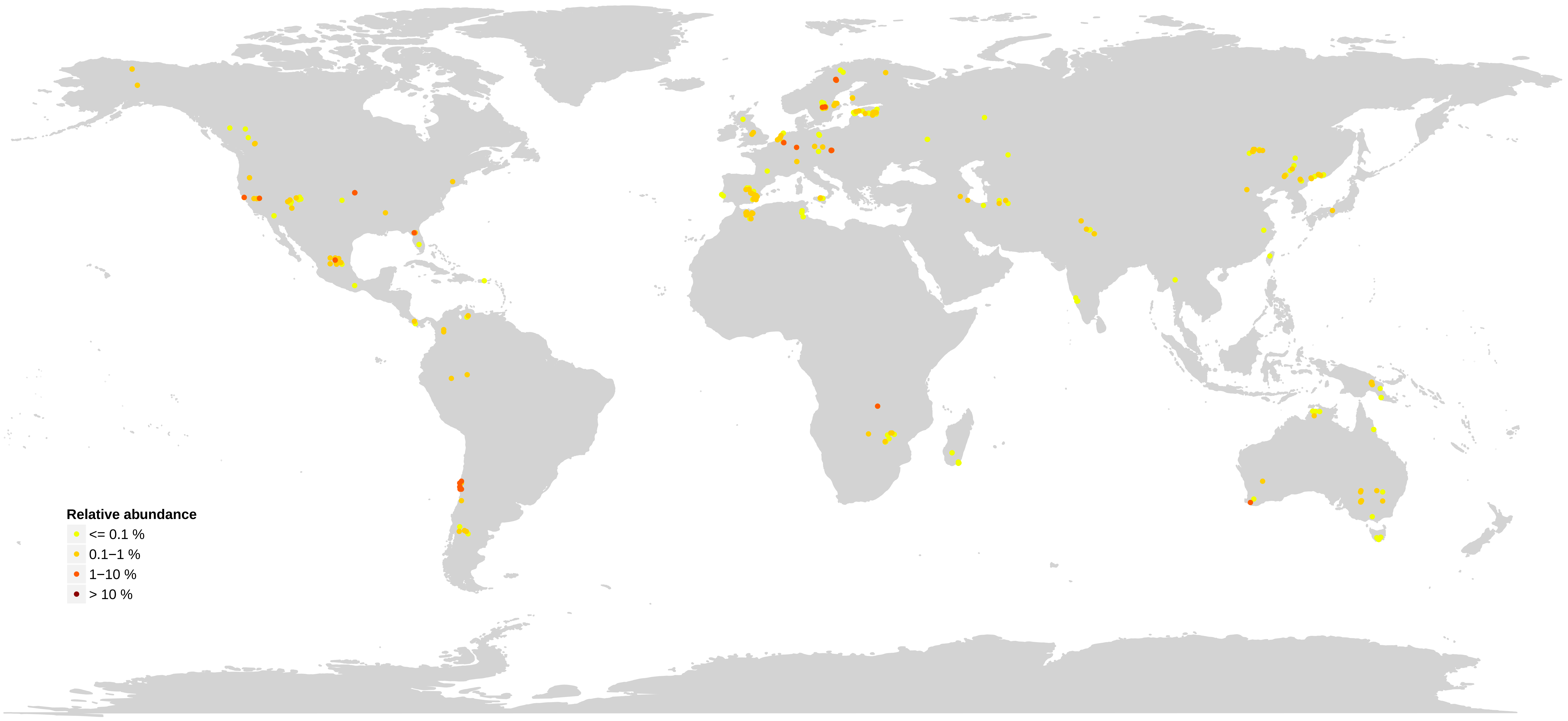

SH196089 Umbelopsis dimorpha

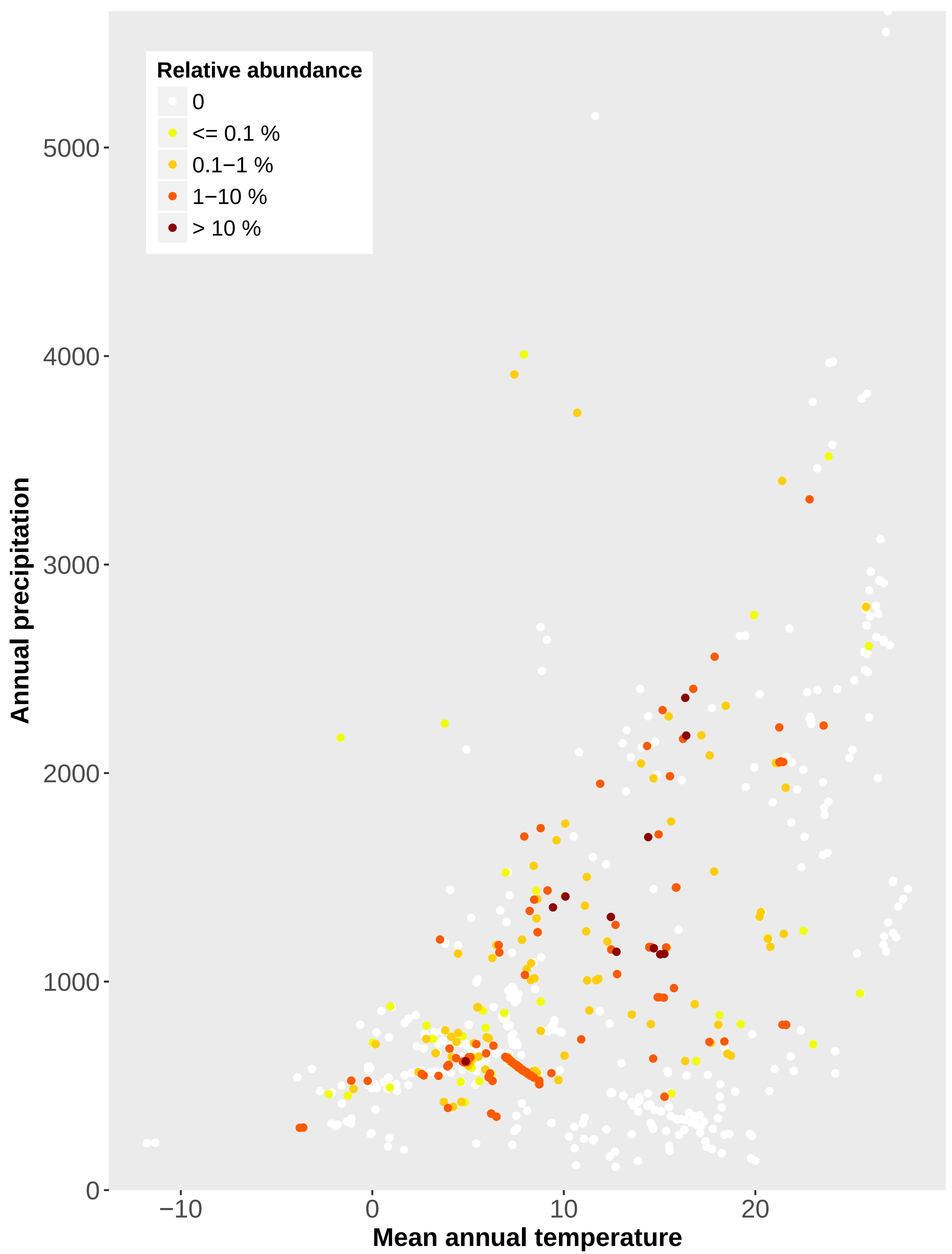

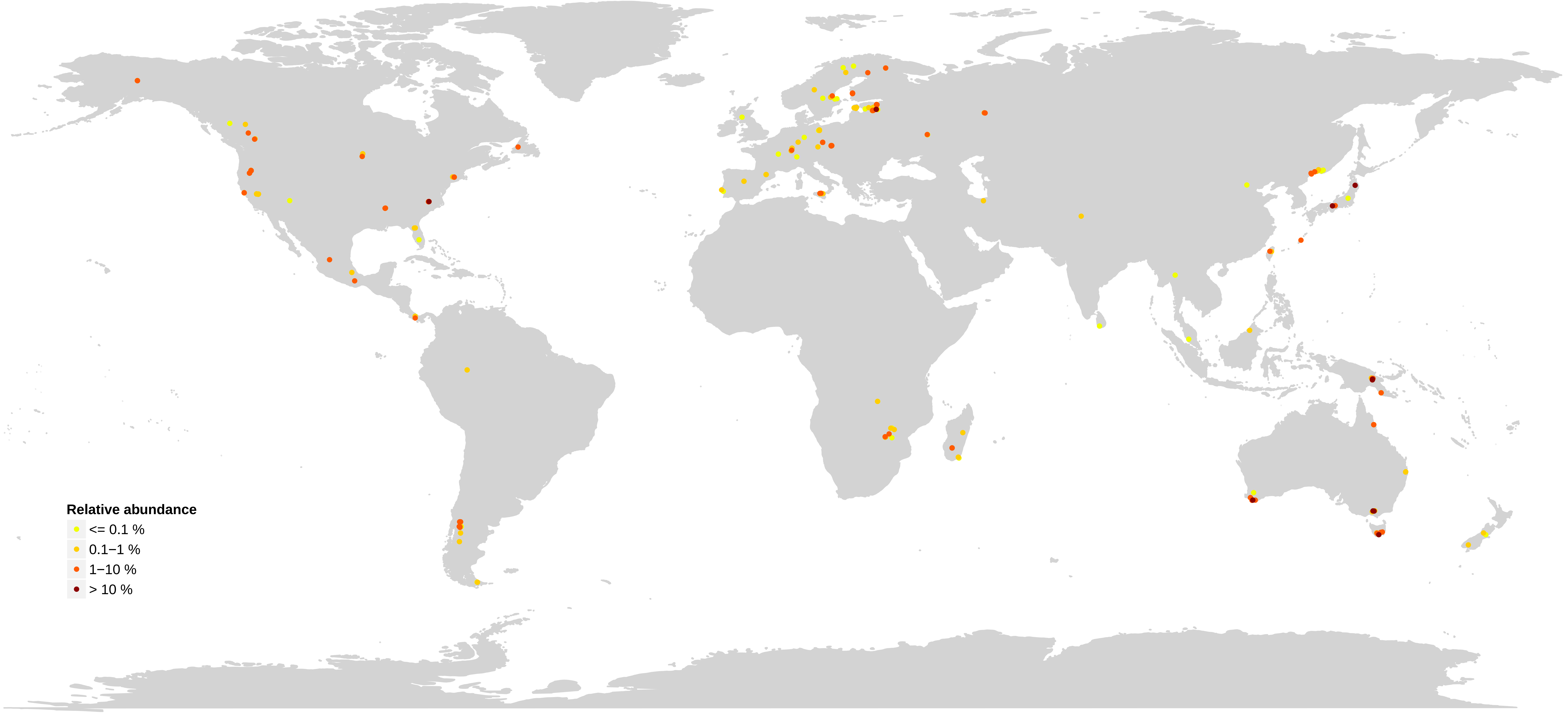

SH374010 Humicola grisea

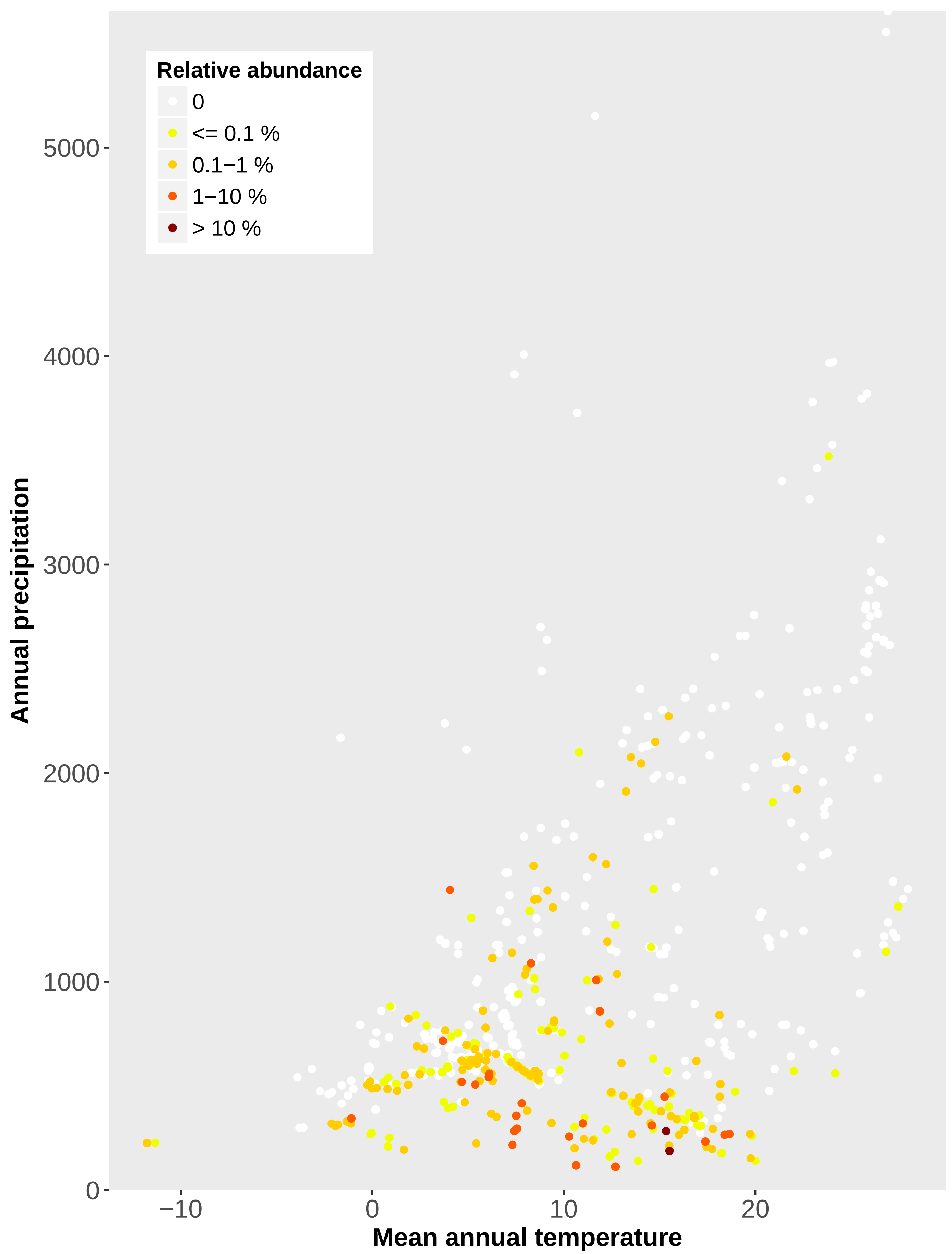

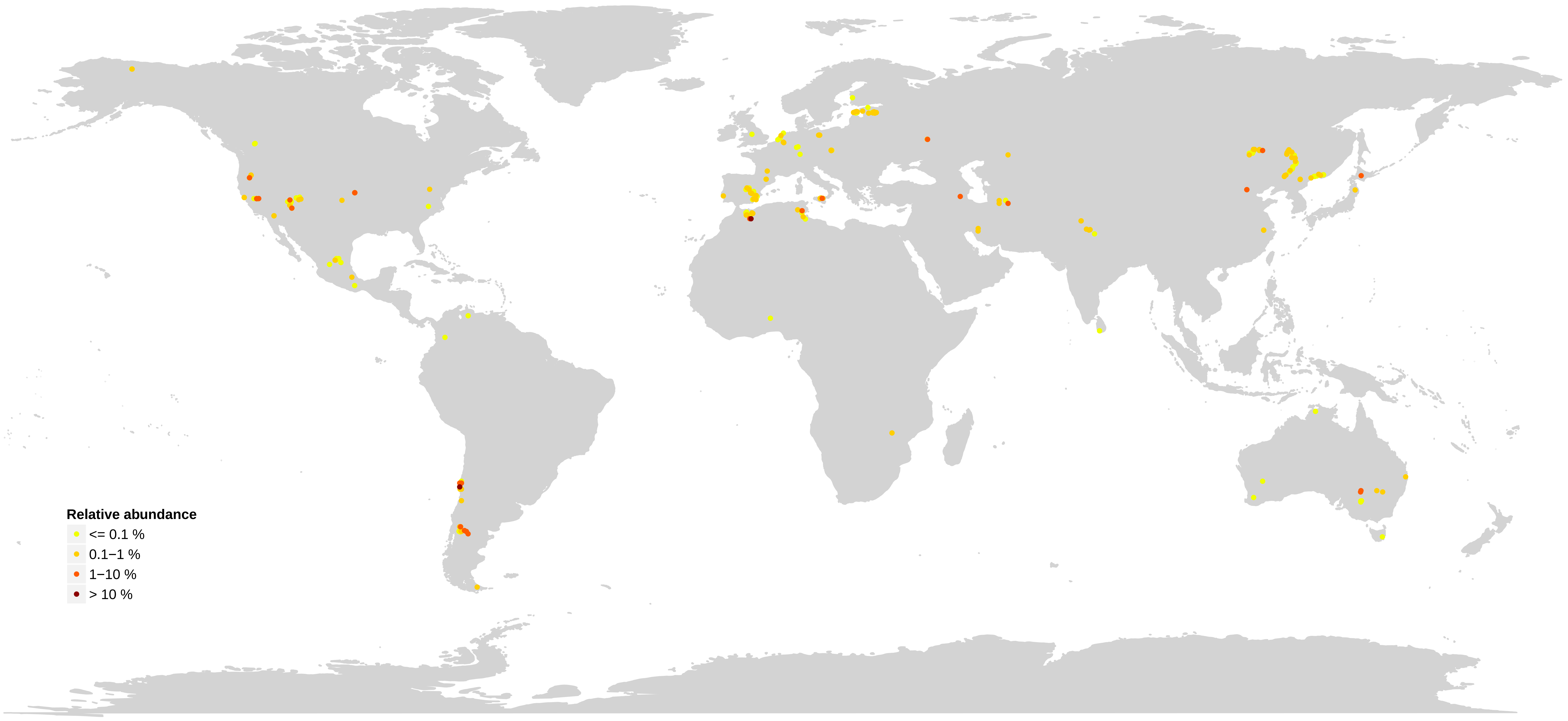

SH181879 Cryptococcus podzolicus

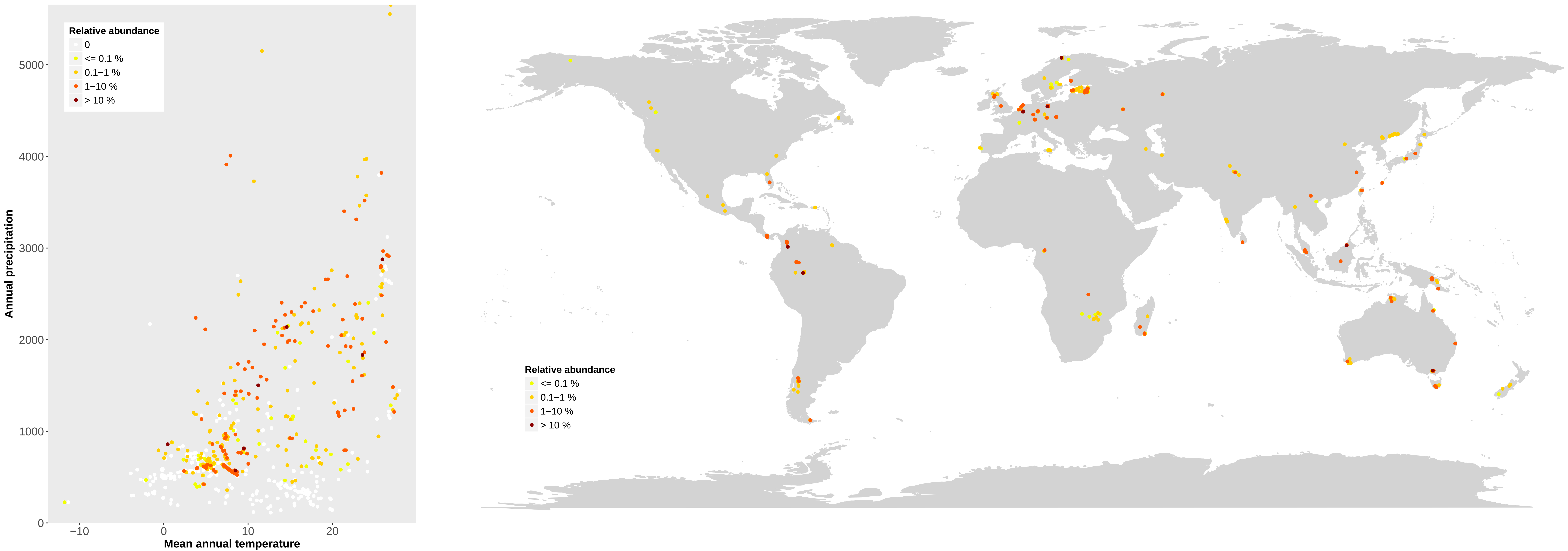

SH219581 Sordariomycetes sp

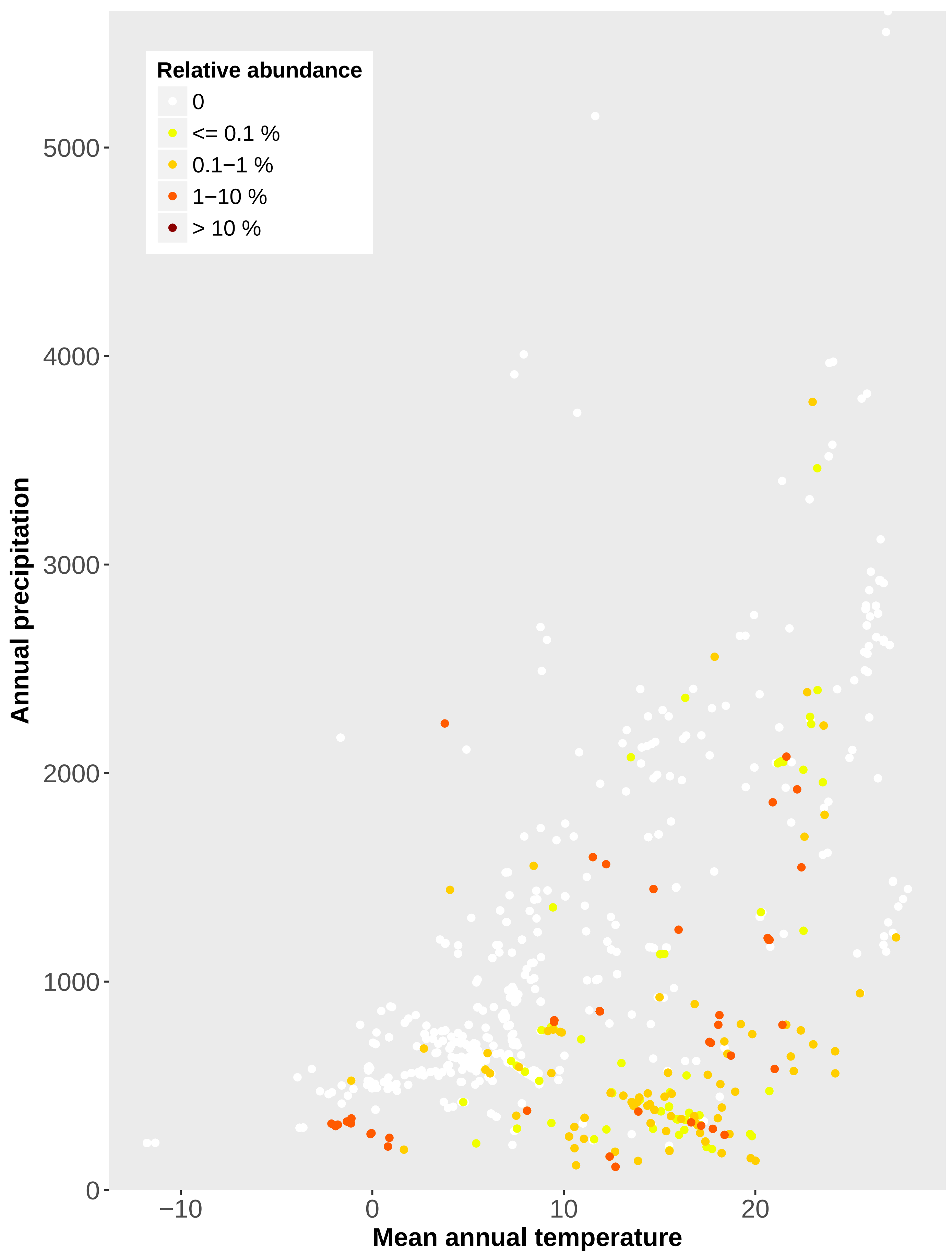

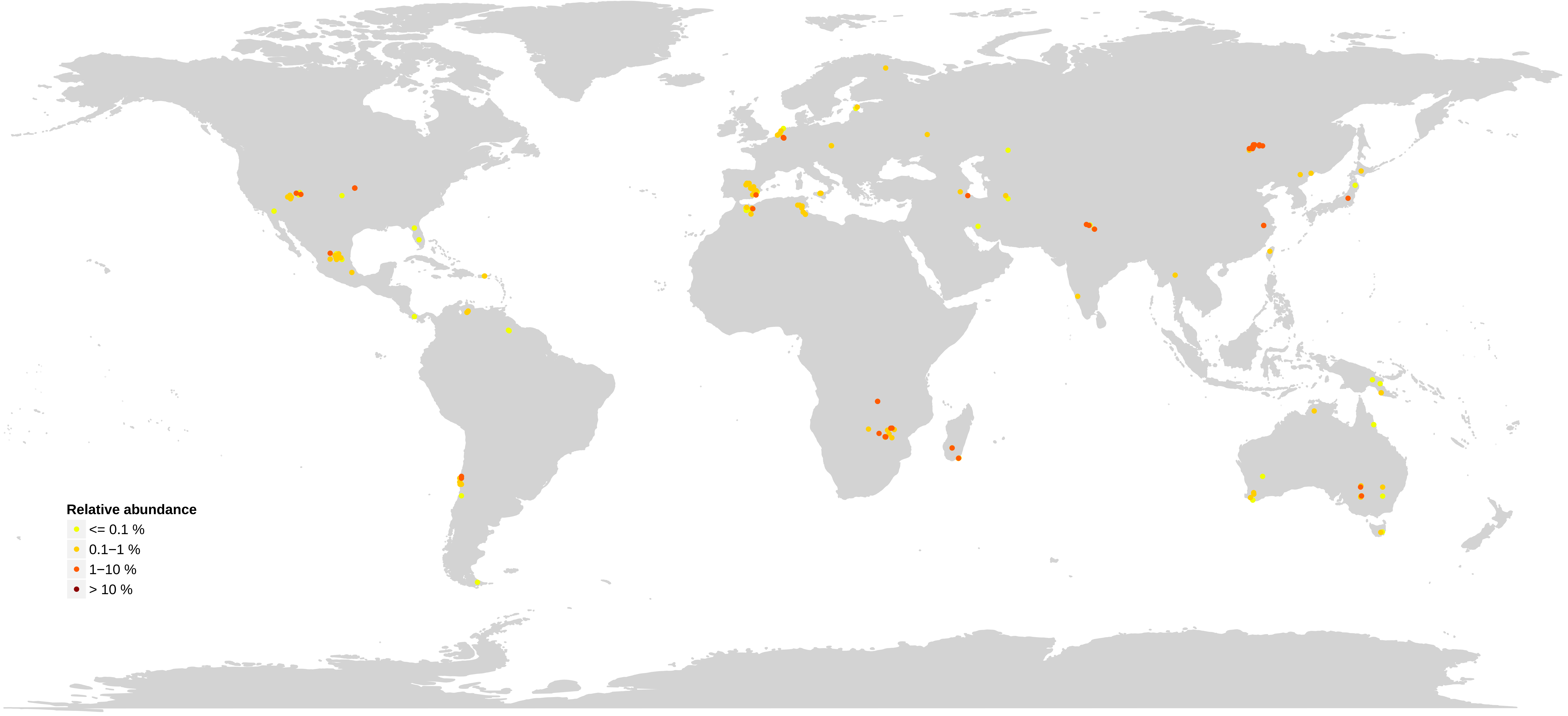

SH215928 Ascomycota sp

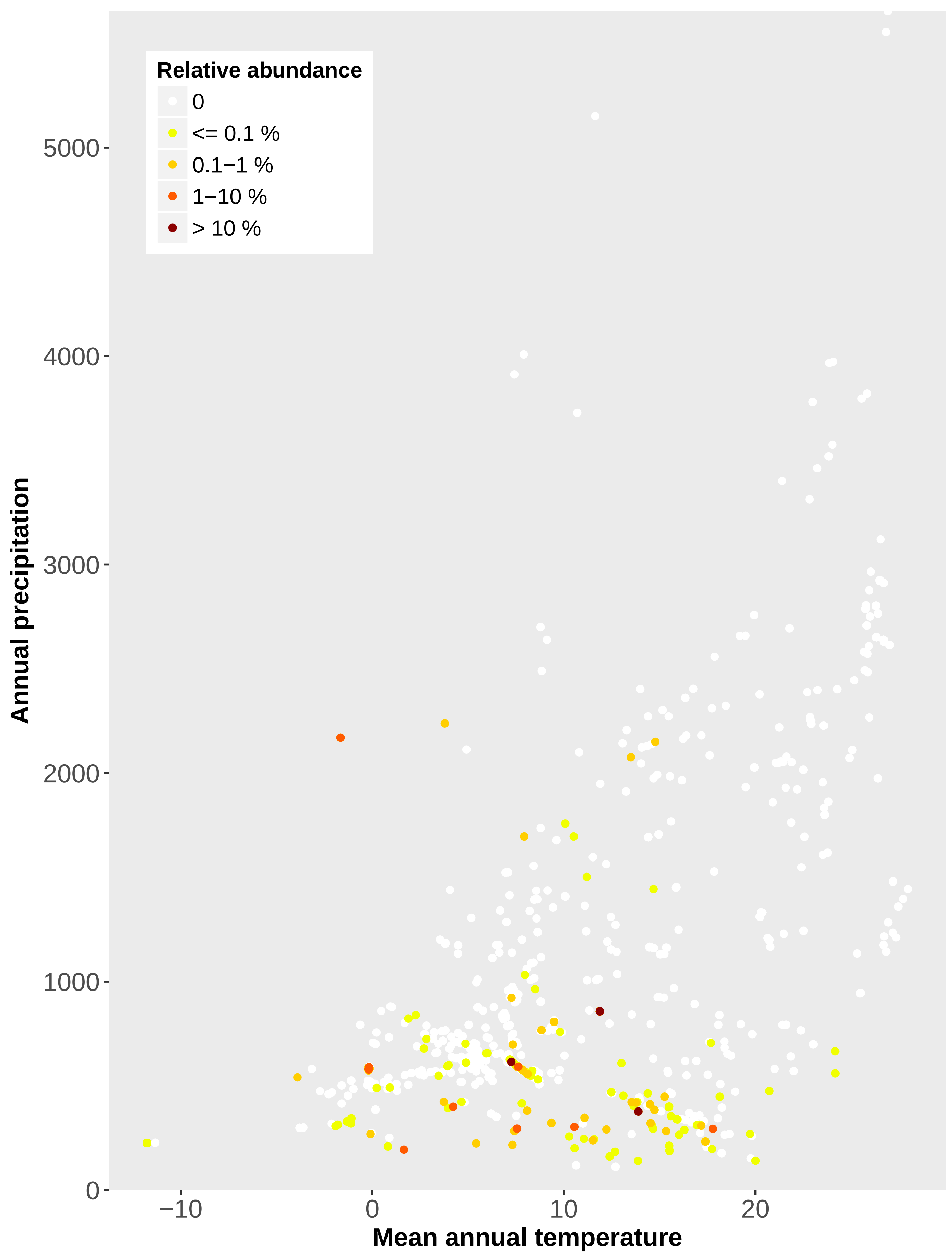

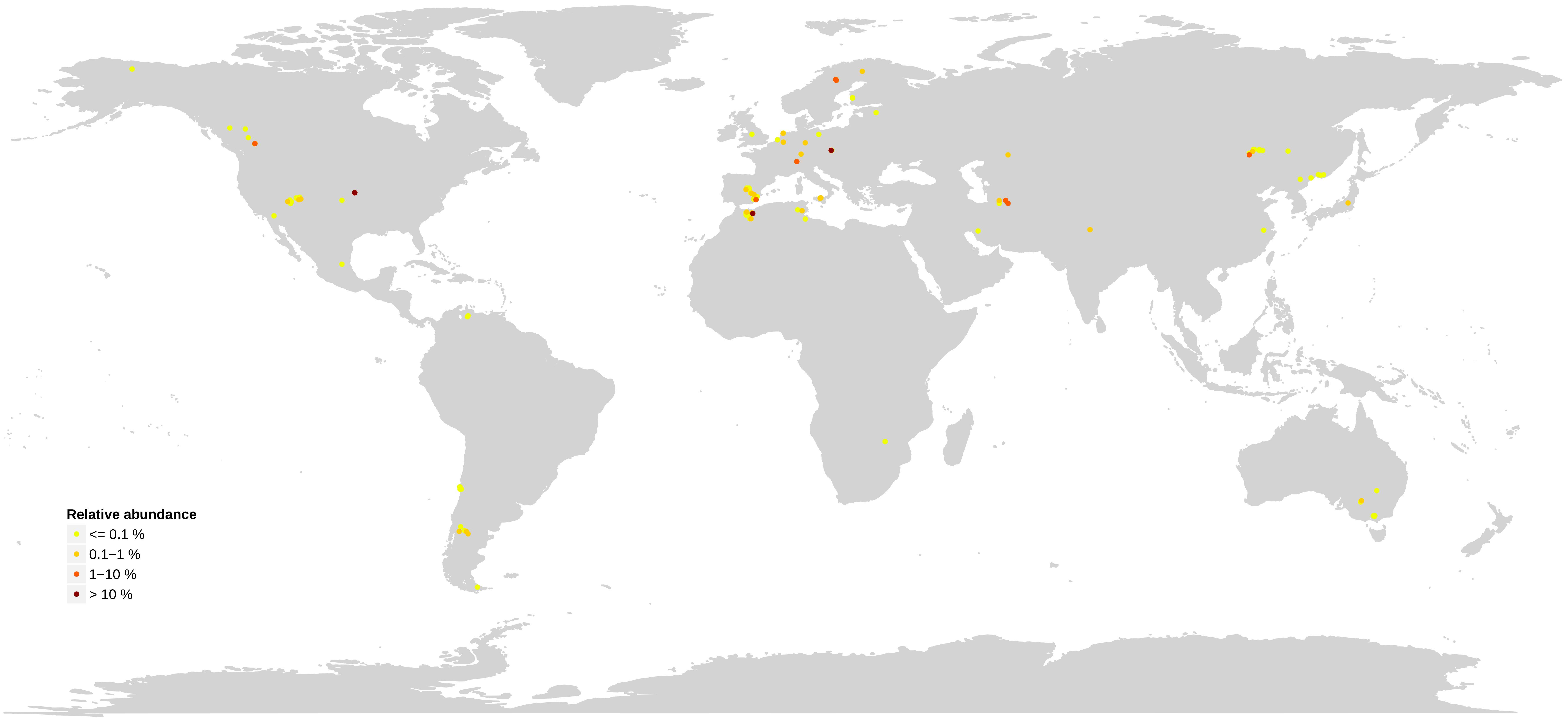

SH490540 Ascomycota sp

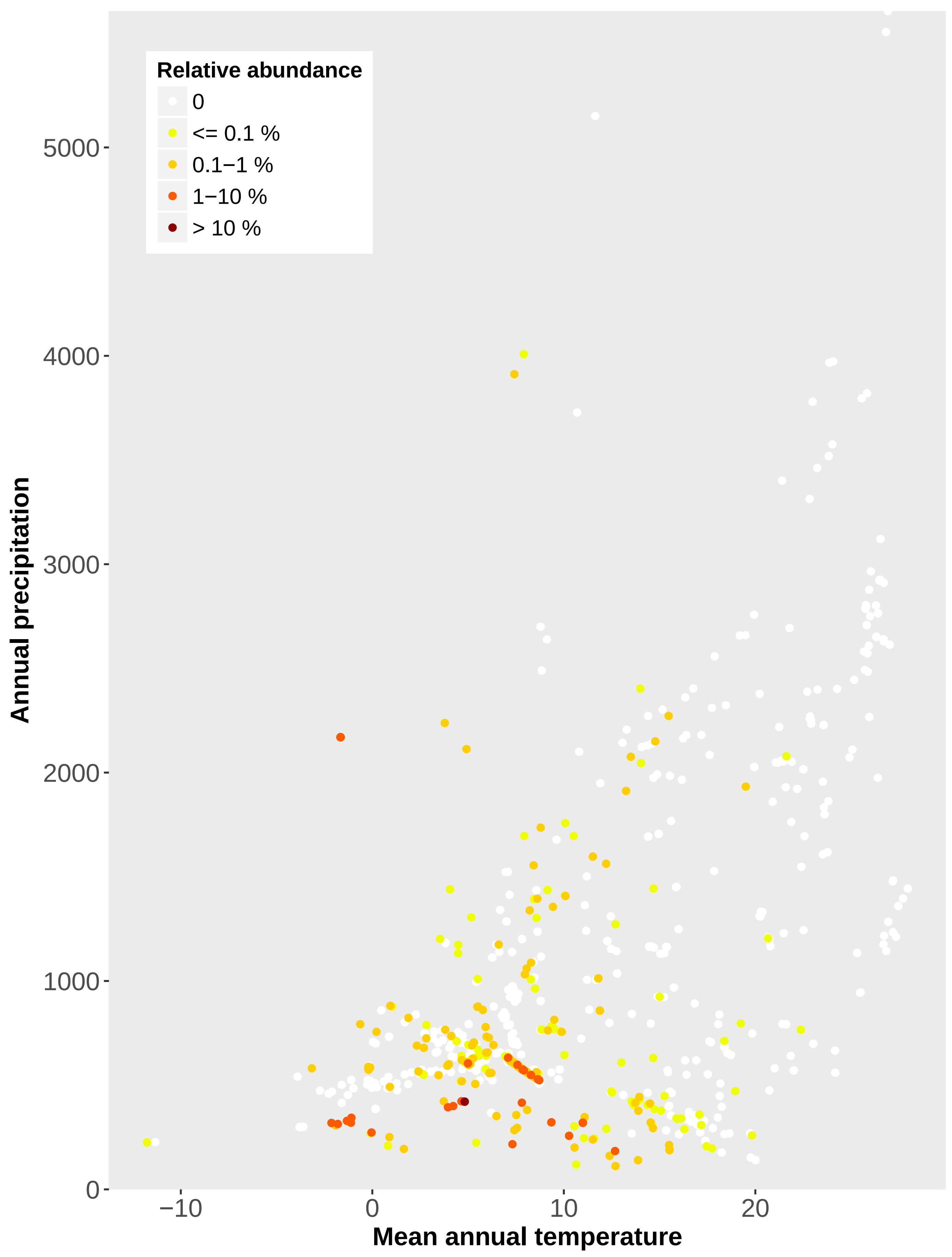

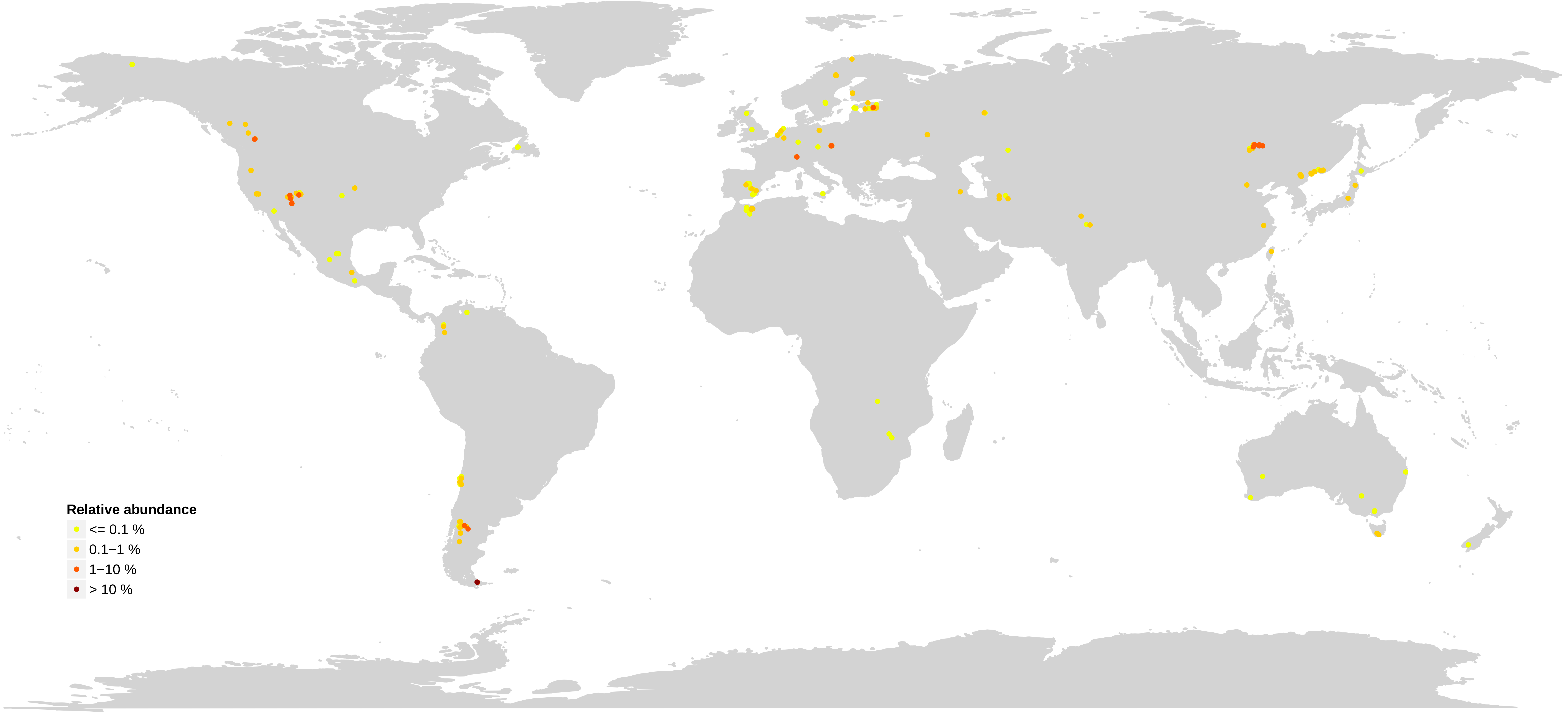

SH219674 Gibberella tricincta

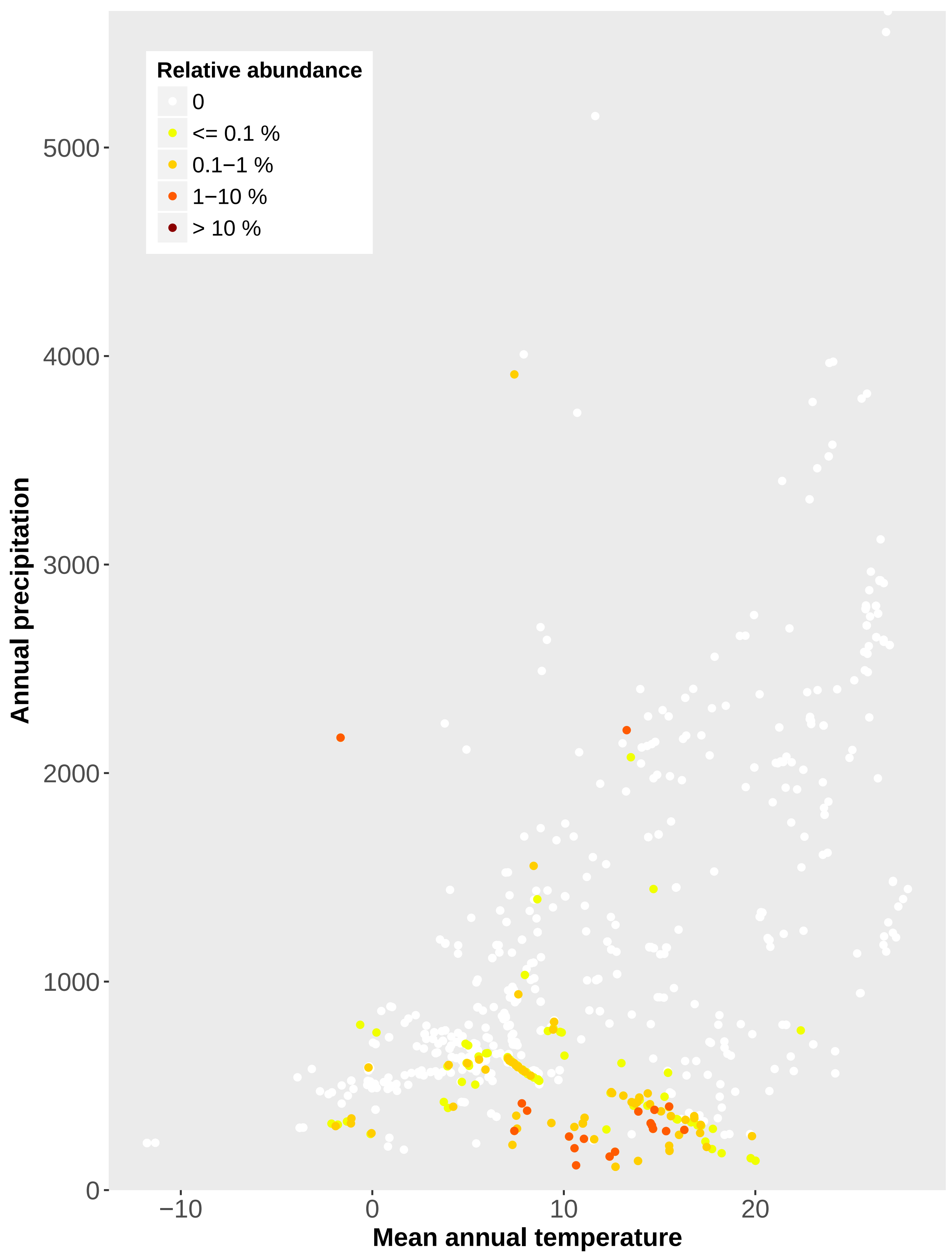

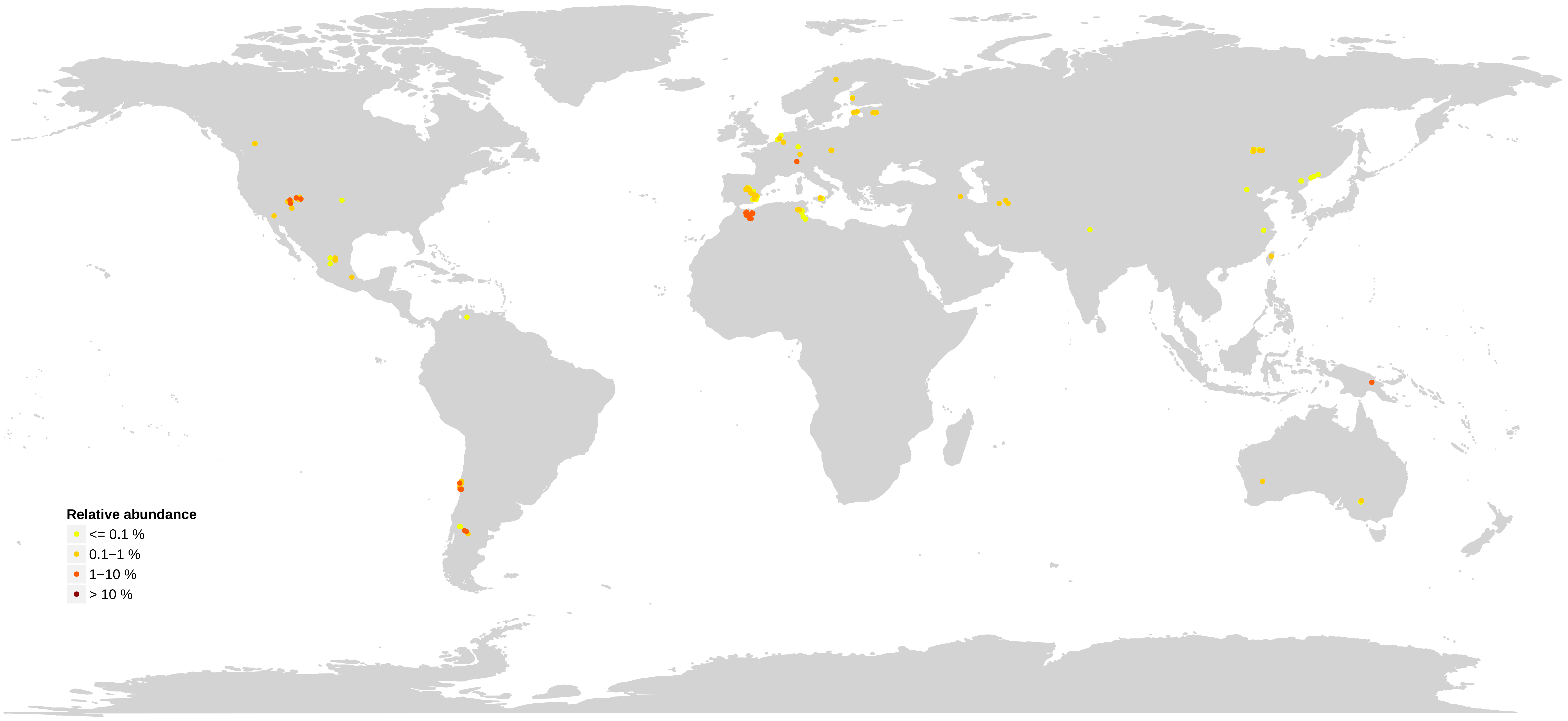

SH196224 Dermateaceae sp

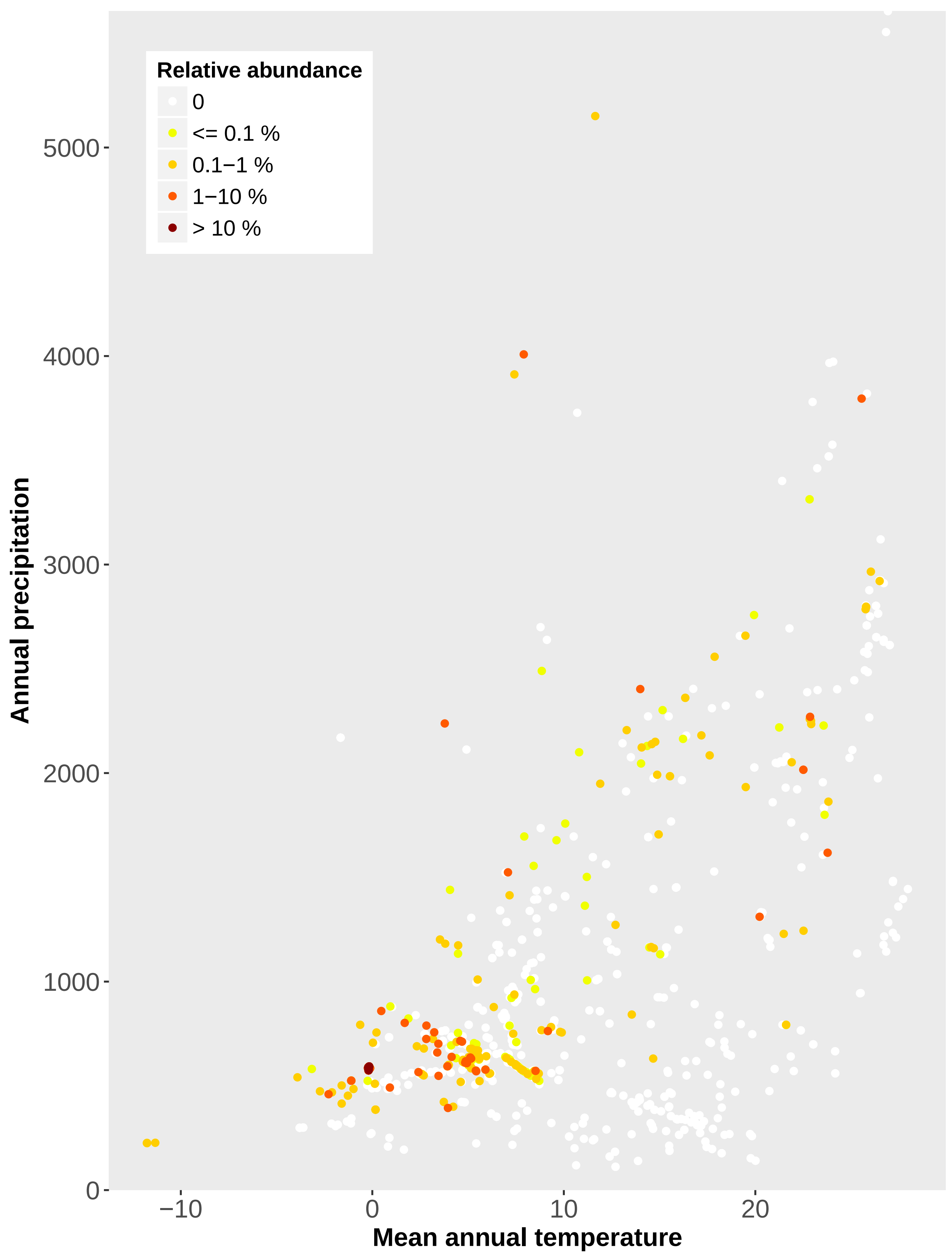

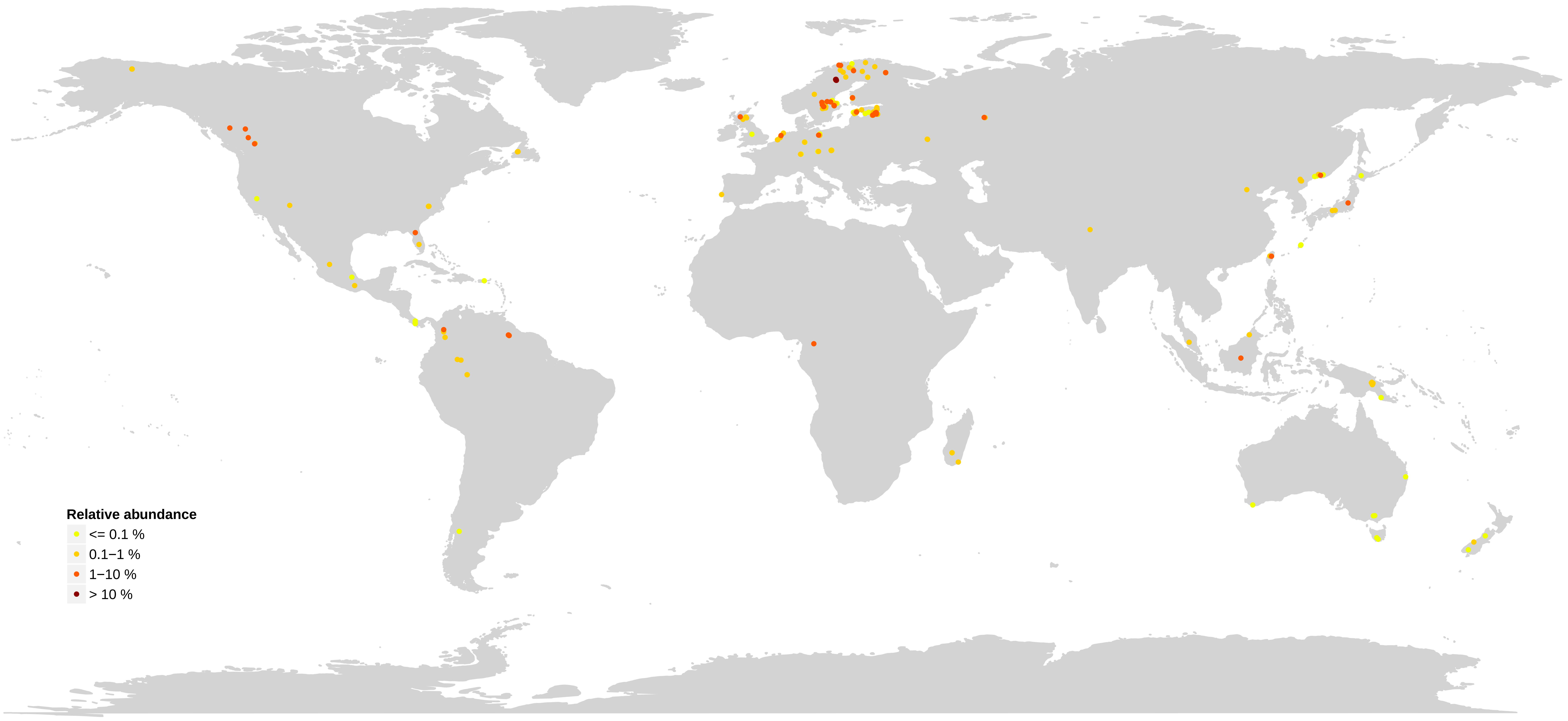

SH429908 Alternaria alternata

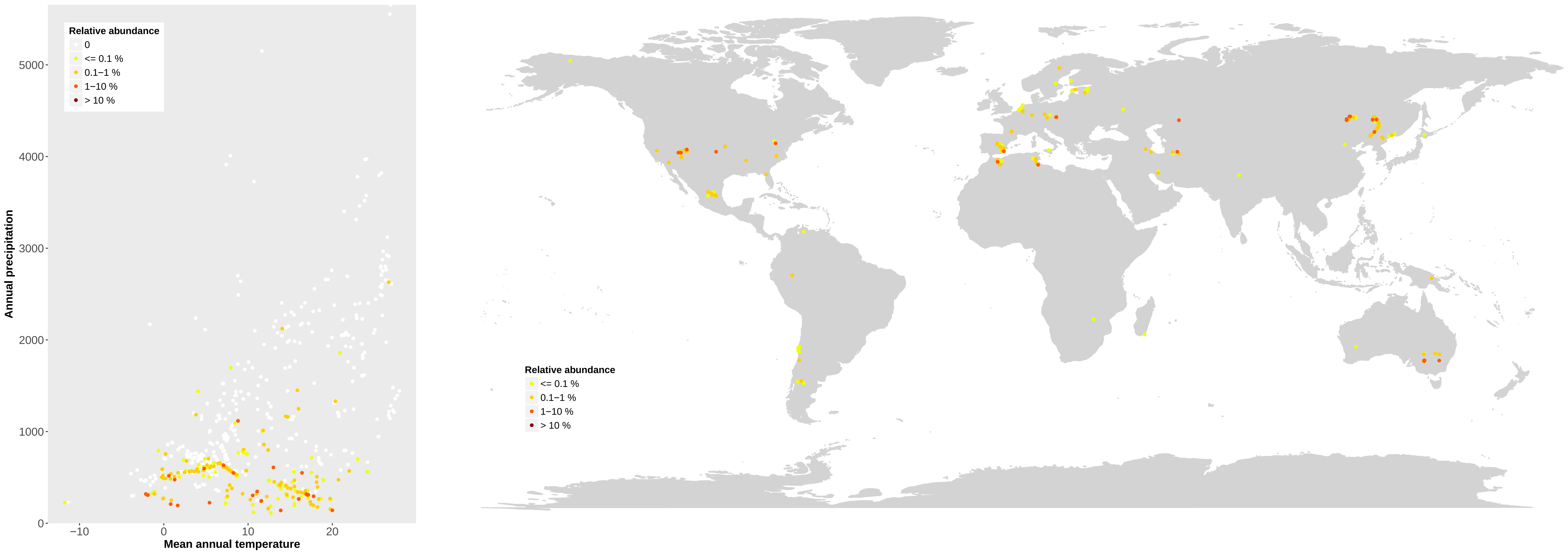

SH220731 Mycena sanguinolenta

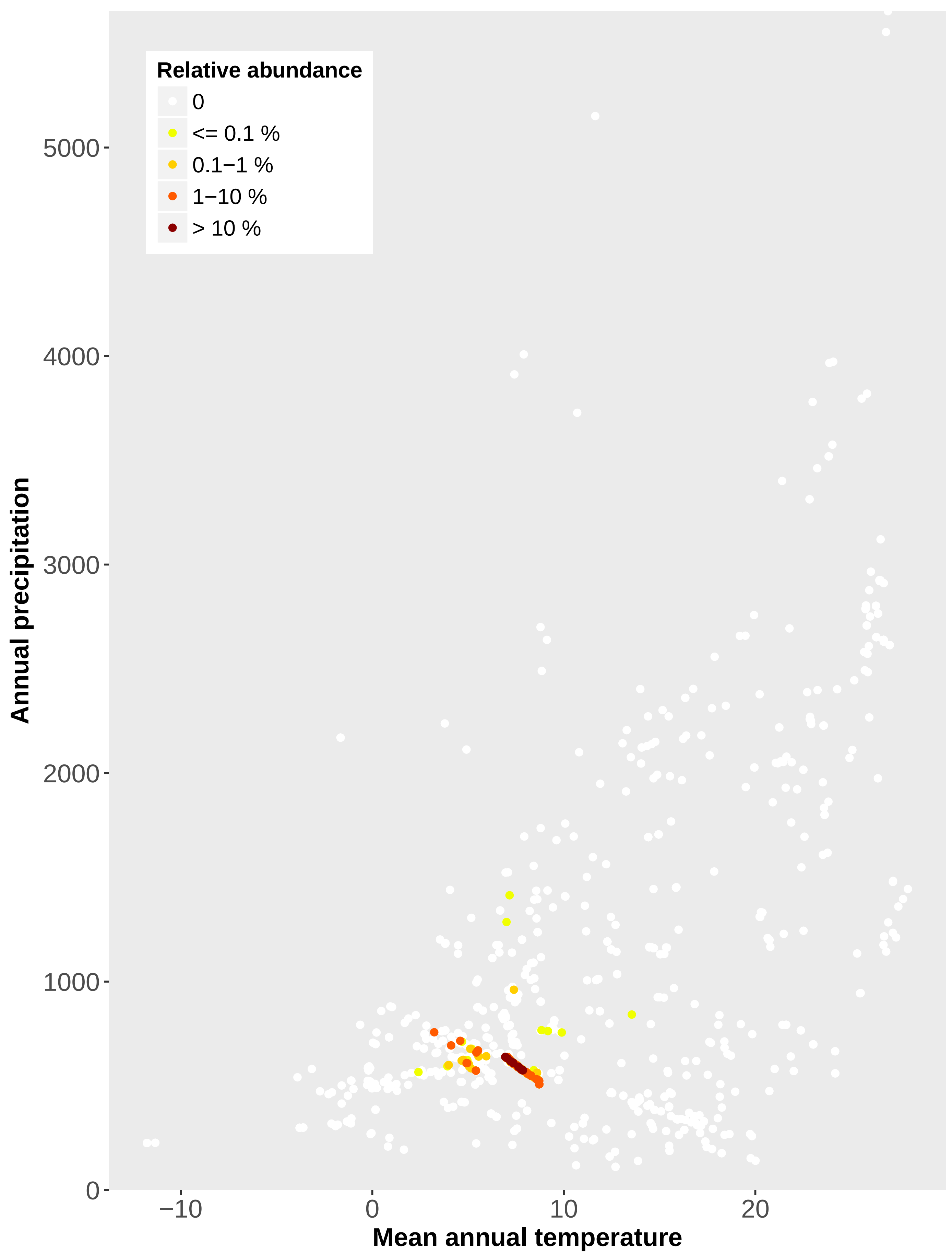

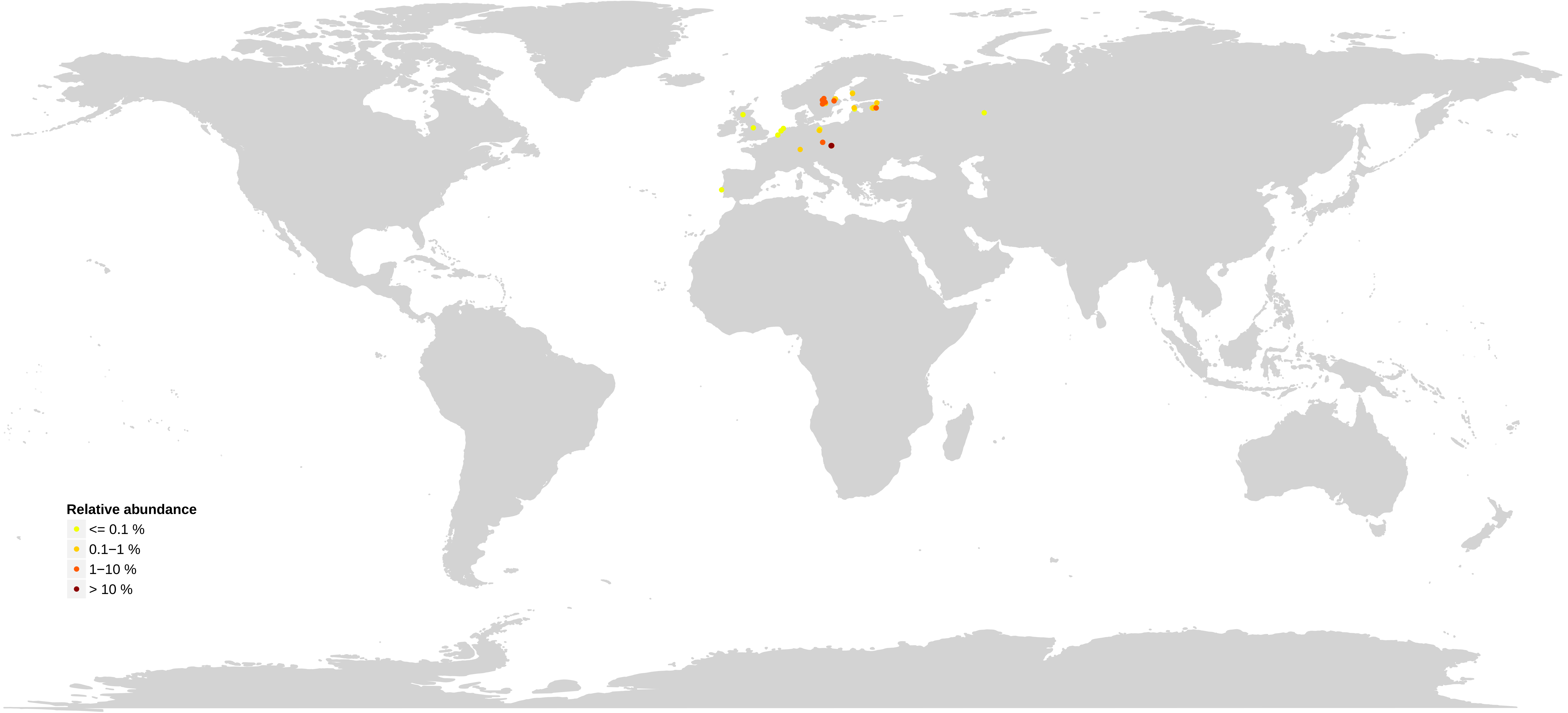

SH216099 Umbelopsis ramanniana

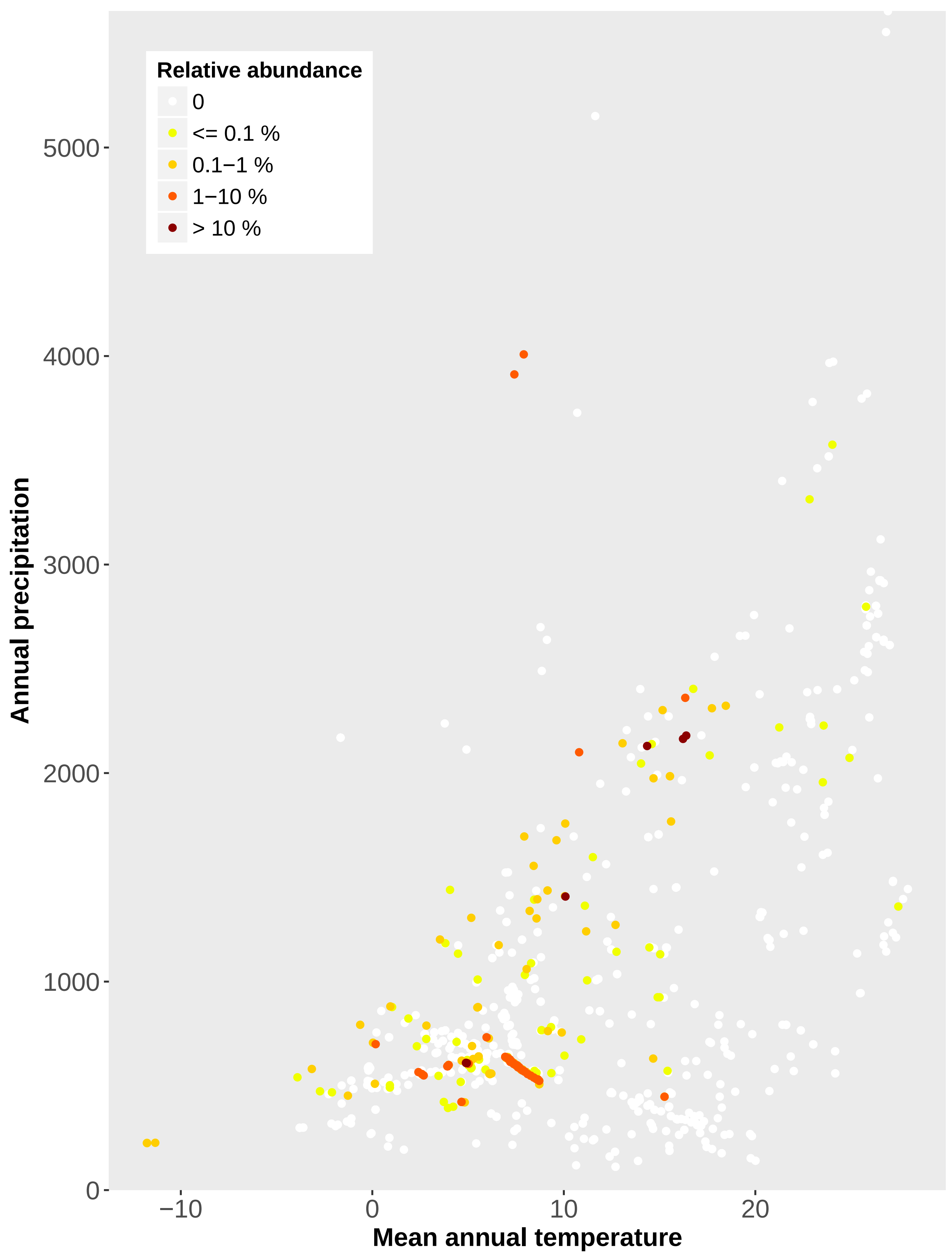

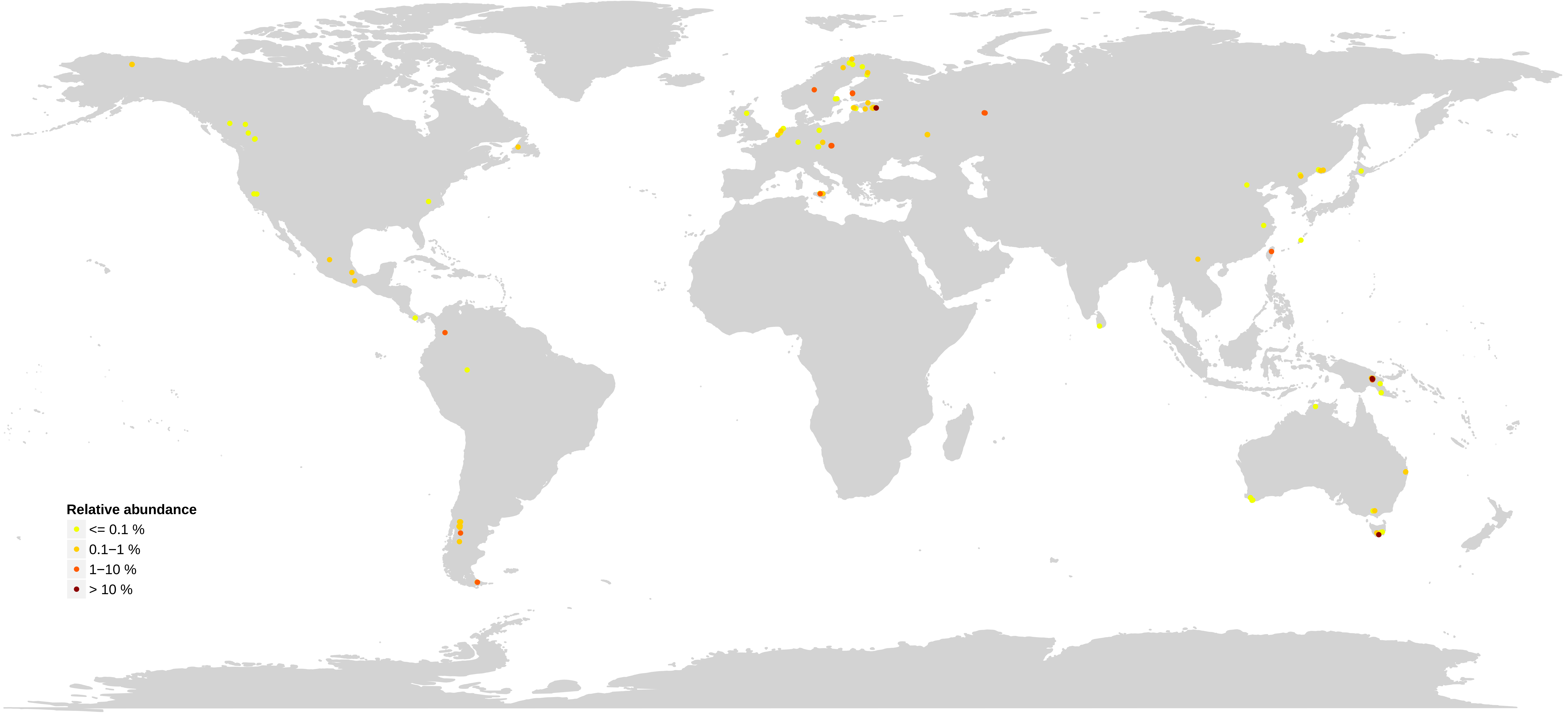

SH216991 Oidiodendron pilicola

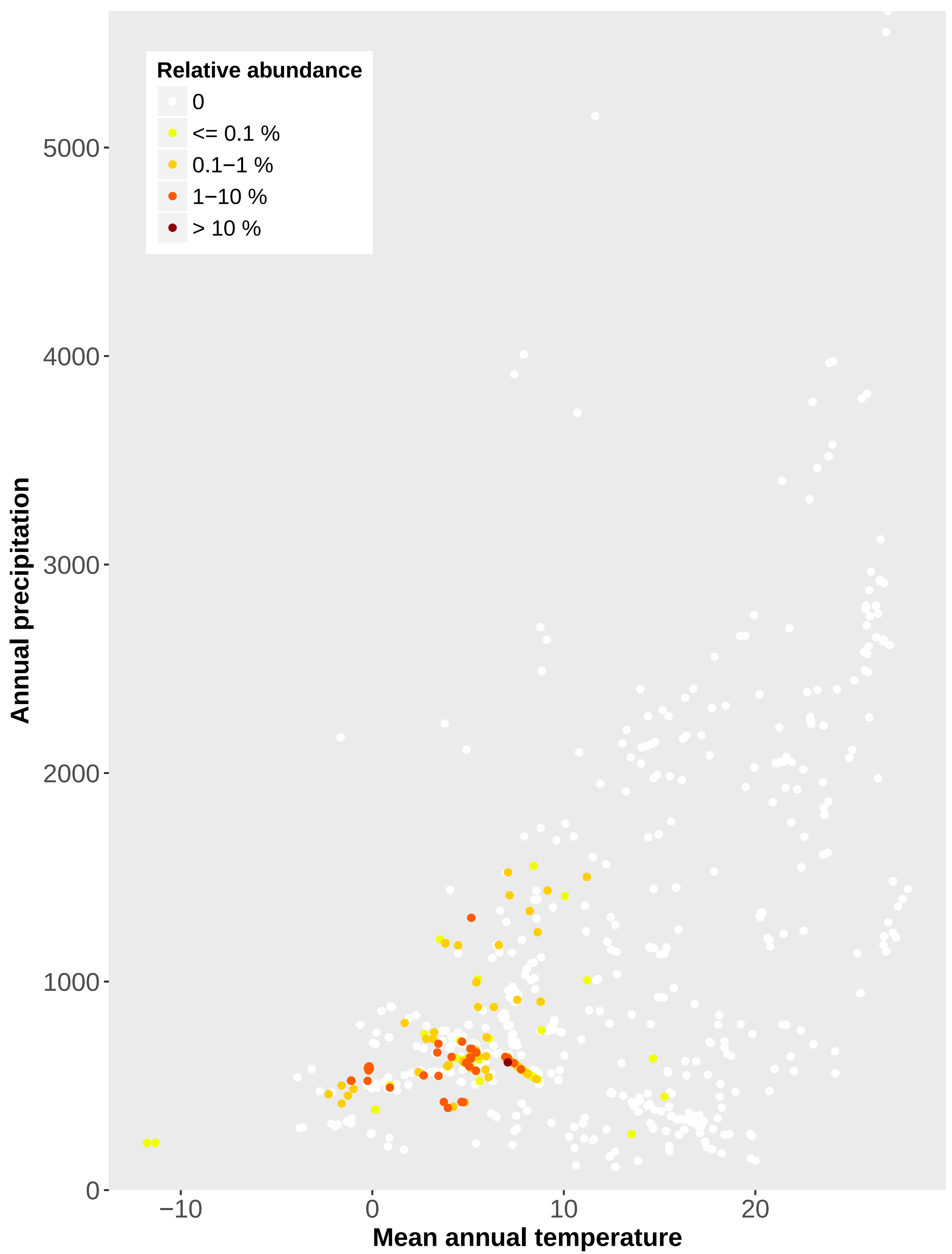

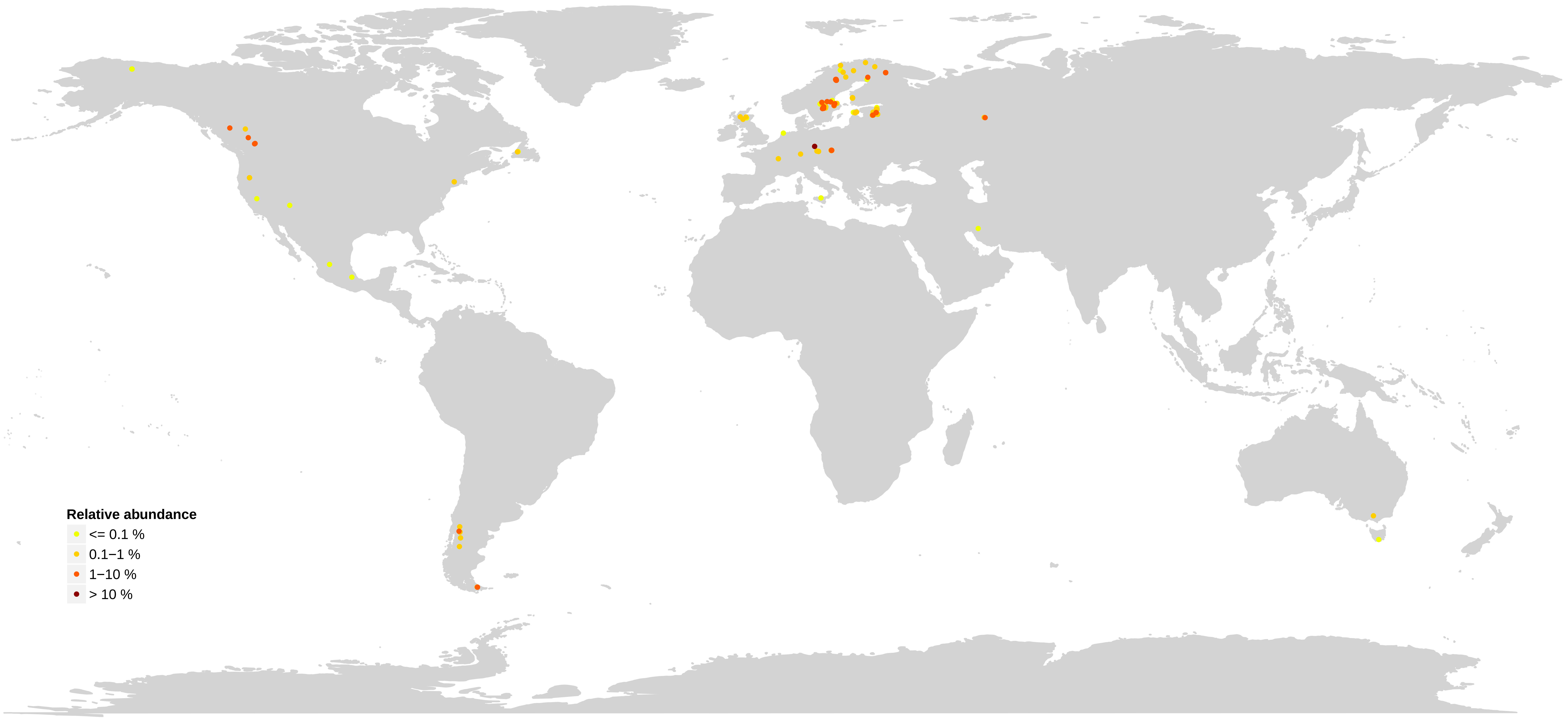

SH186943 Pleosporales sp

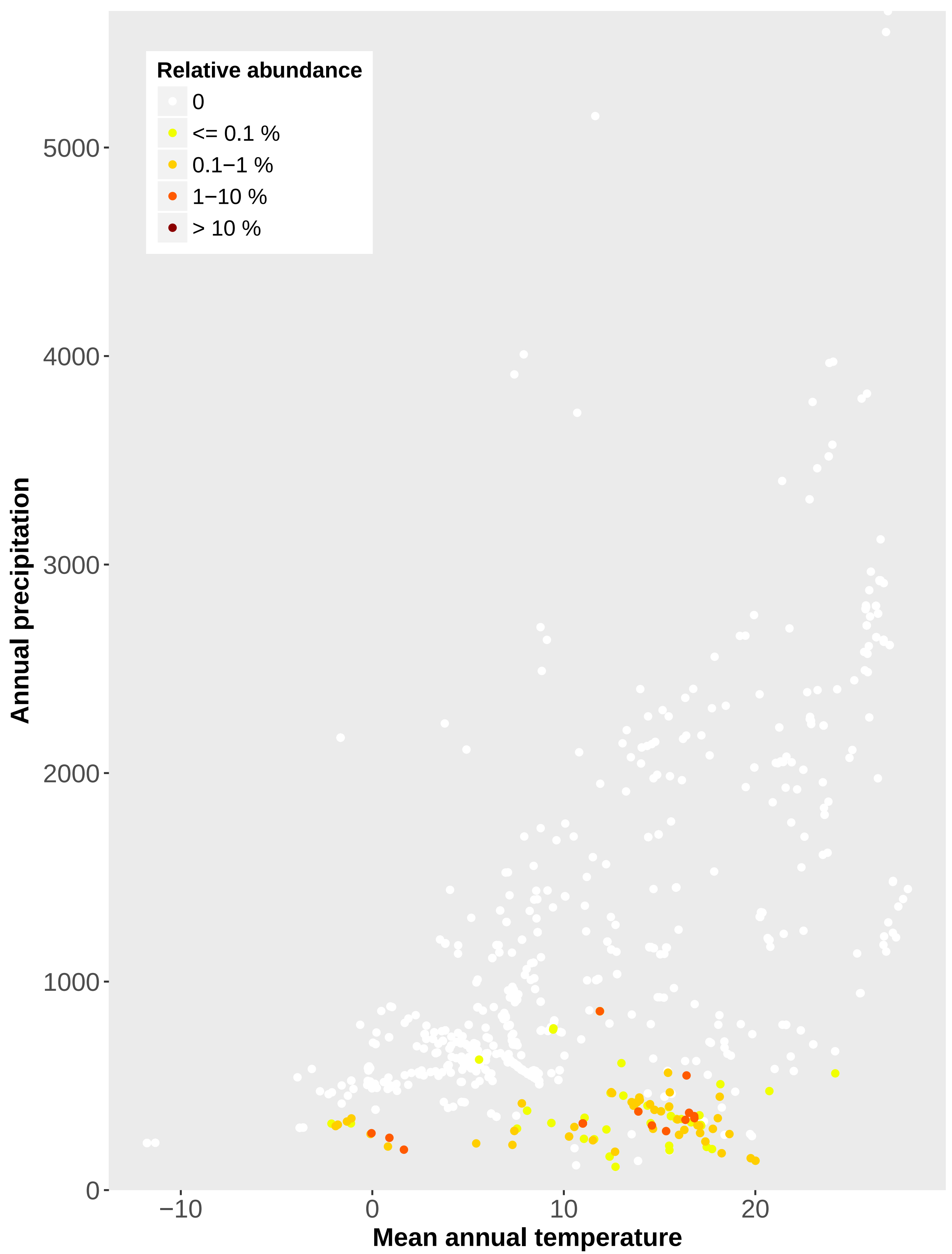

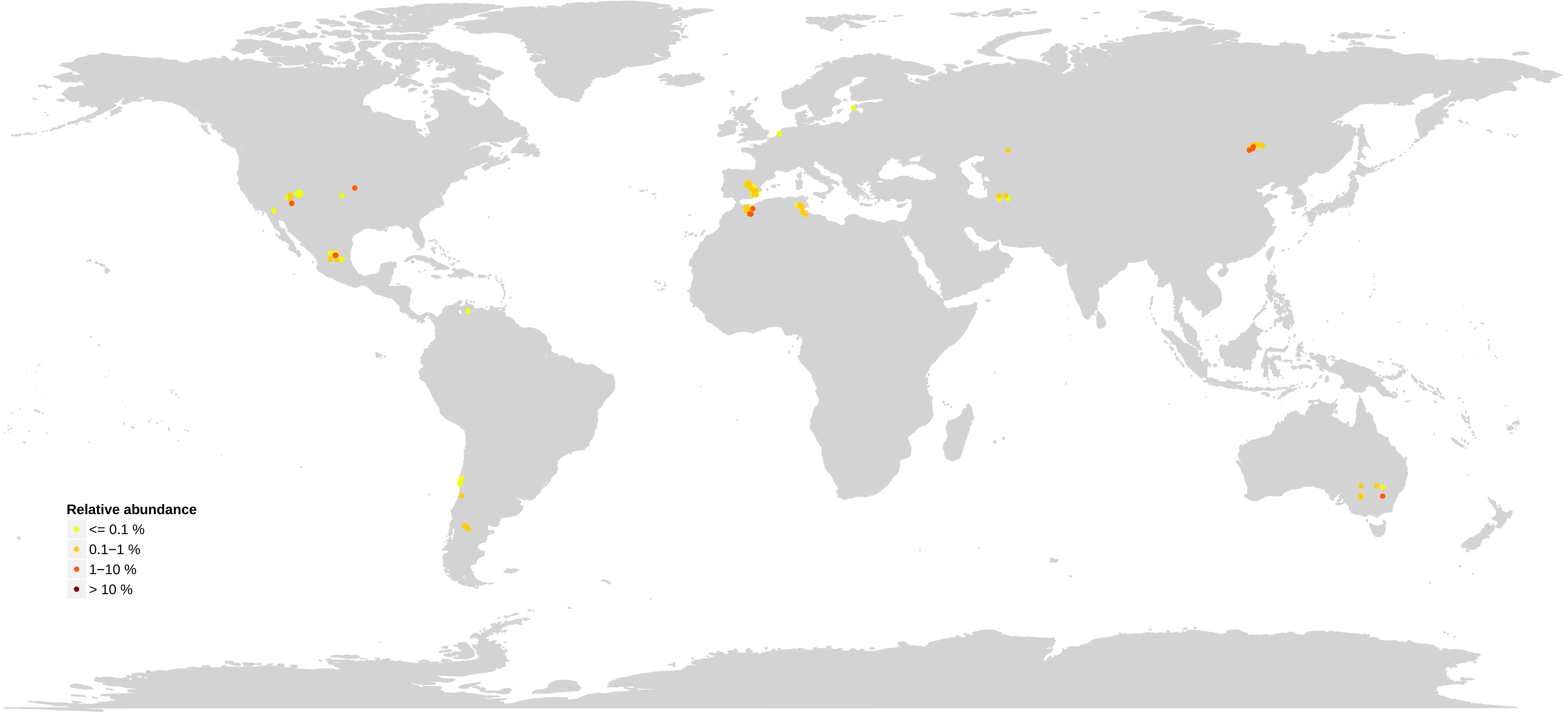

SH186707 Russula cyanoxantha

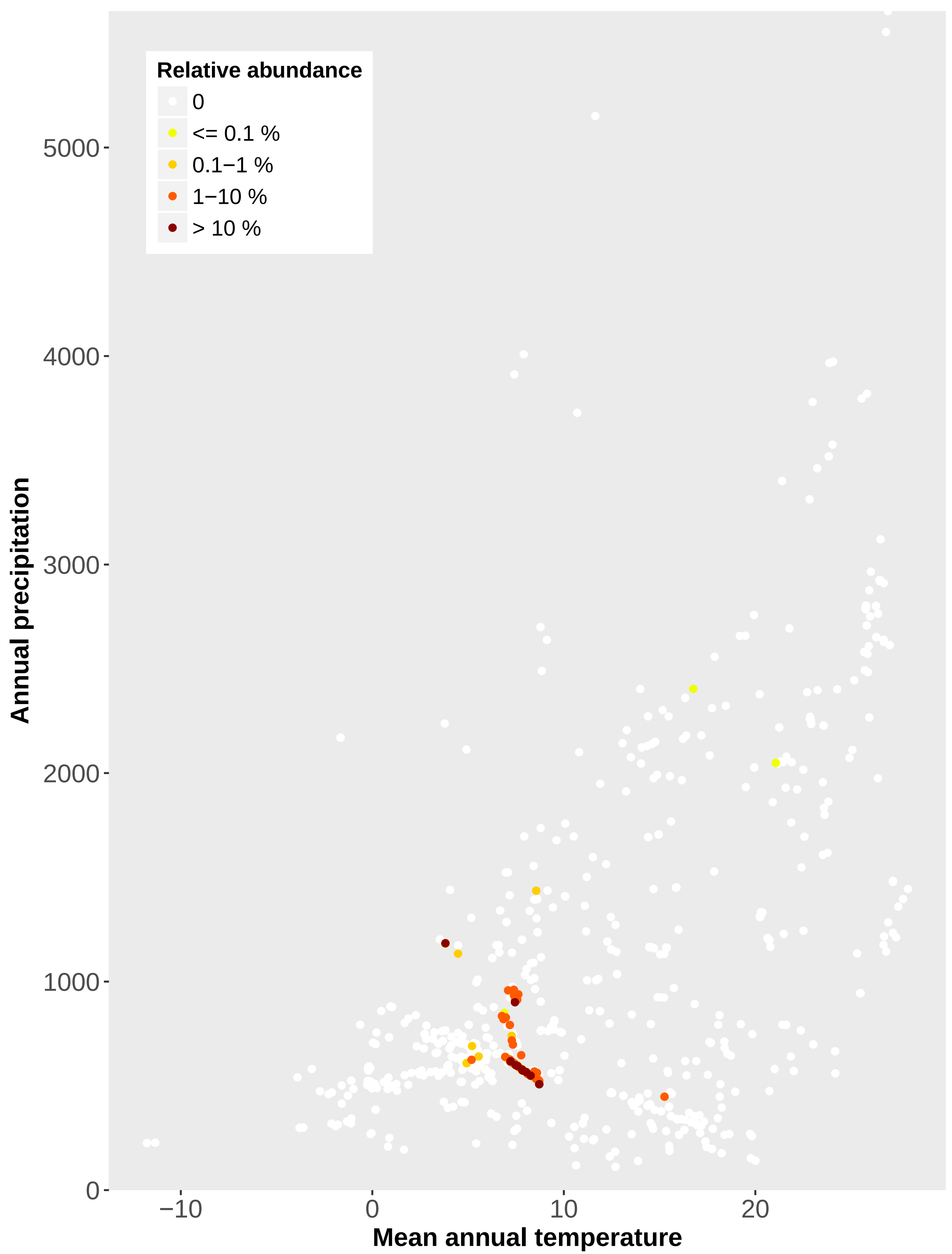

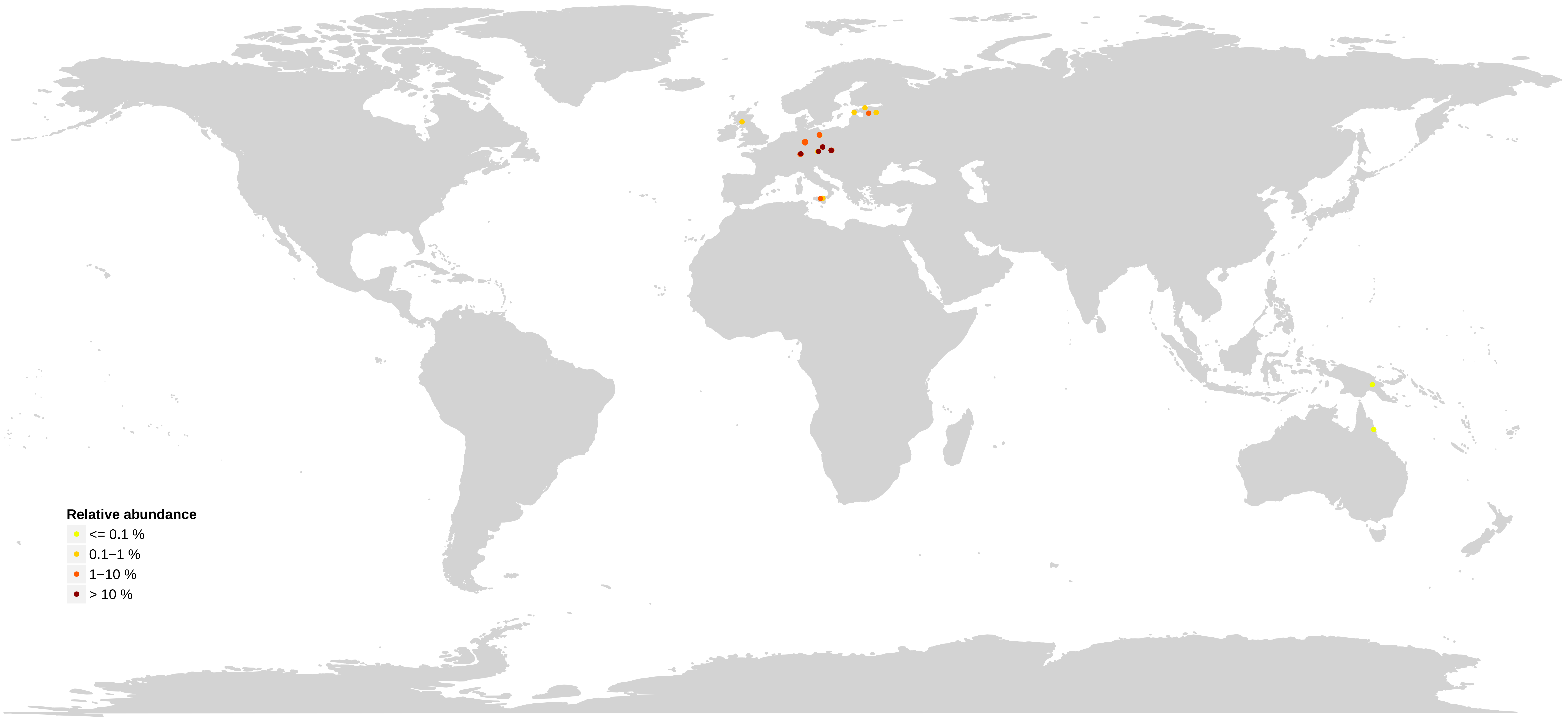

SH023500 Trichocomaceae sp

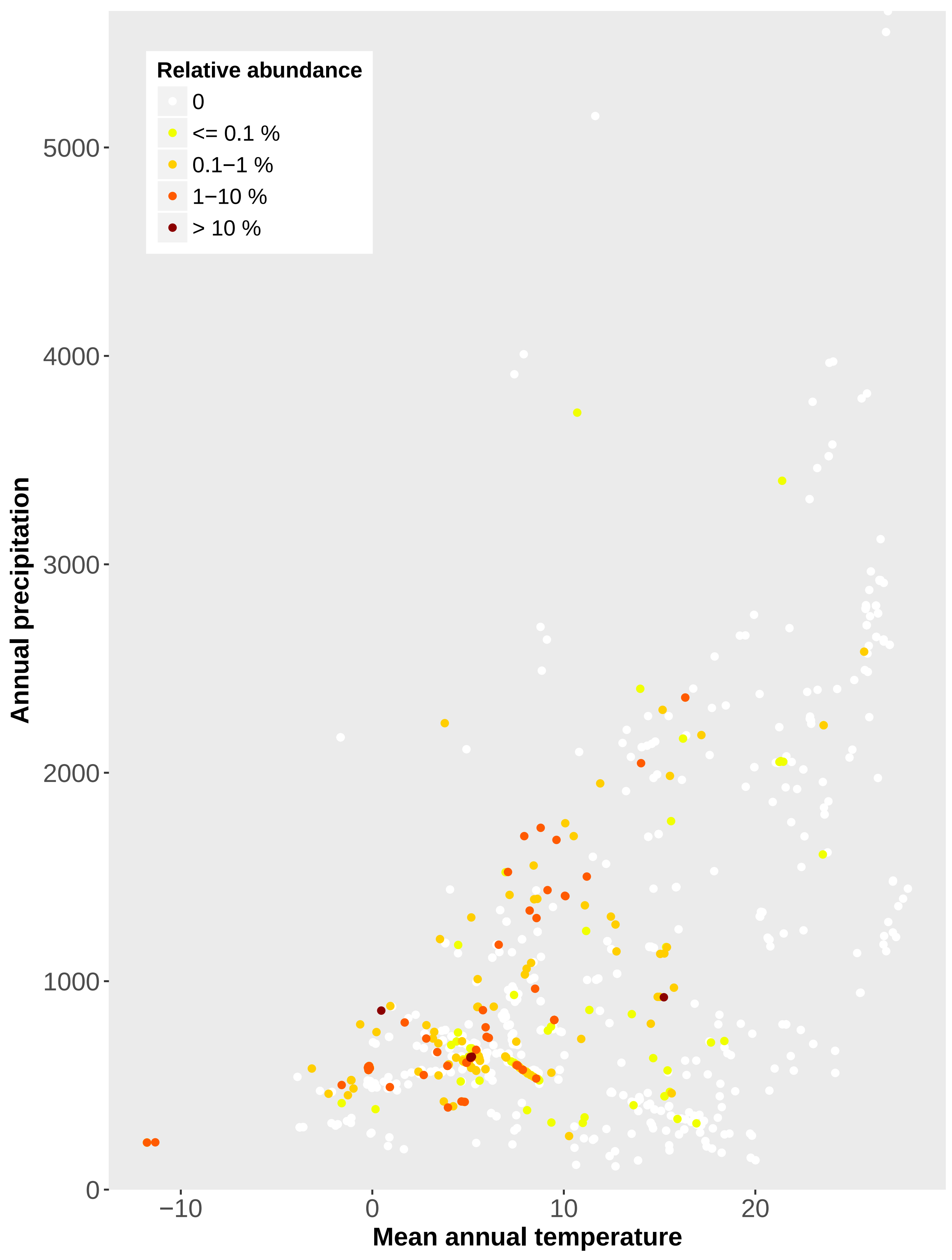

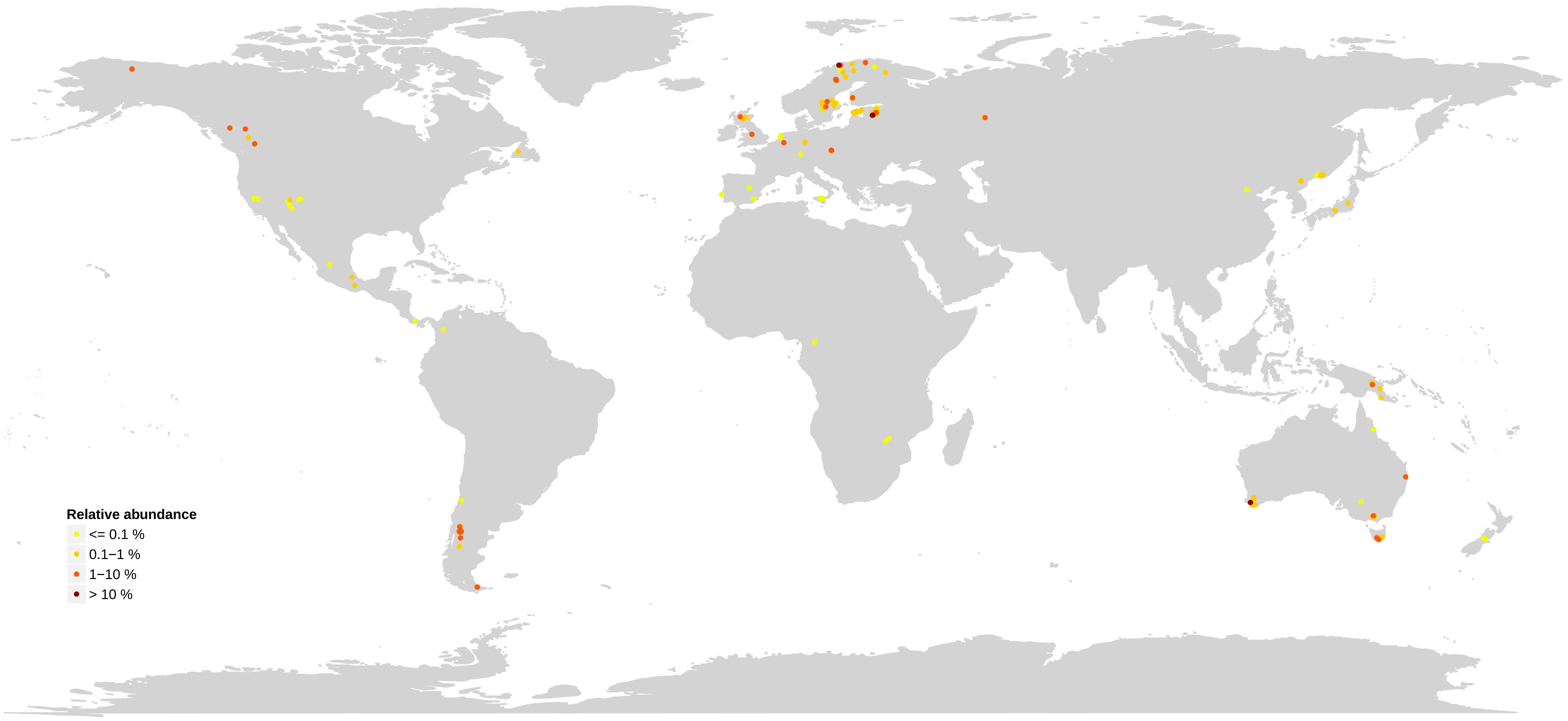

SH005080 Tremellomycetes sp

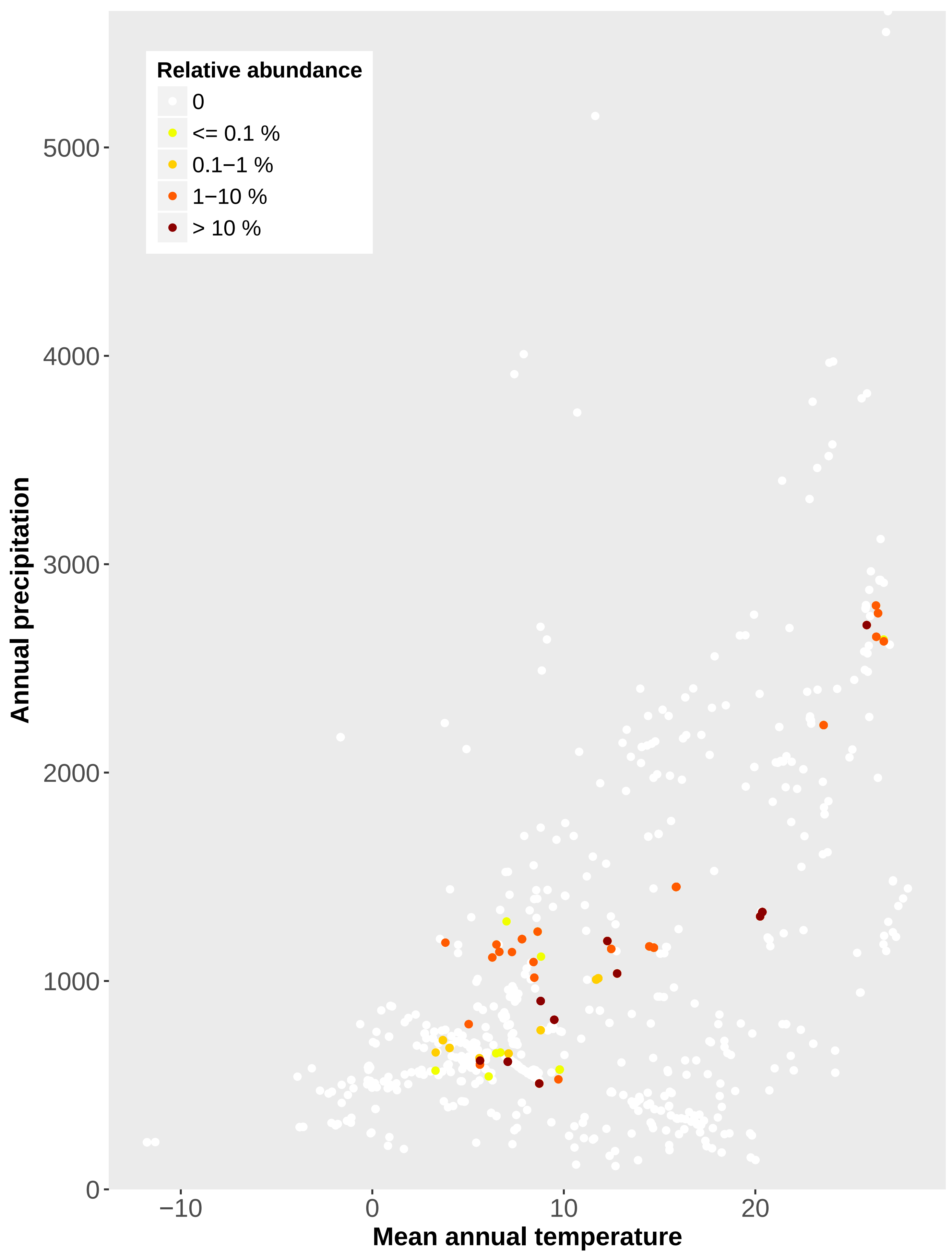

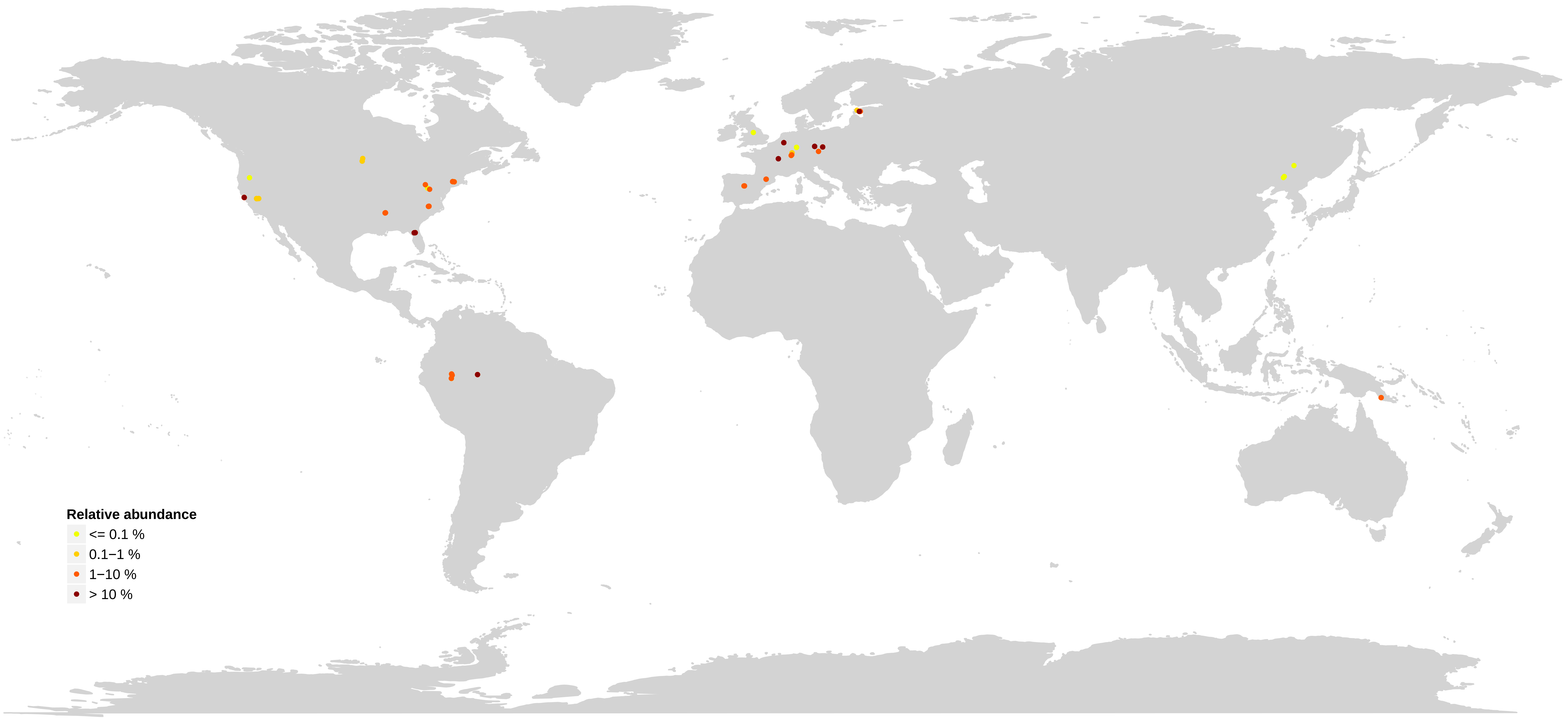

SH216250 Mycosphaerella tassiana

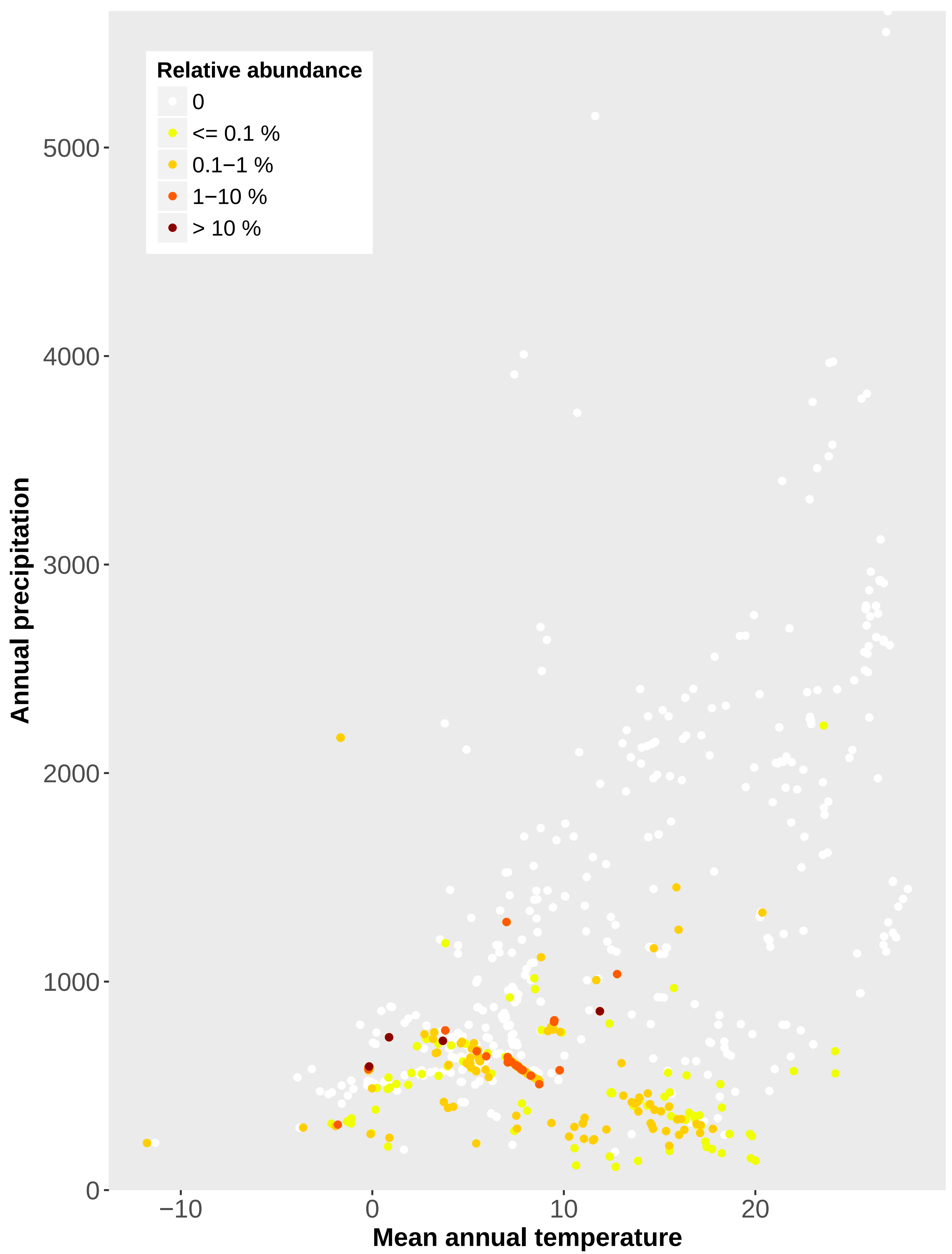

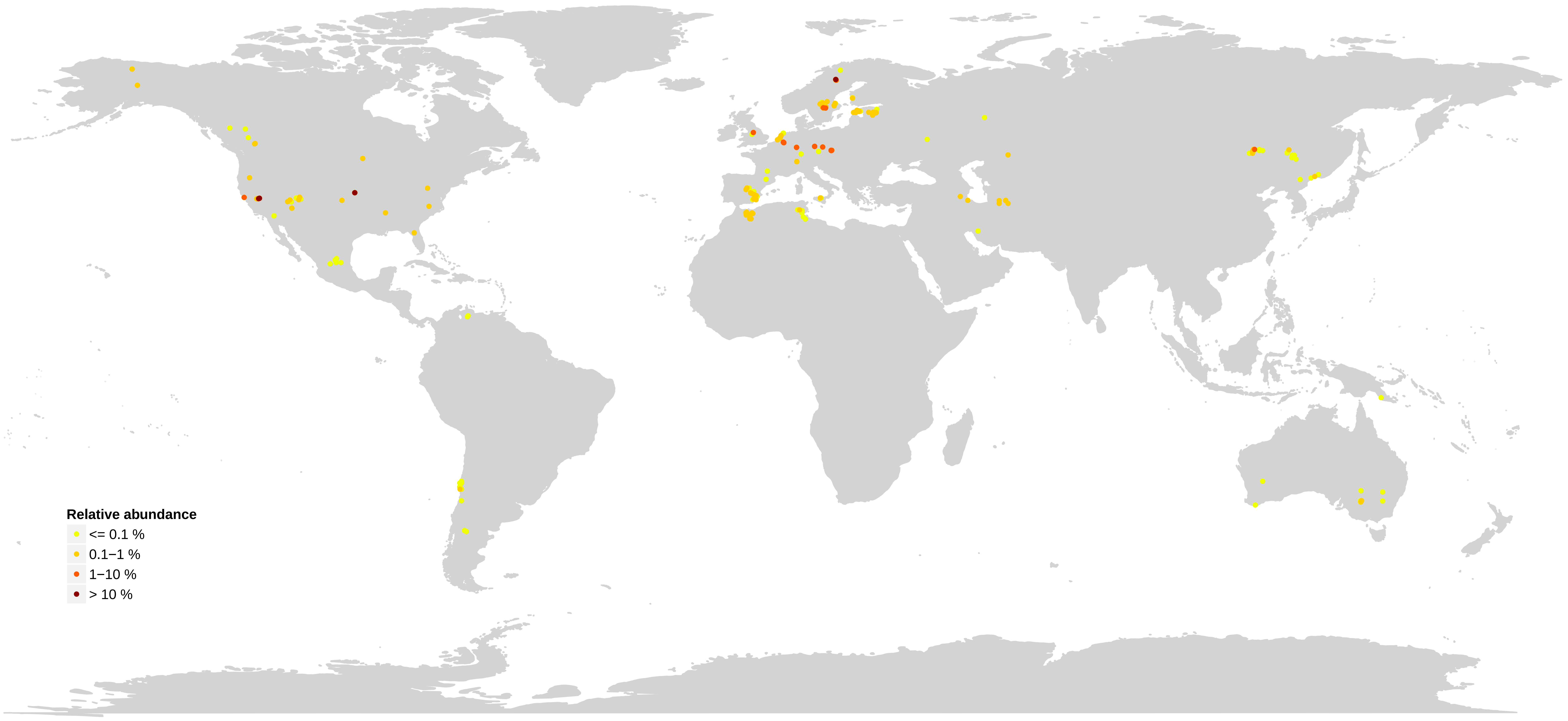

SH490662 Sporormiaceae sp

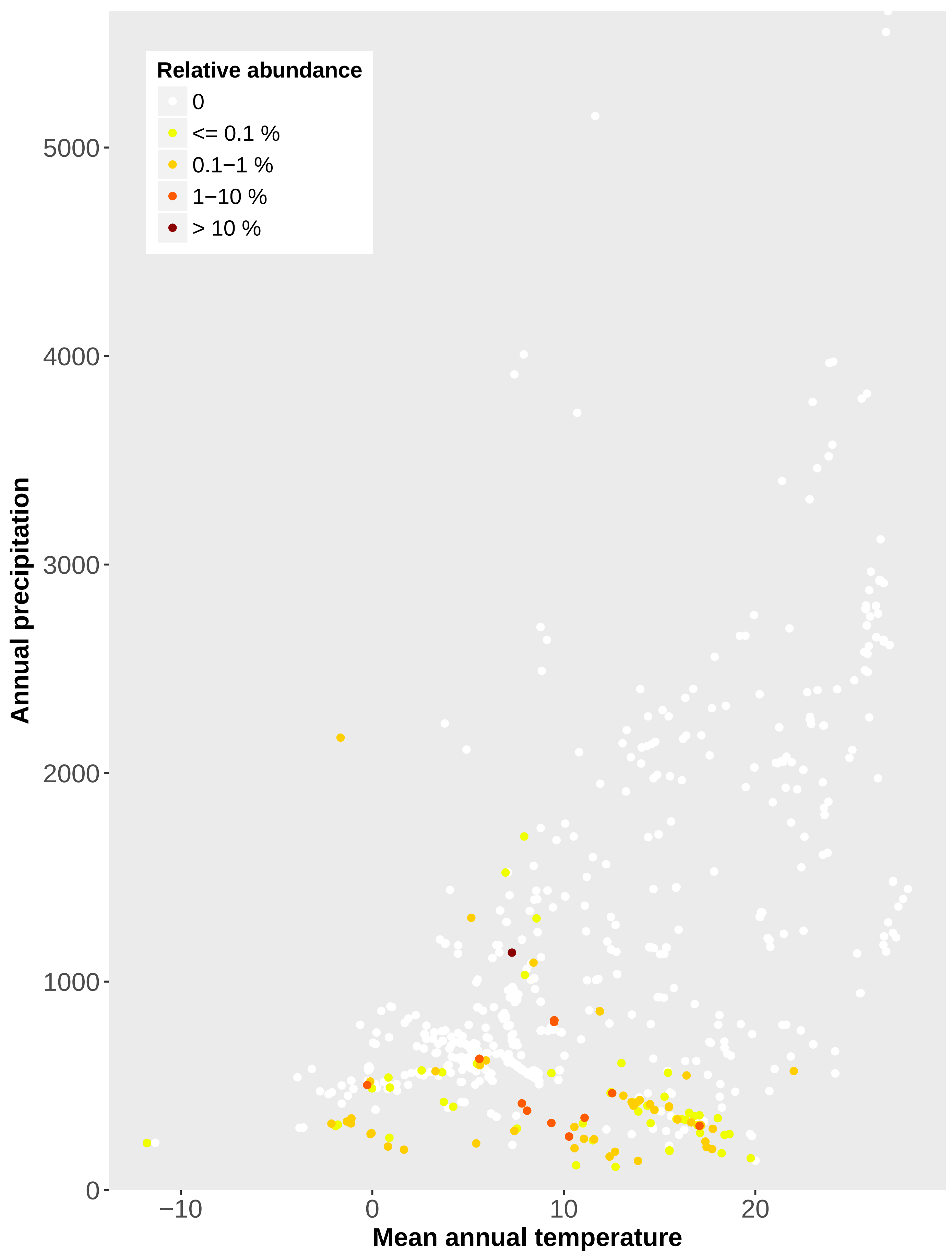

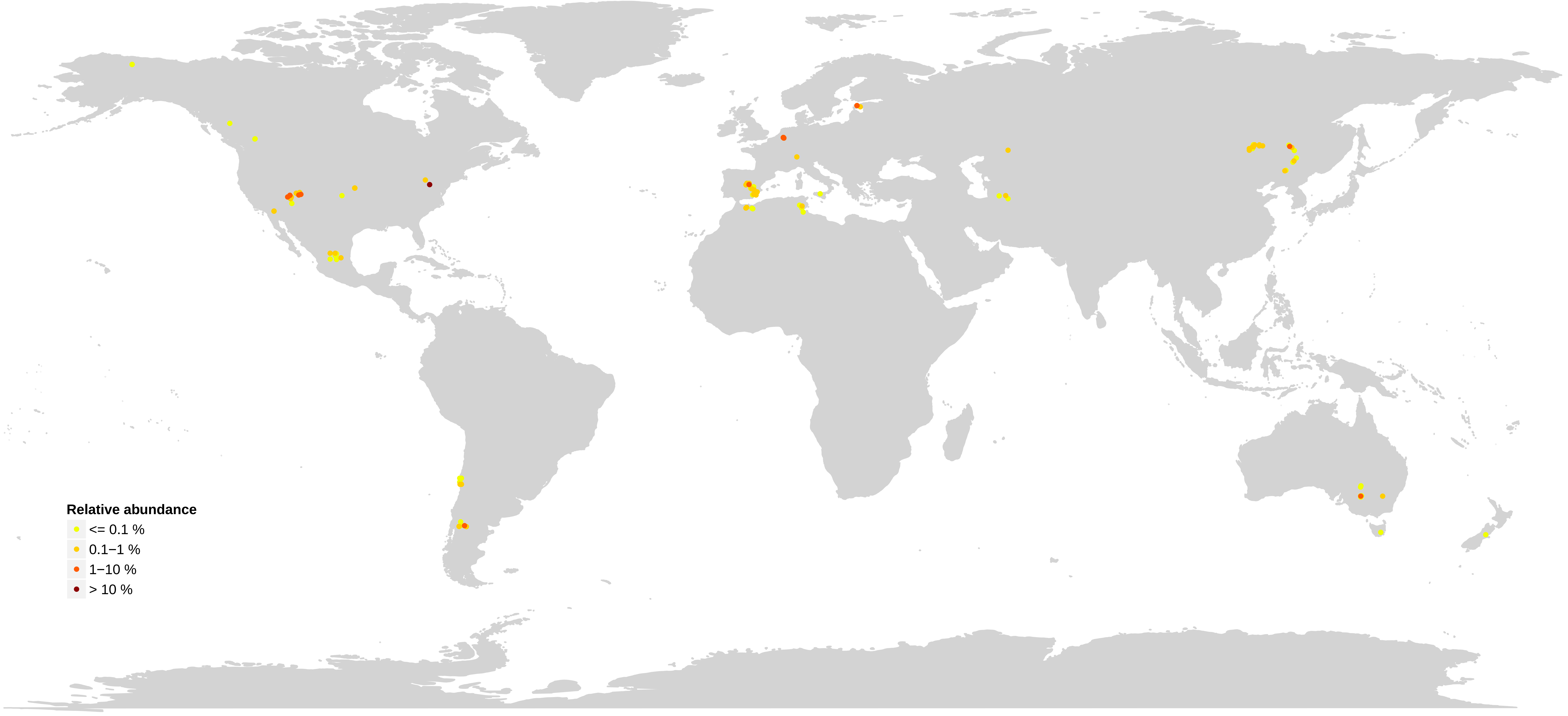

SH216098 Umbelopsis changbaiensis

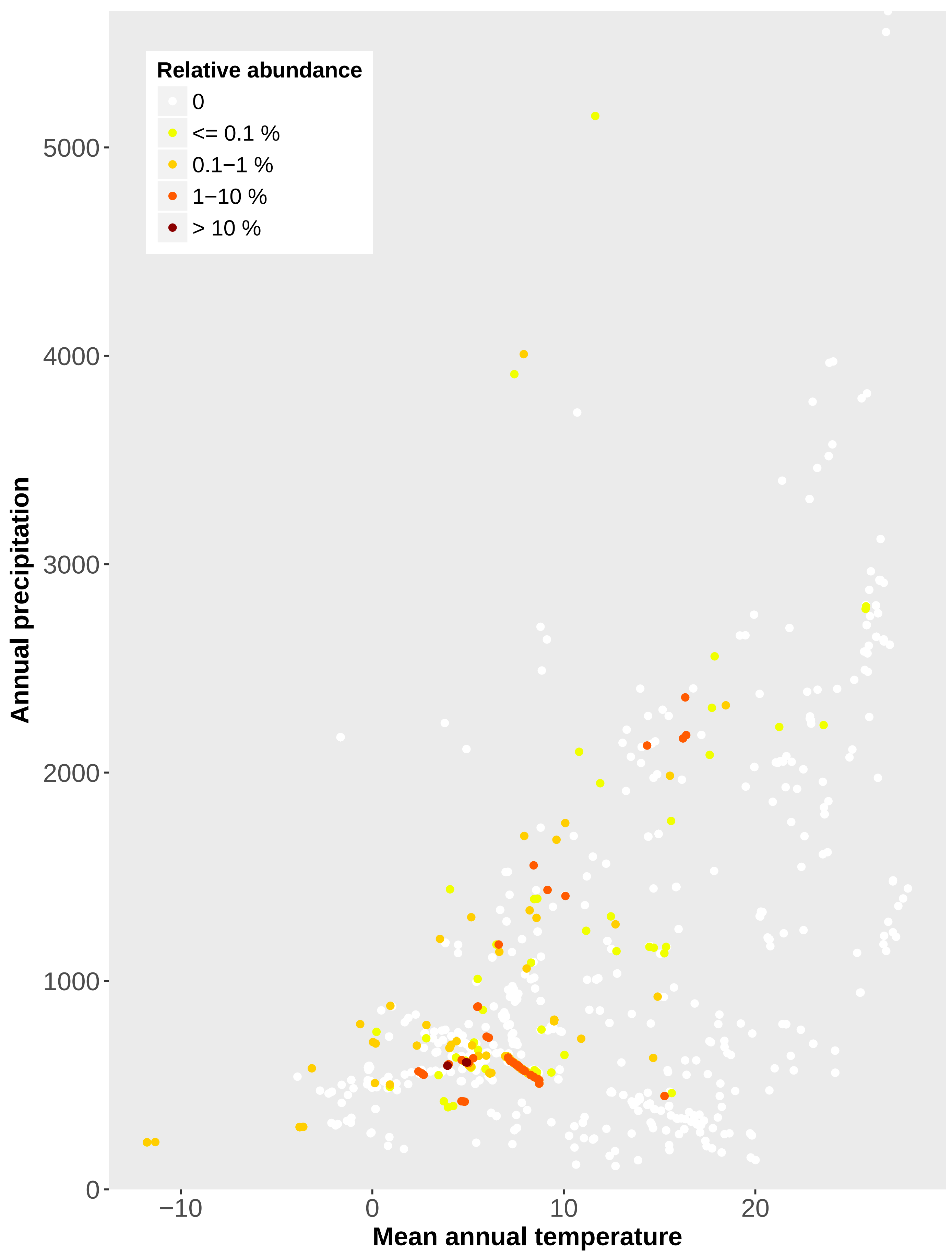

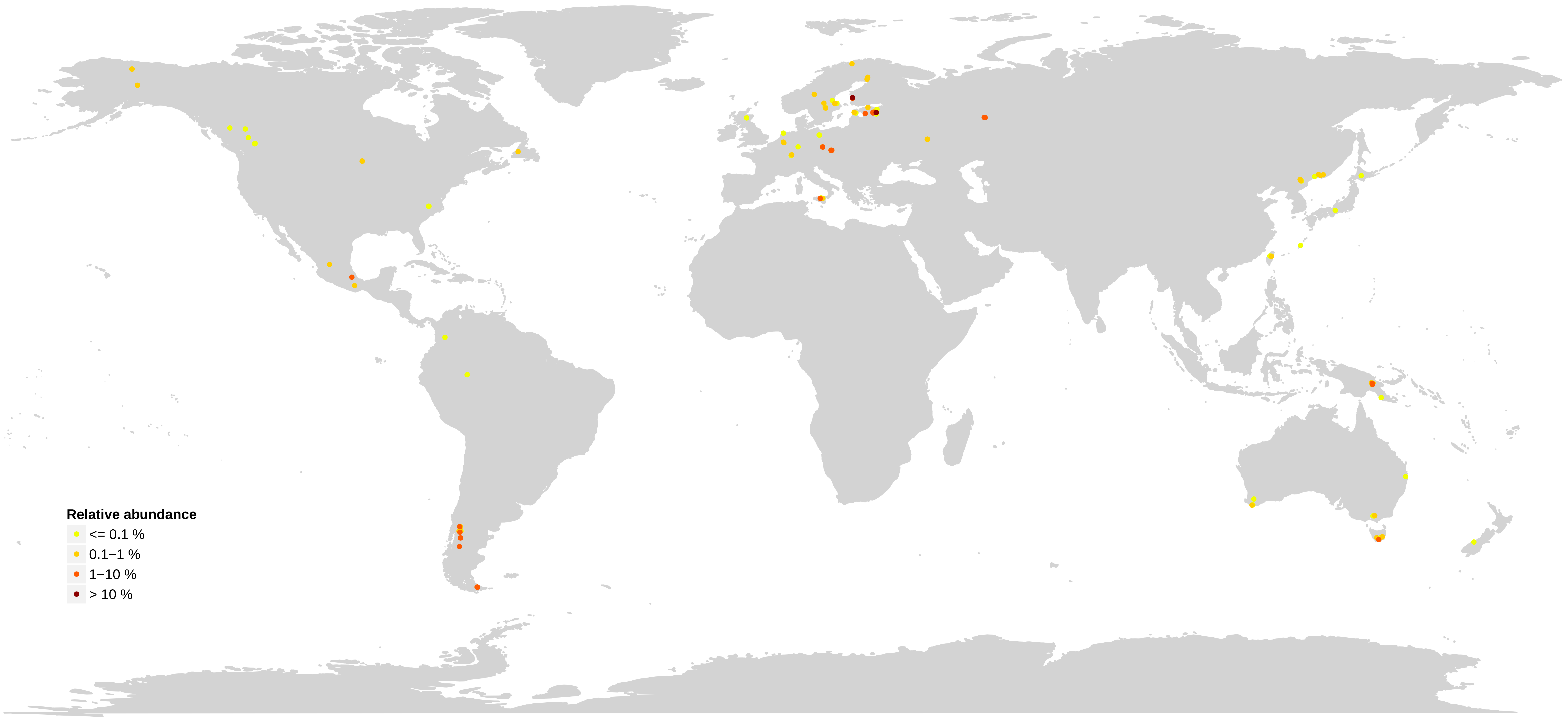

SH219675 Nectriaceae sp

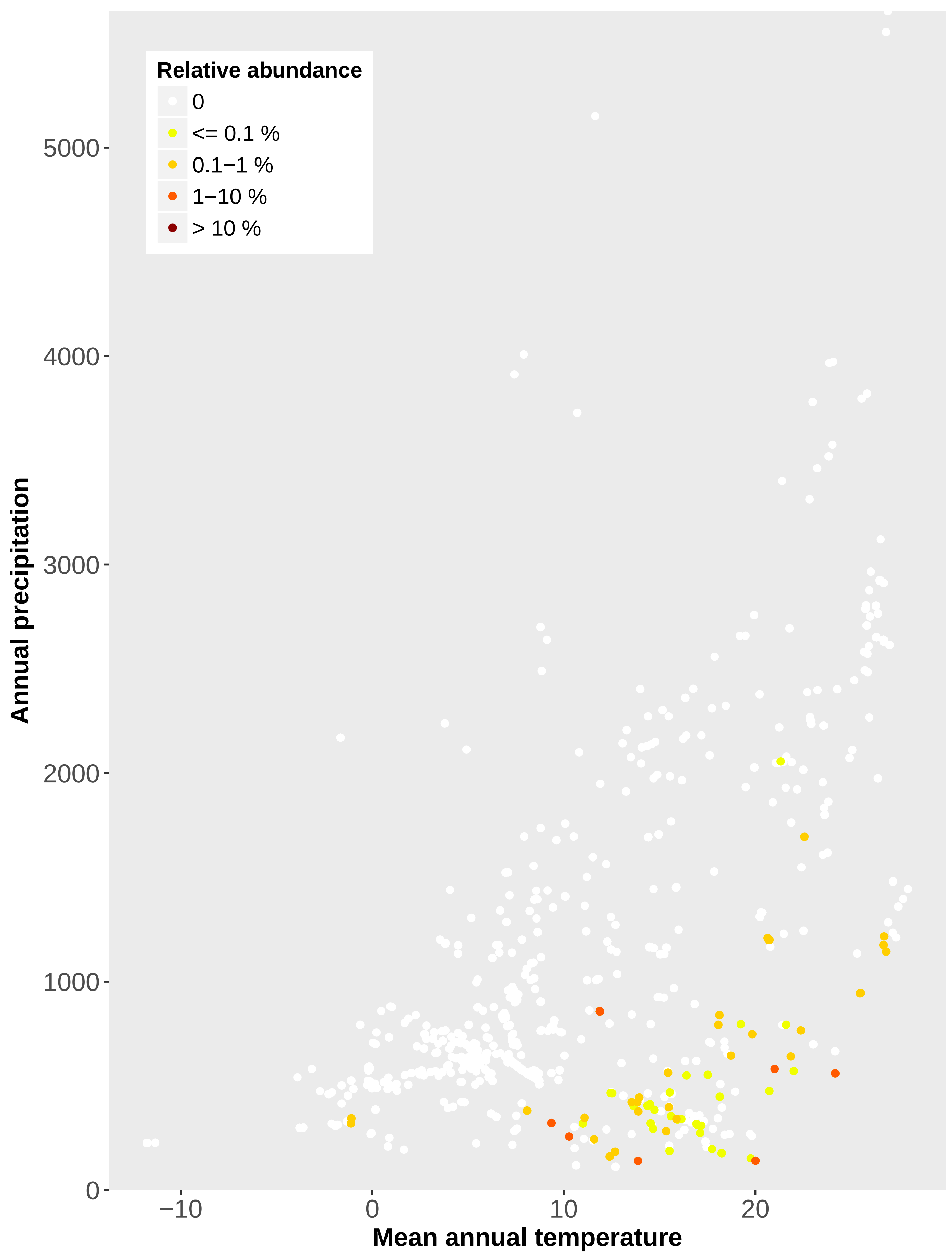

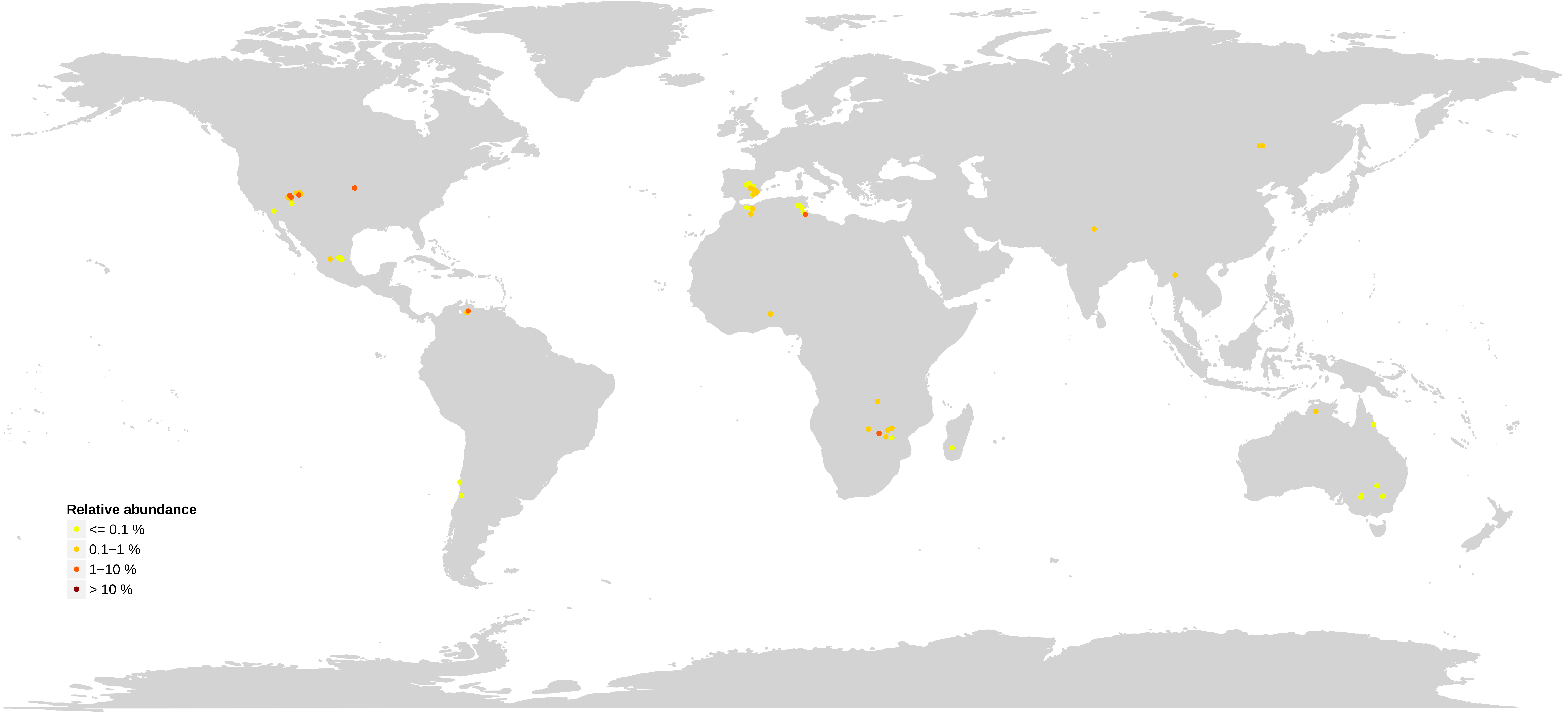

SH379501 Mortierella horticola

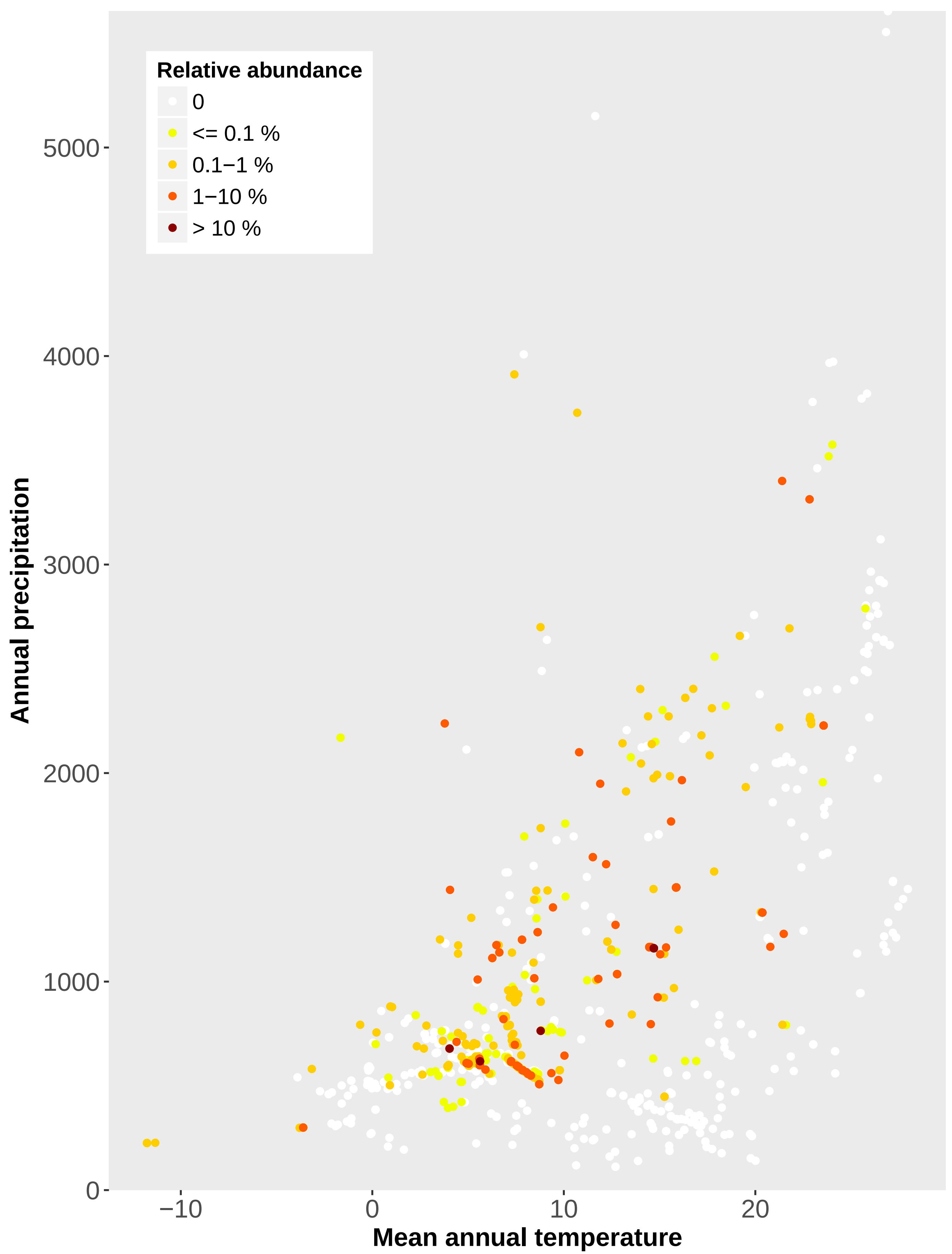

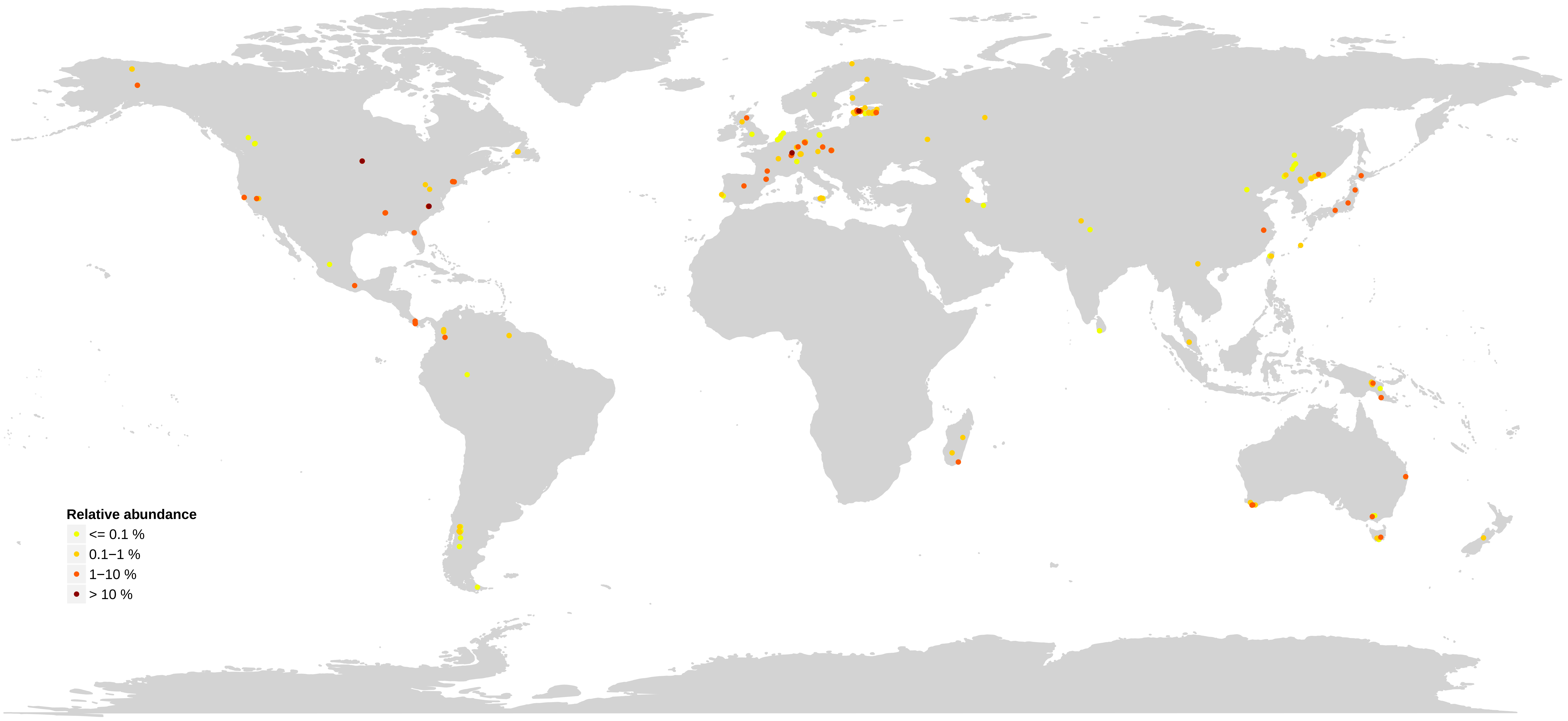

SH245187 Phoma huancayensis

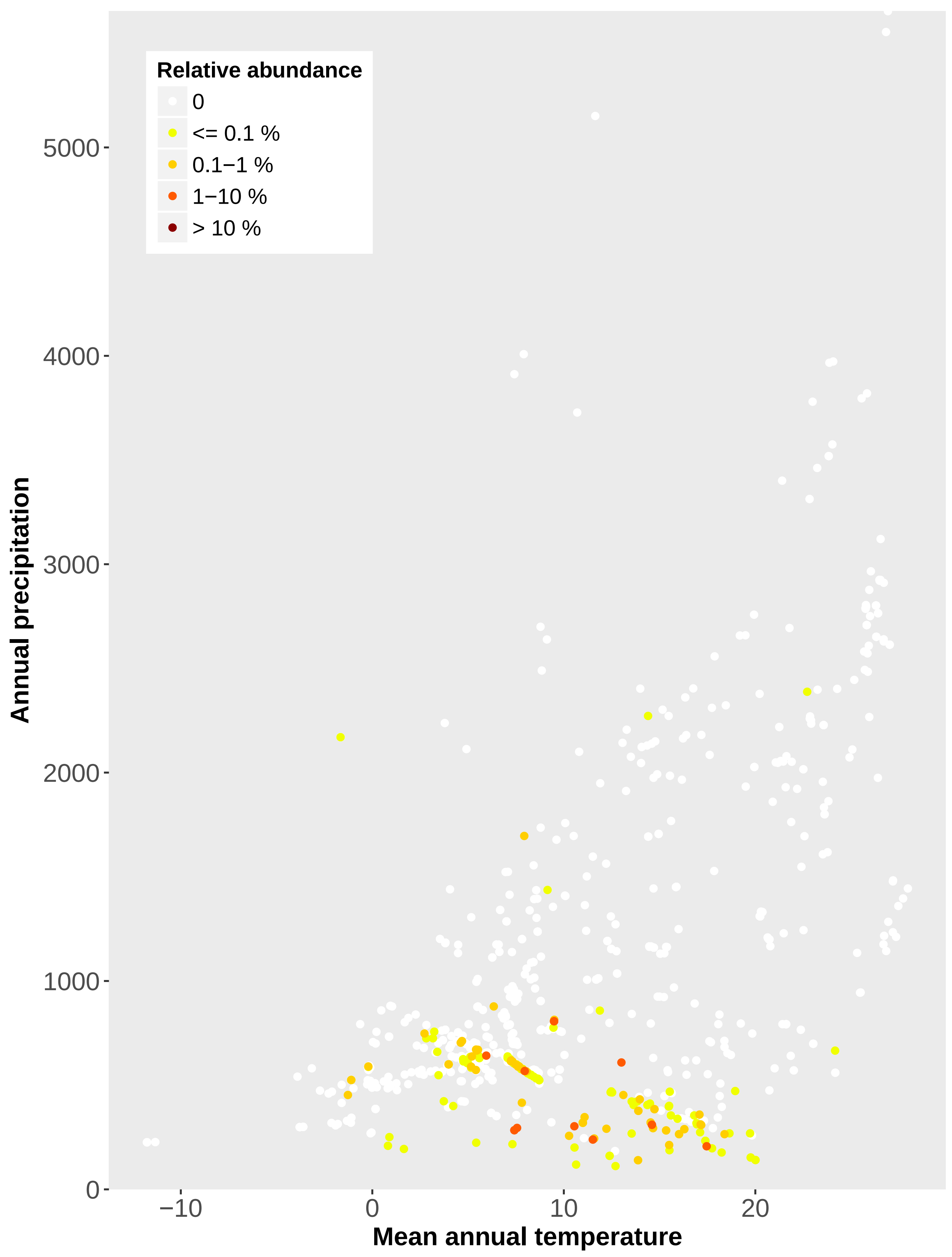

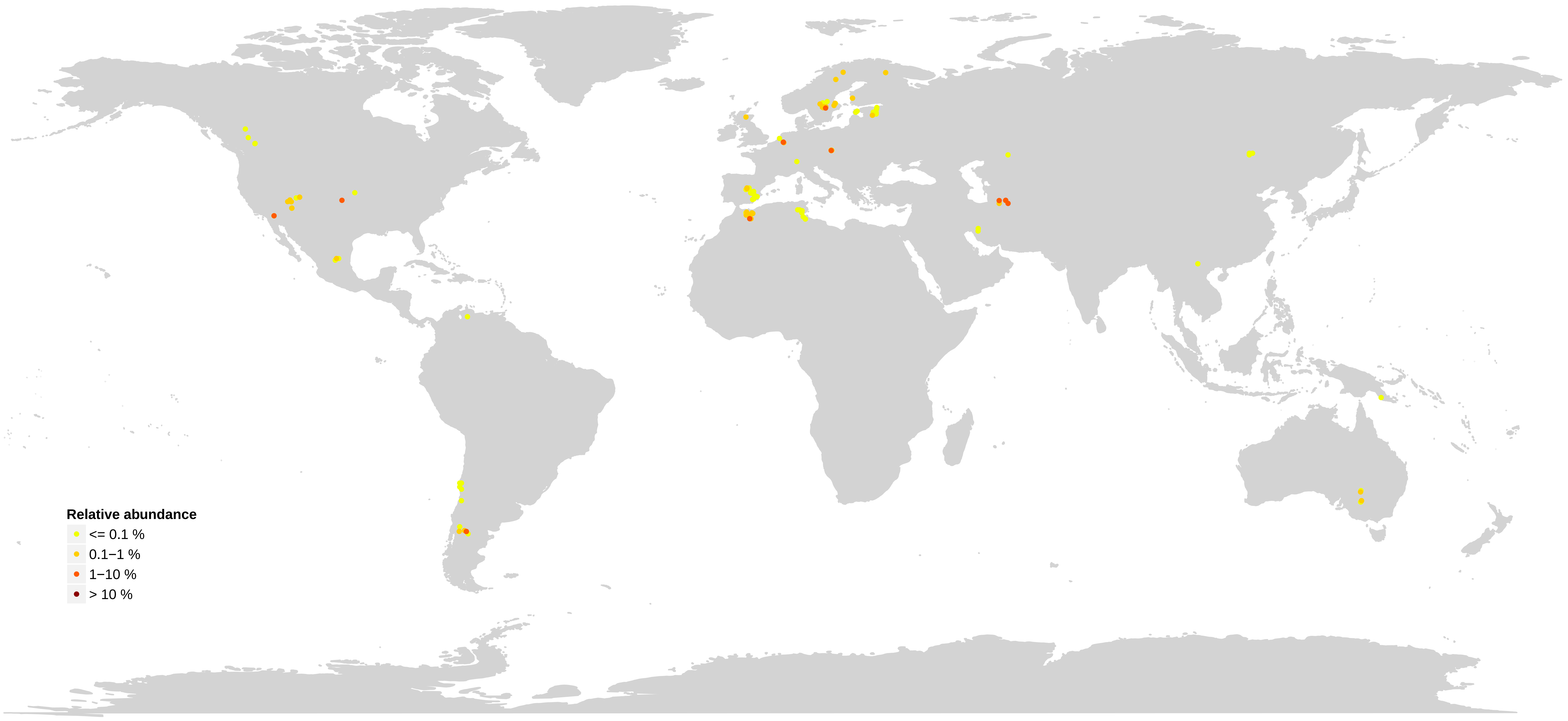

SH208582 Chaetothyriales sp

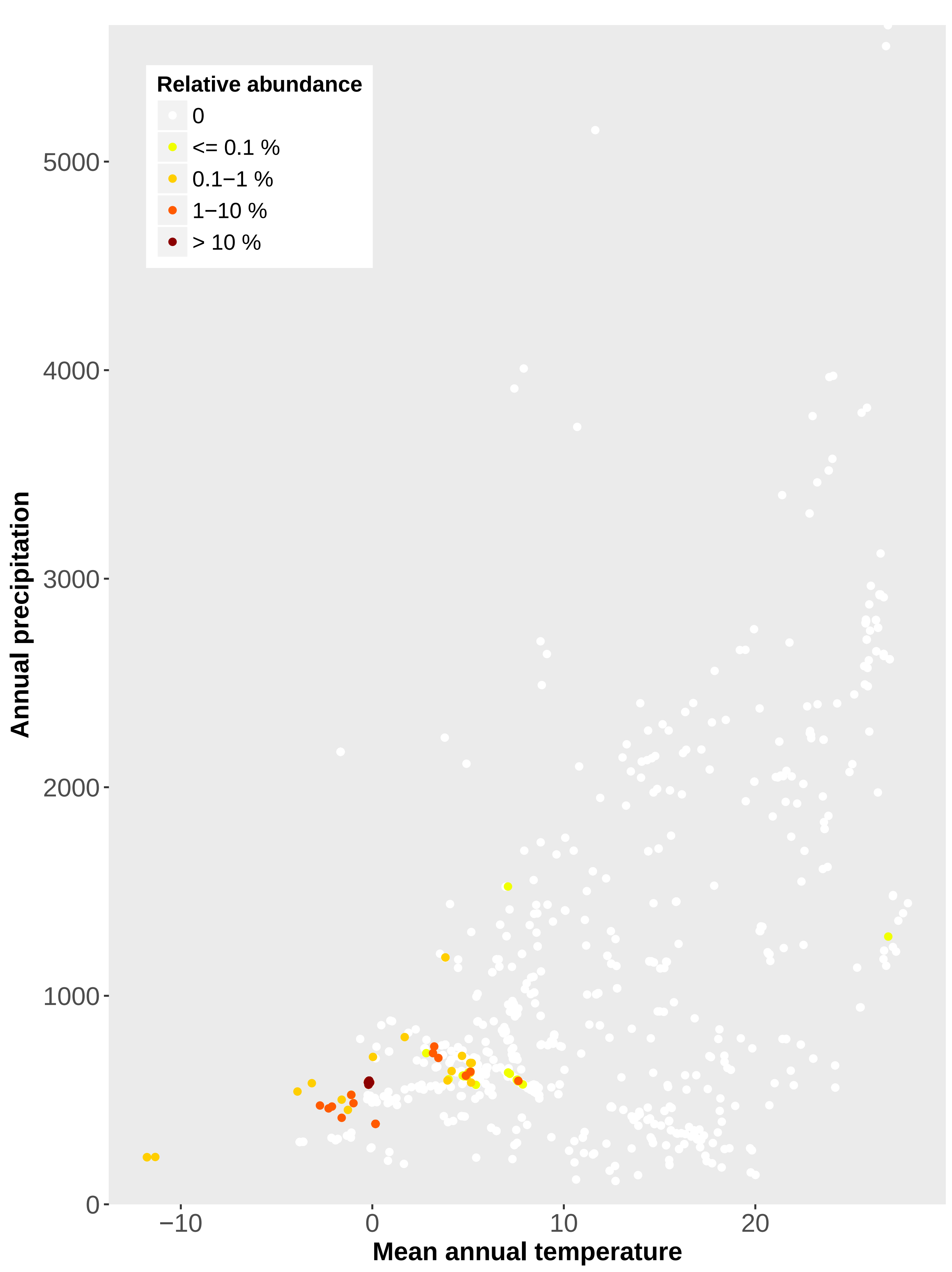

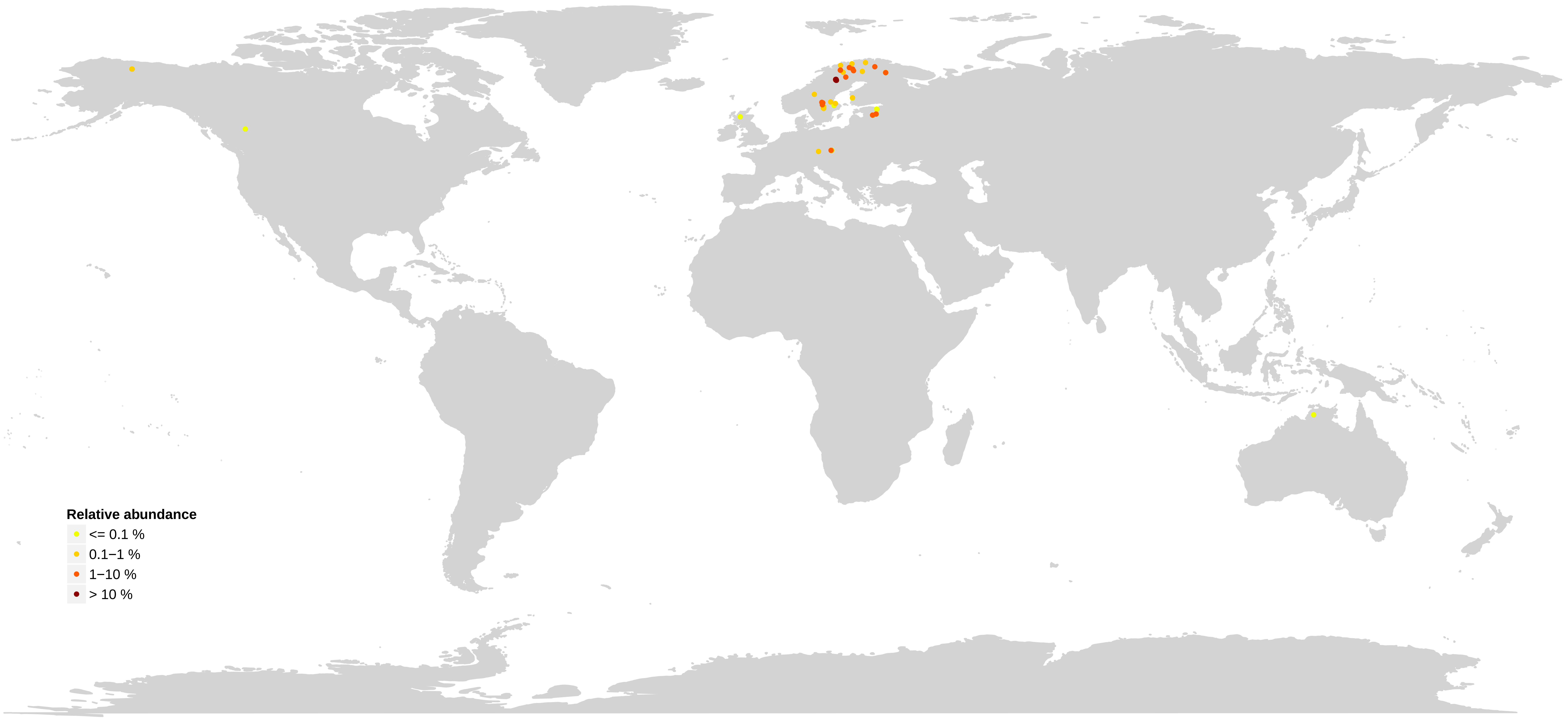

SH497382 Phaeosphaeriaceae sp

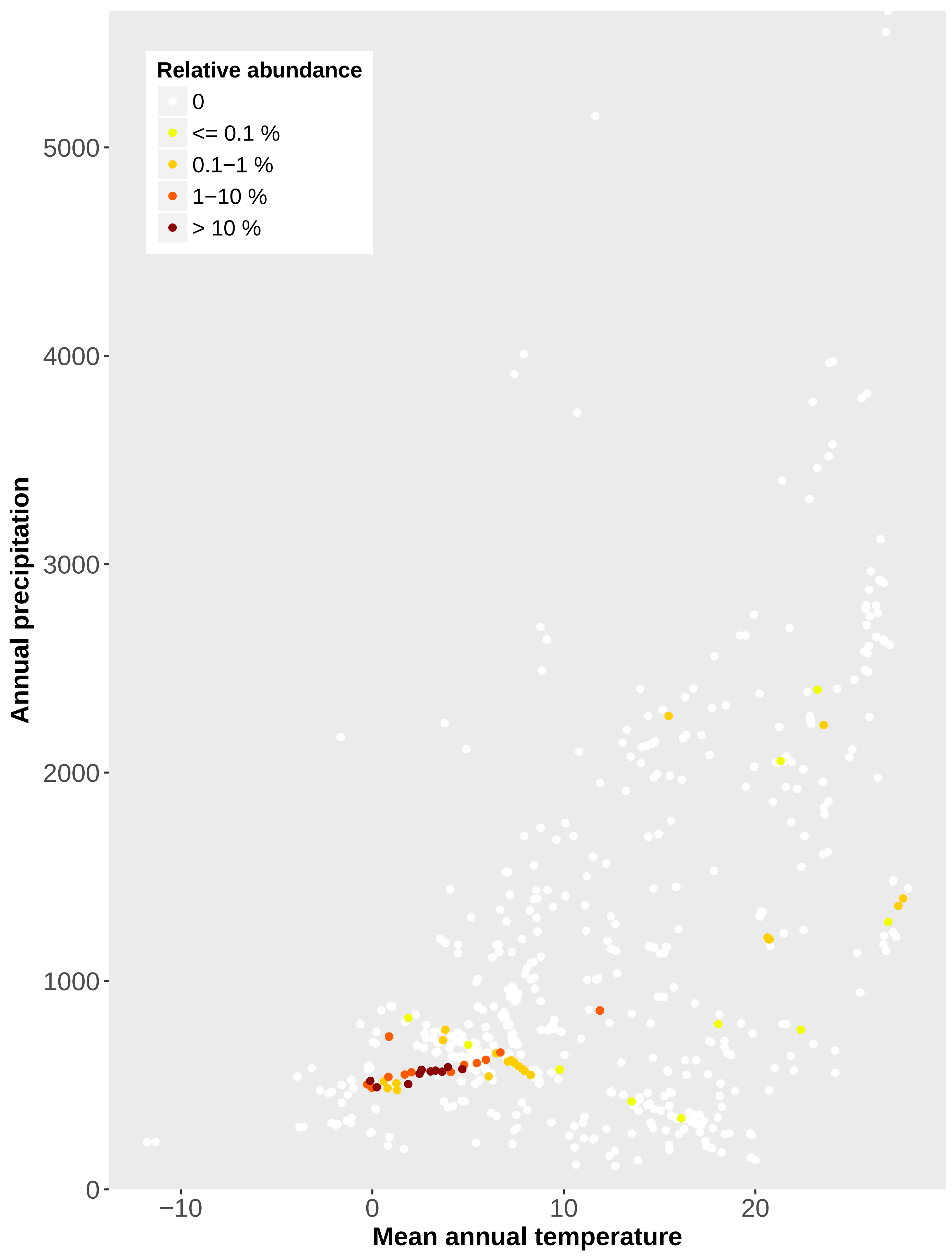

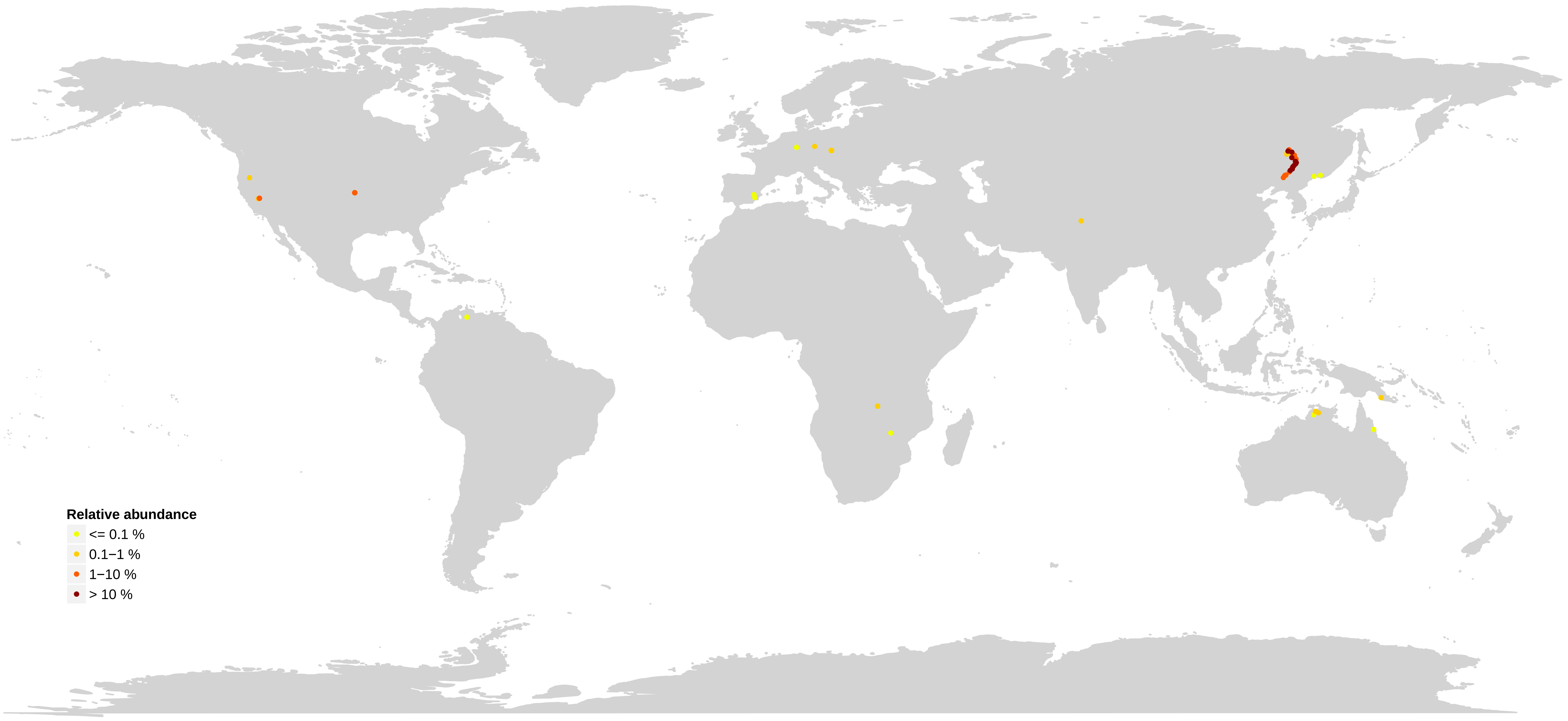

SH523871 Darksidea alpha

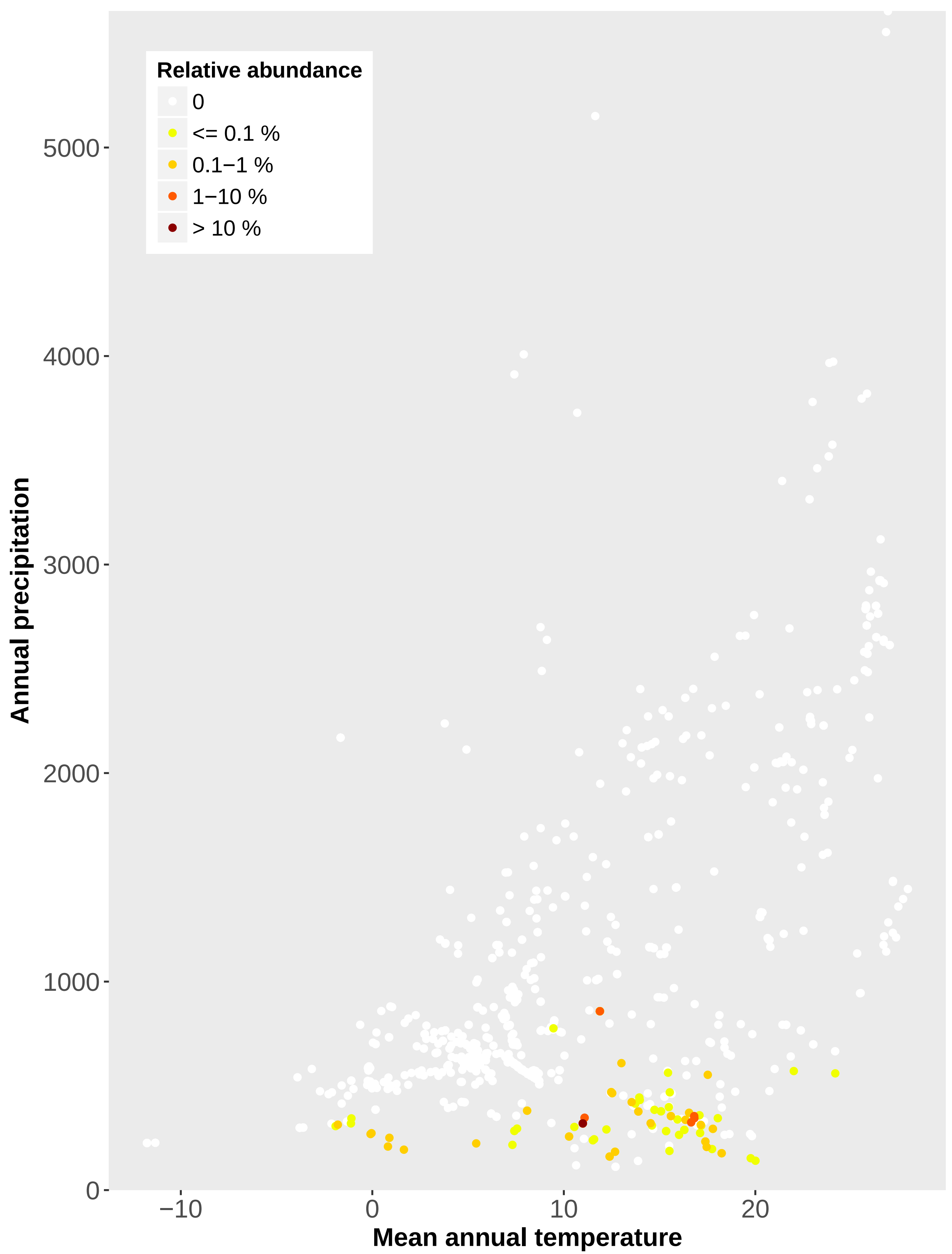

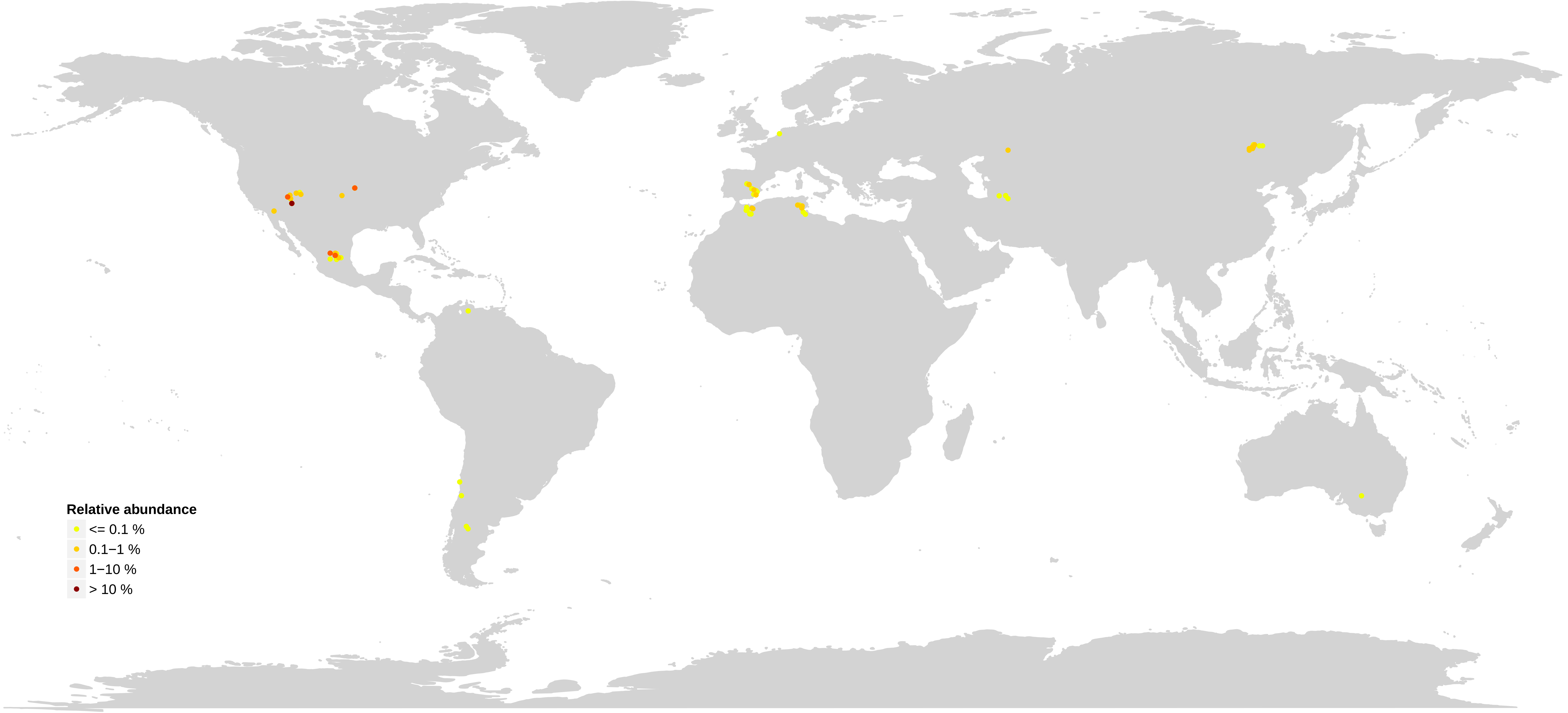

SH198115 Mycoarthris corallina

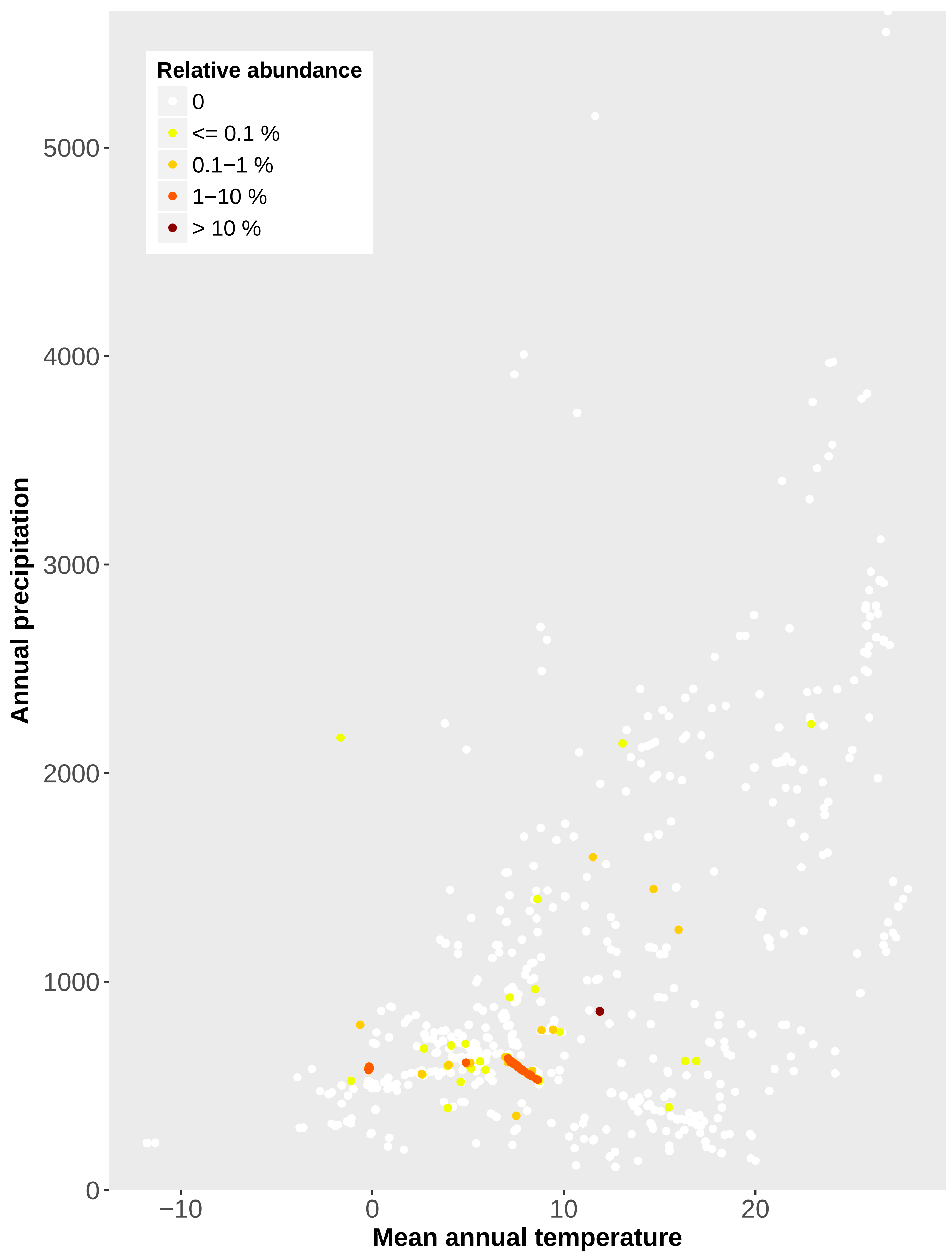

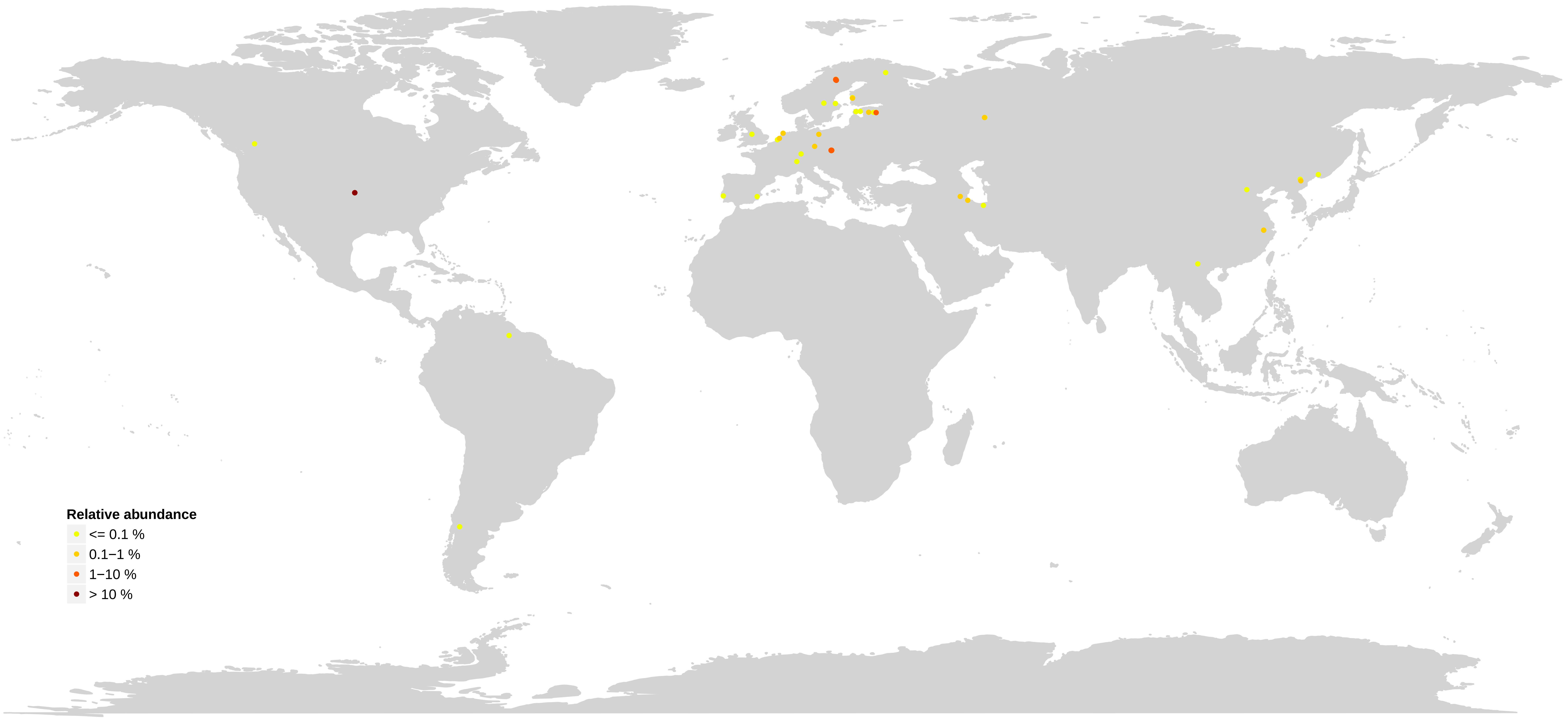

SH196473 Saccharomycetales sp

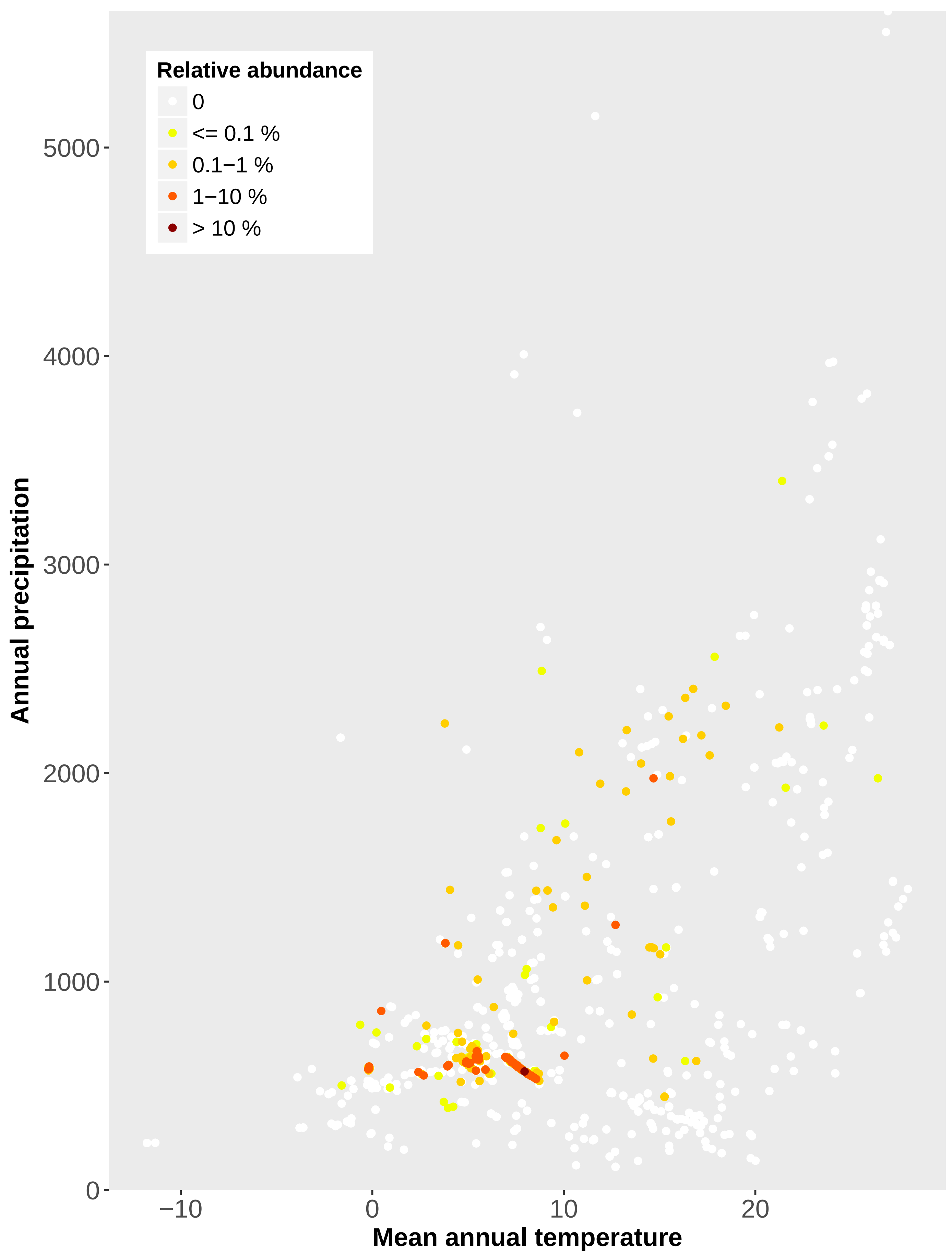

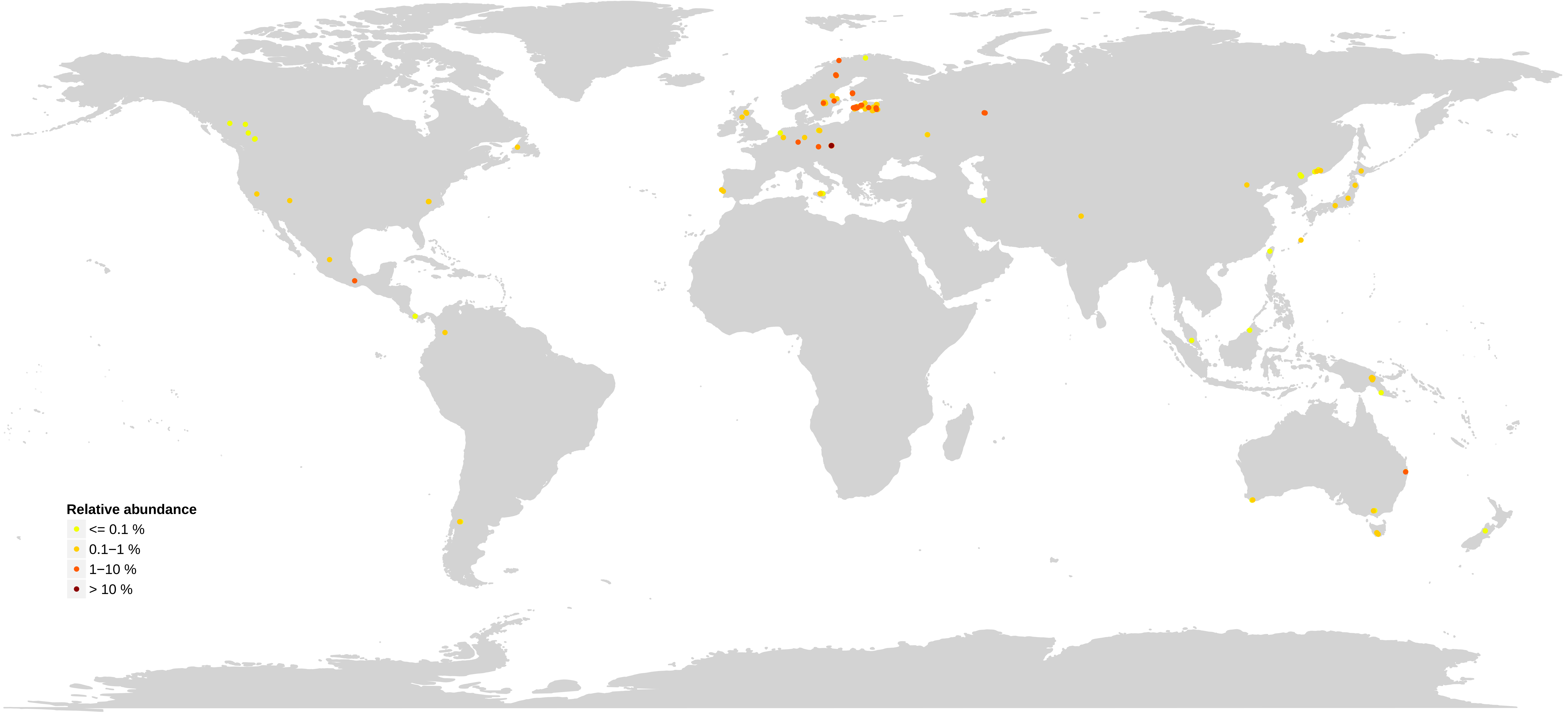

SH205223 Ascomycota sp

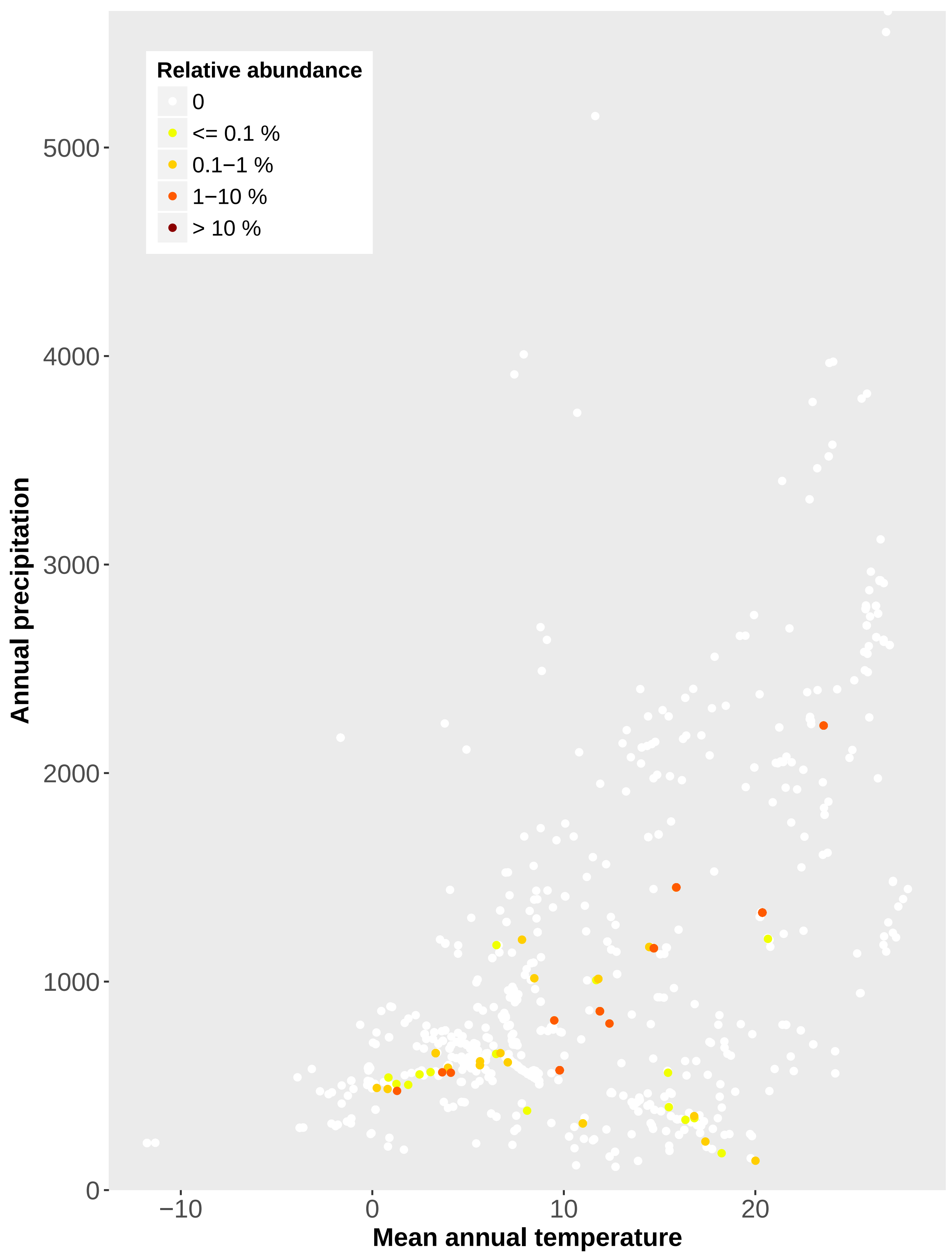

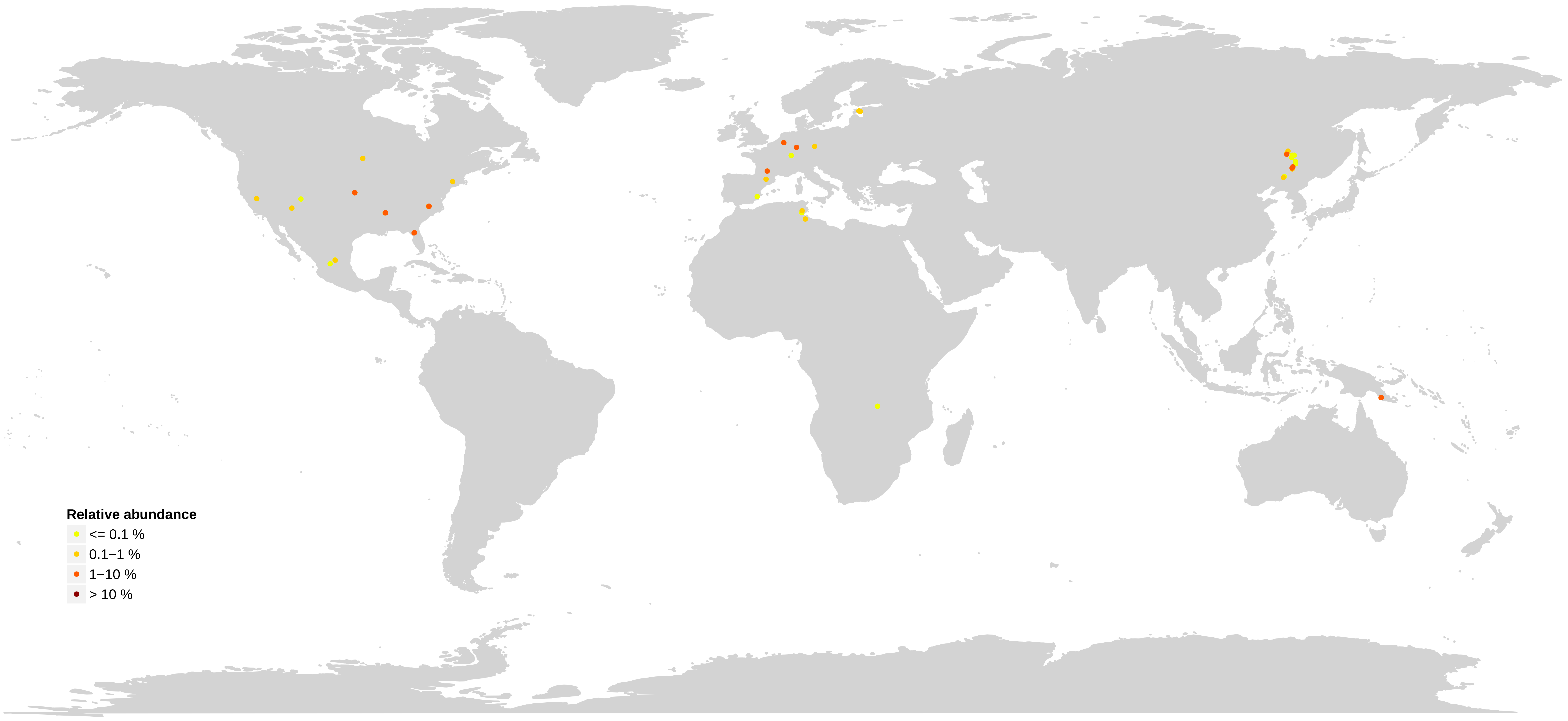

SH206666 Adisciso yakushimense

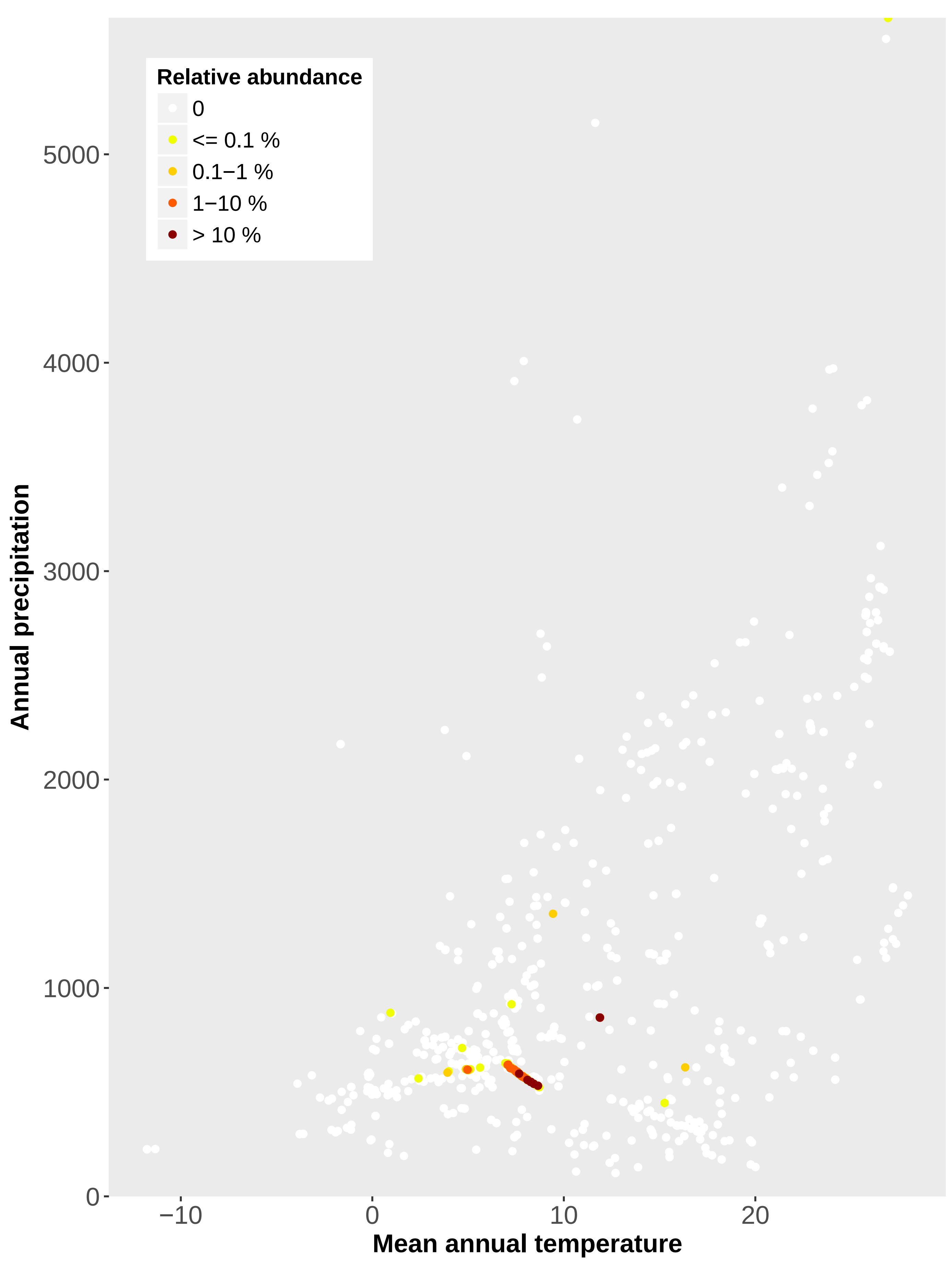

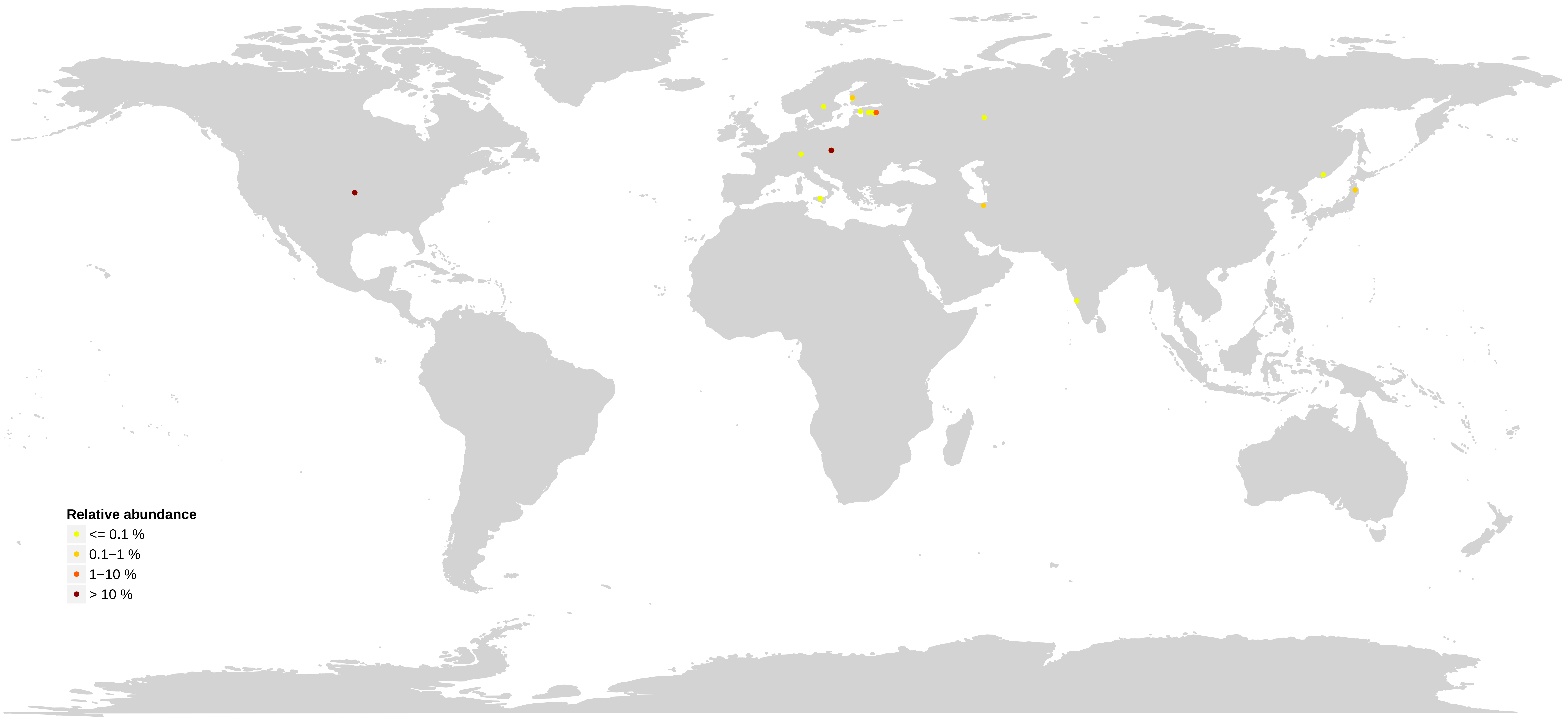

SH208578 Chaetothyriales sp

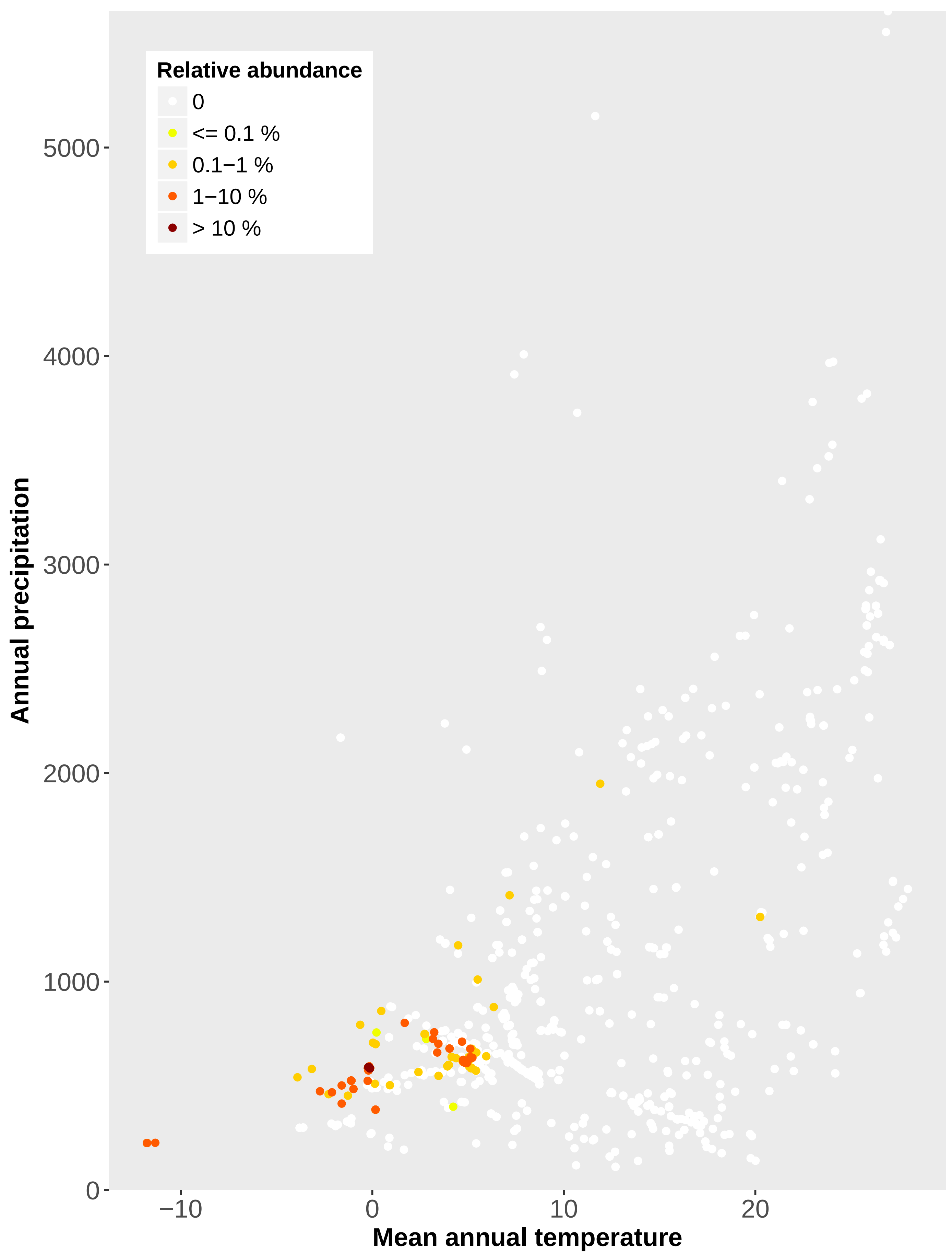

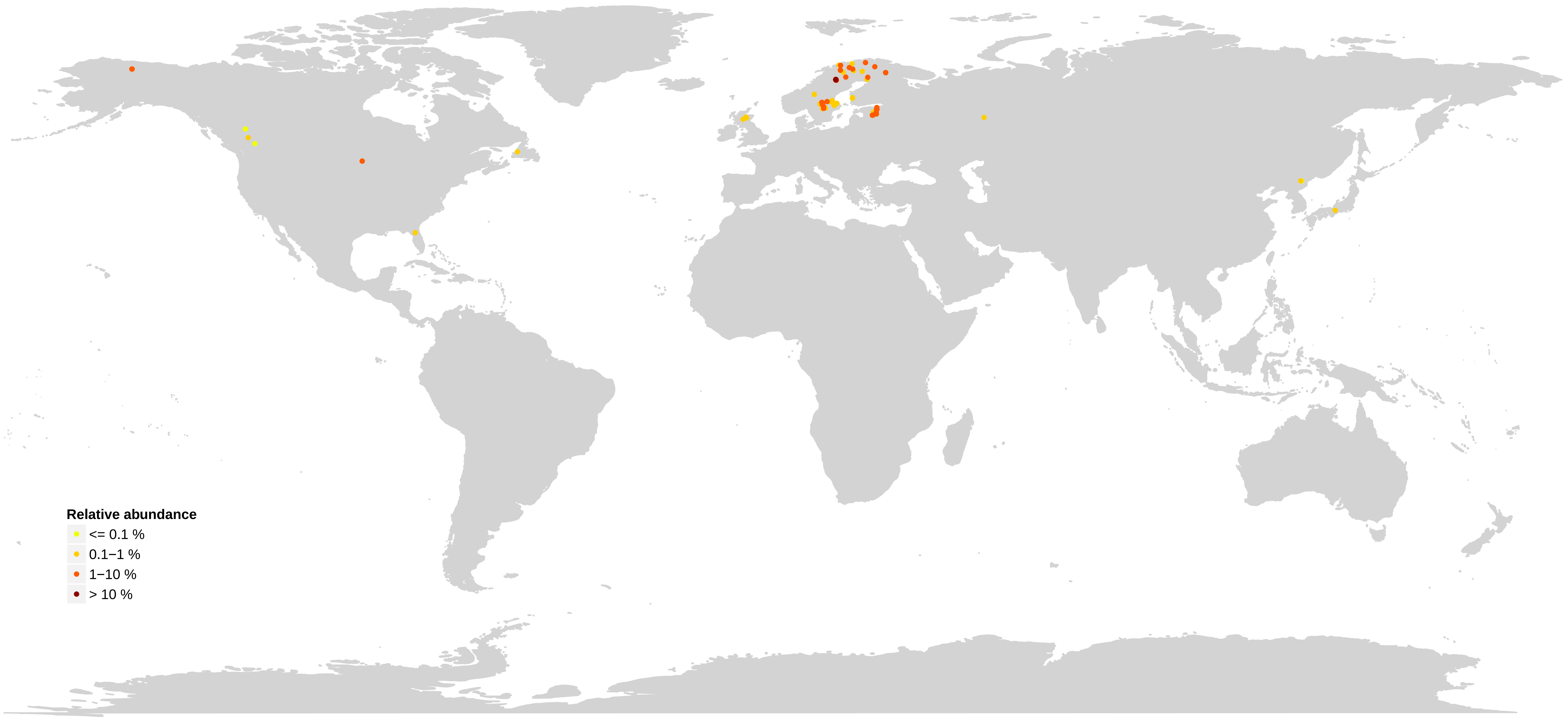

SH023910 Penicillium sp

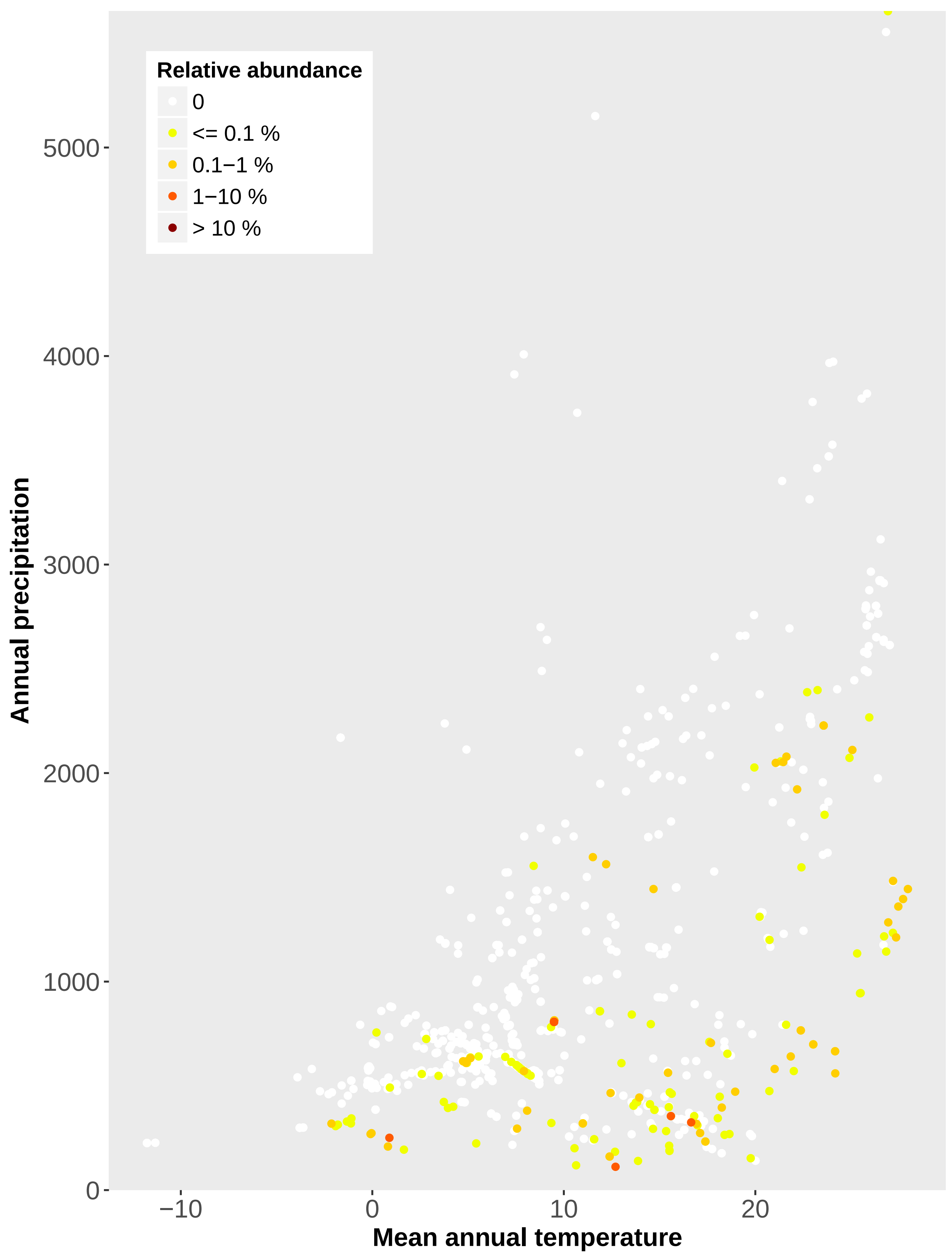

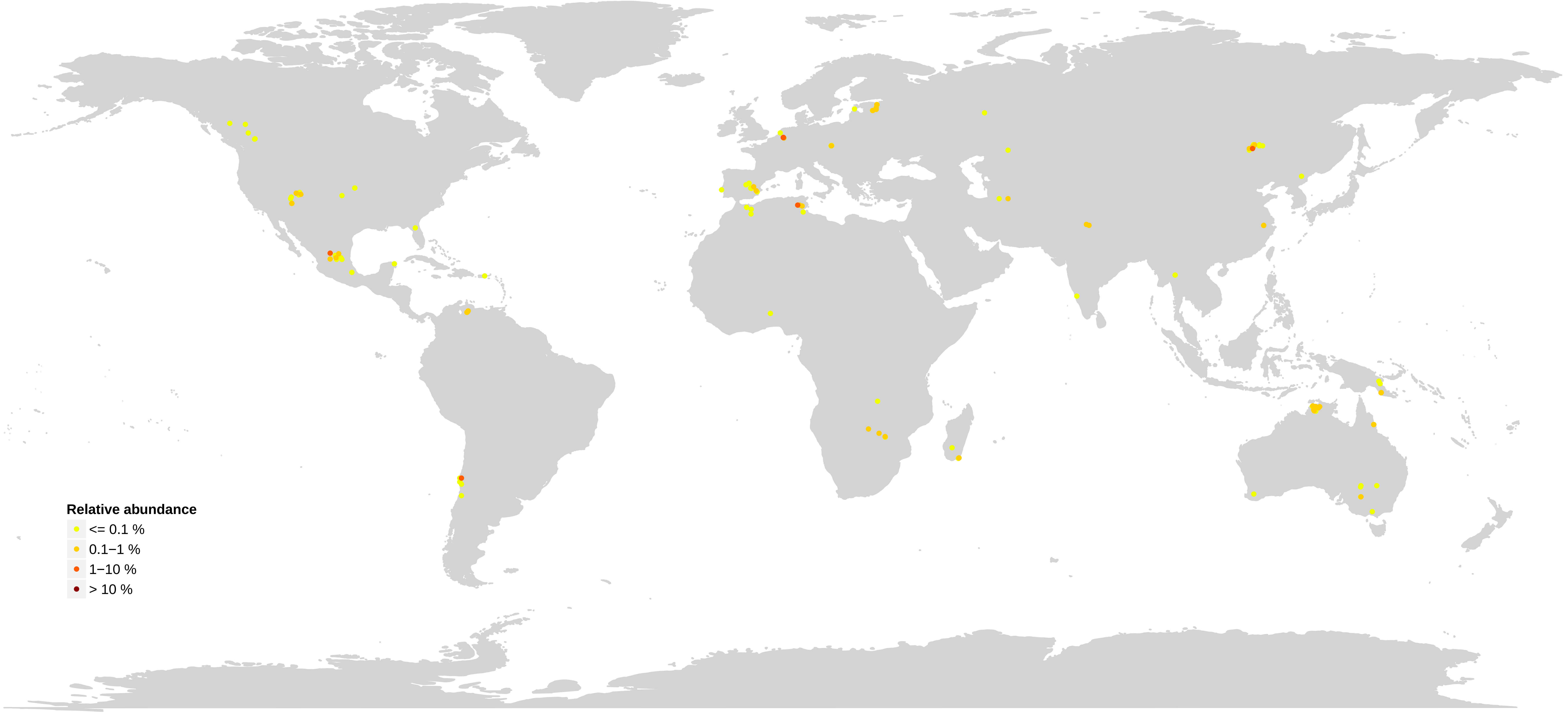

SH182377 Lactarius rufus

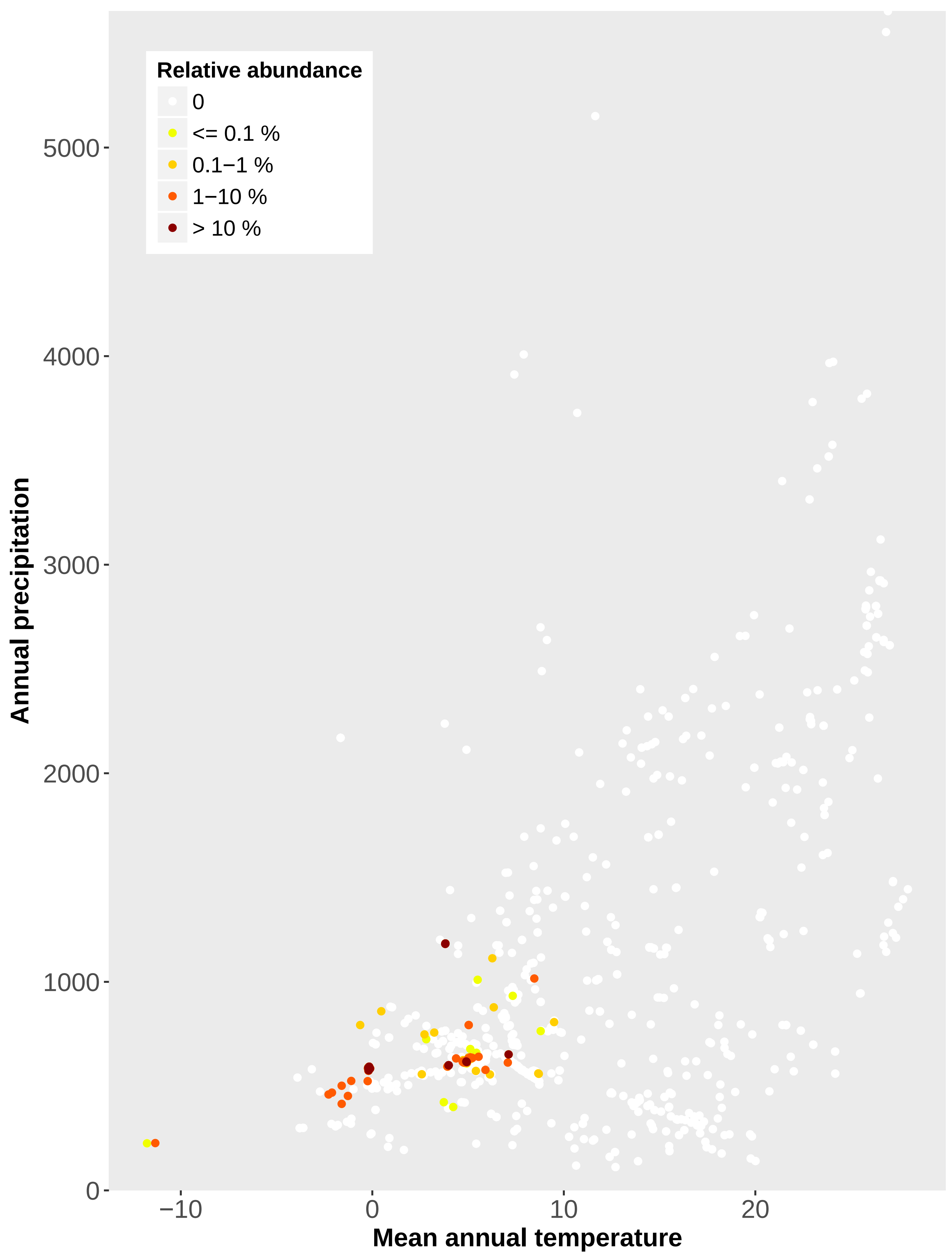

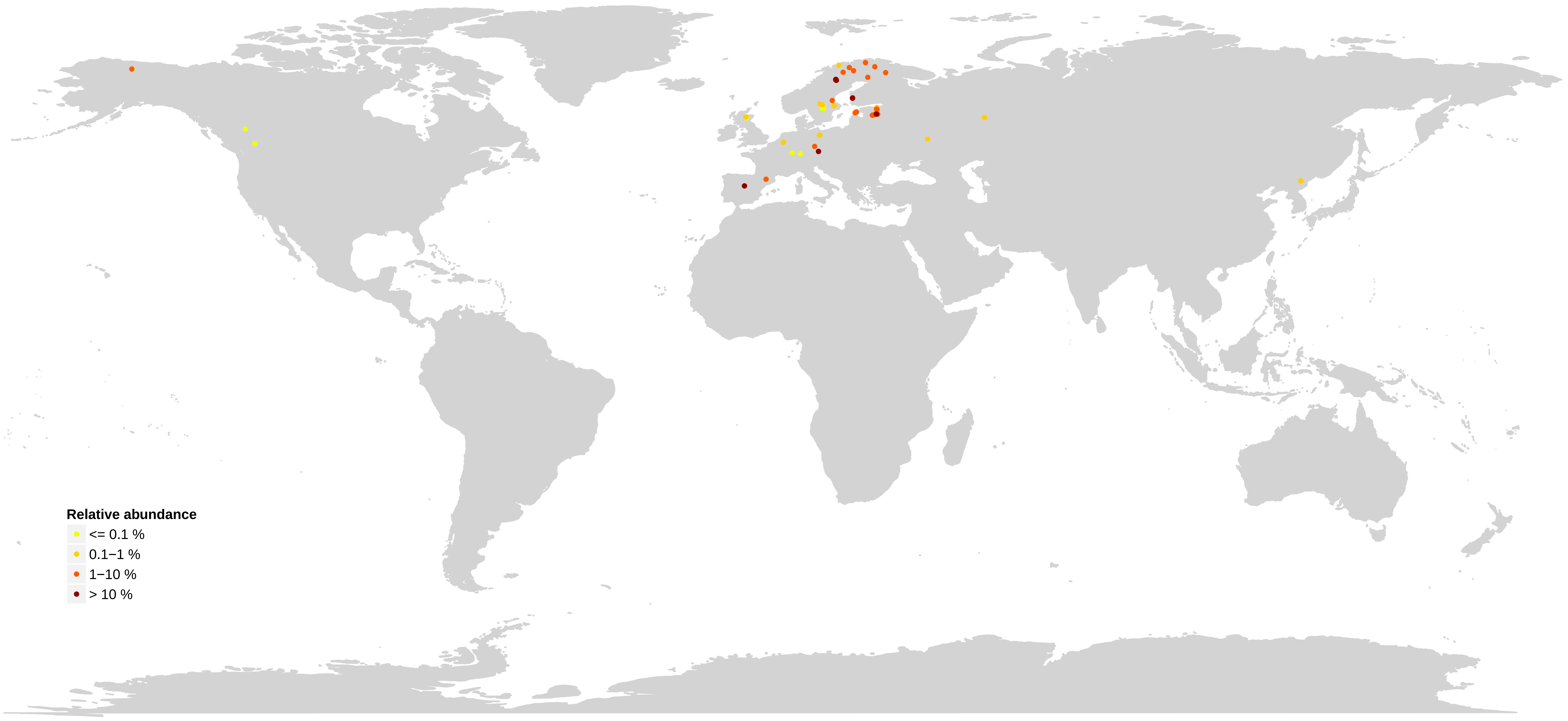

SH214265 Cadophora finlandica

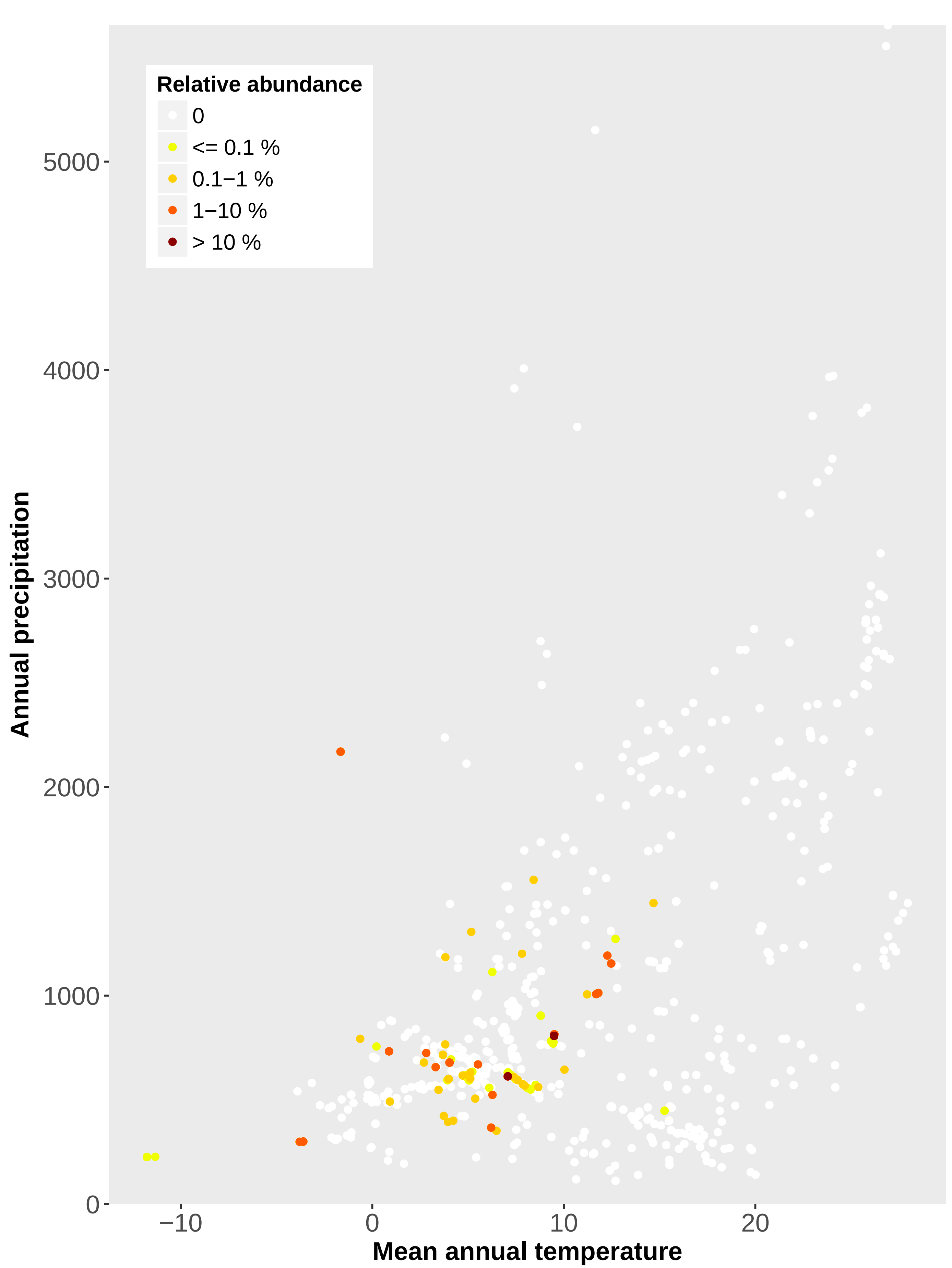

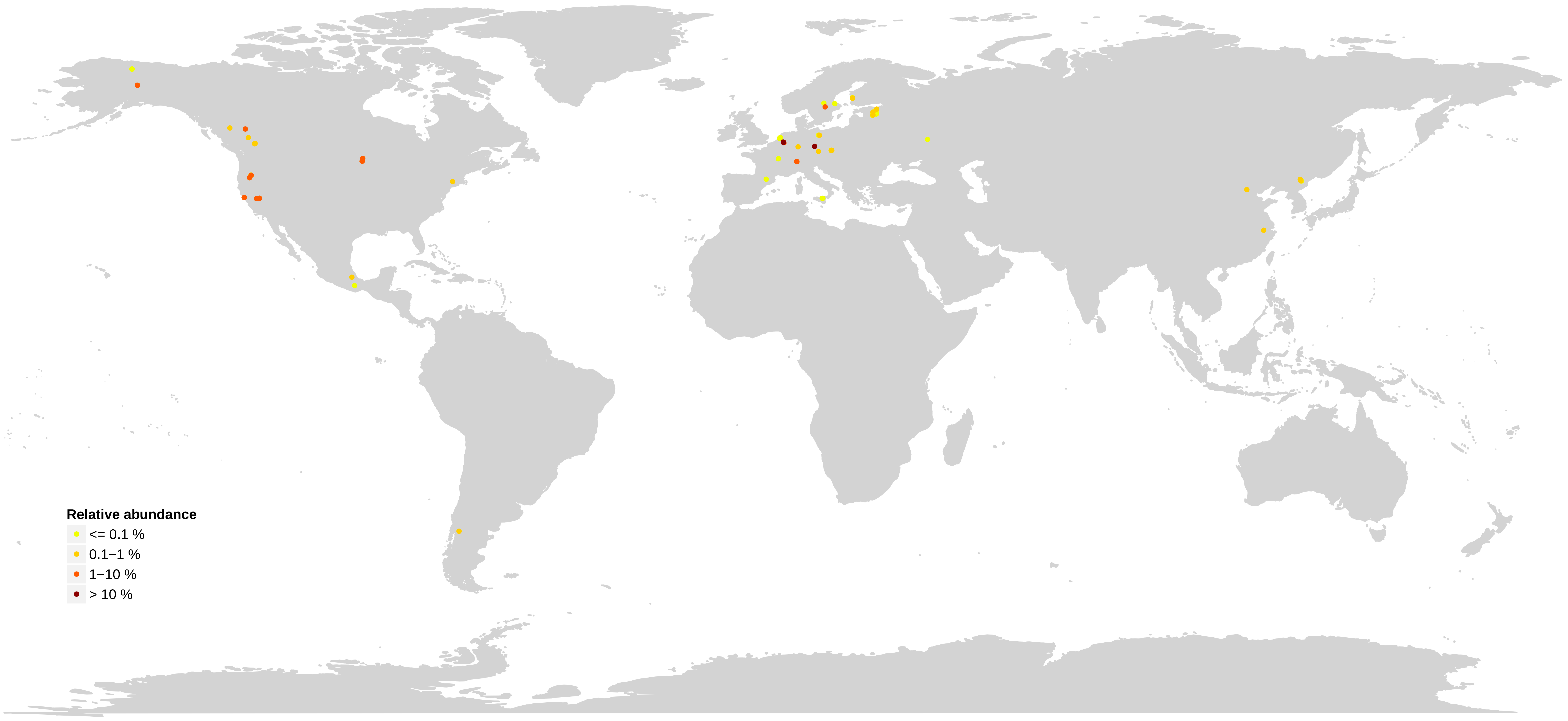

SH194391 Phacidium lacerum

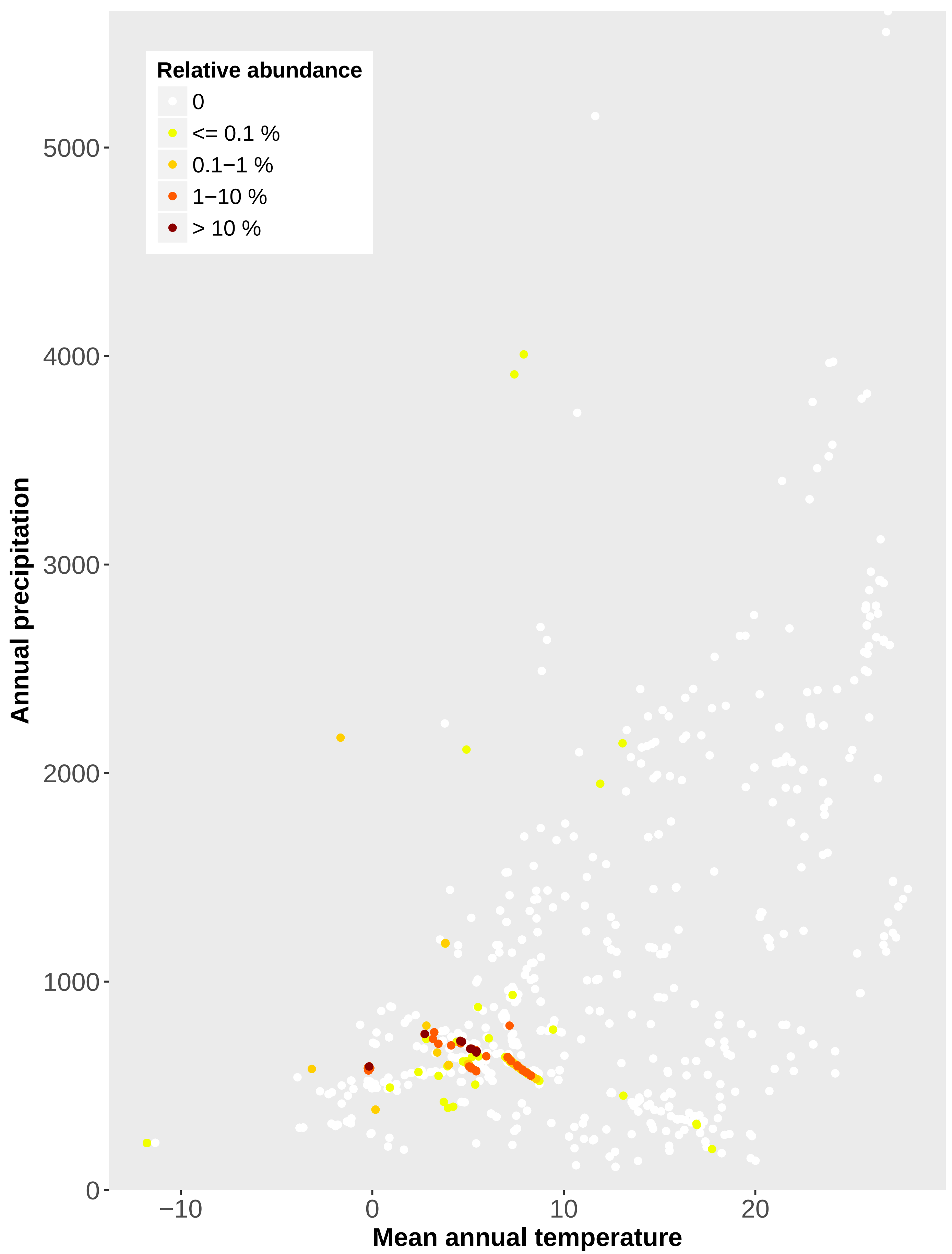

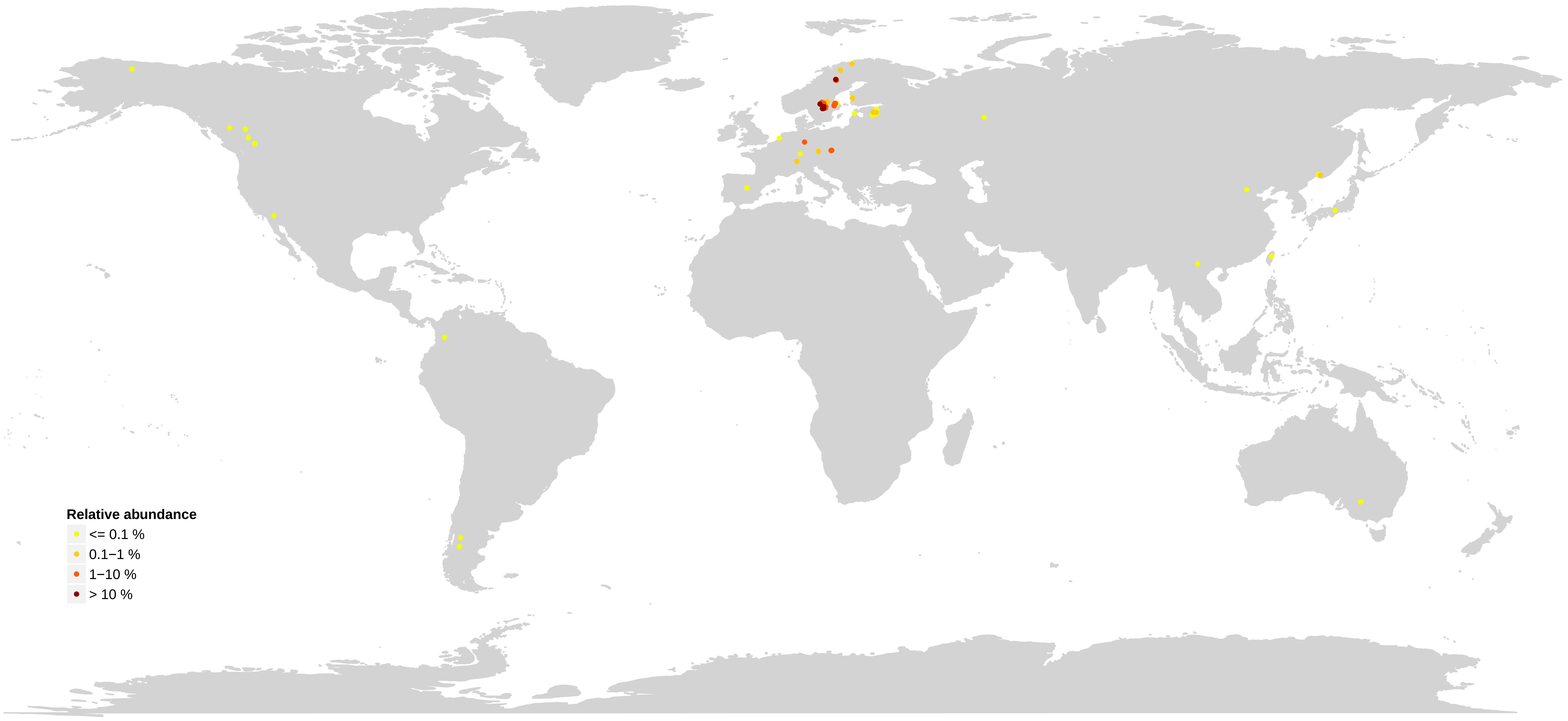

SH021570 Dothideomycetes sp

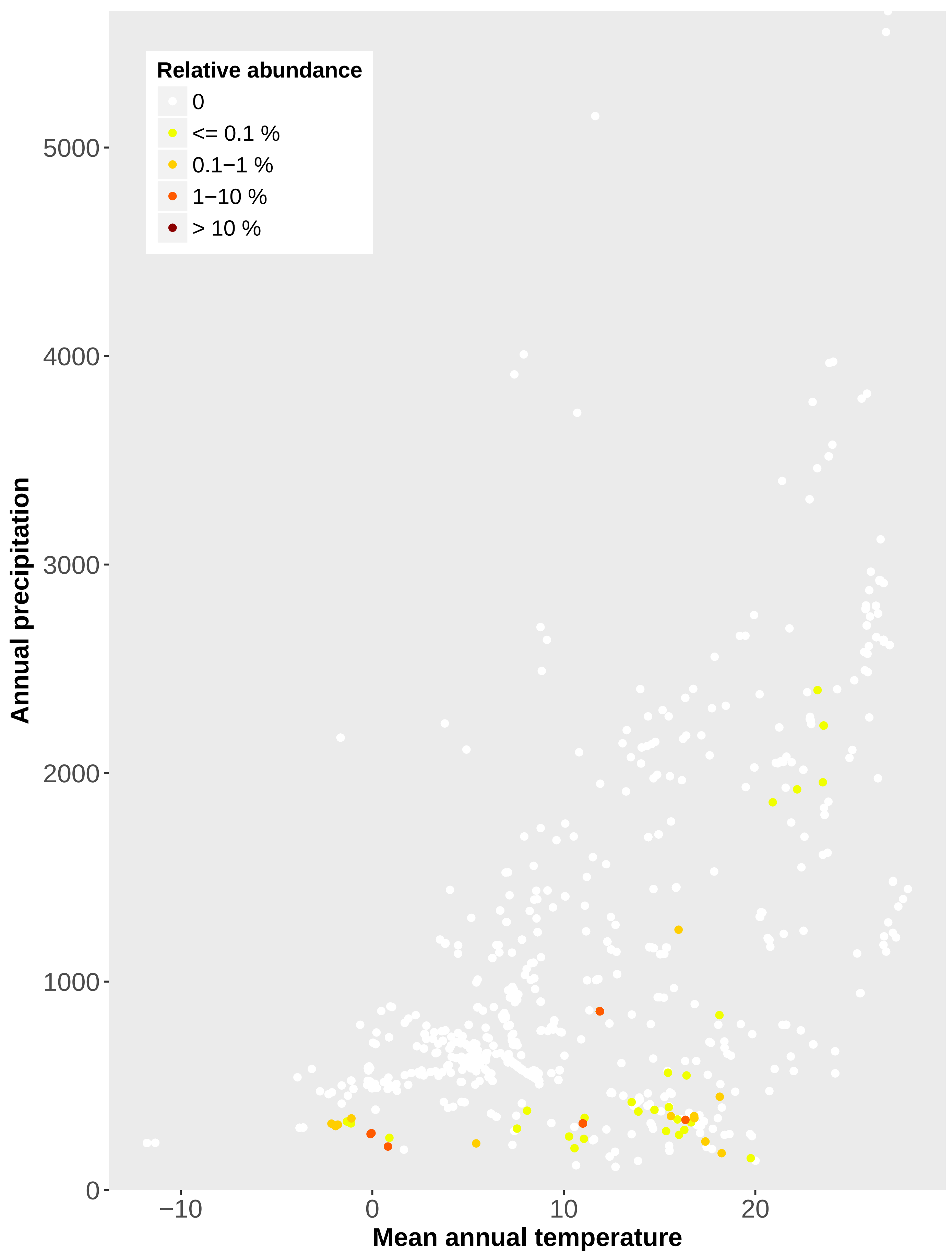

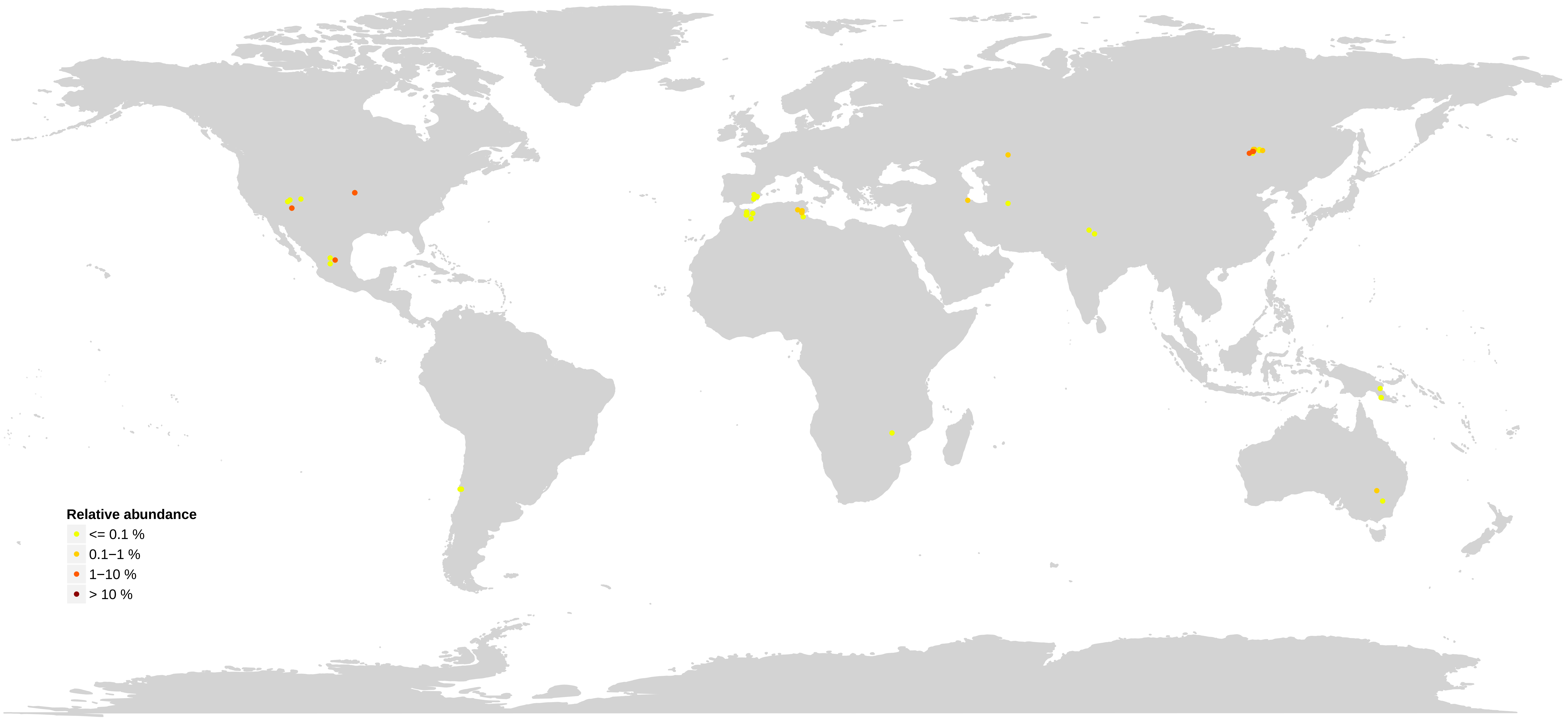

SH195988 Venturiaceae sp

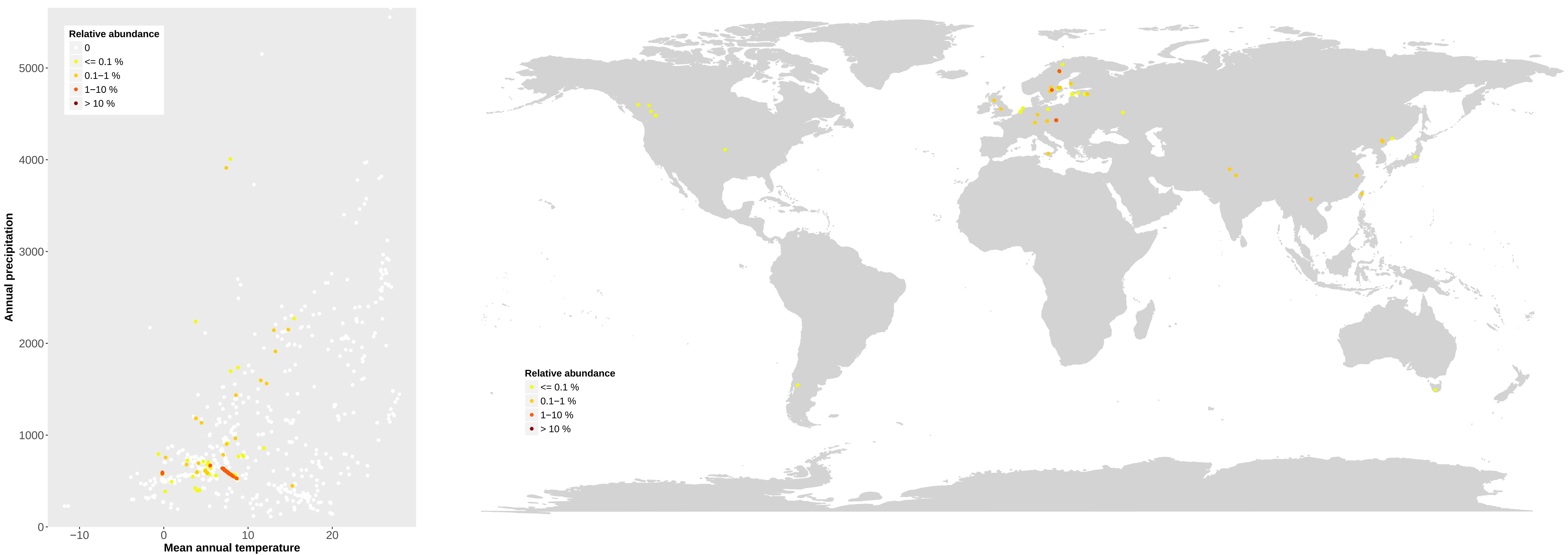

SH220719 Agaricales sp

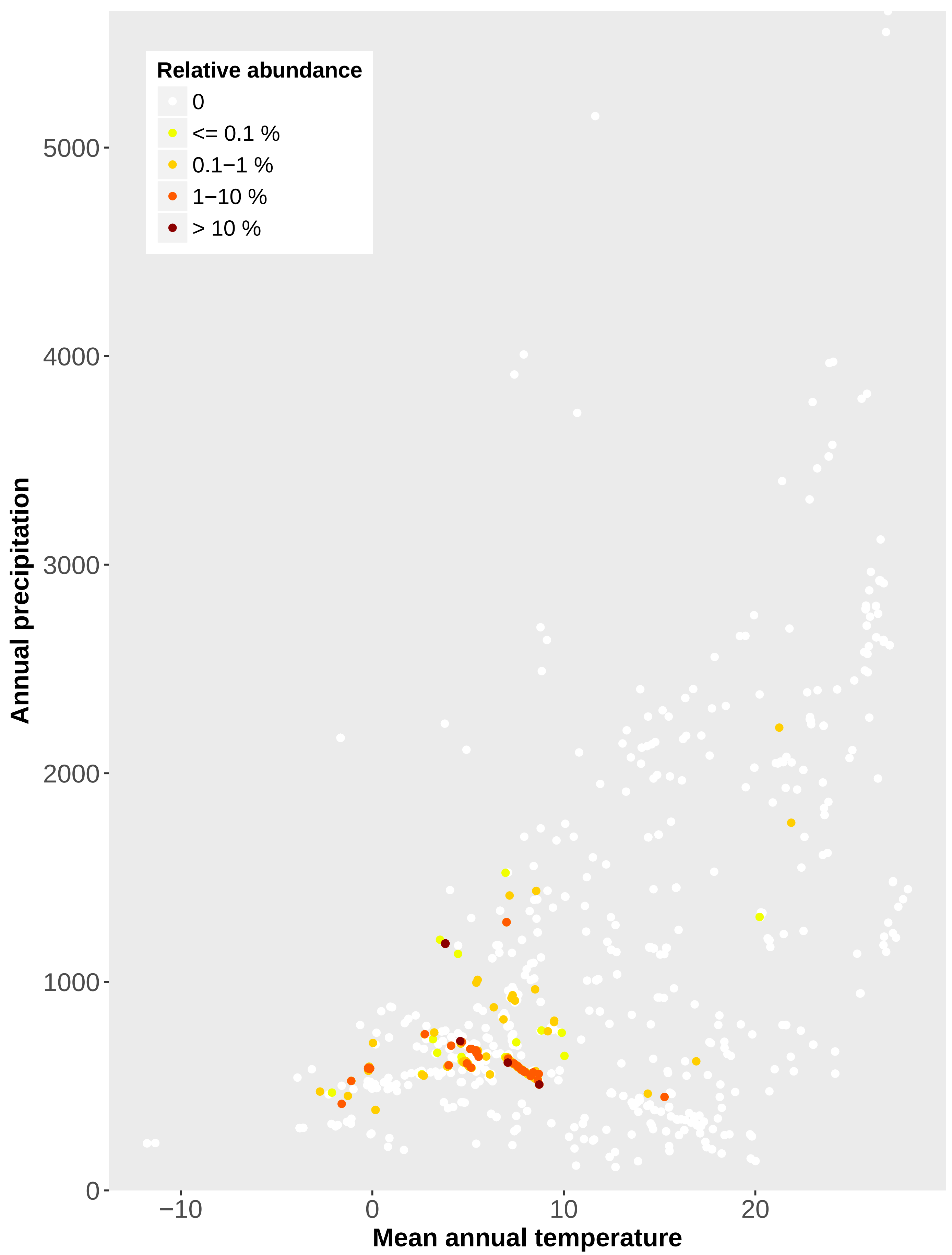

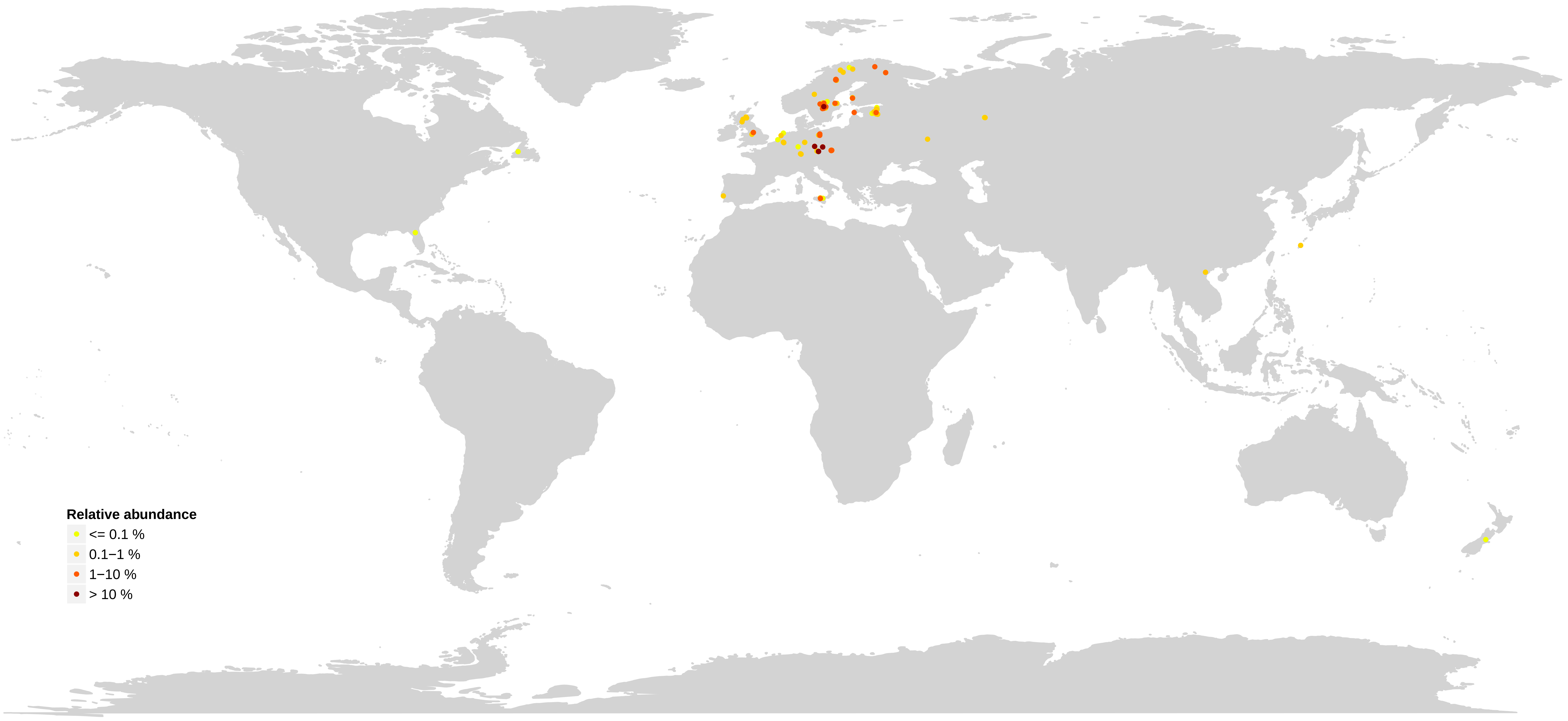

SH183331 Geomyces auratus

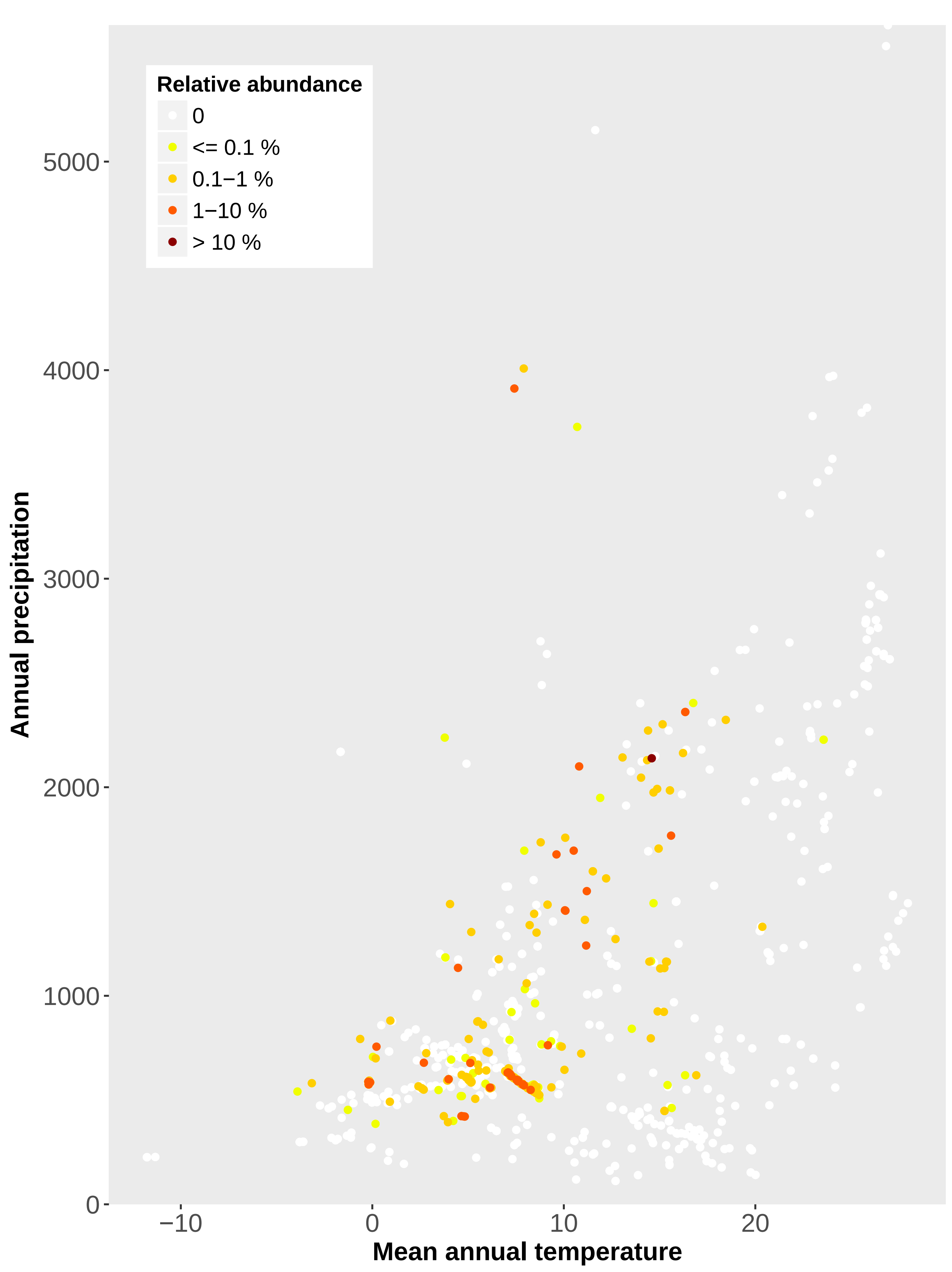

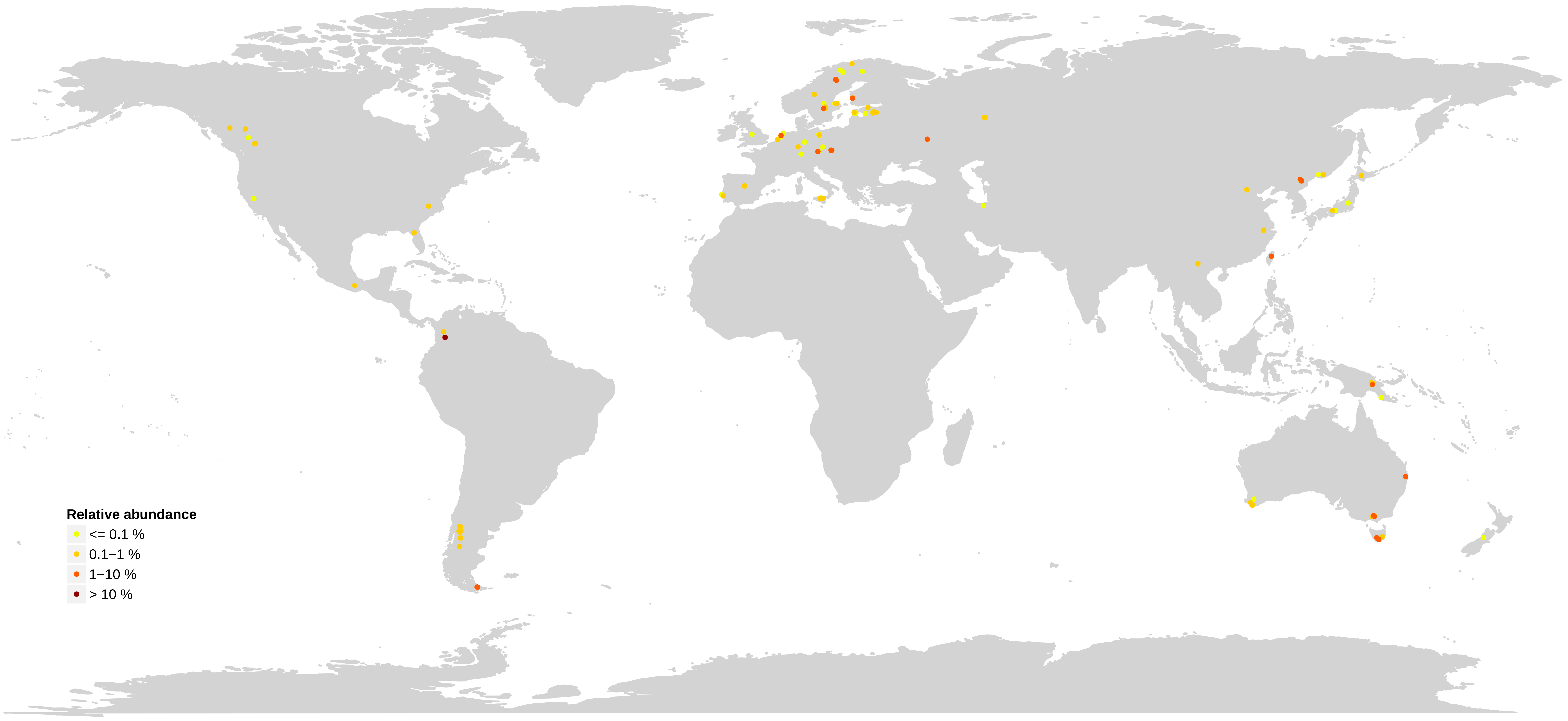

SH195774 Aureobasidium pullulans

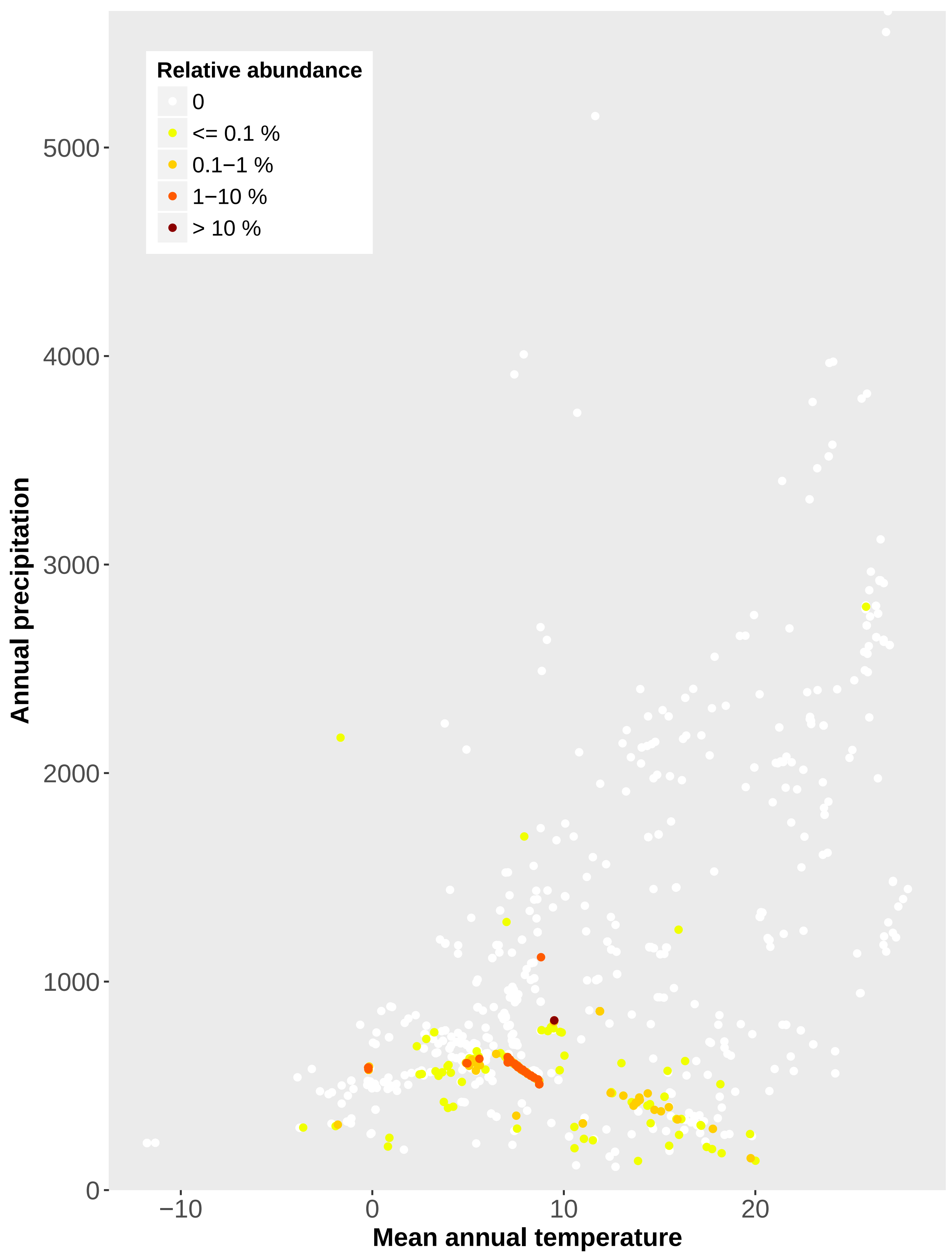

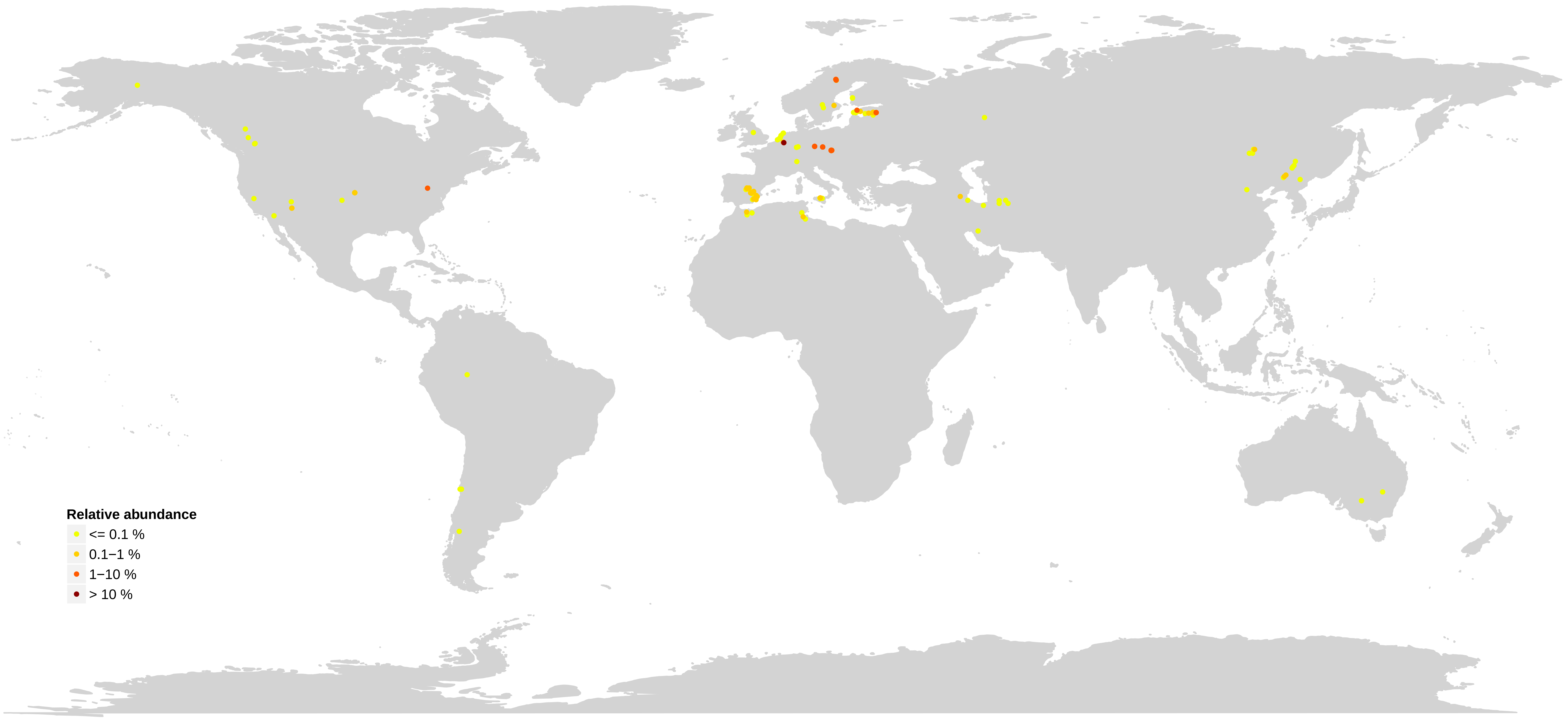

SH190469 Russula ochroleuca

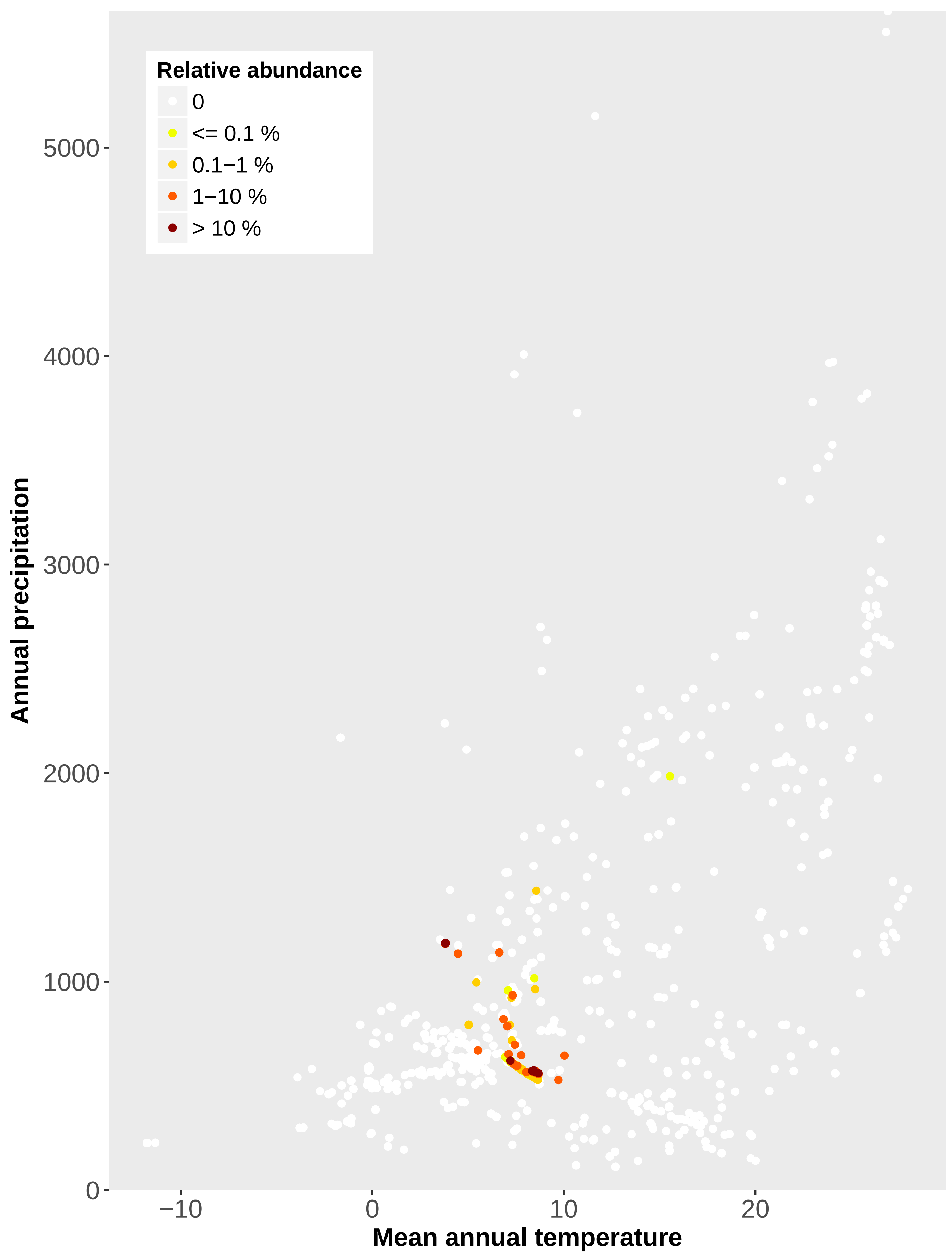

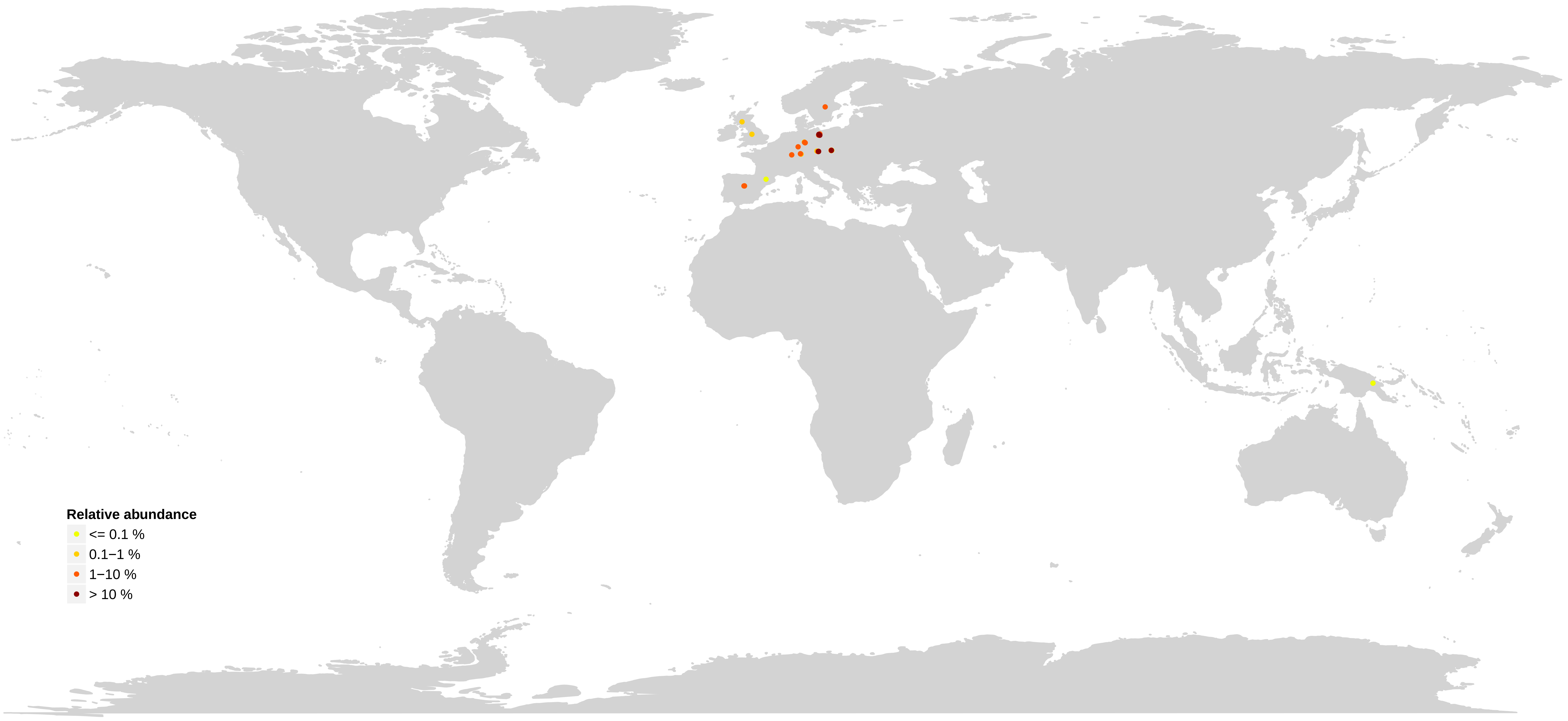

SH220118 Lactarius quietus

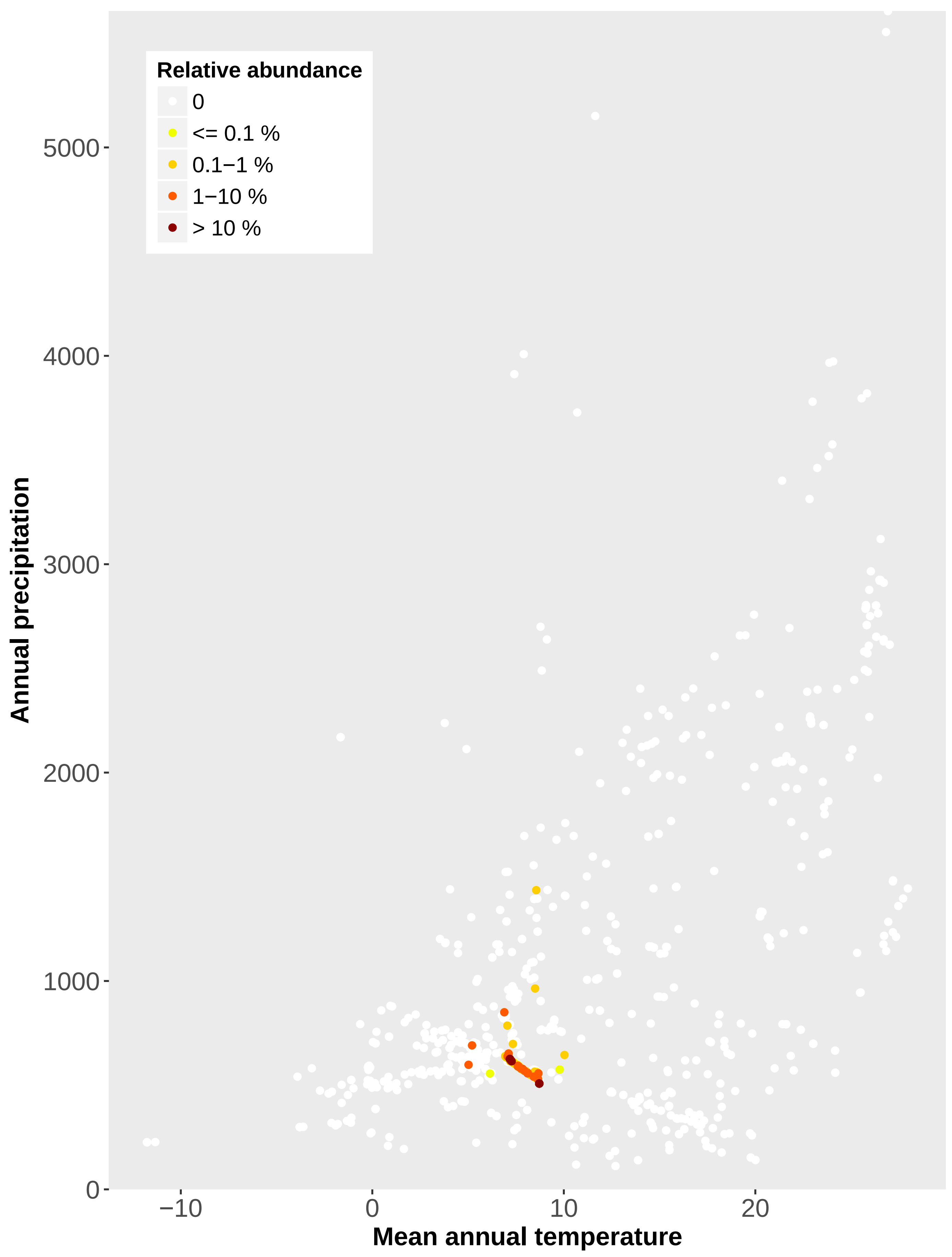

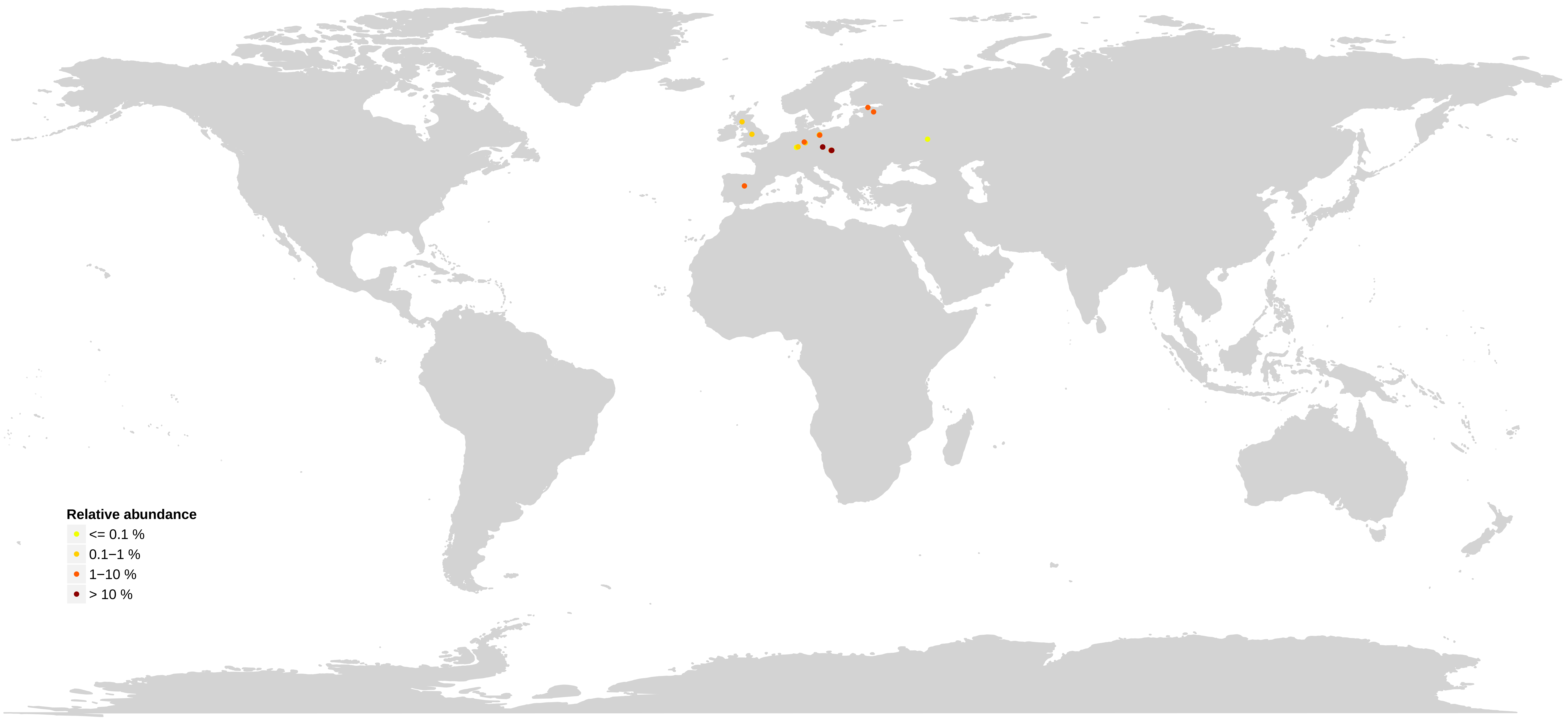

SH184510 Thelephoraceae sp

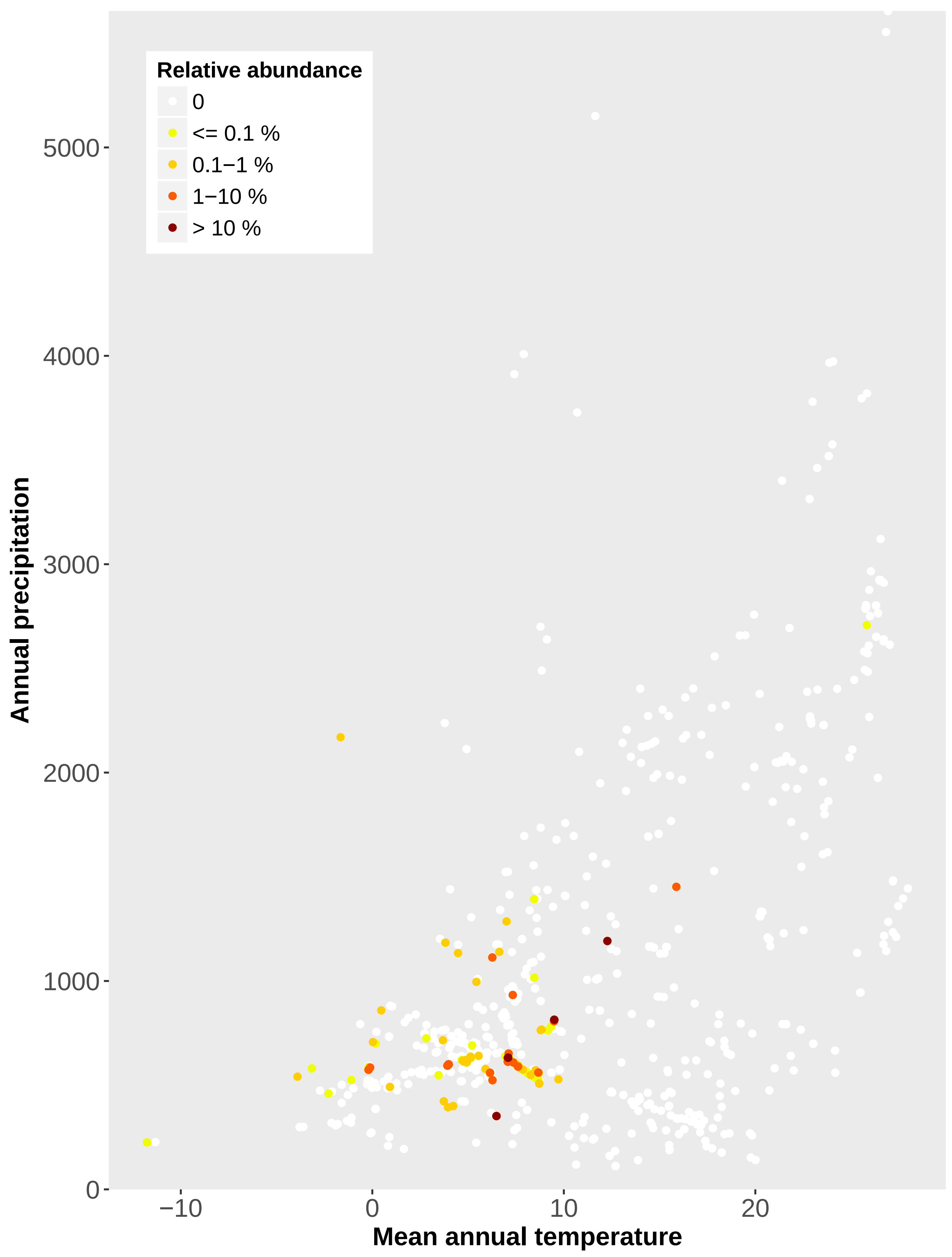

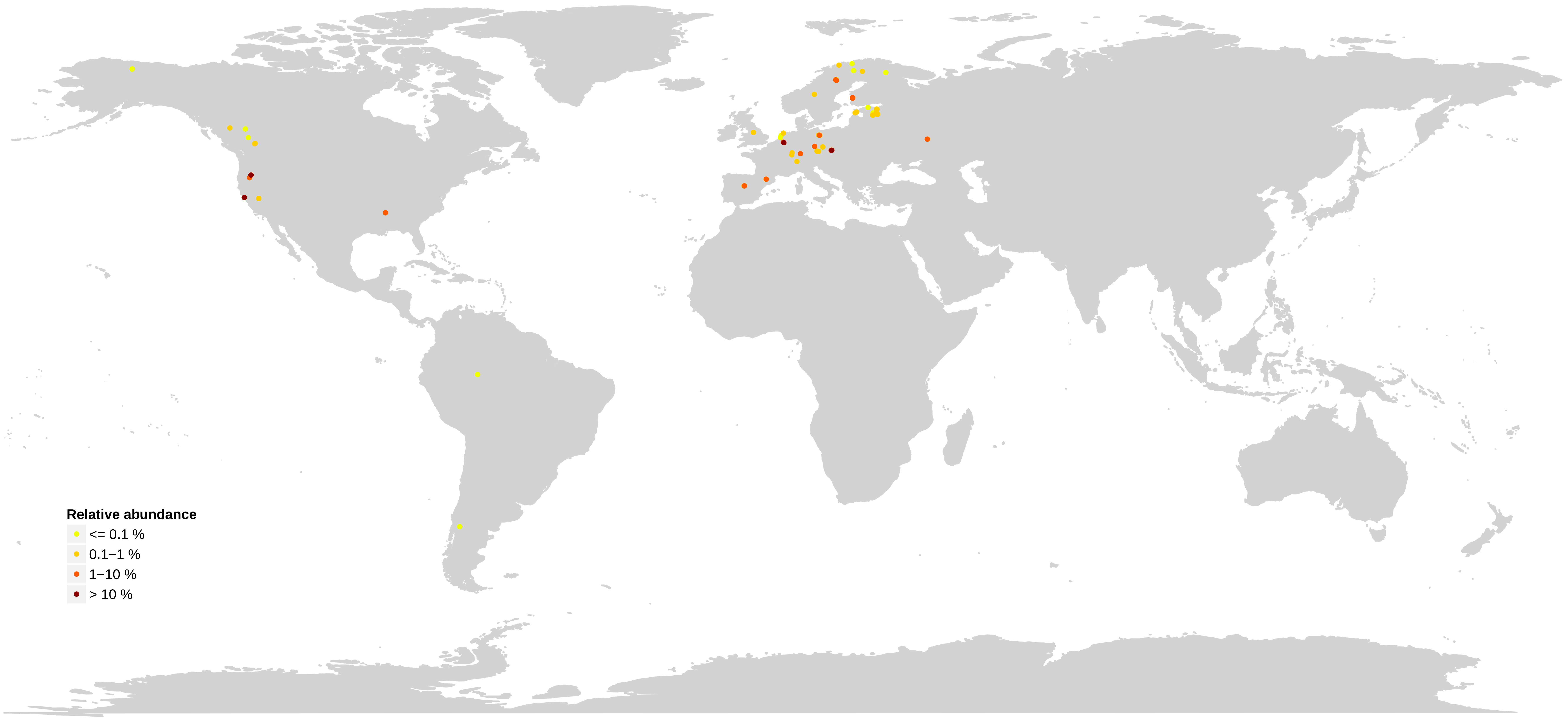

SH345889 Peyronellaea sp

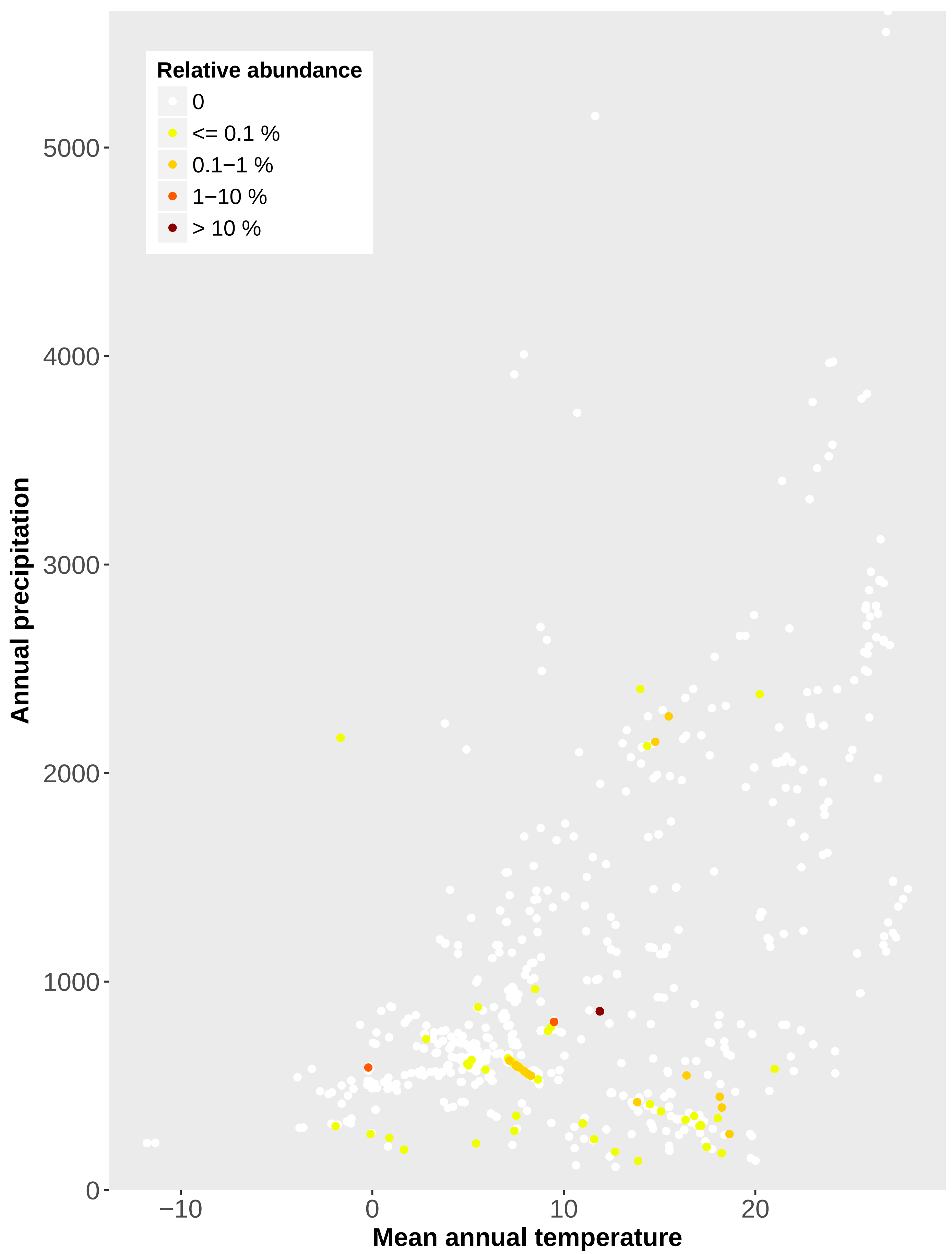

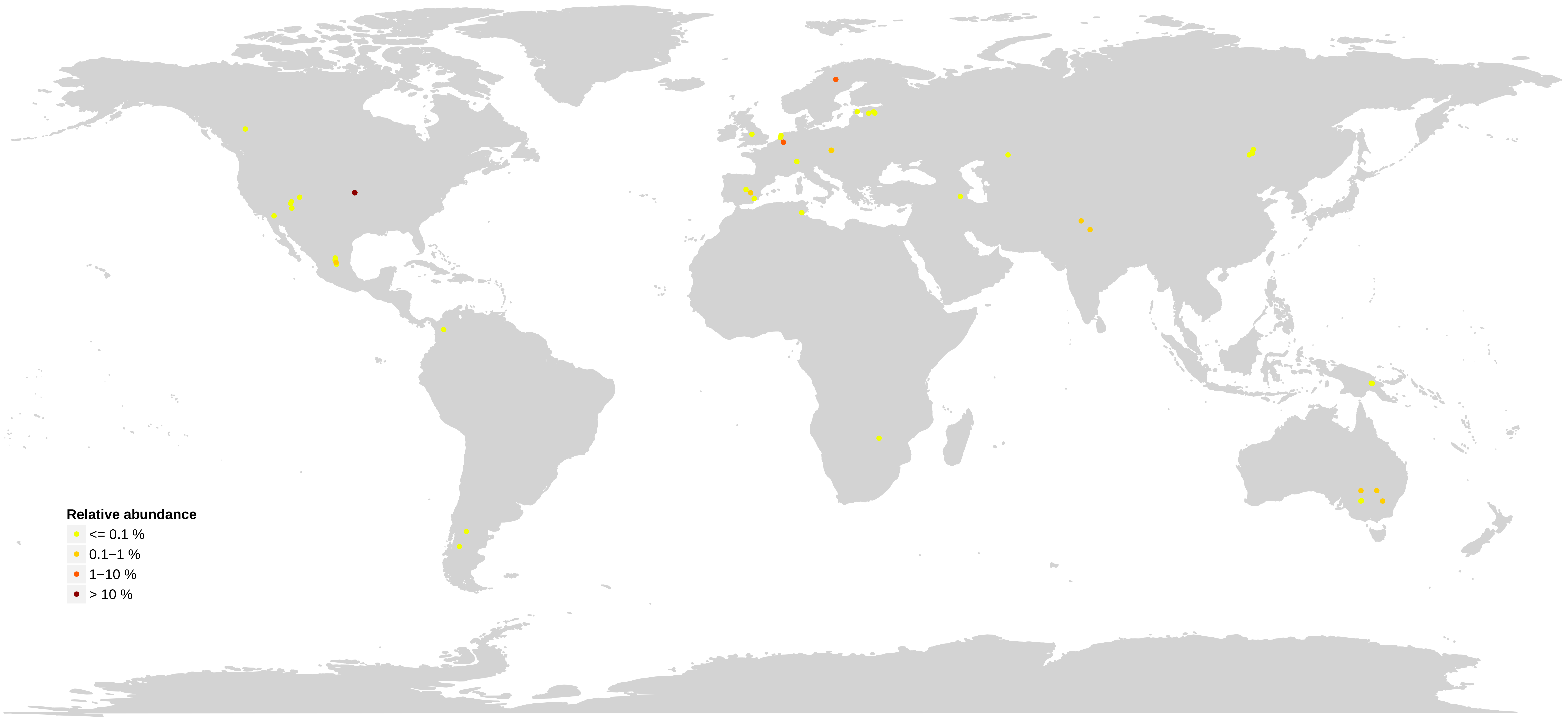

SH216998 Oidiodendron periconioides

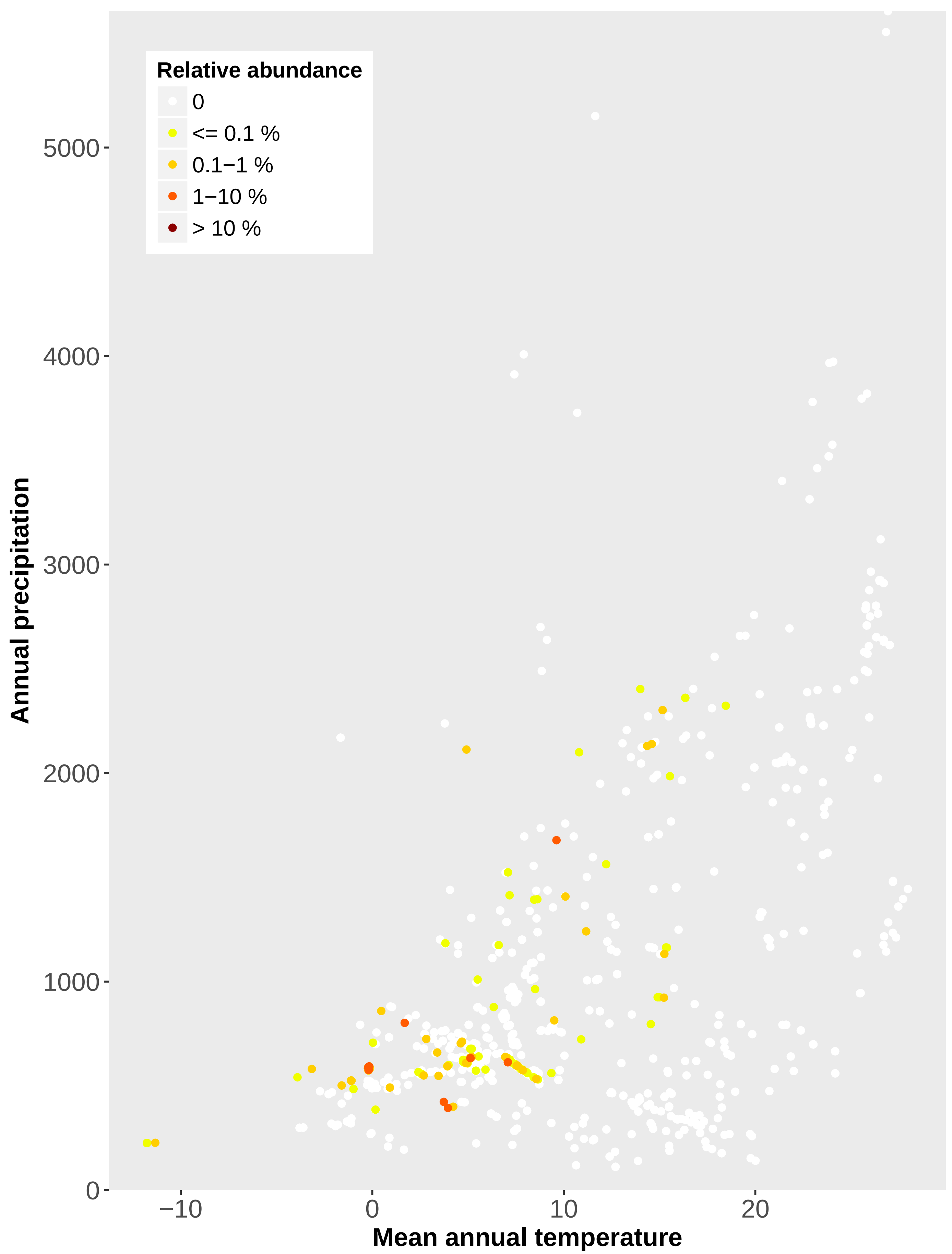

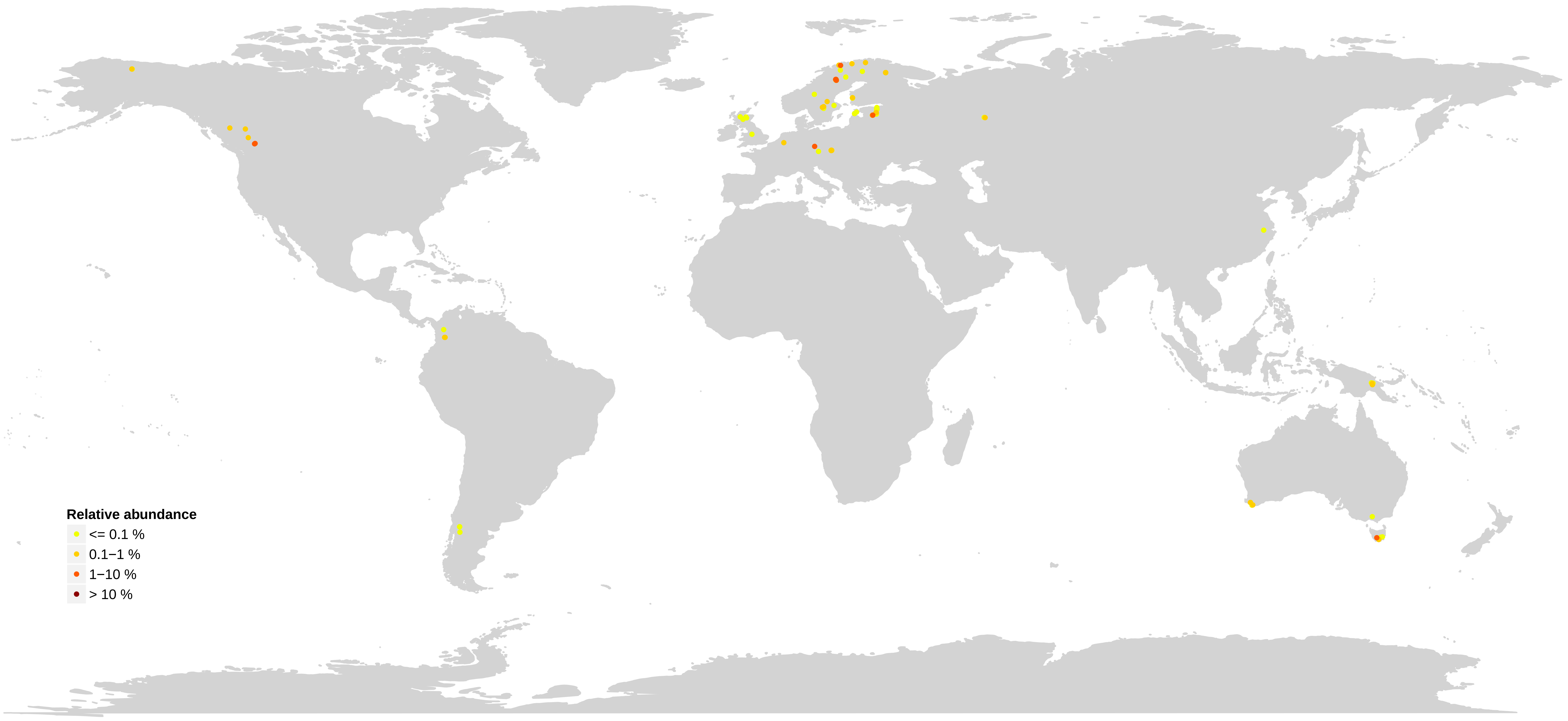

SH215930 Ascomycota sp

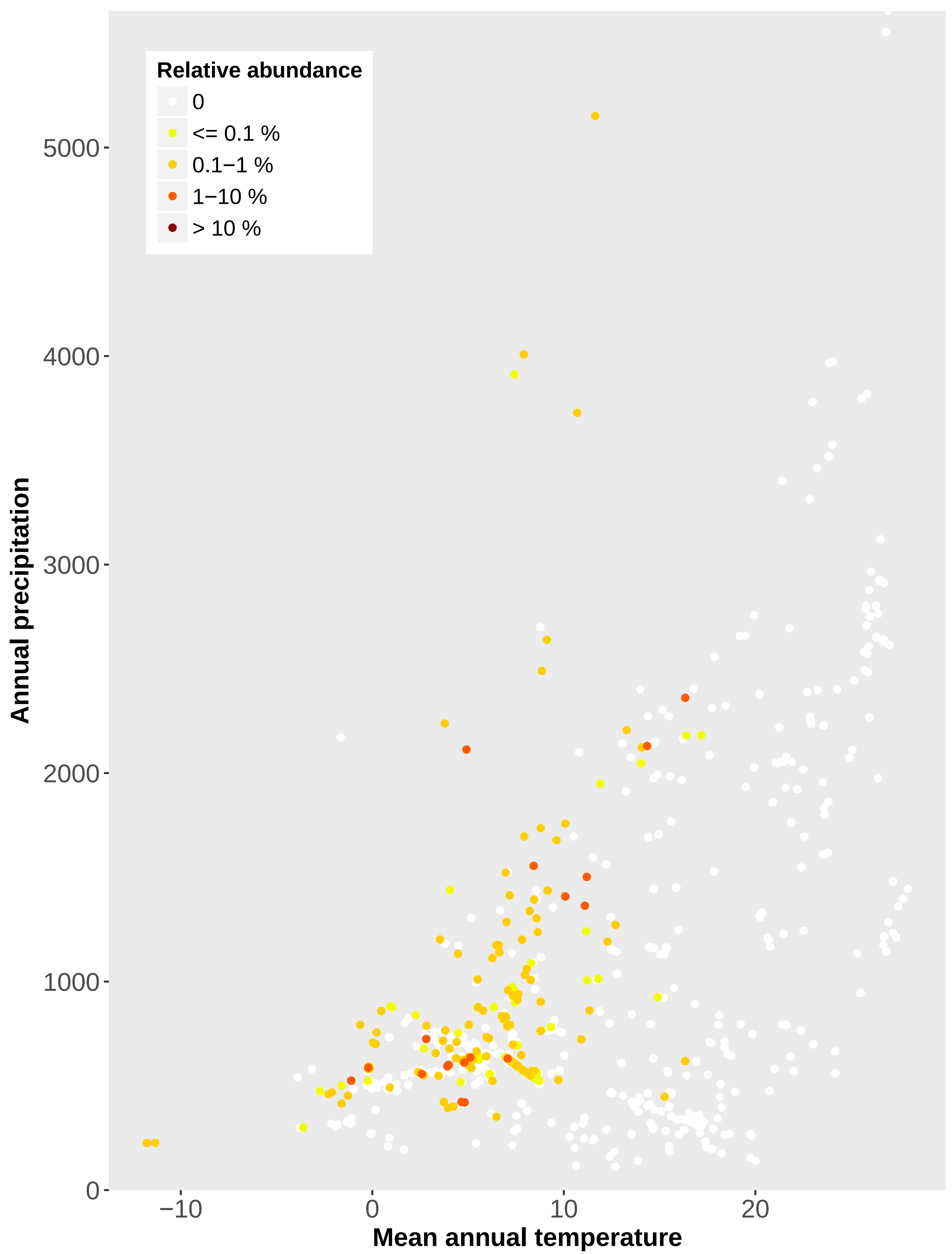

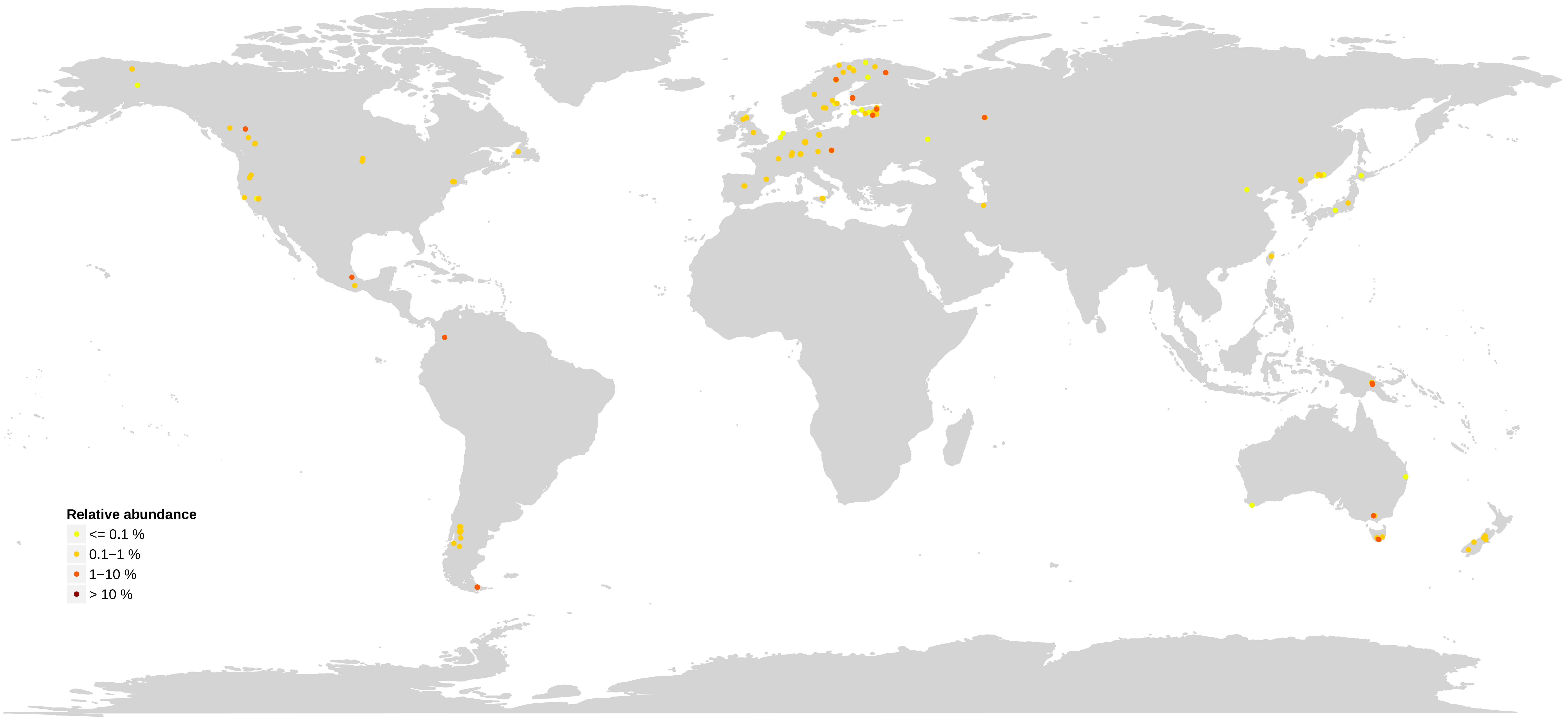

SH193764 Basidiomycota sp

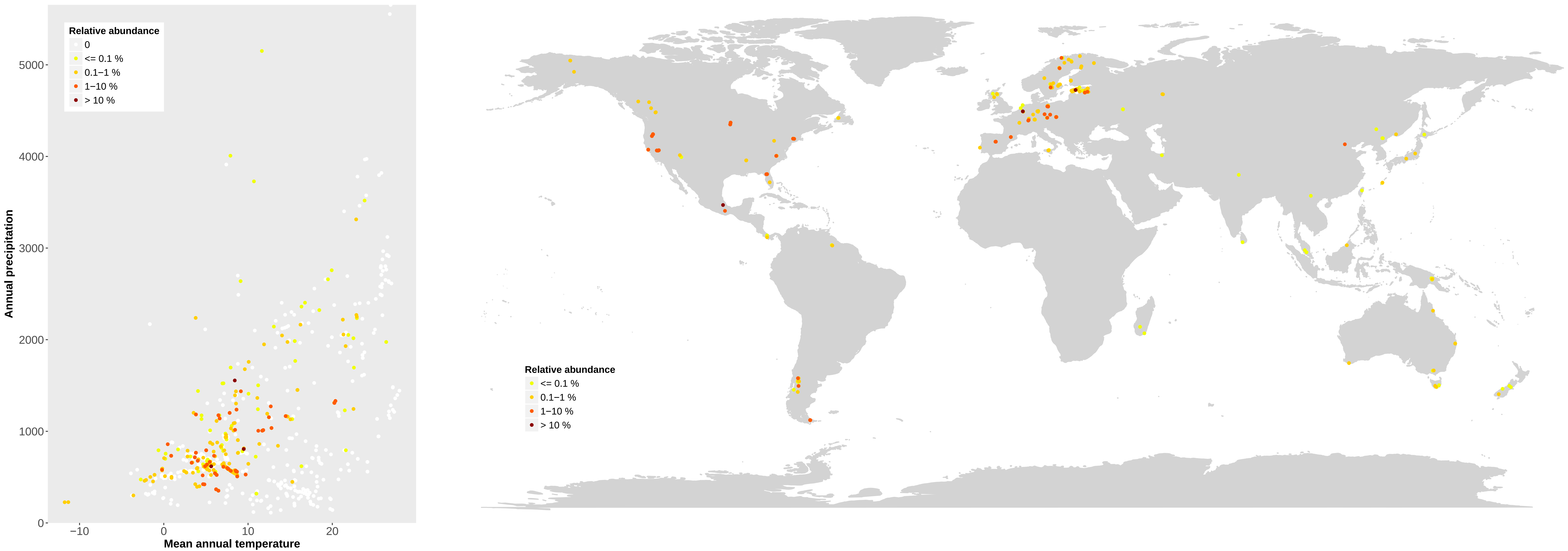

SH194257 Helotiales sp

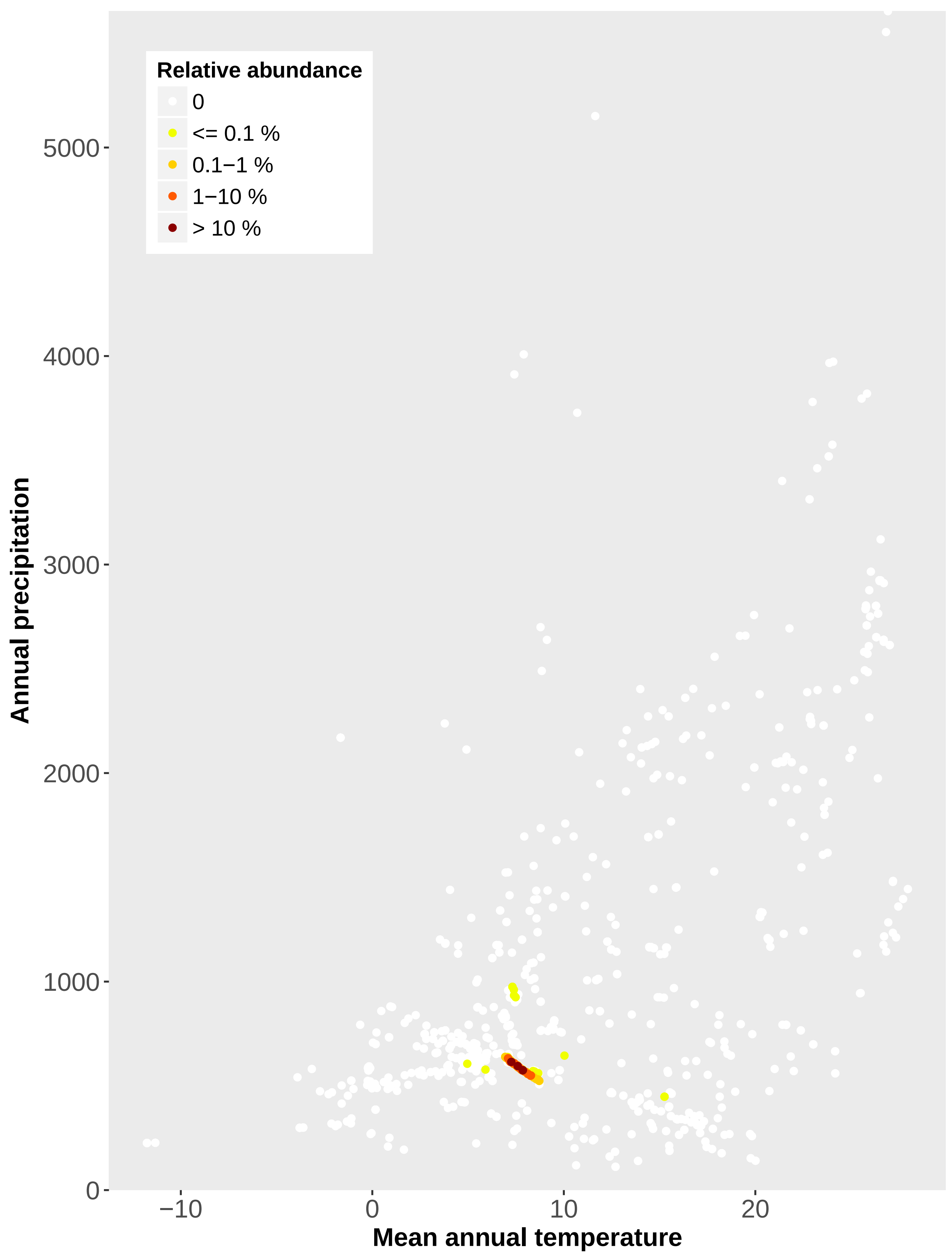

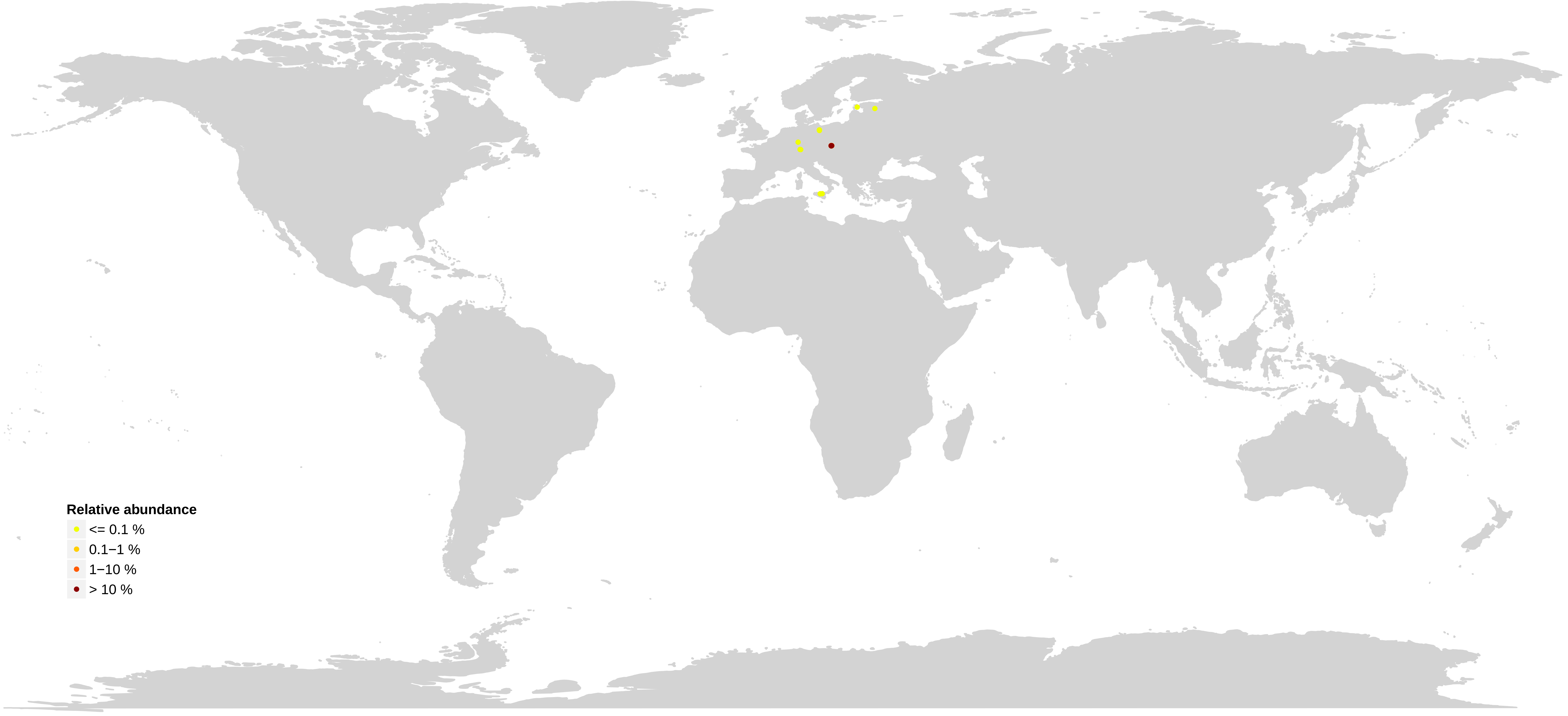

SH187863 Mortierella macrocystis

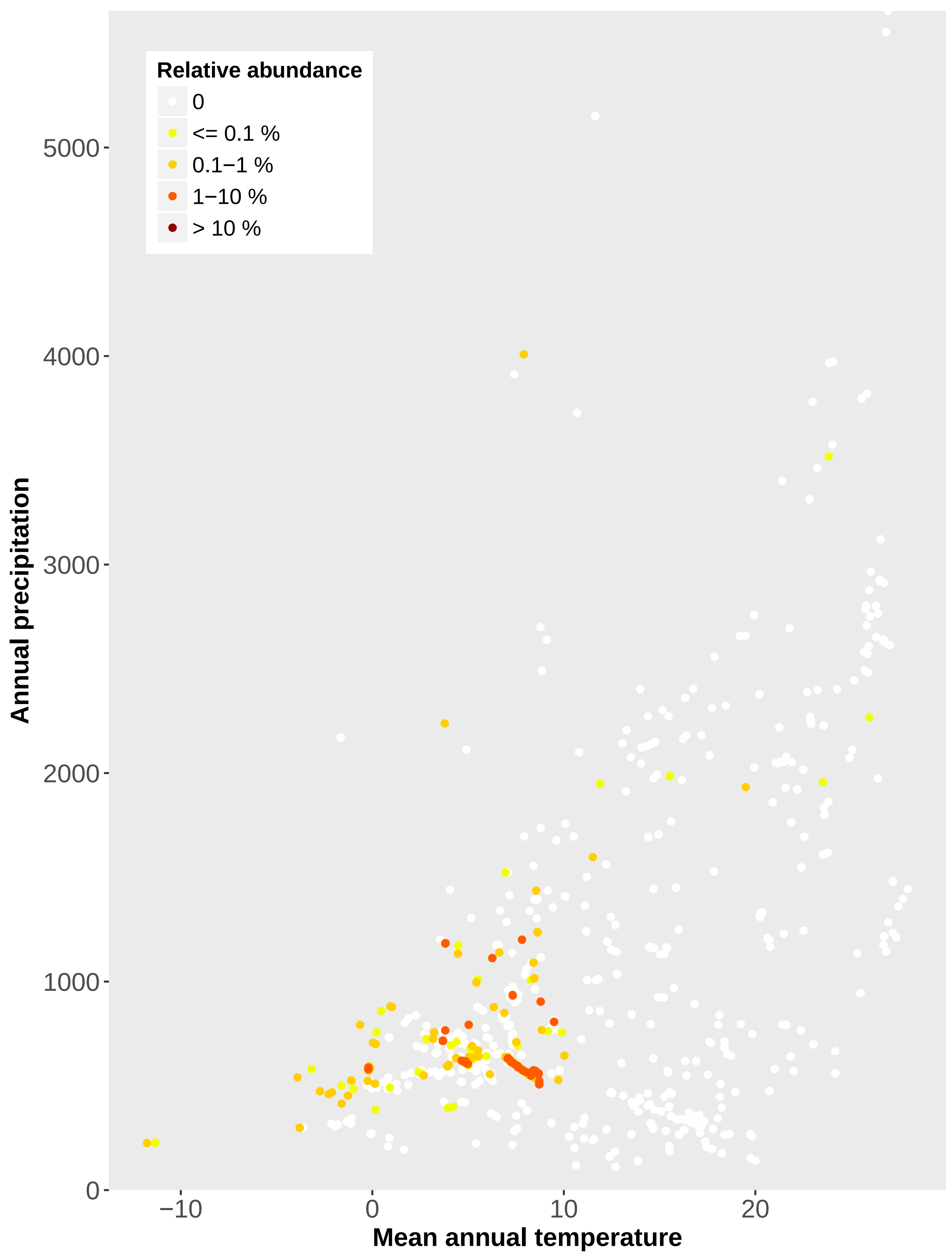

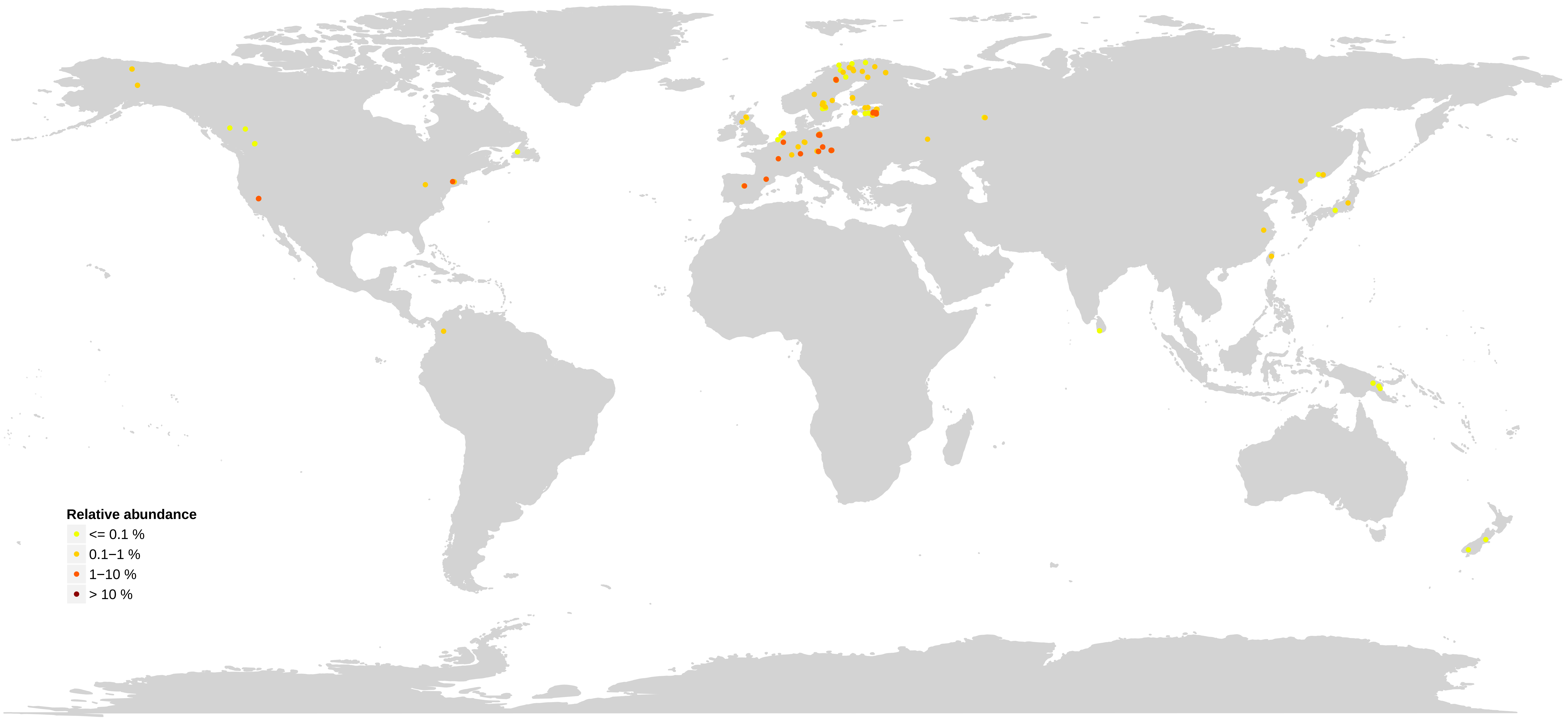

SH196093 Umbelopsis sp

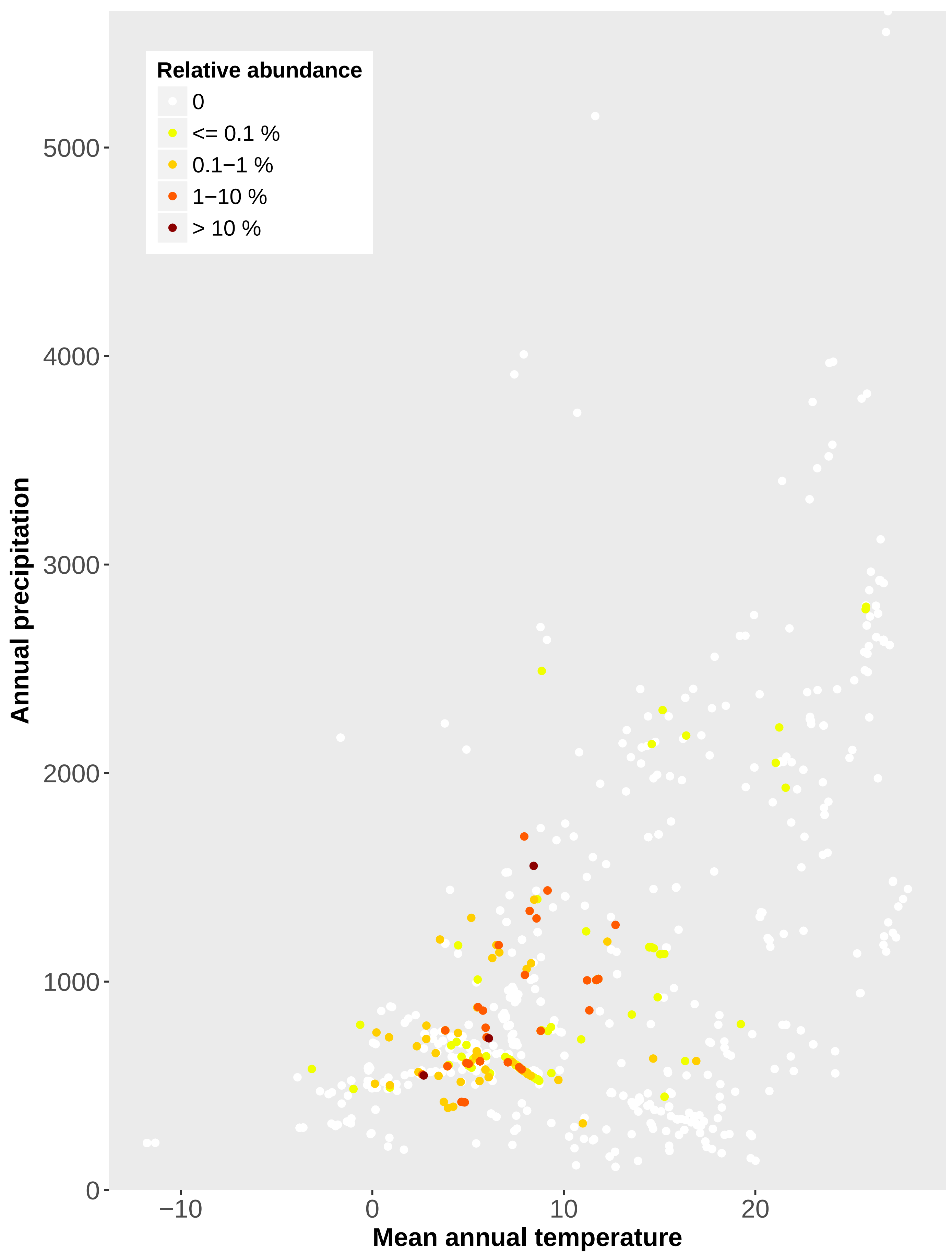

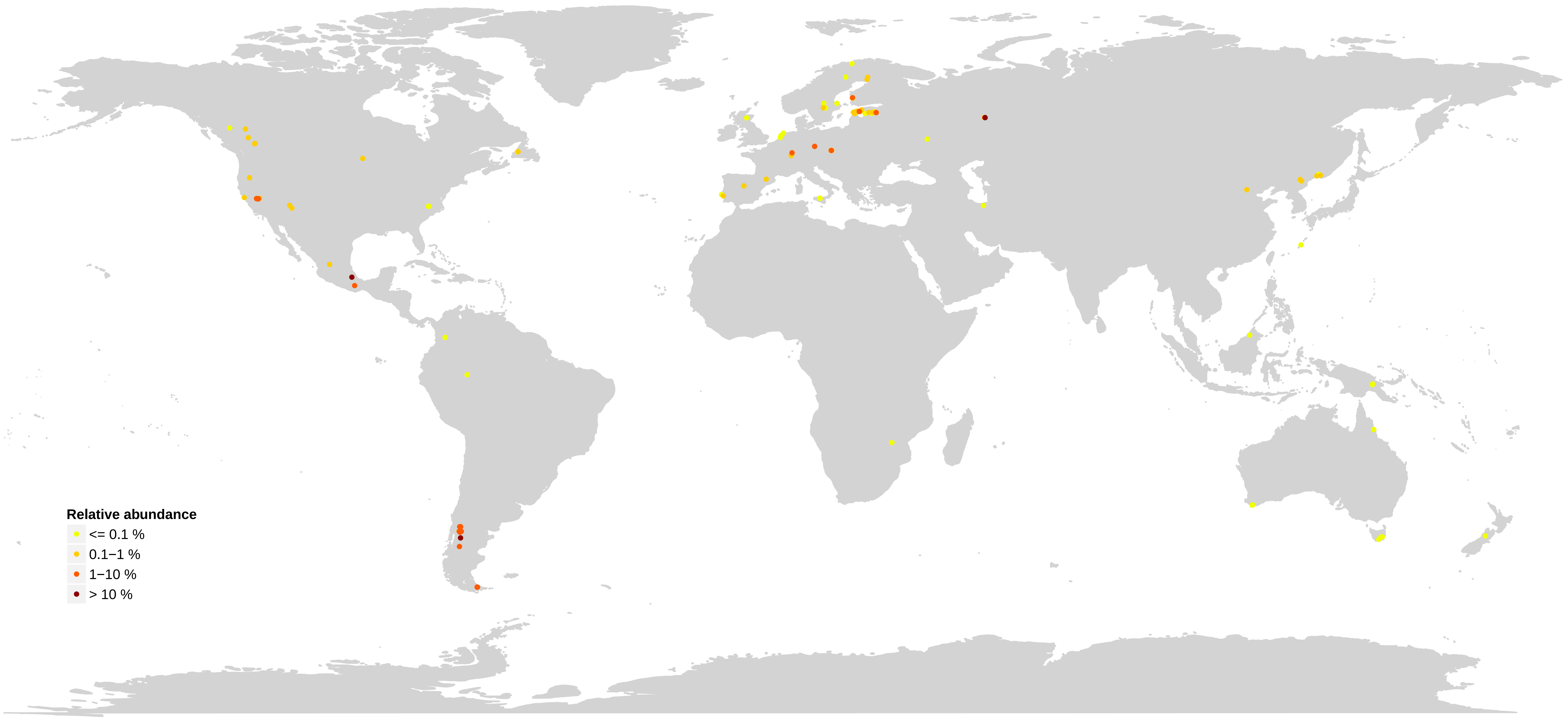

SH188230 Ascomycota sp

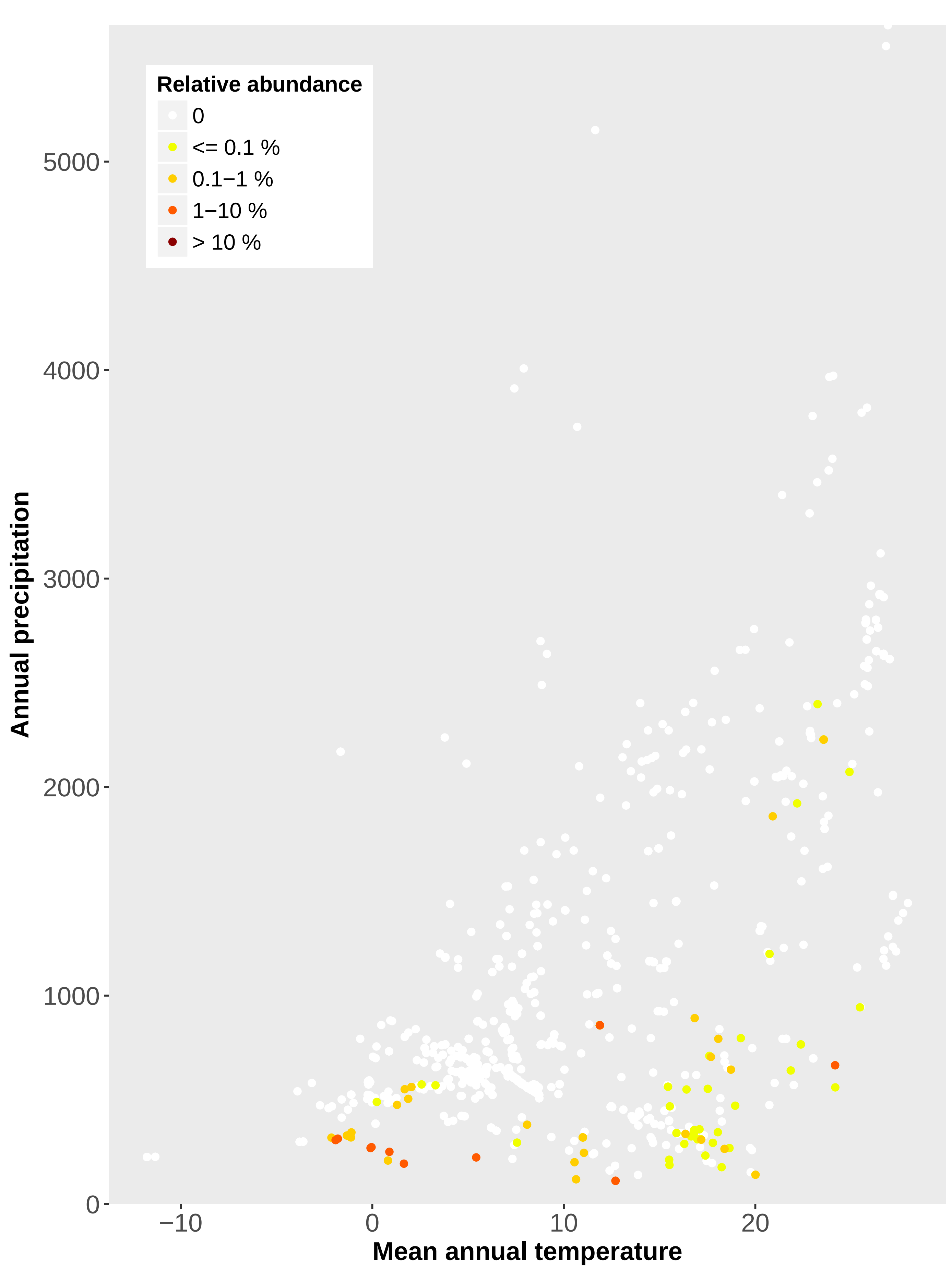

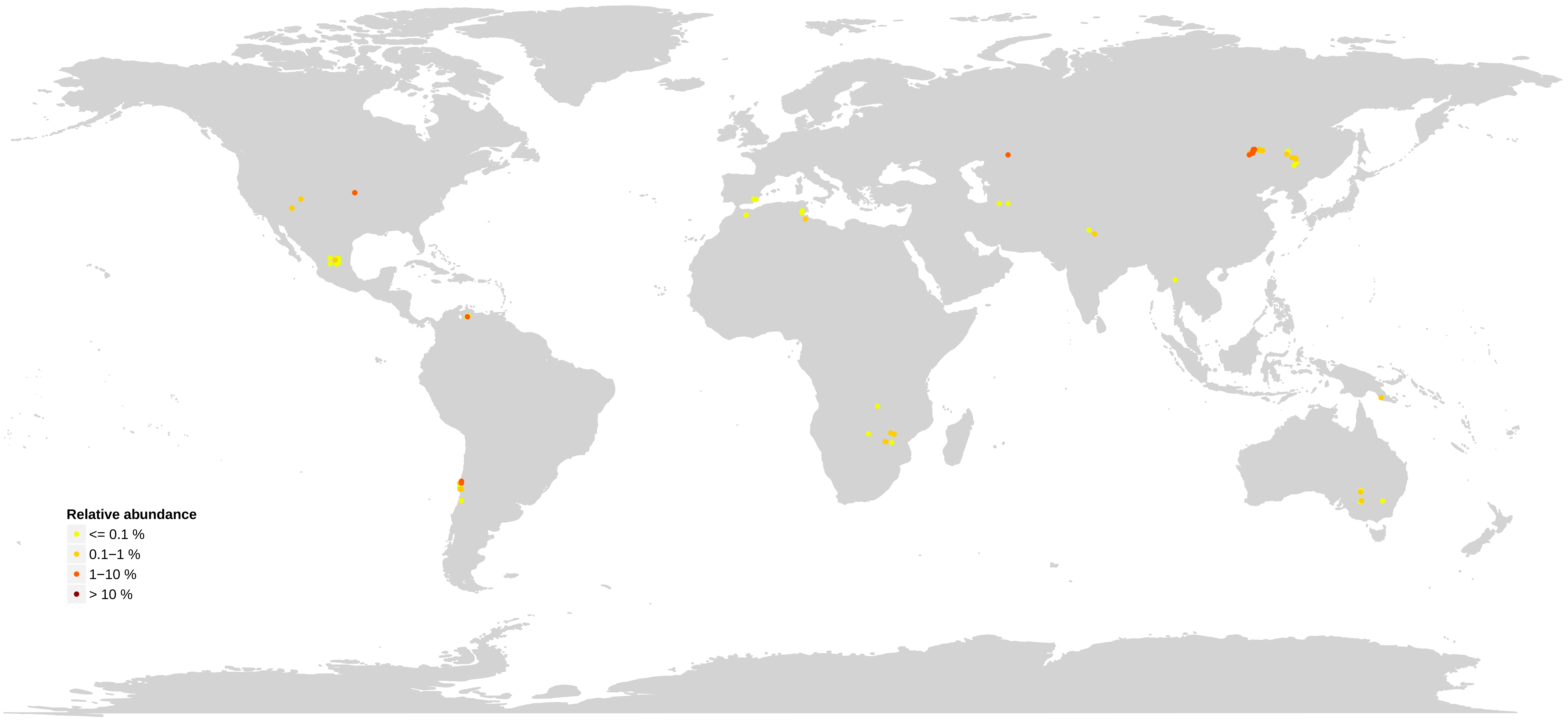

SH214459 Cenococcum geophilum

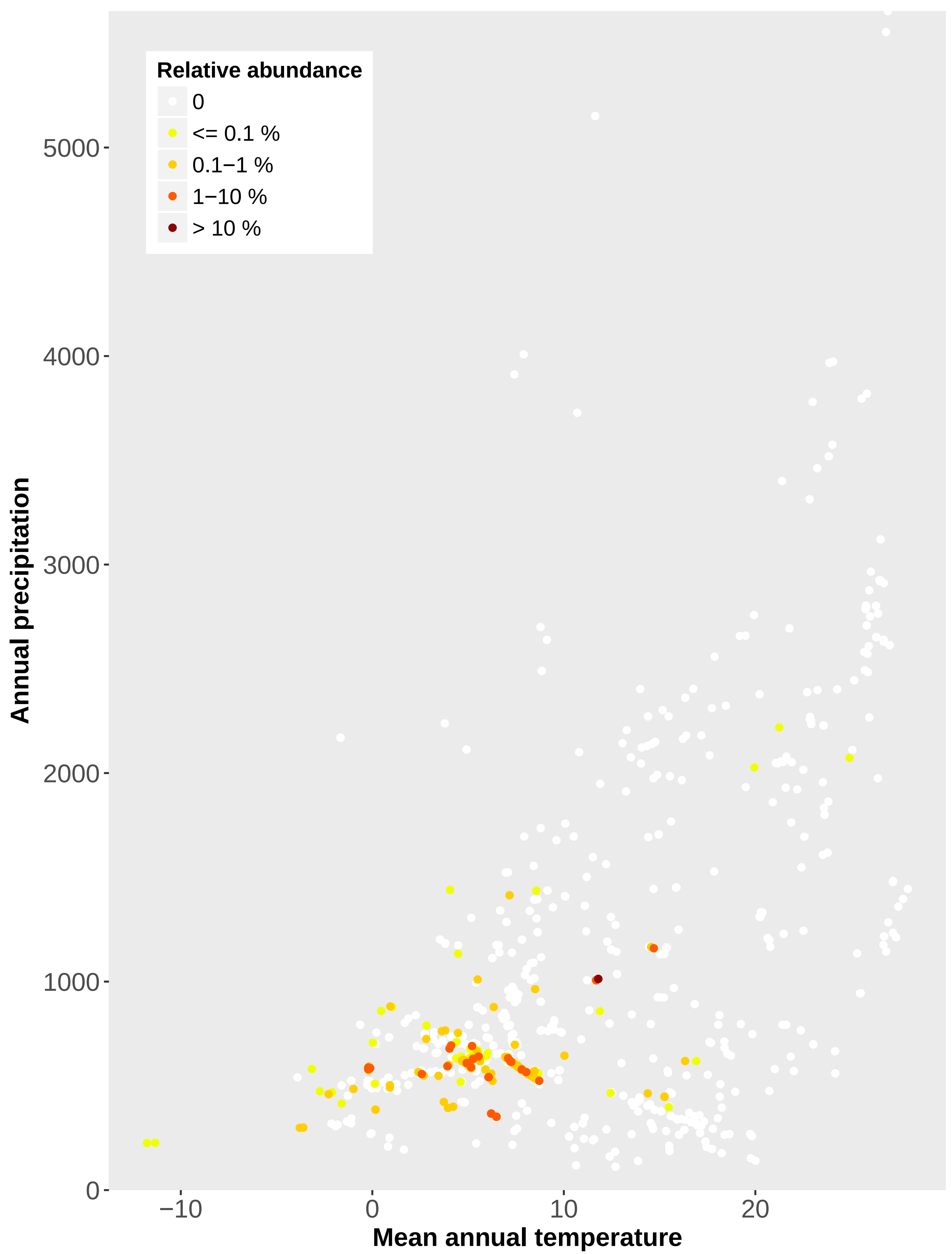

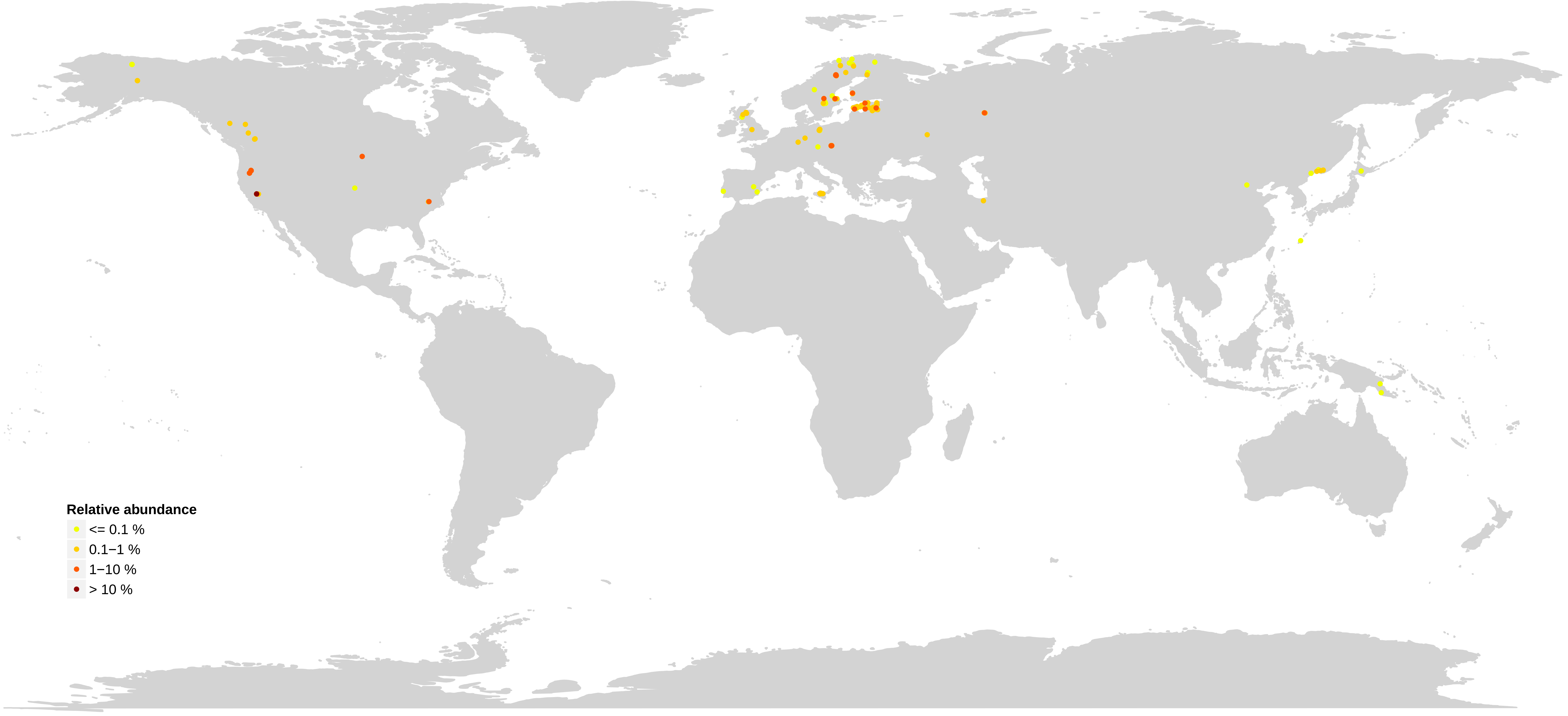

SH198656 Ascomycota sp

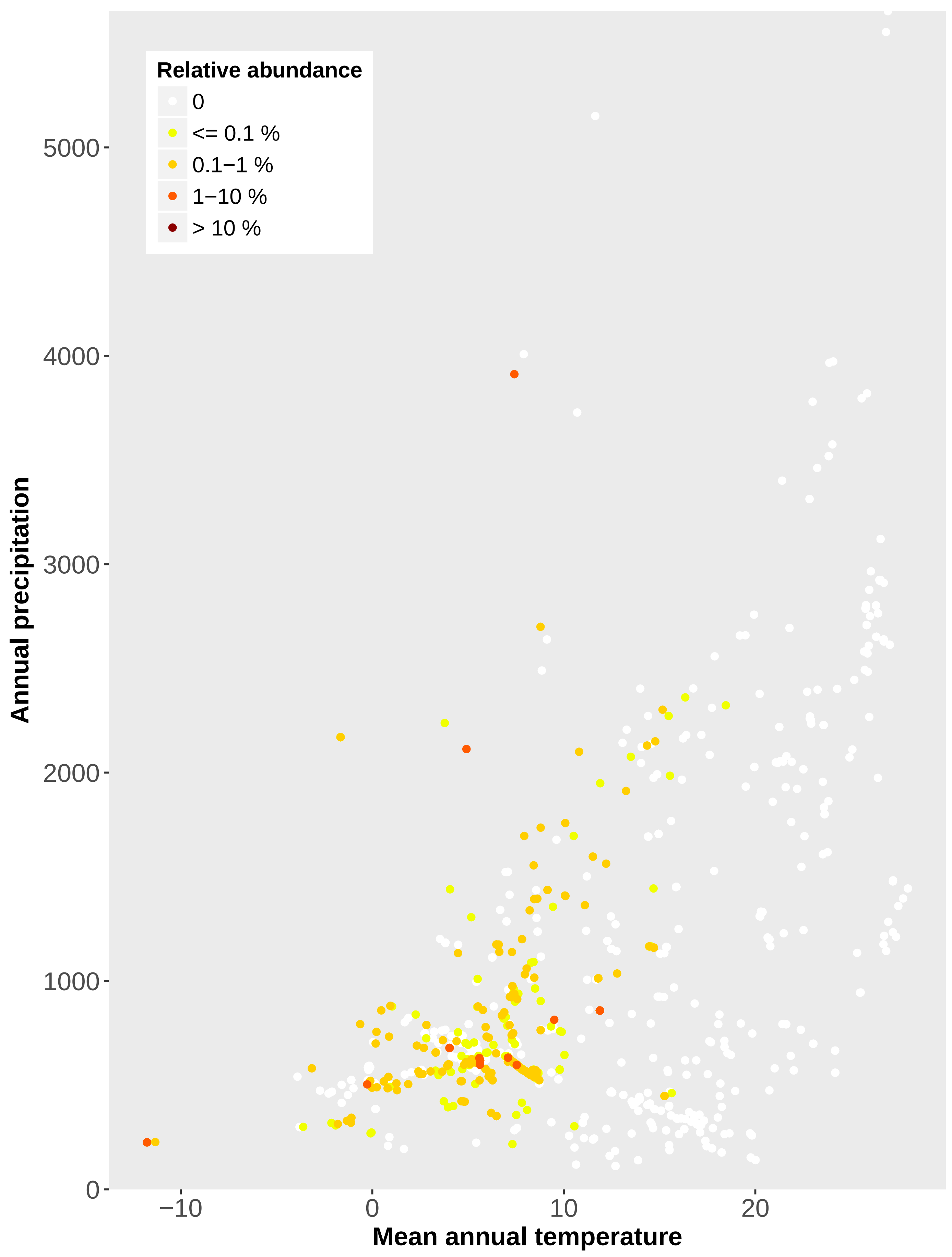

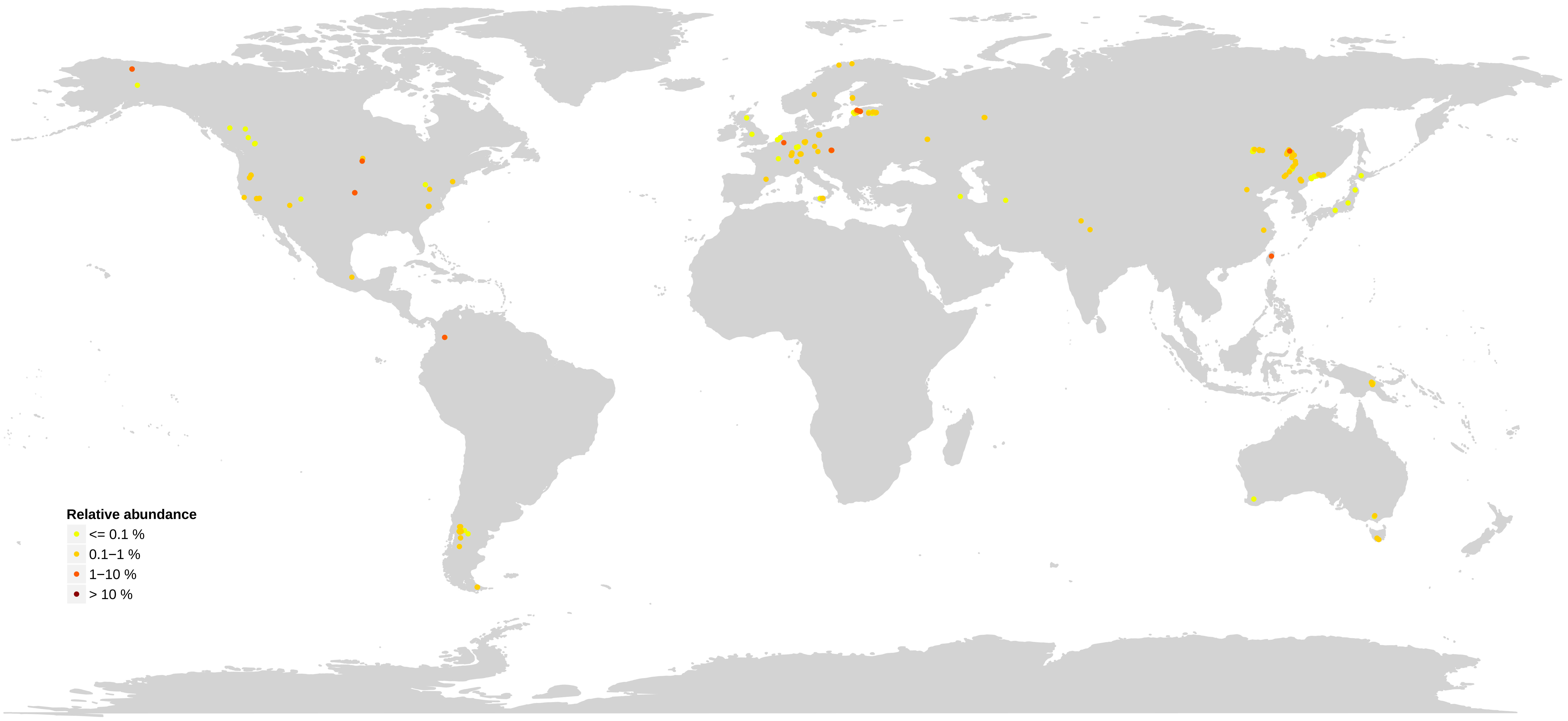

SH279517 Penicillium swieckii

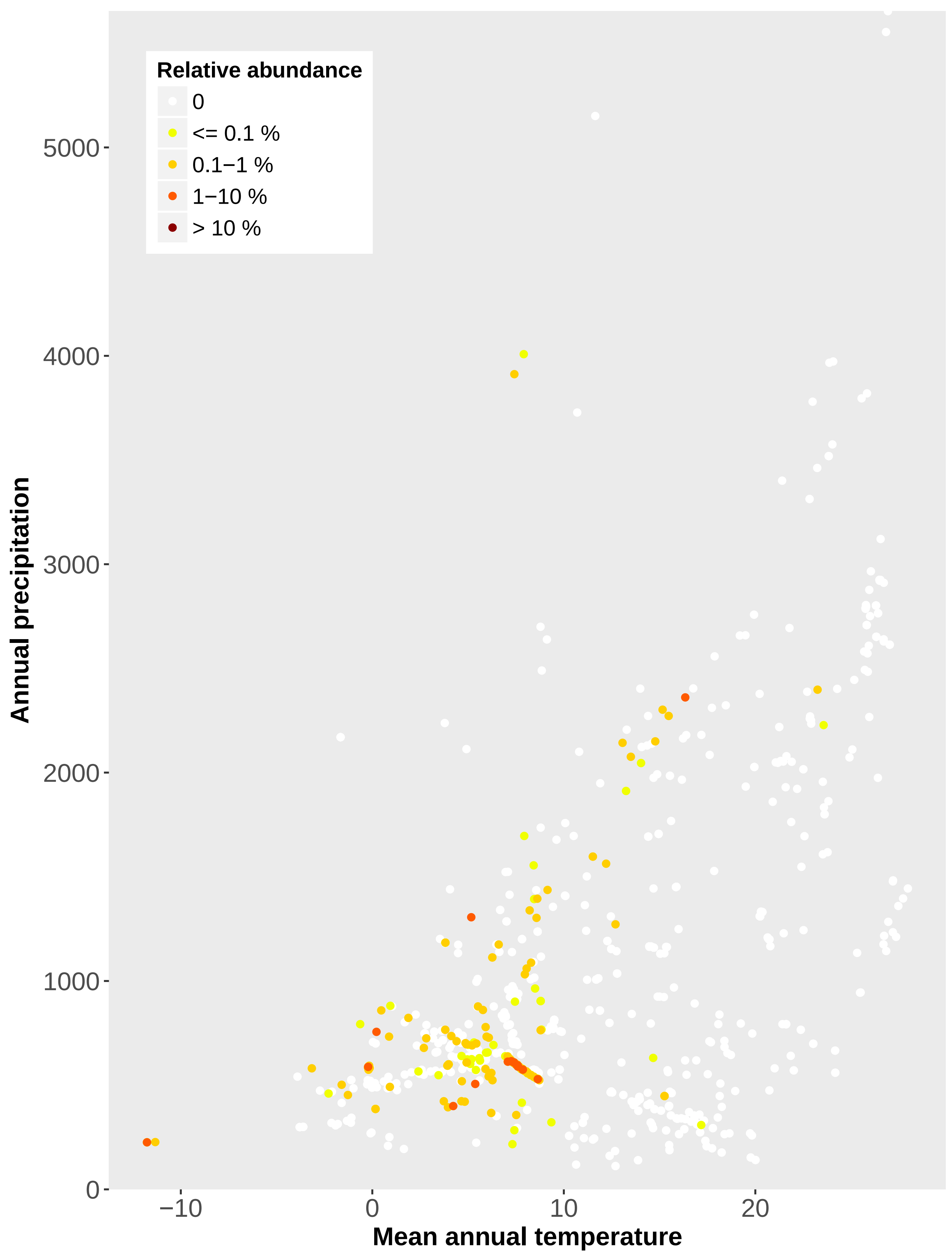

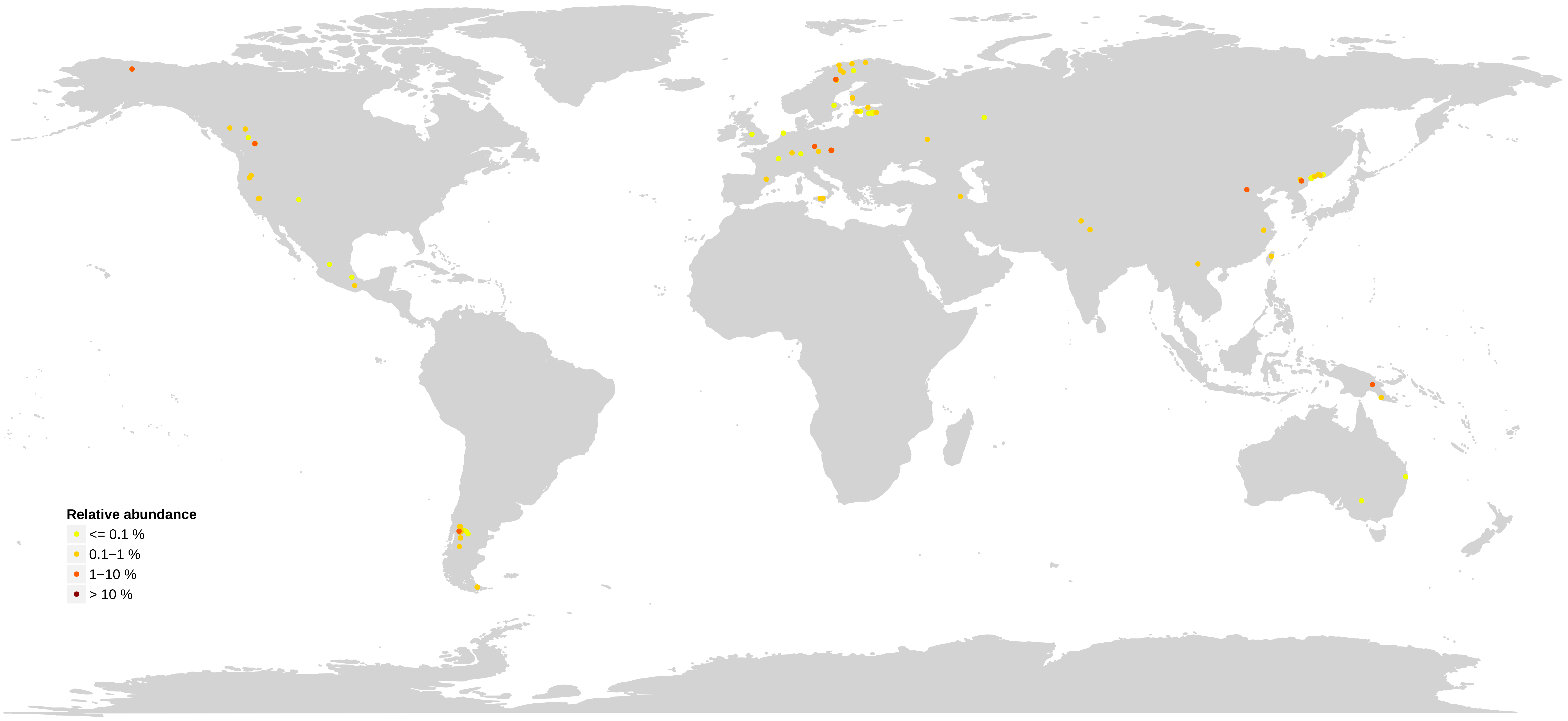

SH179954 Luellia recondita

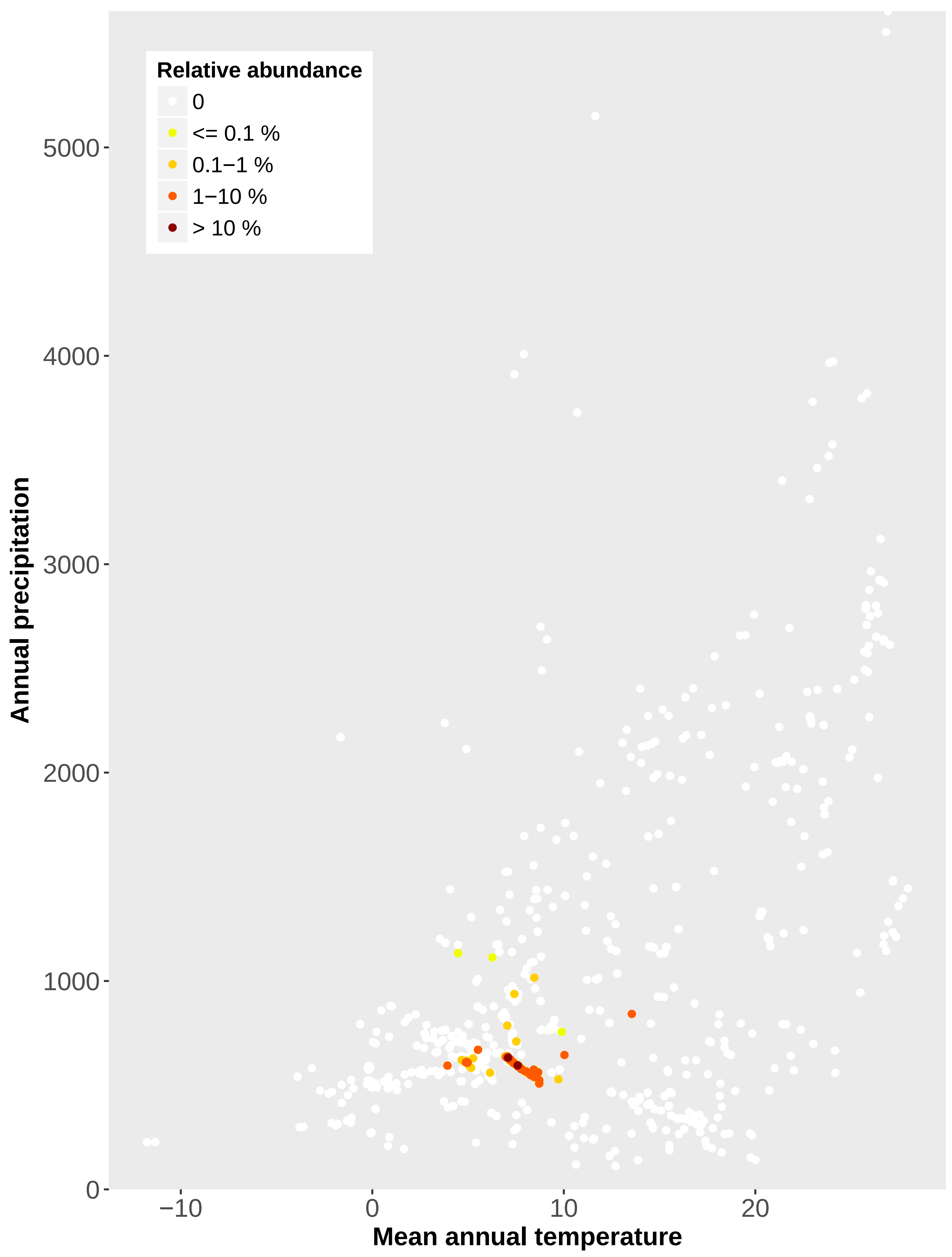

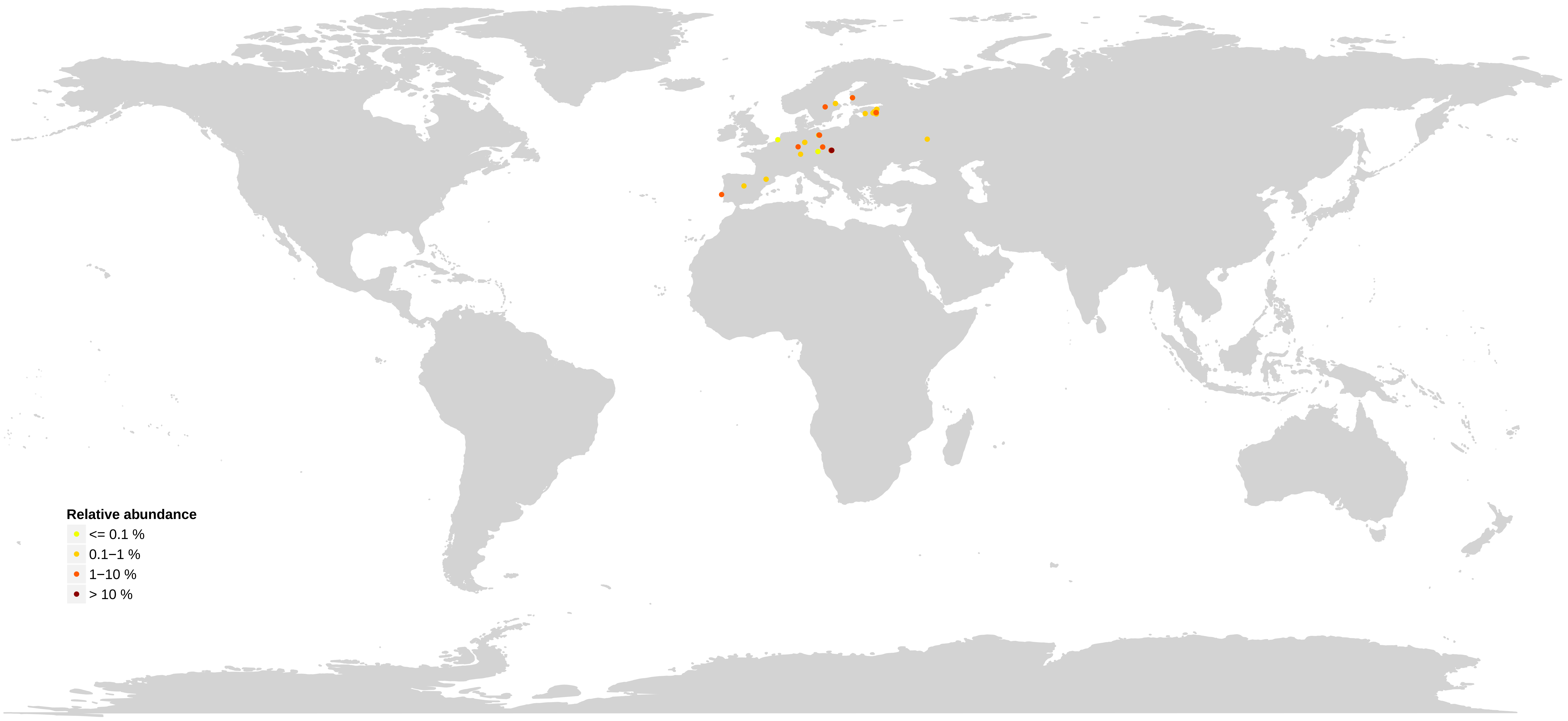

SH219102 Sordariomycetes sp

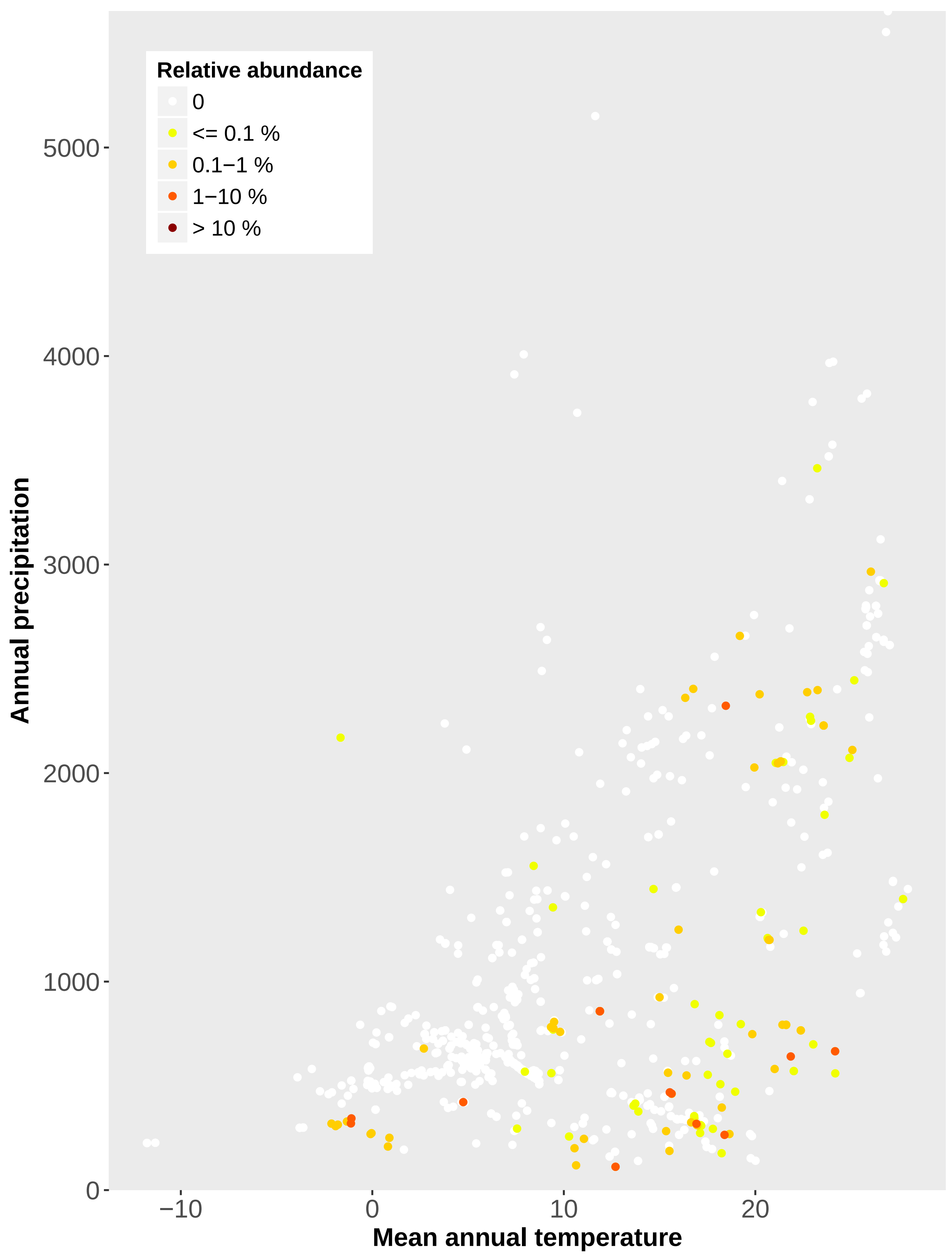

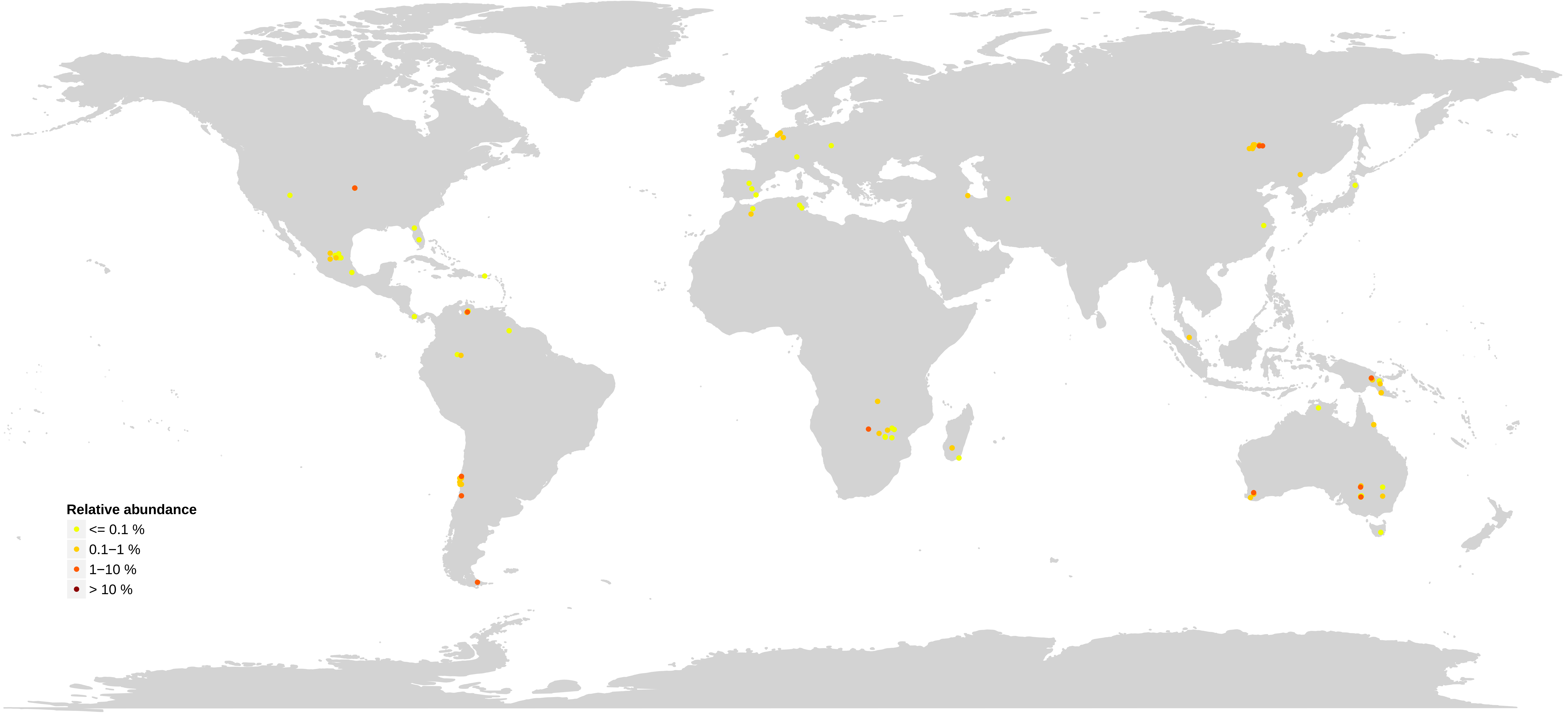

SH199612 Fungi sp

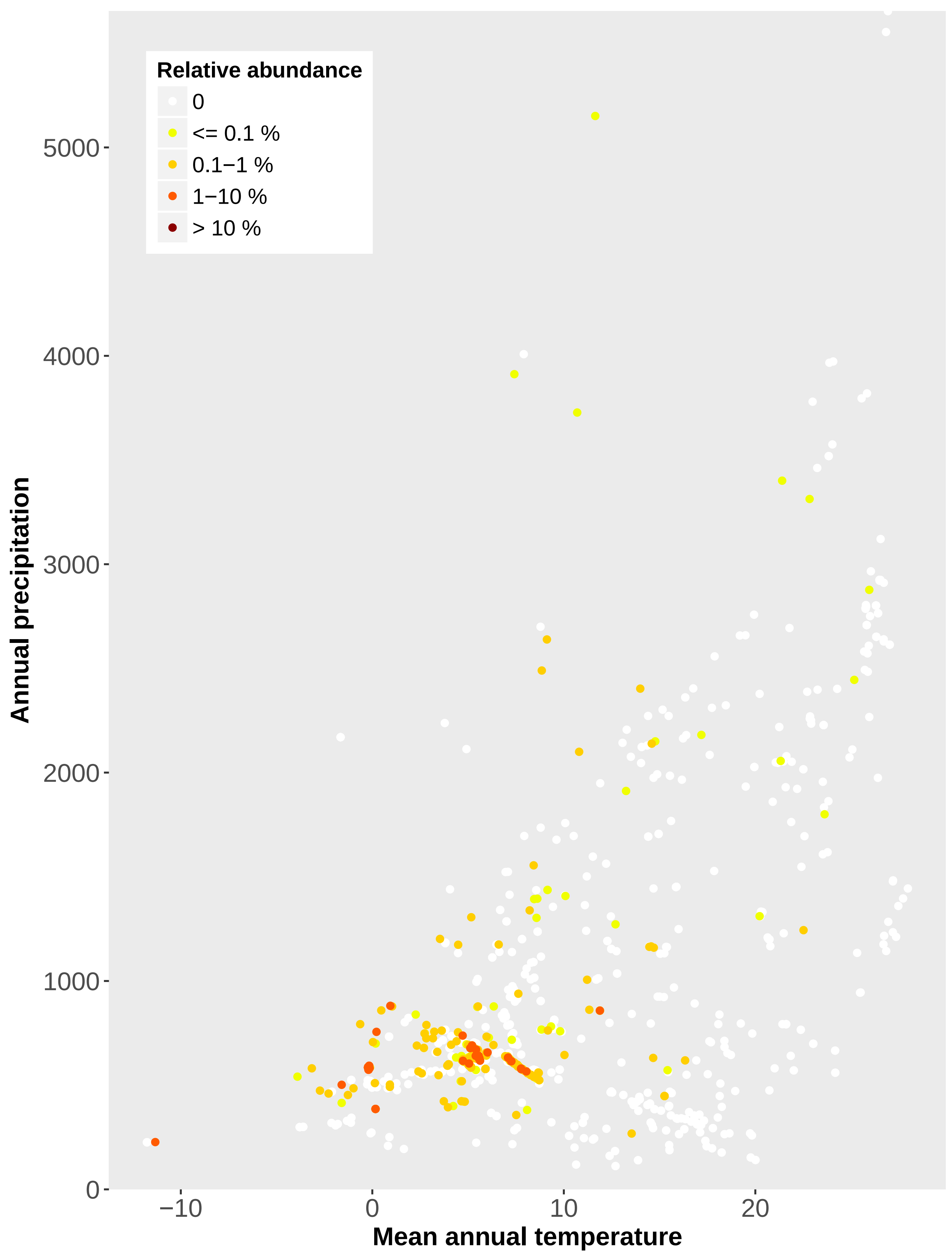

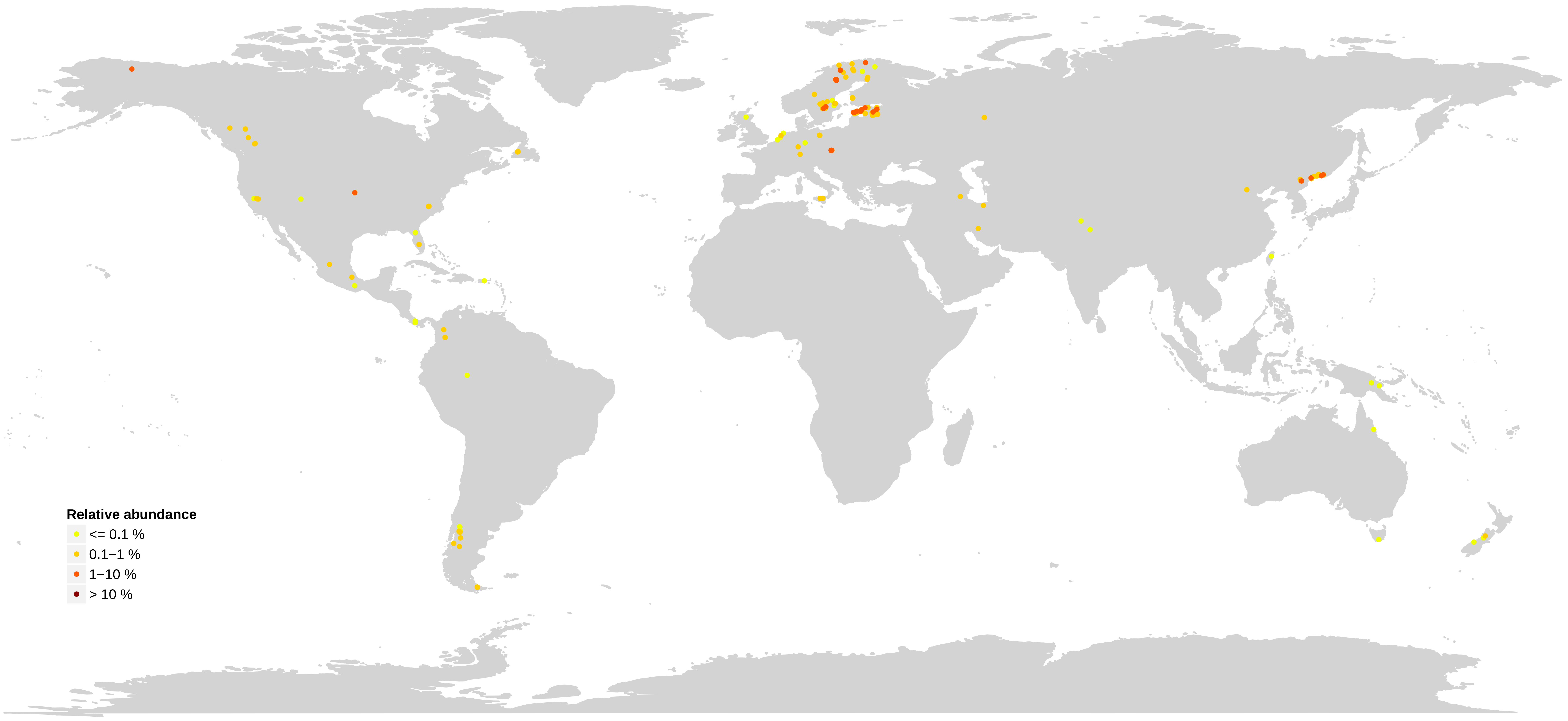

SH407691 Penicillium thomii

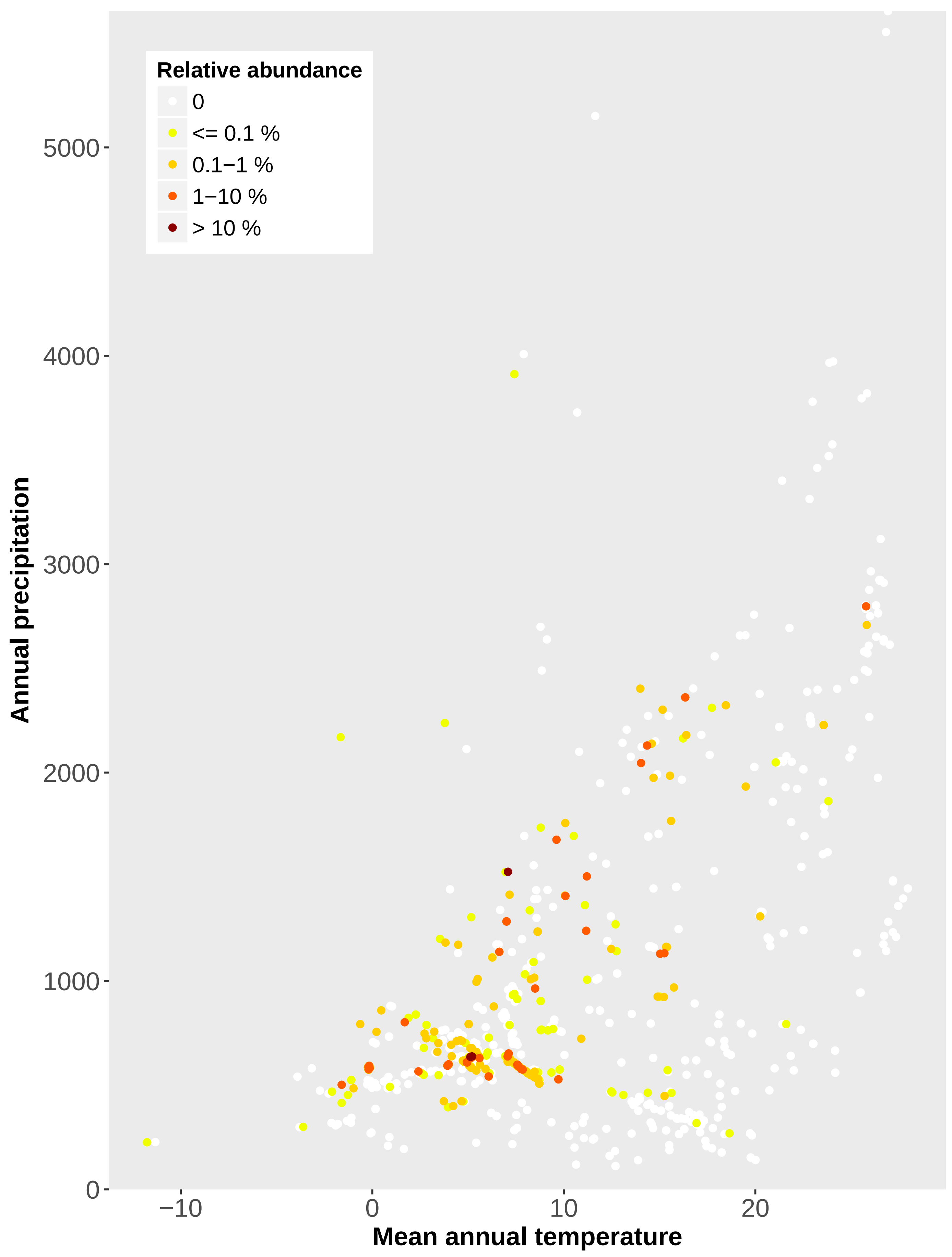

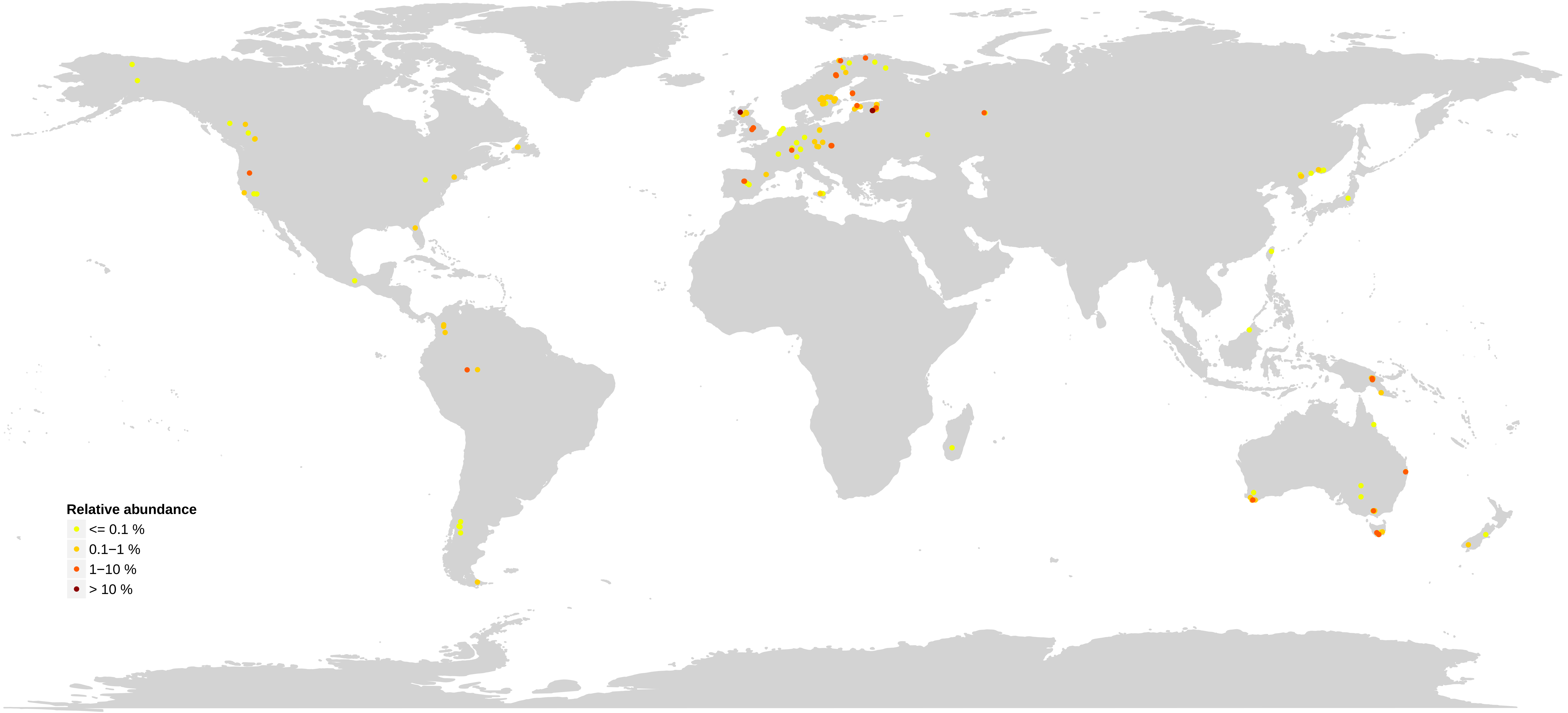

SH221016 Amanita rubescens

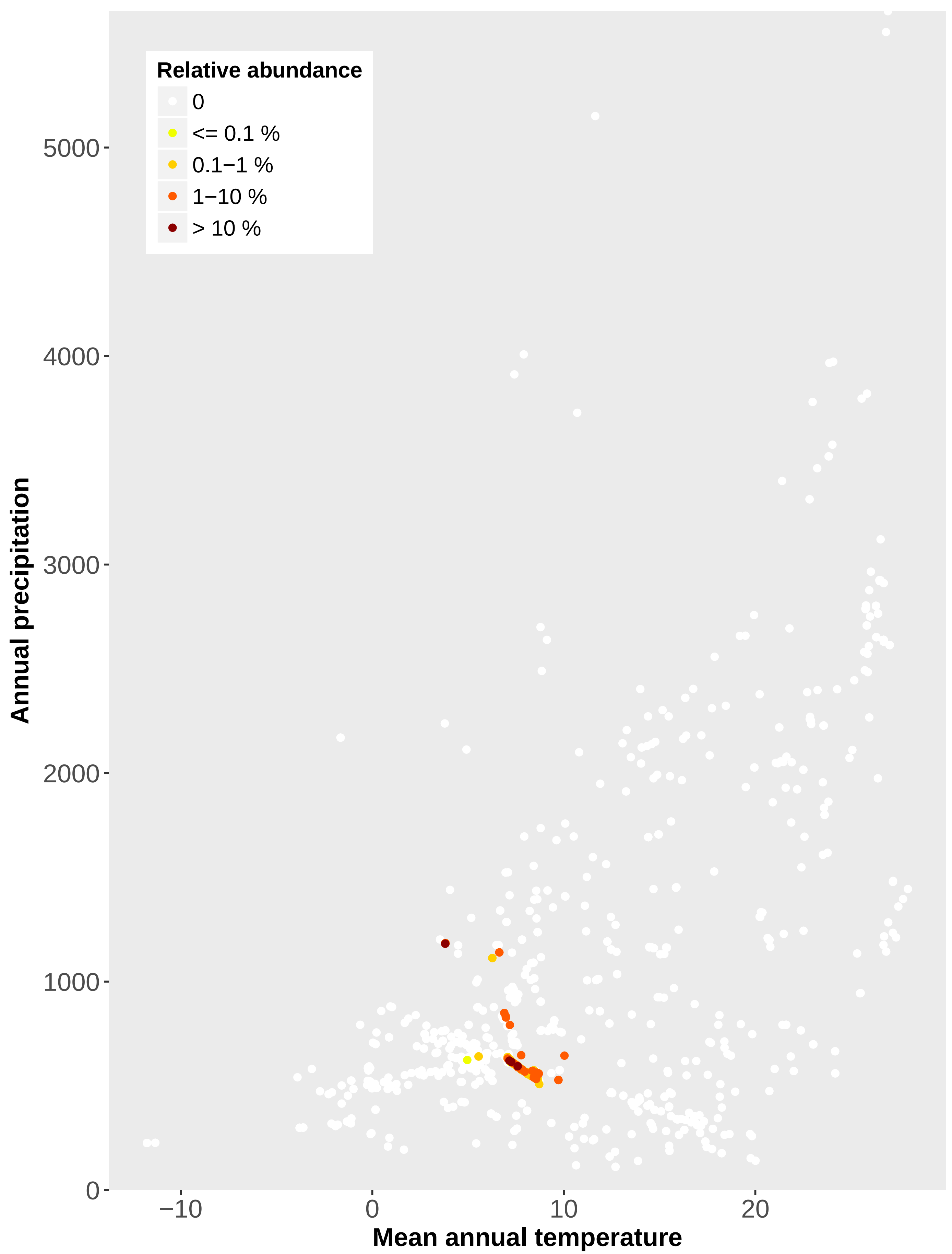

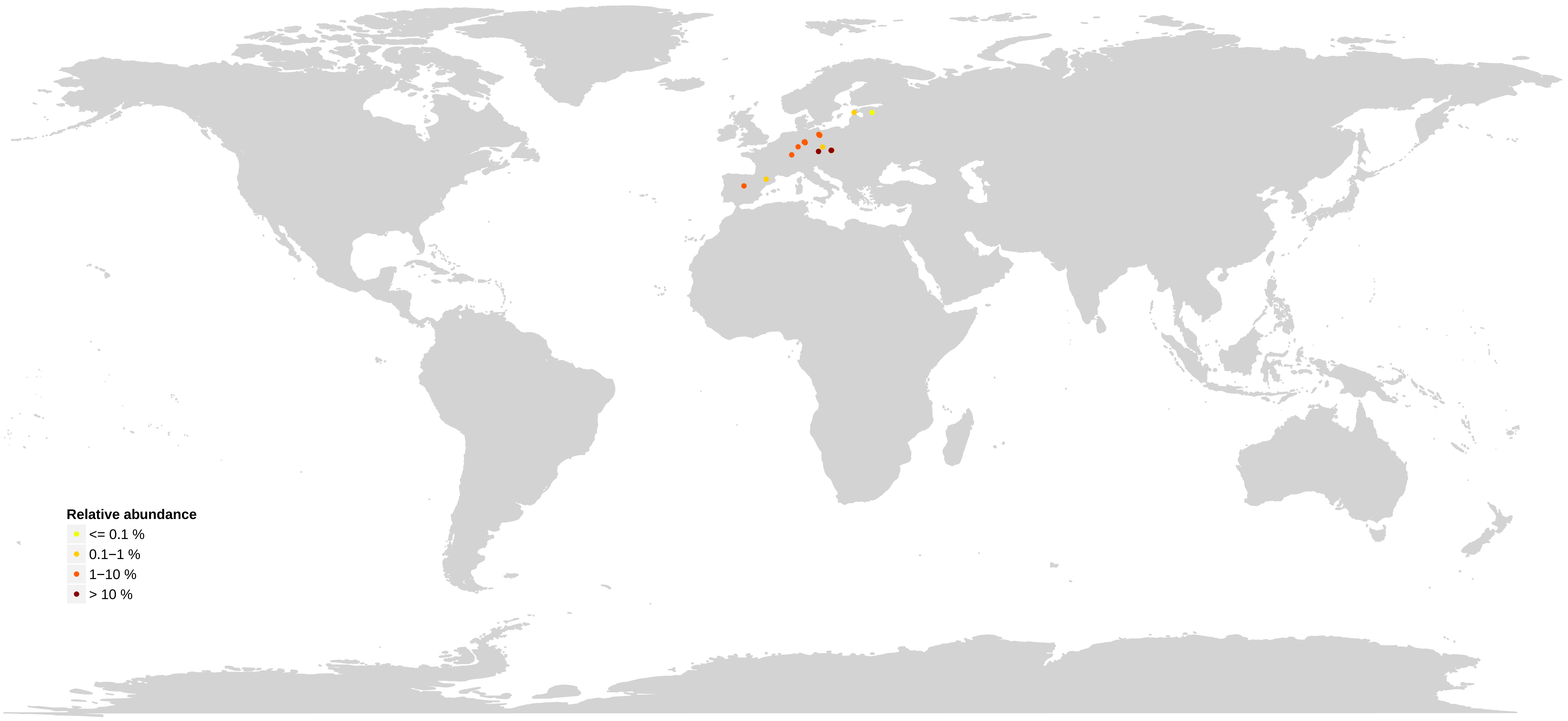

SH217001 Oidiodendron sp

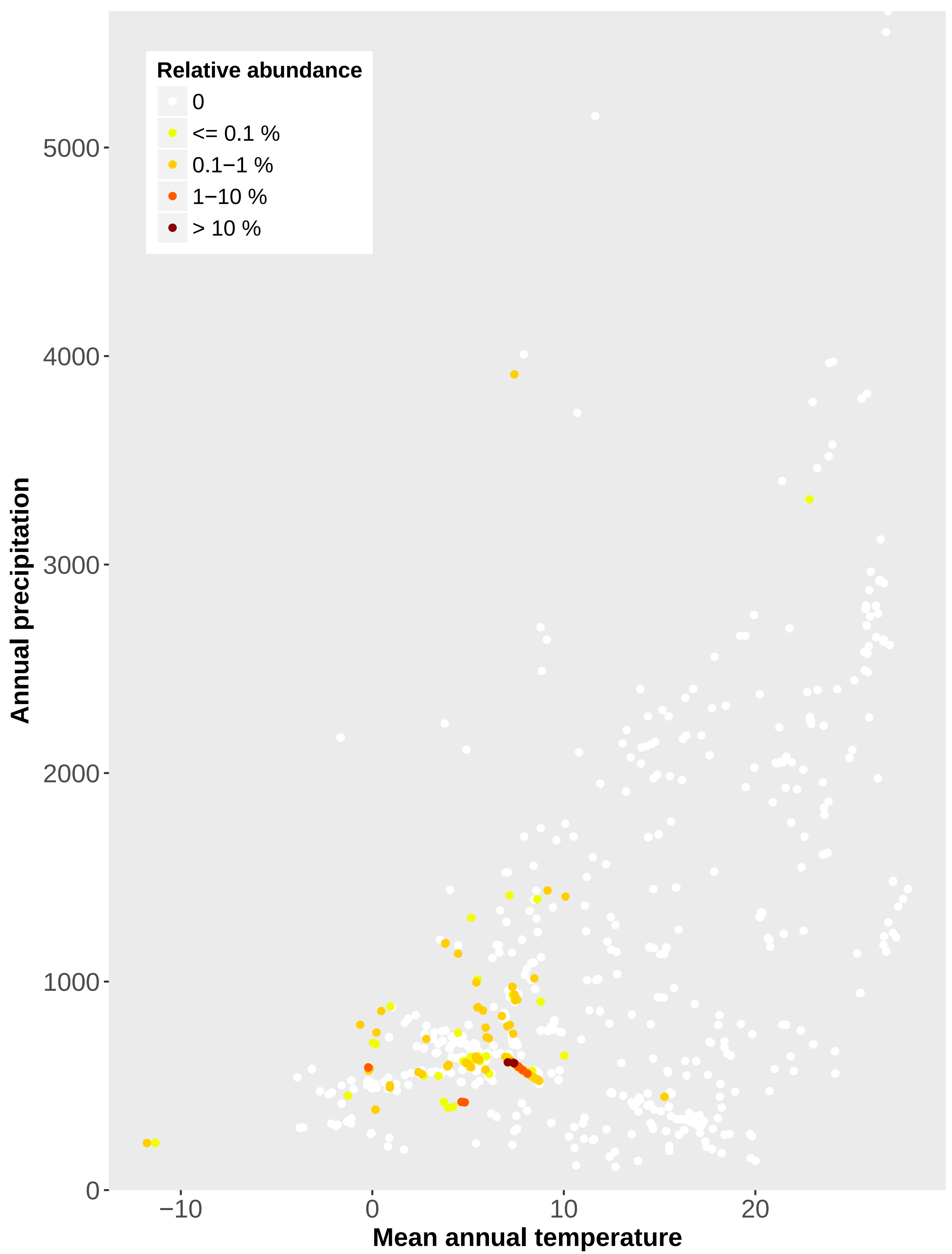

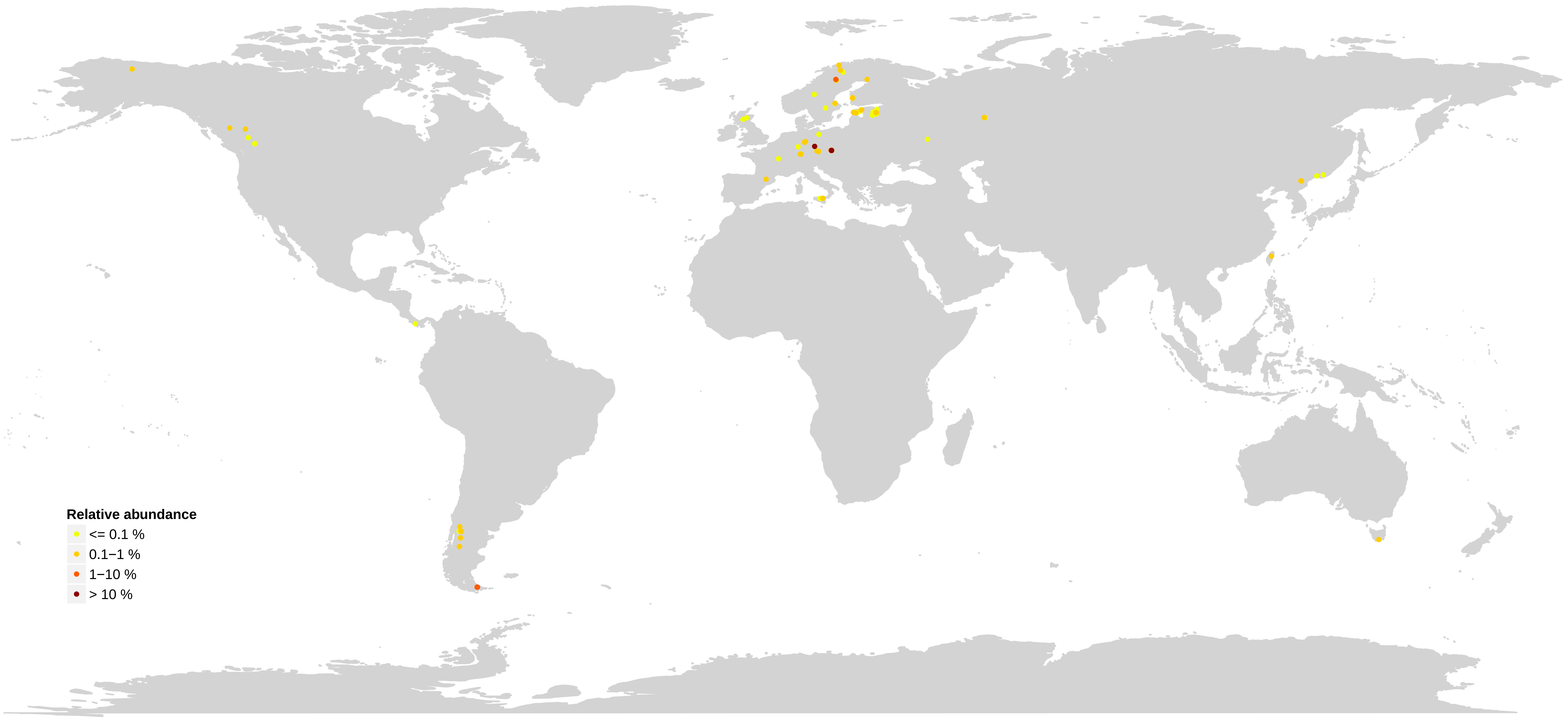

SH184181 Sporormiaceae sp

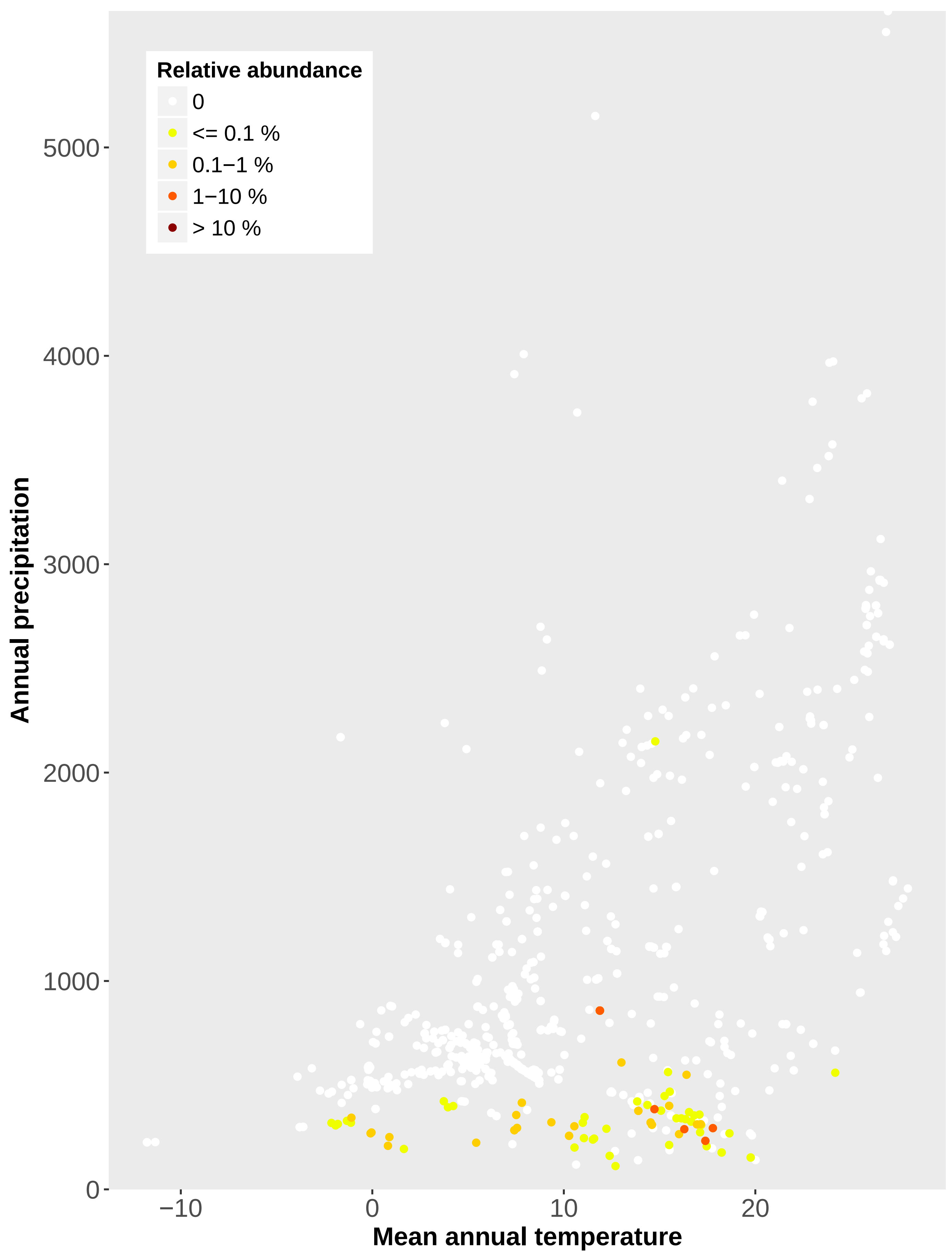

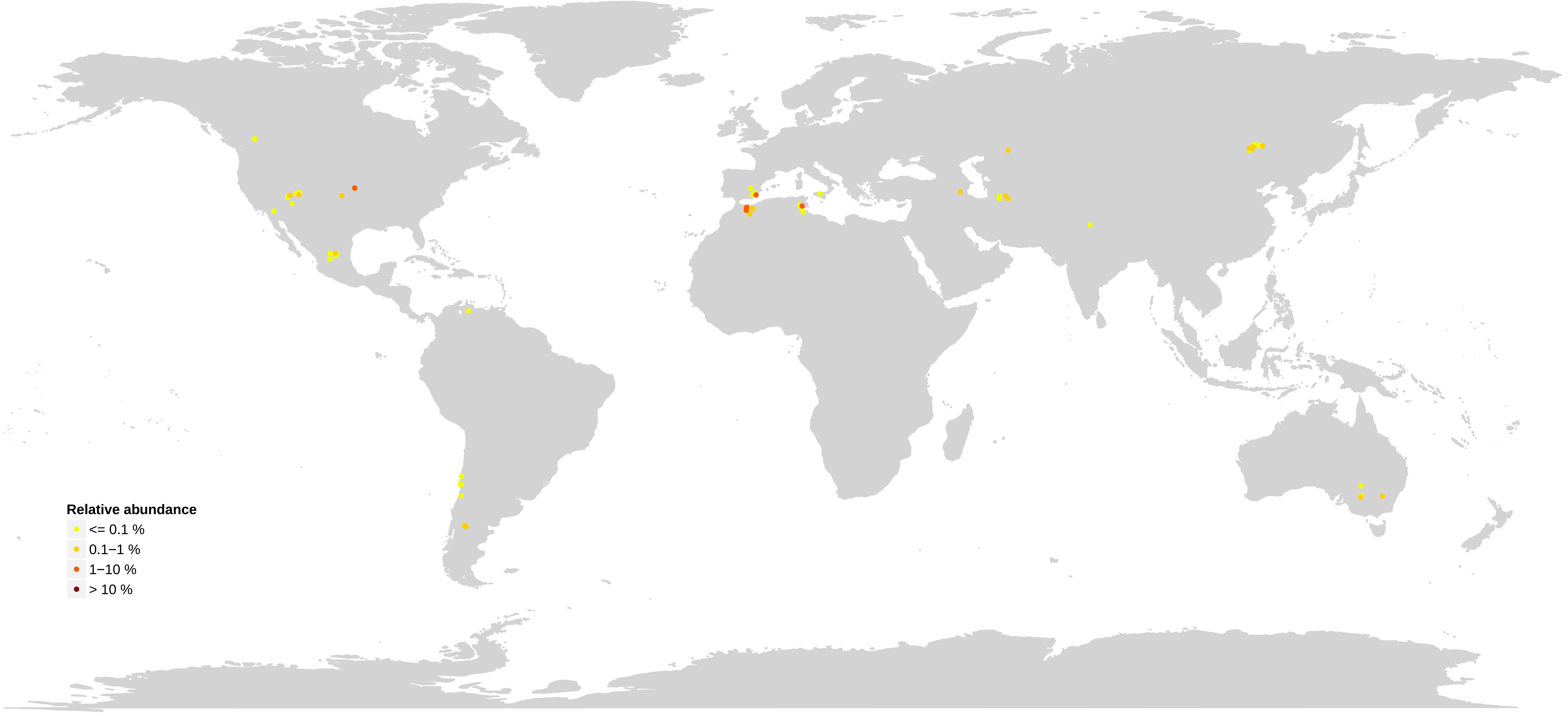

SH197766 Tylospora asterophora

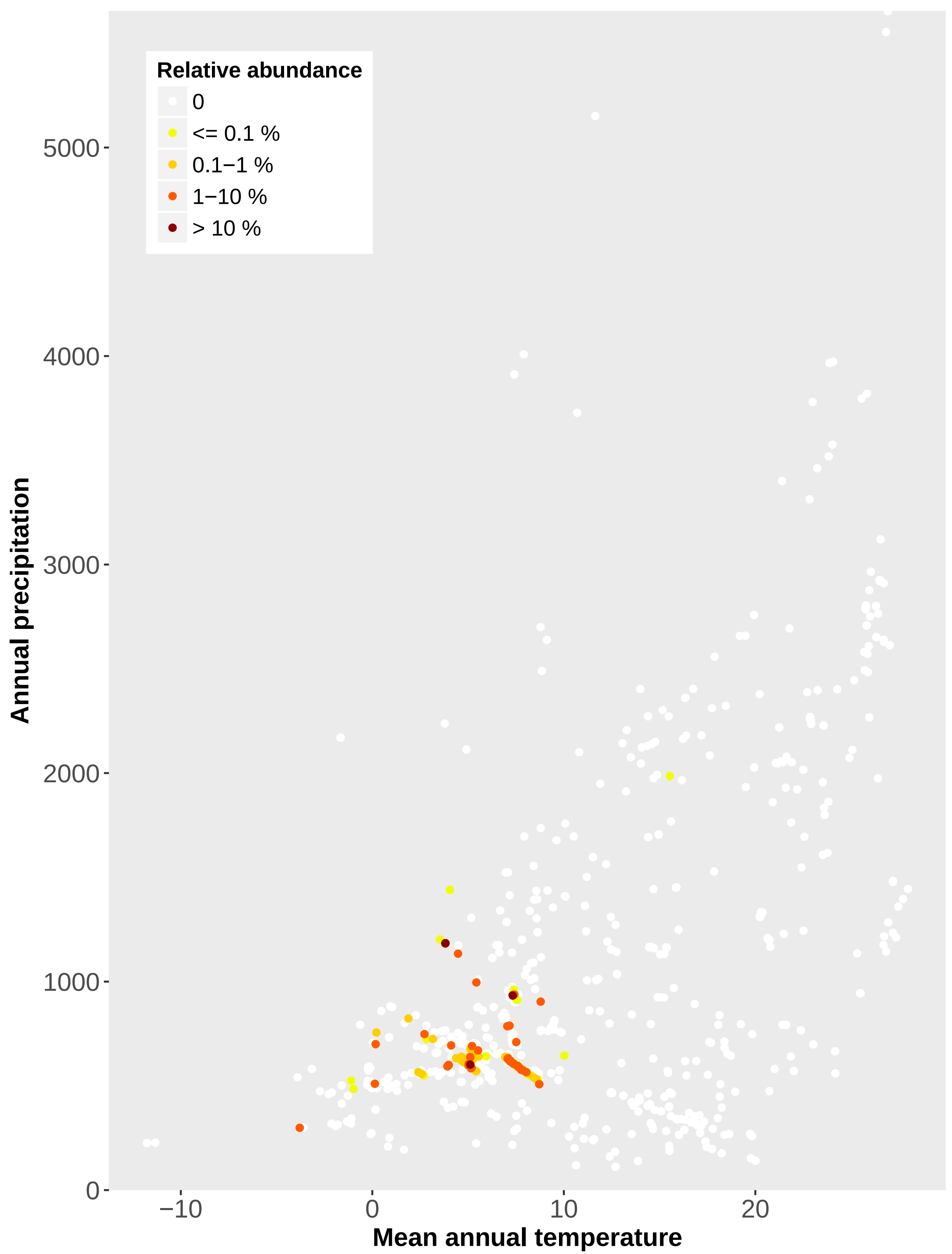

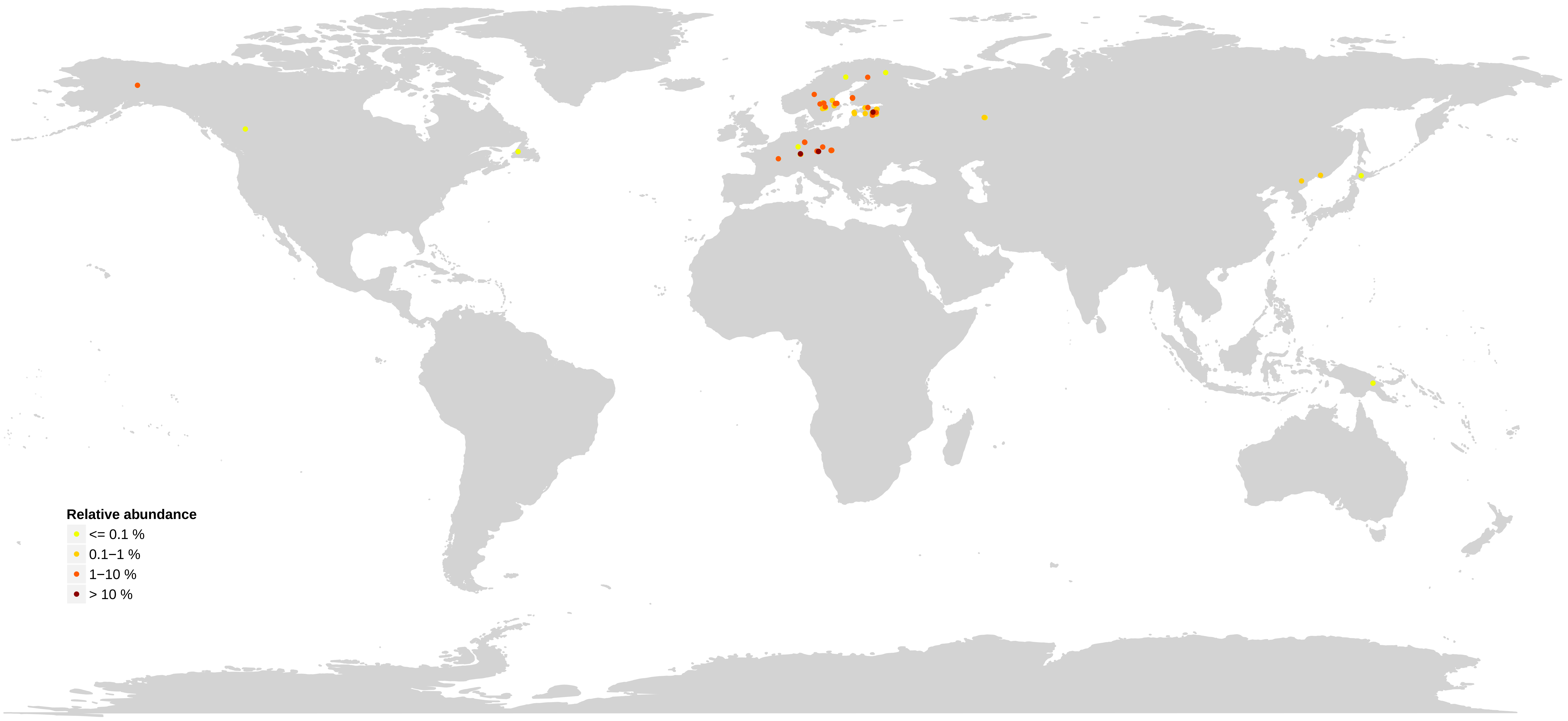

SH186050 Hydnodontaceae sp

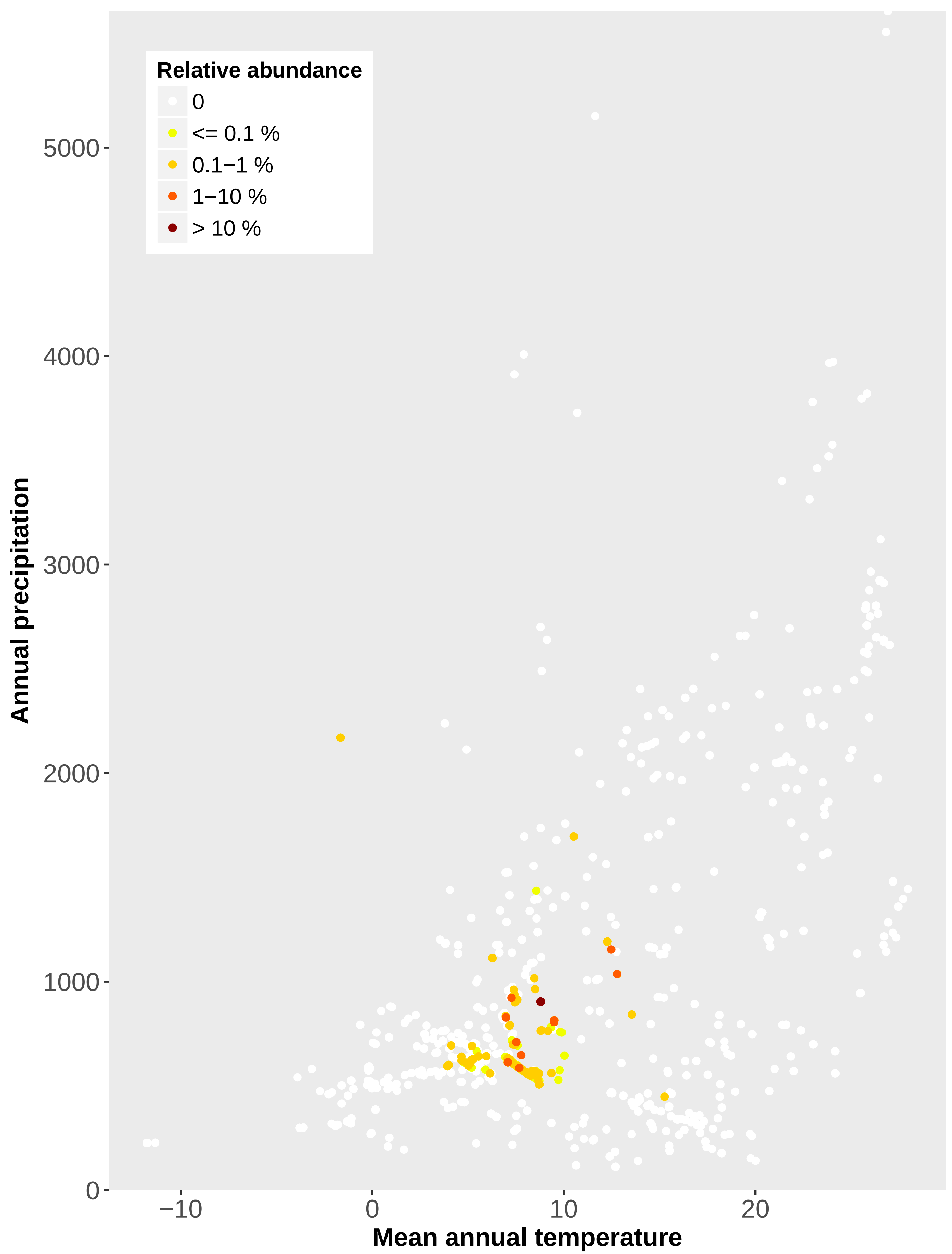

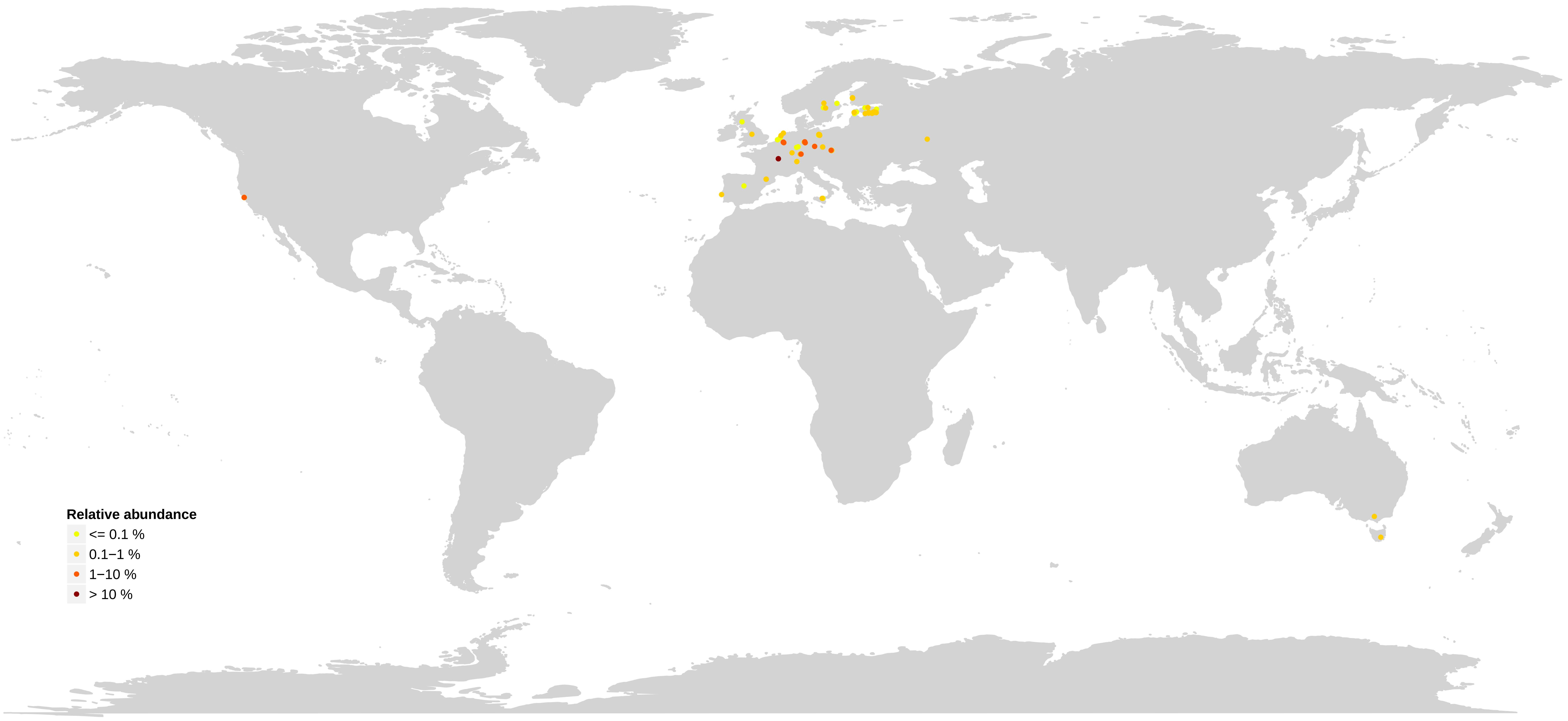

SH197643 Exophiala equina

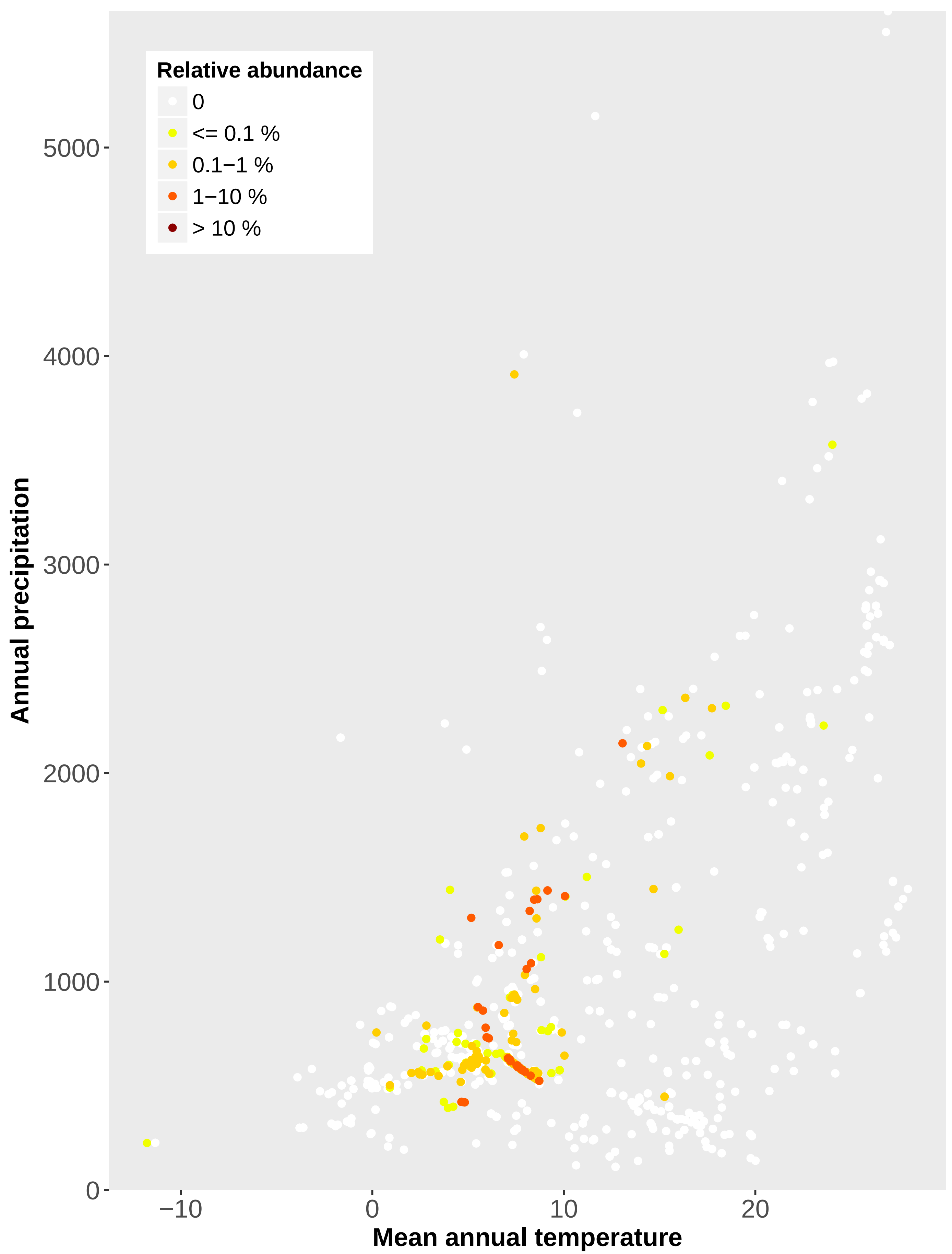

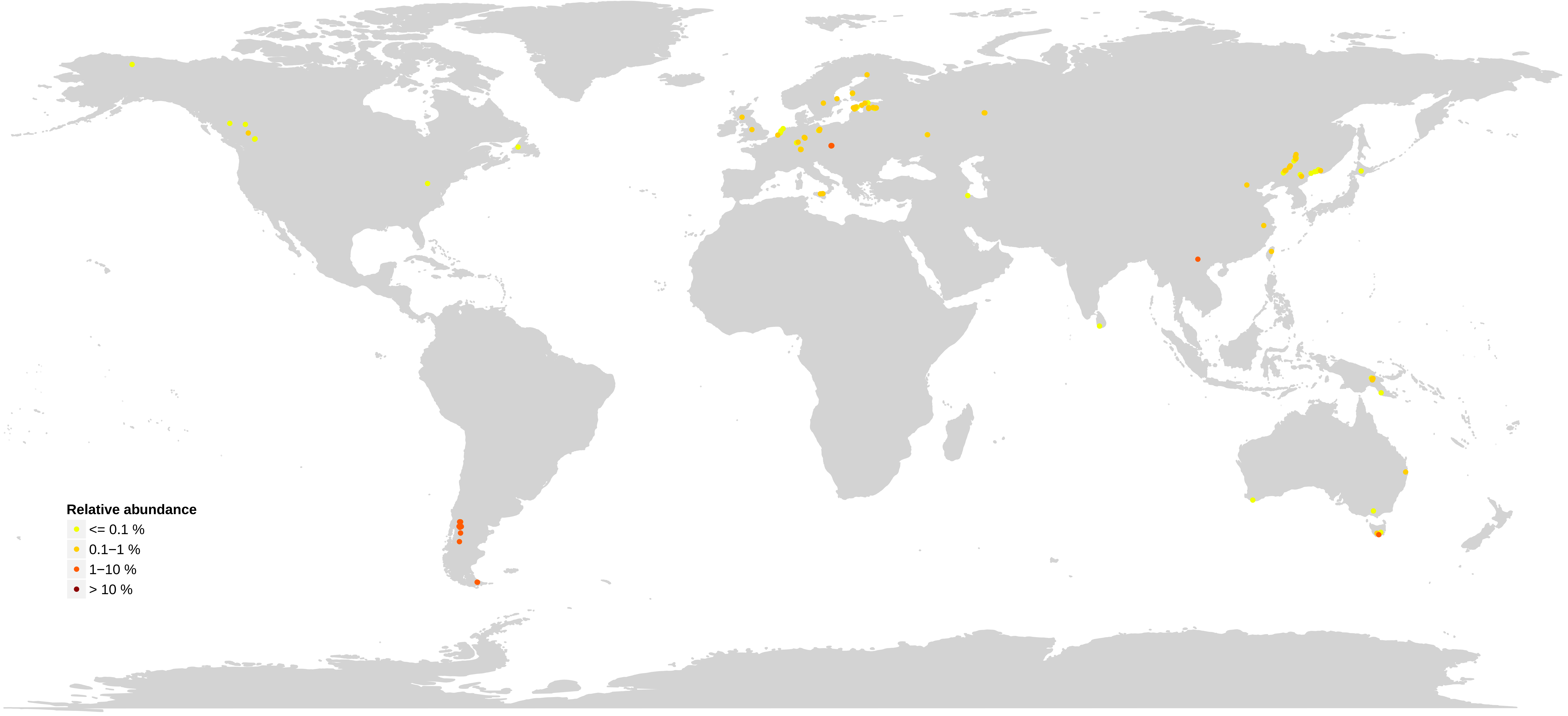

SH008140 Fungi sp

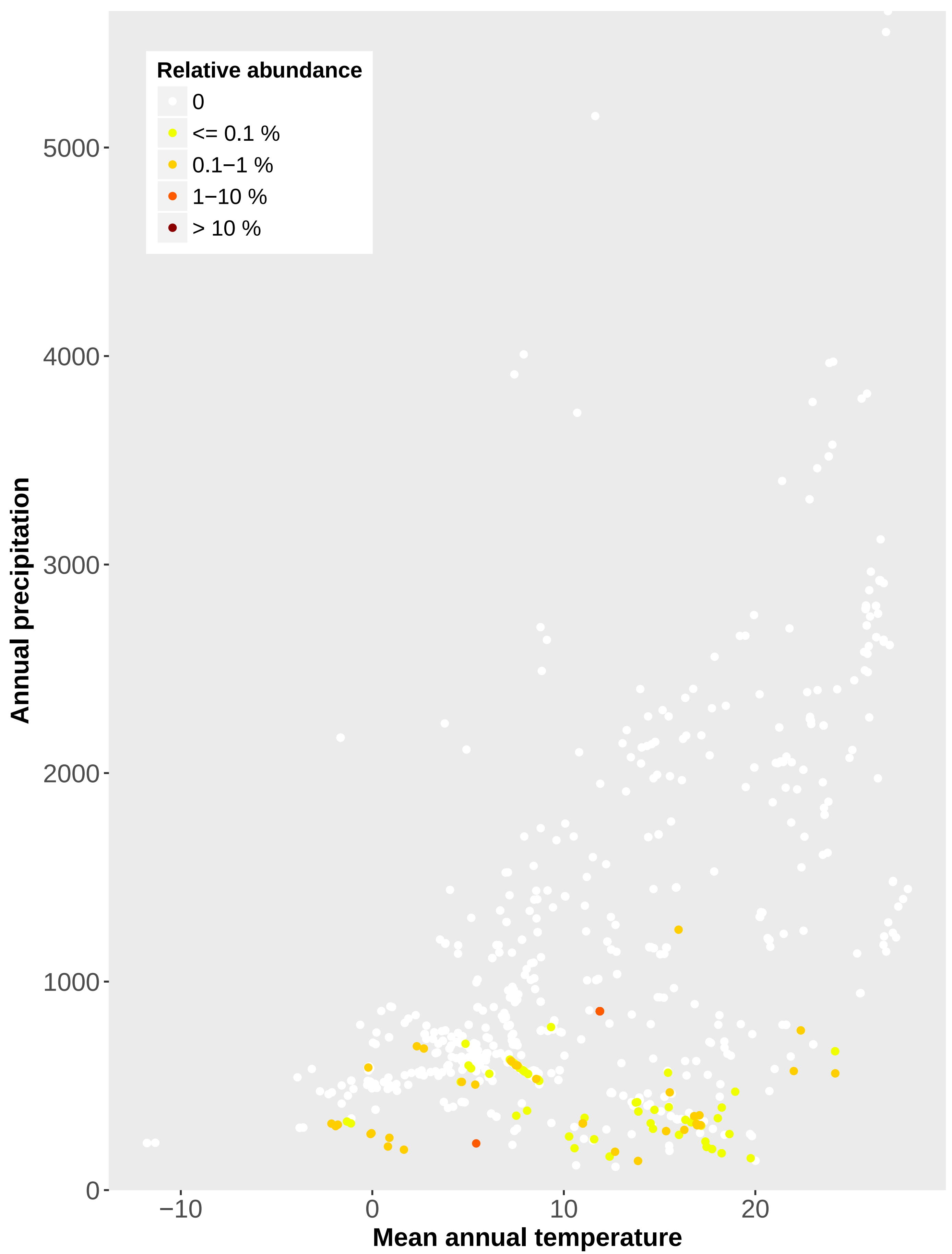

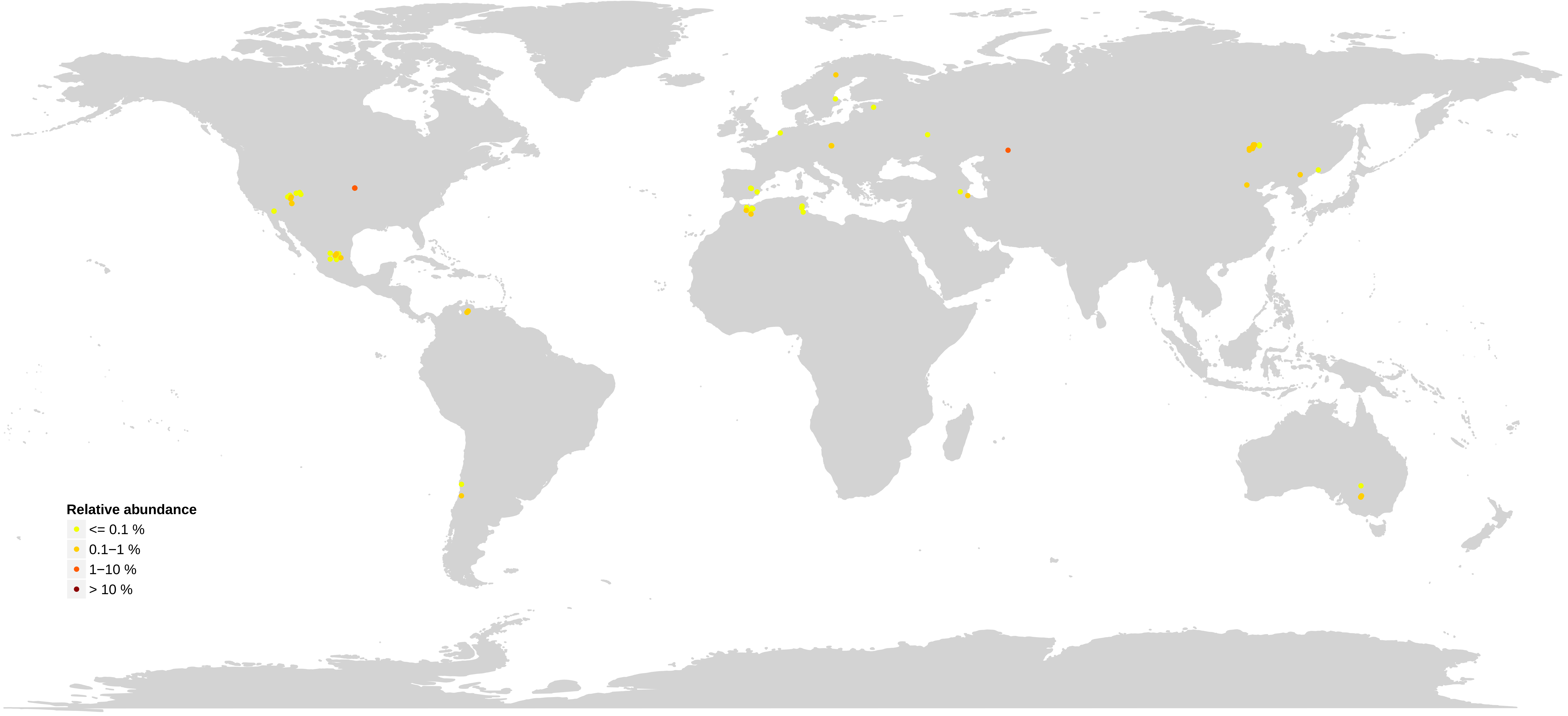

SH196091 Umbelopsis isabellina

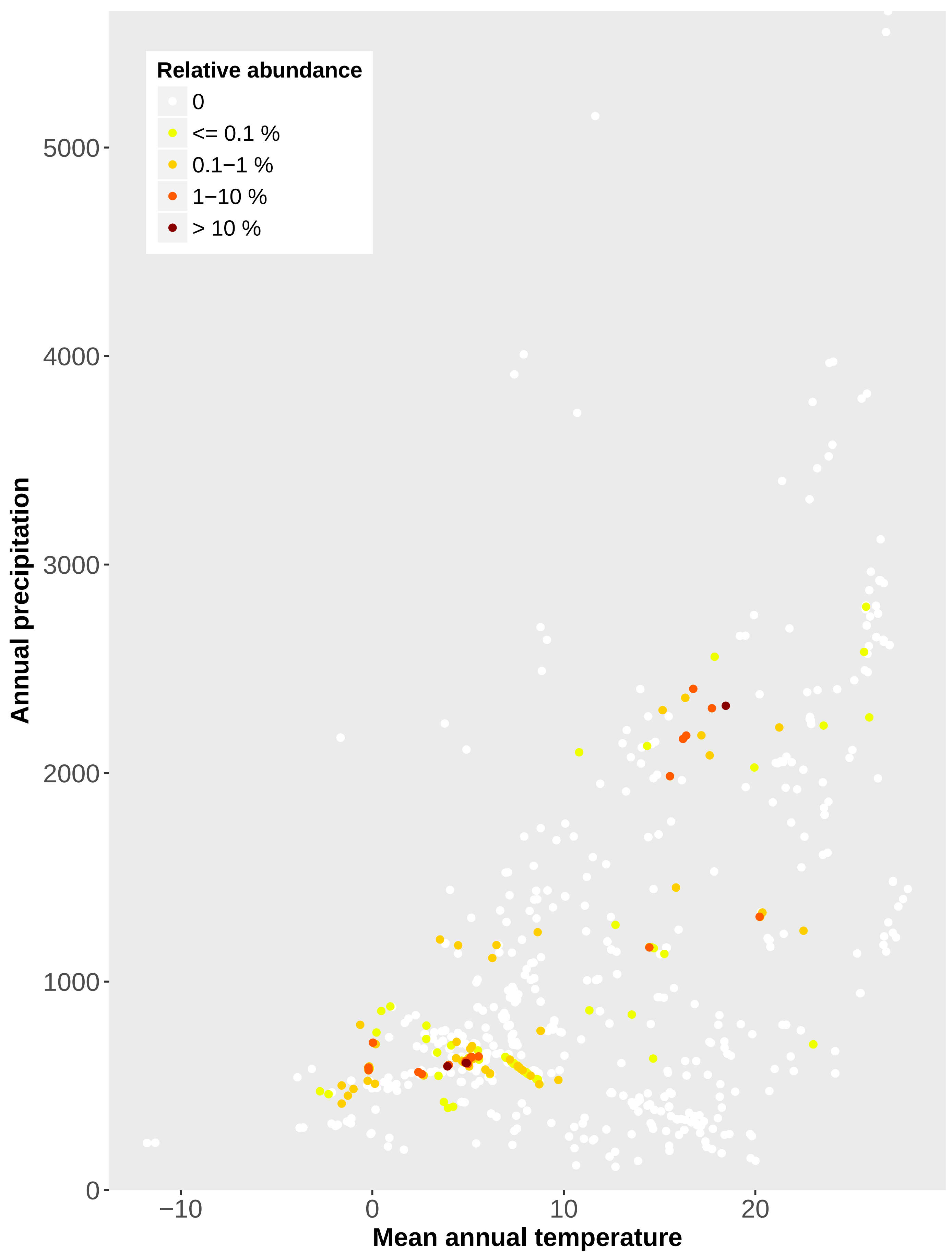

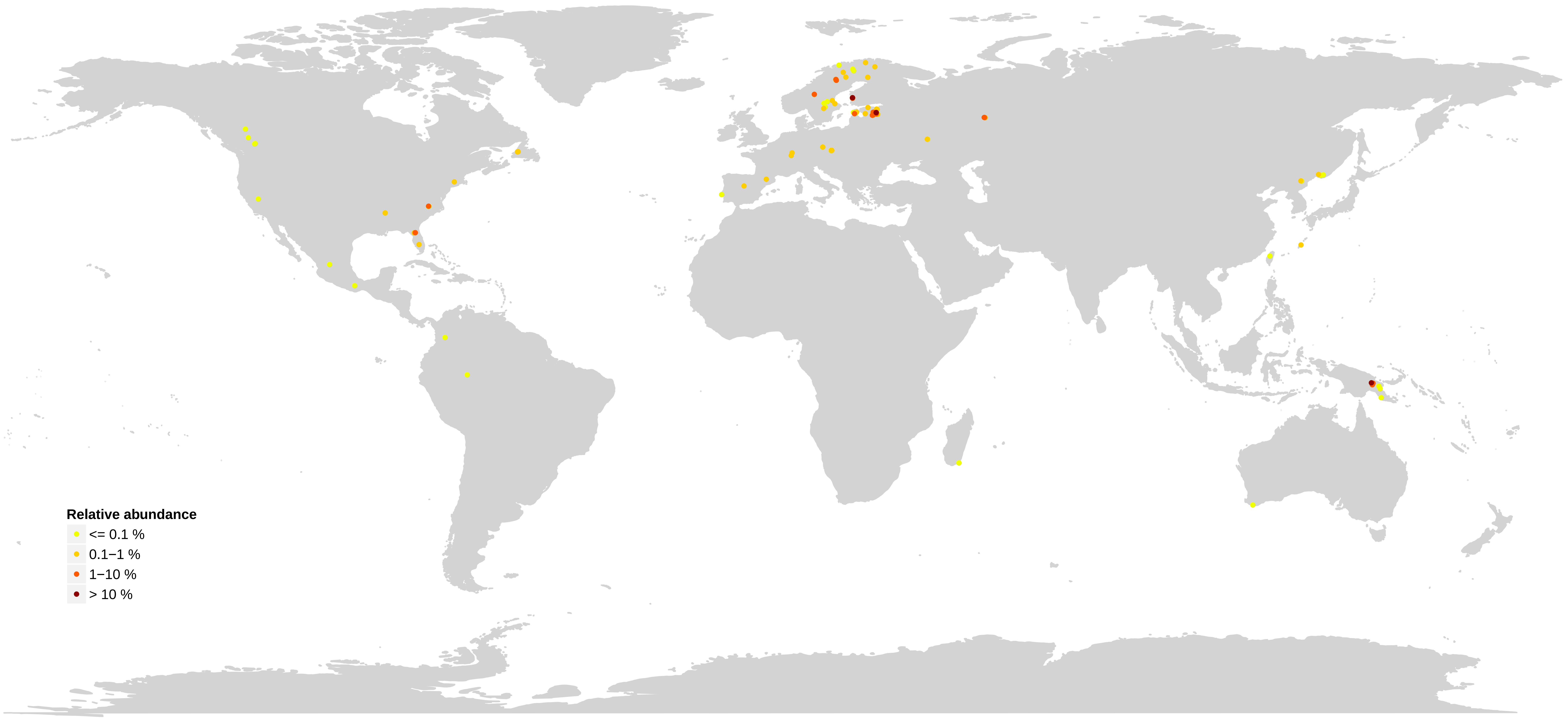

SH183186 Herpotrichiellaceae sp

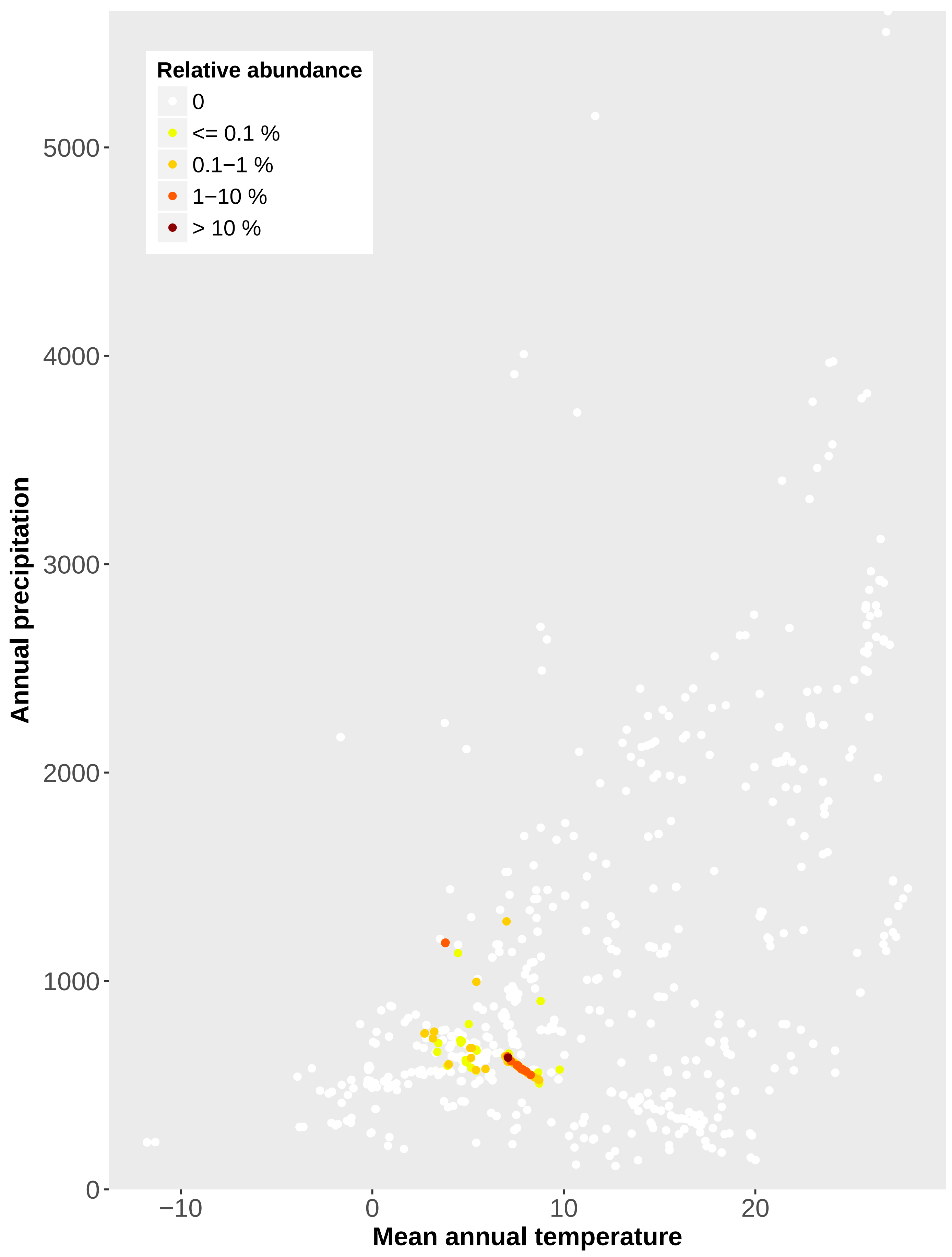

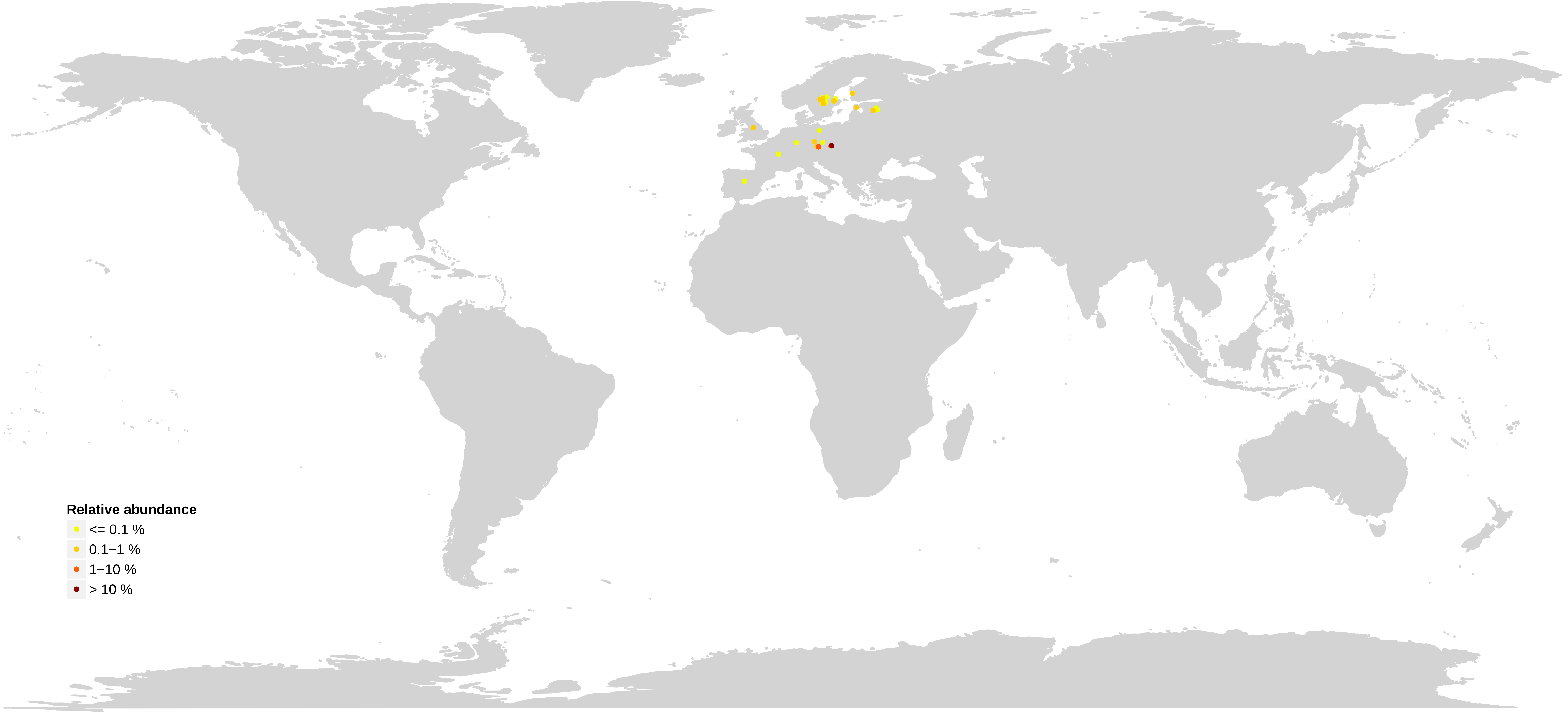

SH426644 Meliniomyces bicolor

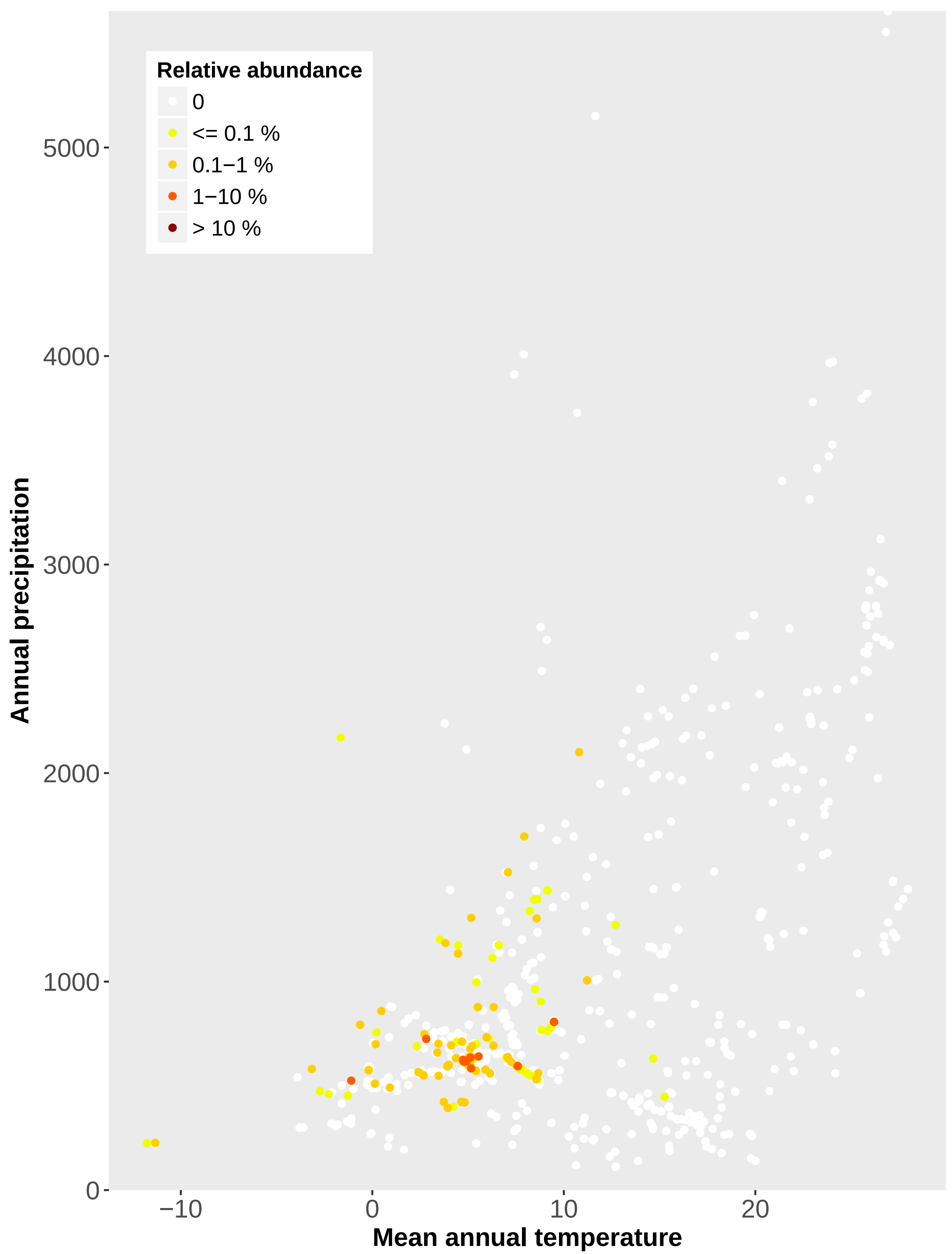

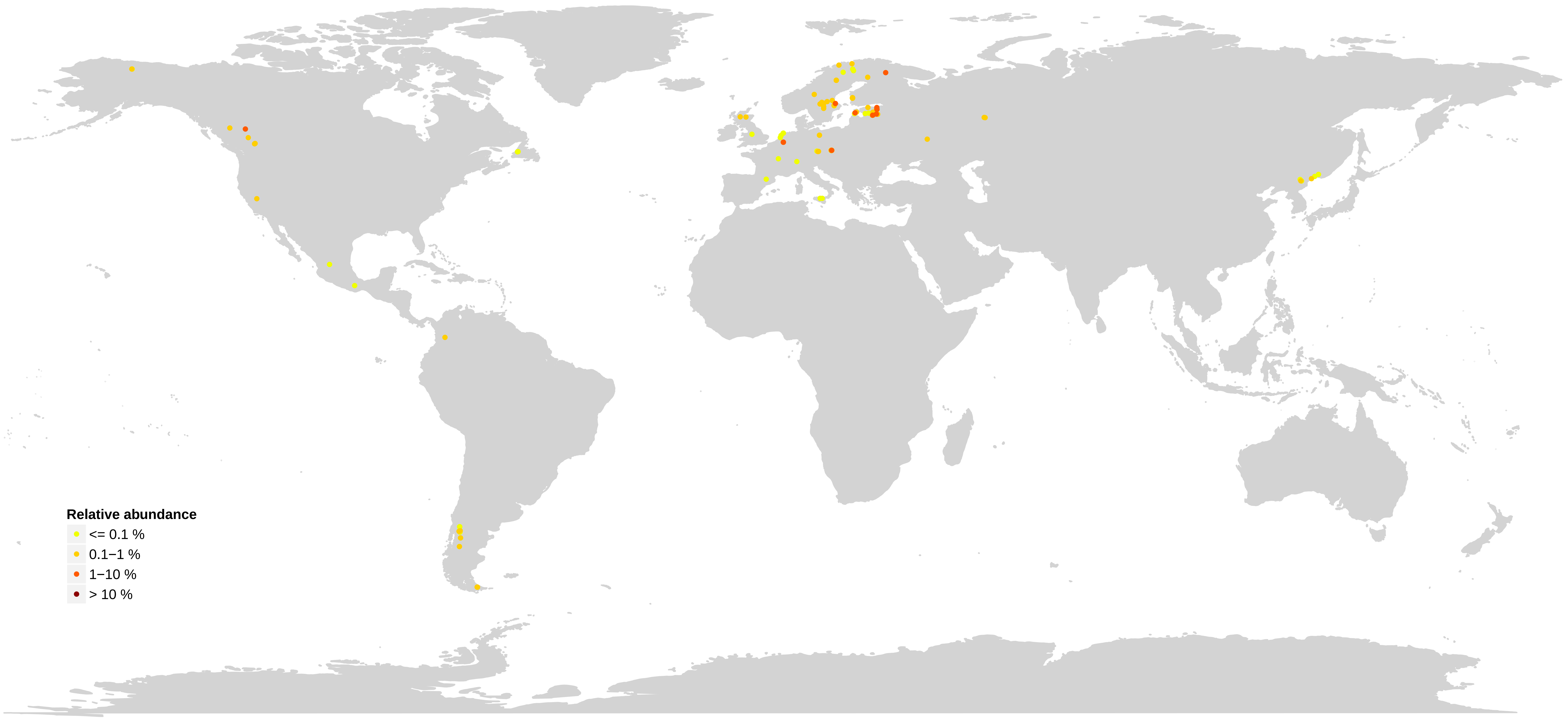

SH207227 Phallus impudicus

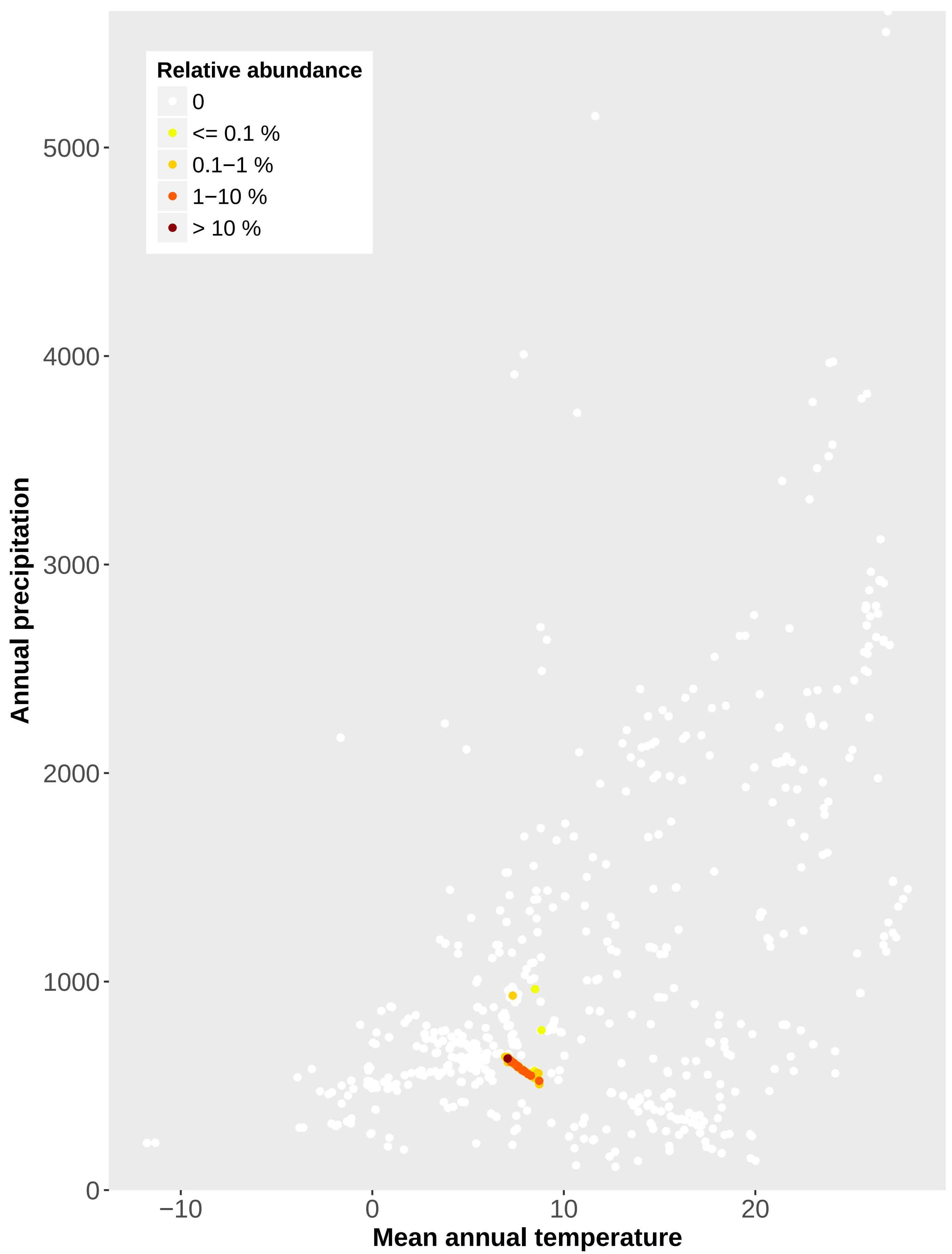

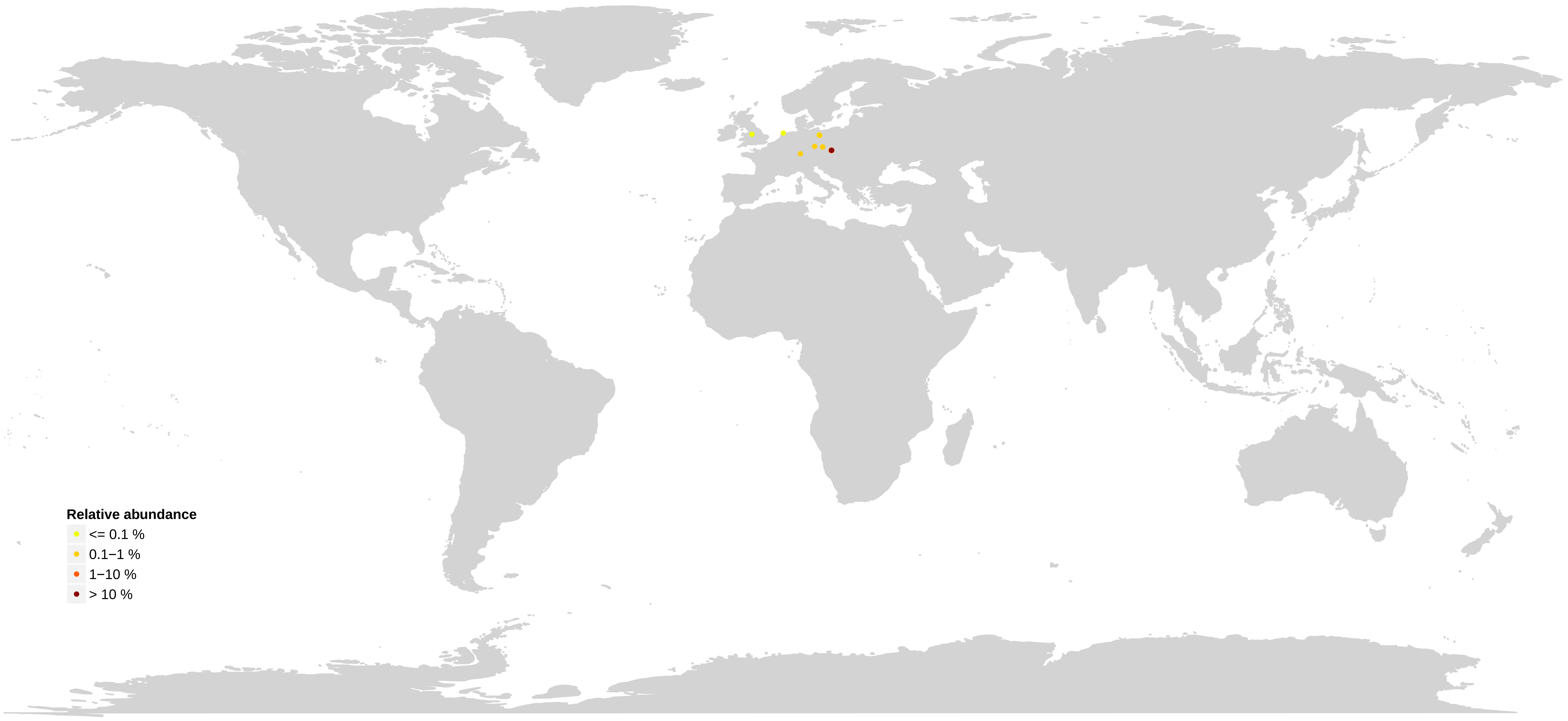

SH199400 Penicillium bialowiezense

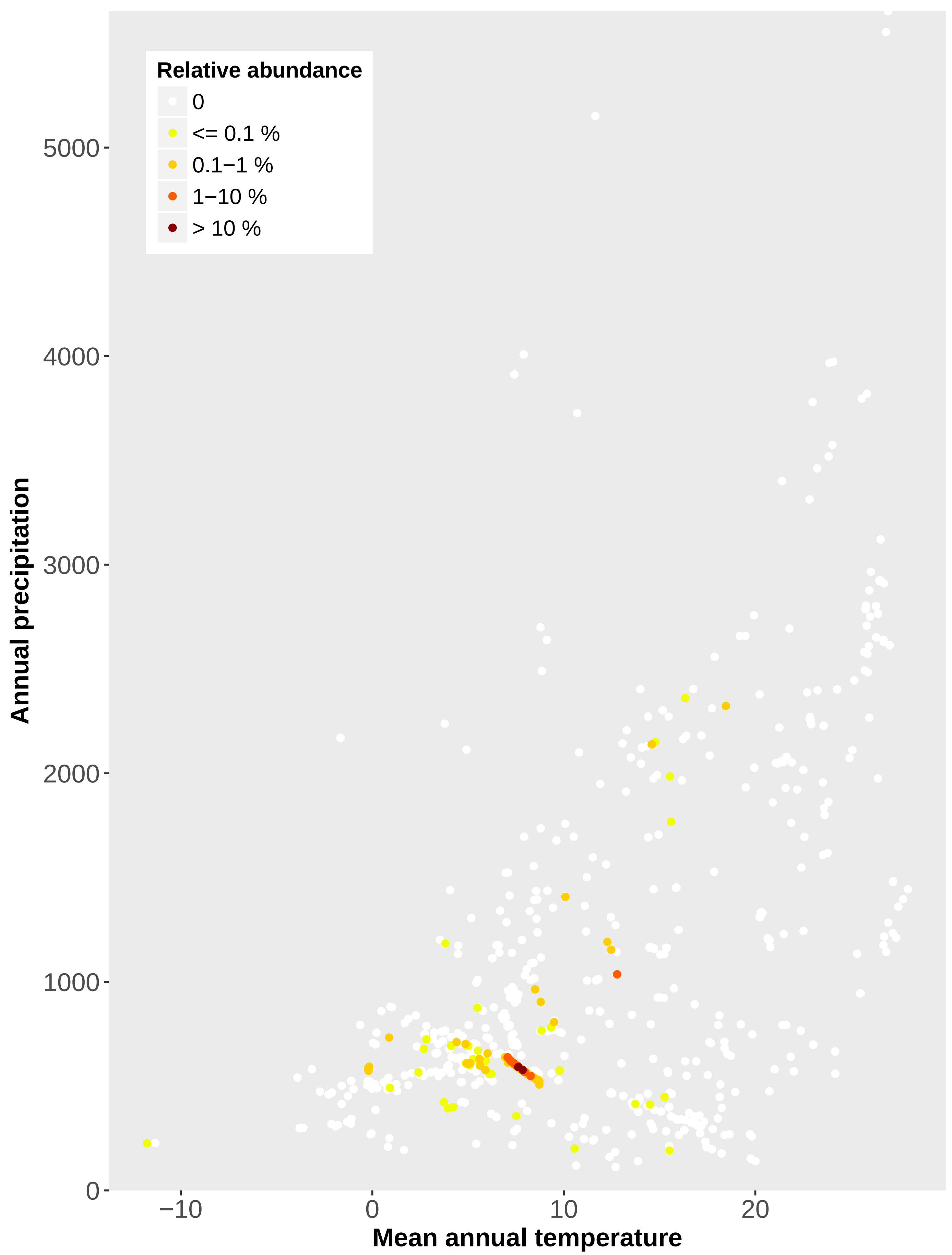

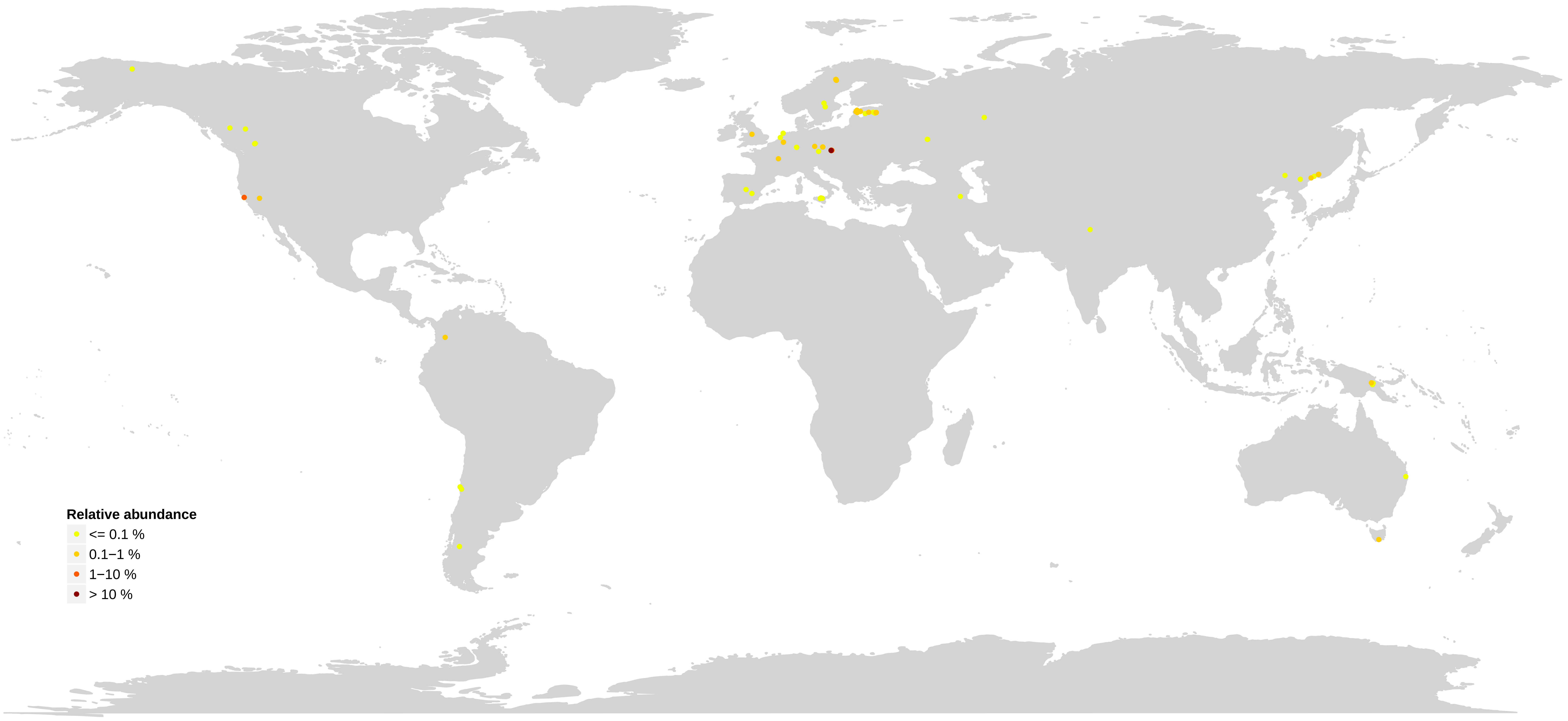

SH216993 Oidiodendron echinulatum

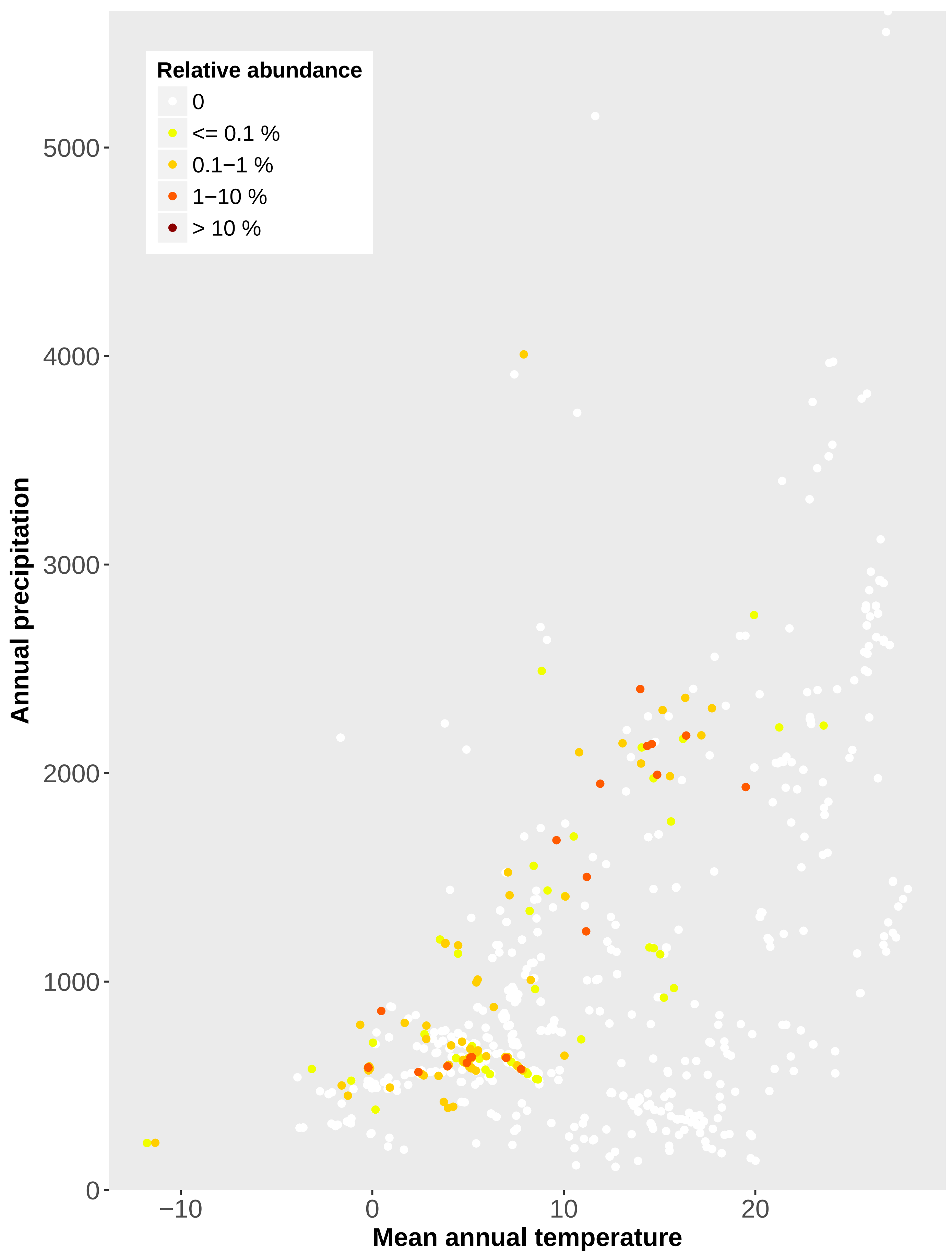

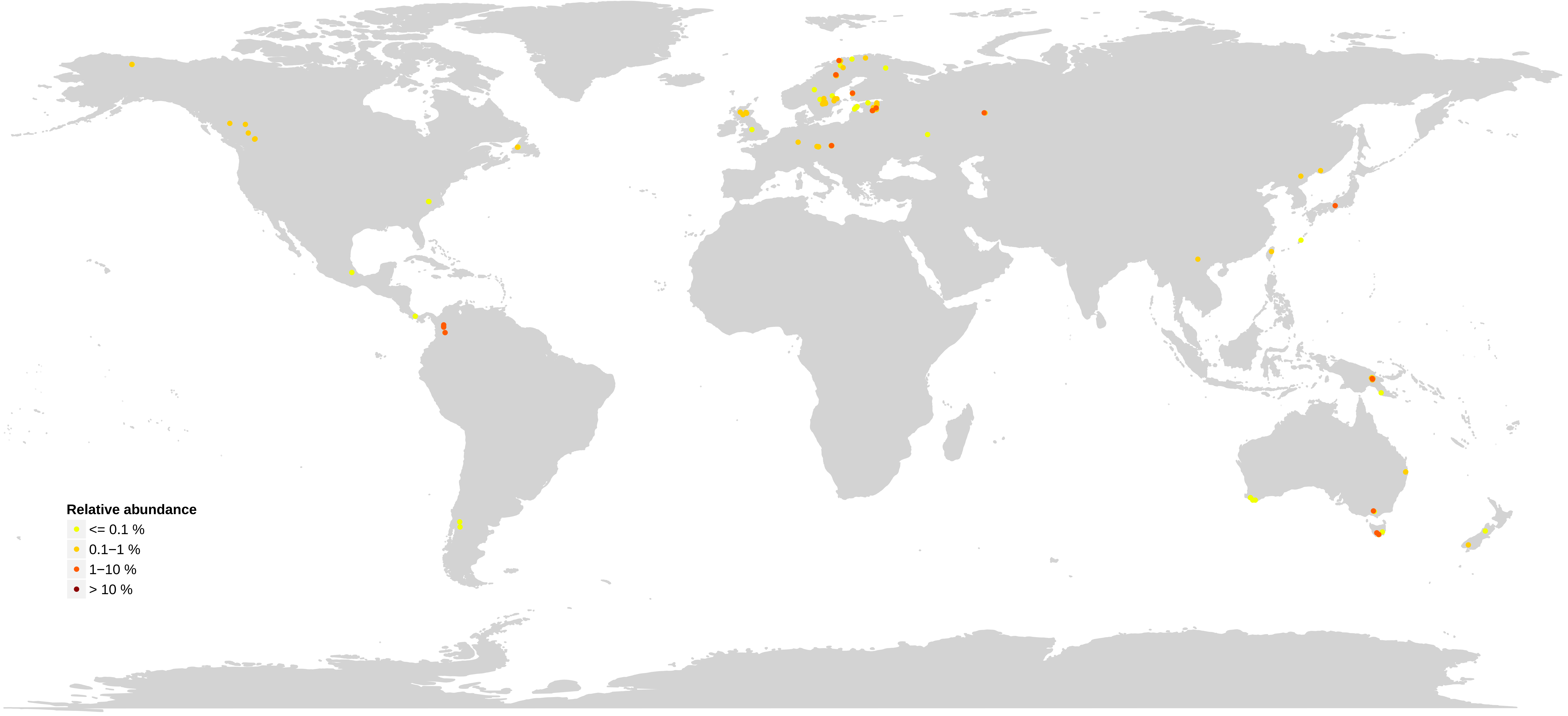

SH182493 Penicillium parviverrucosum

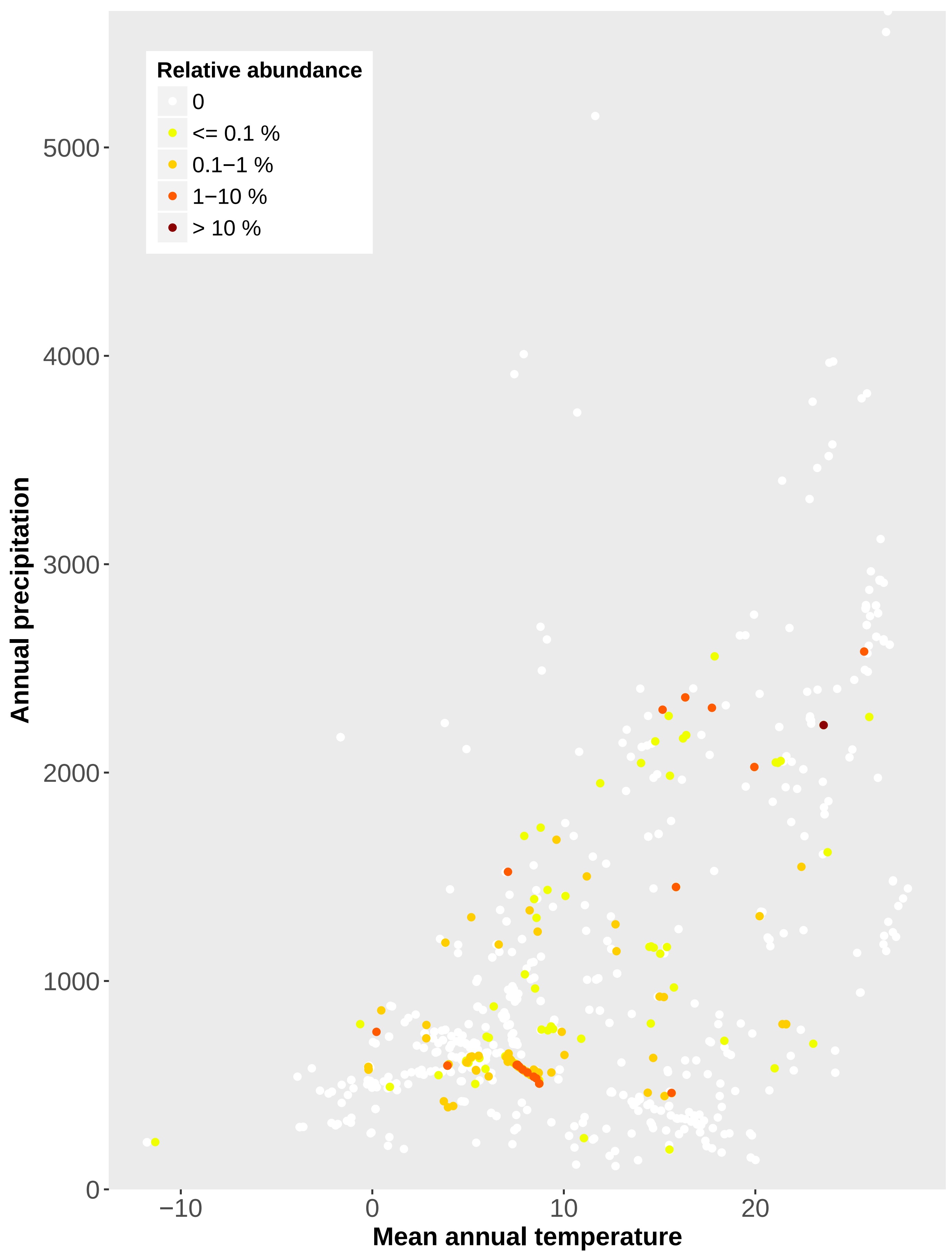

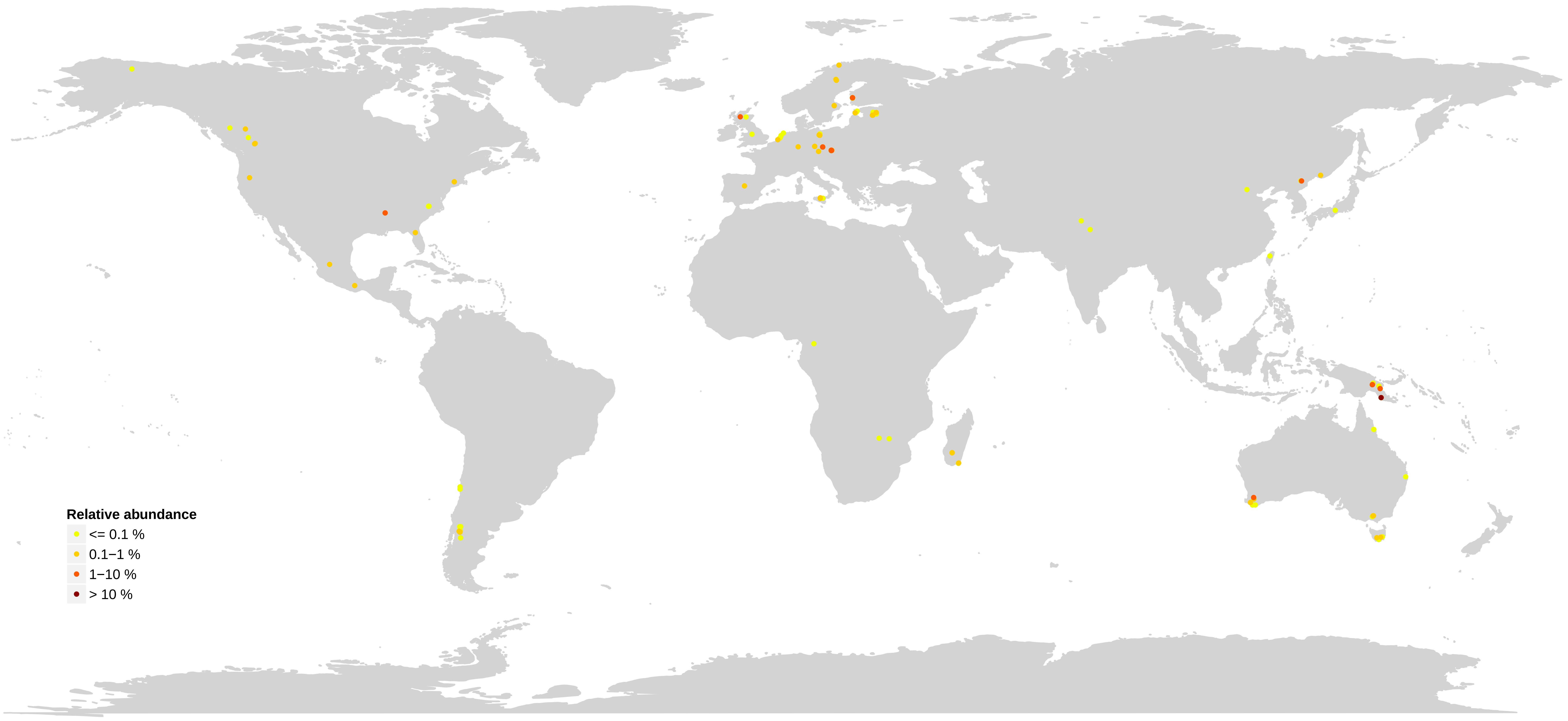

SH208301 Geminibasidium sp

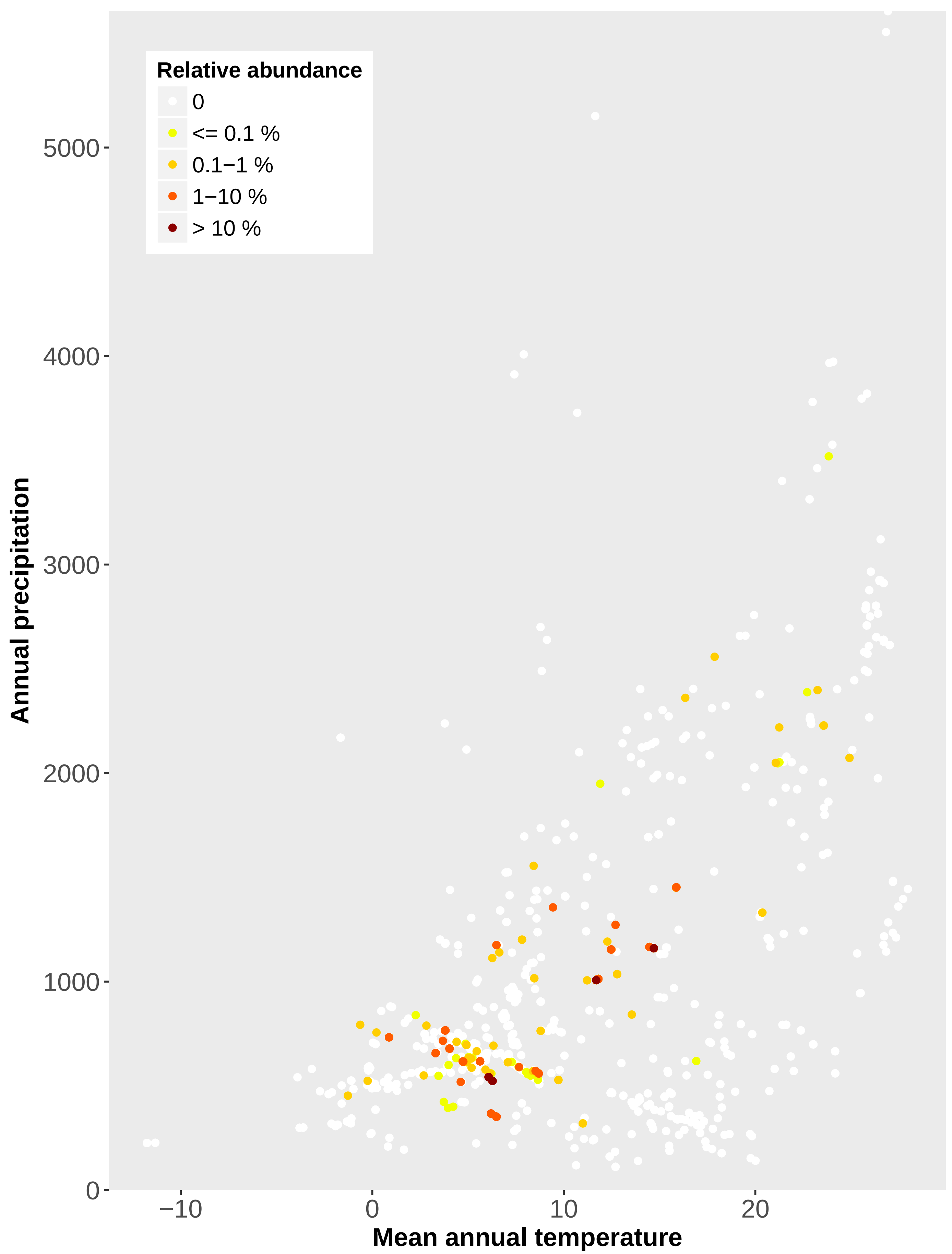

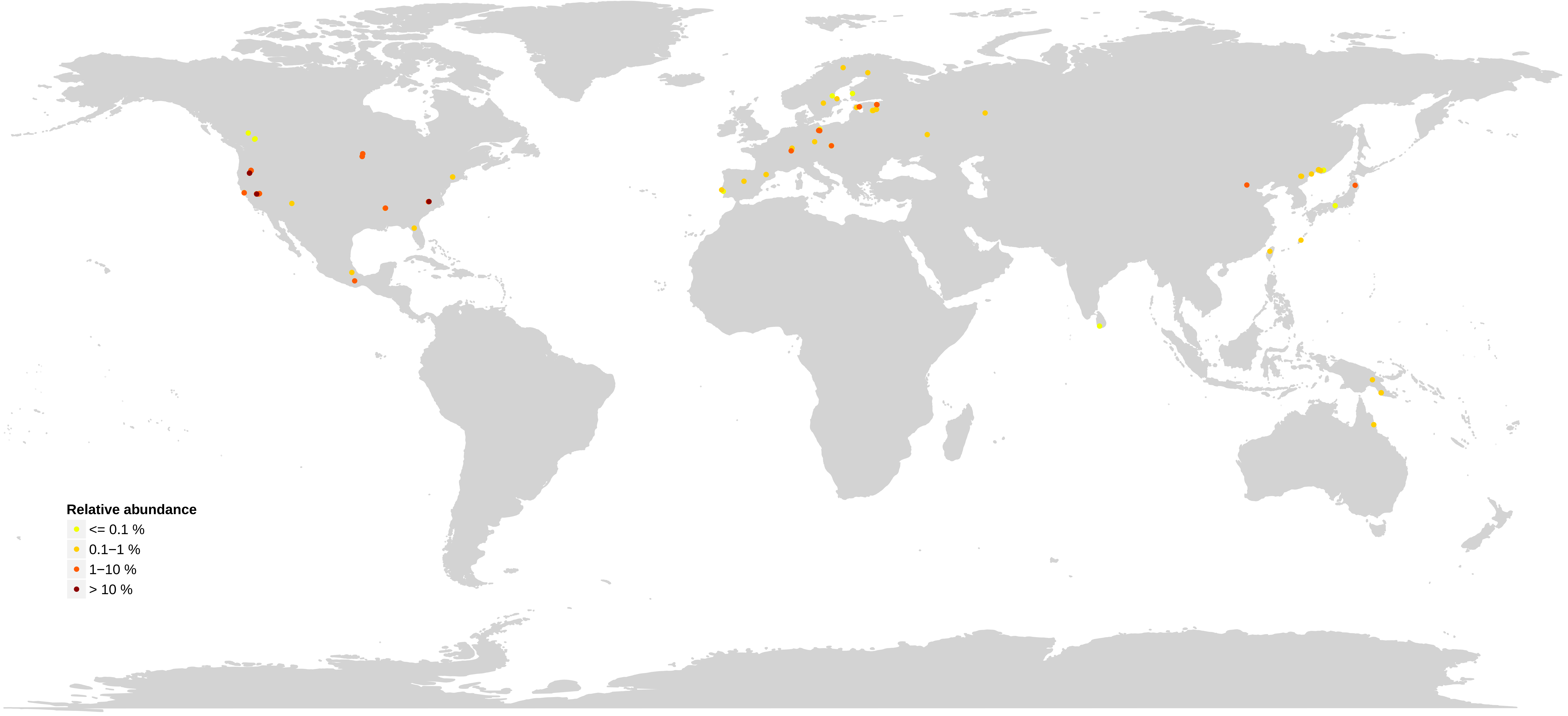

SH223367 Agaricales sp

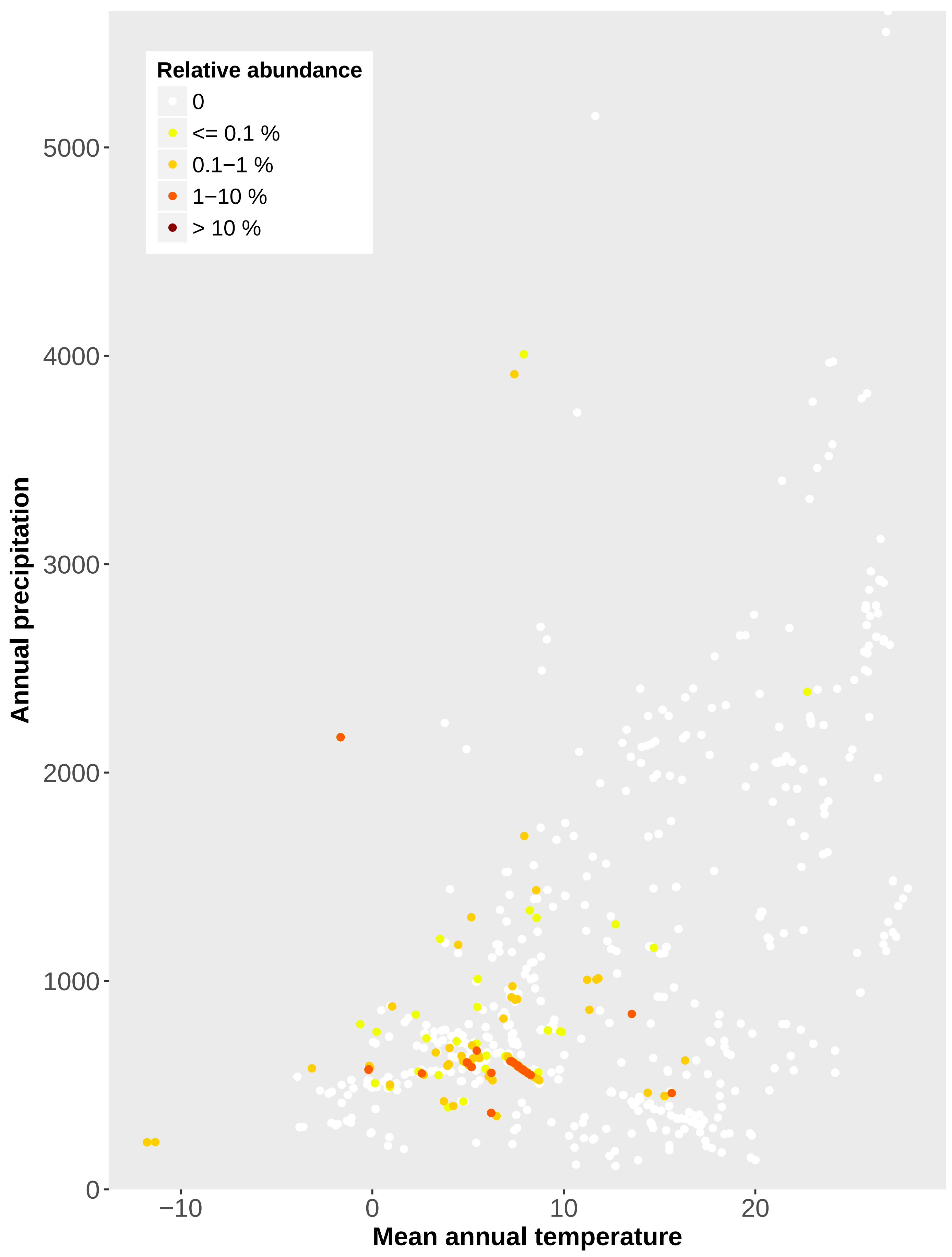

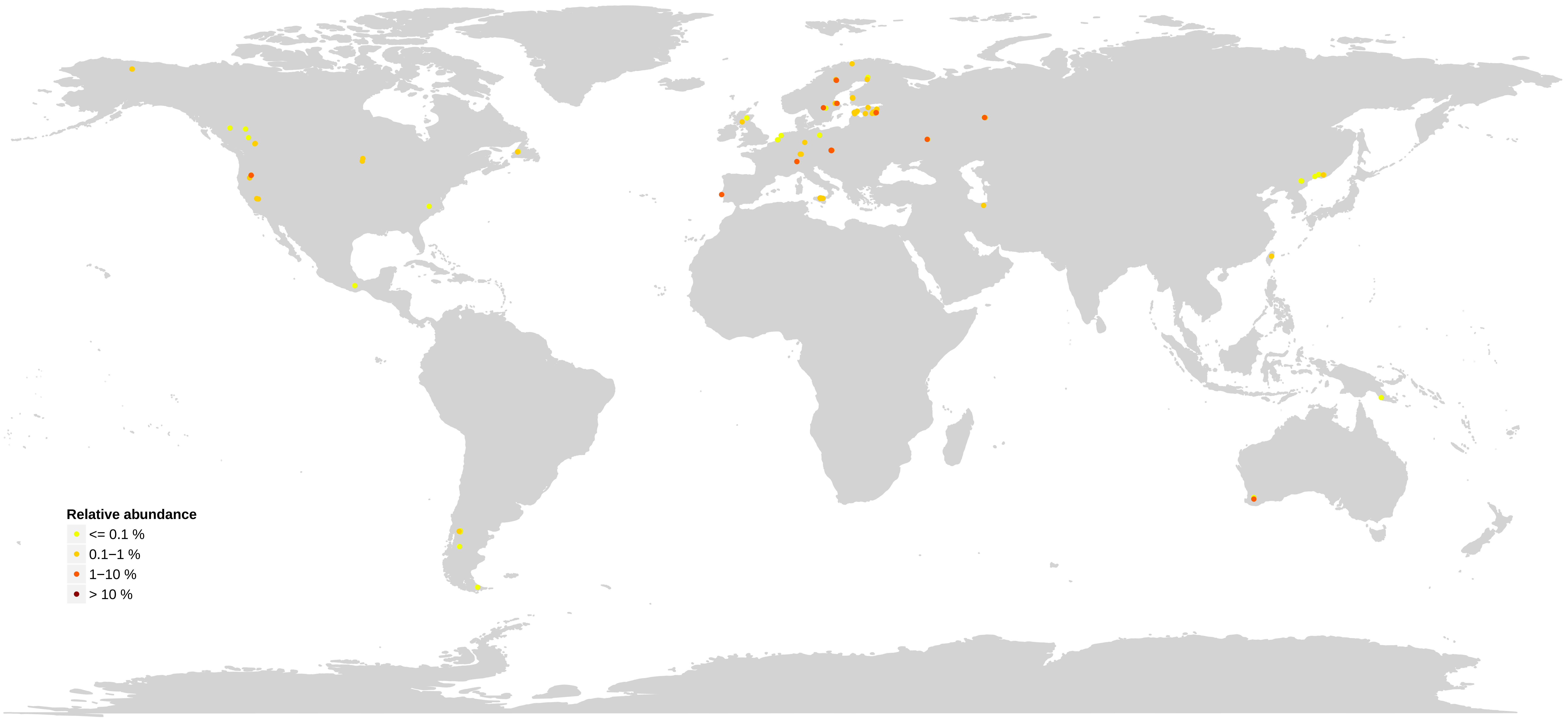

SH184178 Ascomycota sp

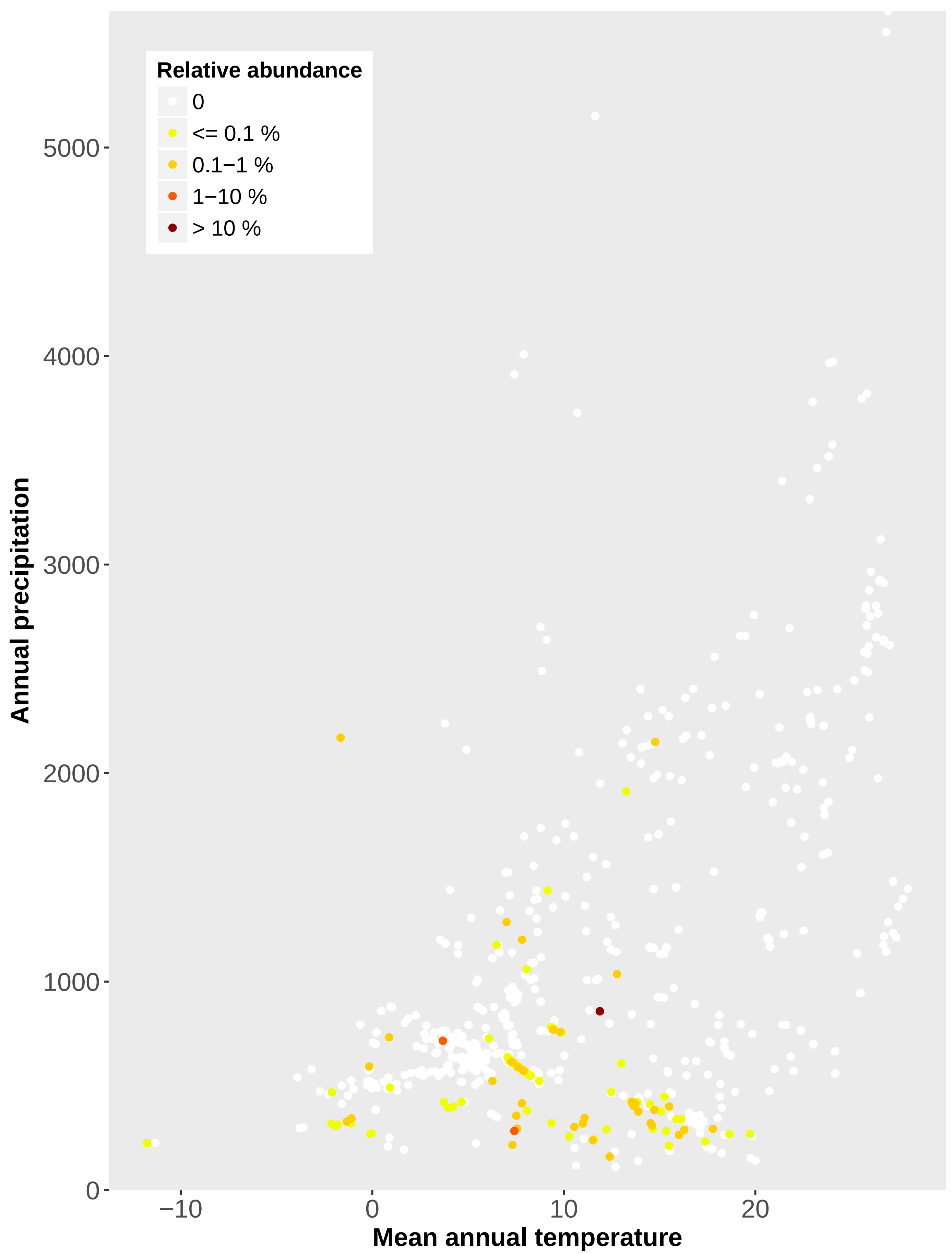

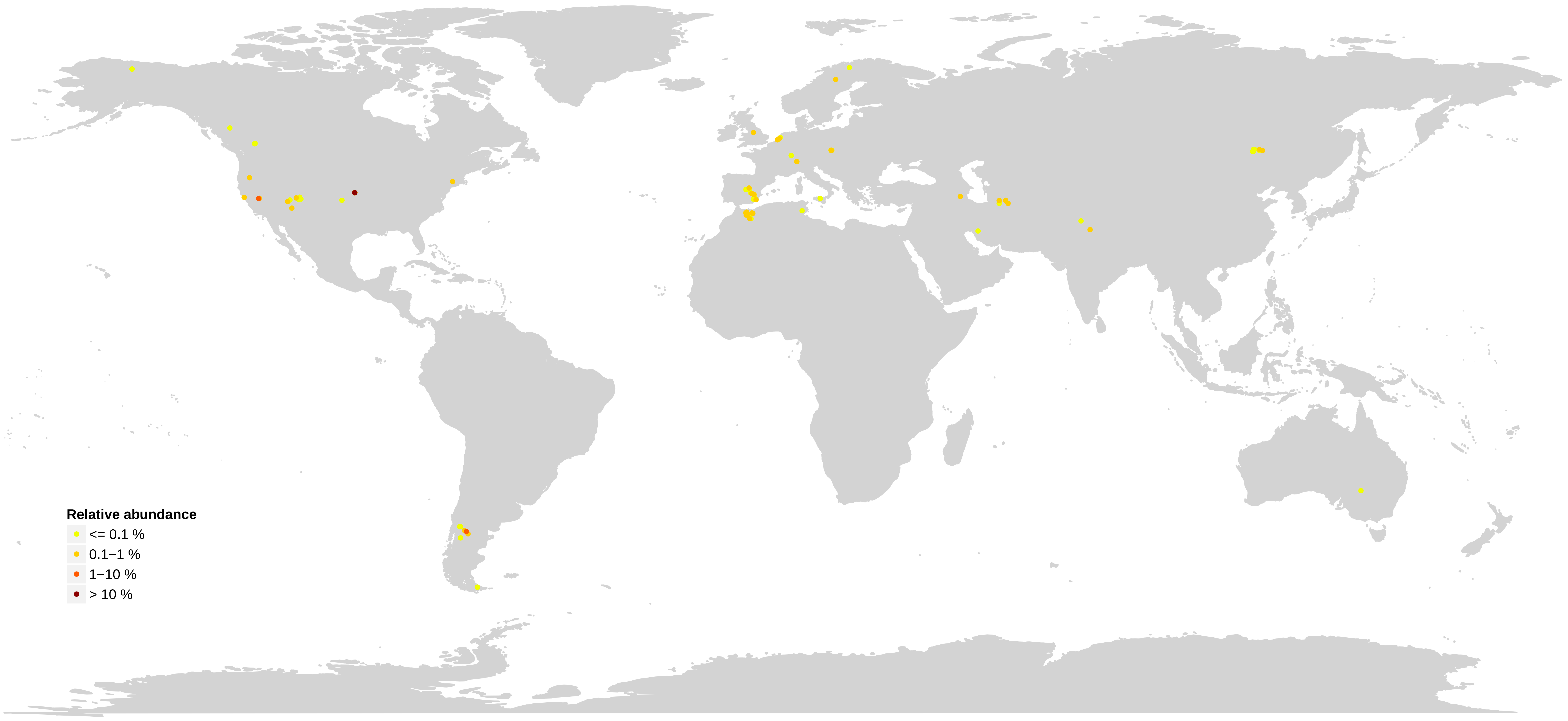

SH220720 Mycena cinerella

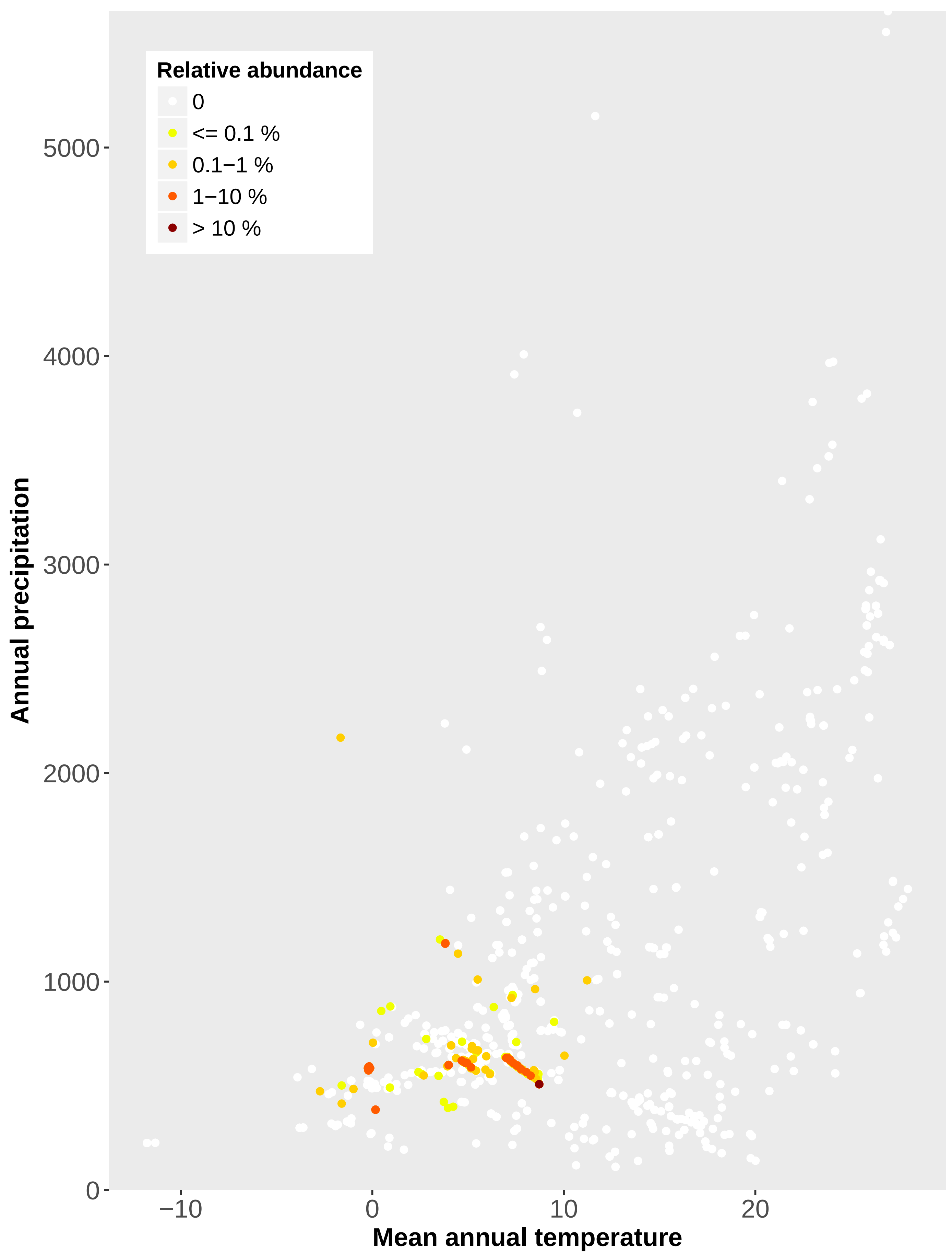

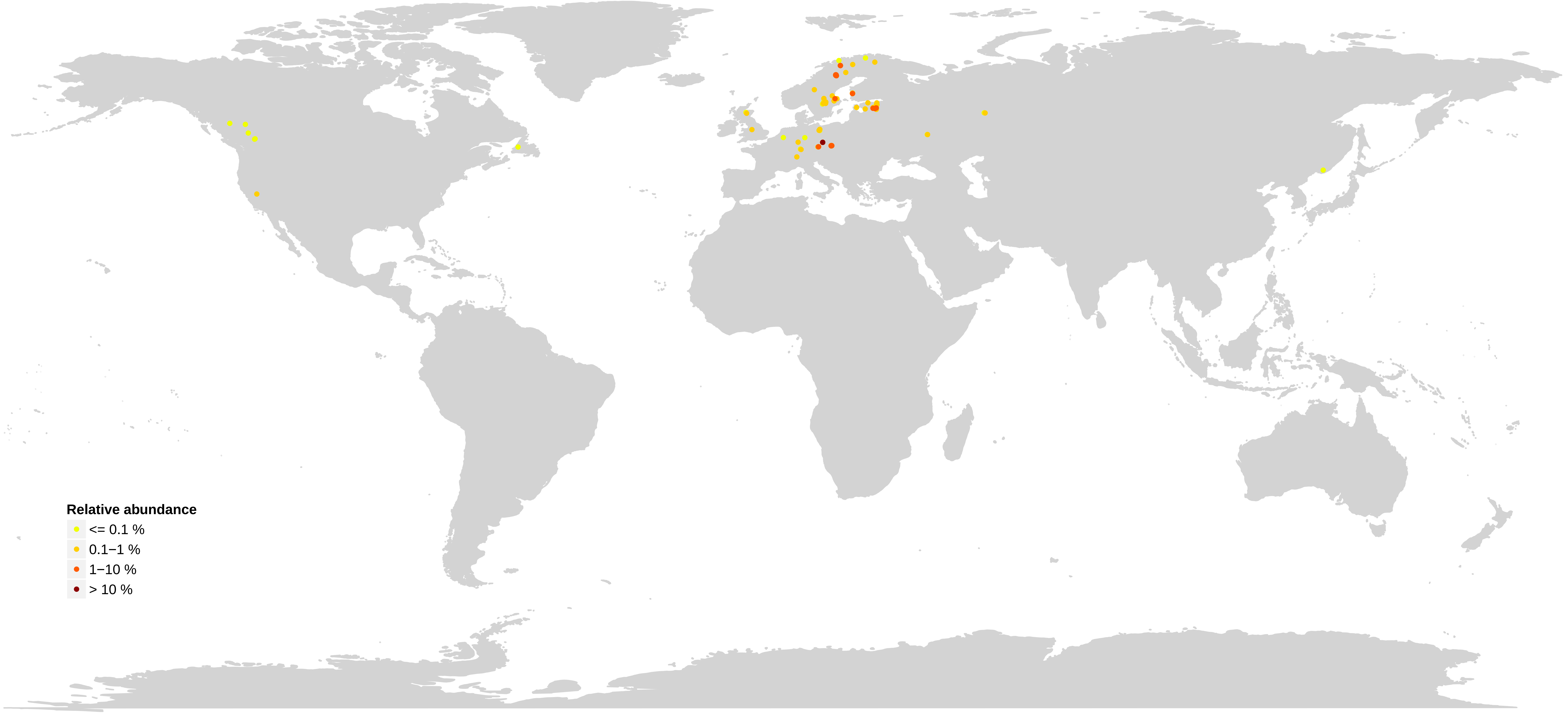

SH176741 Suillus variegatus

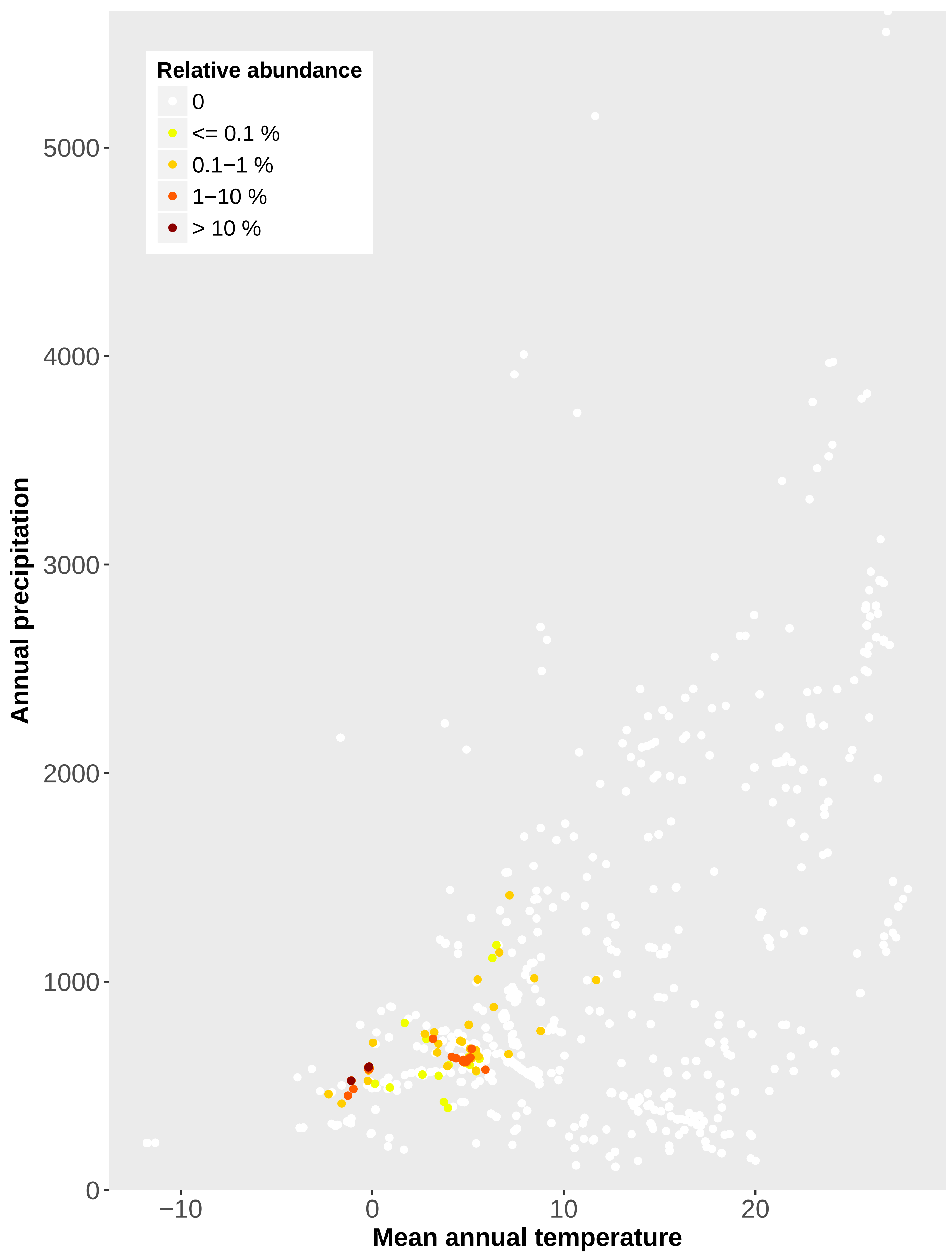

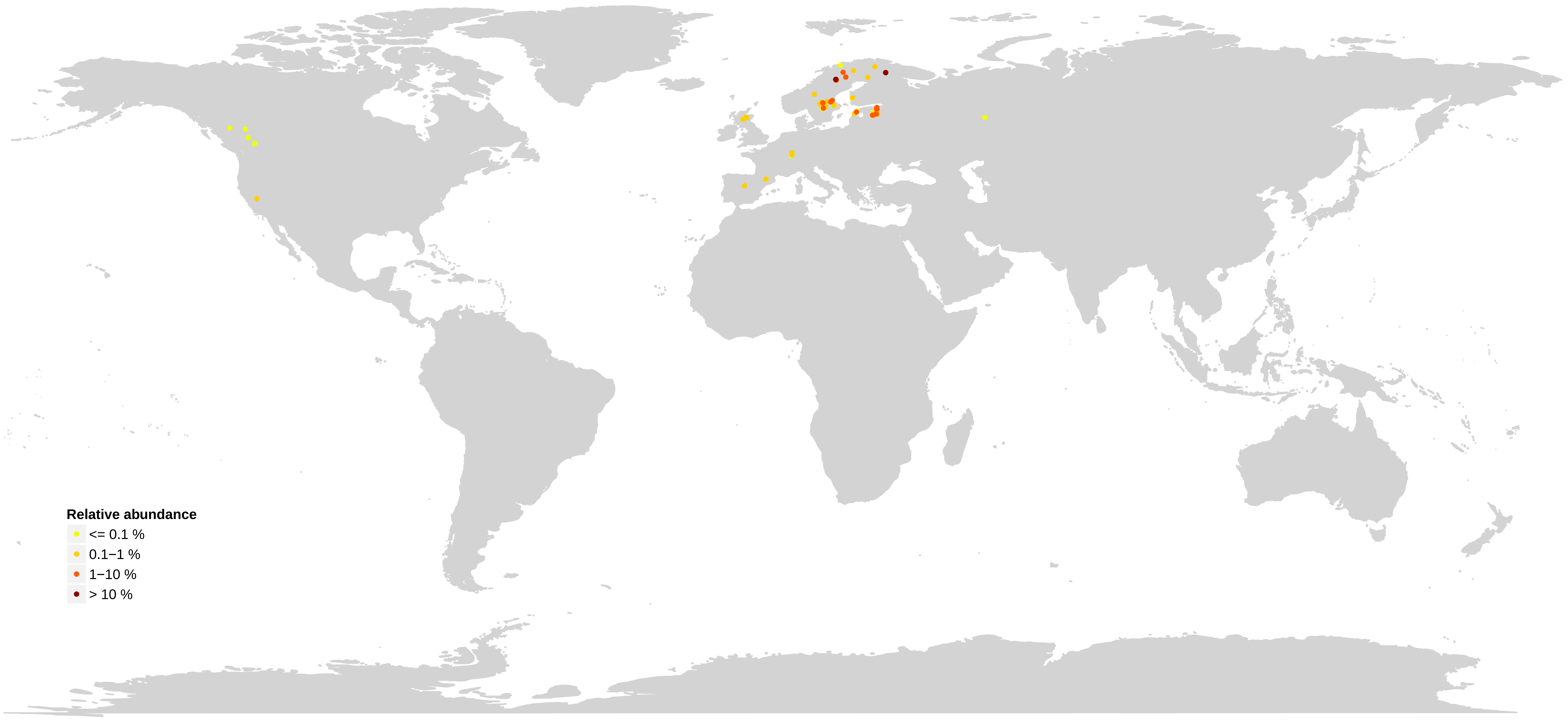

SH196092 Umbelopsis sp

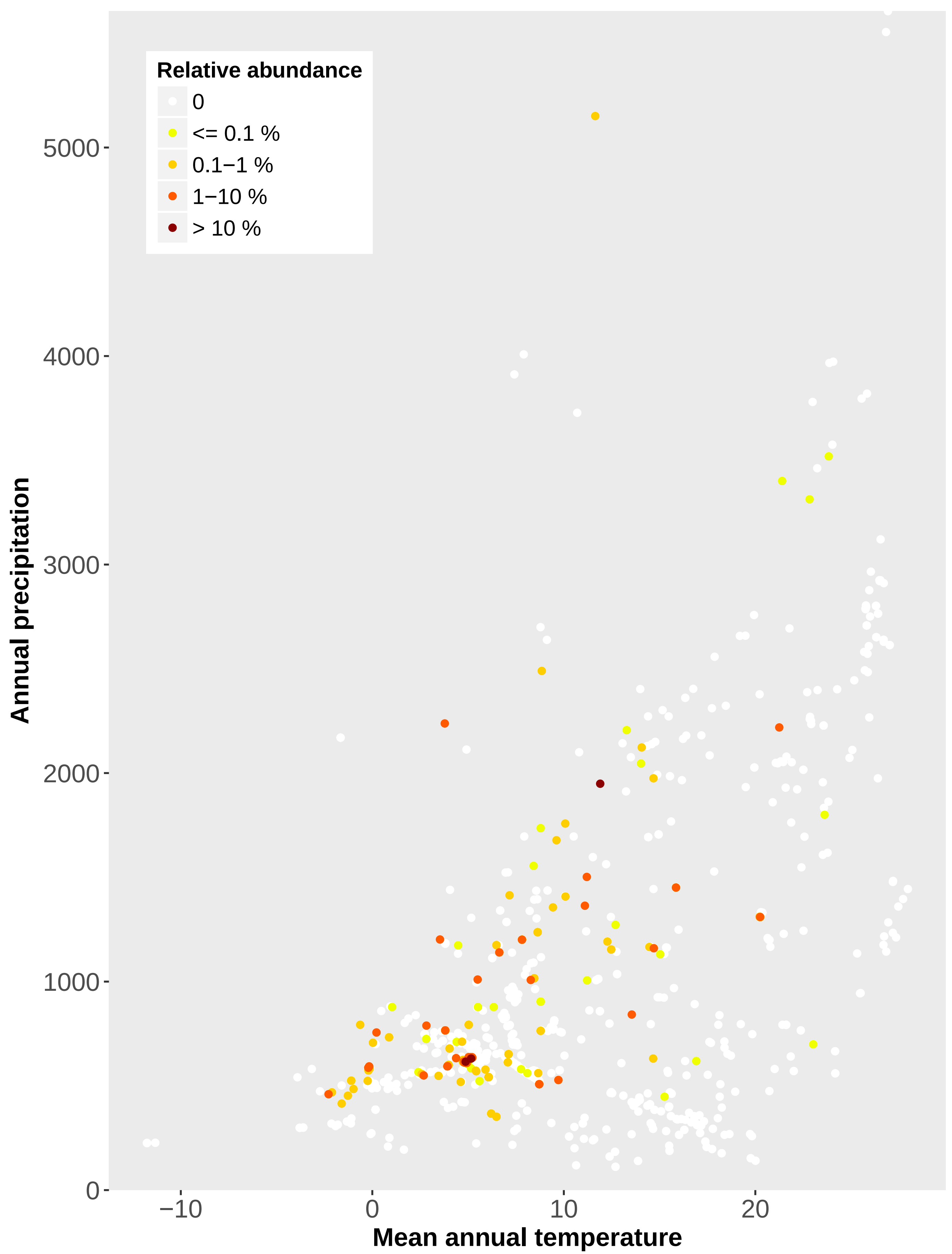

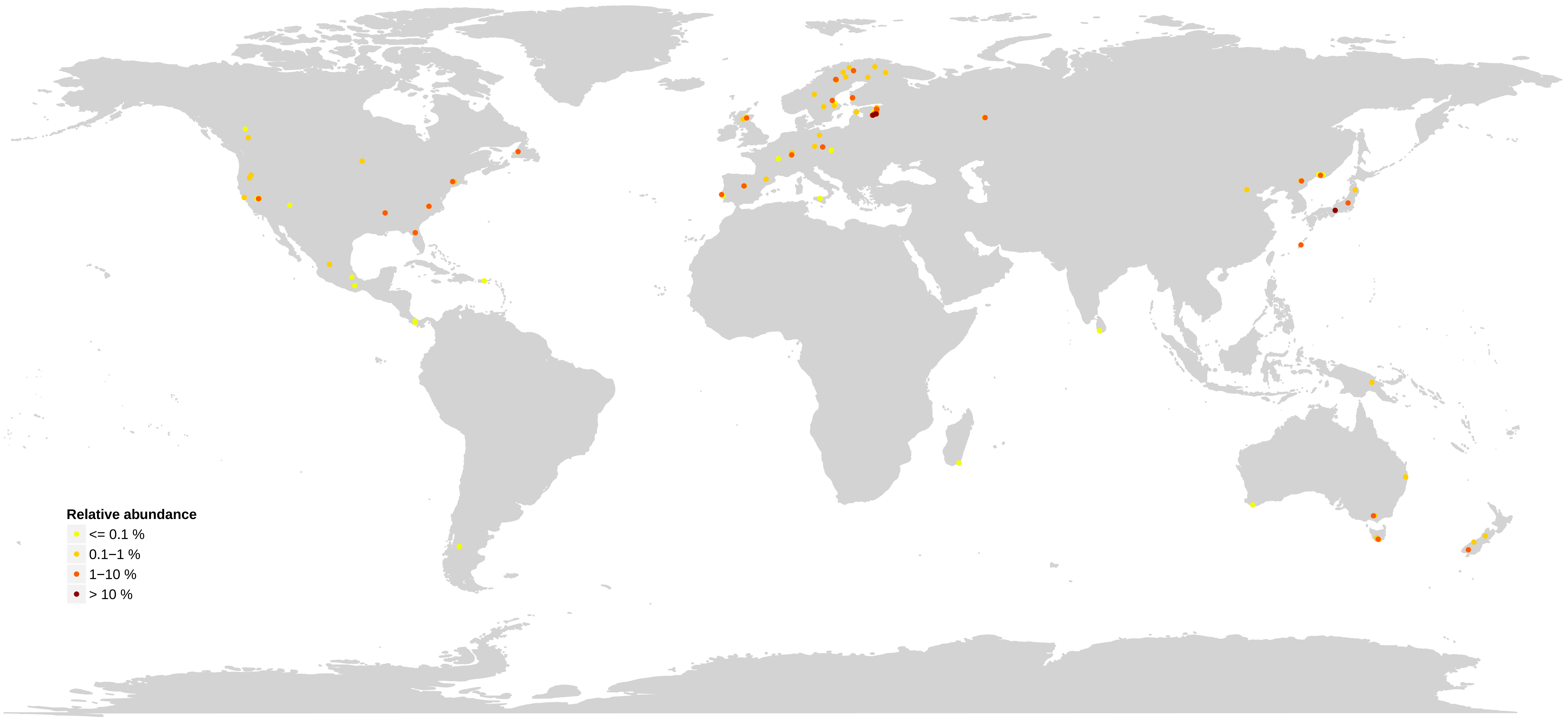

SH208762 Mortierella pseudozygospora

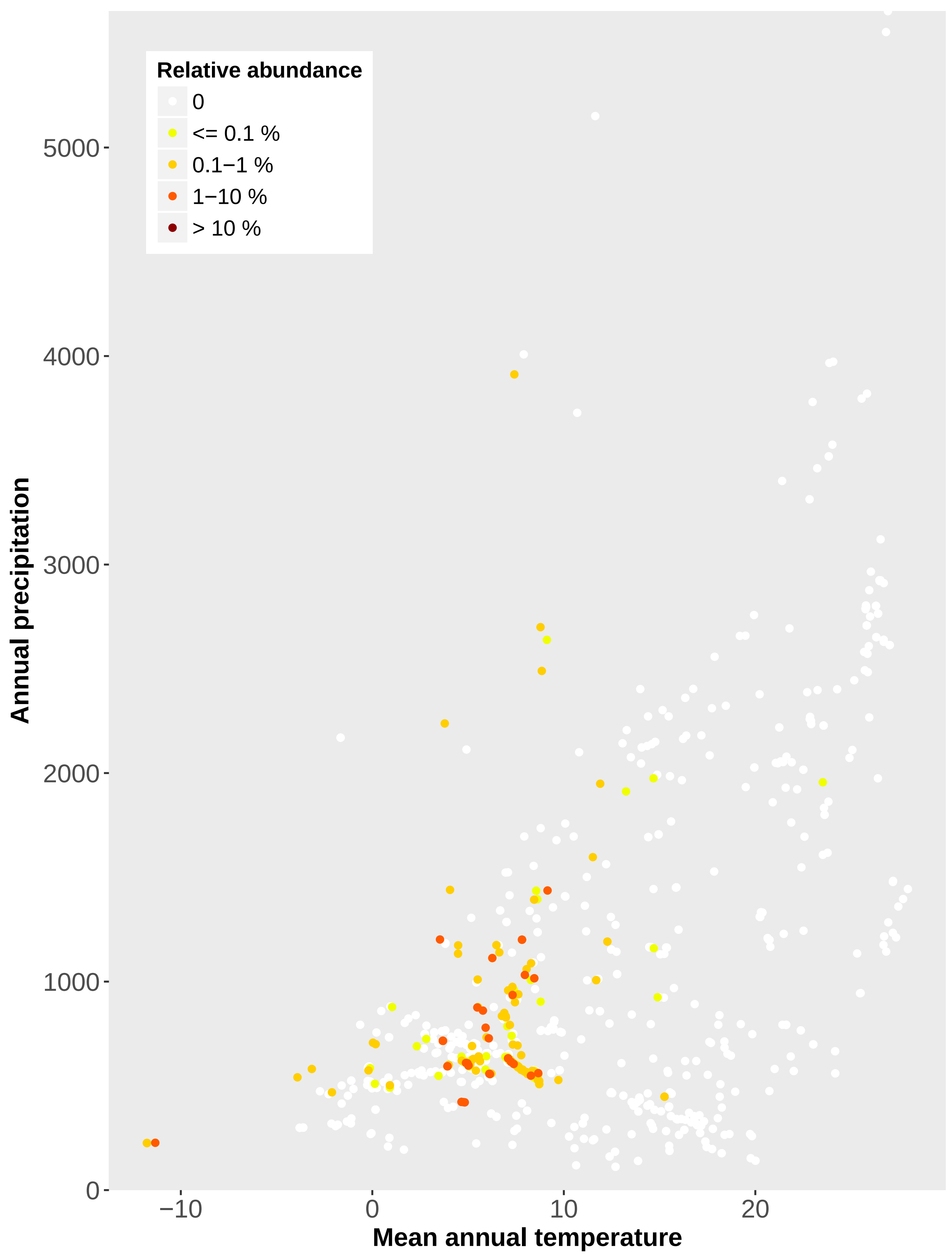

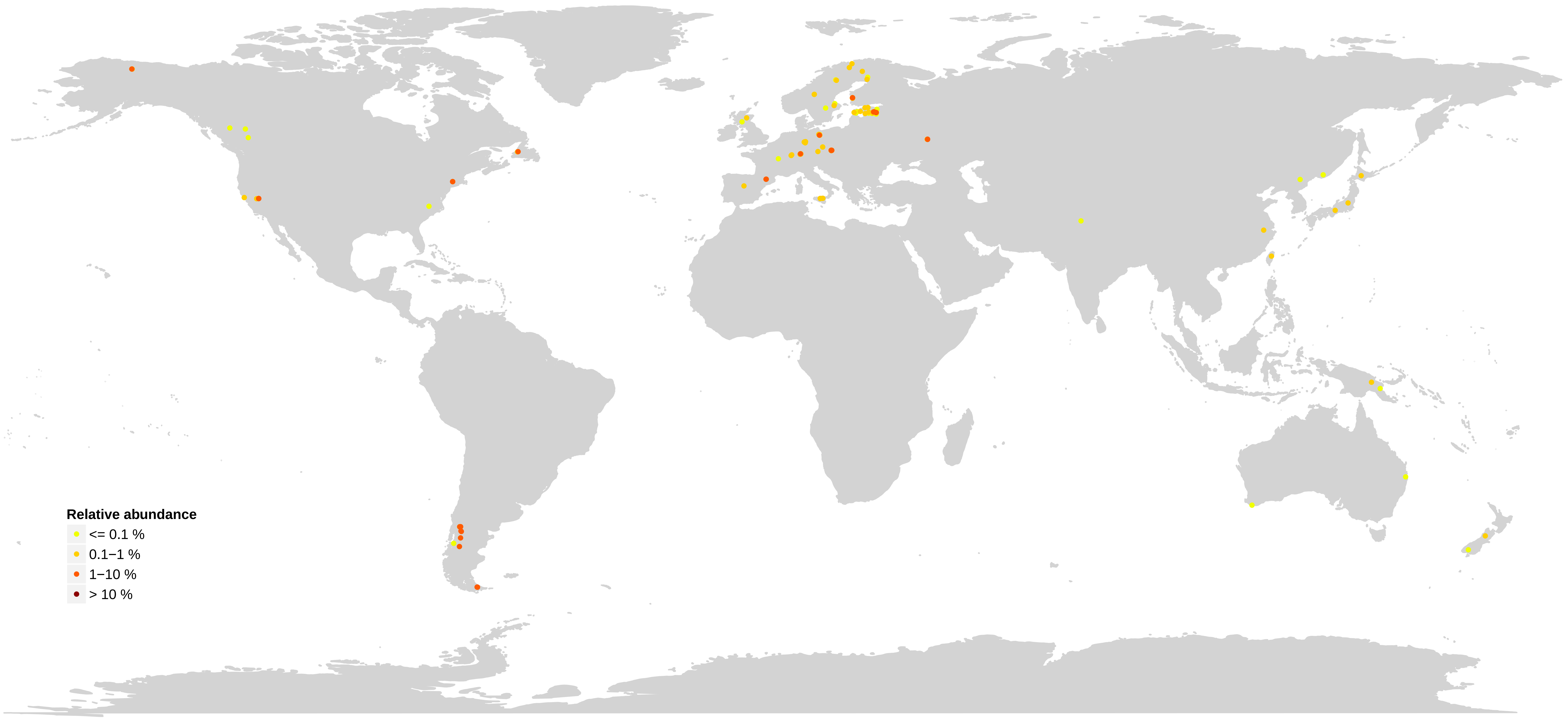

SH180125 Mortierella sp

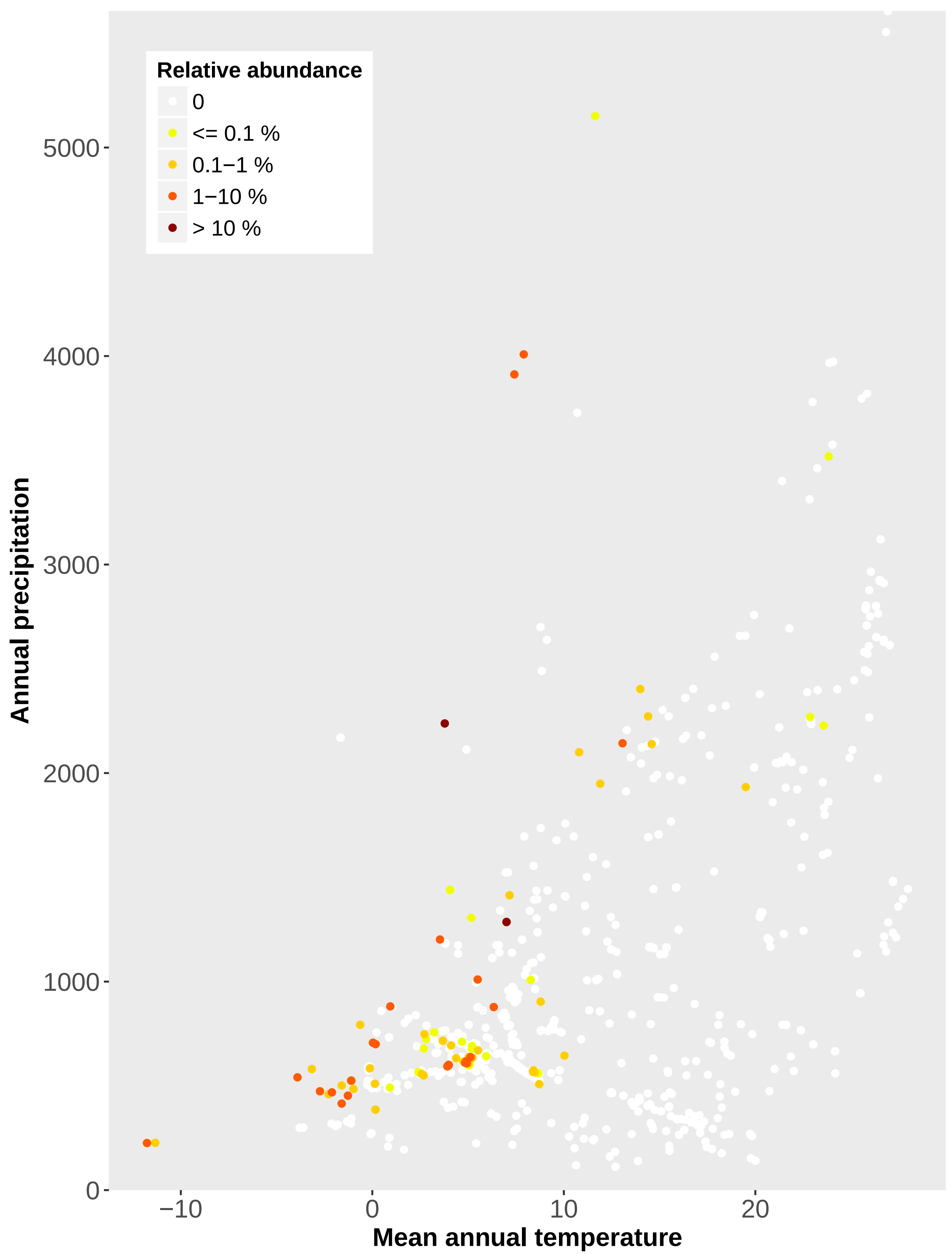

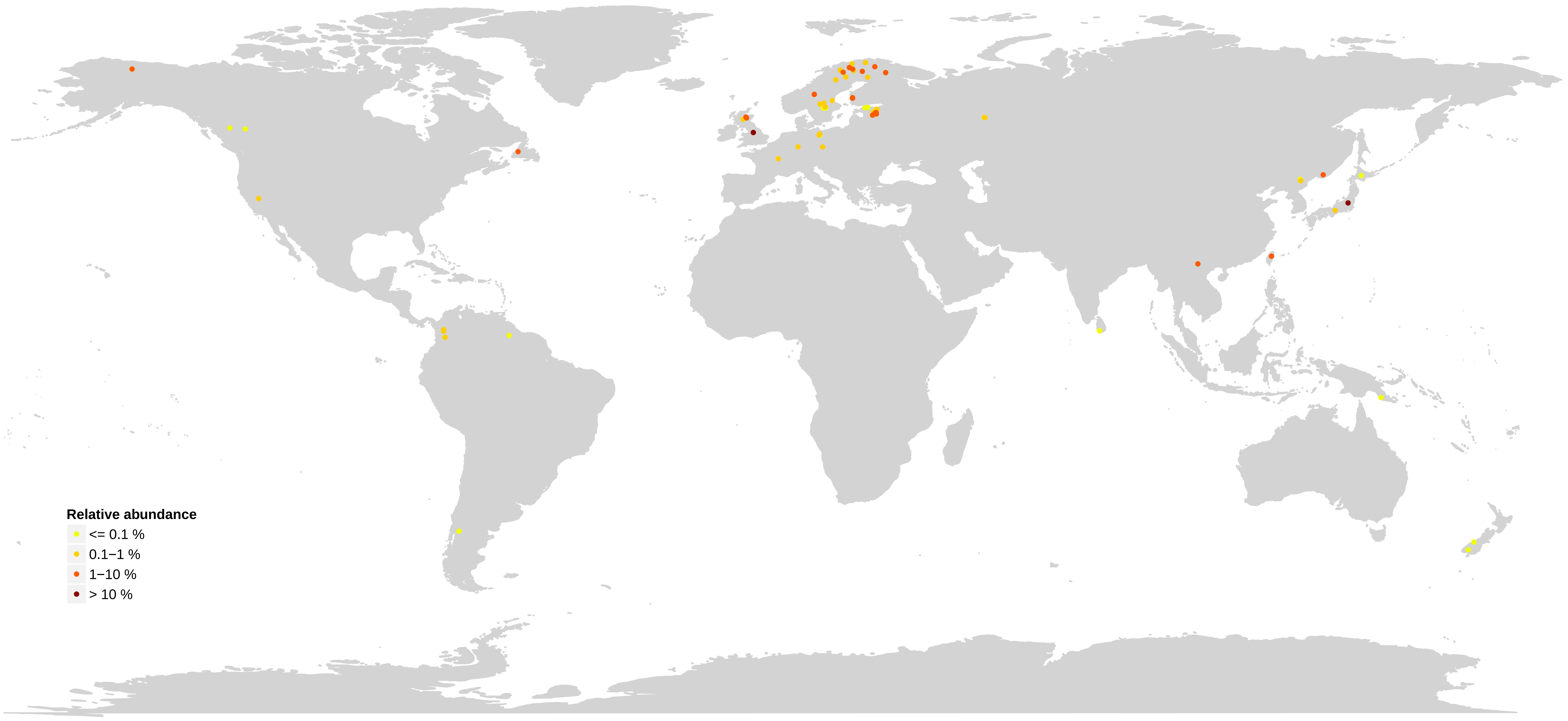

SH008258 Cadophora sp

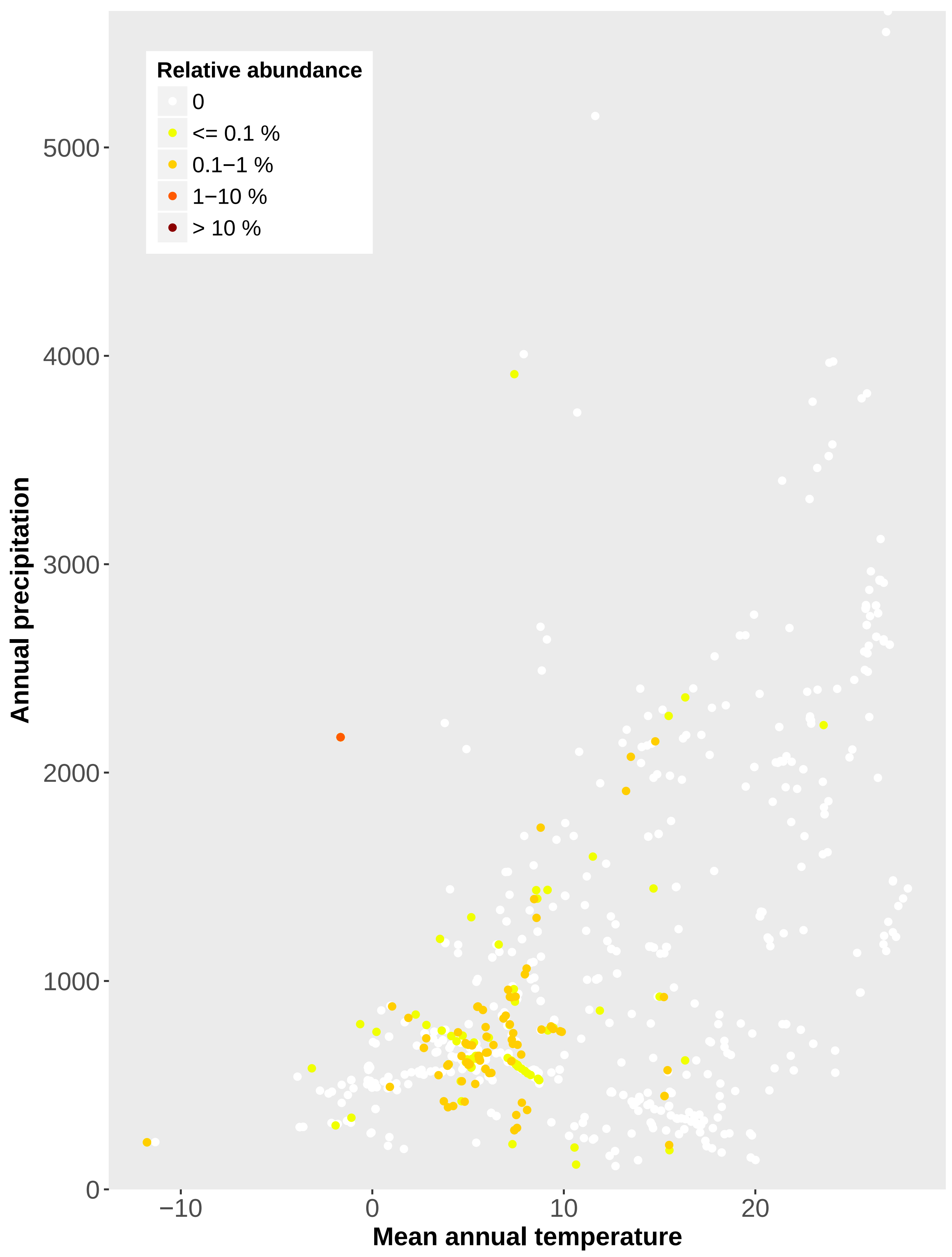

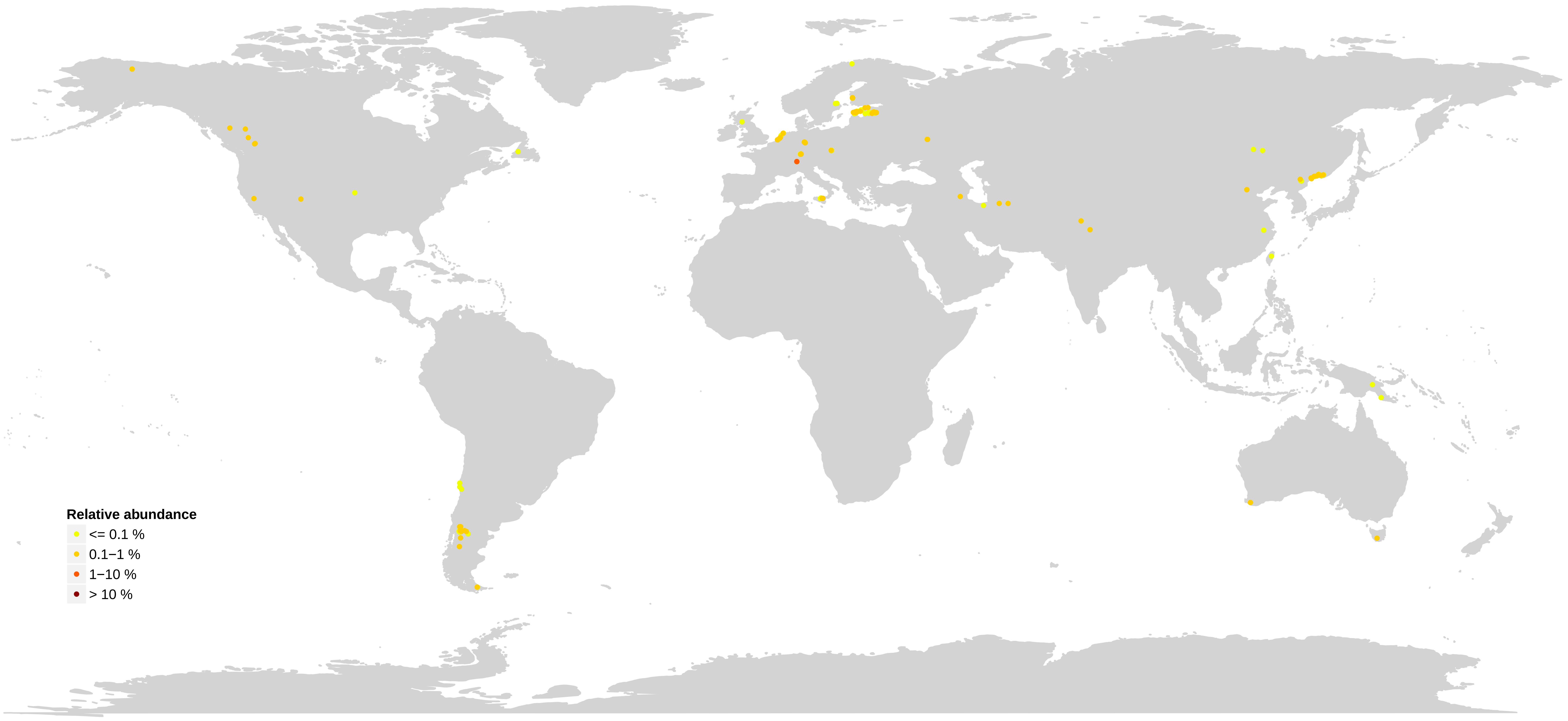

SH117088 Epicoccum nigrum

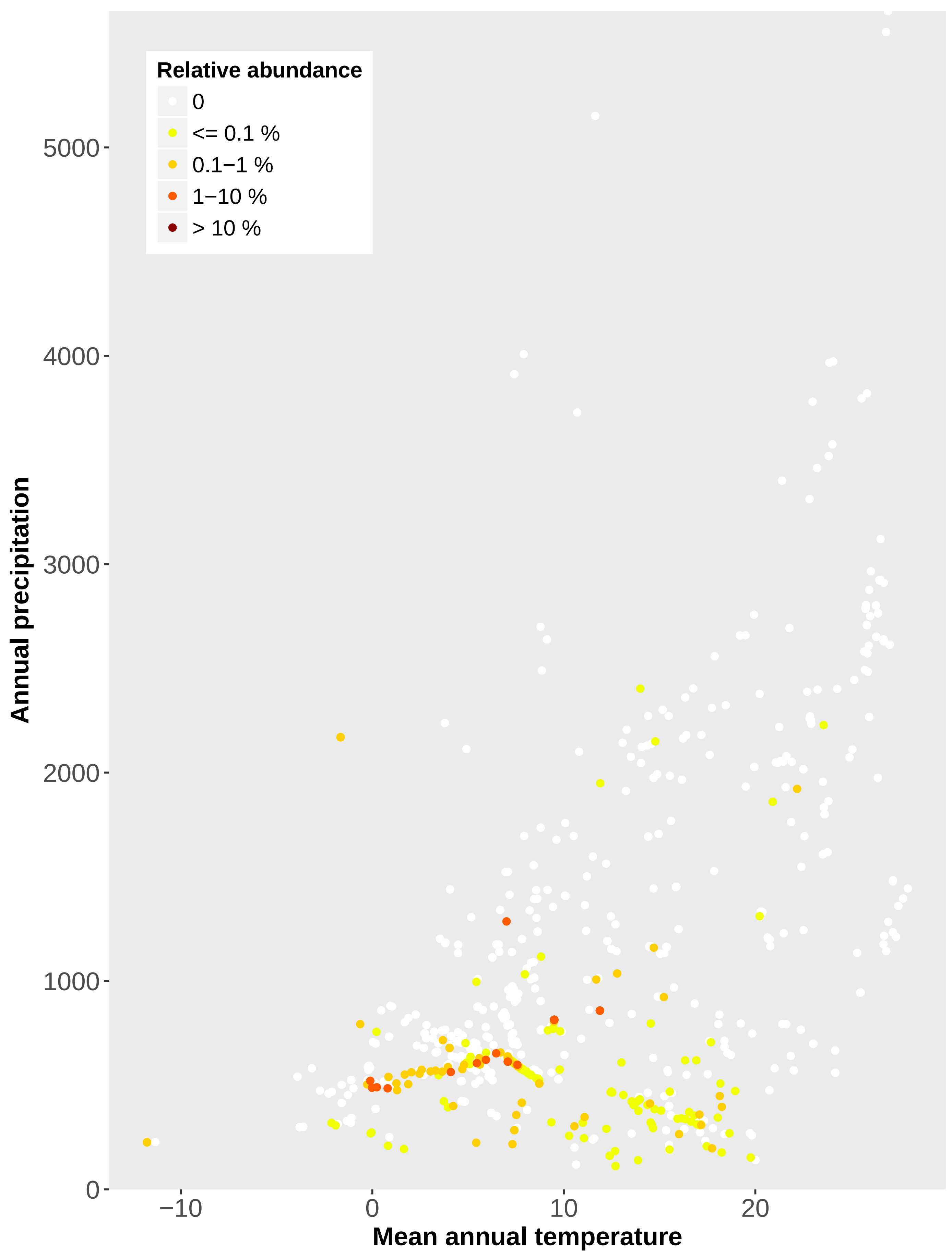

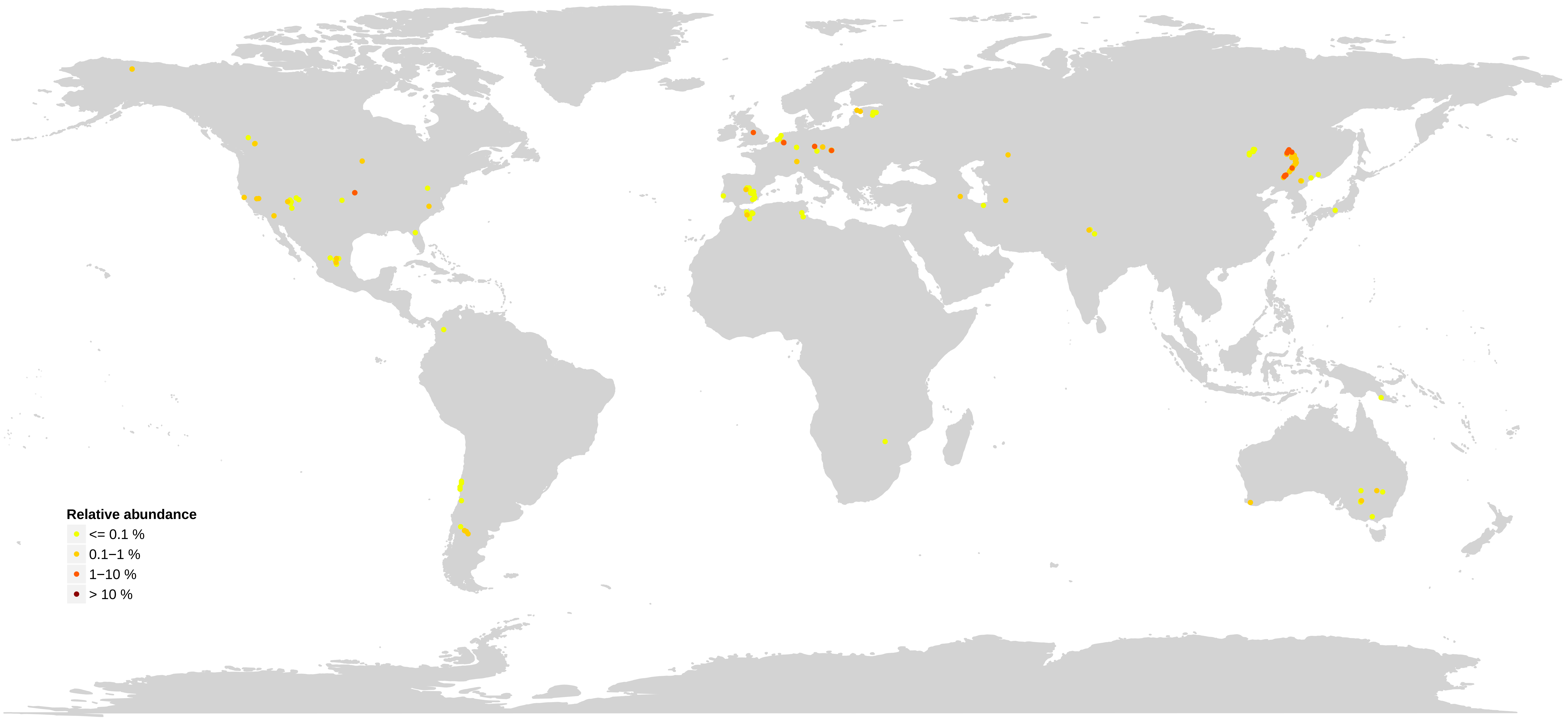

SH190868 Trichoderma spirale

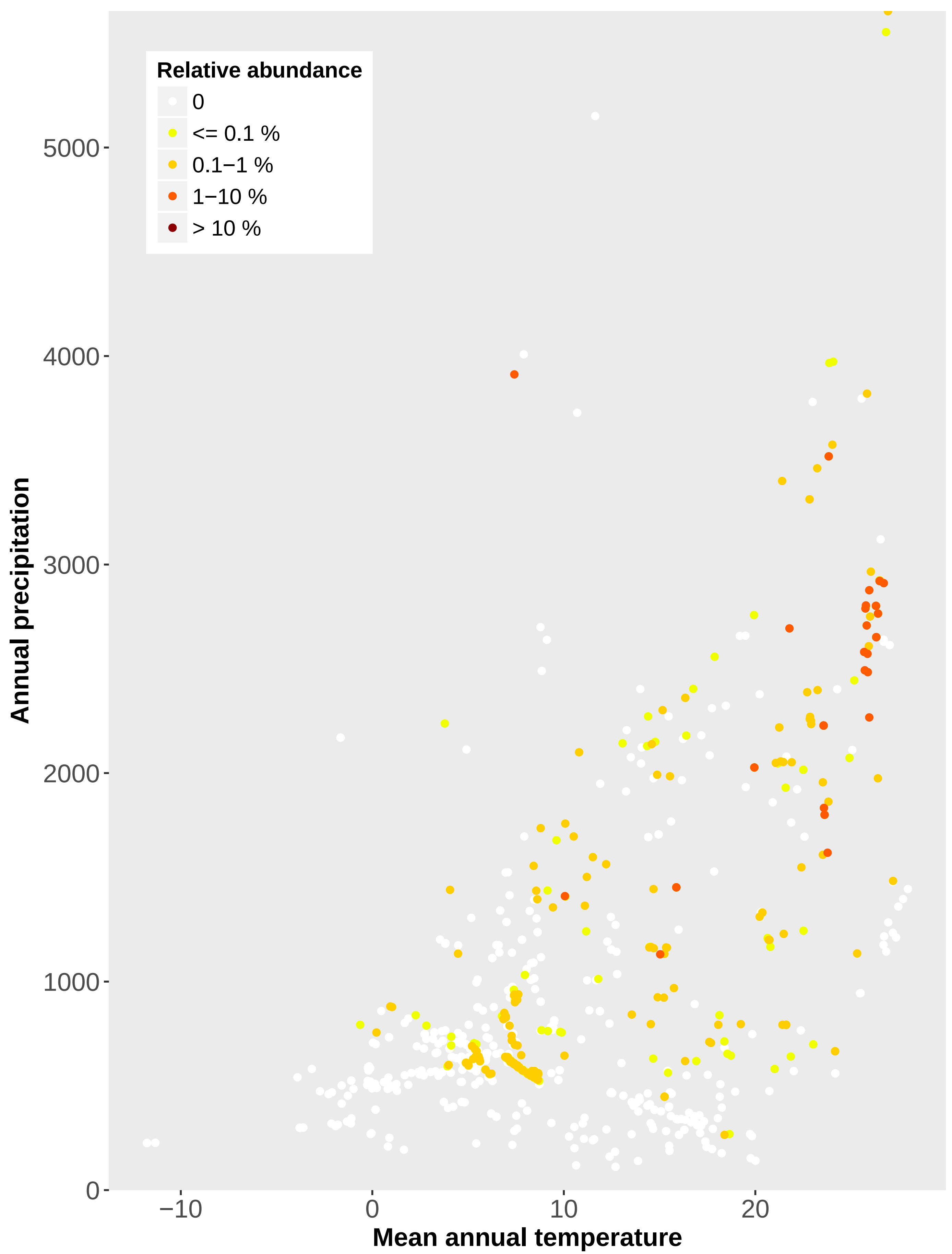

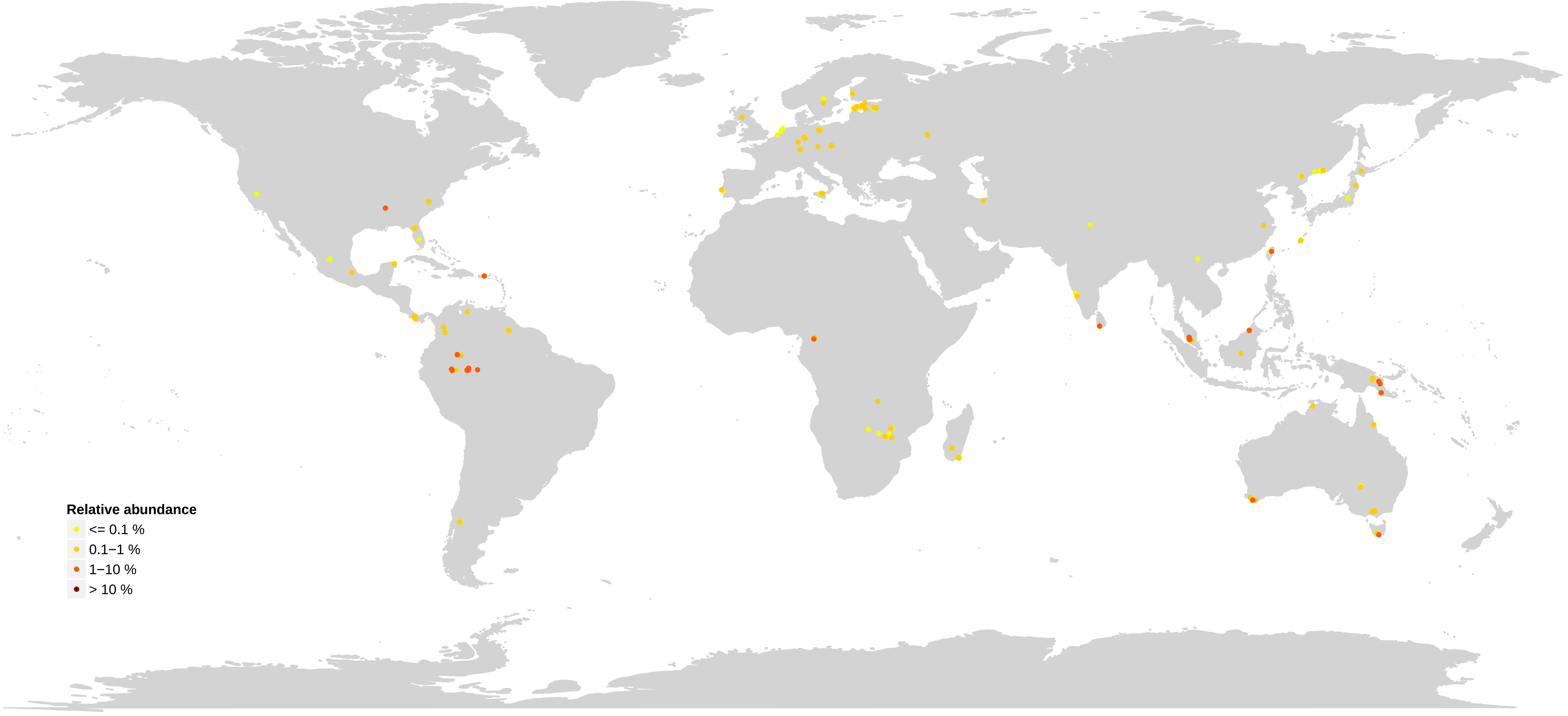

SH197469 Sebacinales sp

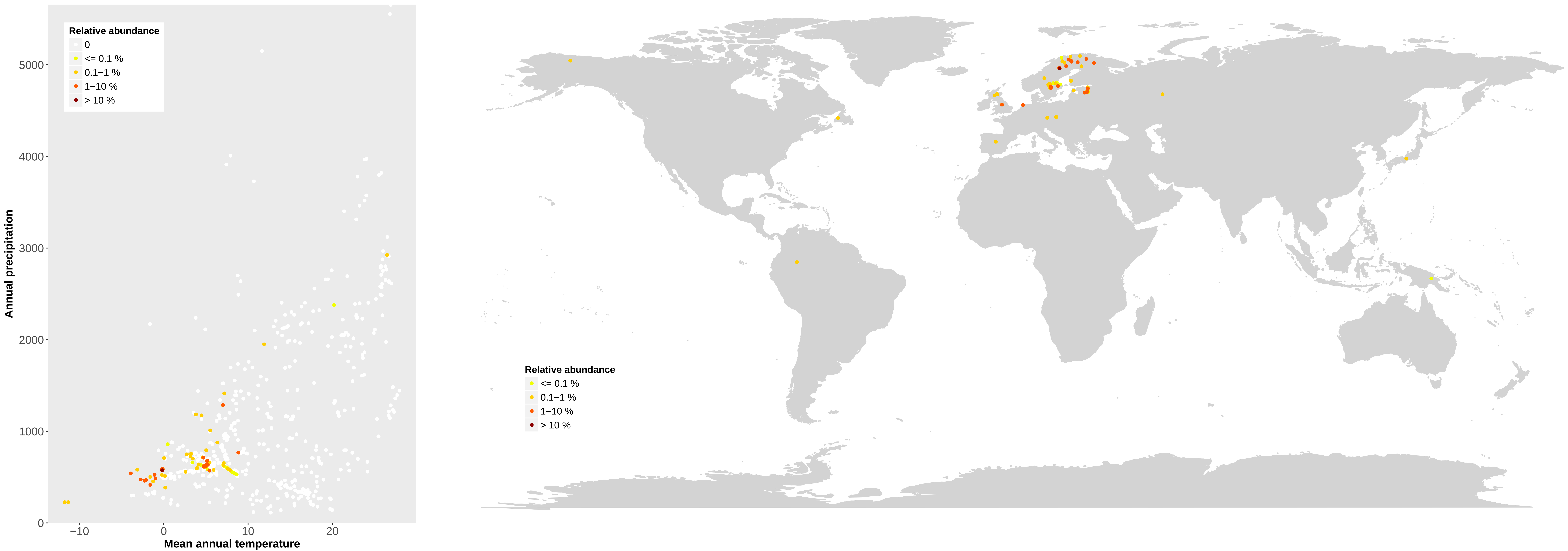

SH214267 Ascomycota sp

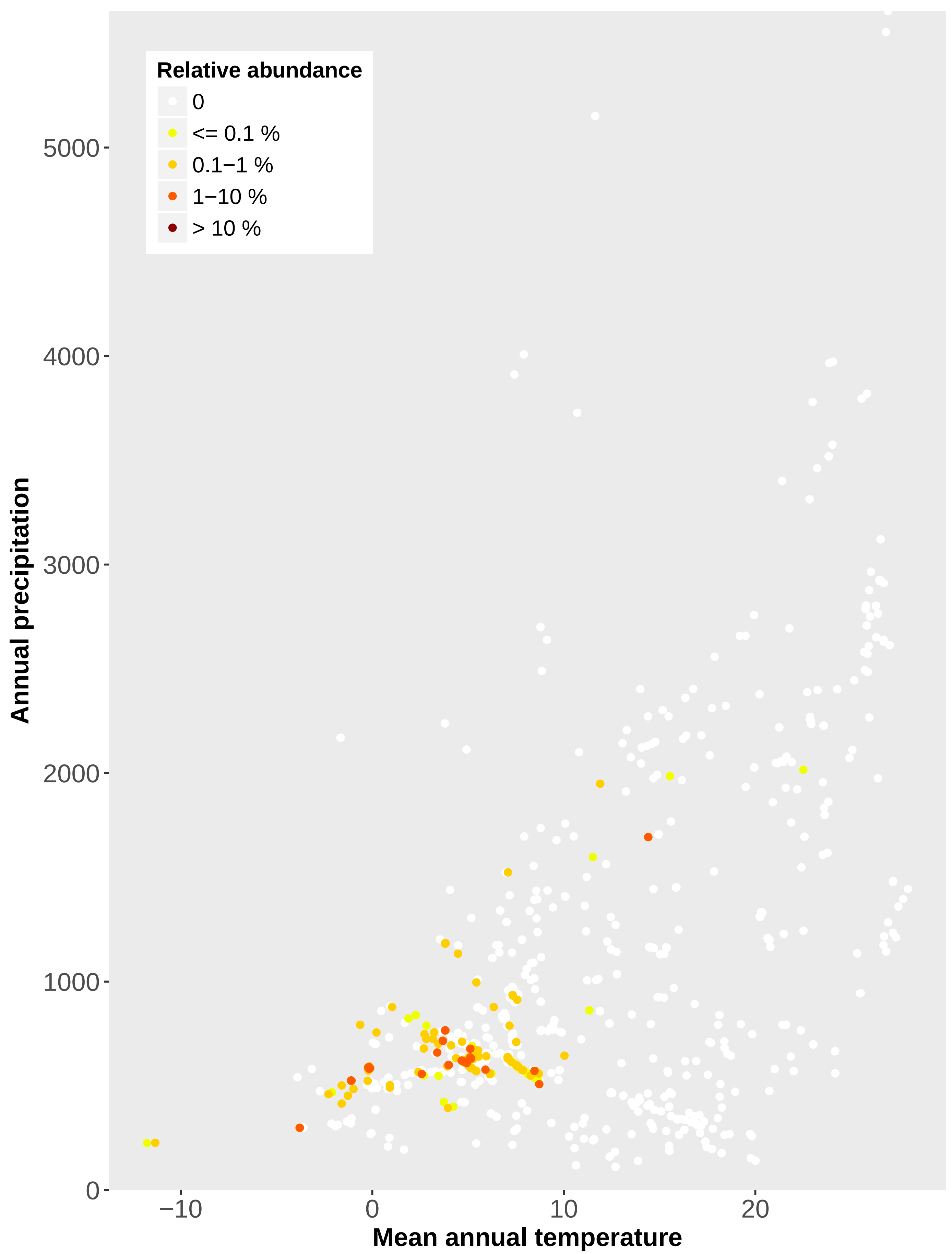

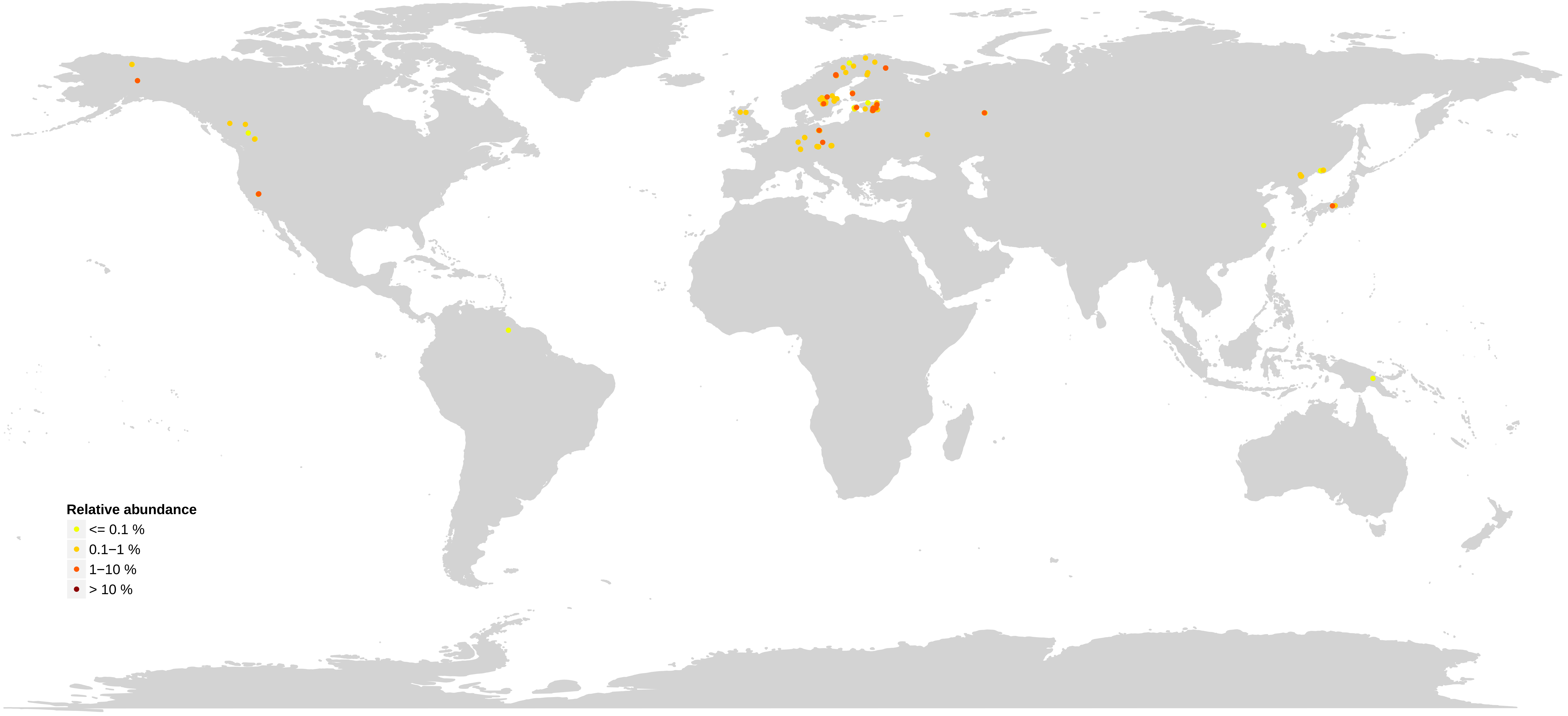

SH201320 Dothideomycetes sp

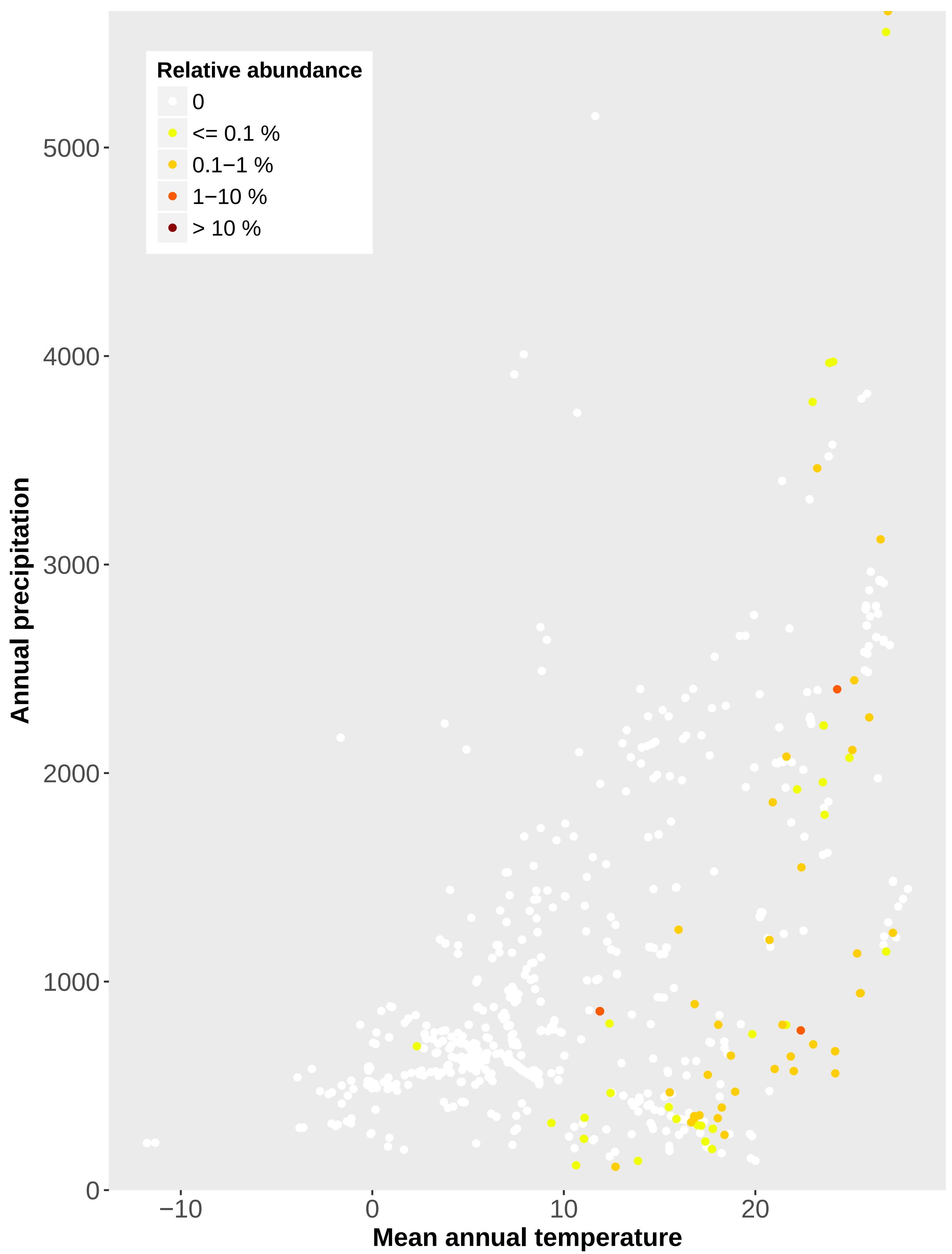

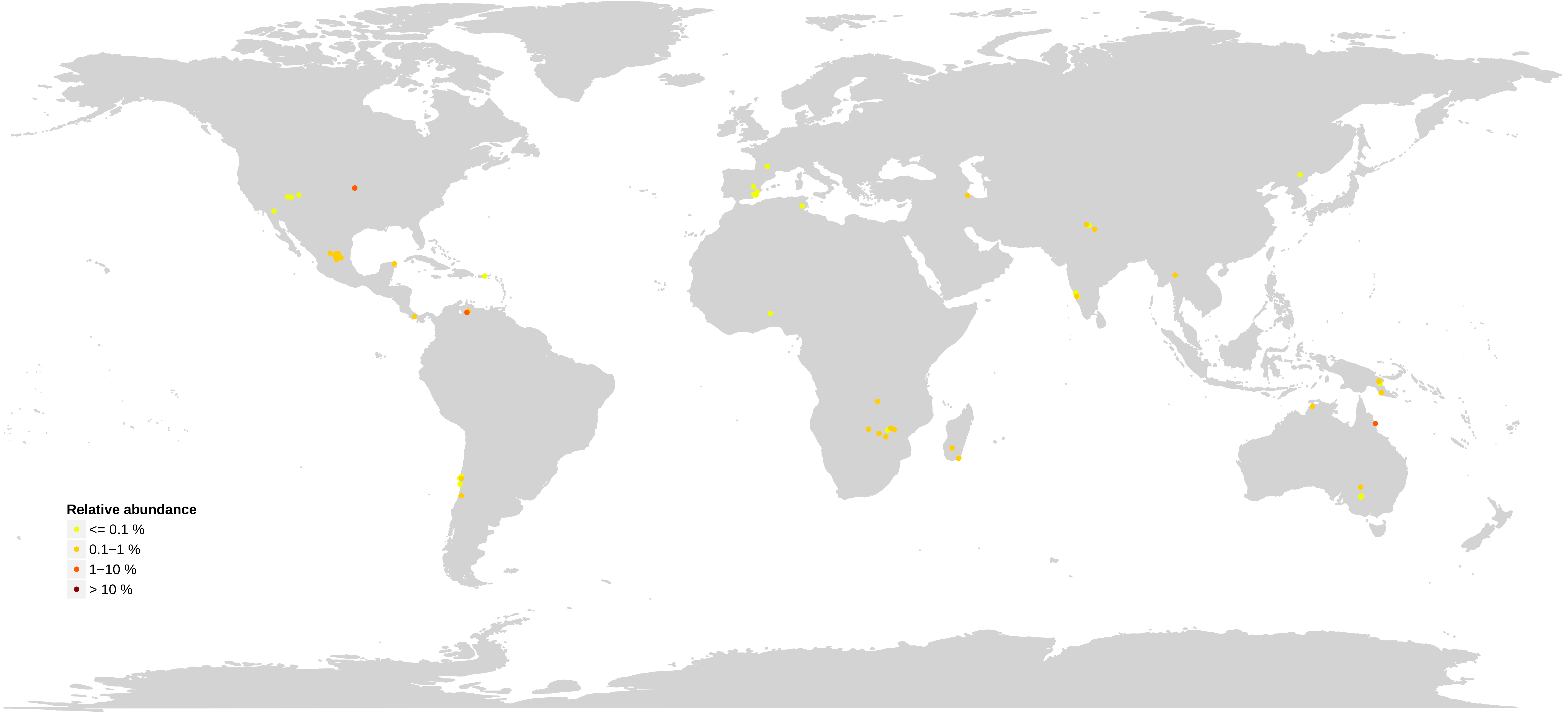

SH350573 Trichoderma koningiopsis

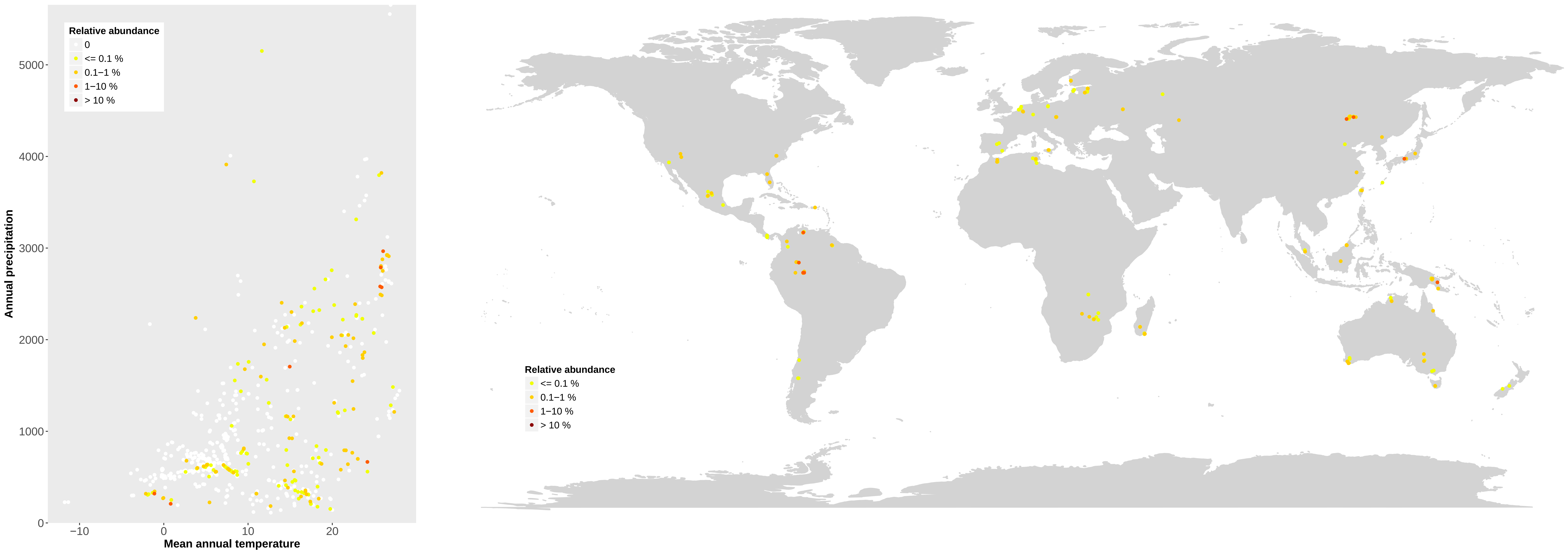

SH193237 Chaetothyriales sp

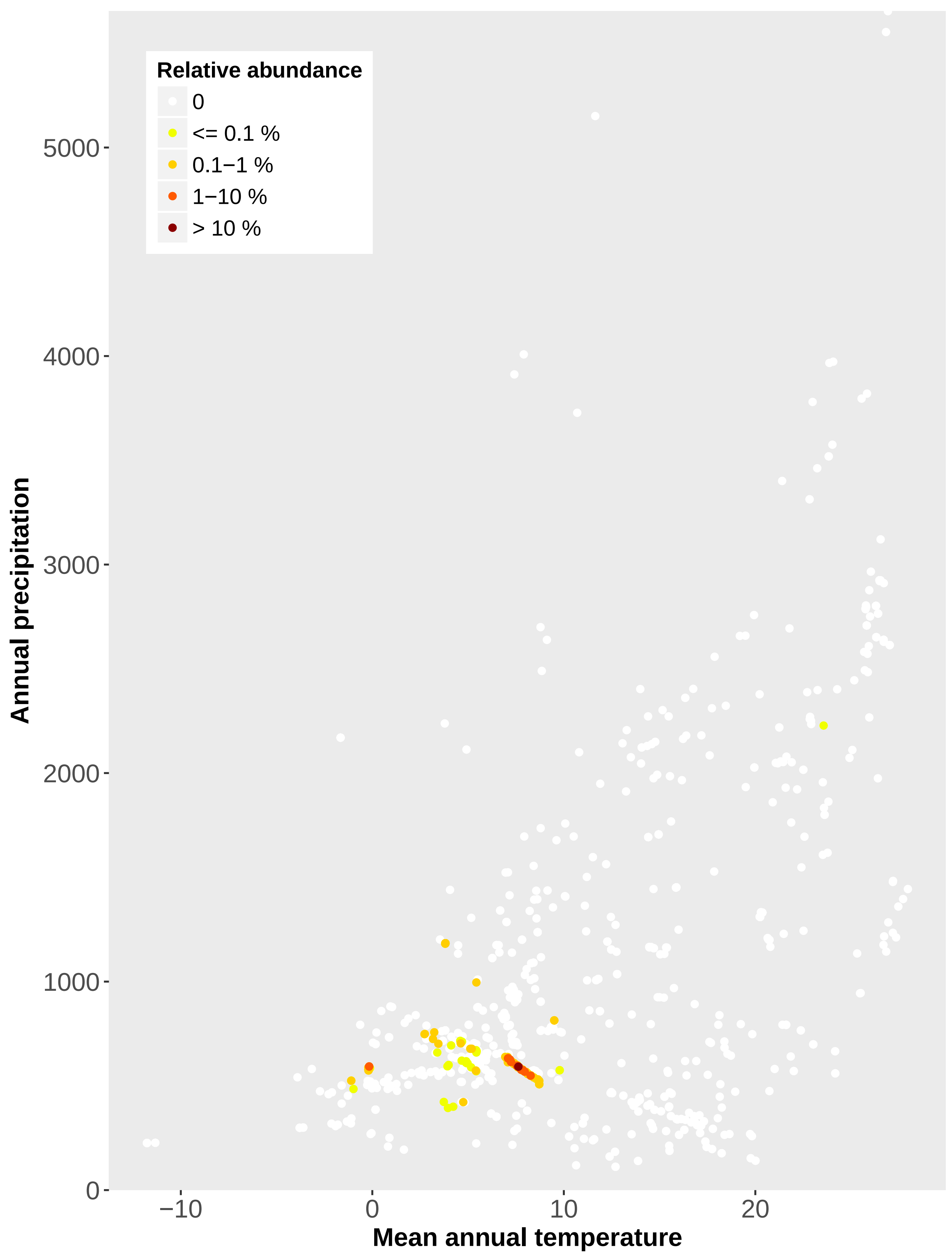

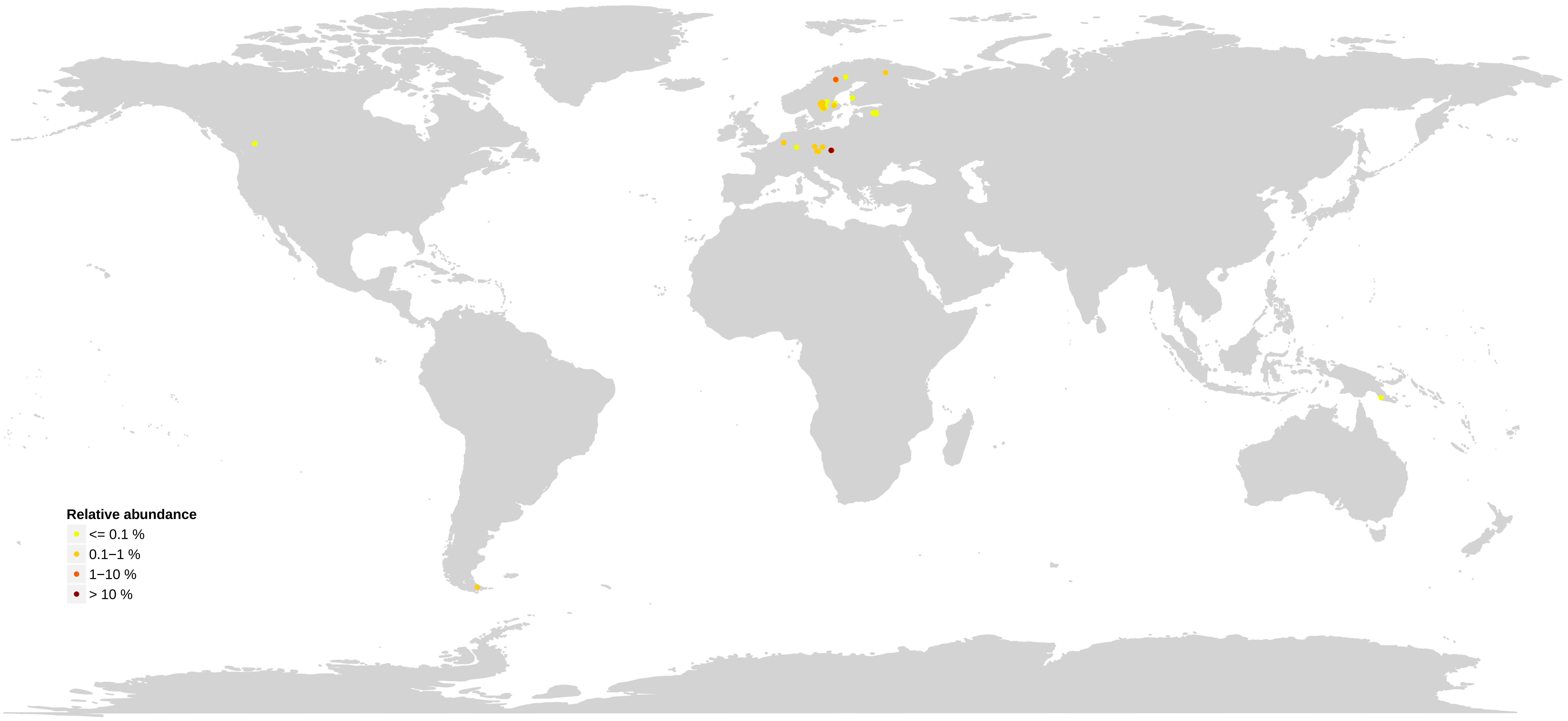

SH180109 Mortierella rishikesha

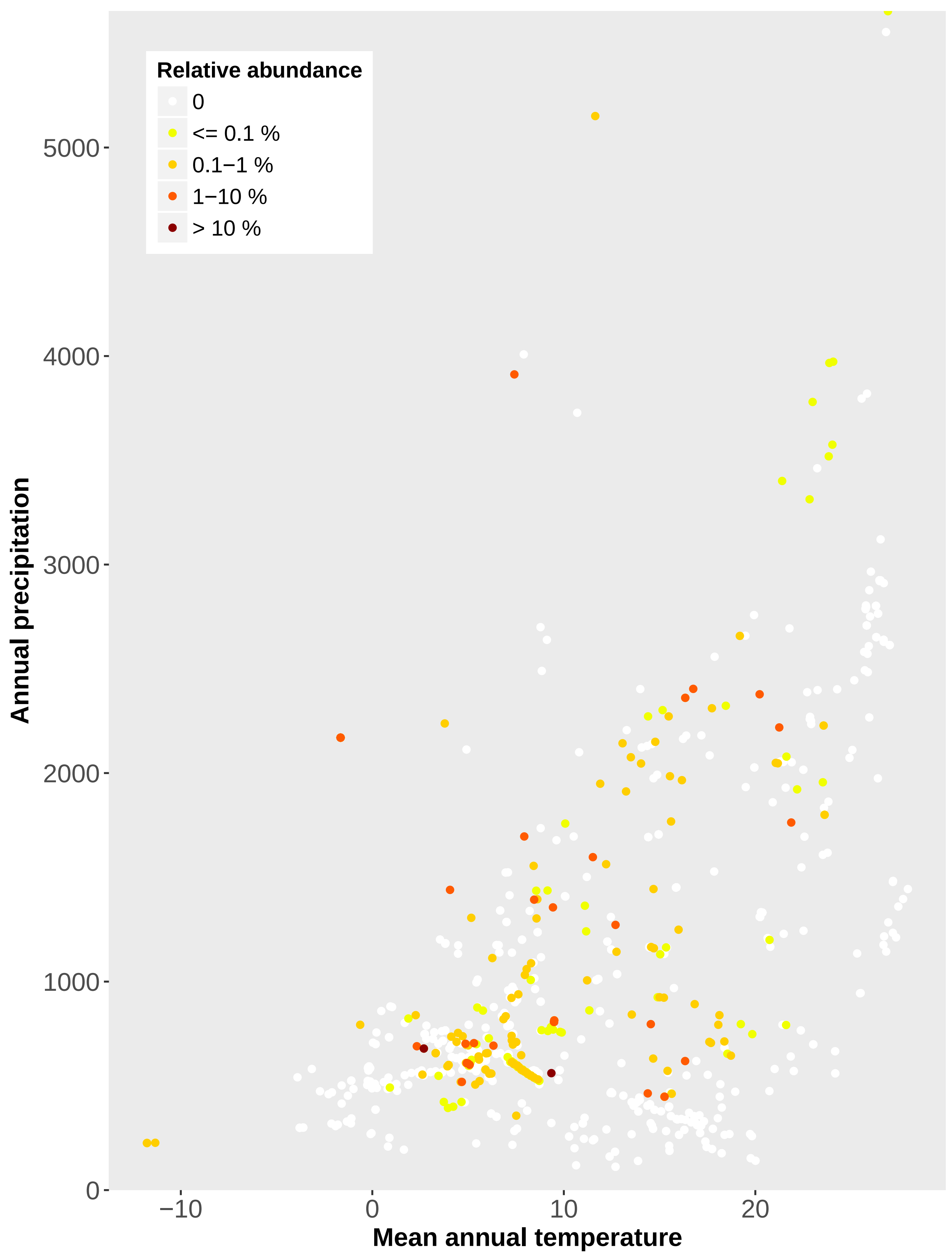

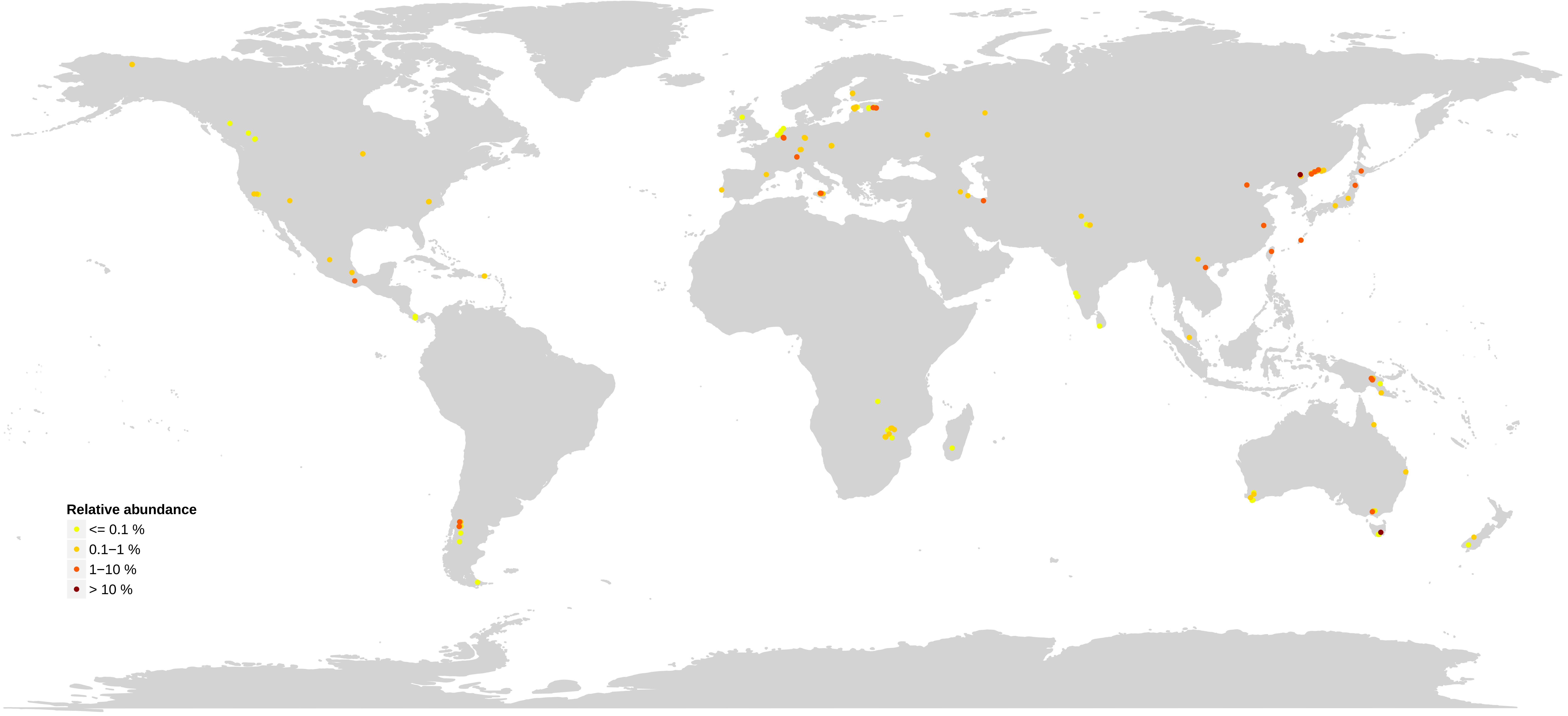

SH182624 Lophodermium piceae

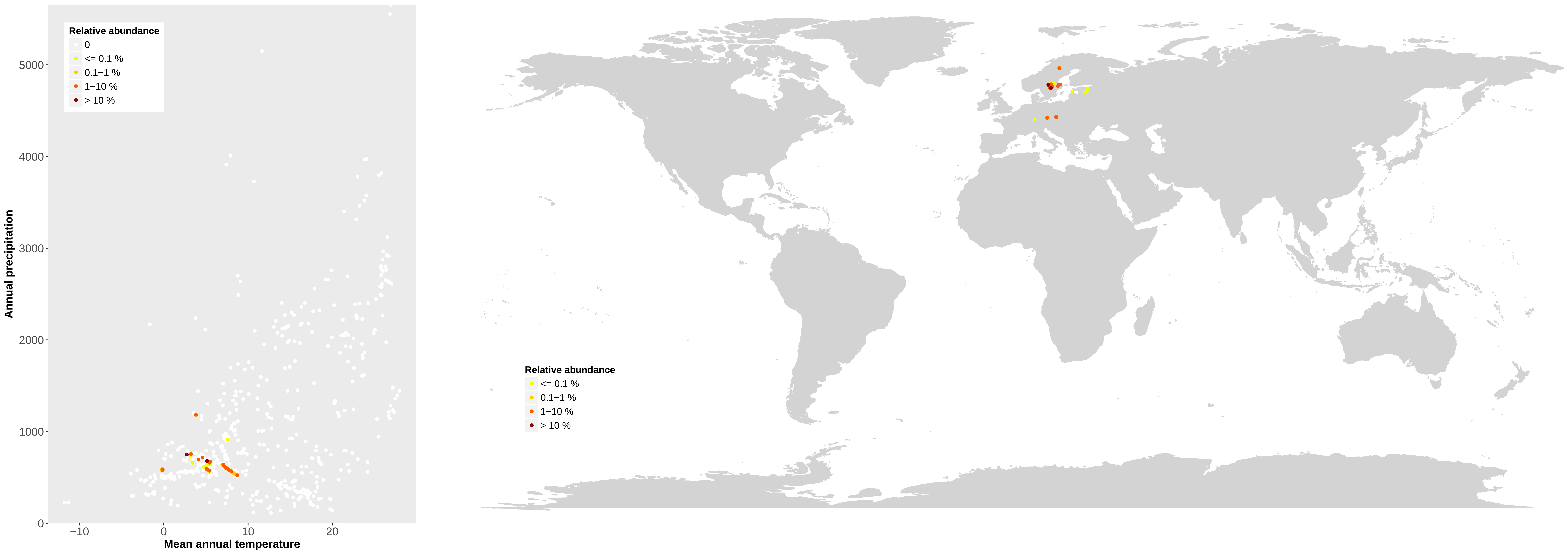

SH200238 Ascomycota sp

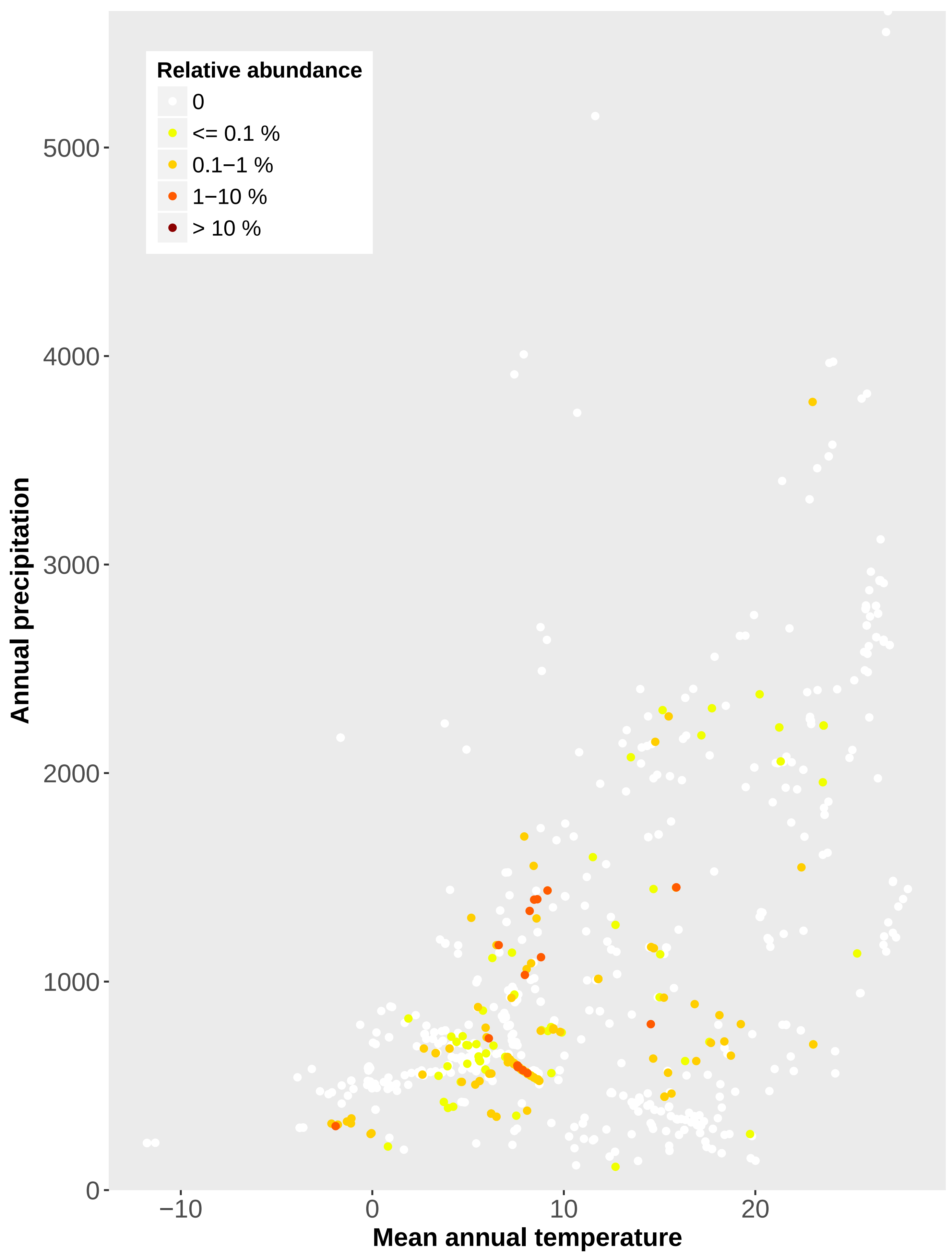

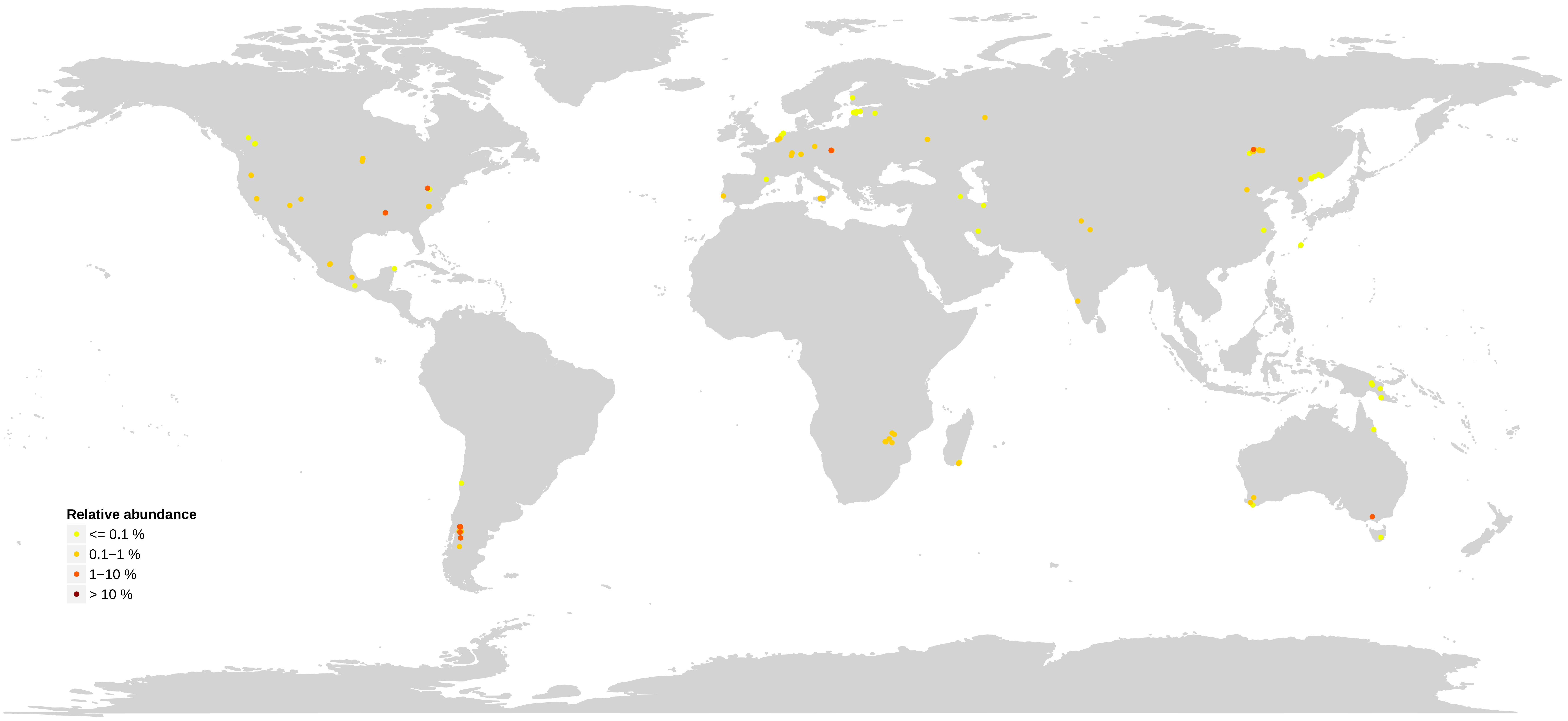

SH198655 Ascomycota sp

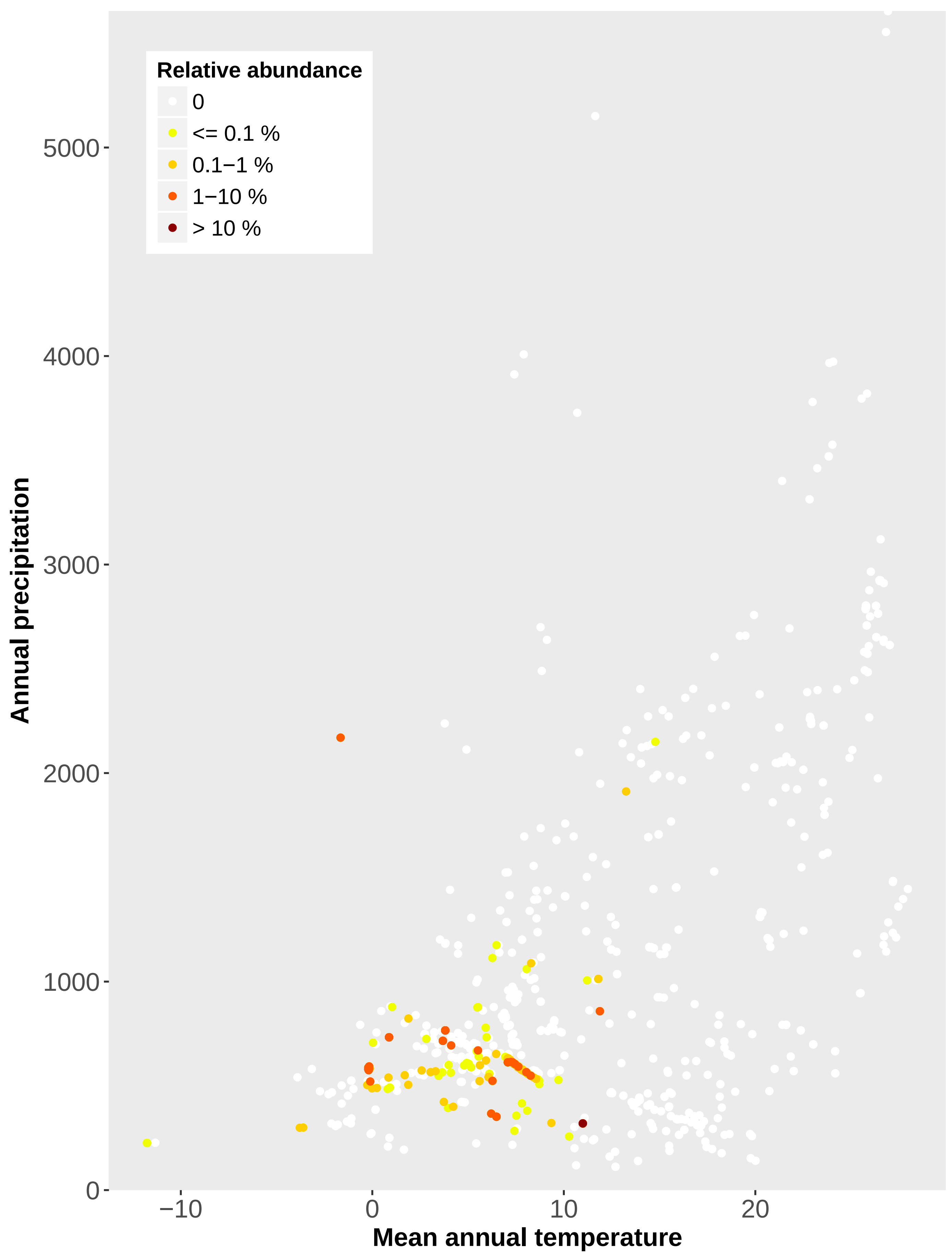

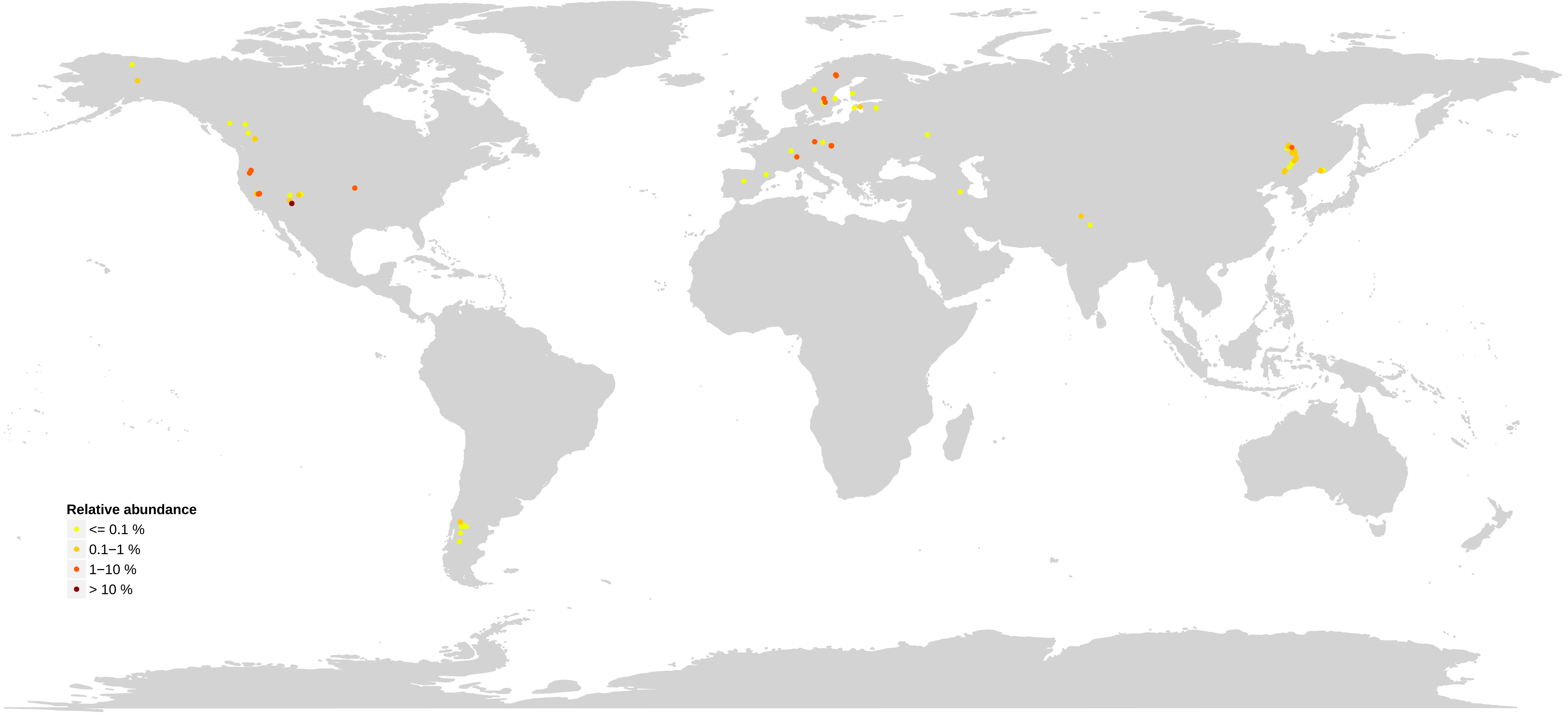

SH214904 Ascomycota sp

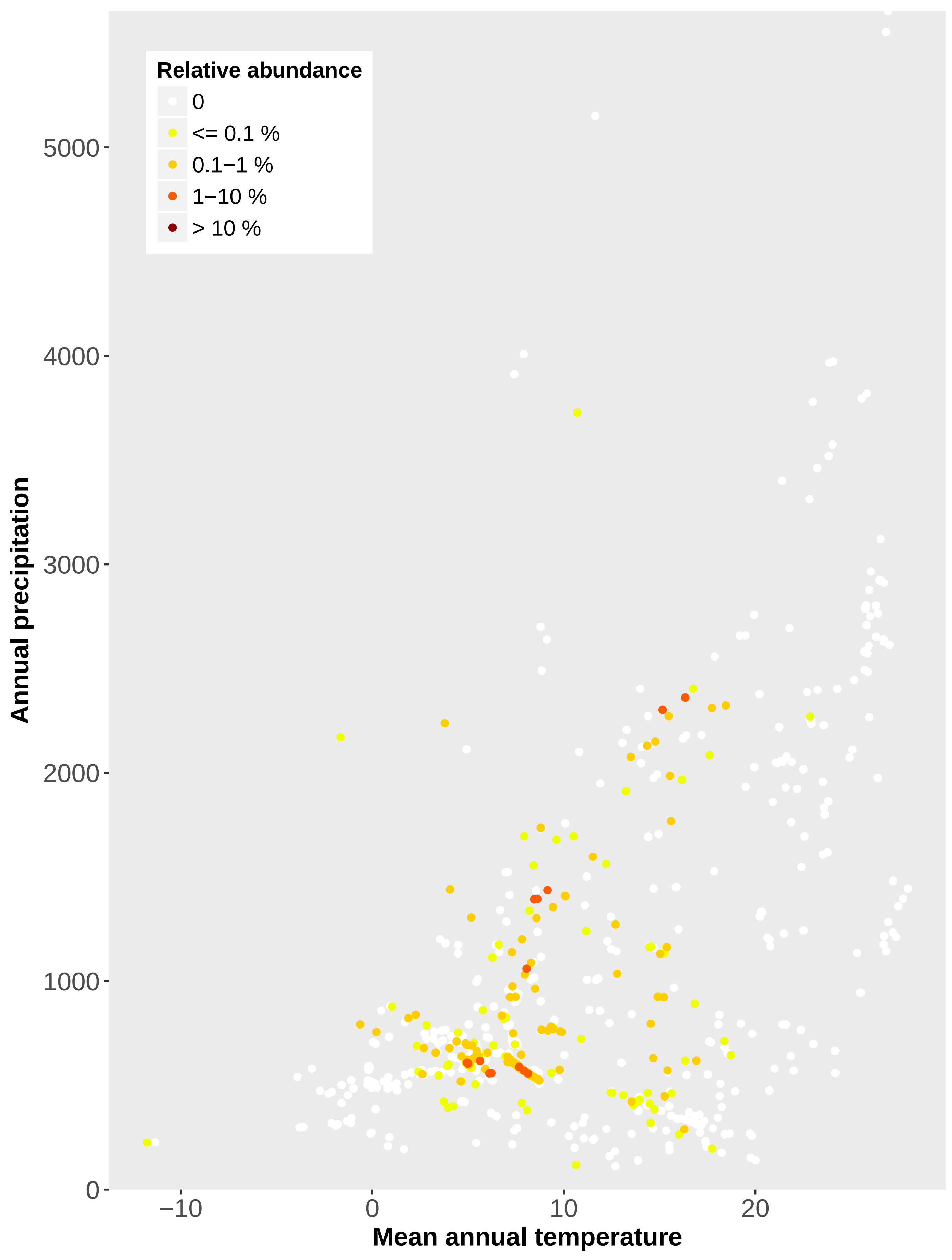

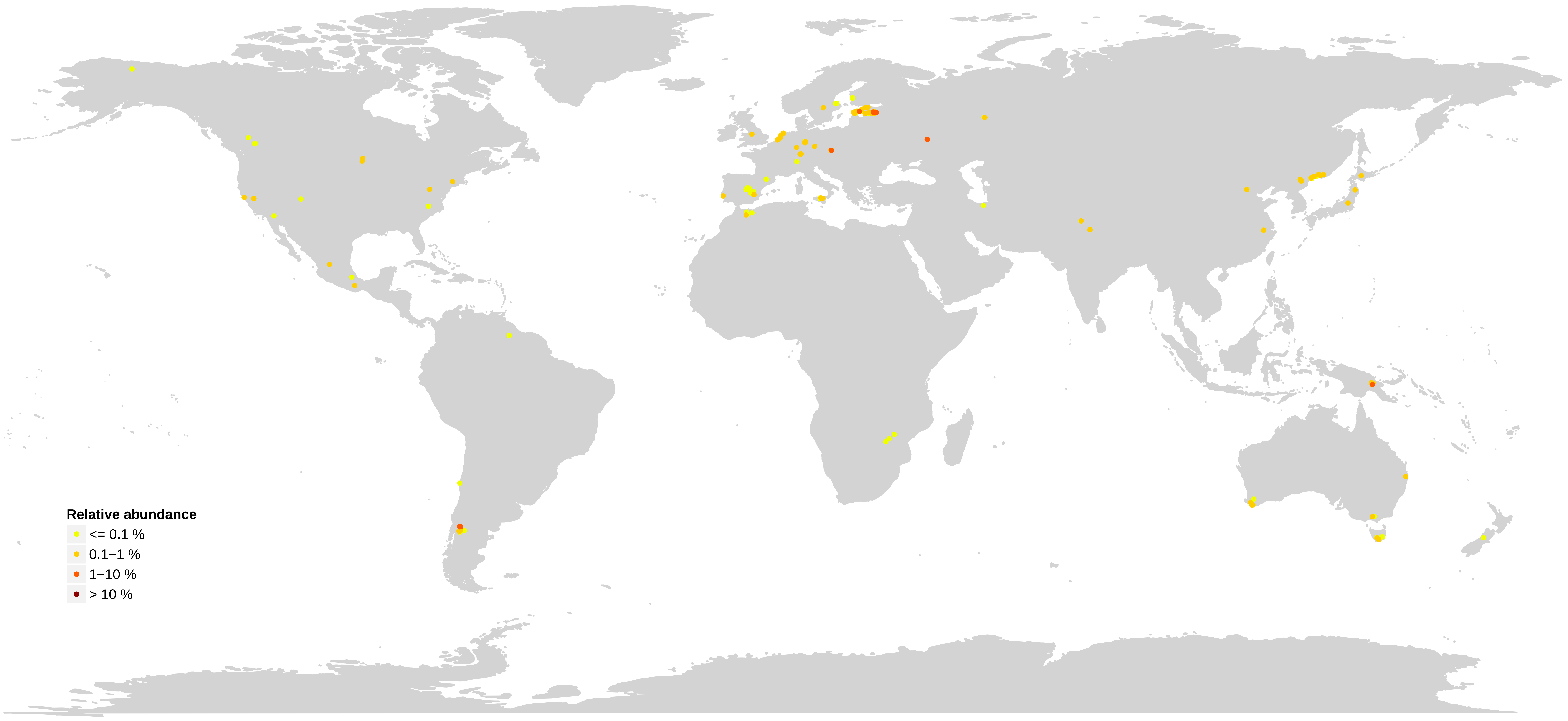

SH187862 Mortierella macrocystopsis

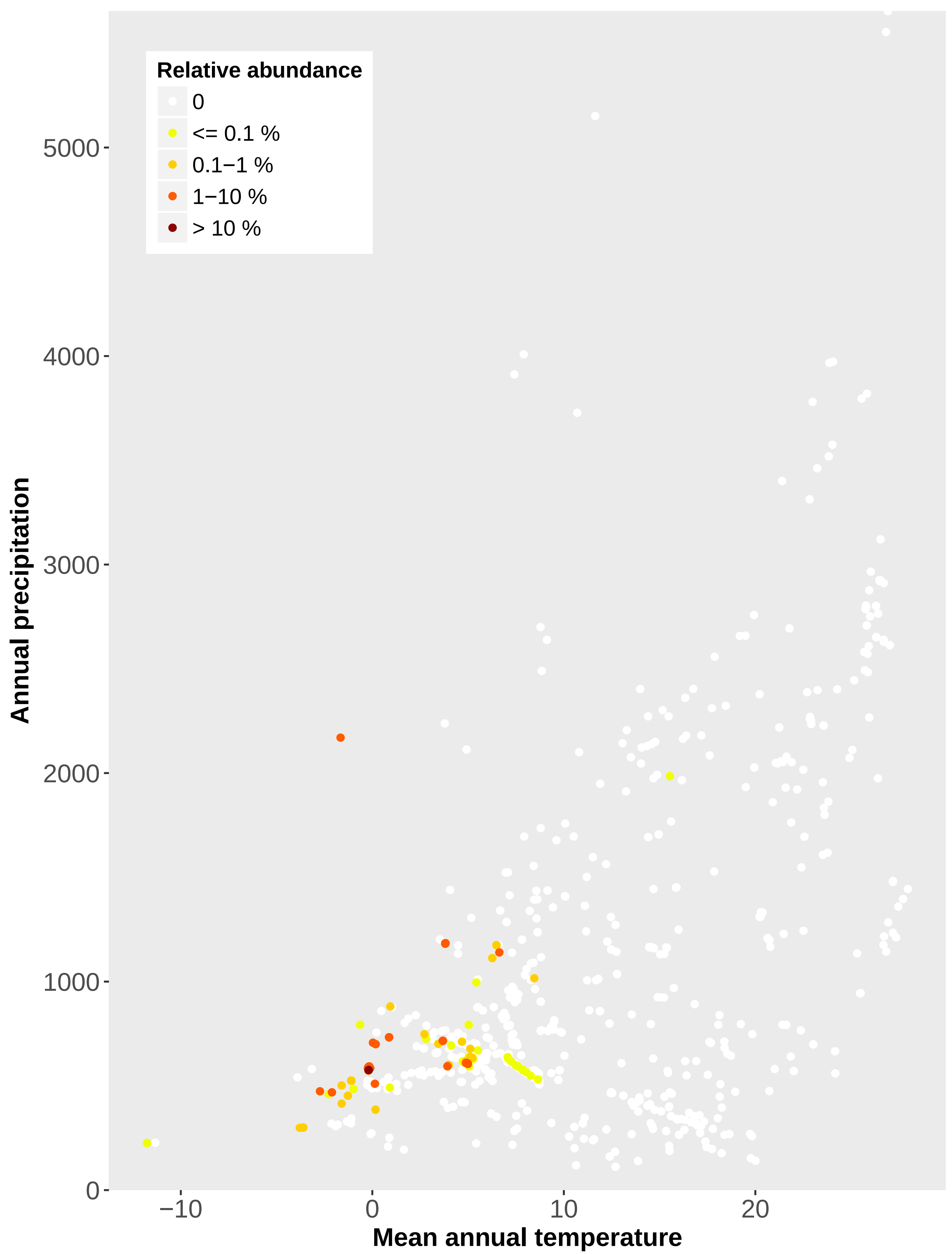

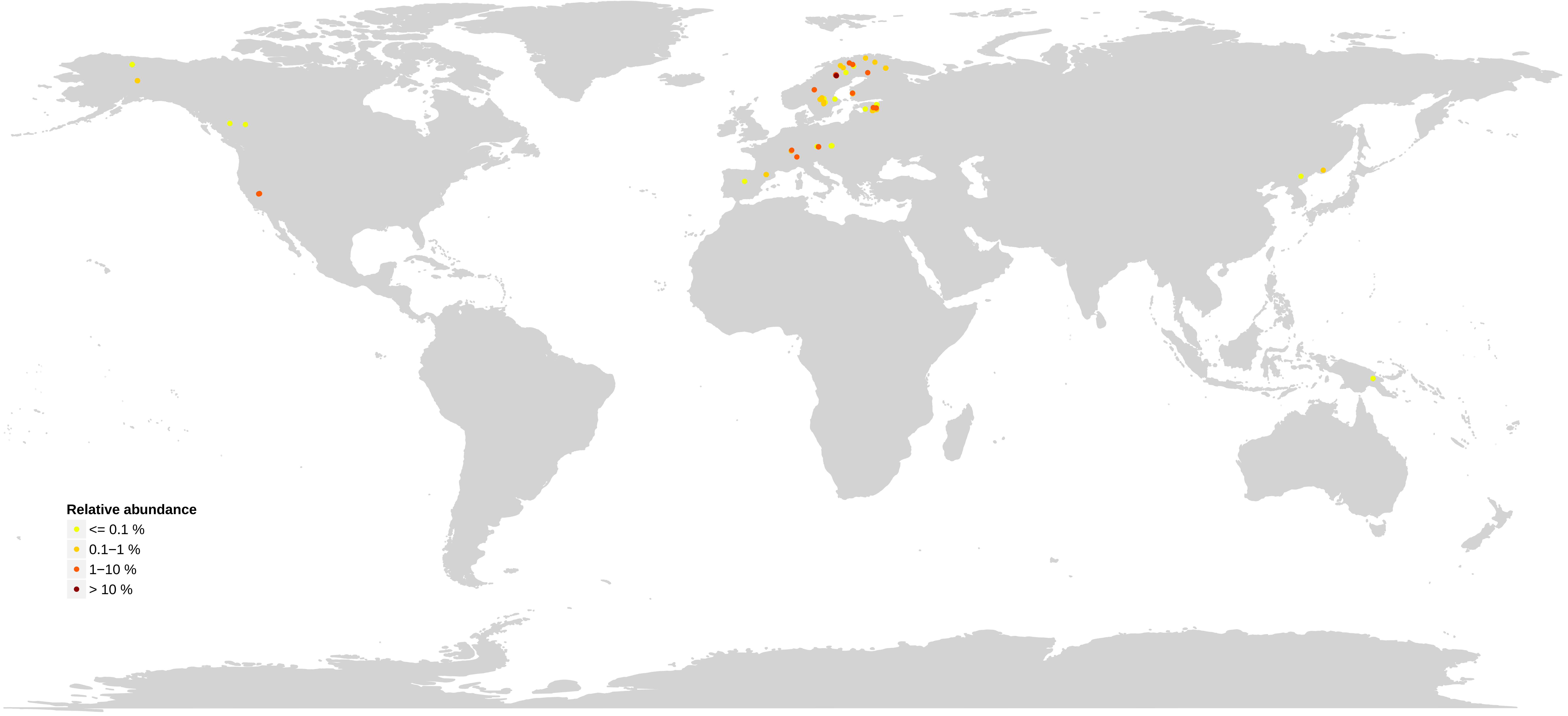

SH193939 Mortierella pulchella

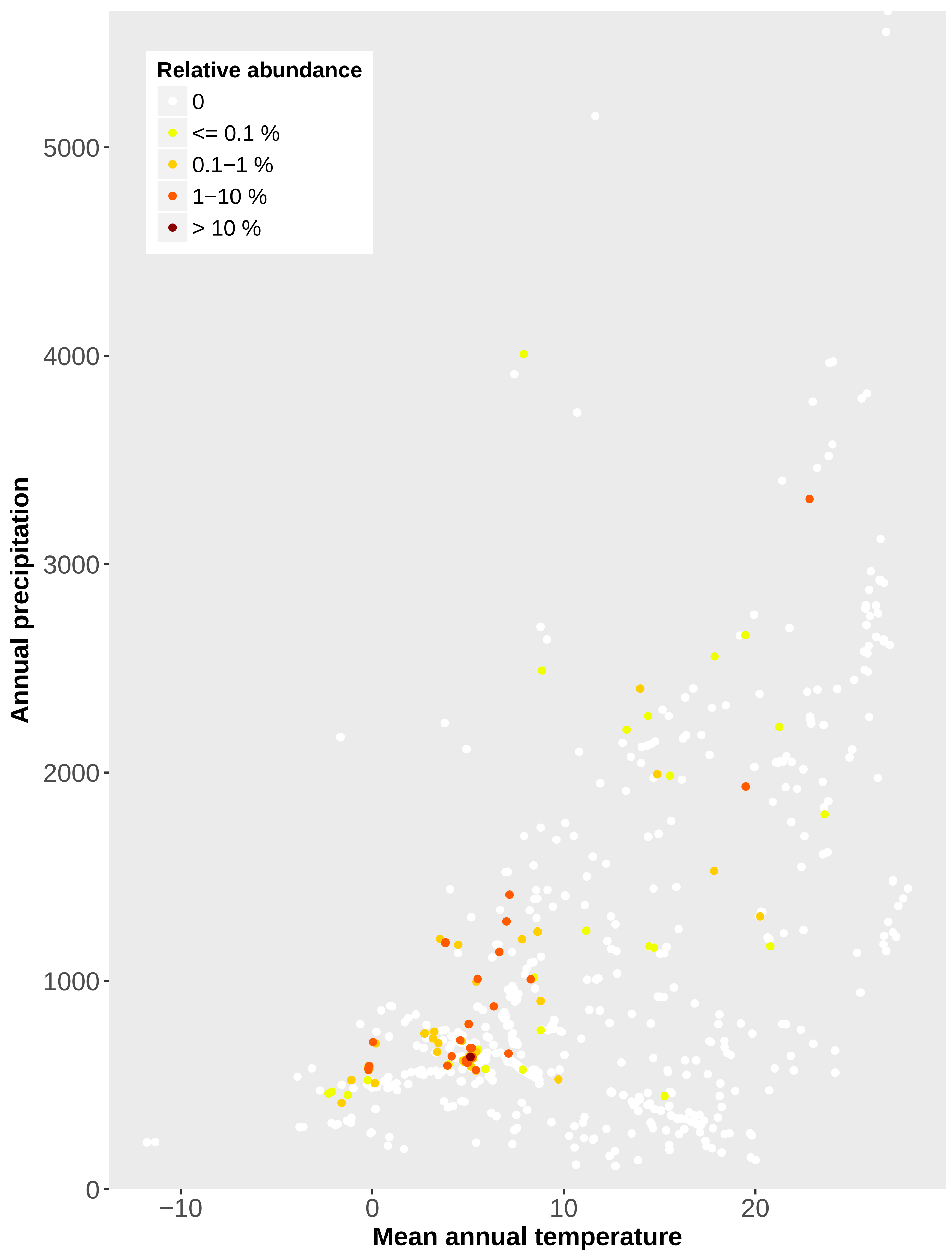

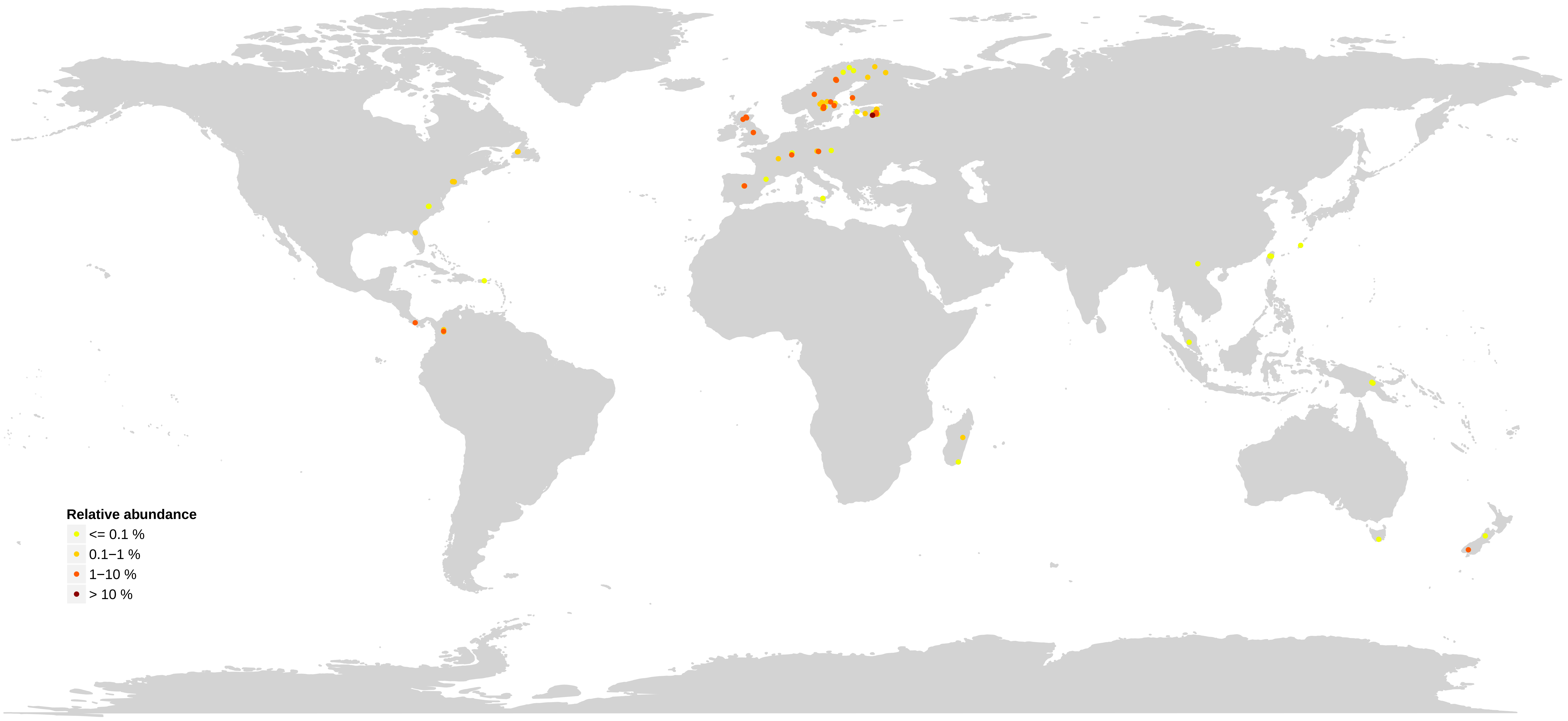

SH196234 Helotiales sp

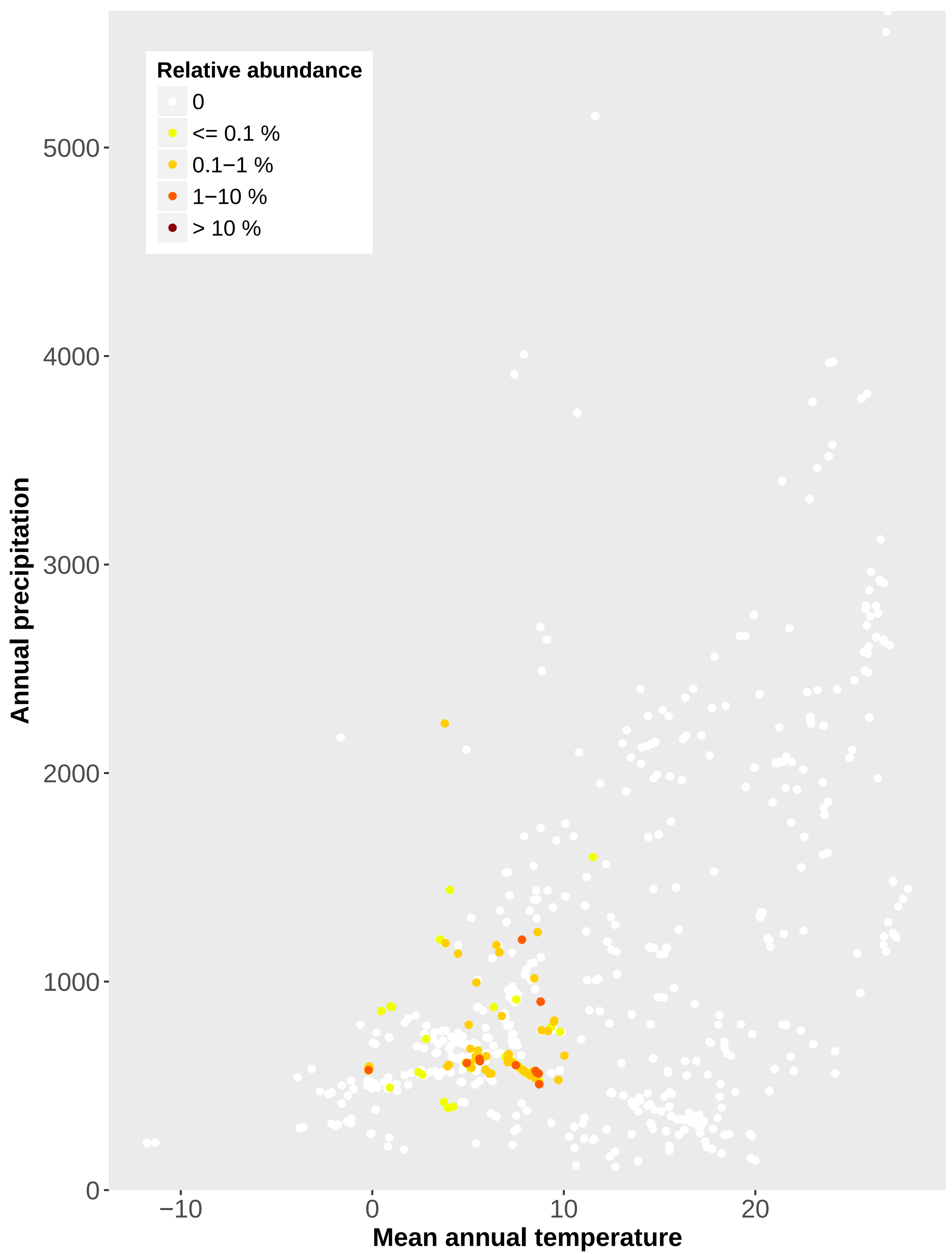

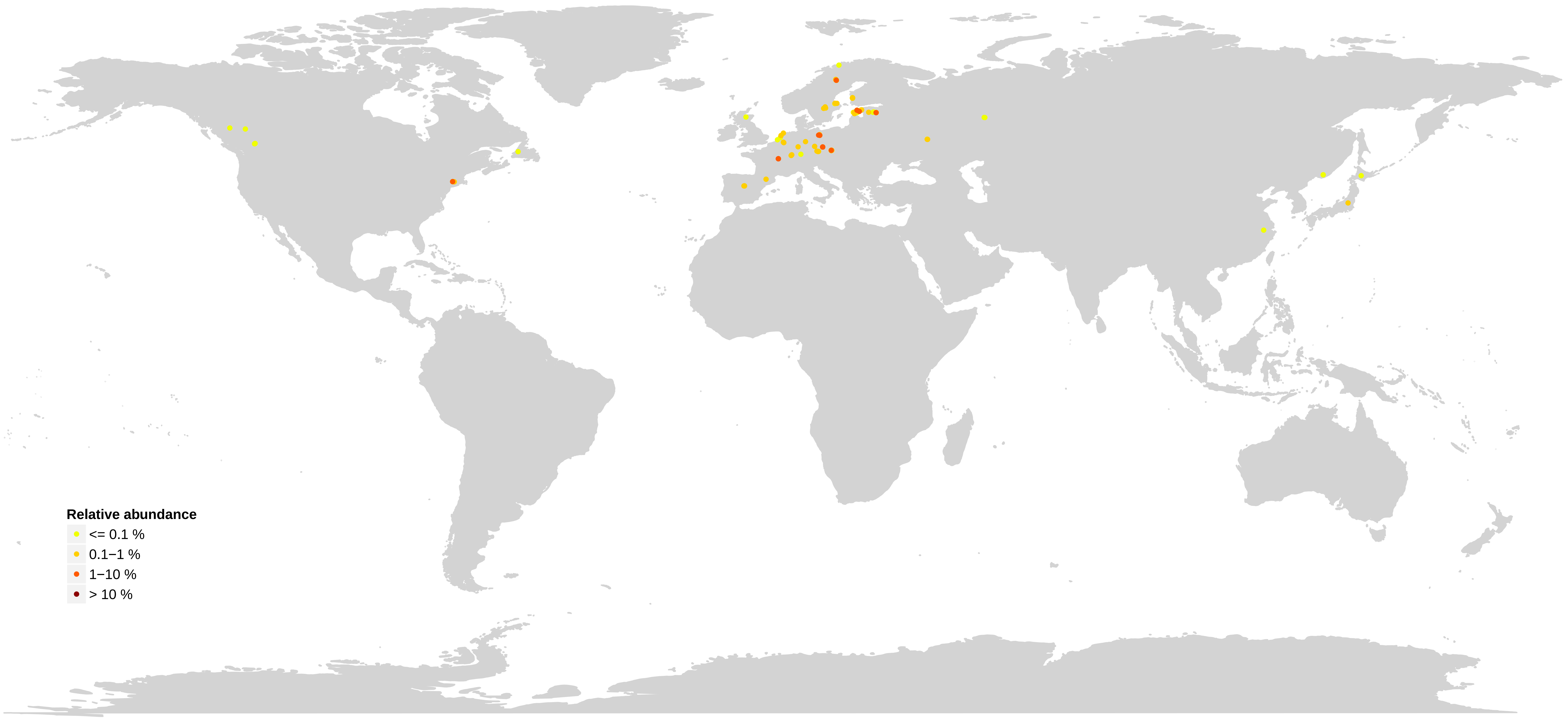

SH495089 Herpotrichiellaceae sp

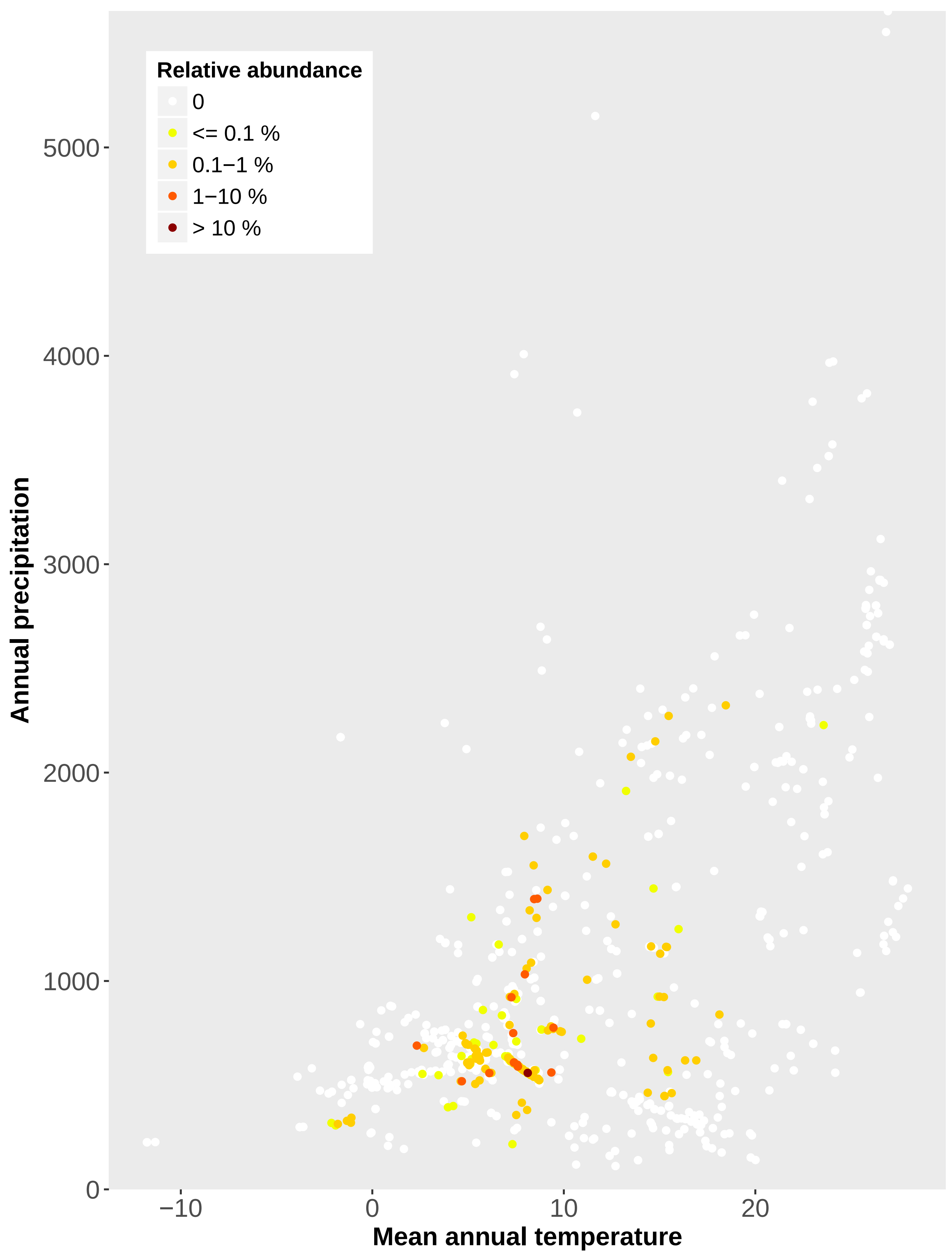

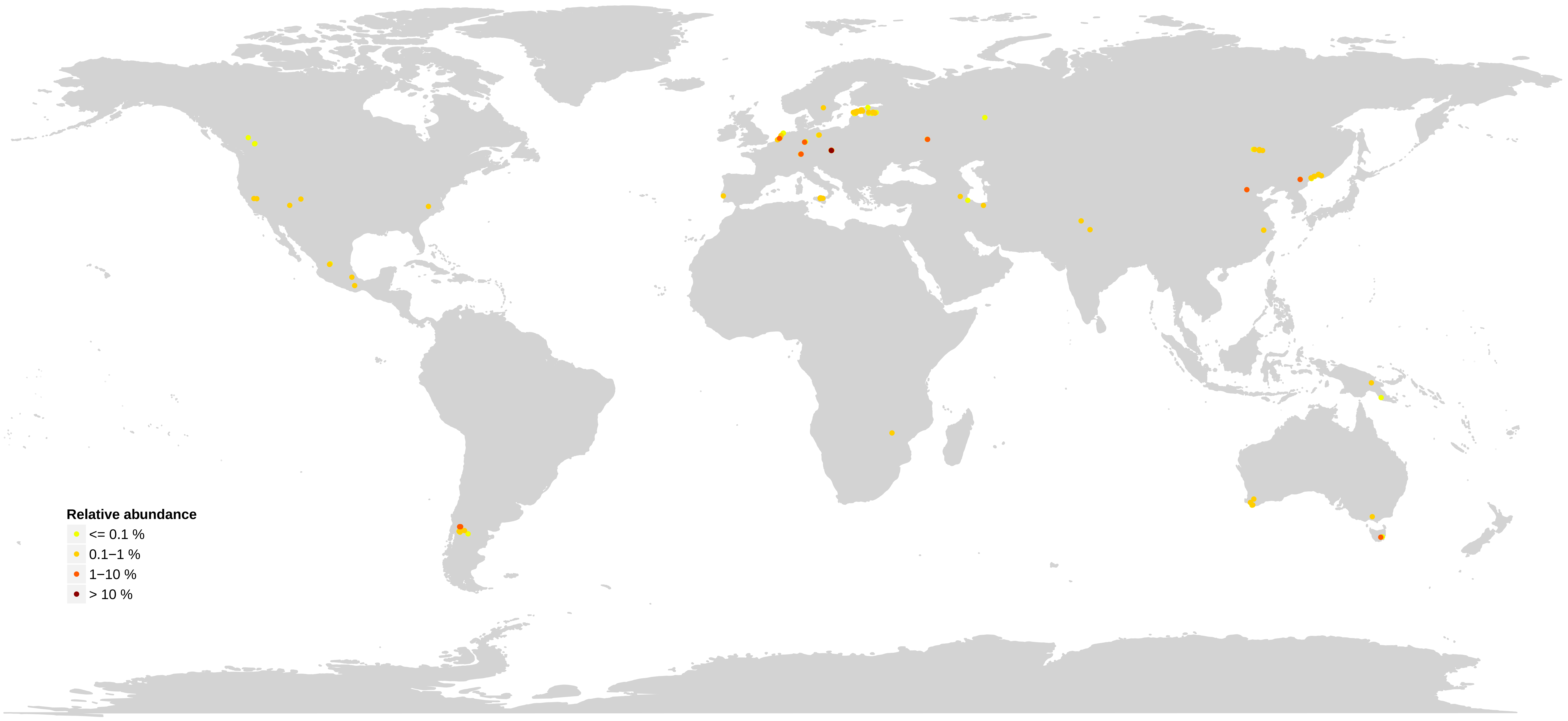

SH205441 Hypocreales sp

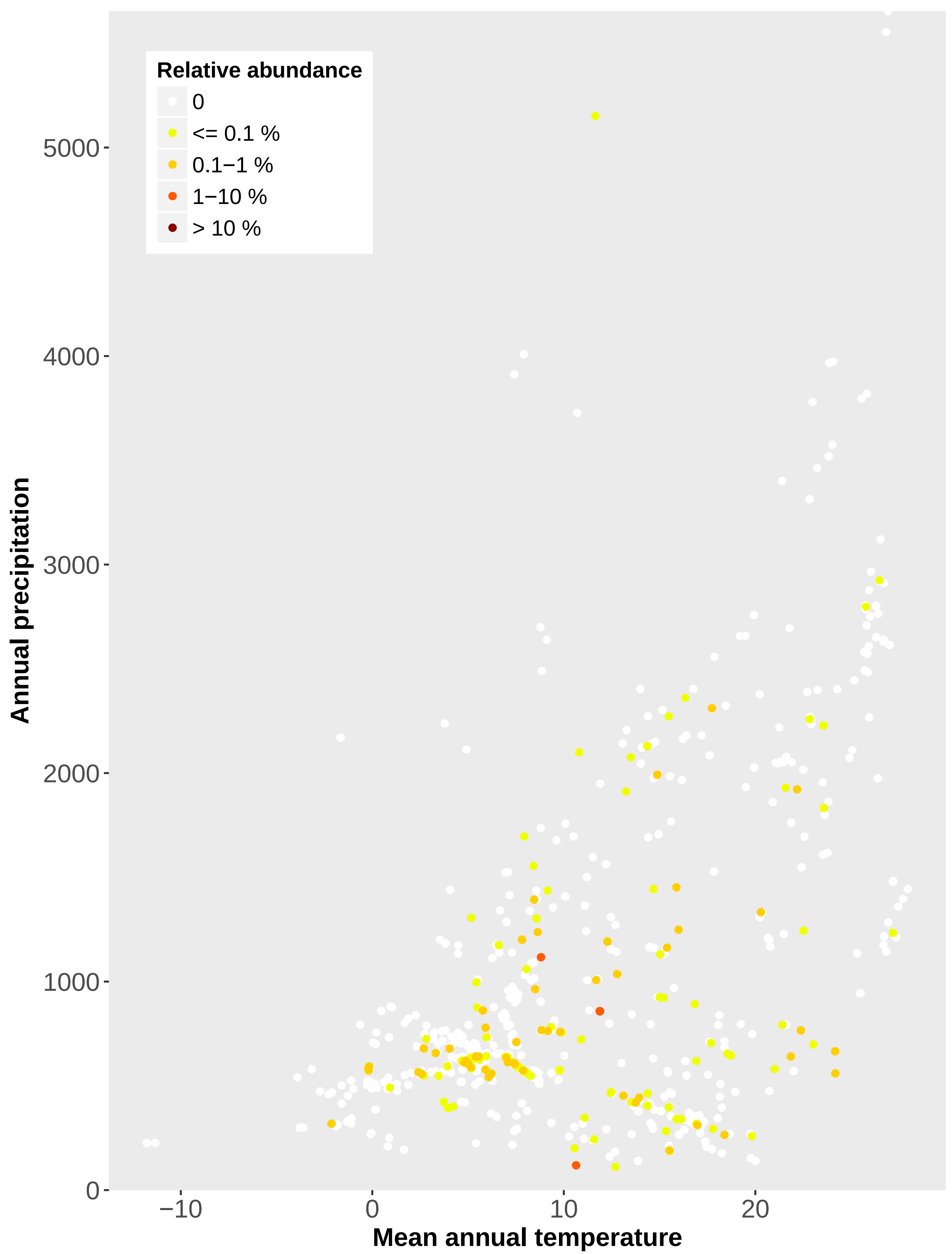

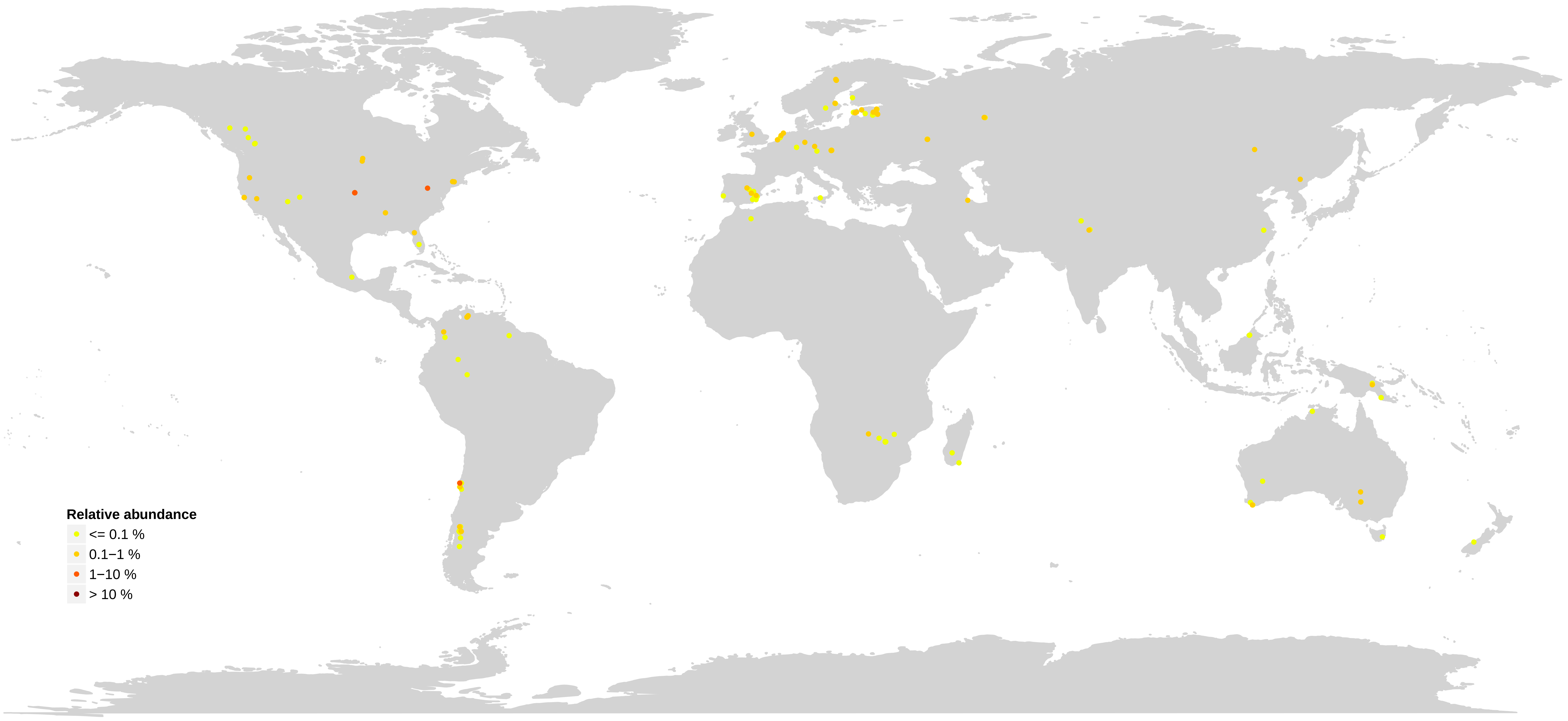

SH186928 Ascomycota sp

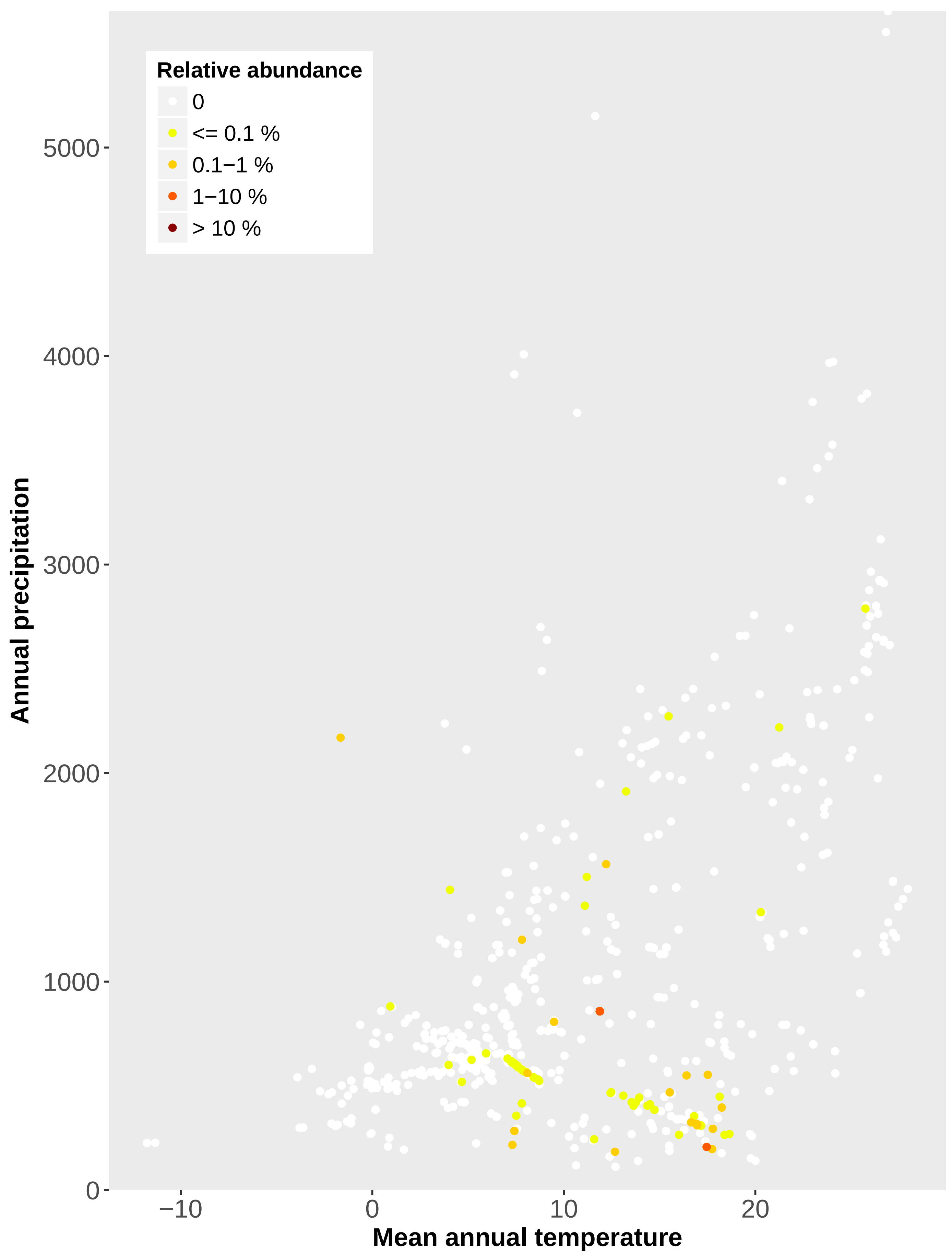

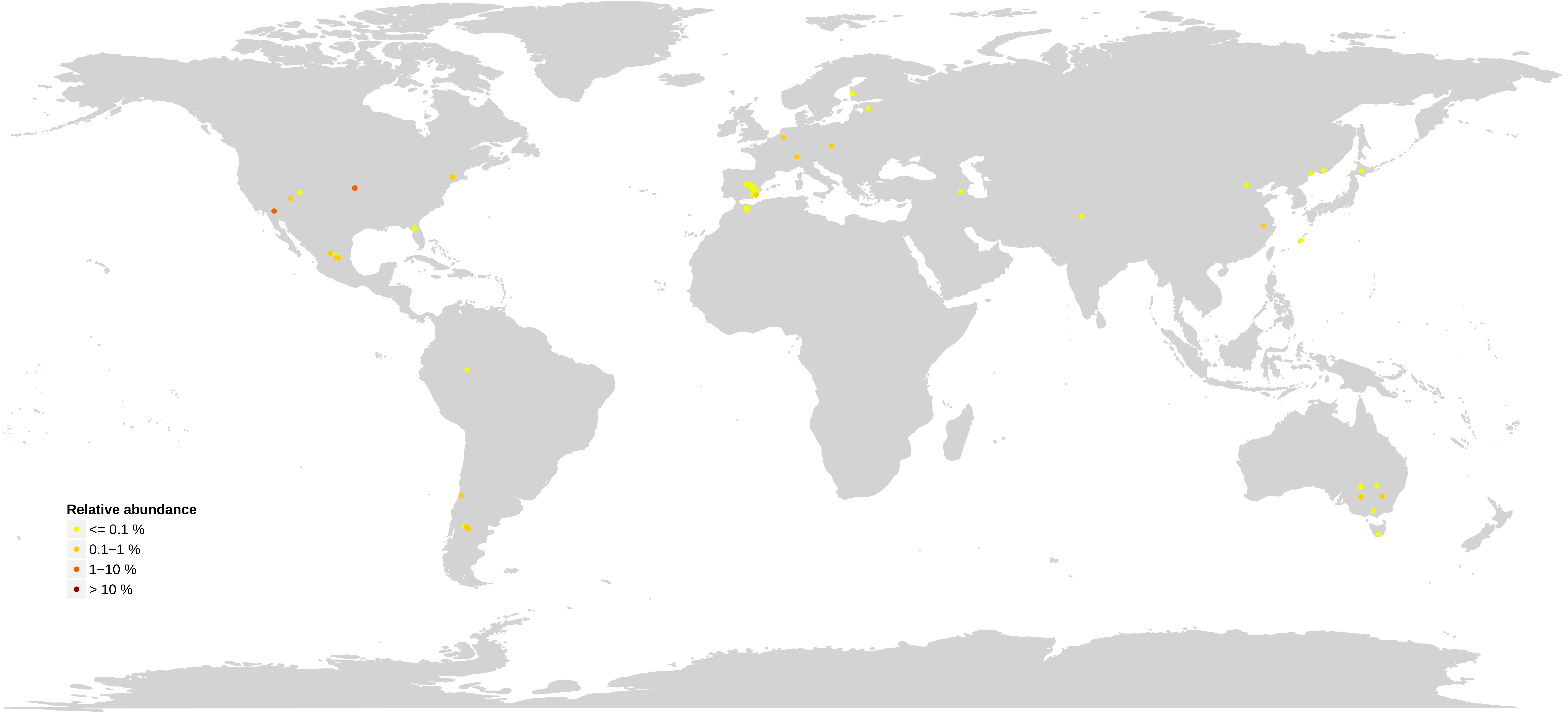

SH182483 Penicillium decumbens

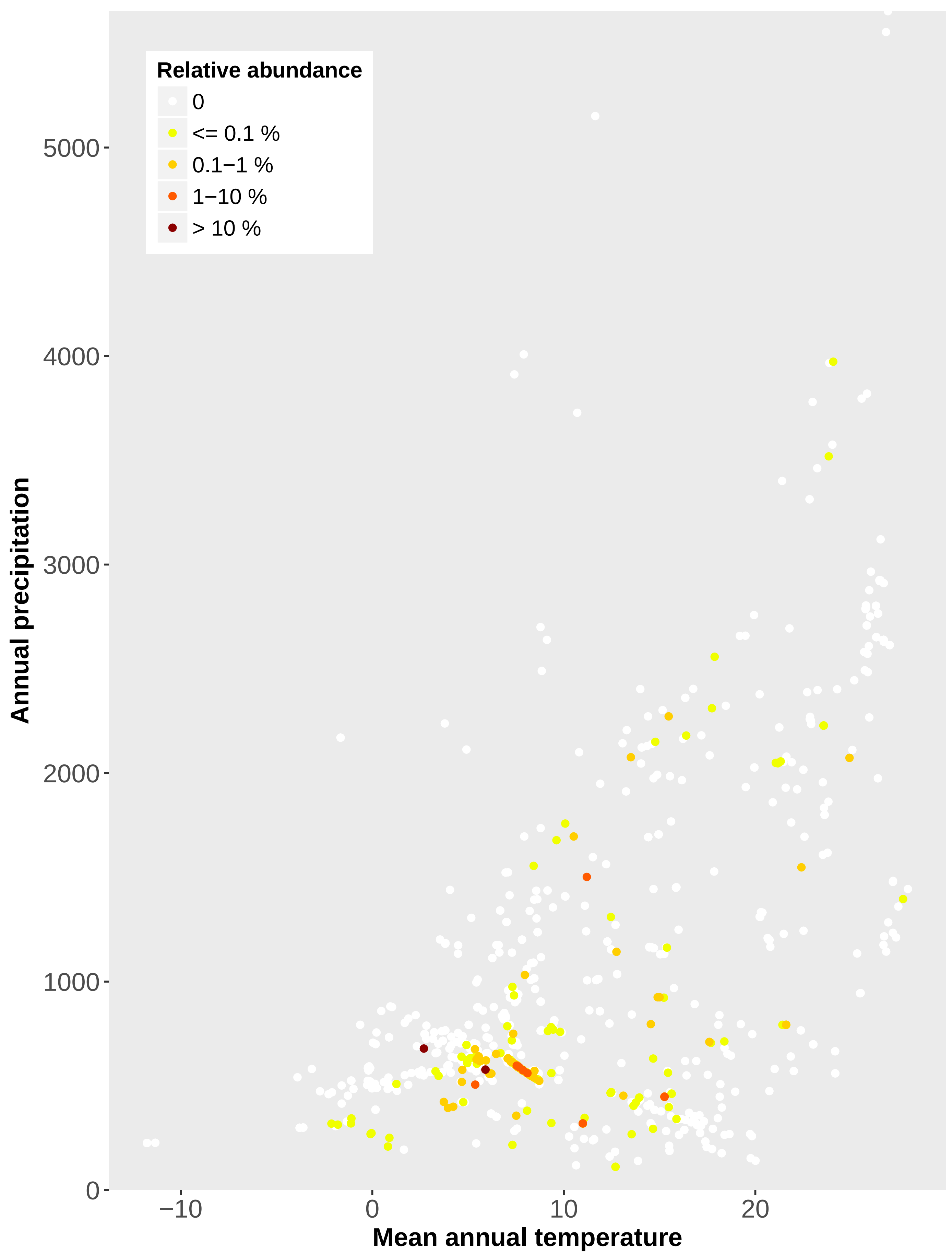

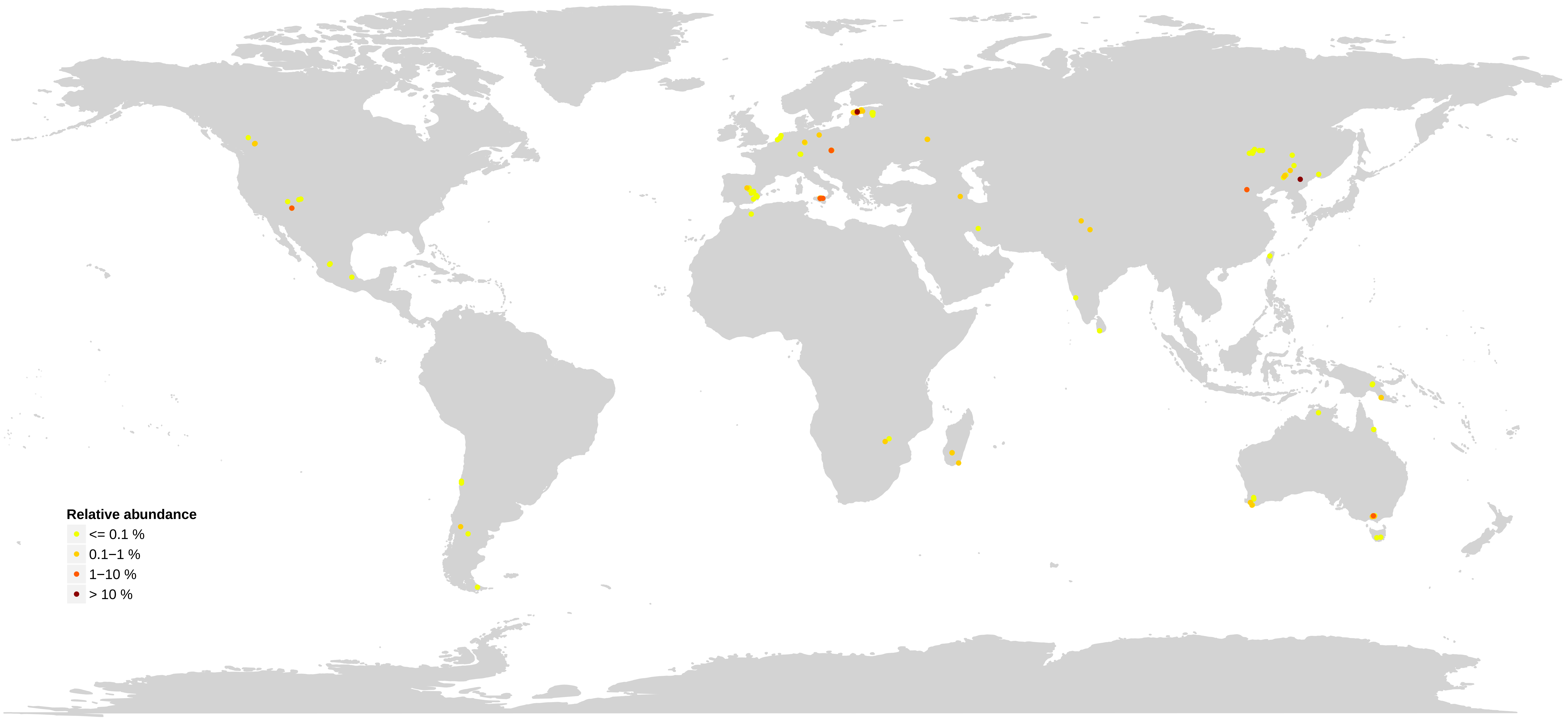

SH180110 Mortierella gamsii

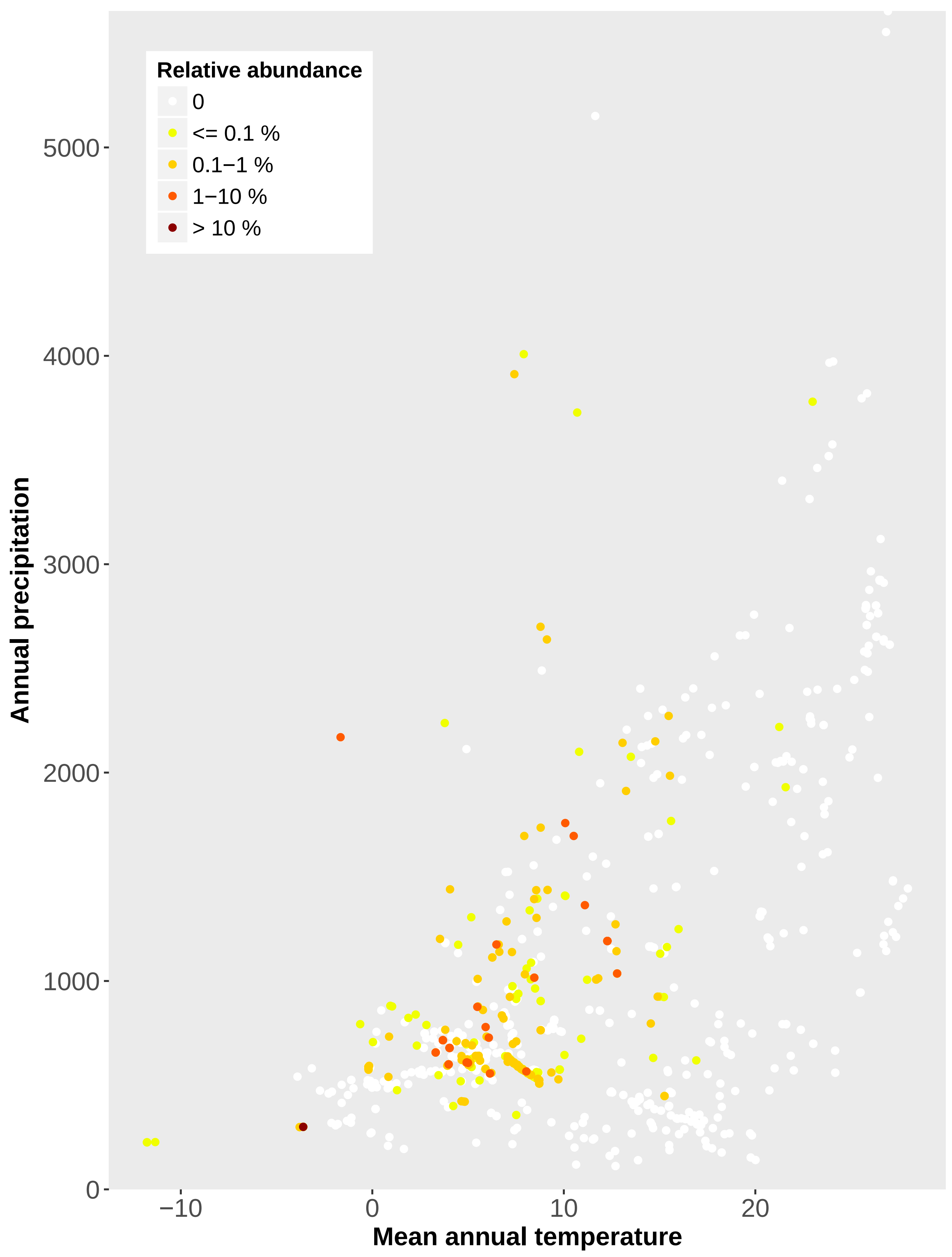

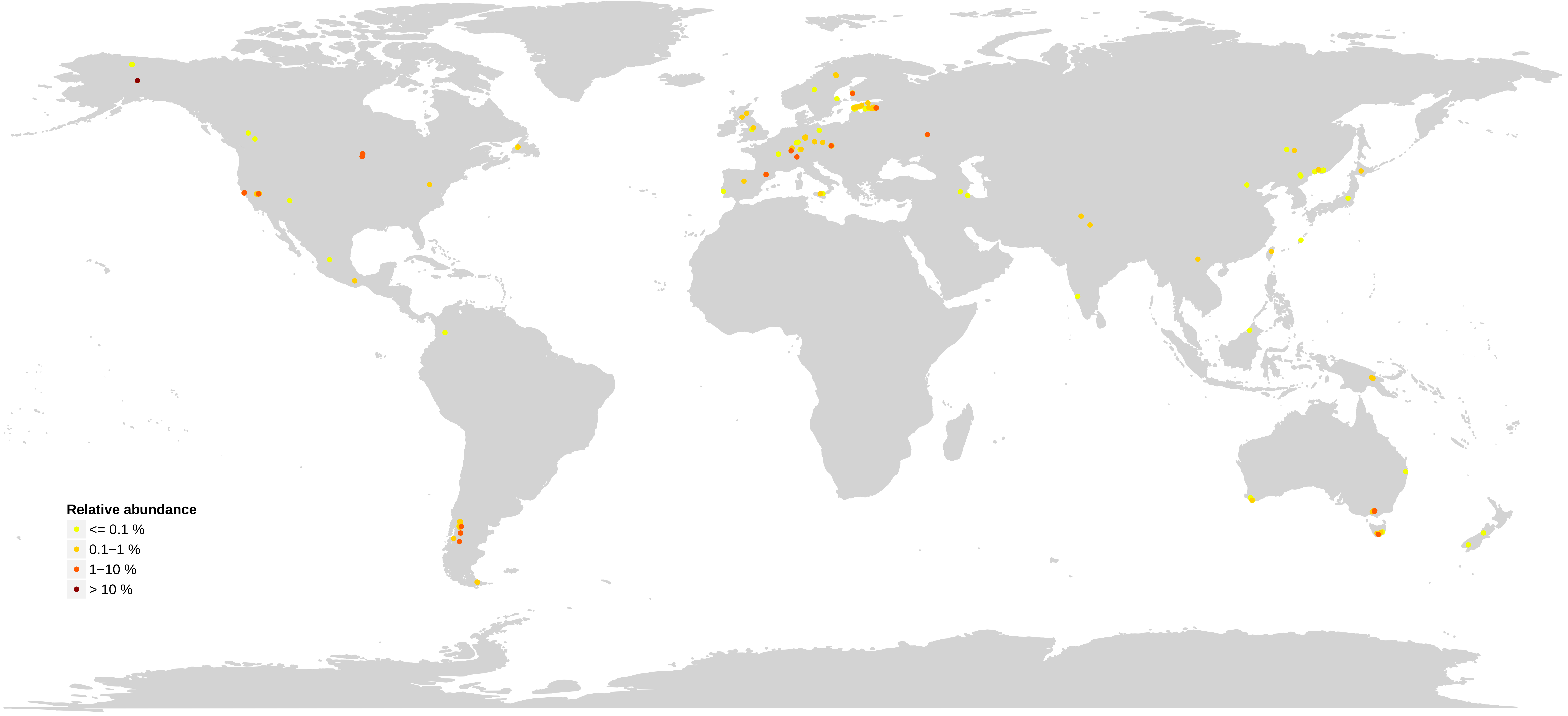

SH496541 Fungi sp

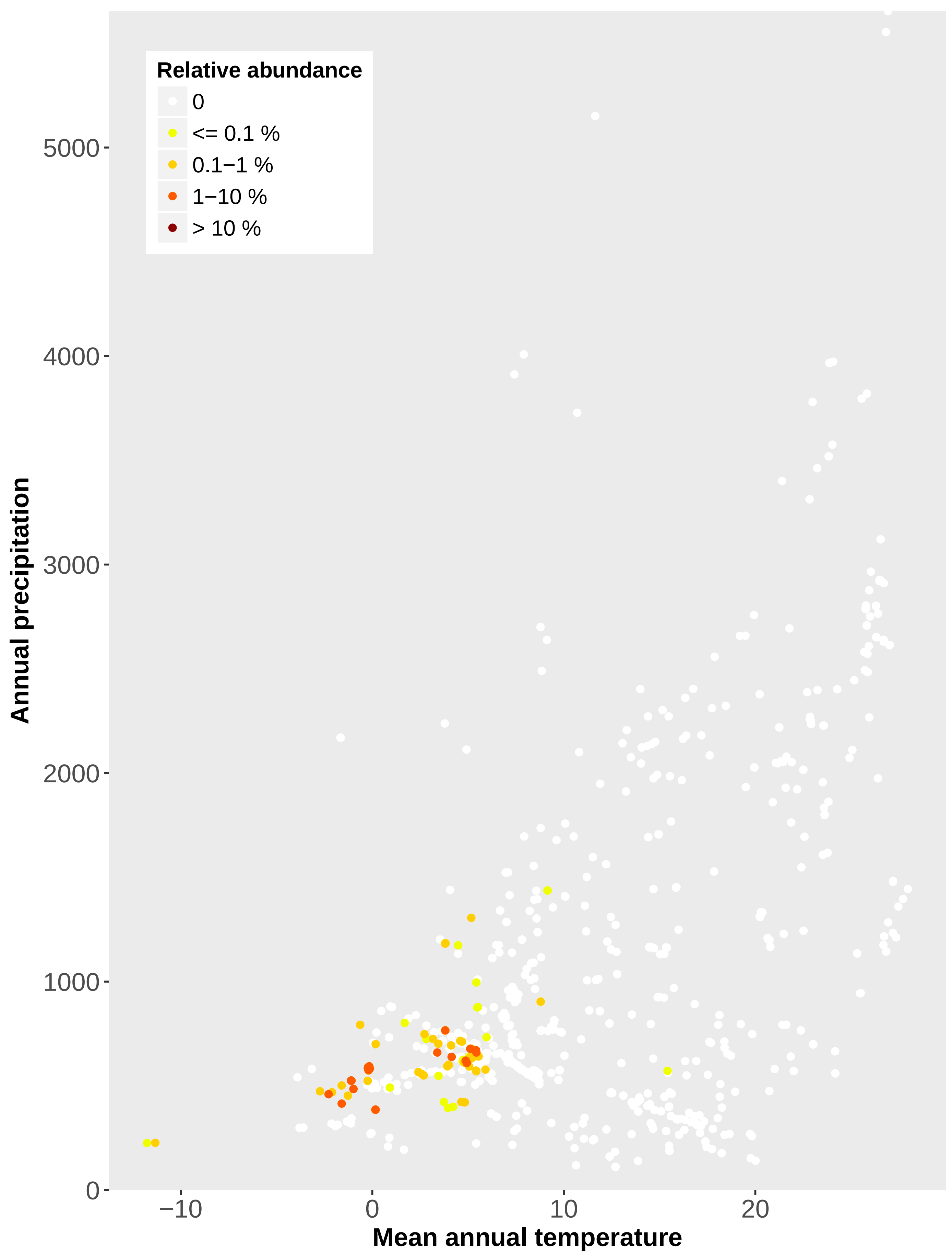

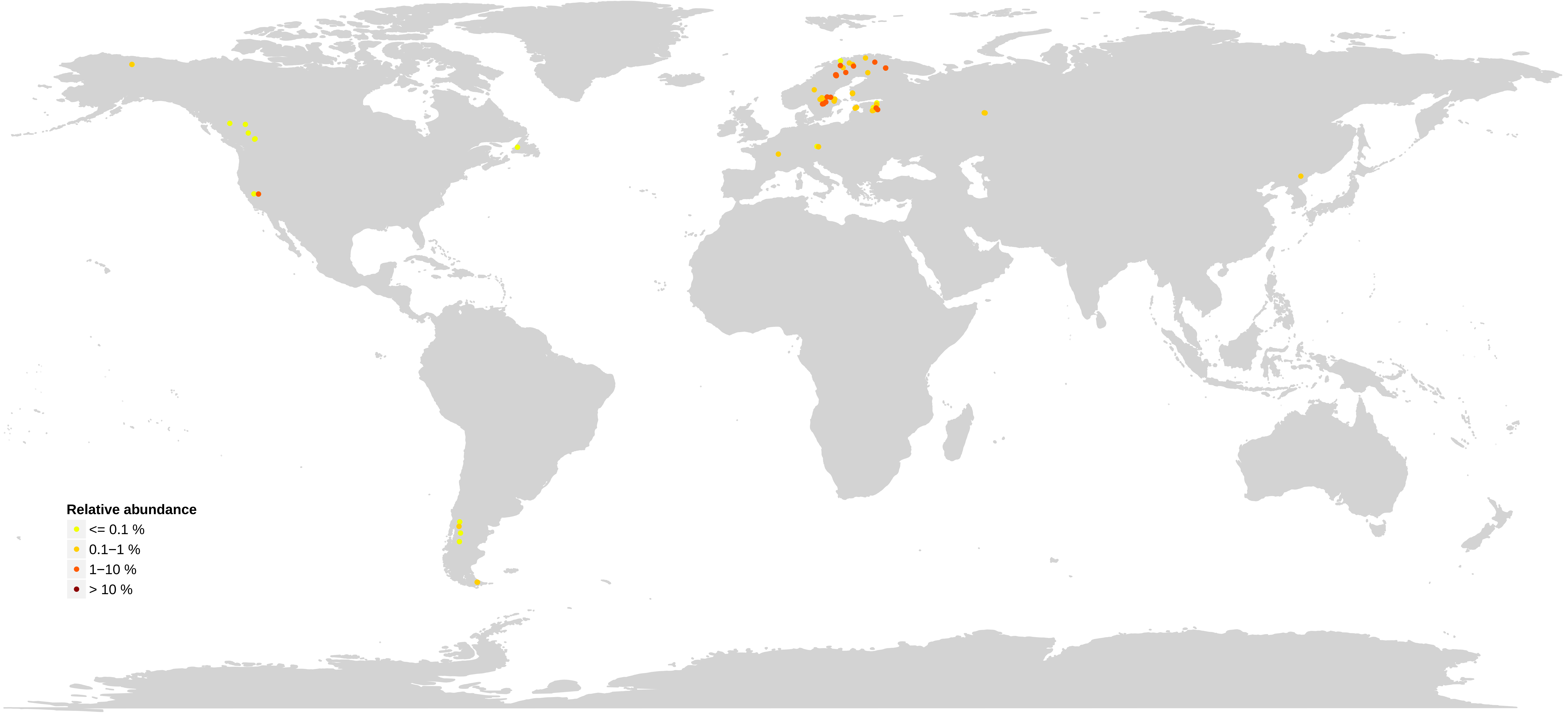

SH182180 Tremellomycetes sp

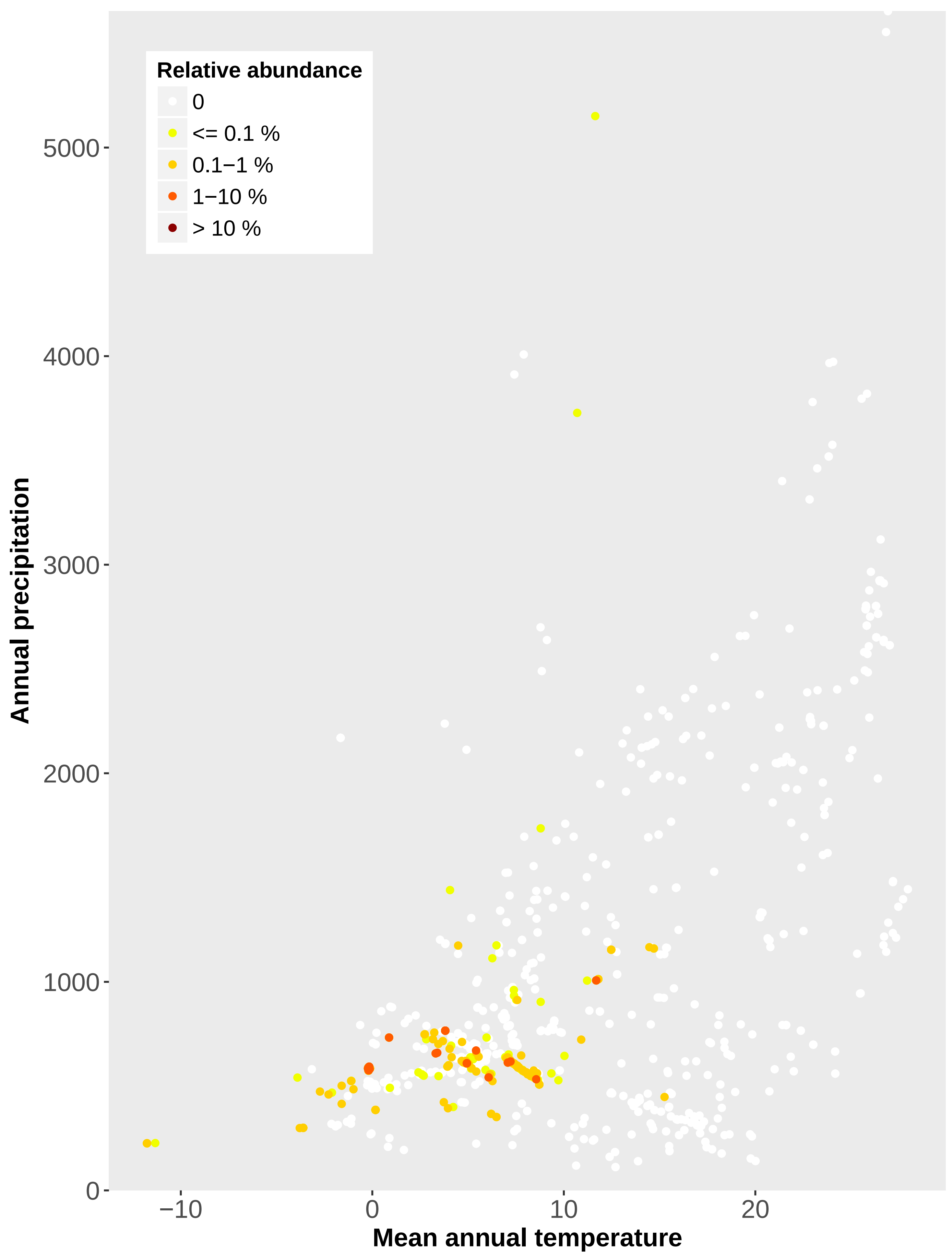

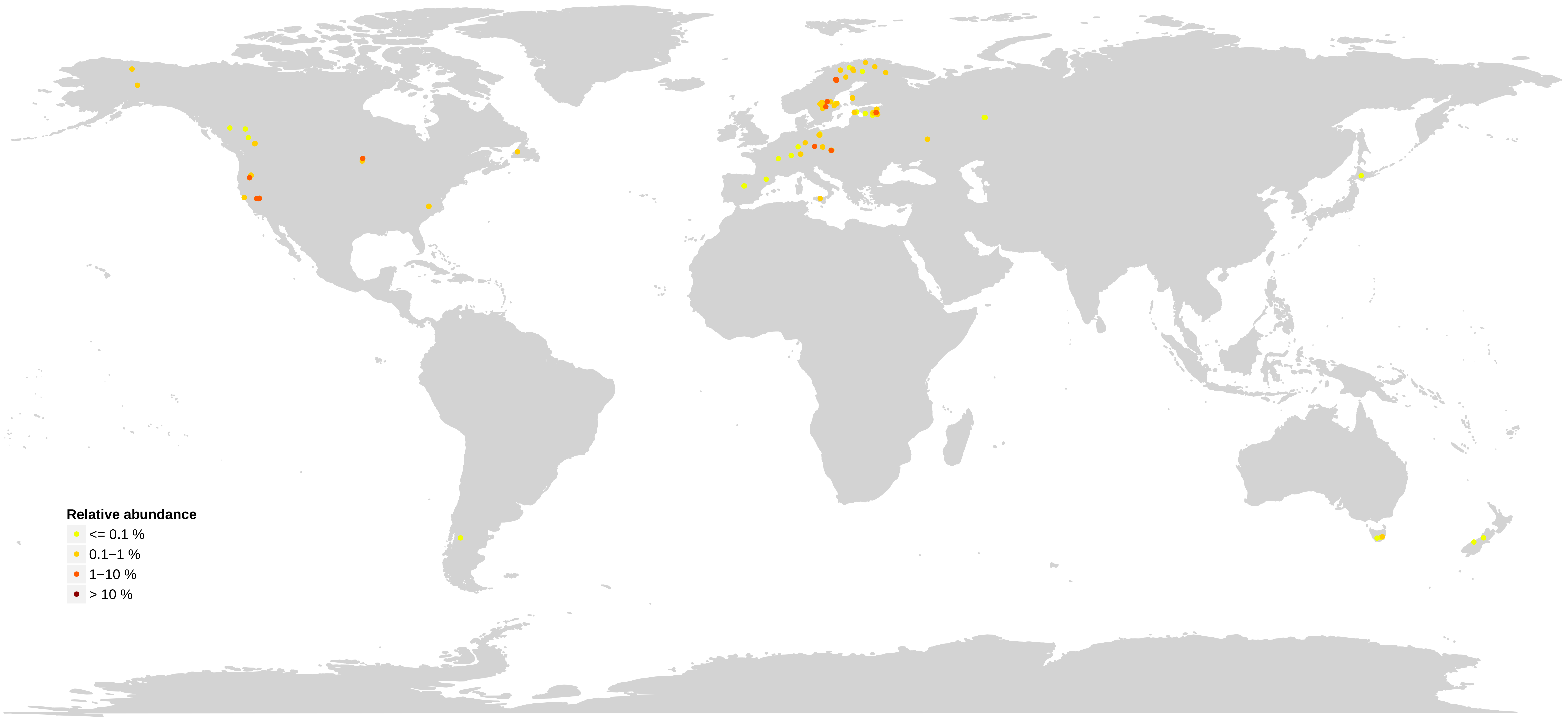

SH287579 Beauveria pseudobassiana

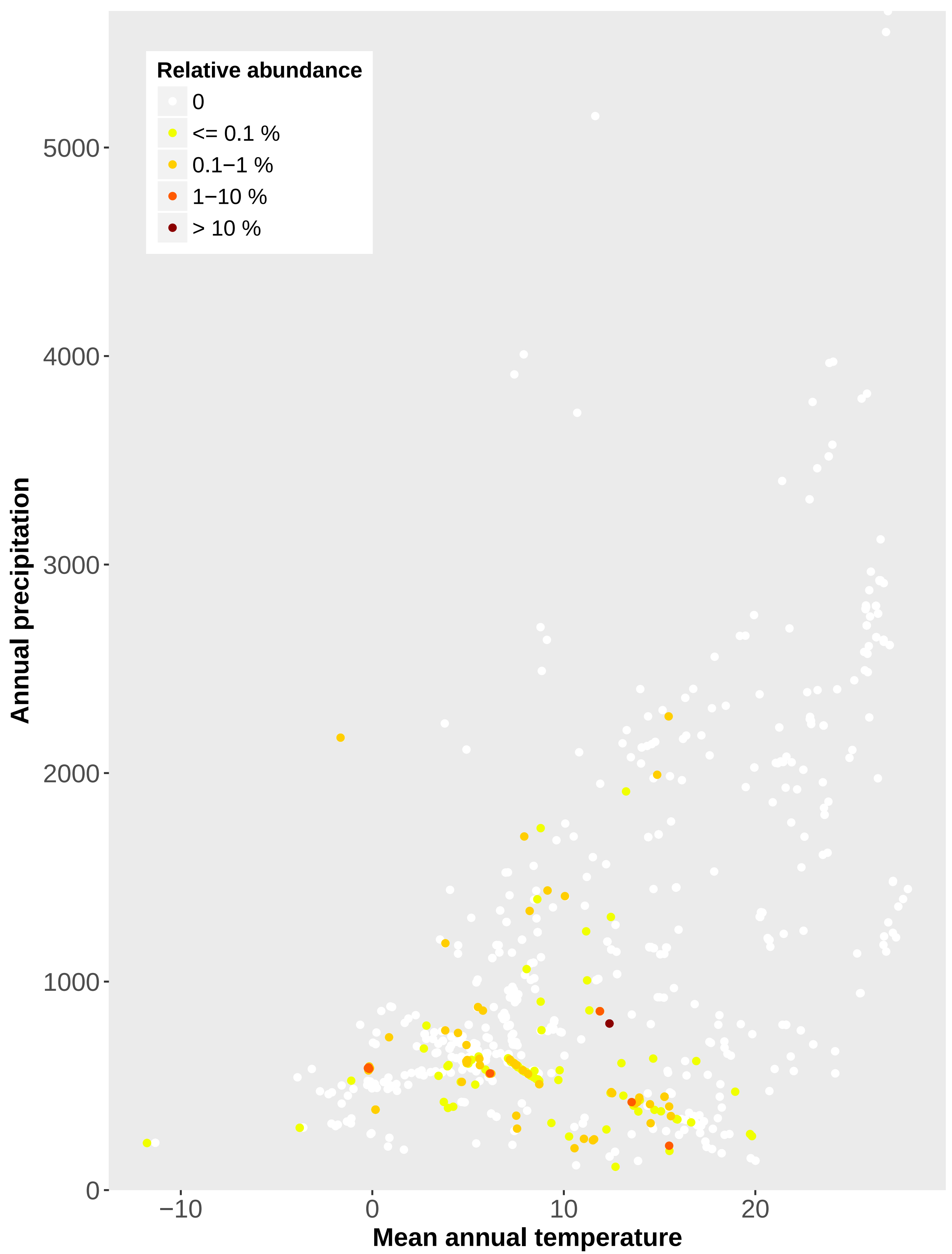

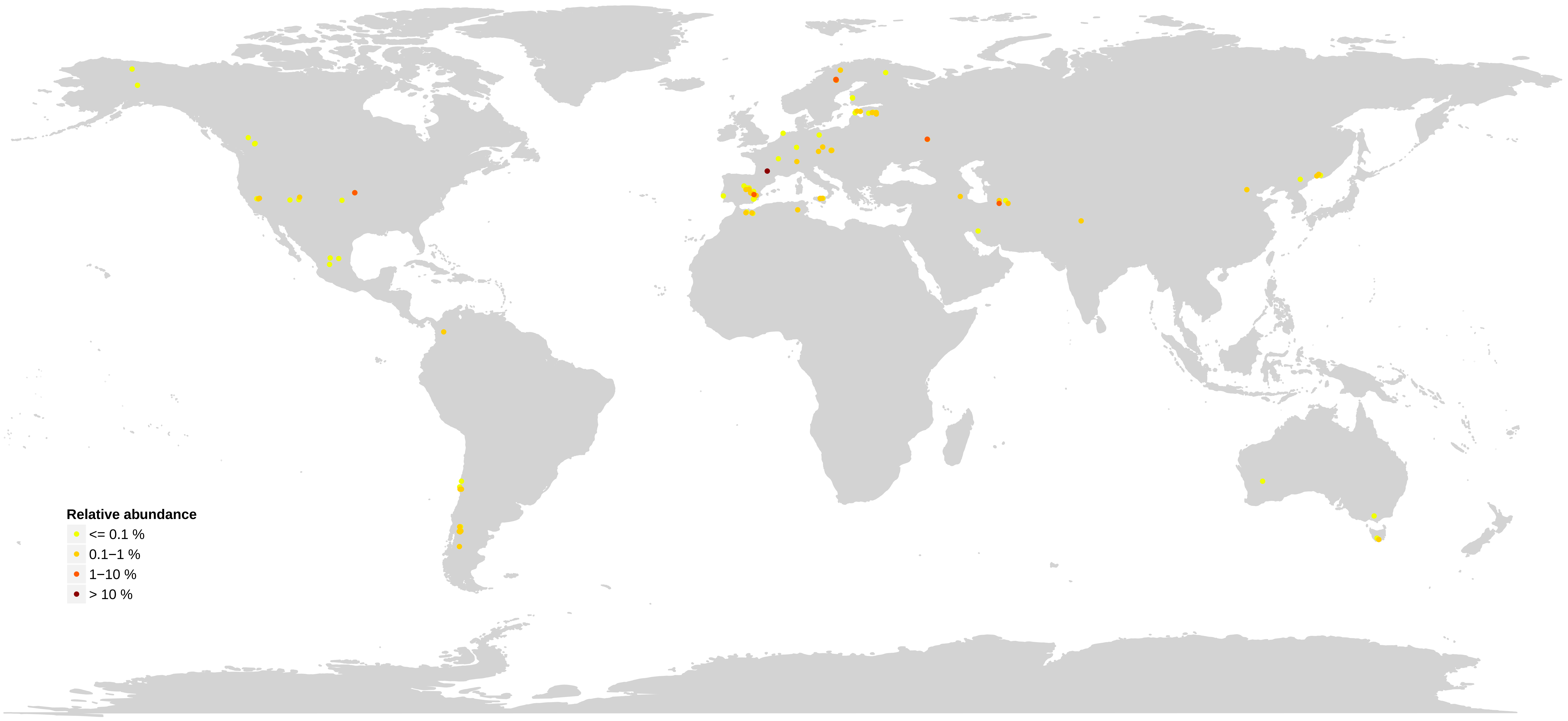

SH219615 Venturia sp

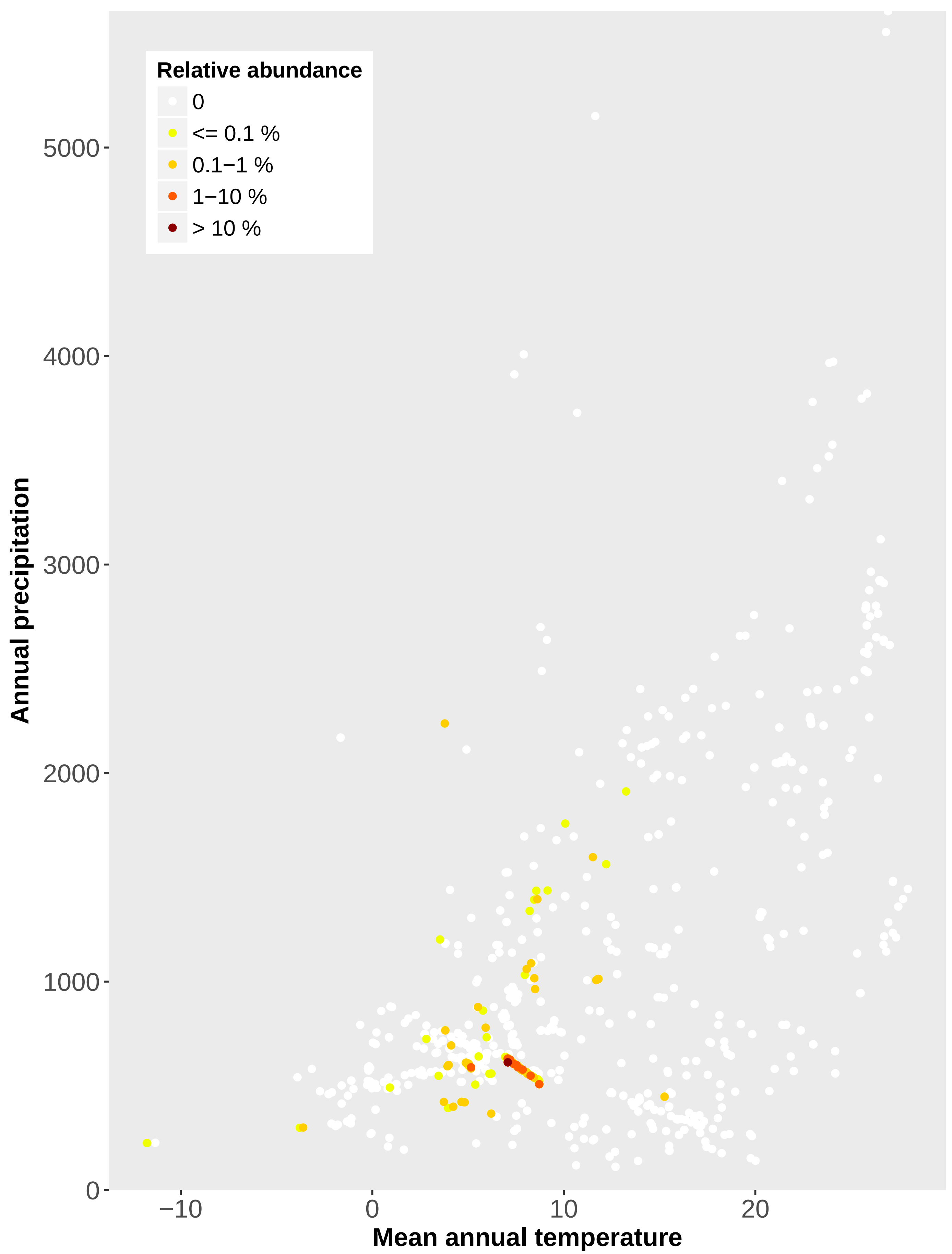

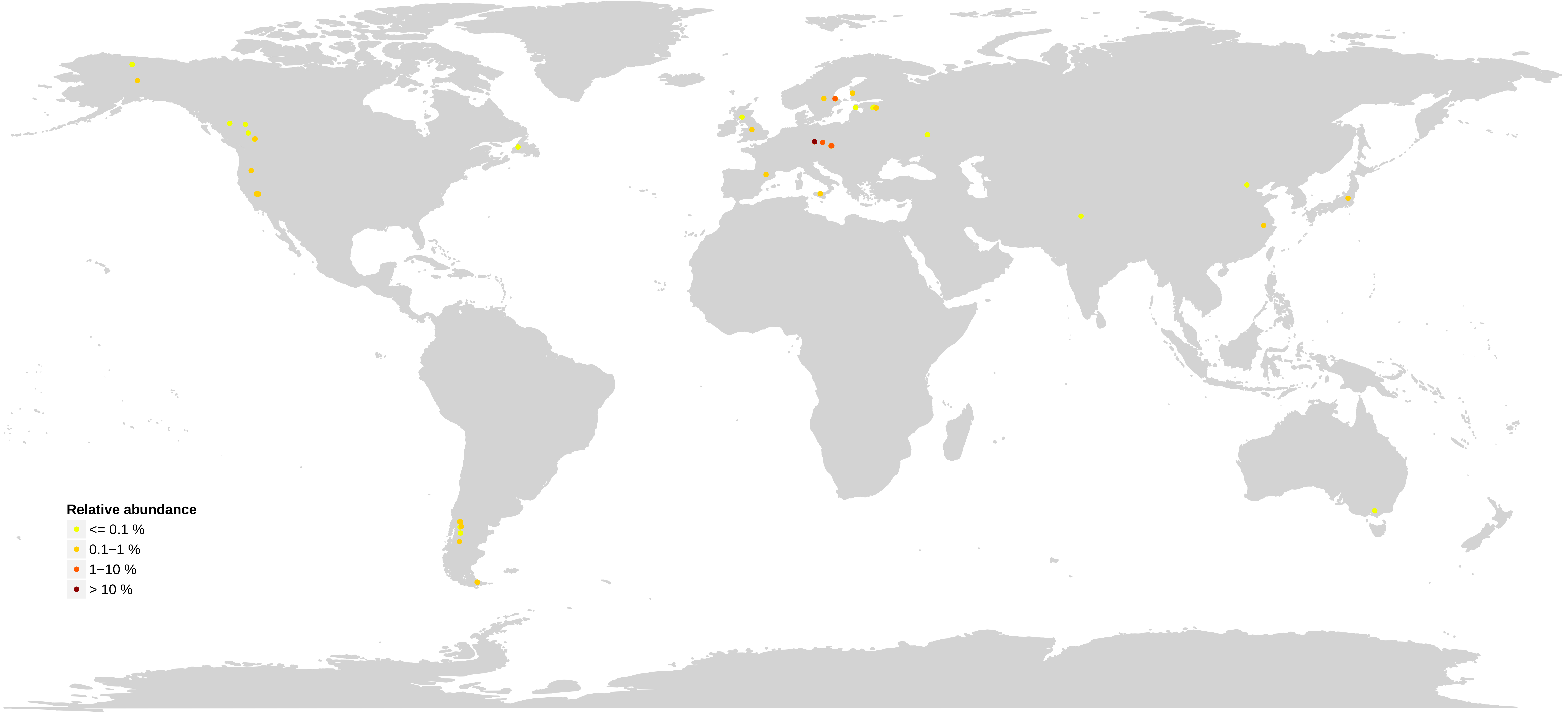

SH196475 Helotiales sp

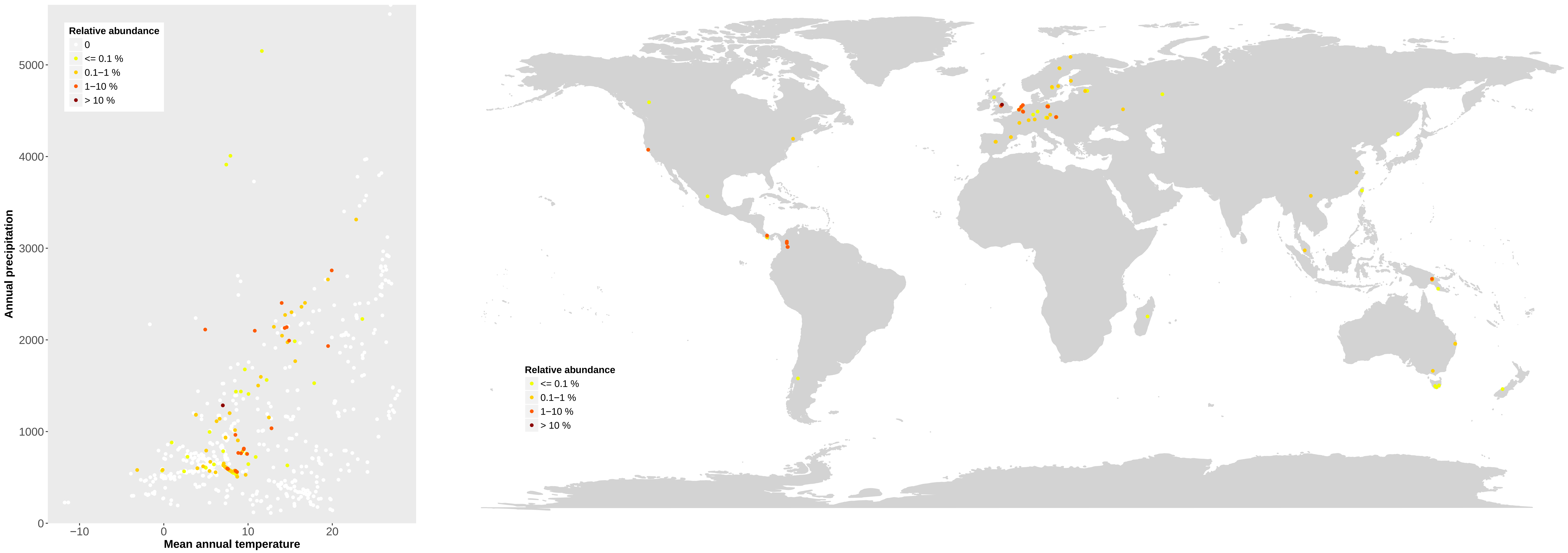

SH193238 Cladophialophora sp

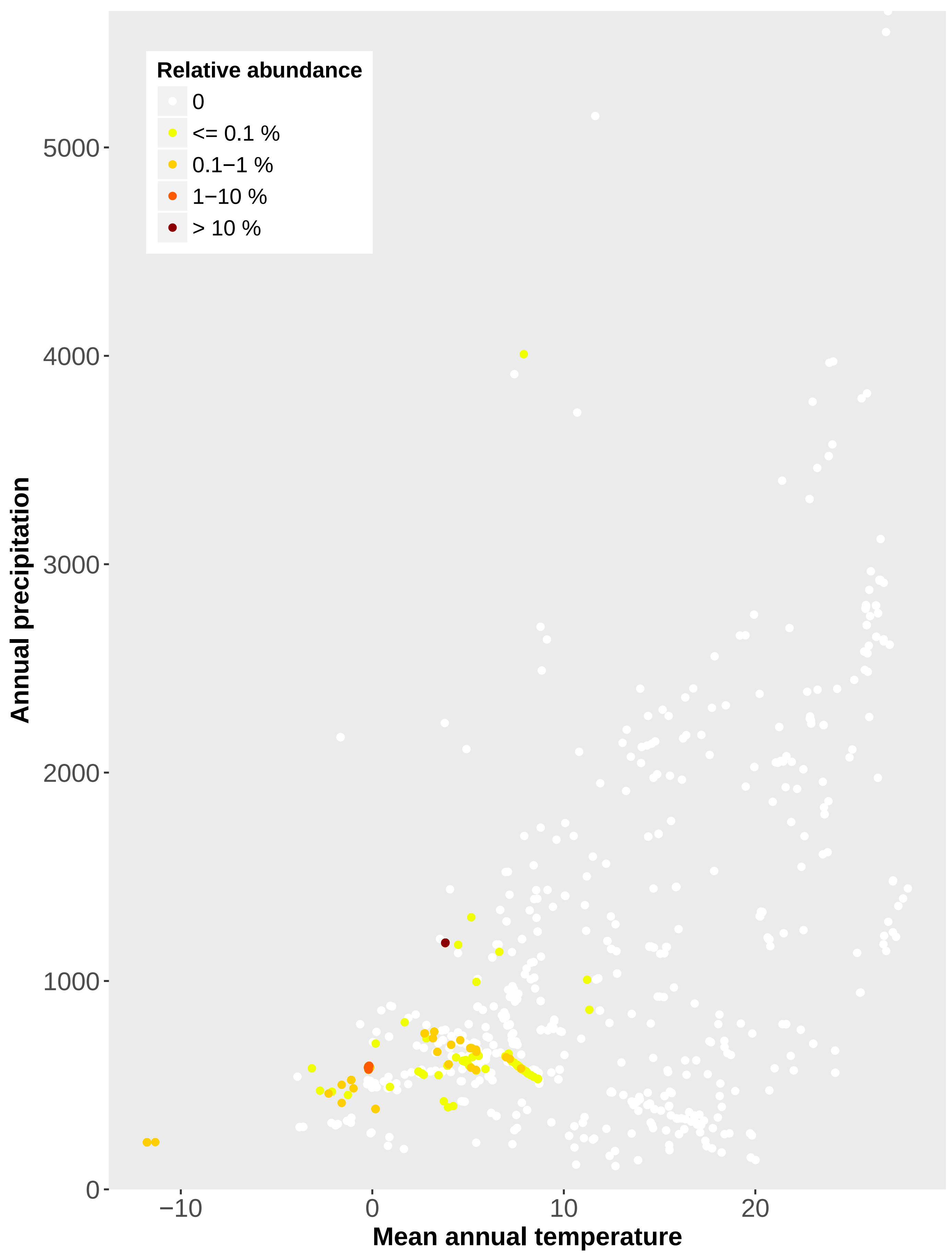

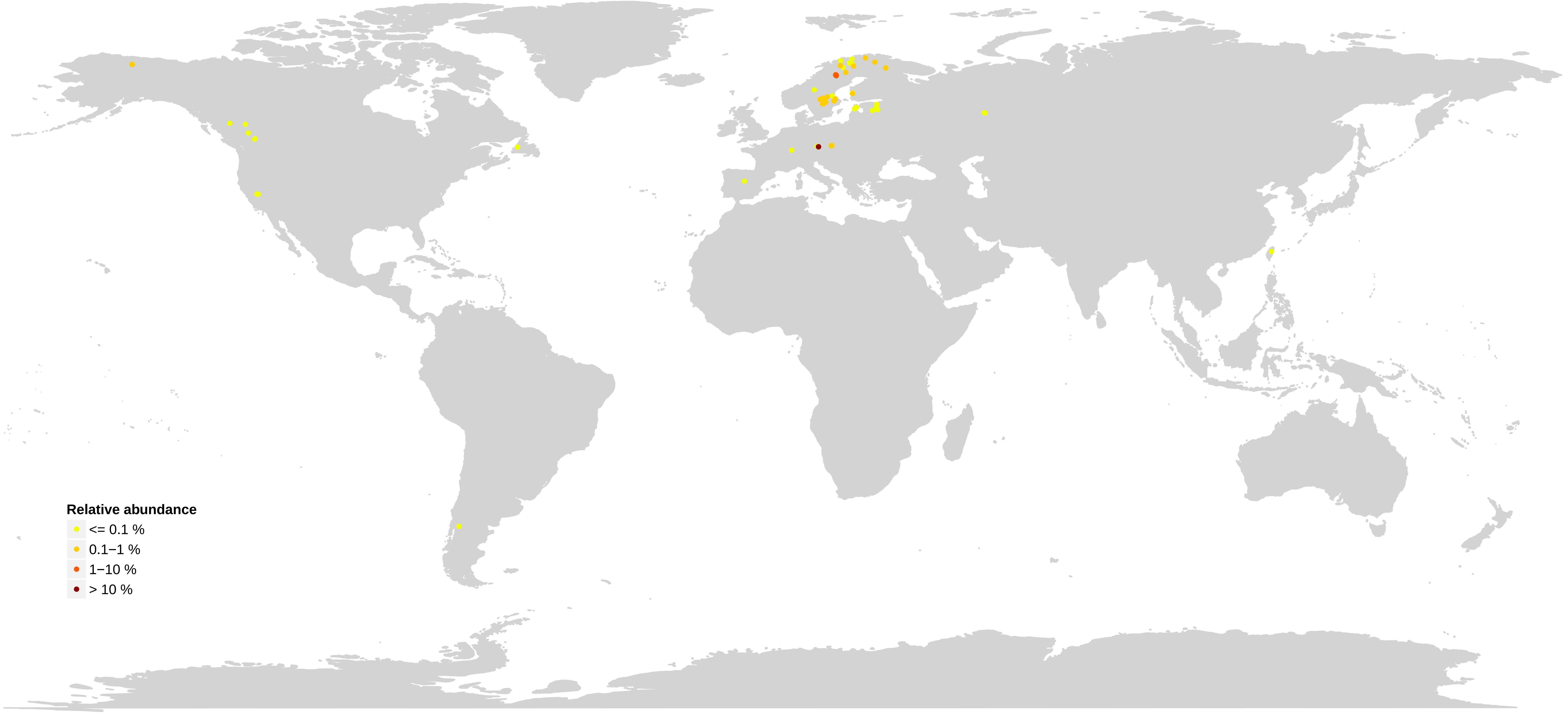

SH211242 Sordariaceae sp

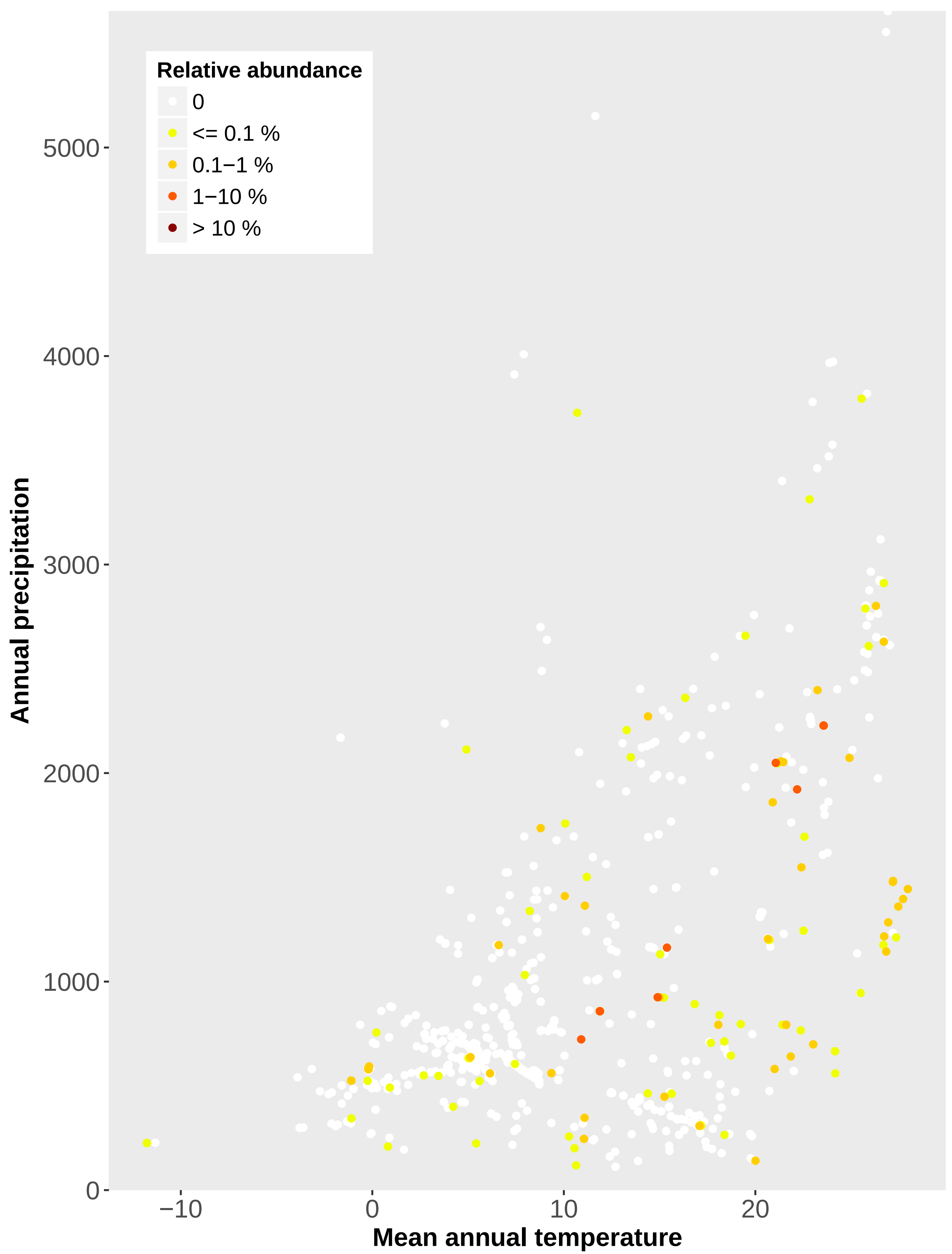

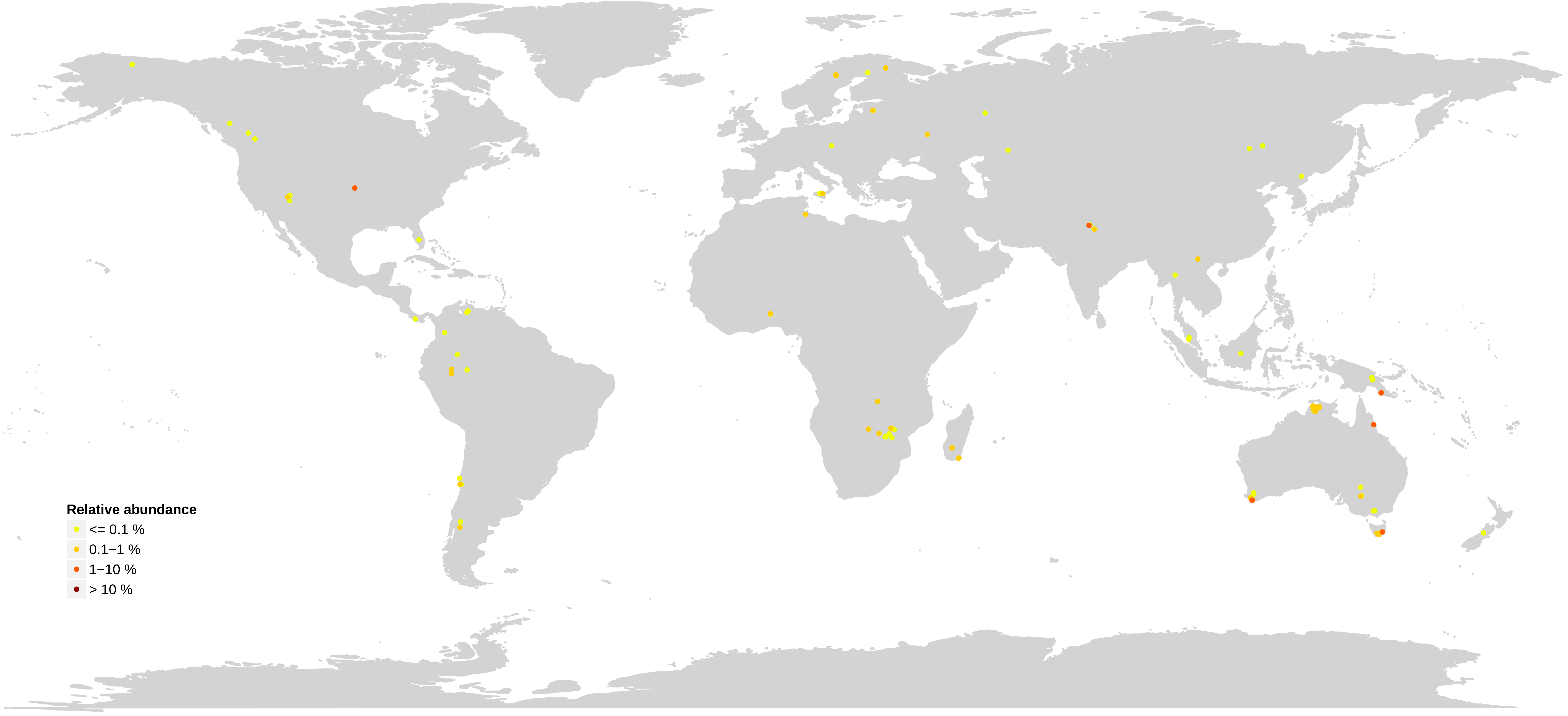

SH196641 Trichosporon dehoogii

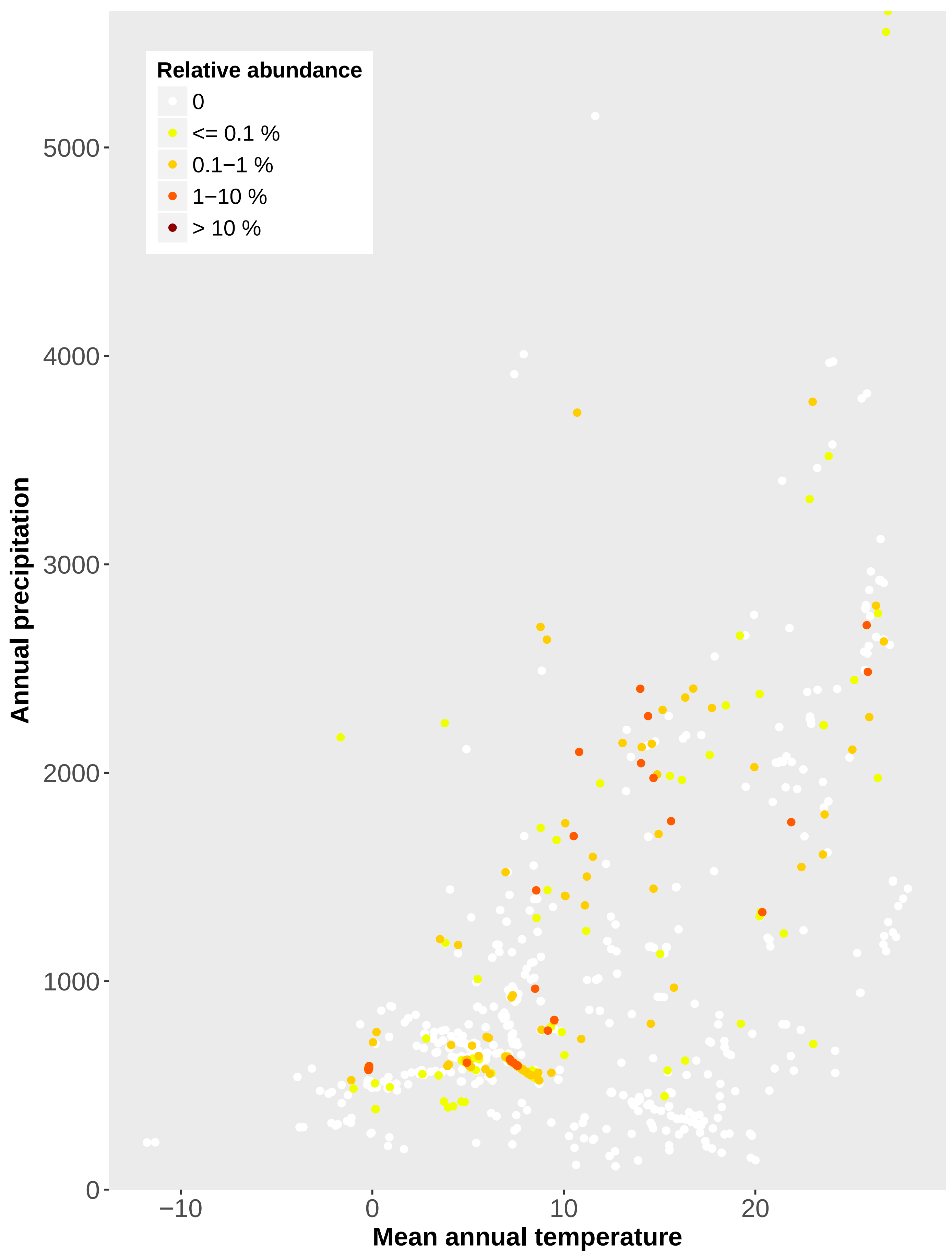

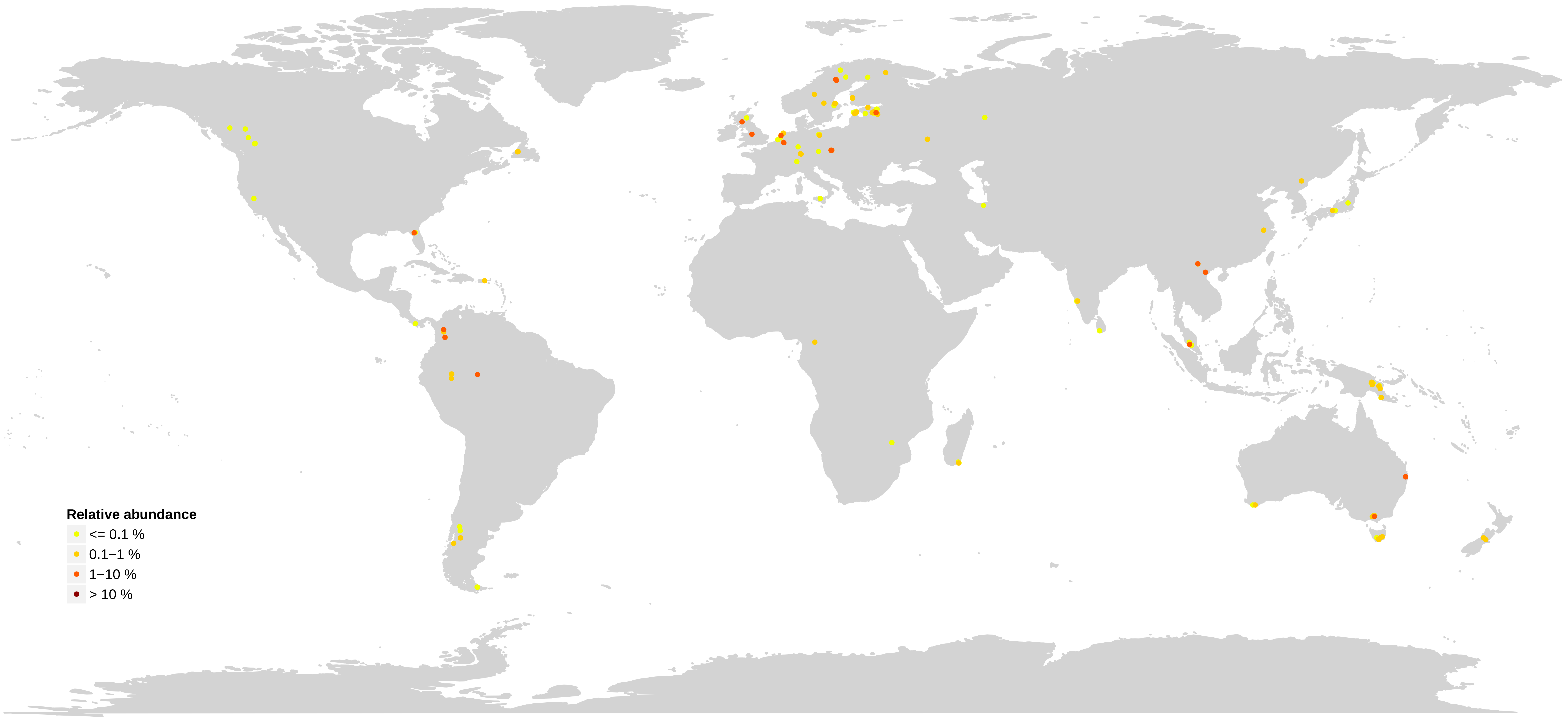

SH198996 Pyrenochaetopsis leptospora

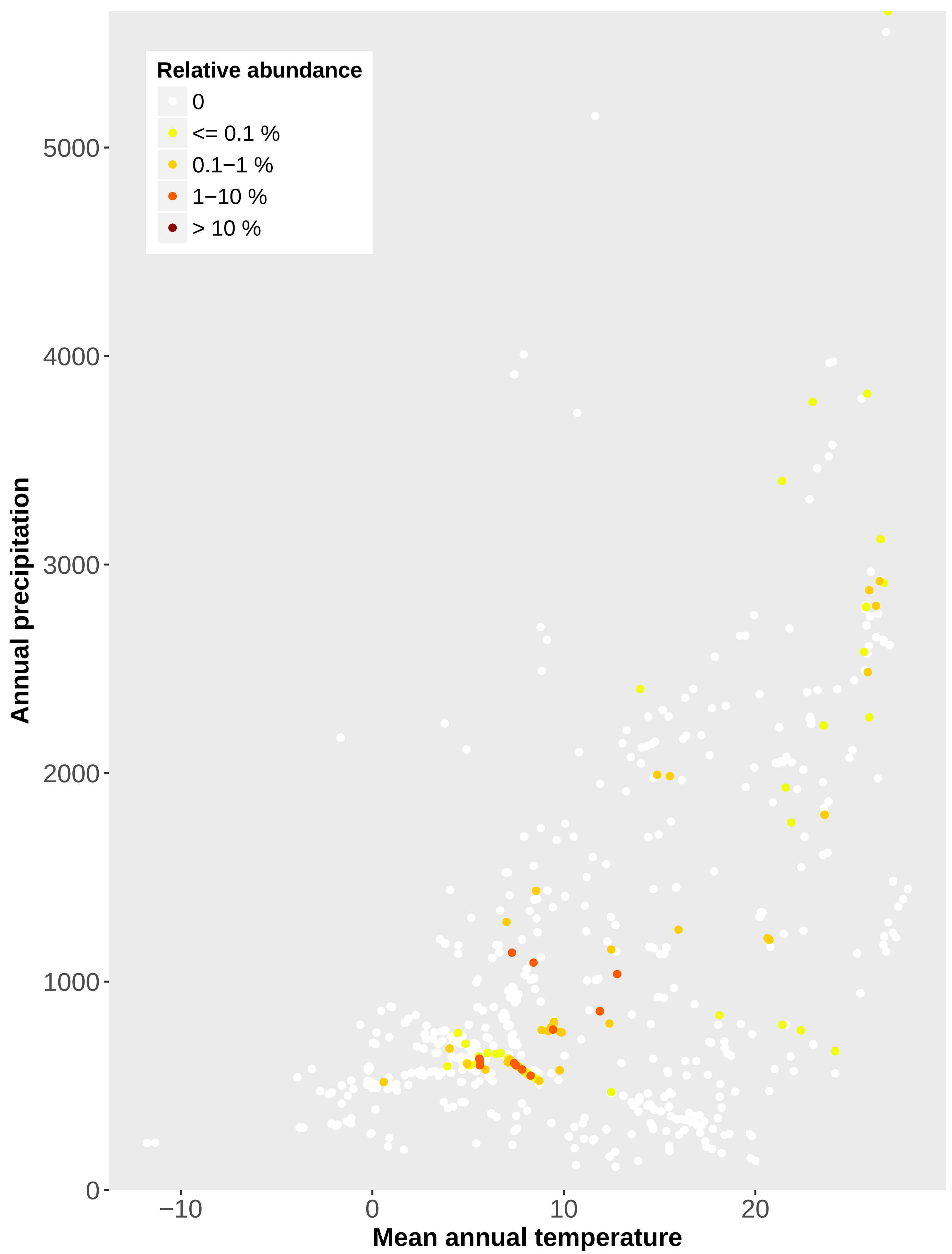

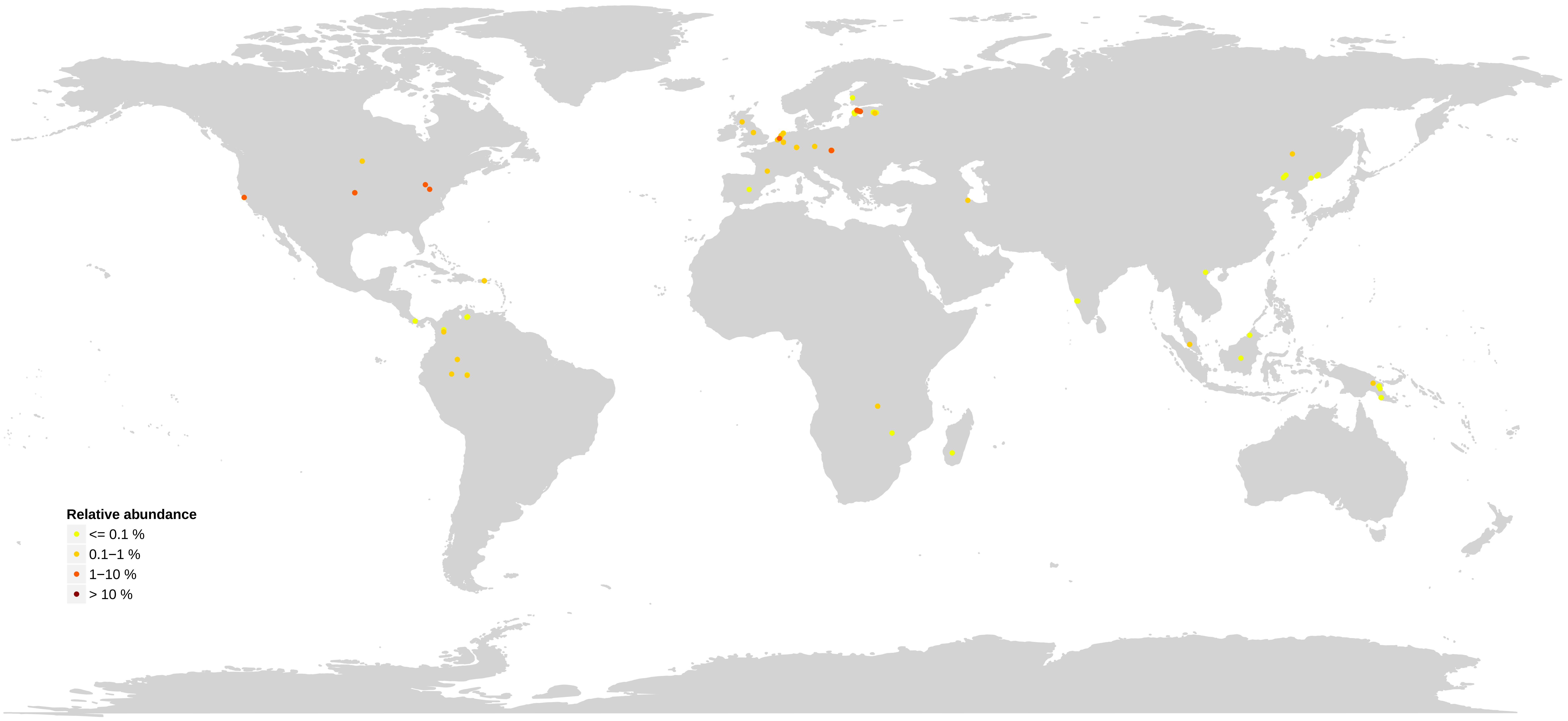

SH211298 Tetracladium sp

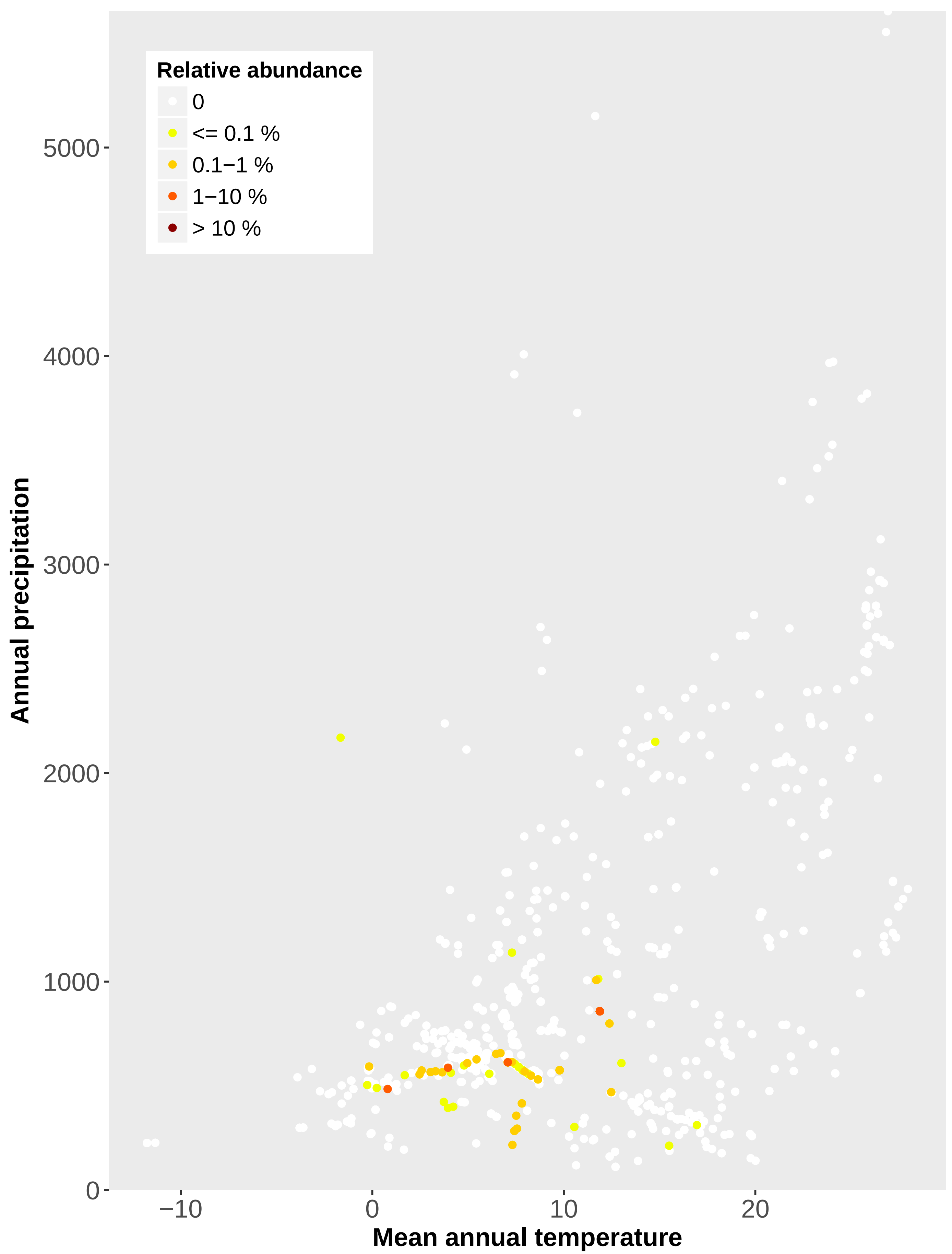

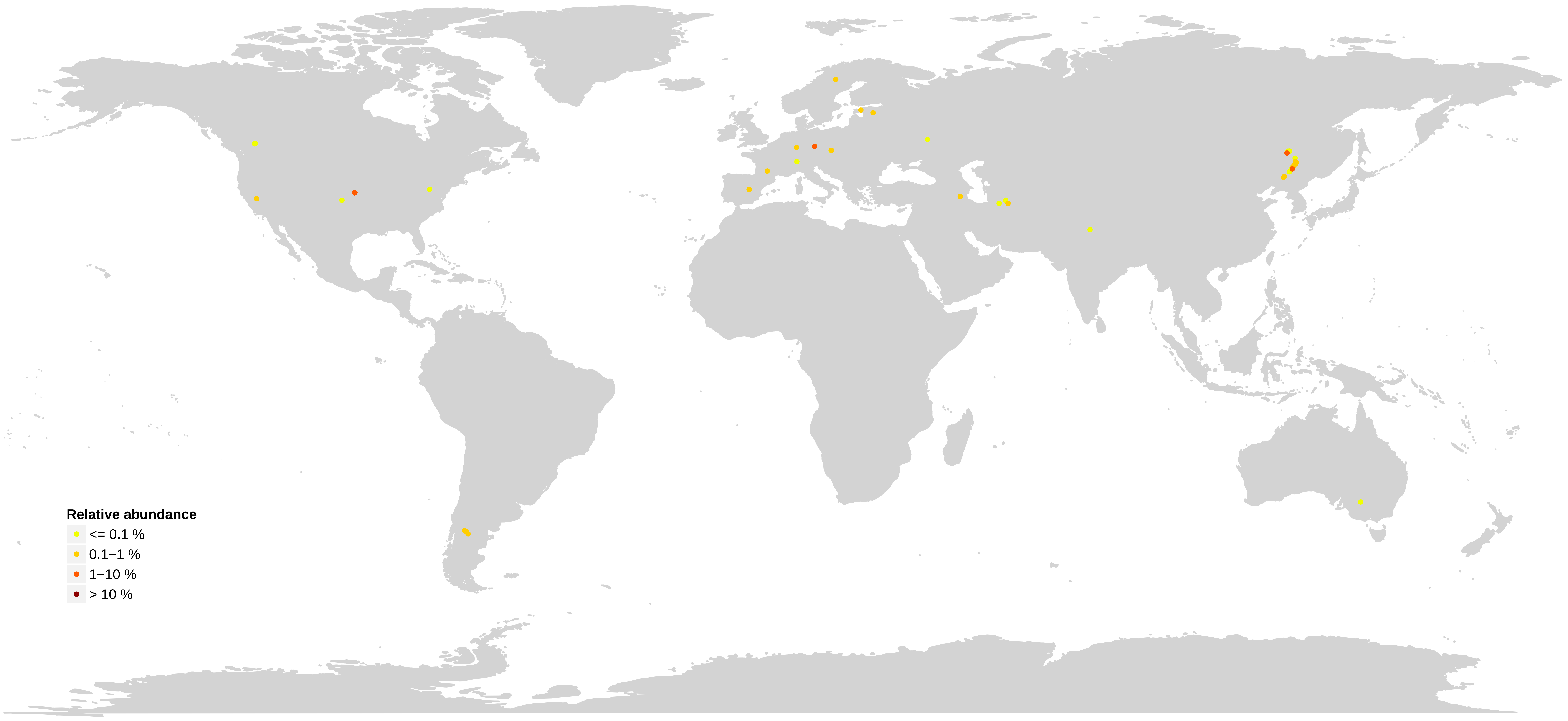

SH182794 Ilyonectria macrodidyma

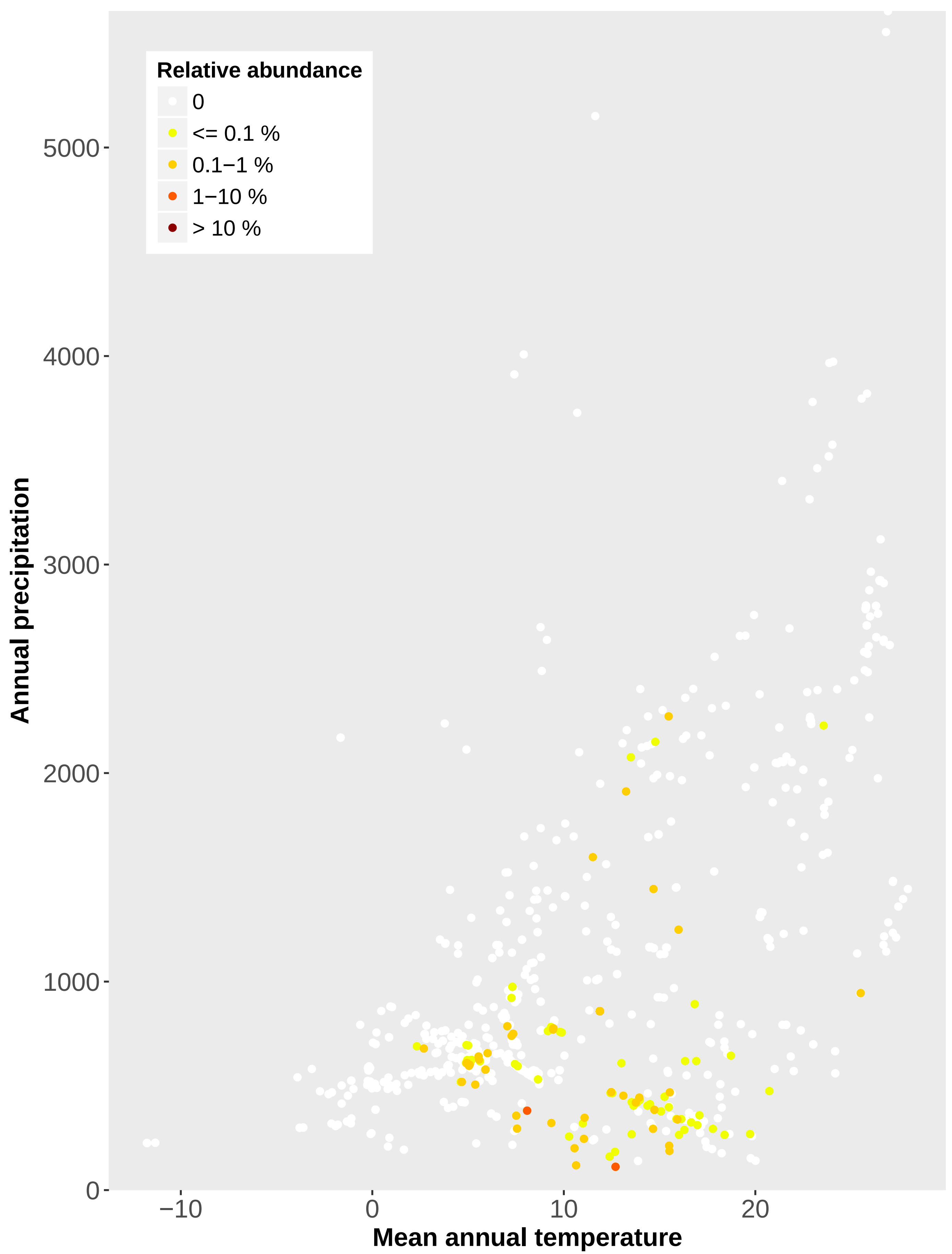

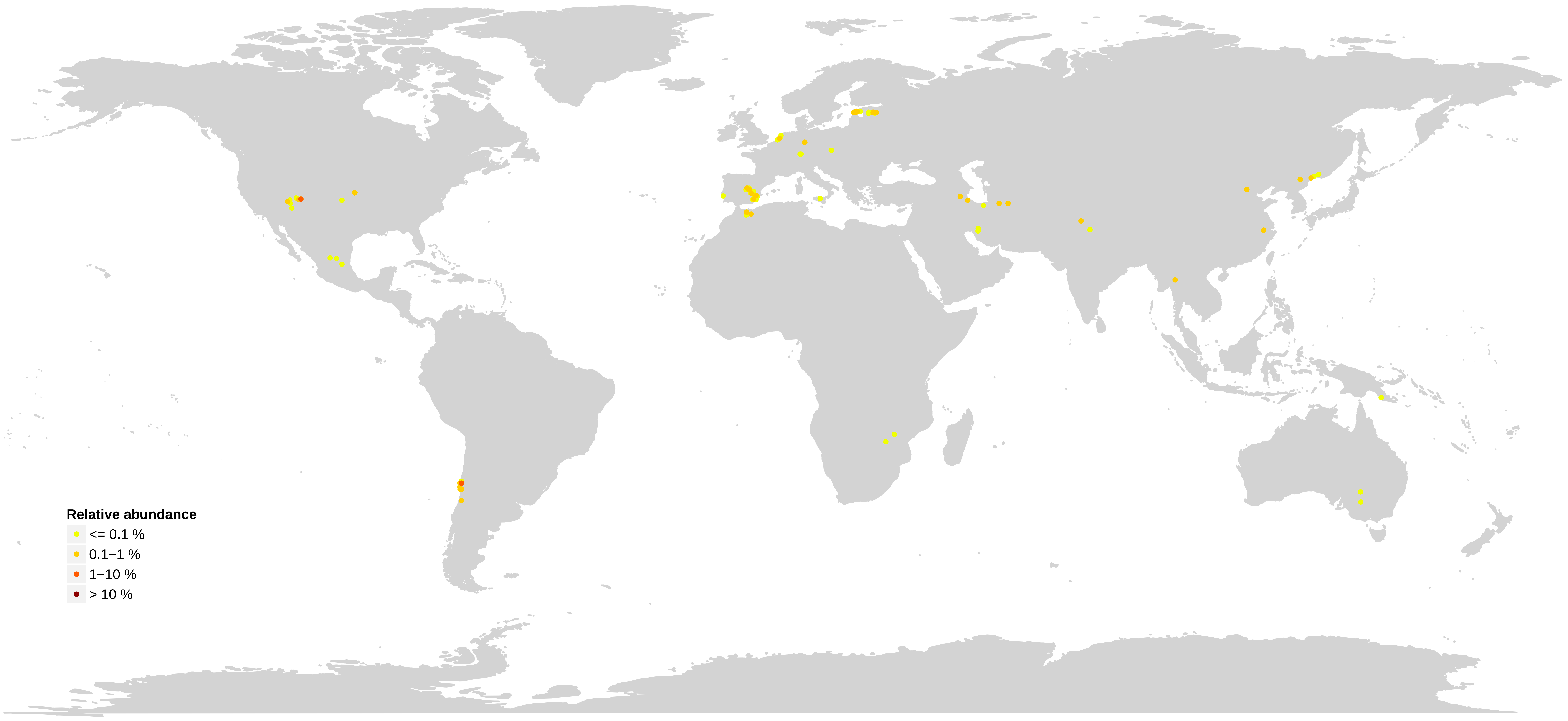

SH179955 Luellia sp

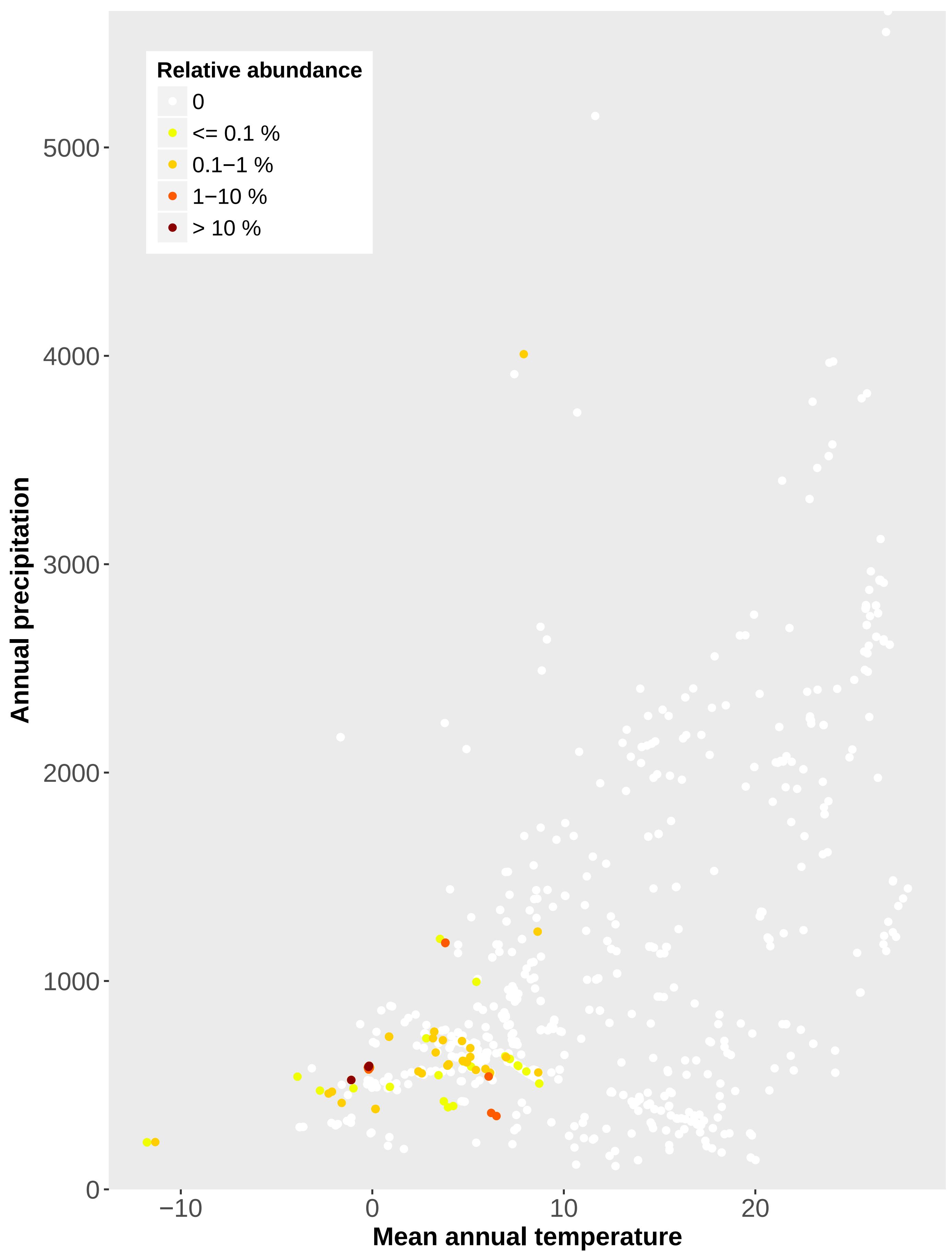

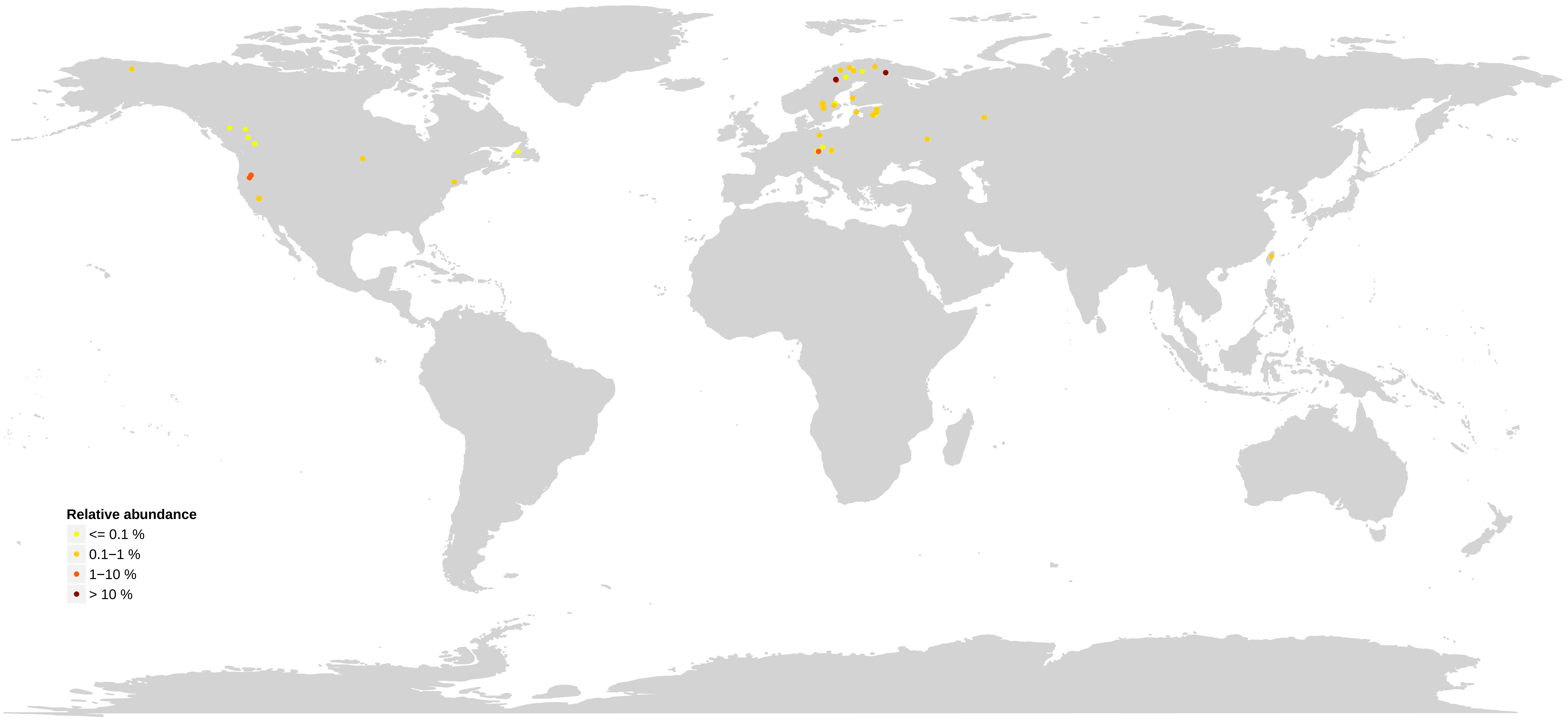

SH208302 Geminibasidium sp

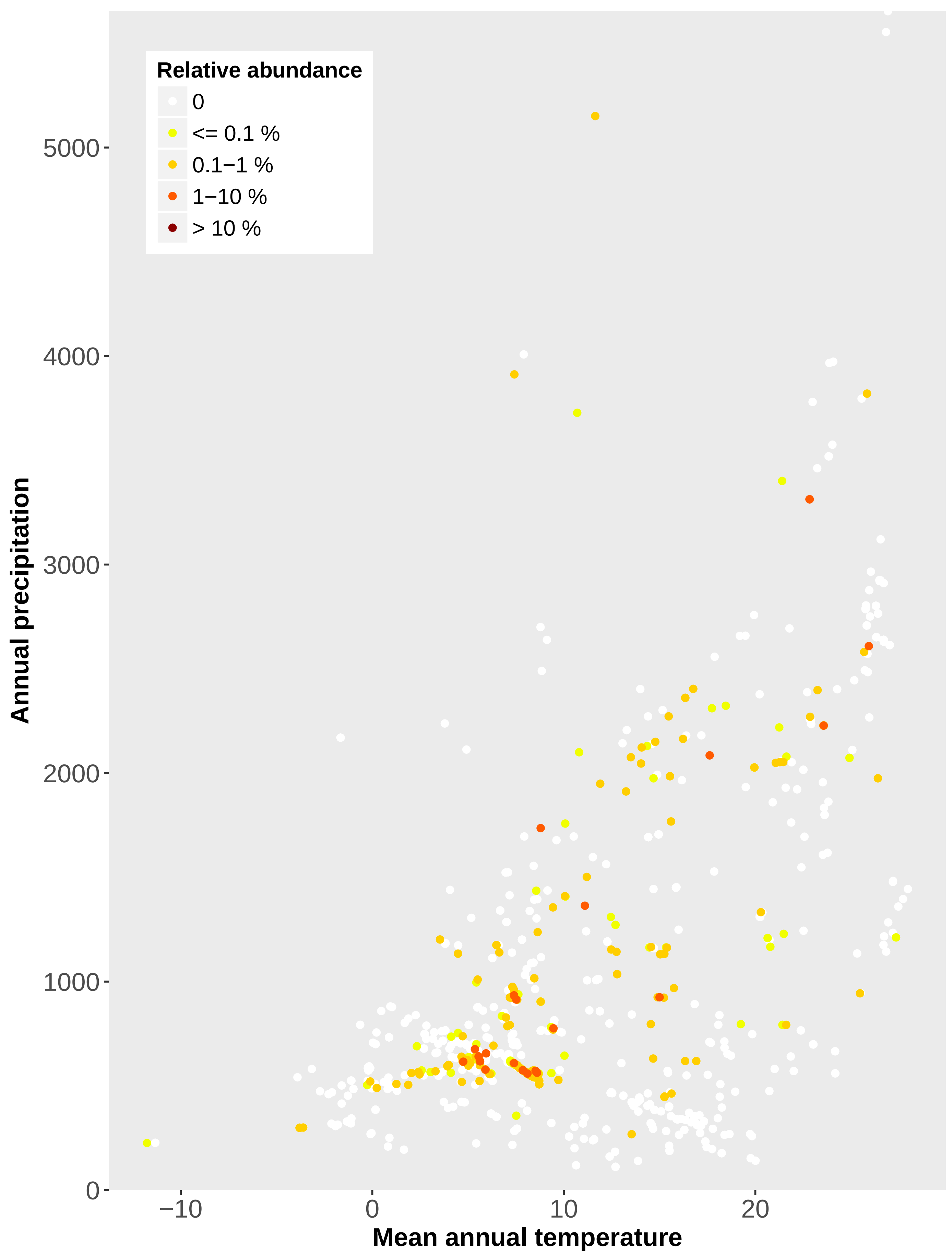

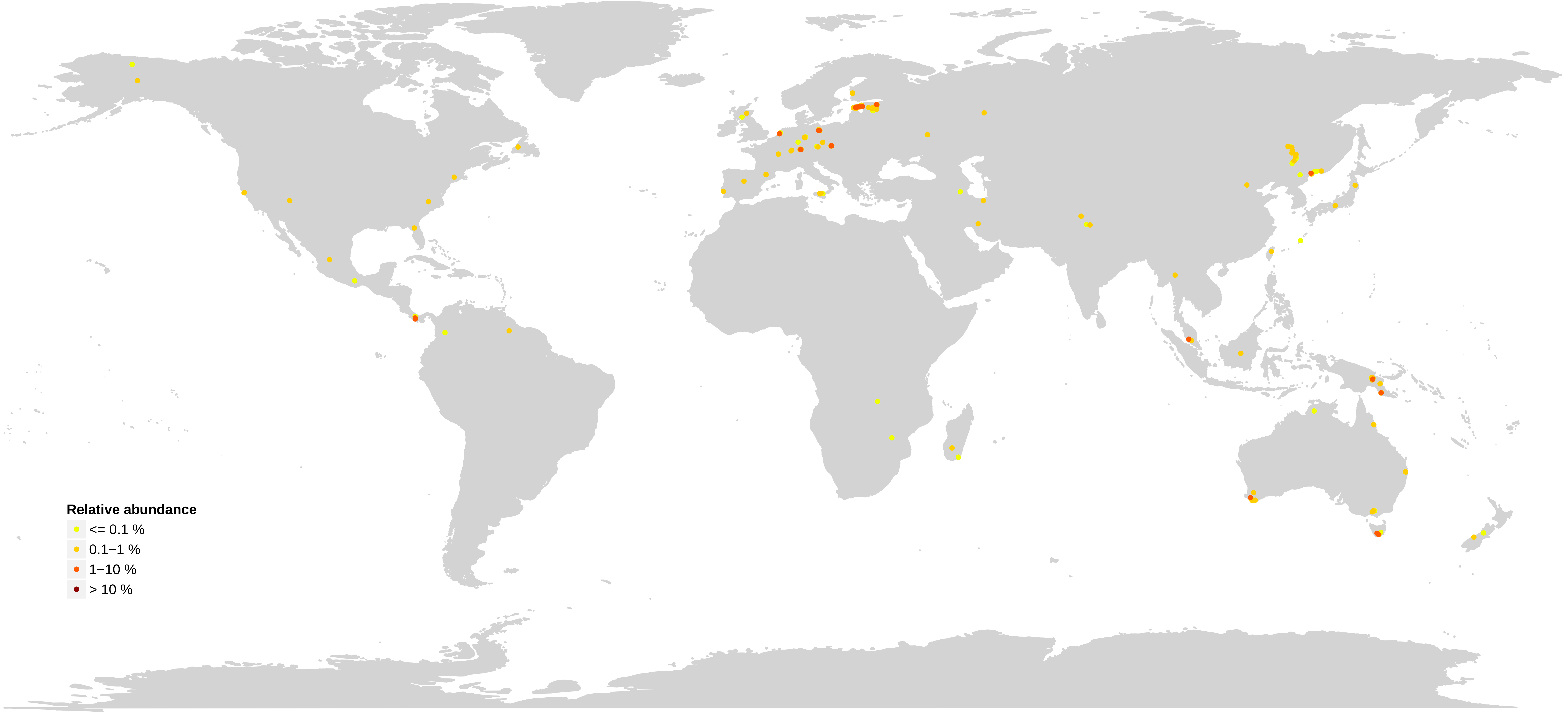

SH215677 Byssonectria fusispora

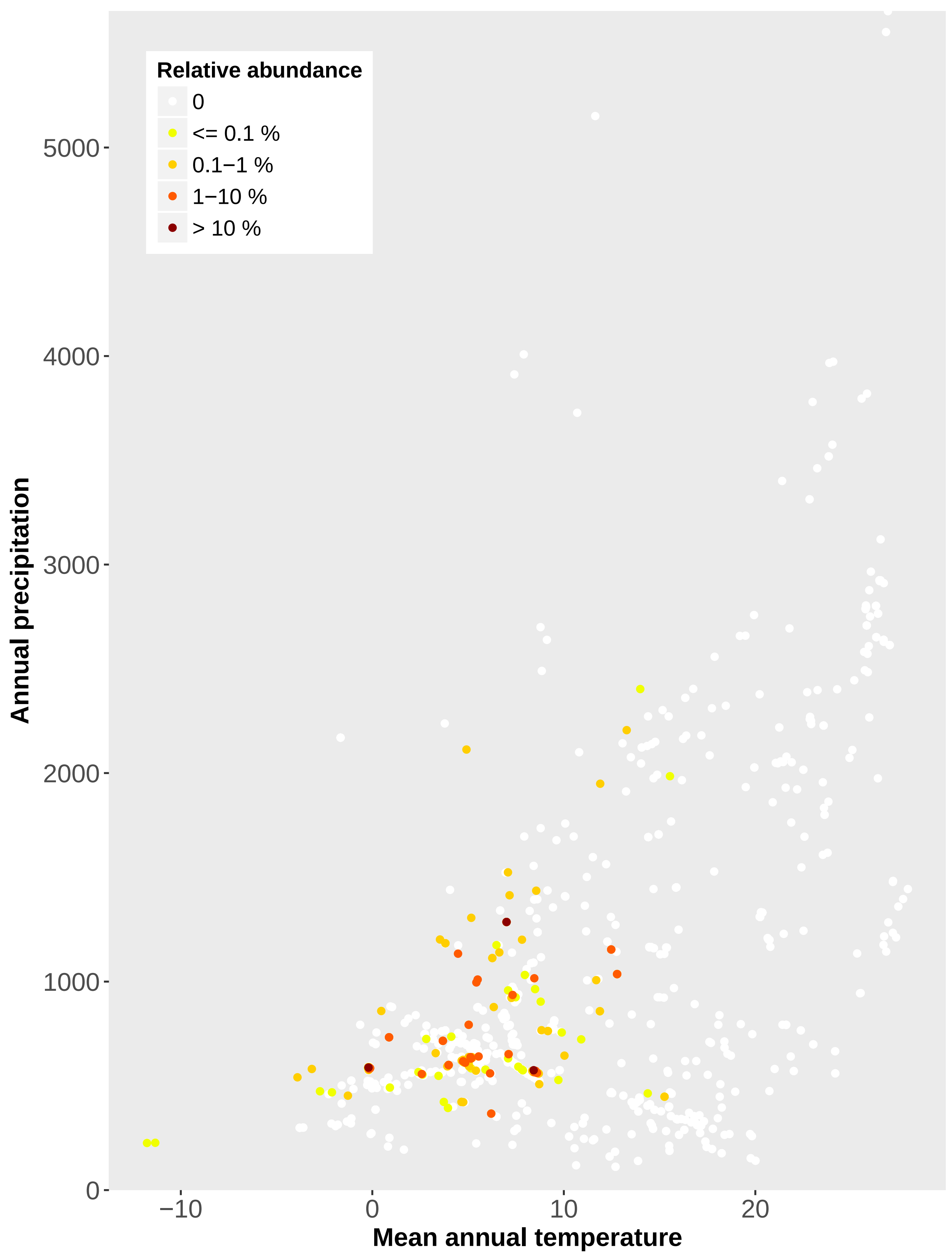

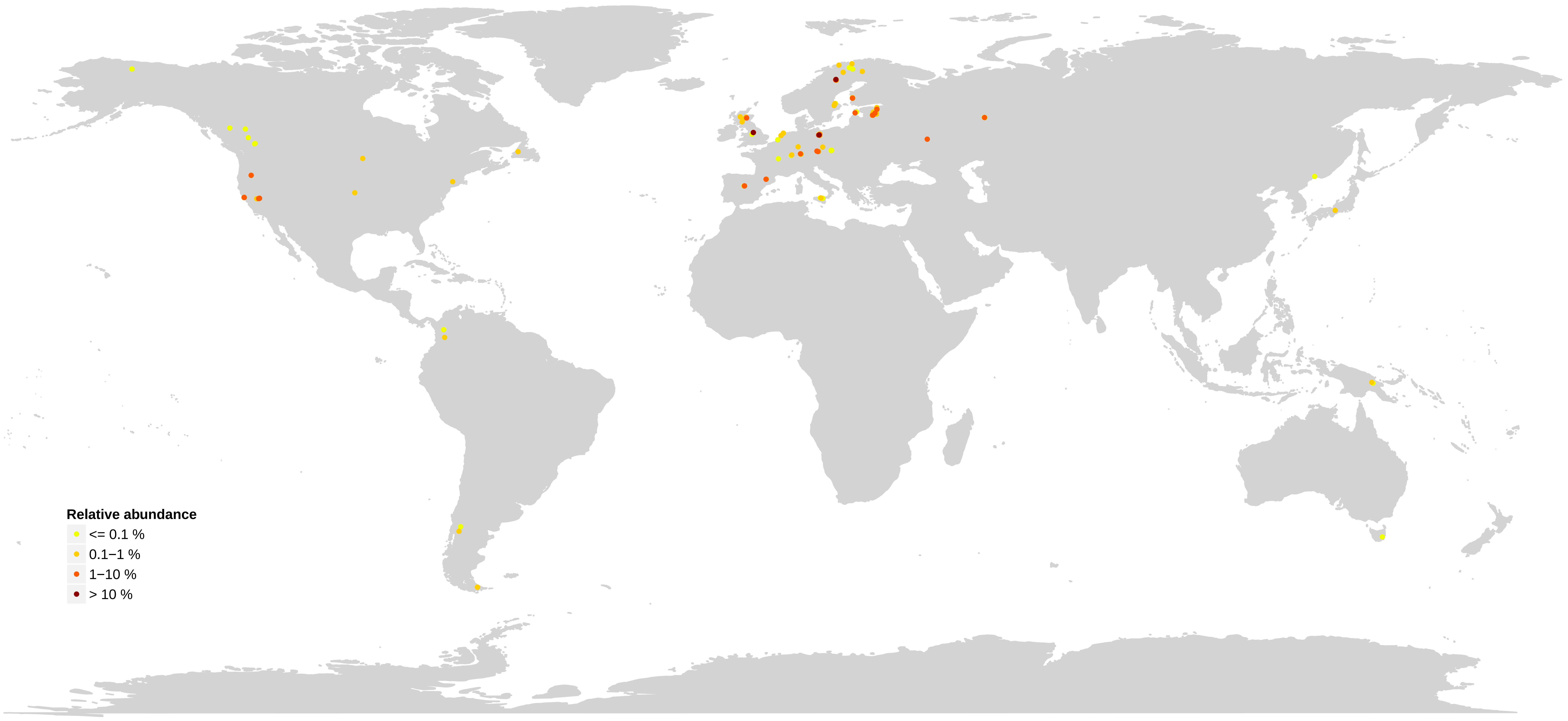

SH180111 Mortierella exigua

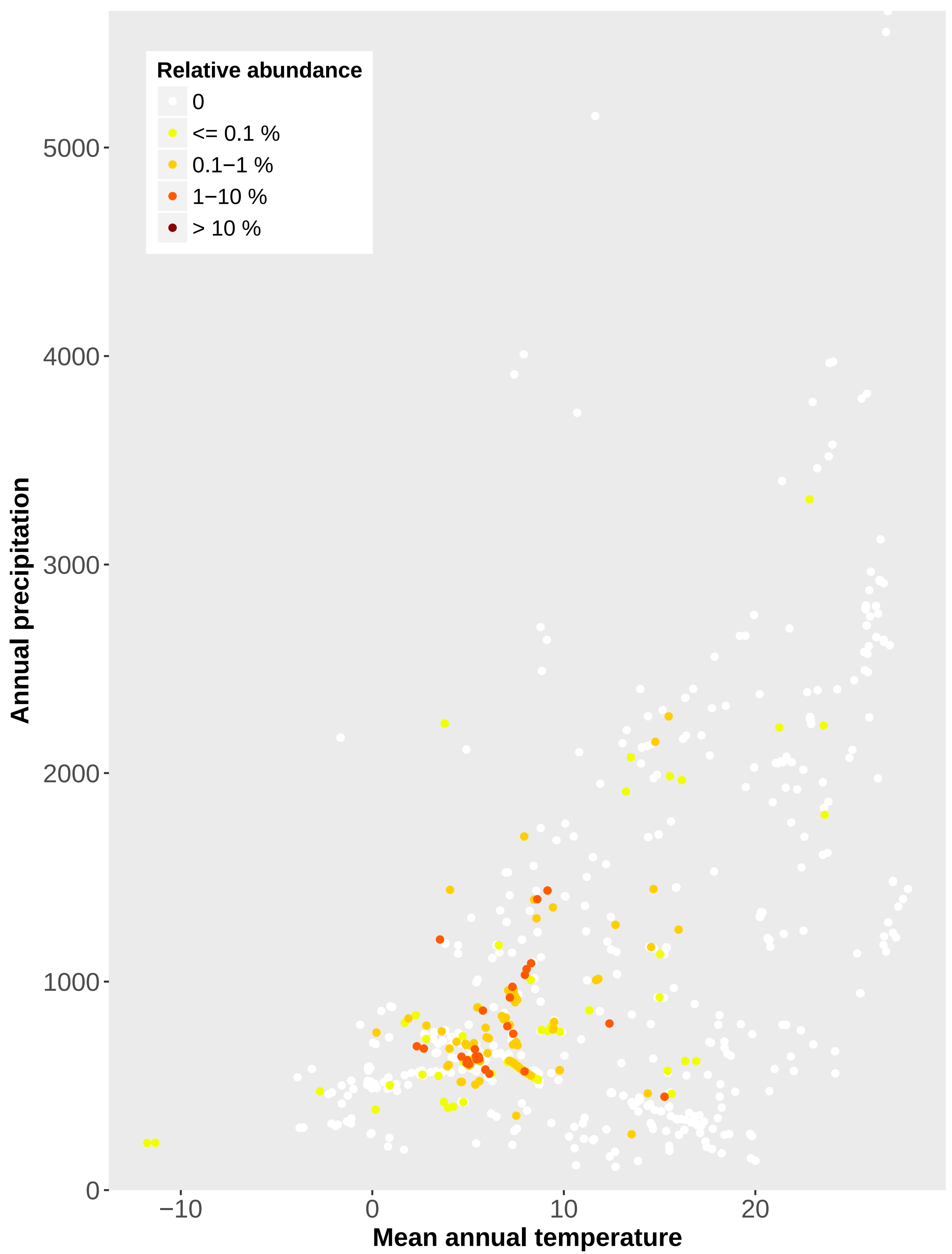

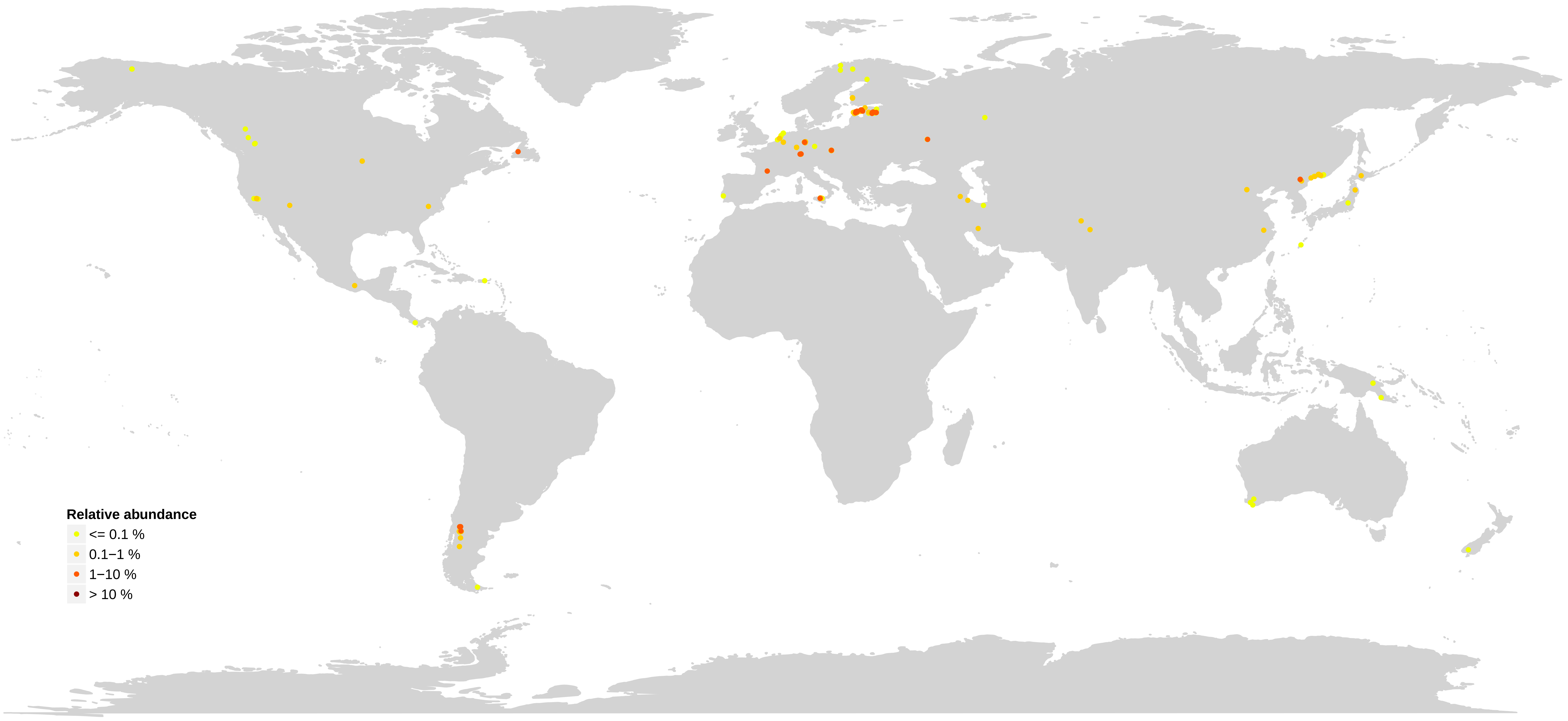

SH219610 Venturia sp

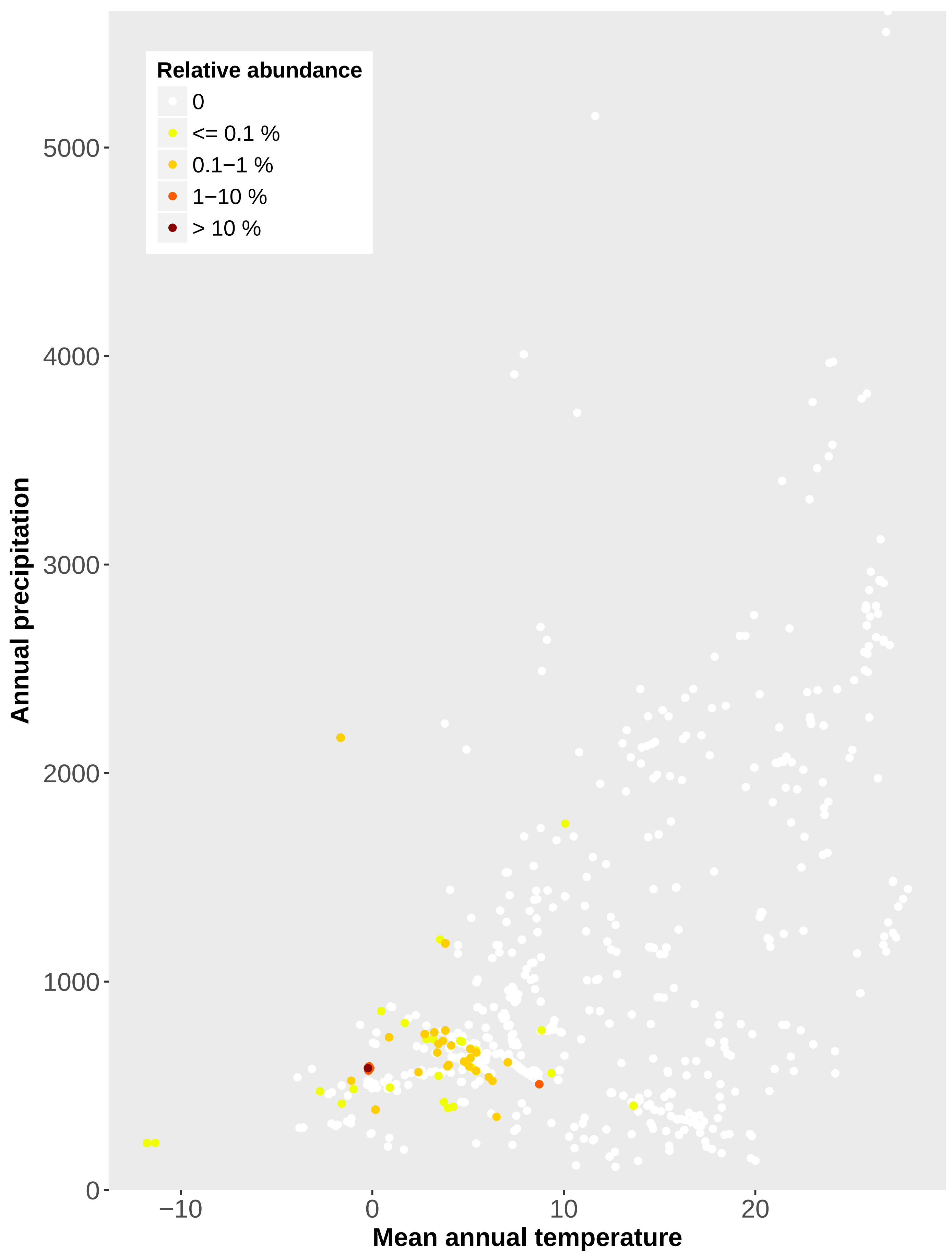

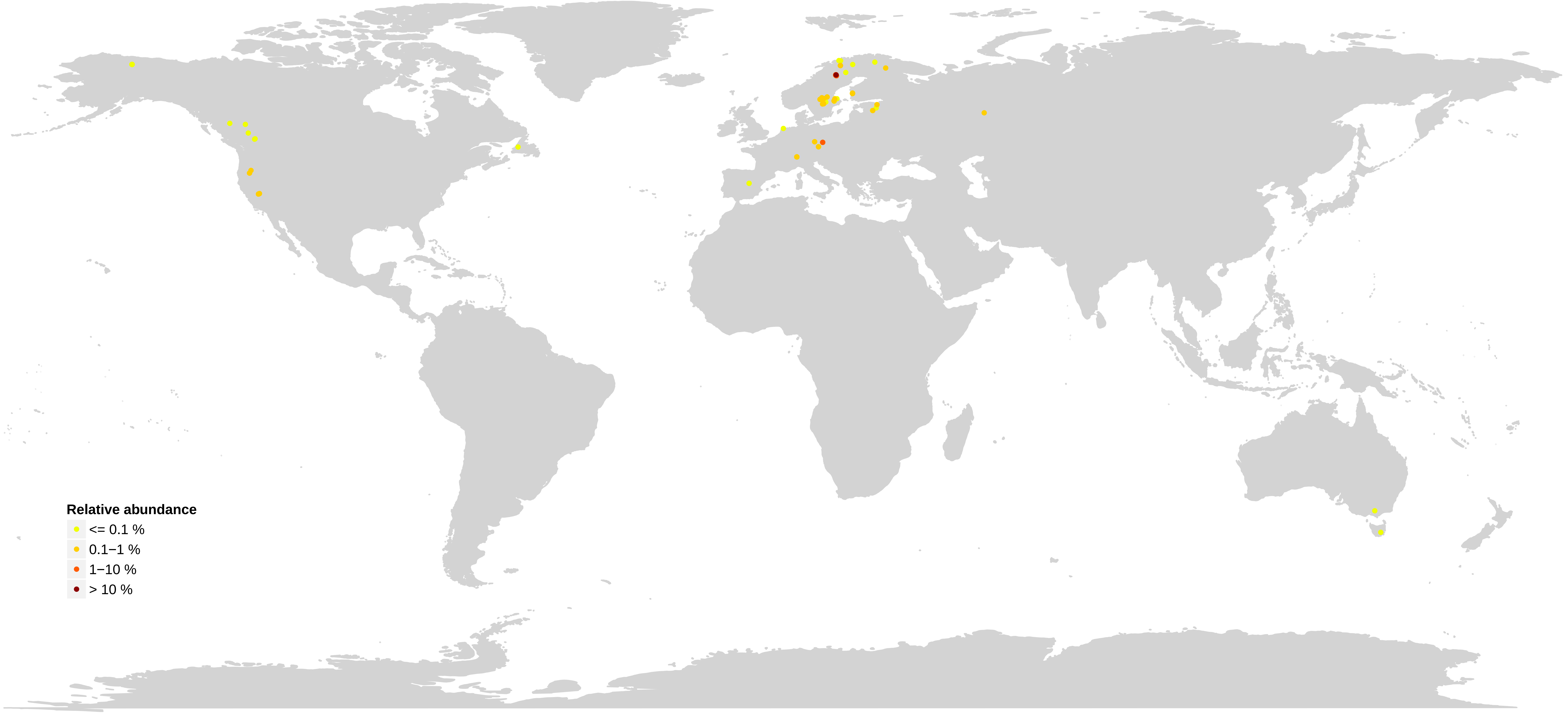

SH181085 Meliniomyces vraolstadiae

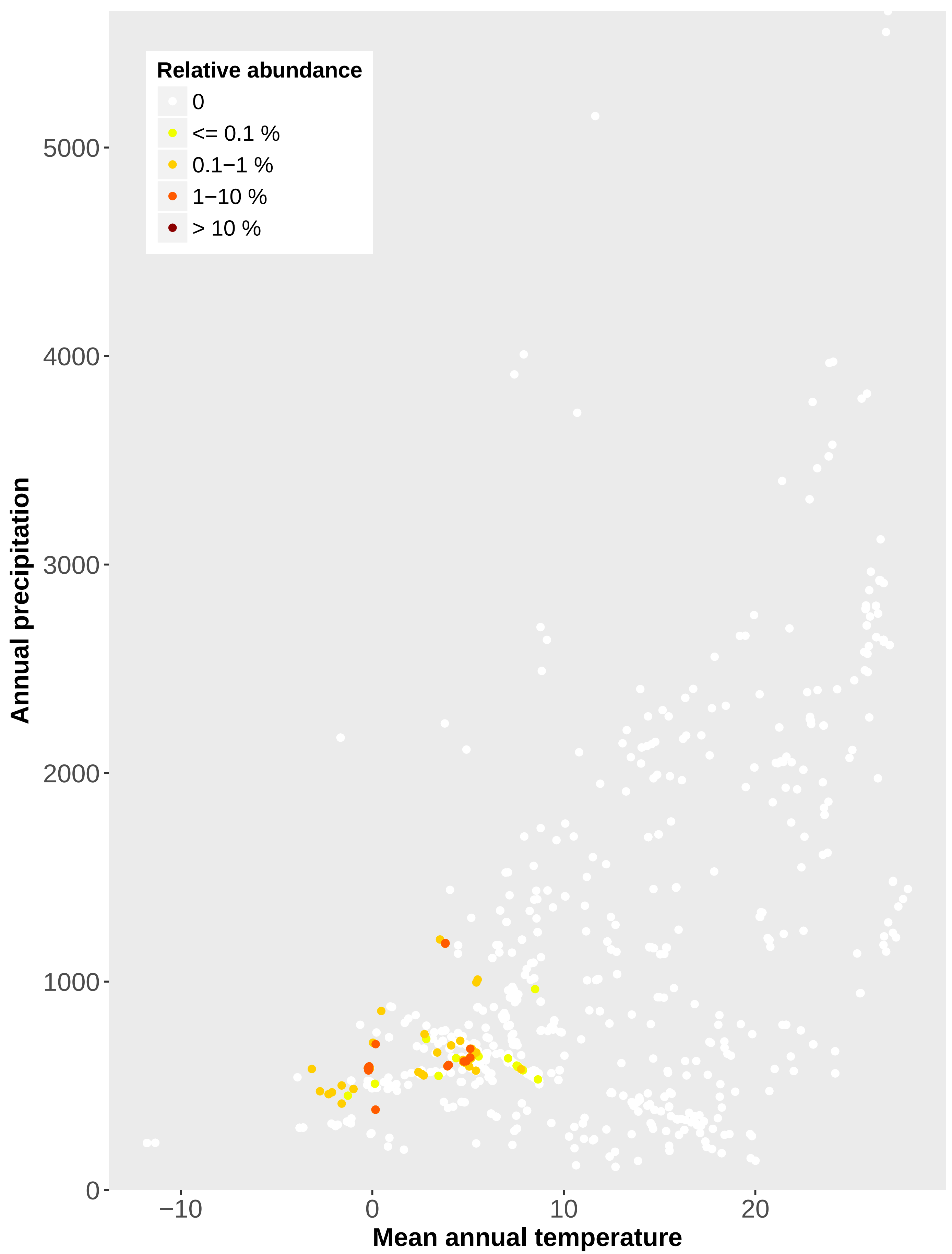

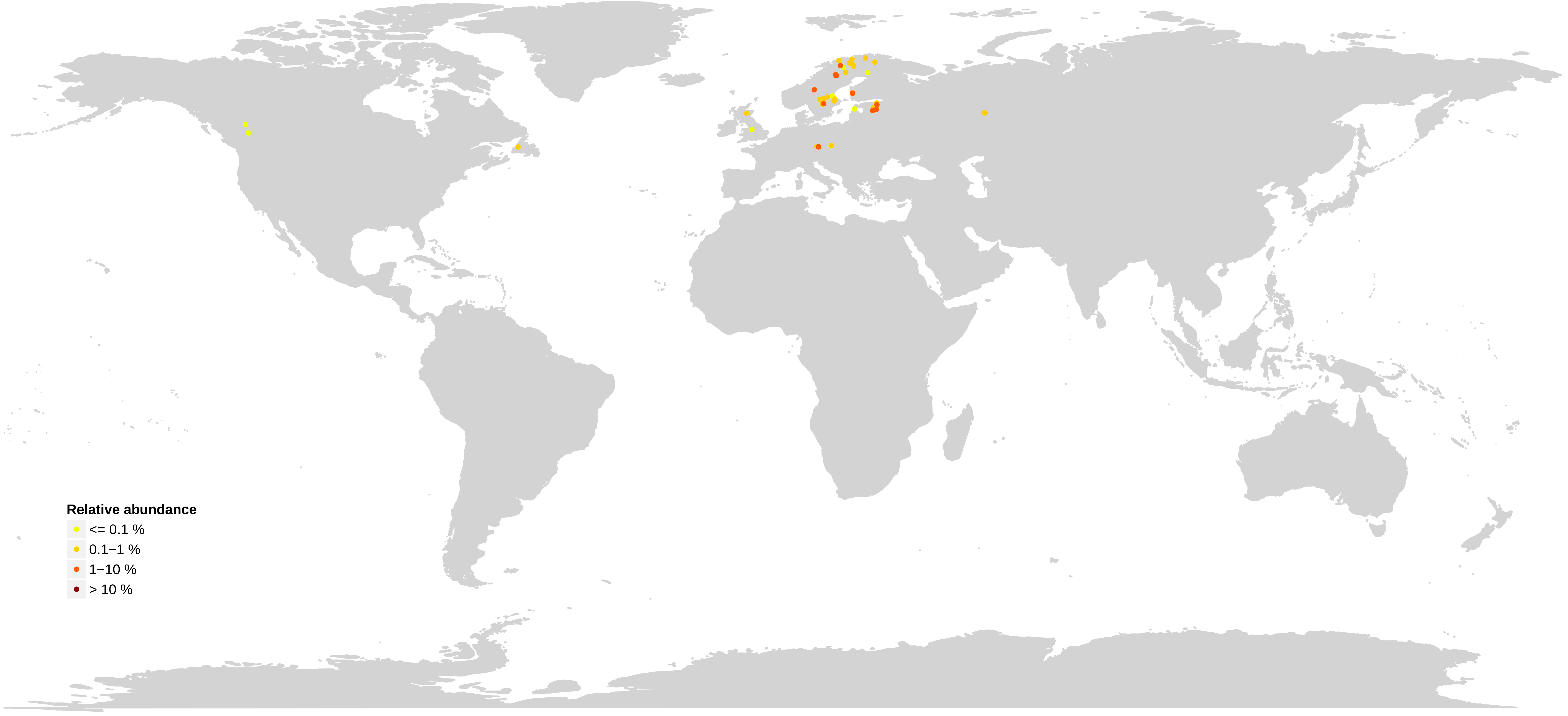

SH194252 Helotiales sp

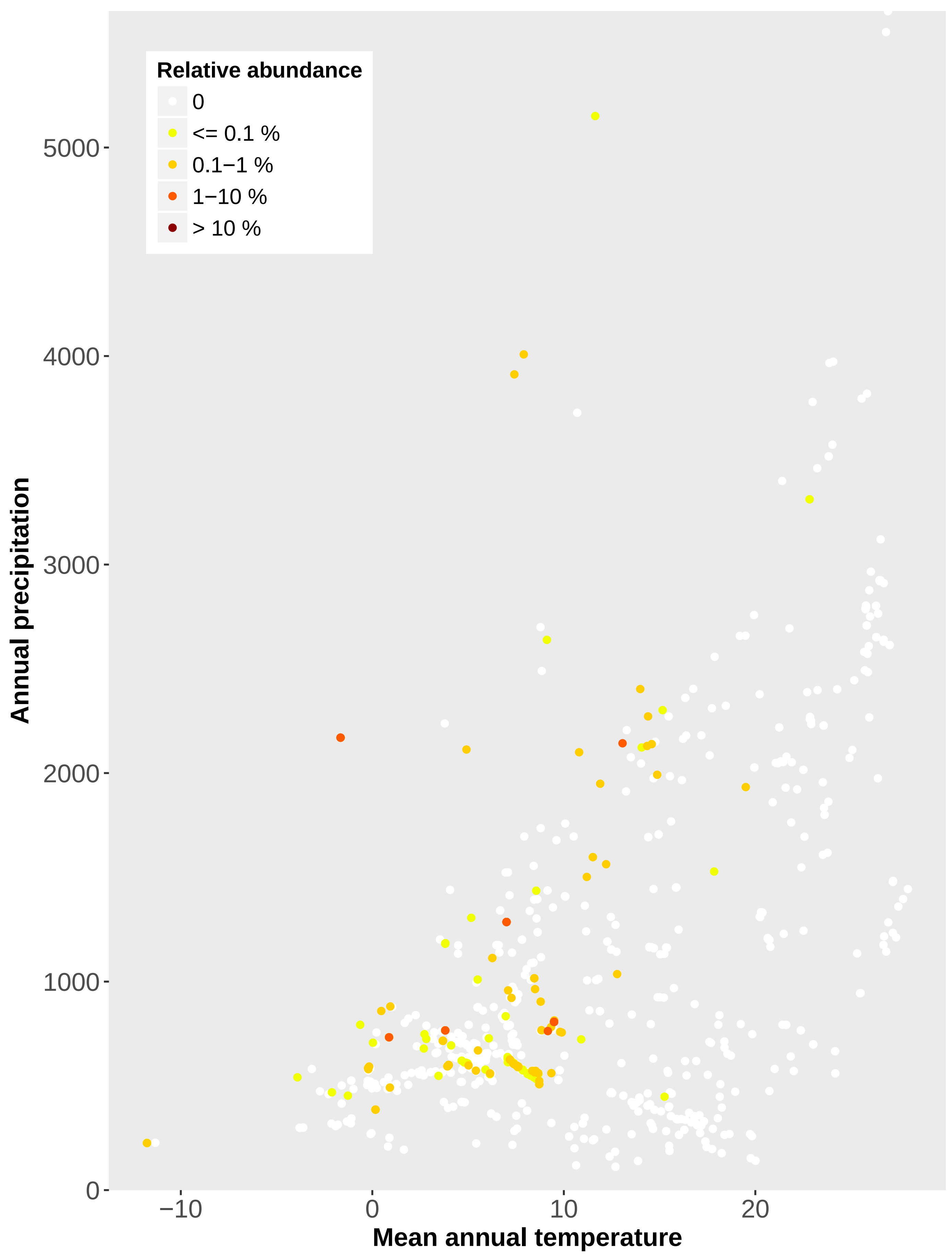

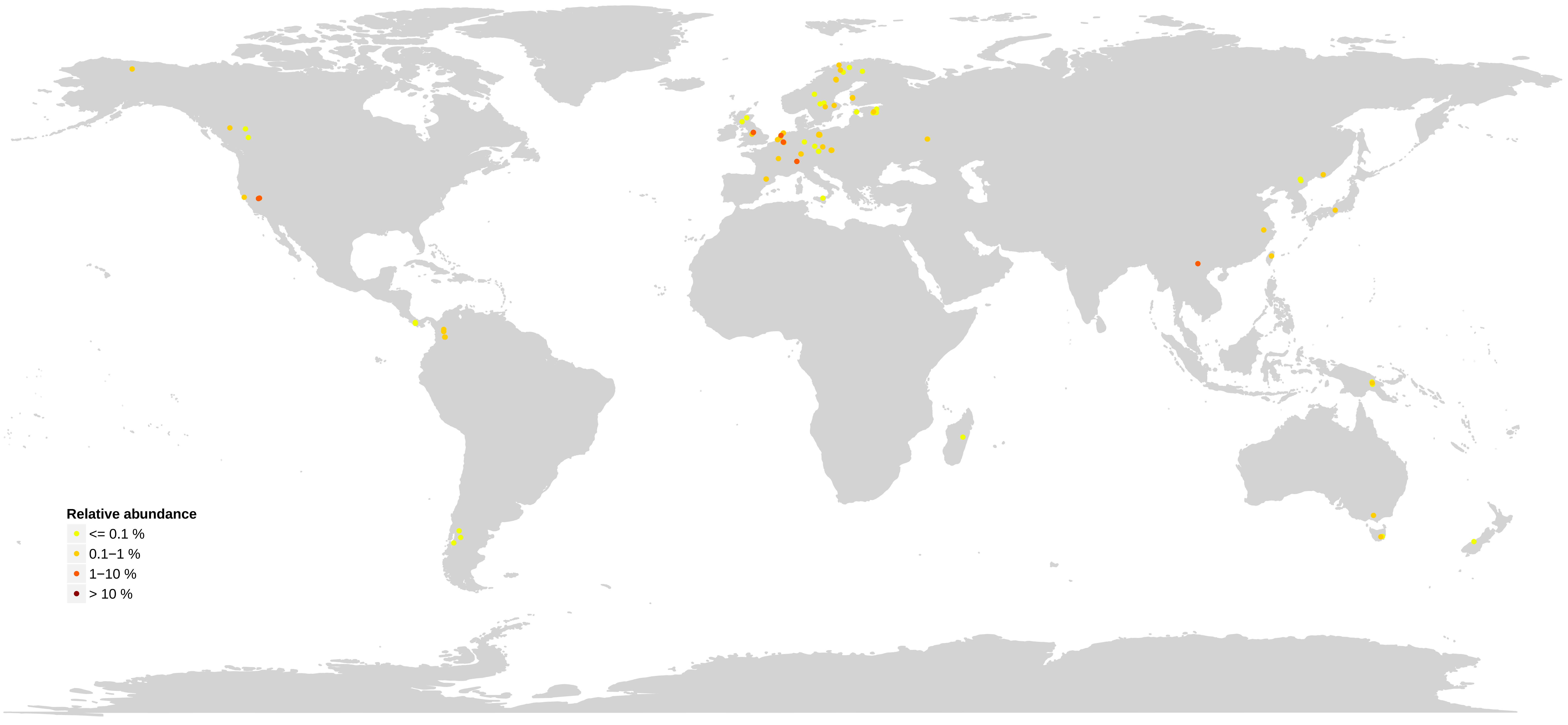

SH202710 Ascomycota sp

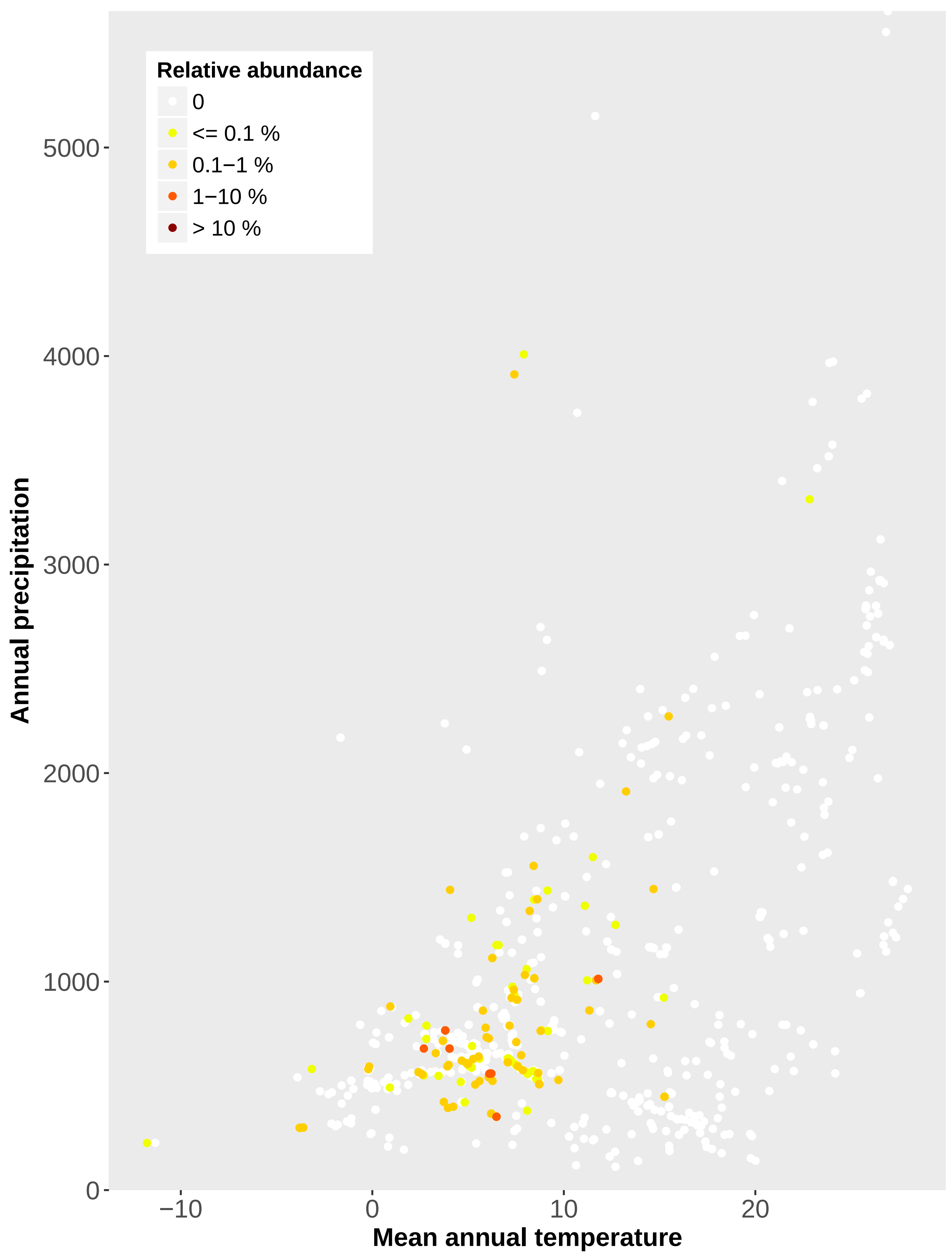

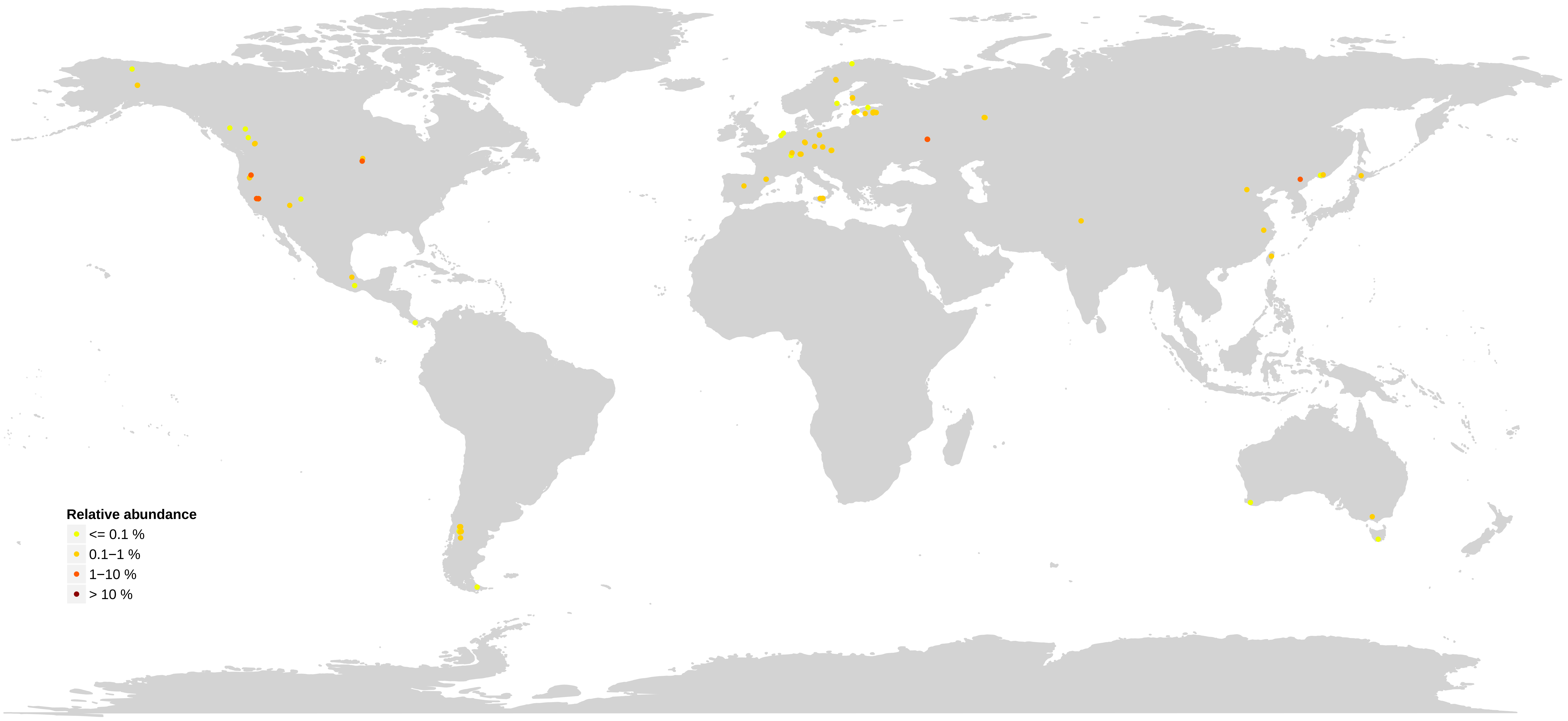

SH209754 Sordariales sp

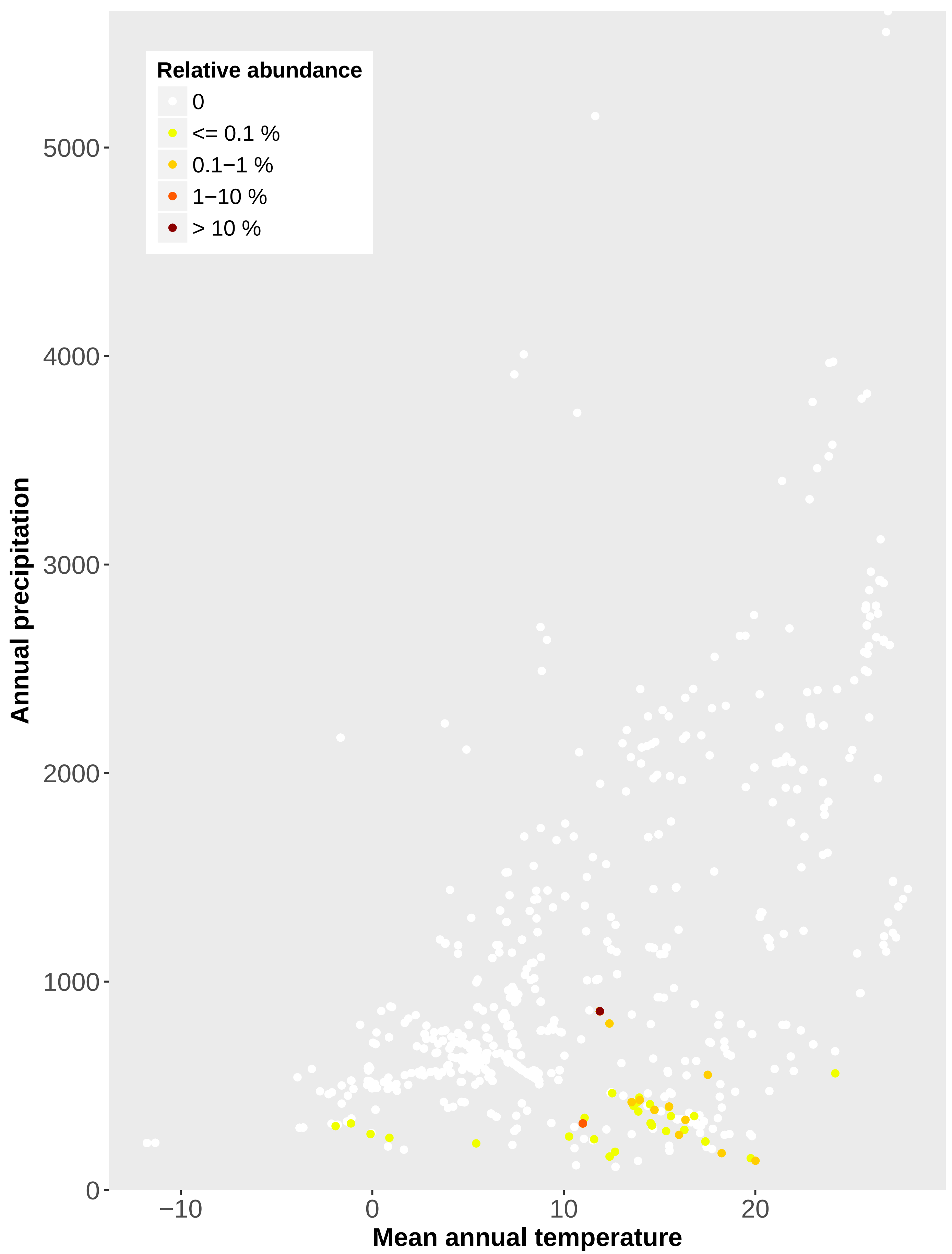

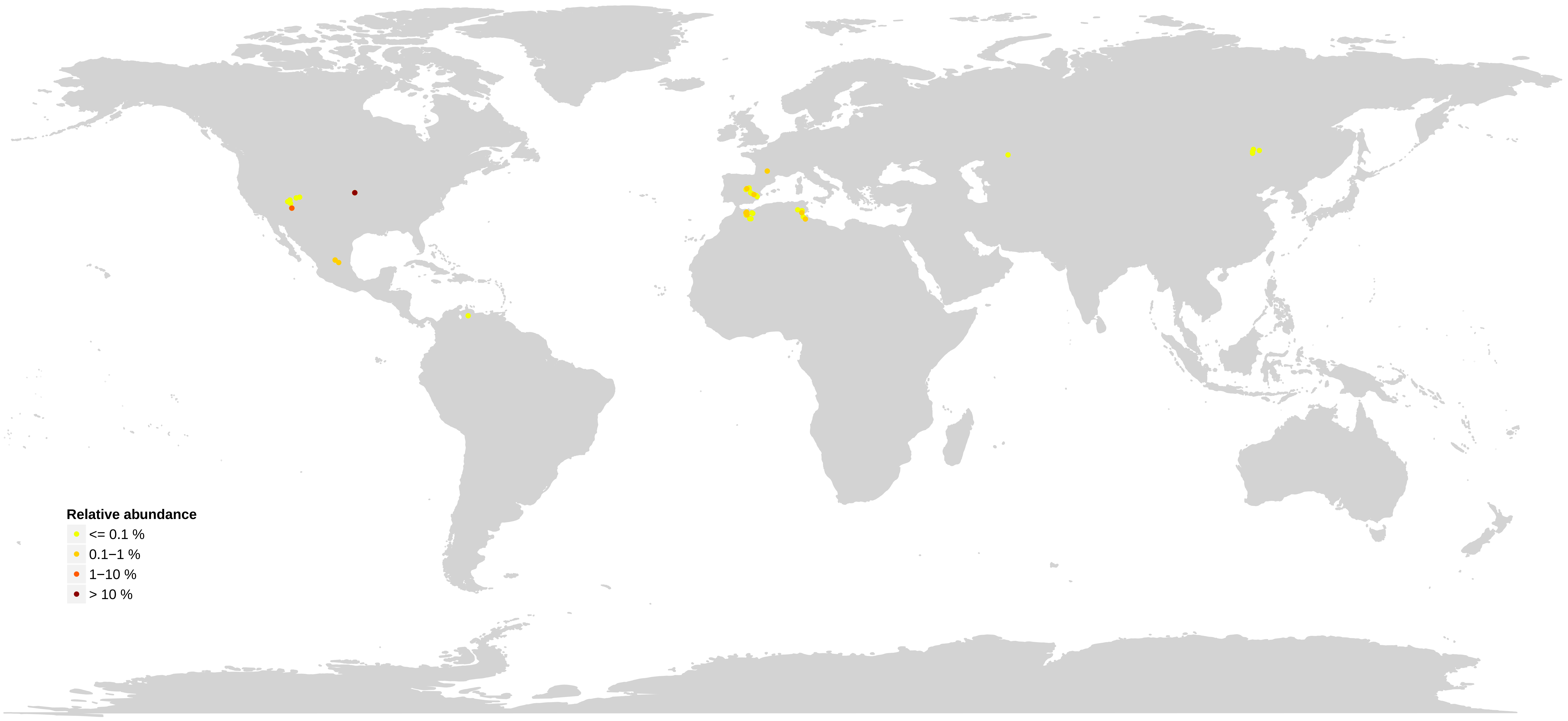

SH217841 Herpotrichiellaceae sp

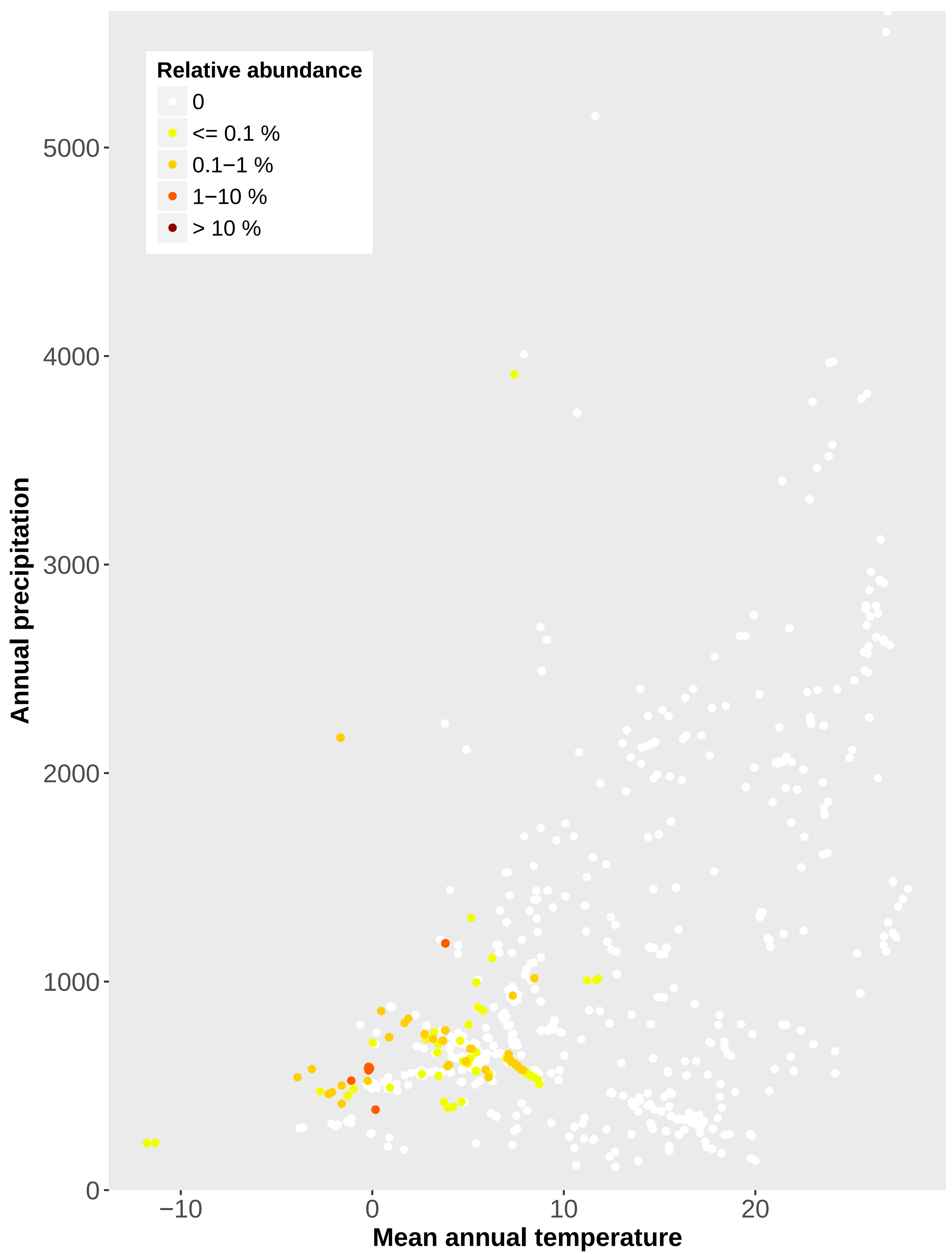

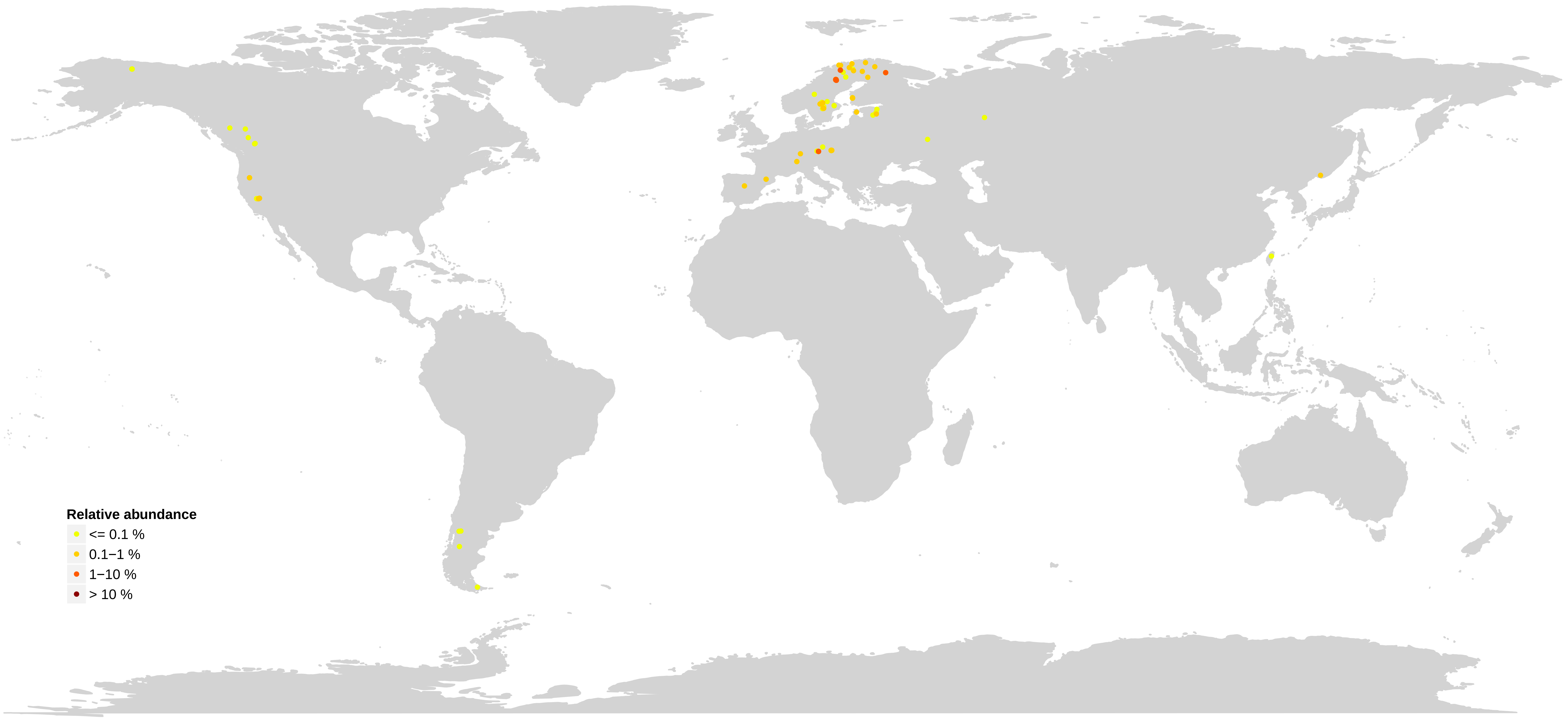

SH408326 Peyronellaea sancta

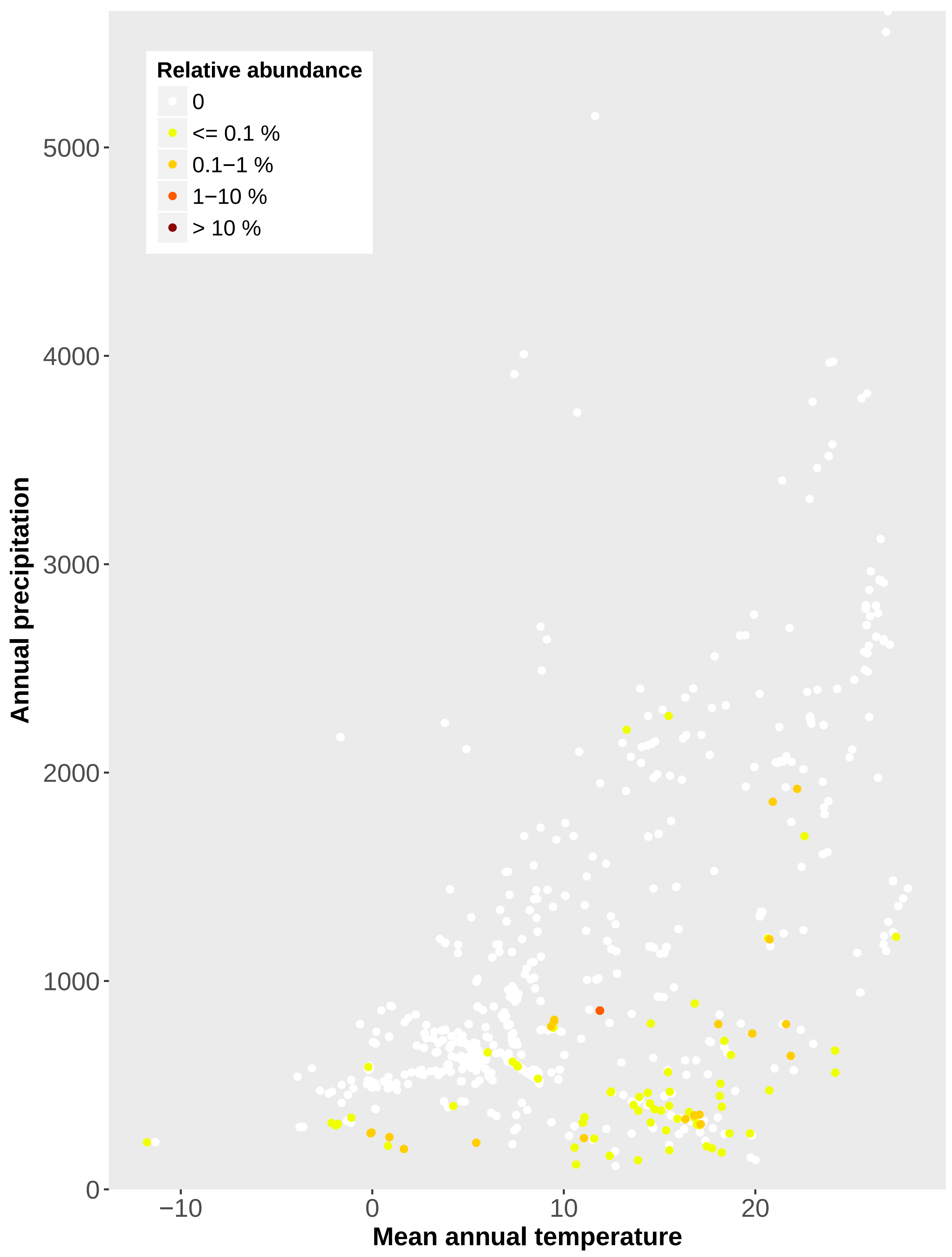

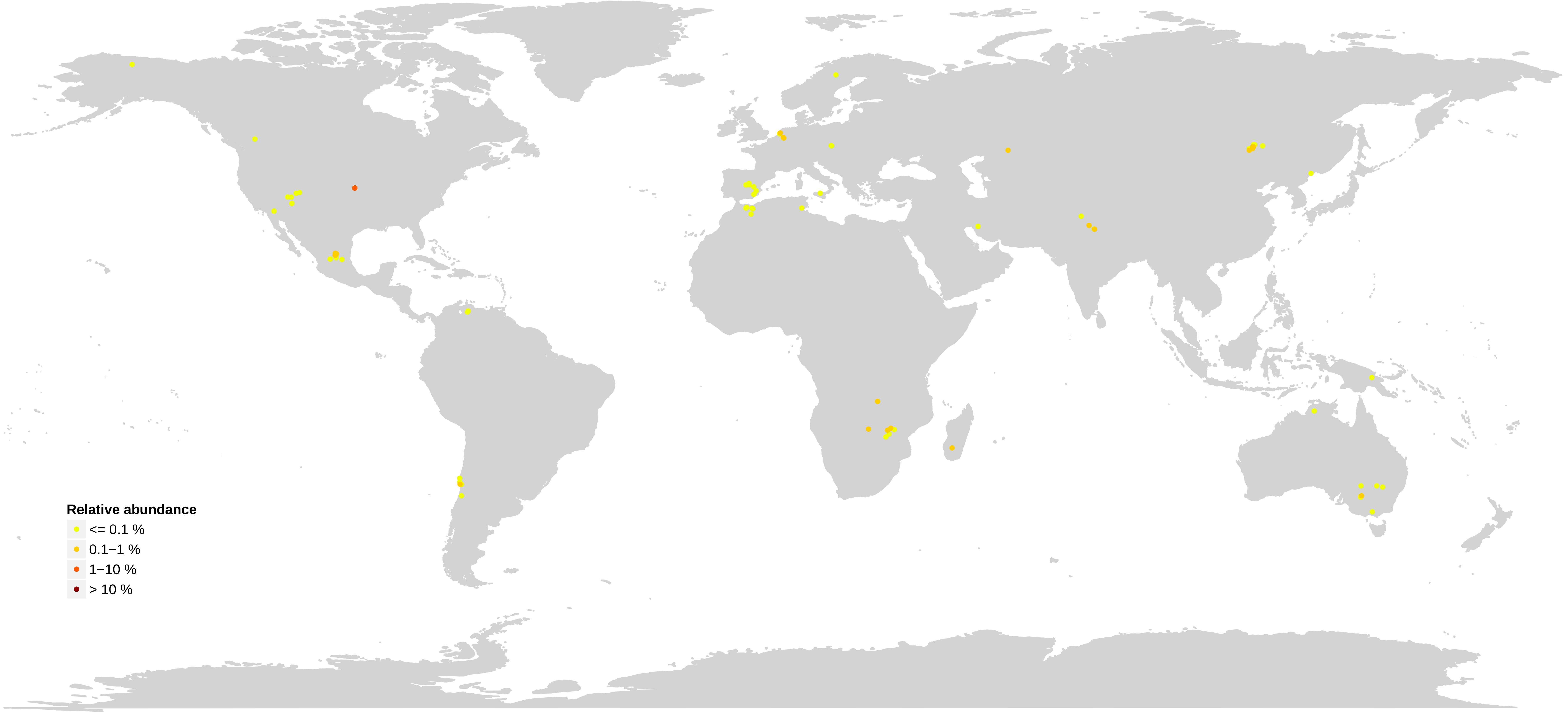

SH491957 Ascomycota sp

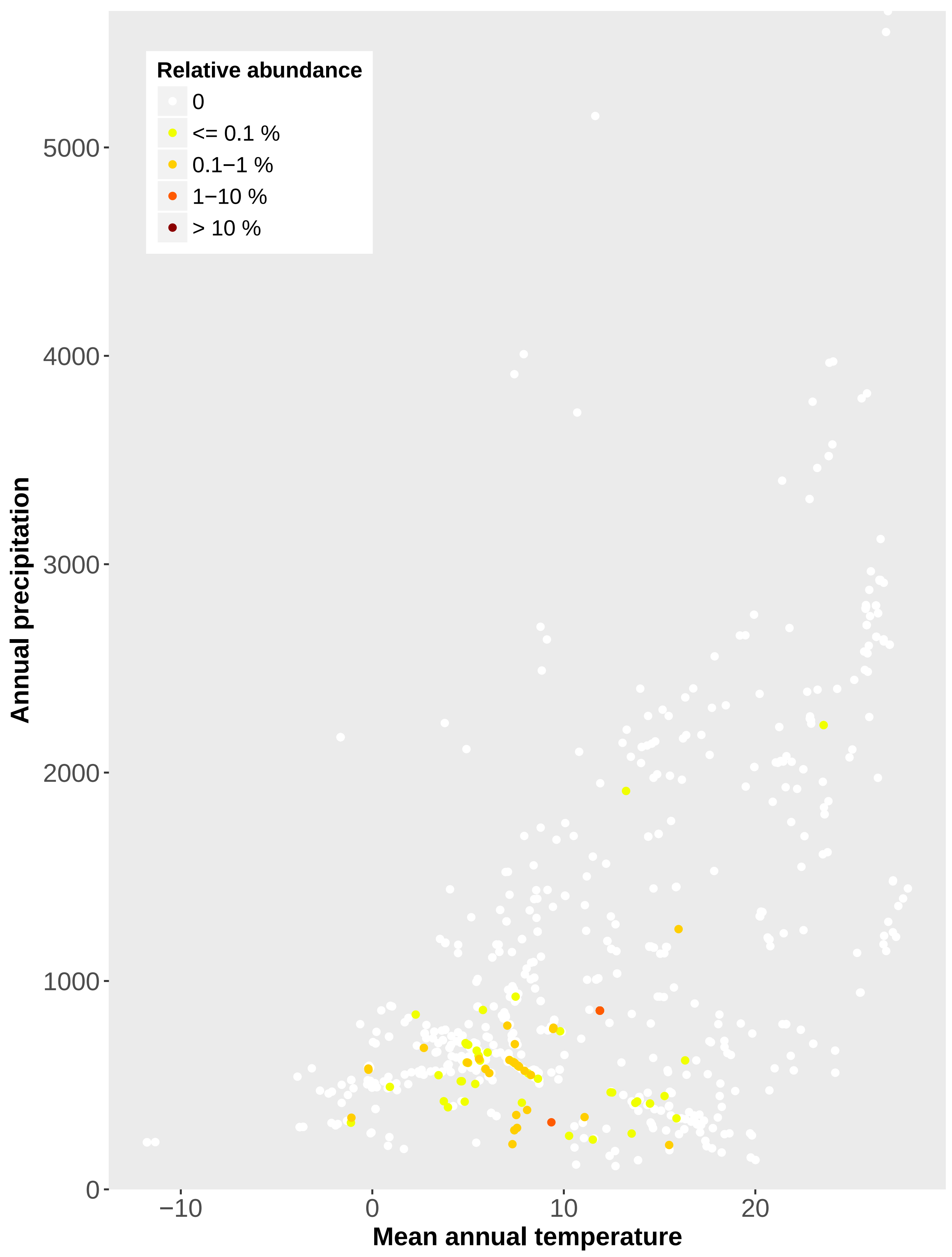

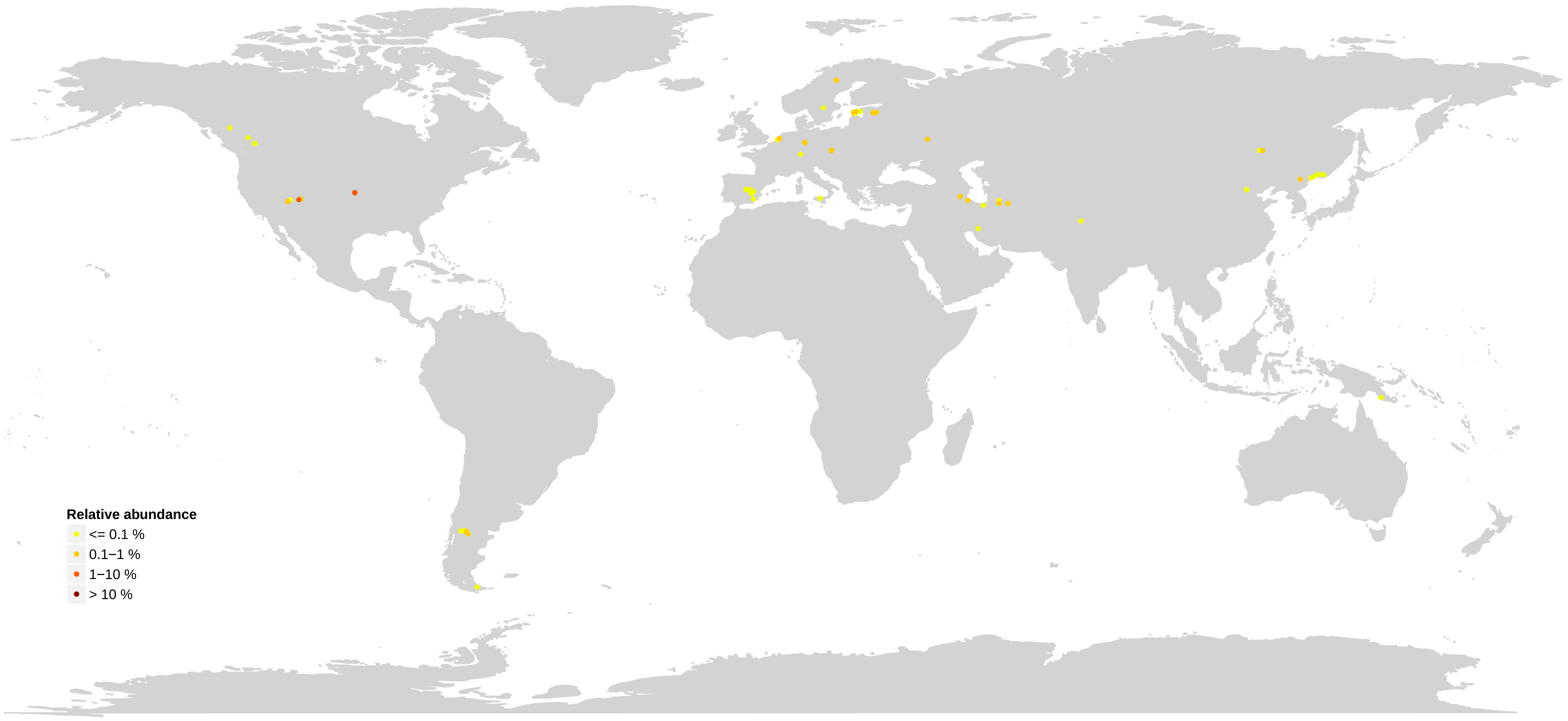

SH216987 Oidiodendron maius

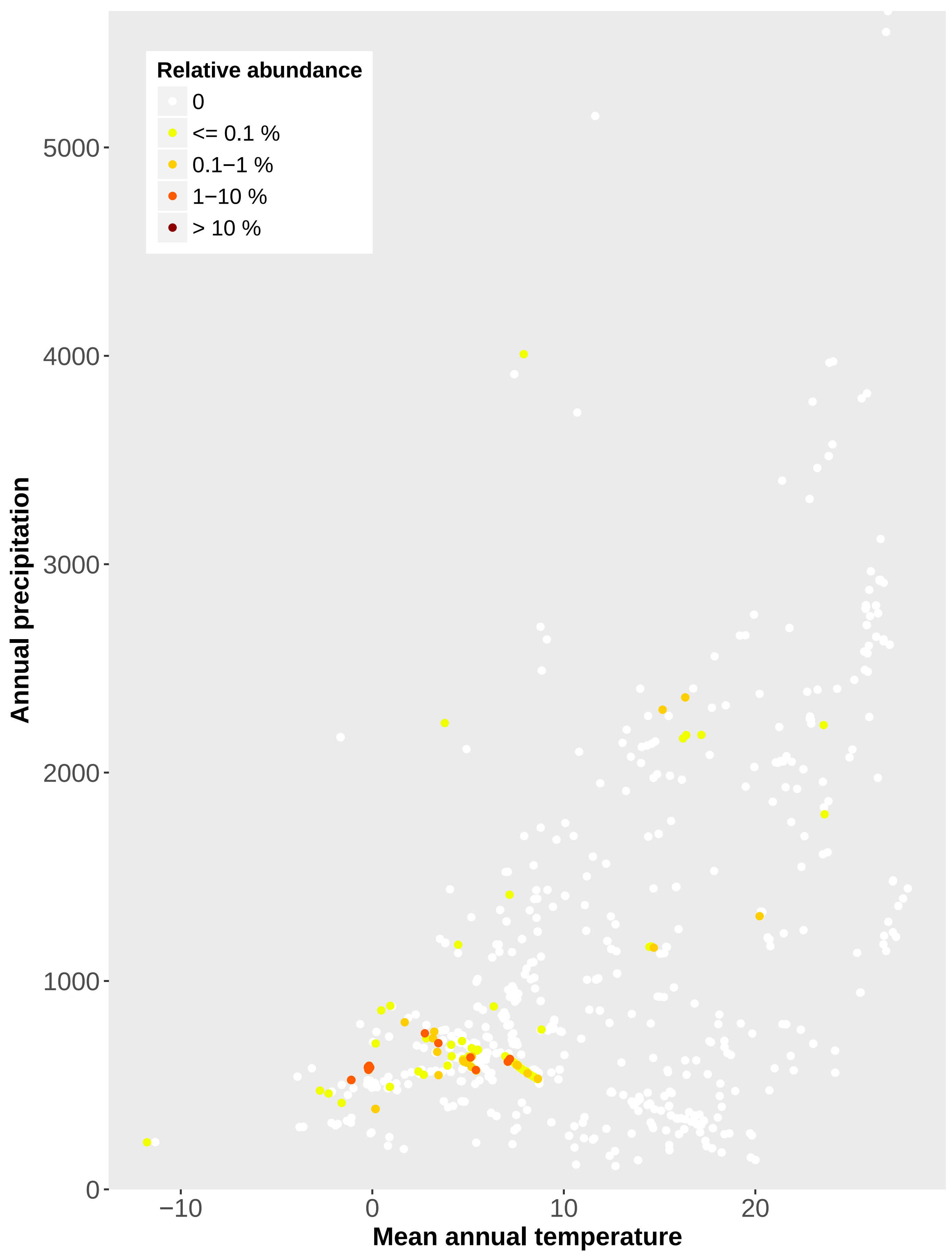

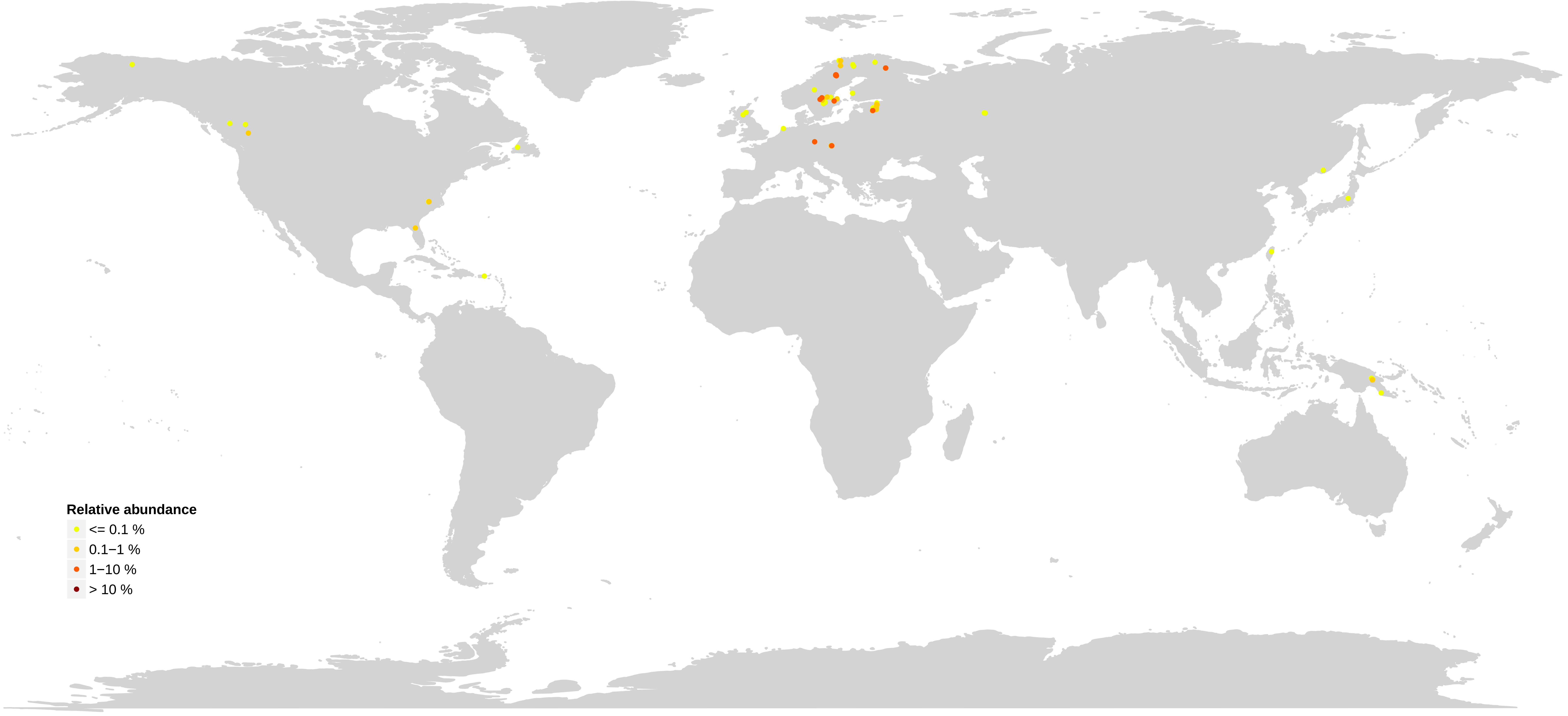

SH182678 Clonostachys rosea

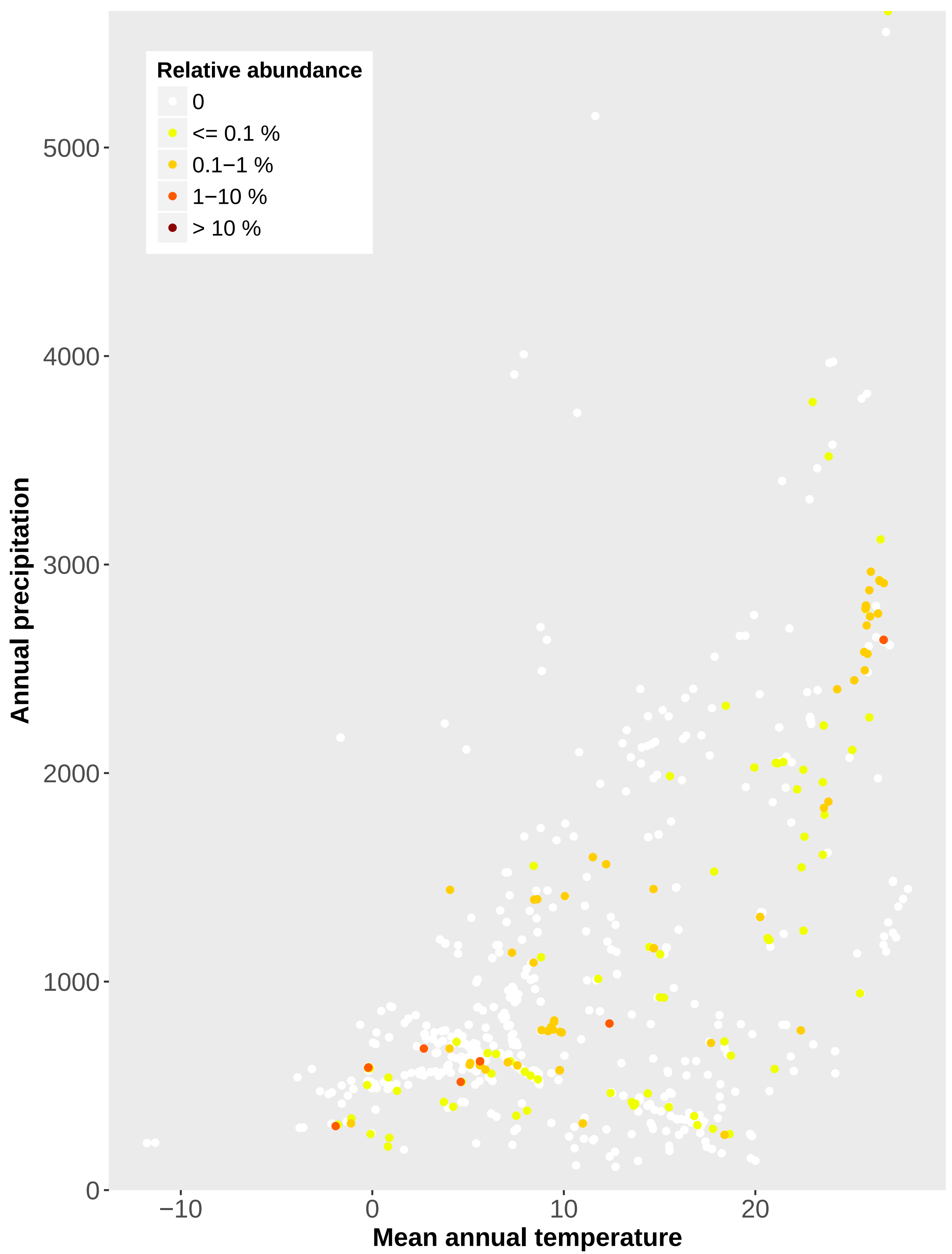

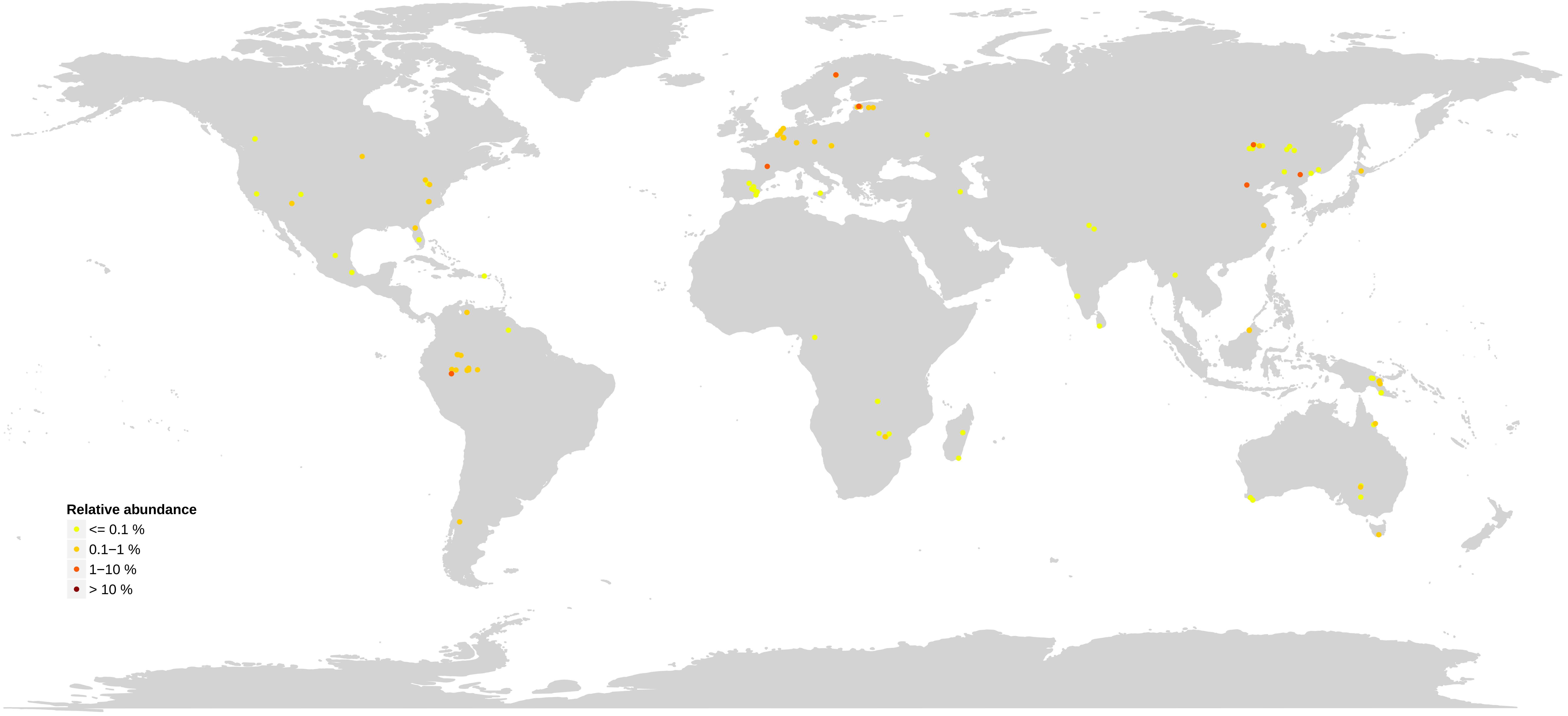

SH372021 Penicillium cairnsense

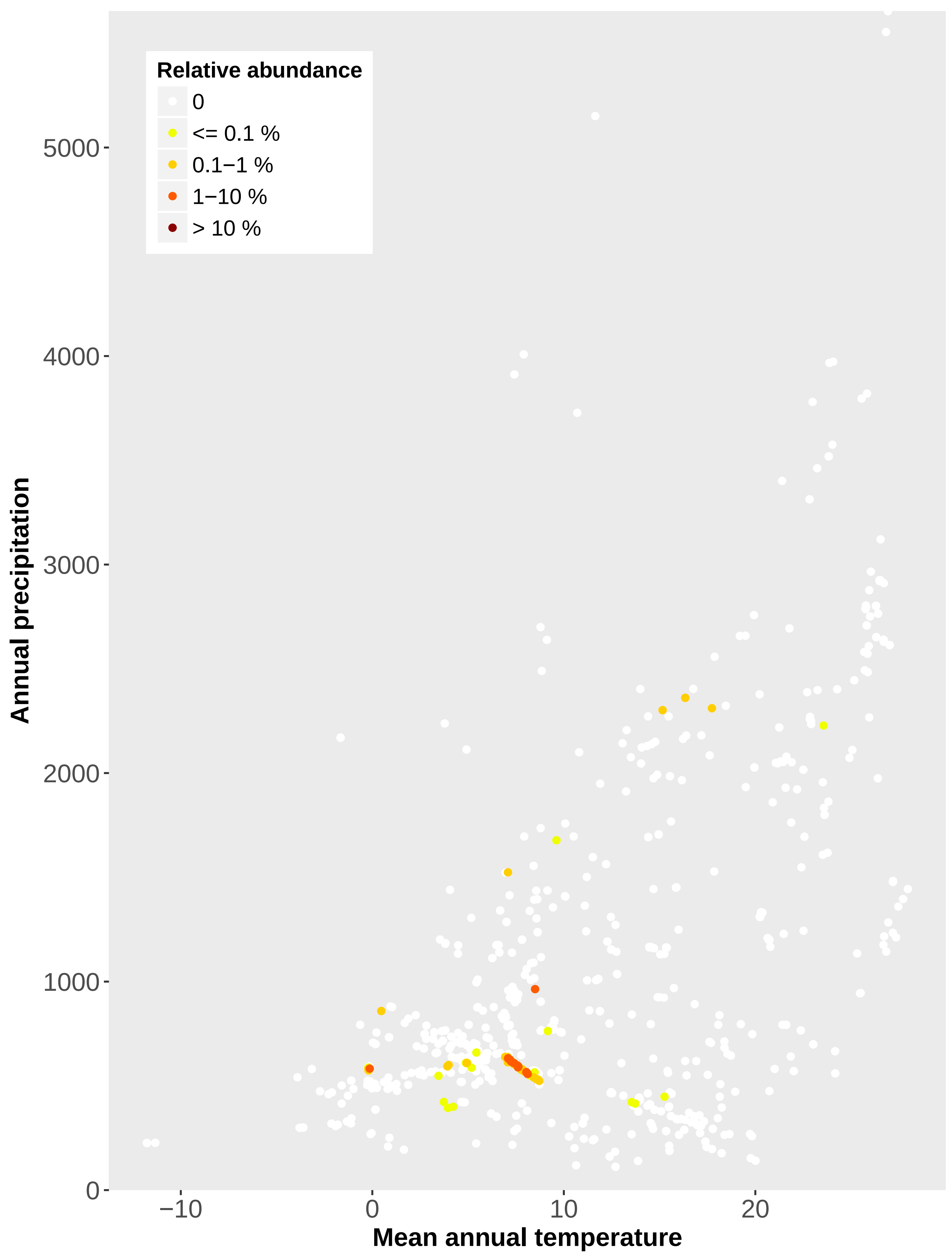

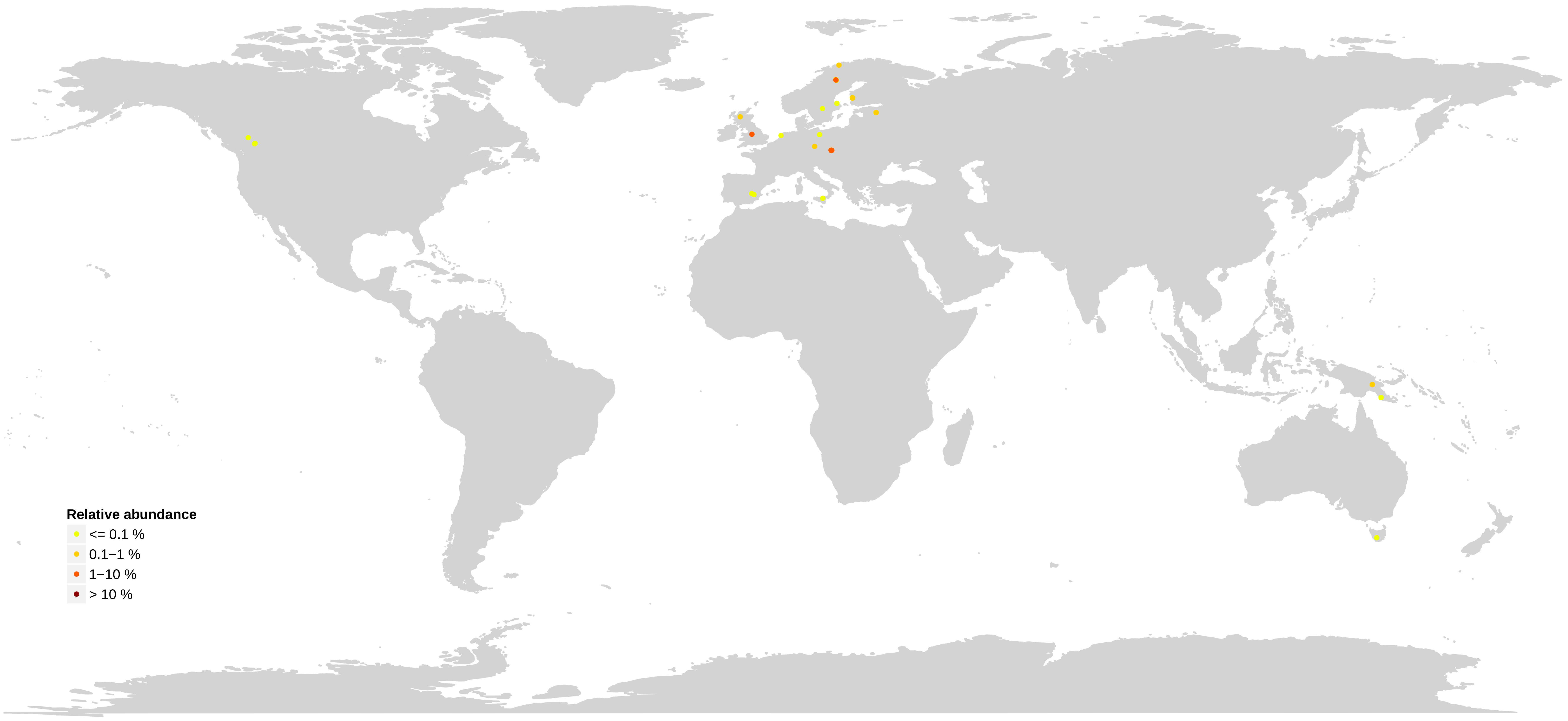

SH185196 Mortierella gemmifera

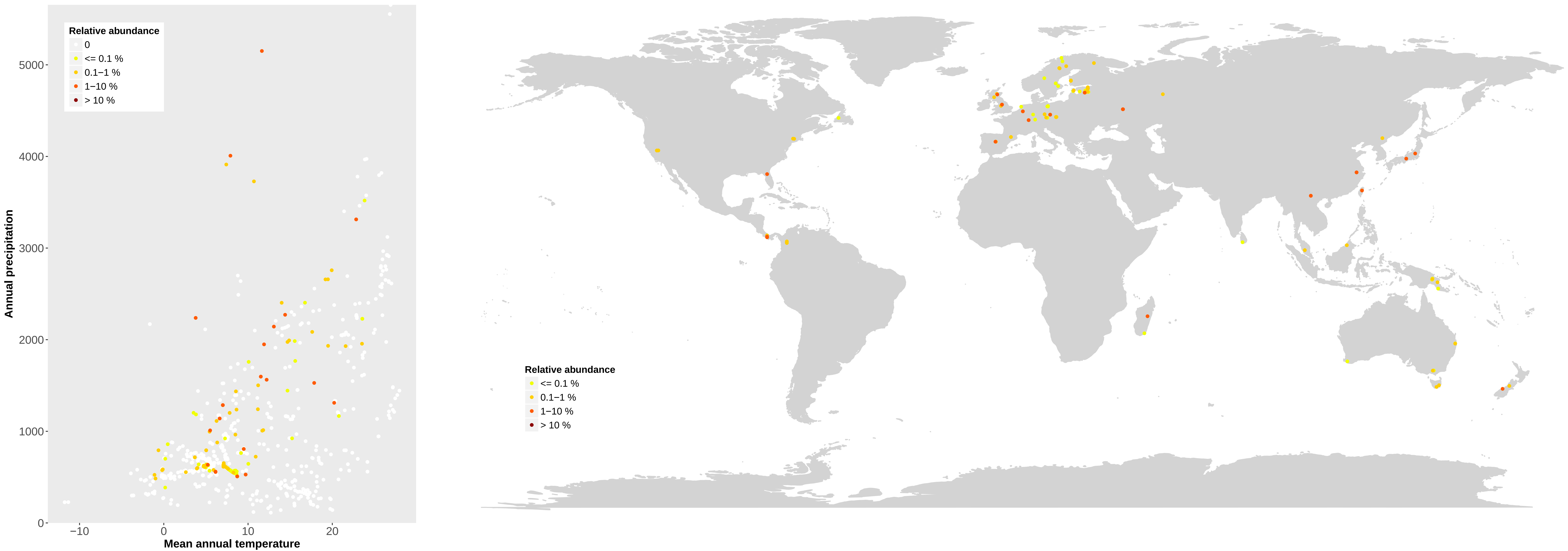

SH201404 Penicillium penicillioides

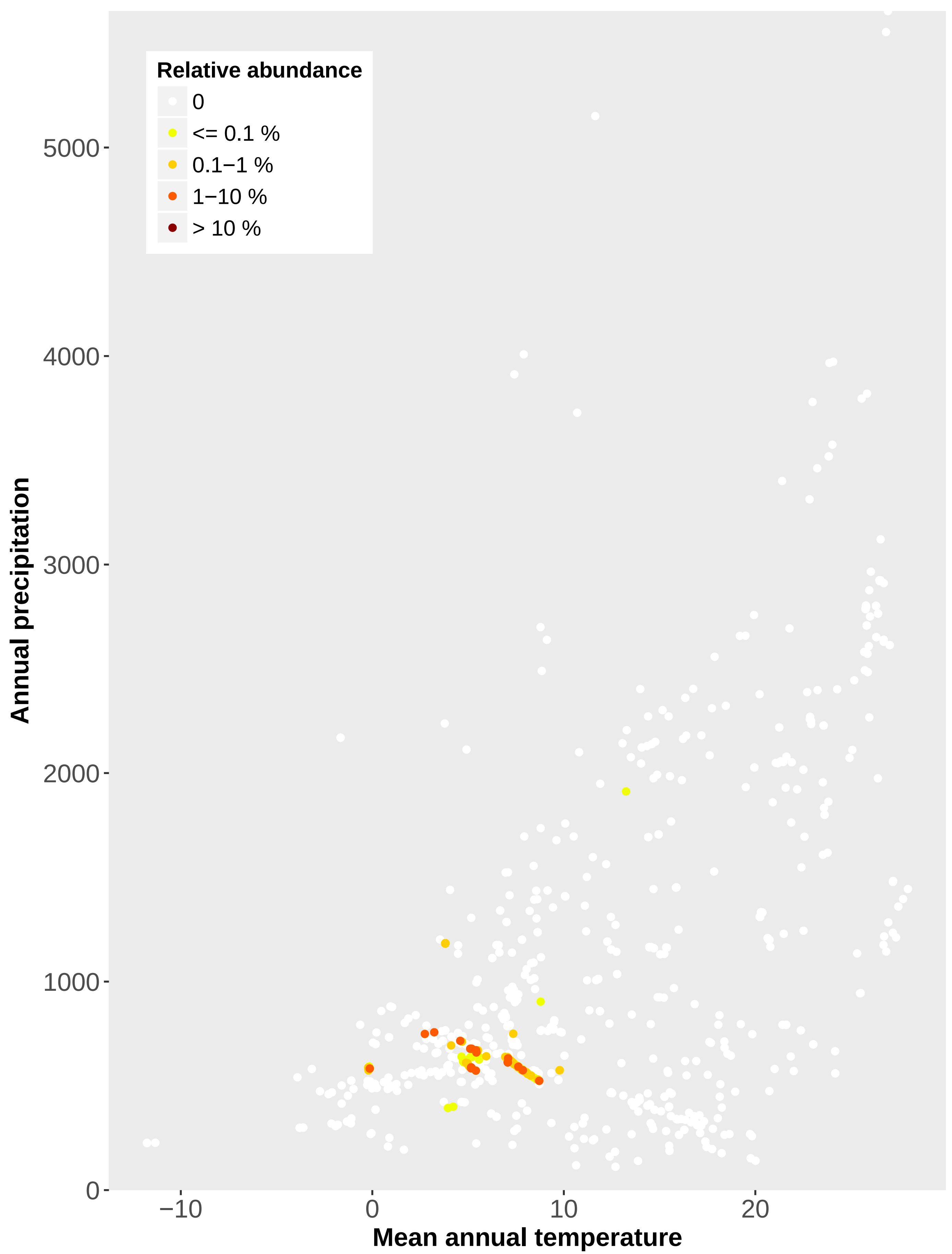

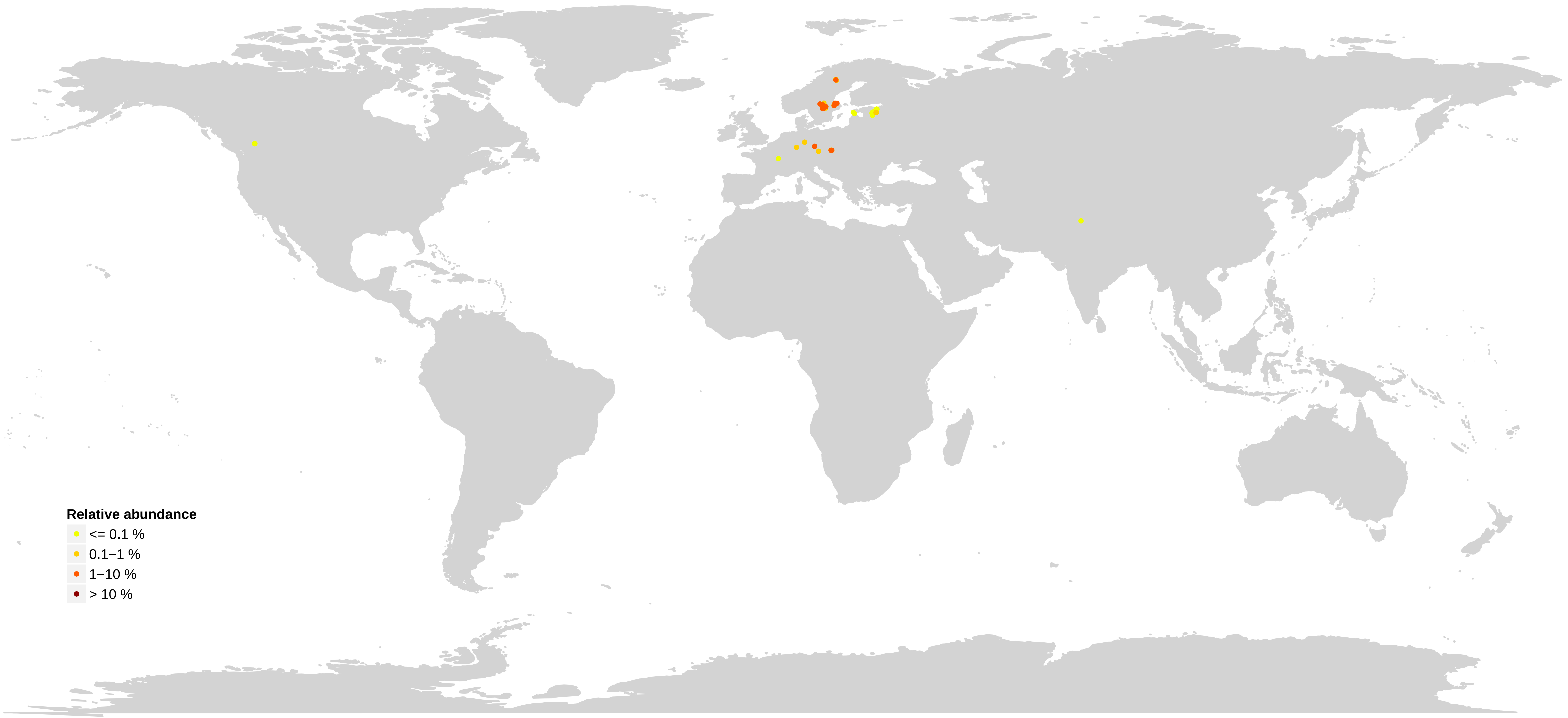

SH203693 Cadophora sp

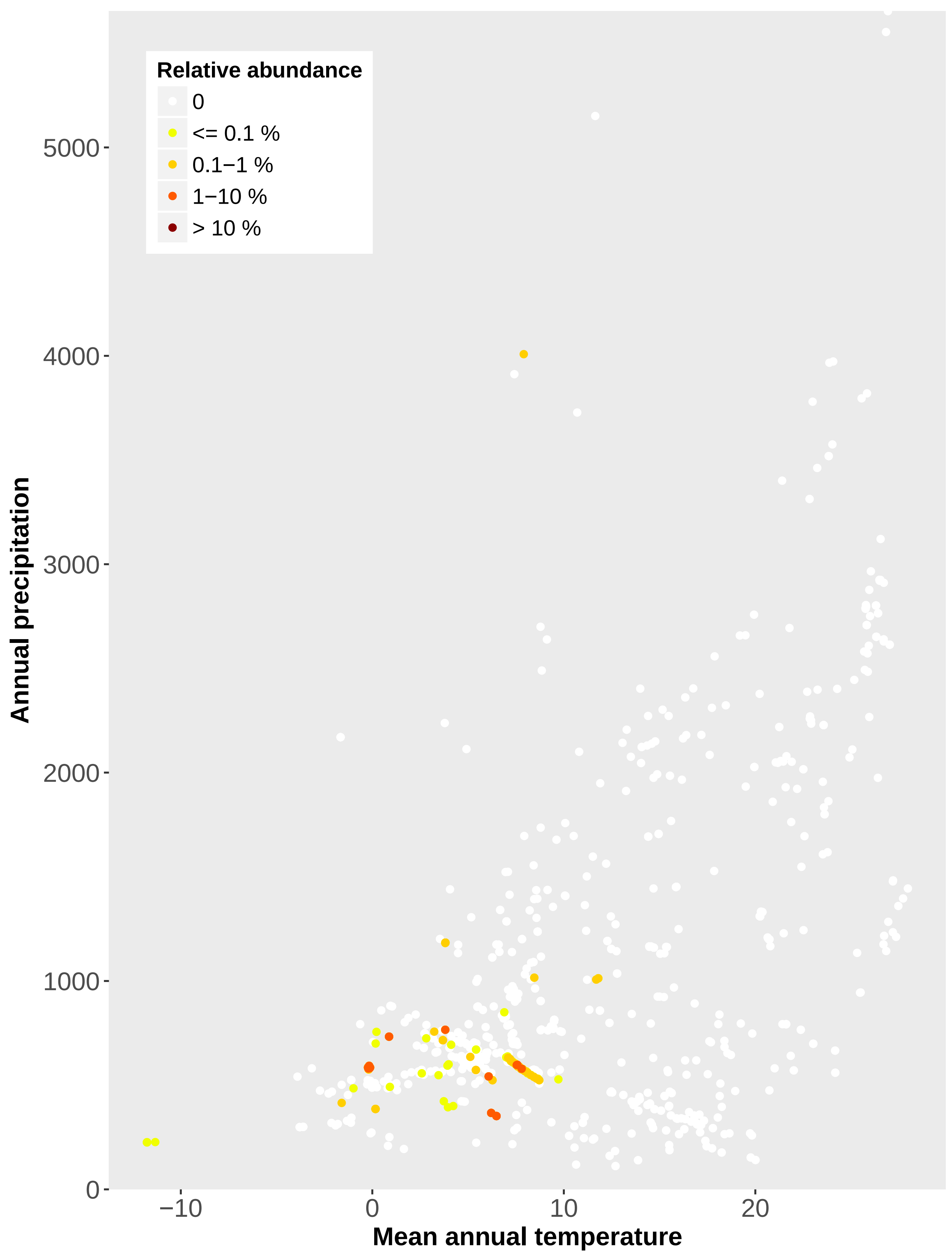

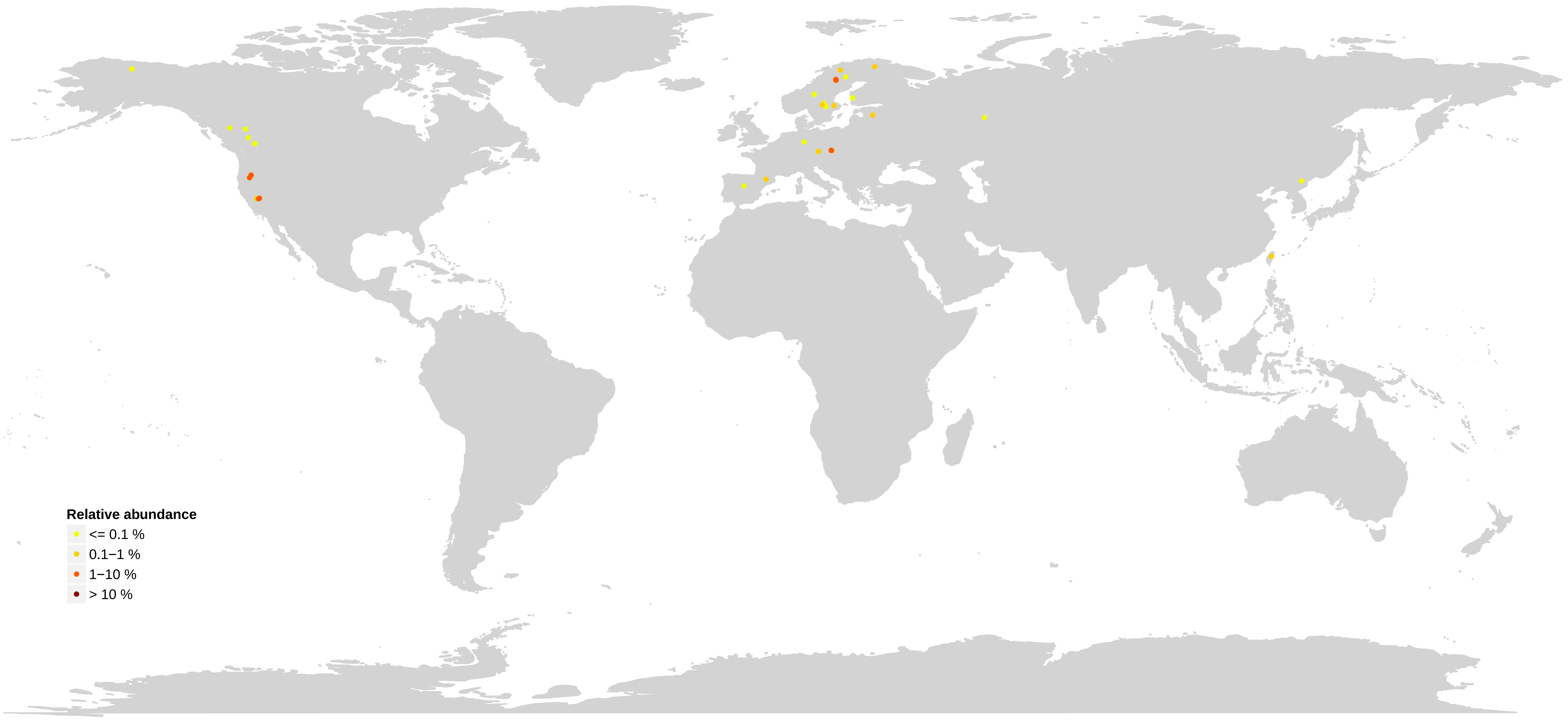

SH187861 Mortierella alliacea

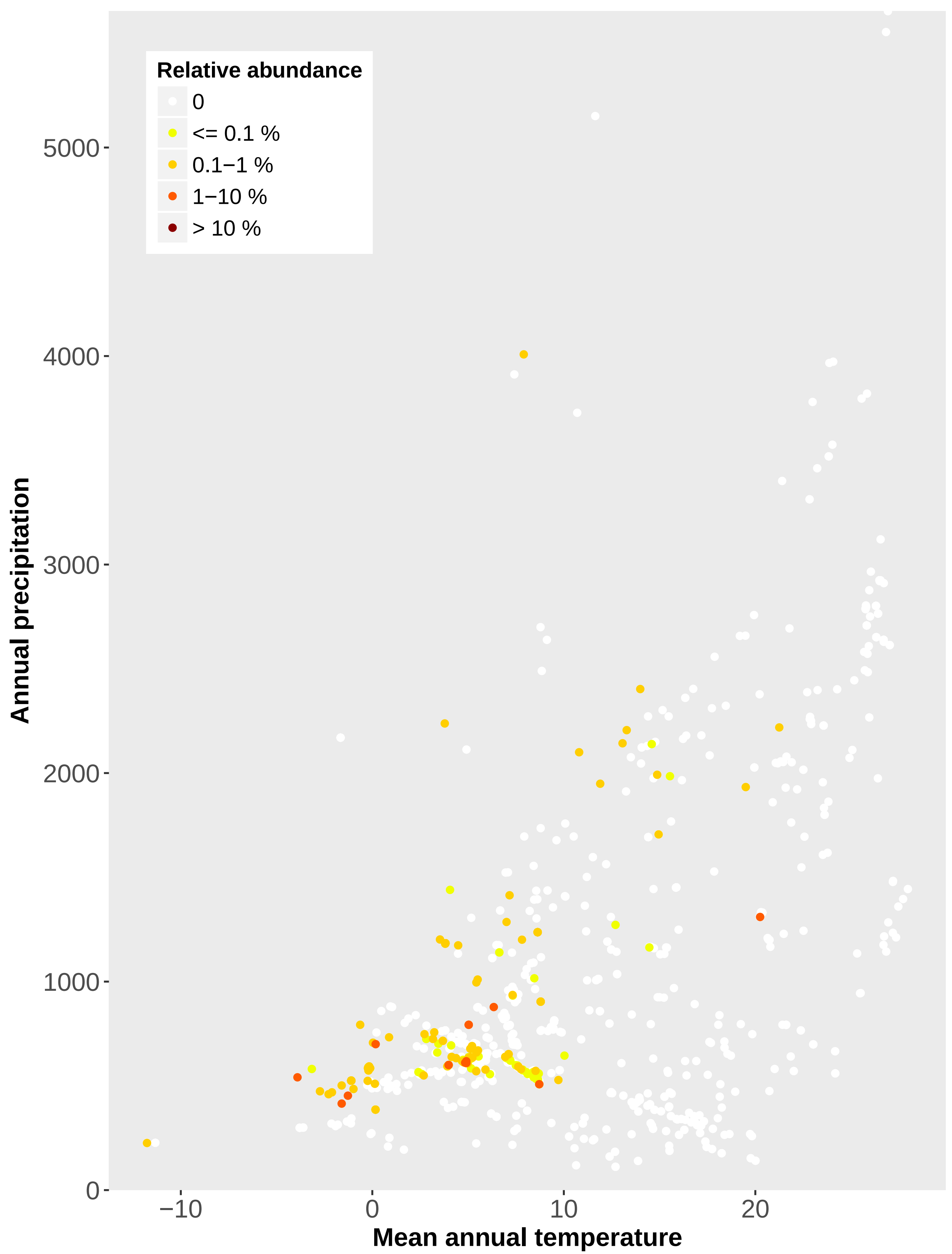

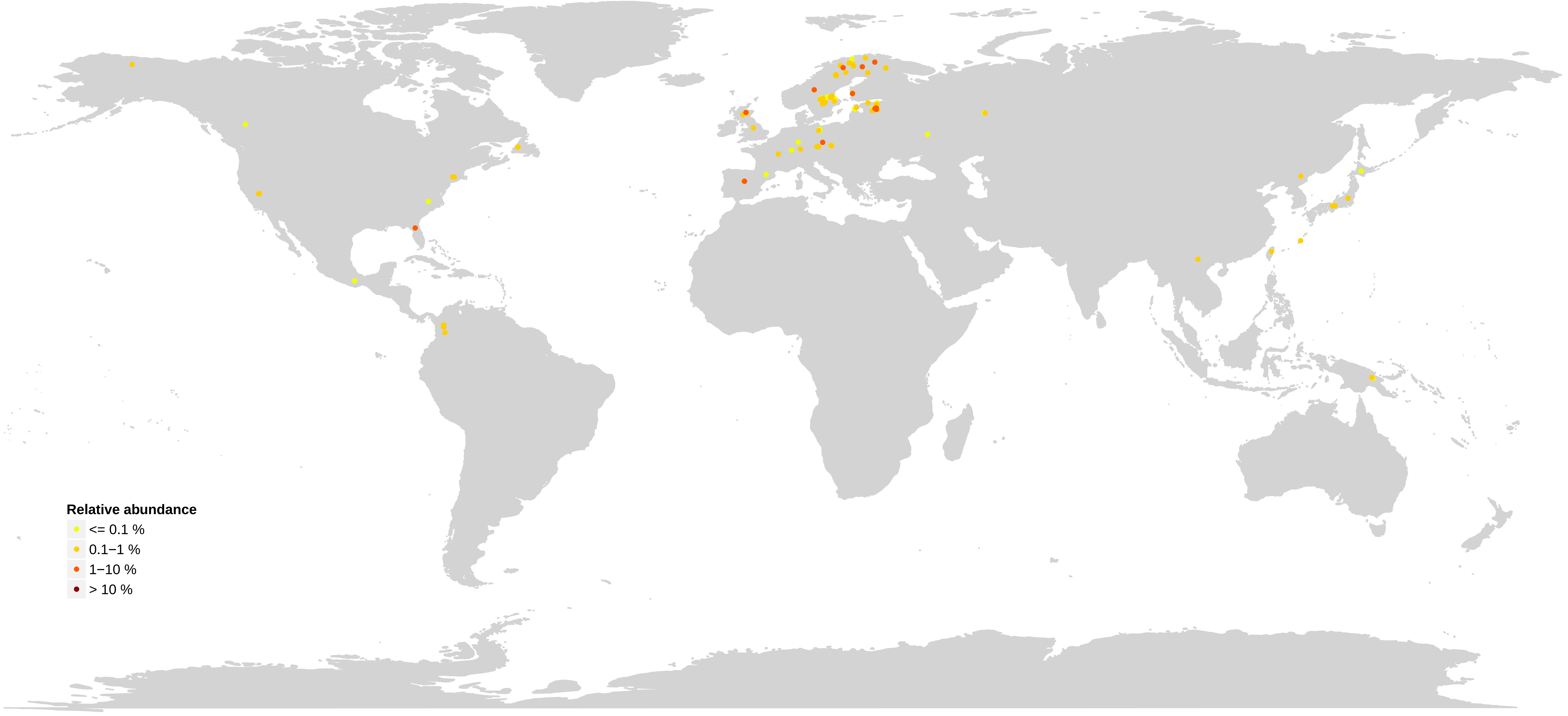

SH215250 Helotiales sp

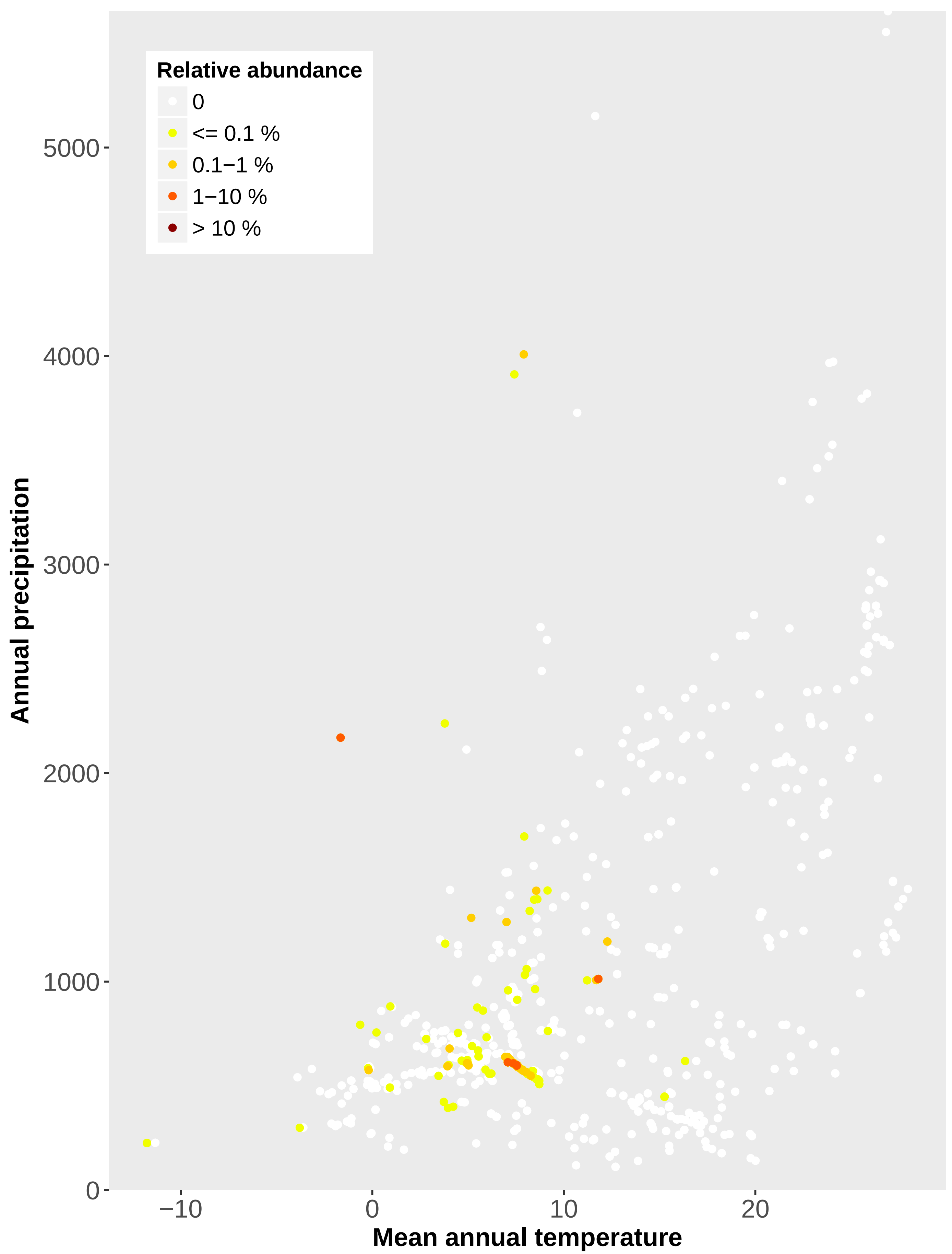

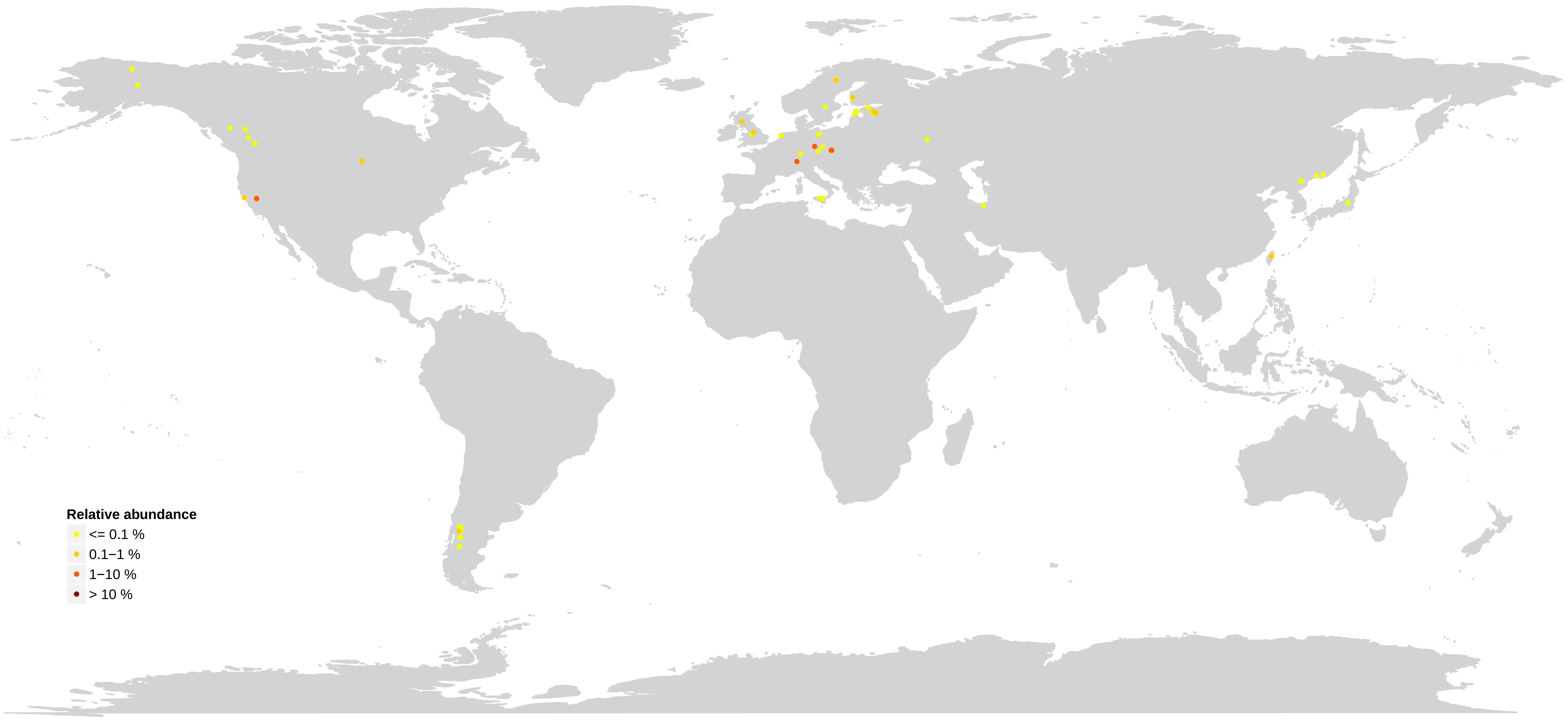

SH184180 Preussia flanaganii

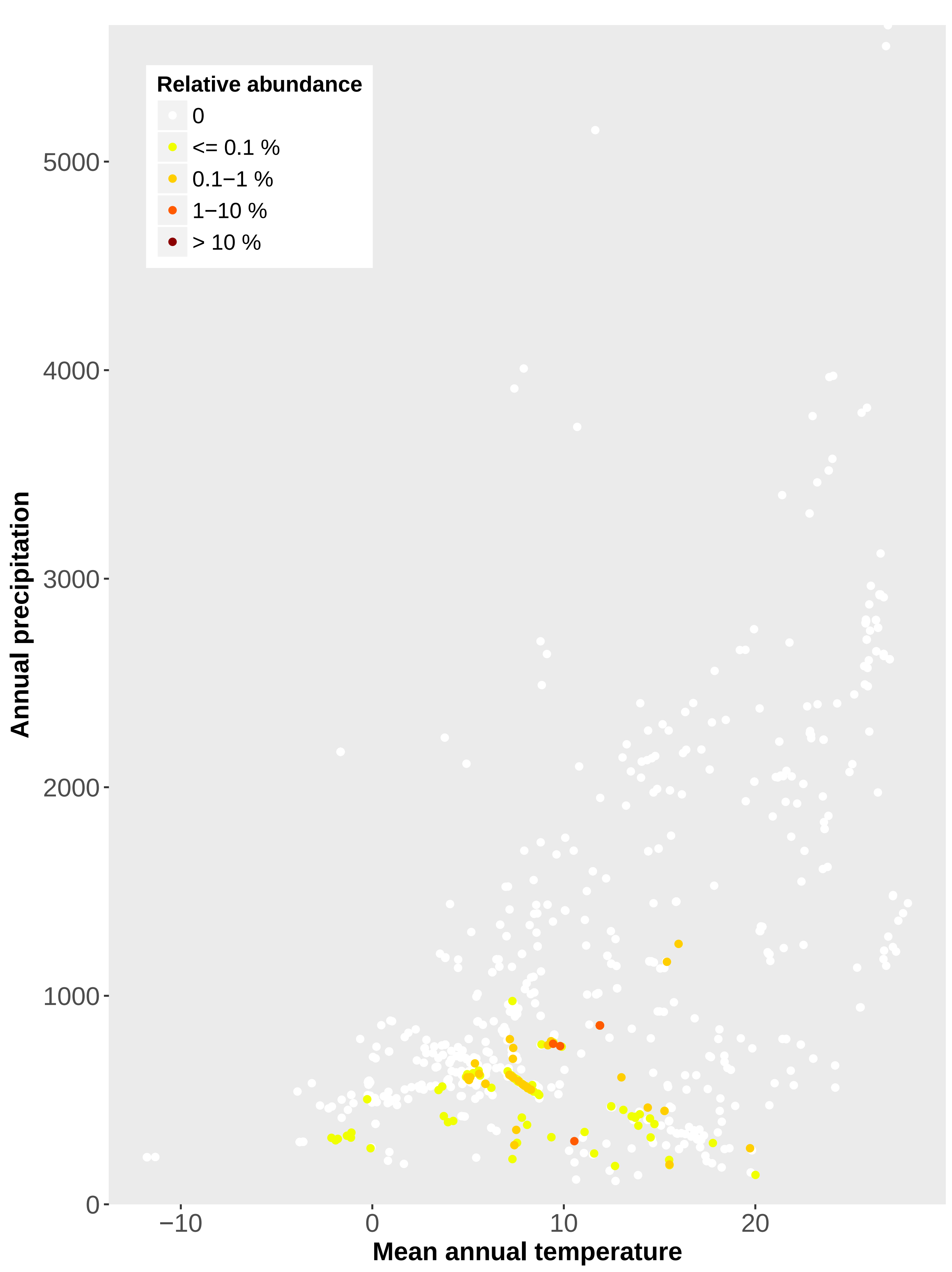

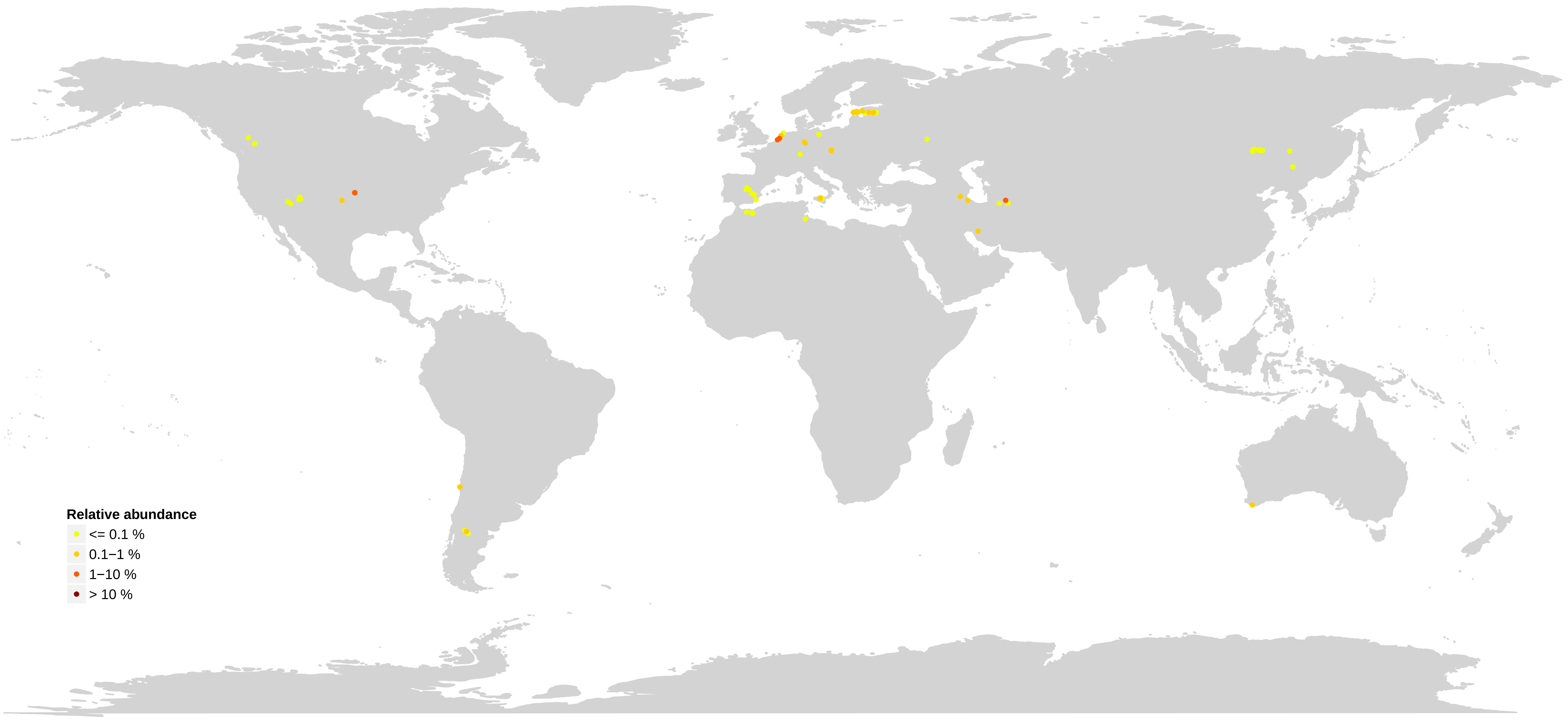

SH208845 Sagenomella diversispora

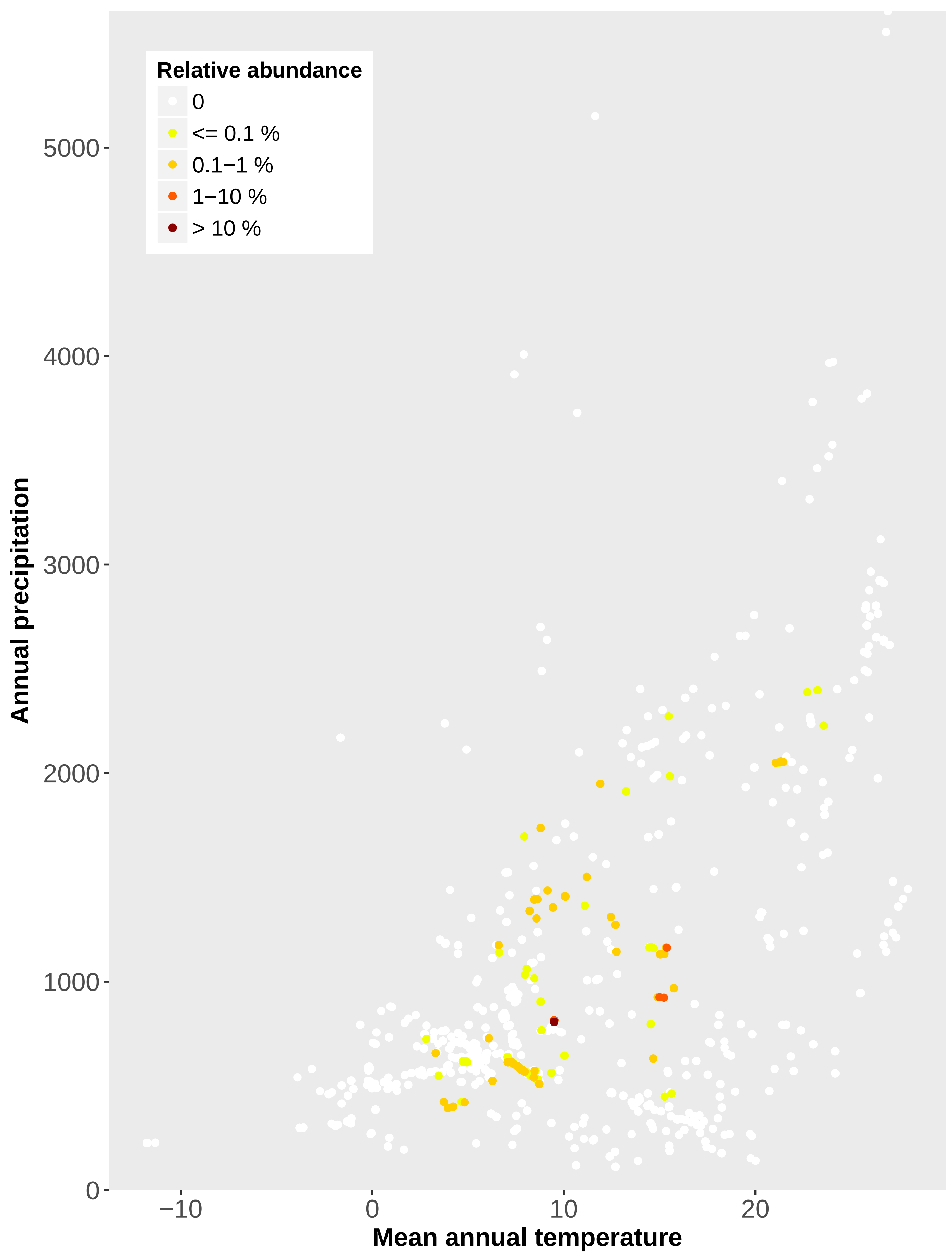

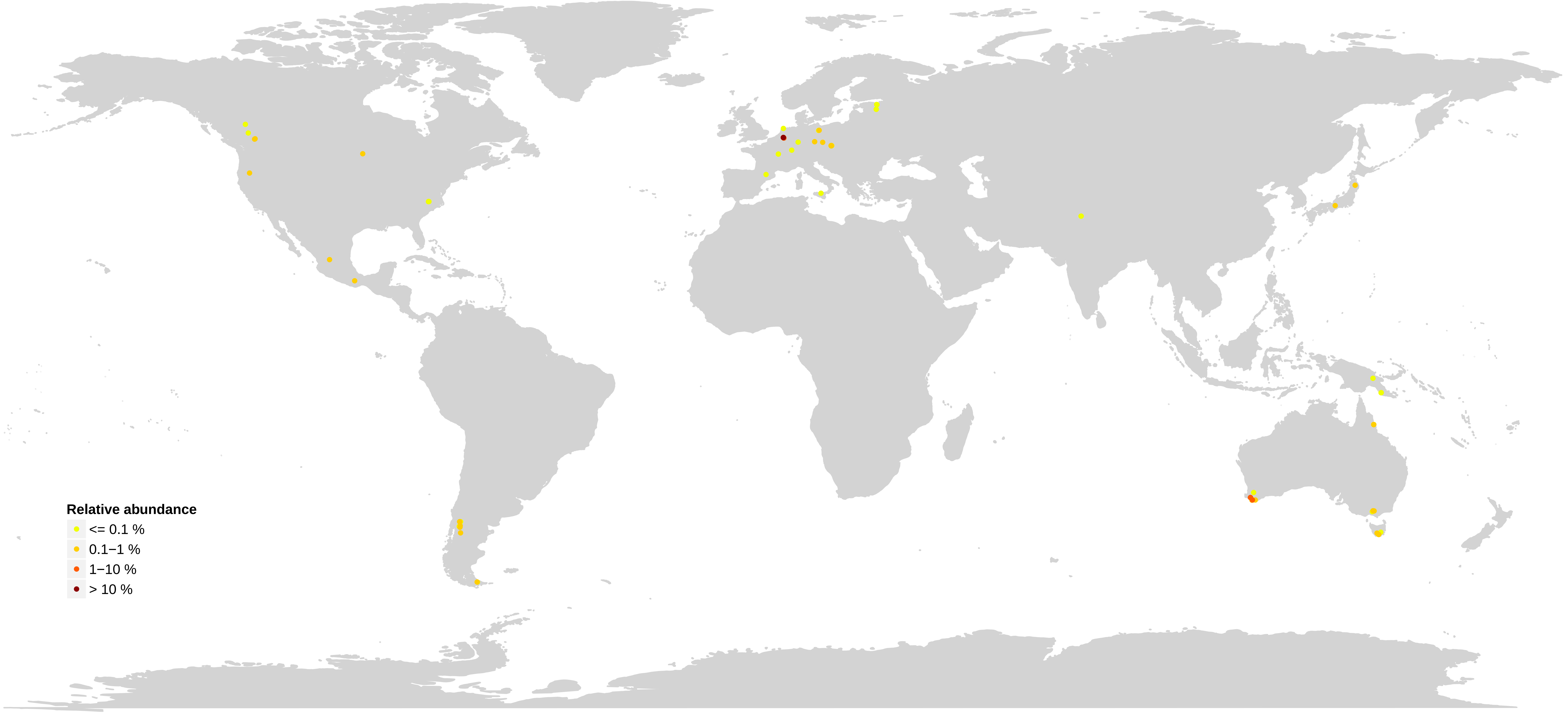

SH497383 Mortierellaceae sp

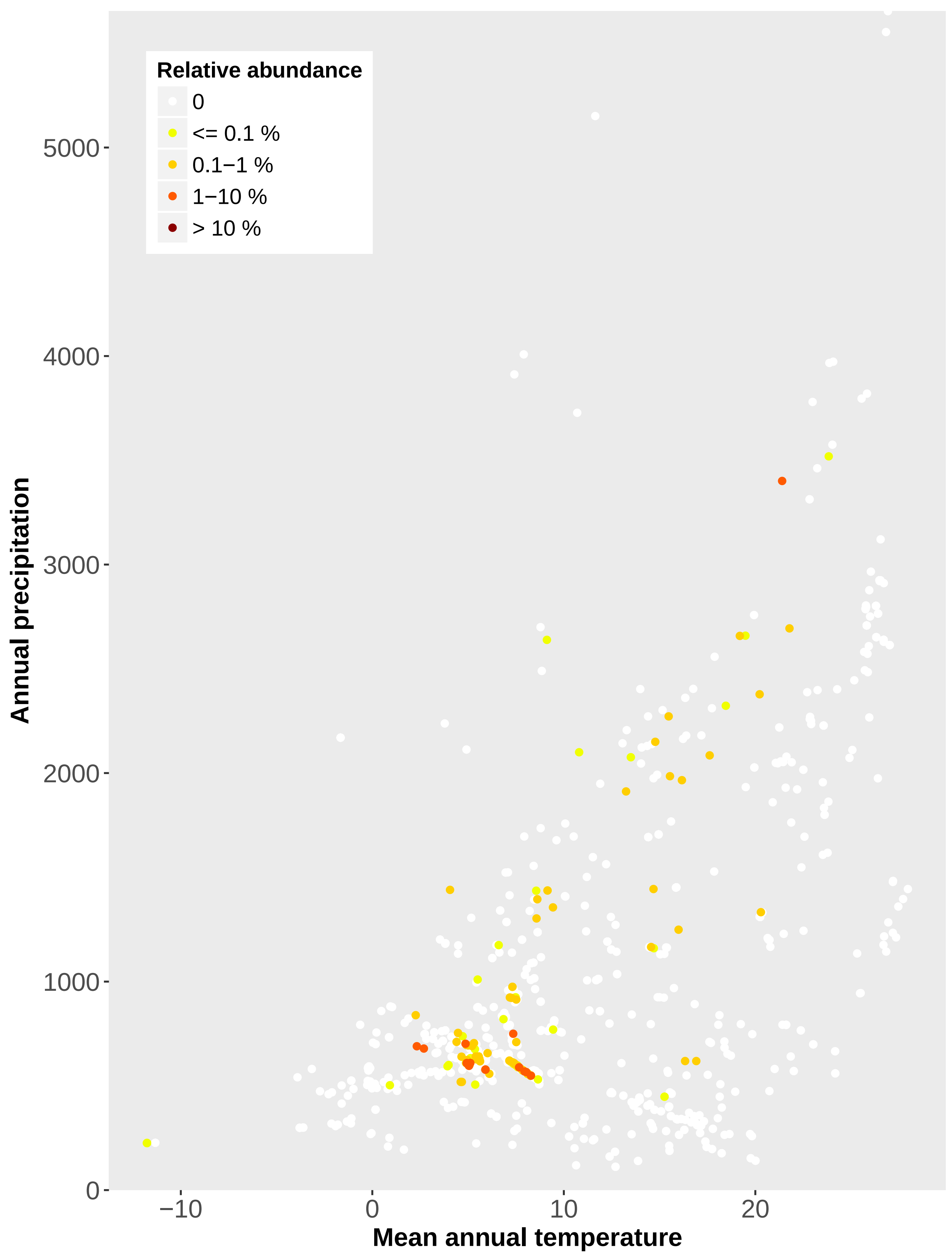

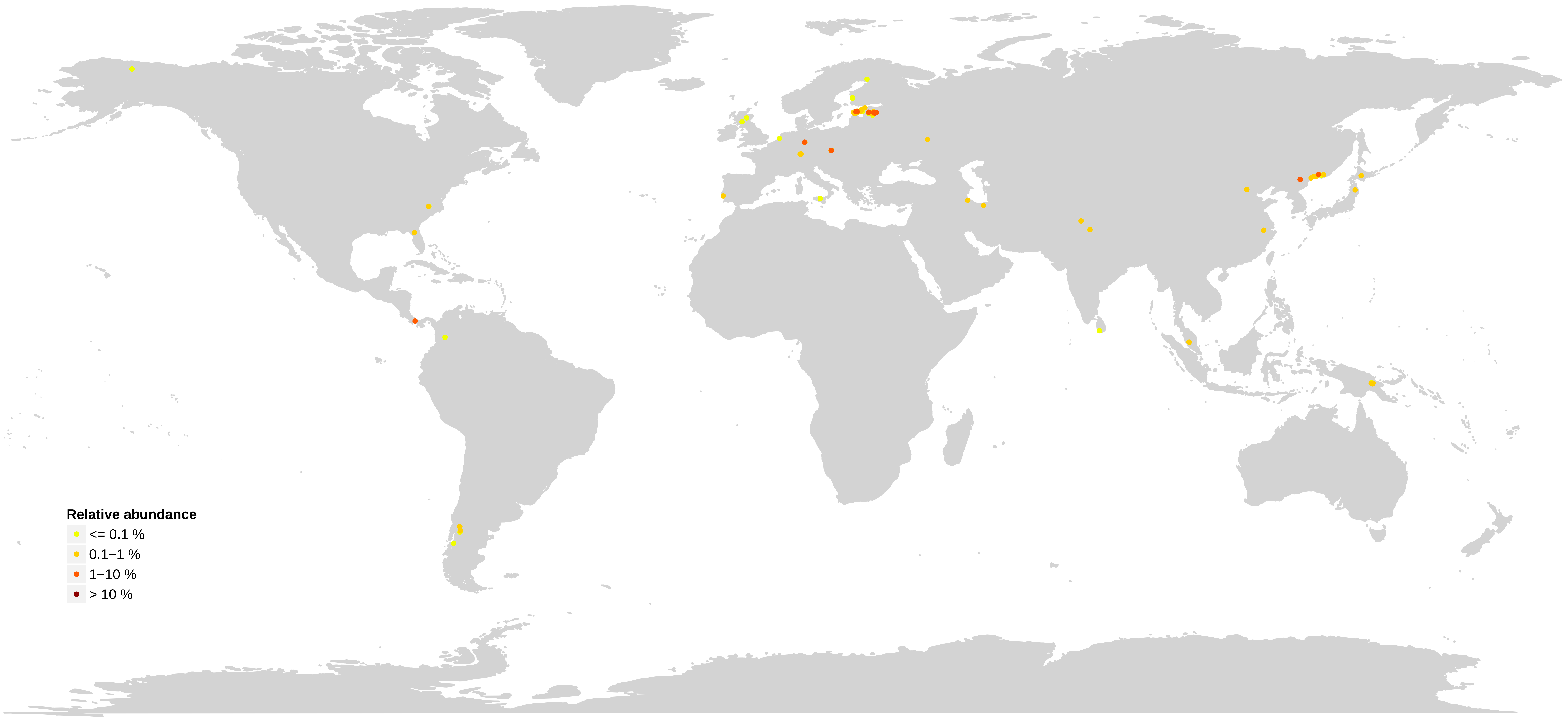

SH213261 Cladophialophora sp

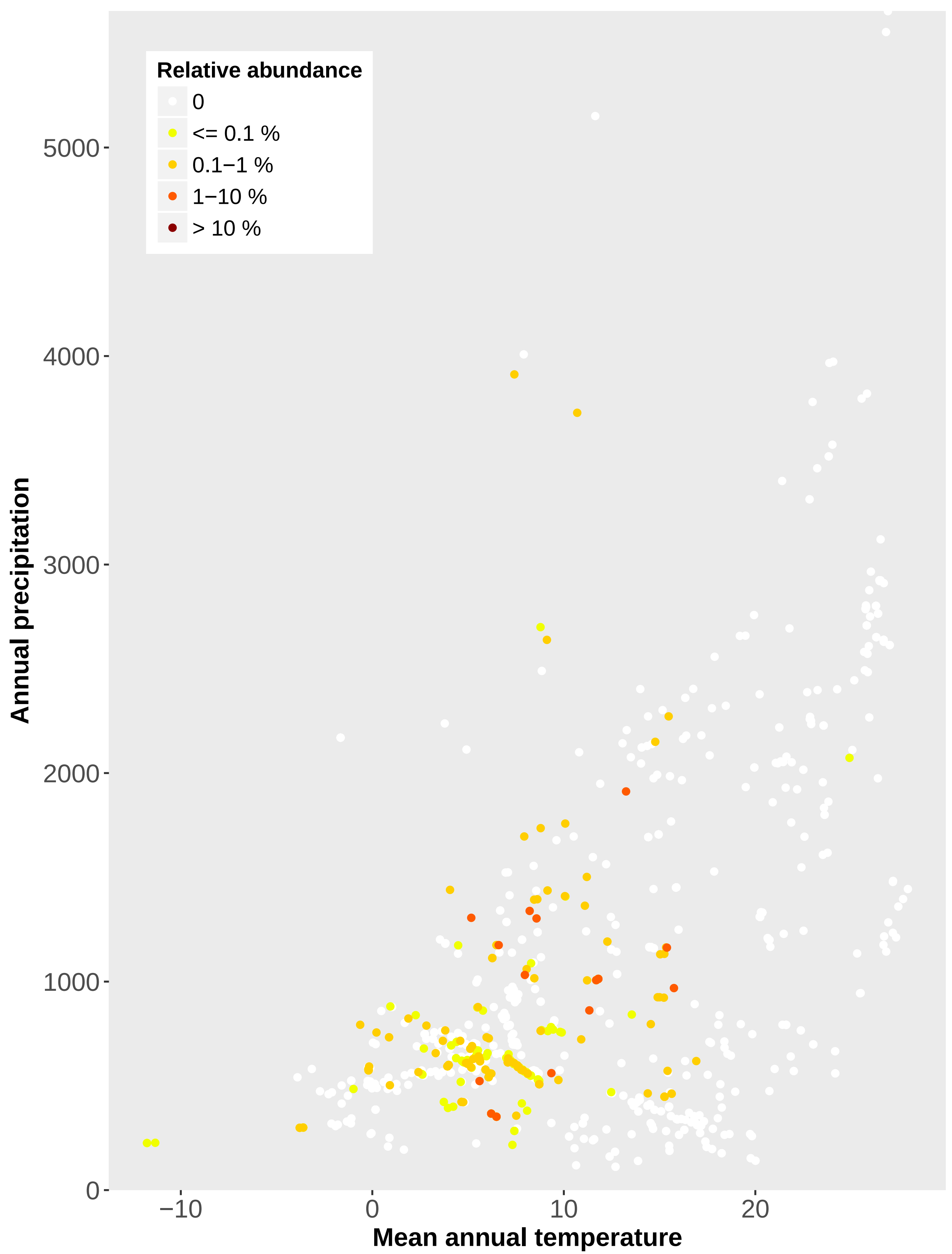

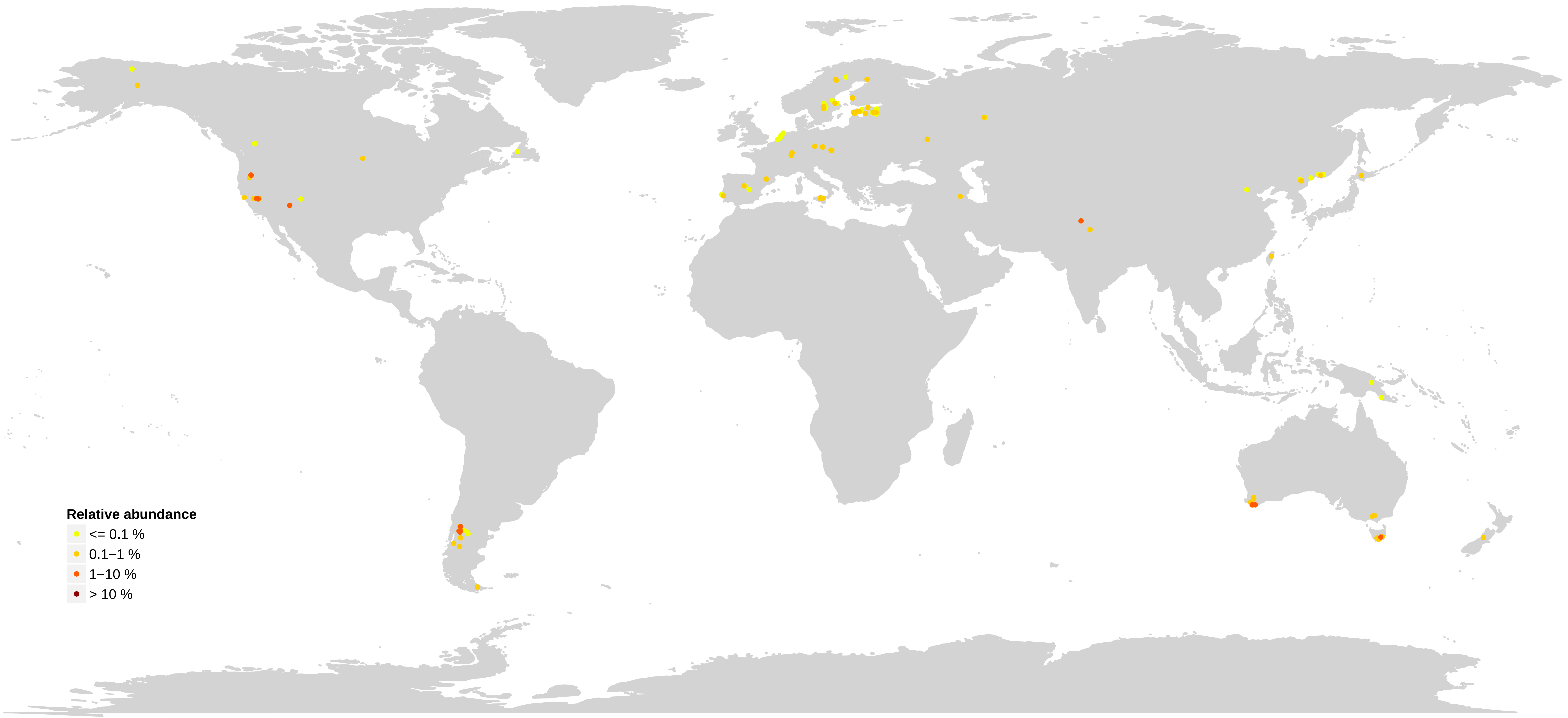

SH181628 Cryptococcus victoriae

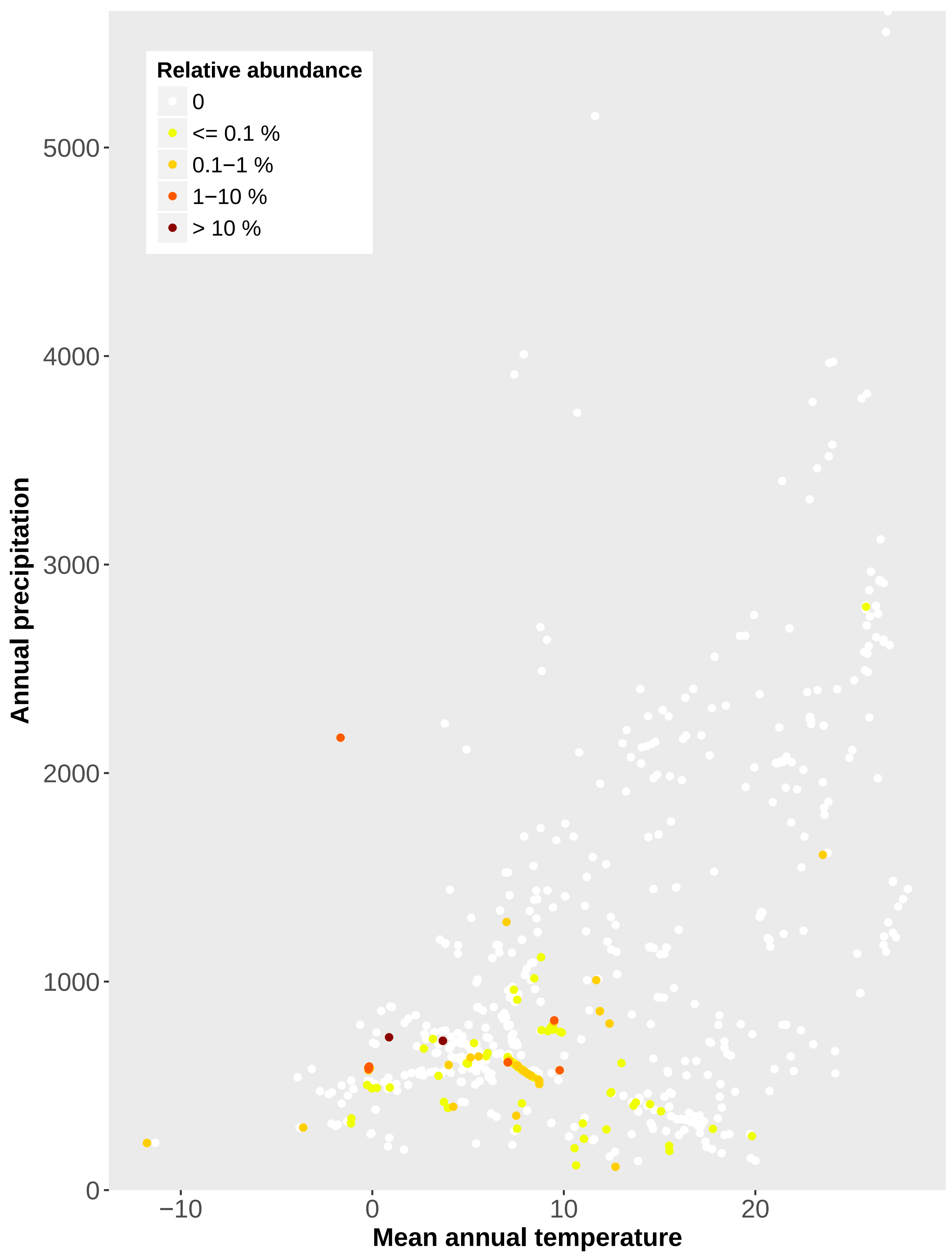

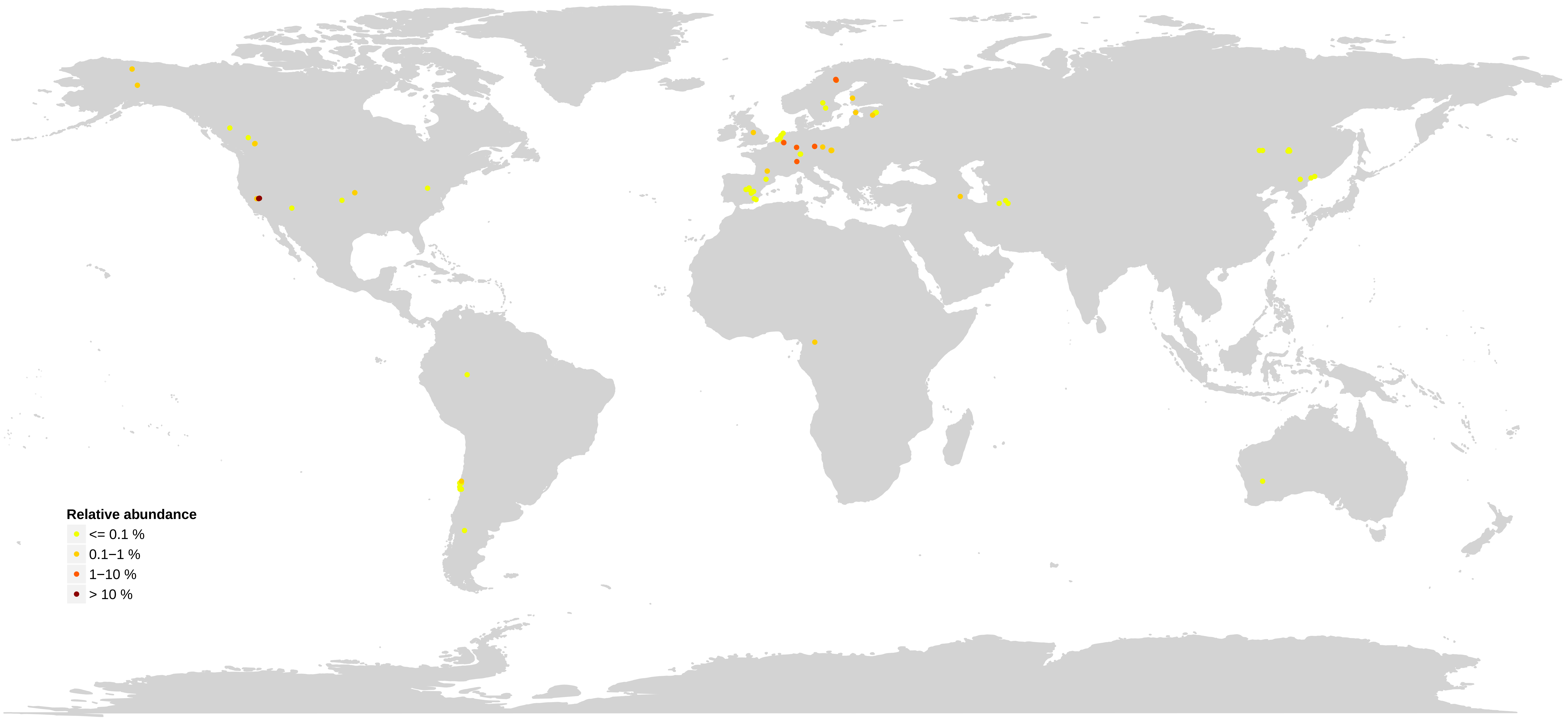

SH329706 Trichosporon dulcitum

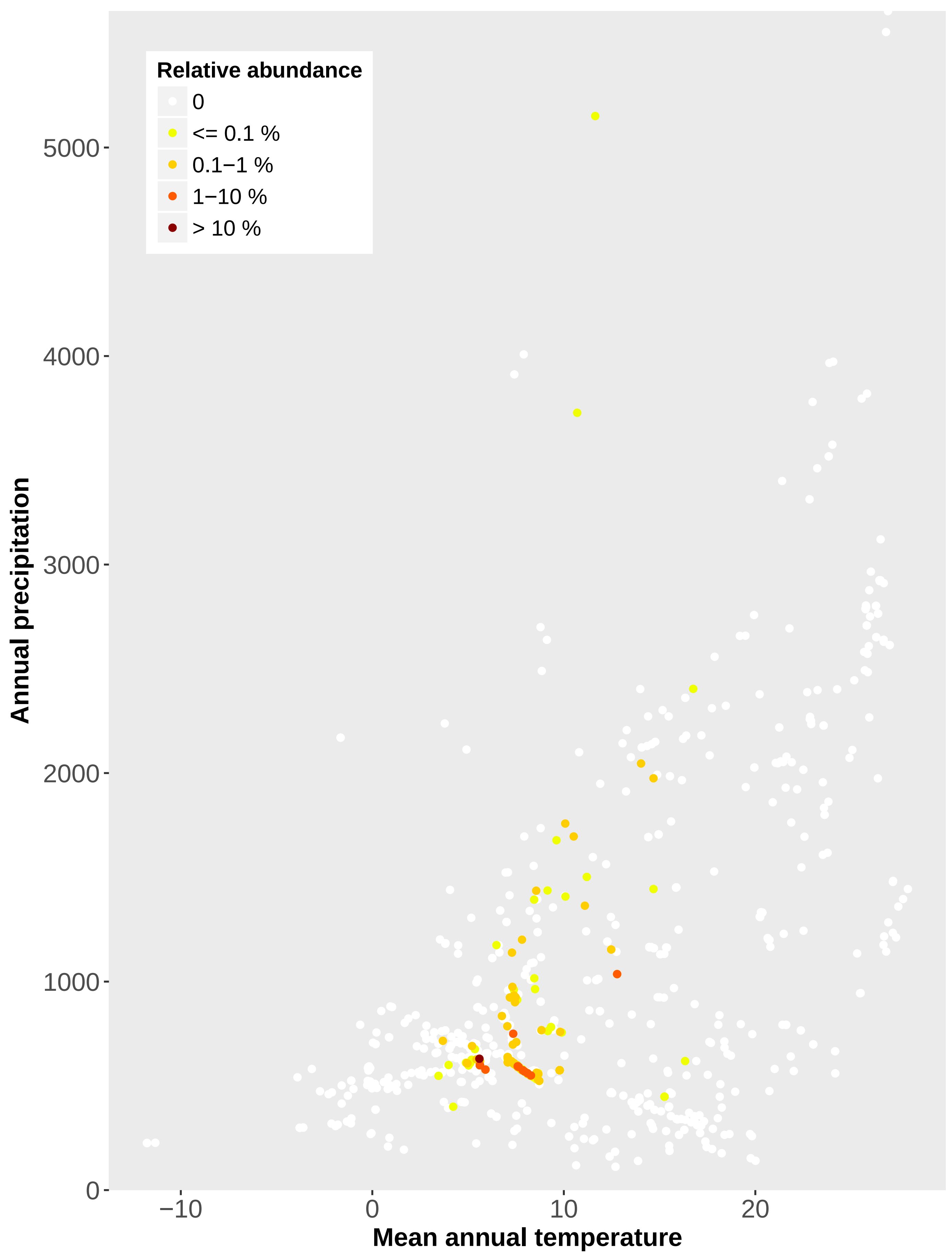

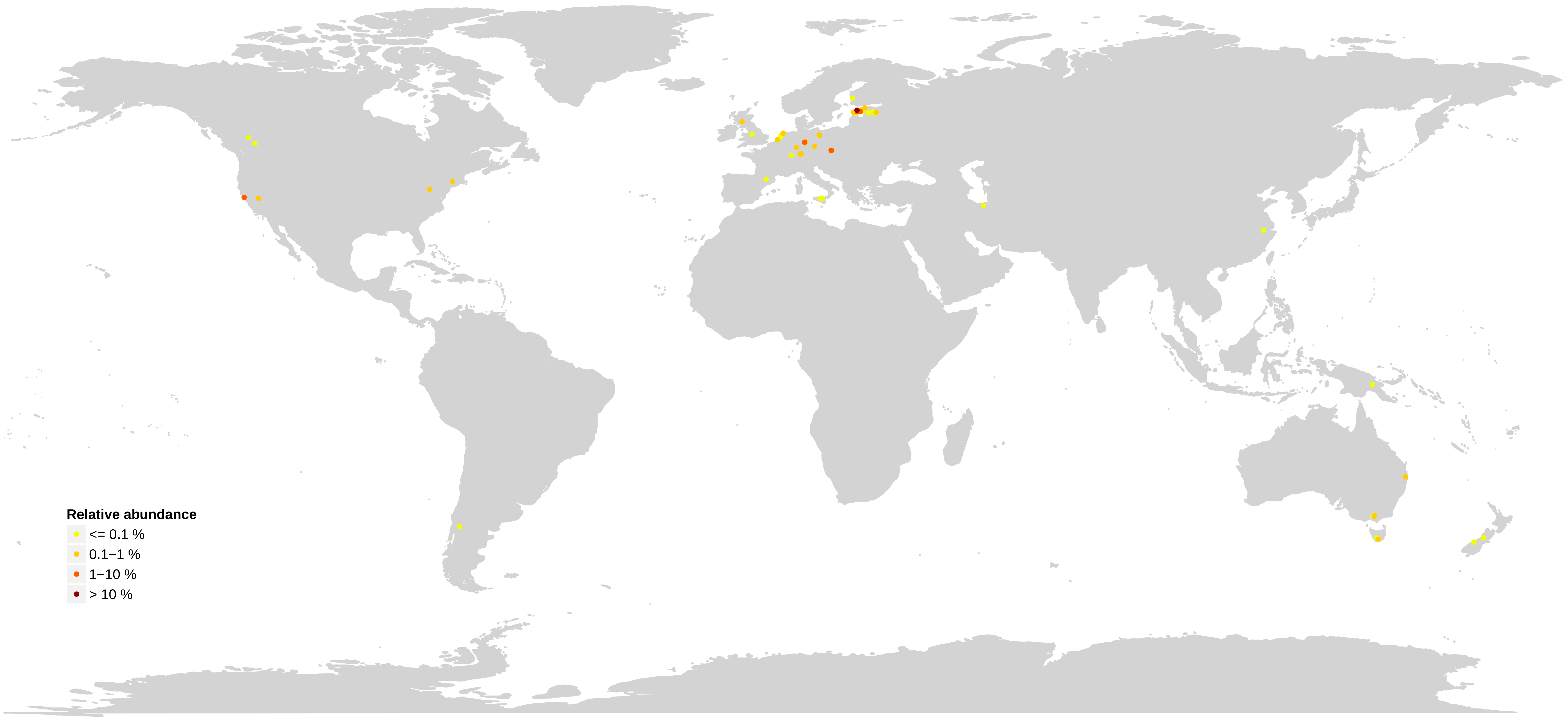

SH407693 Penicillium griseofulvum

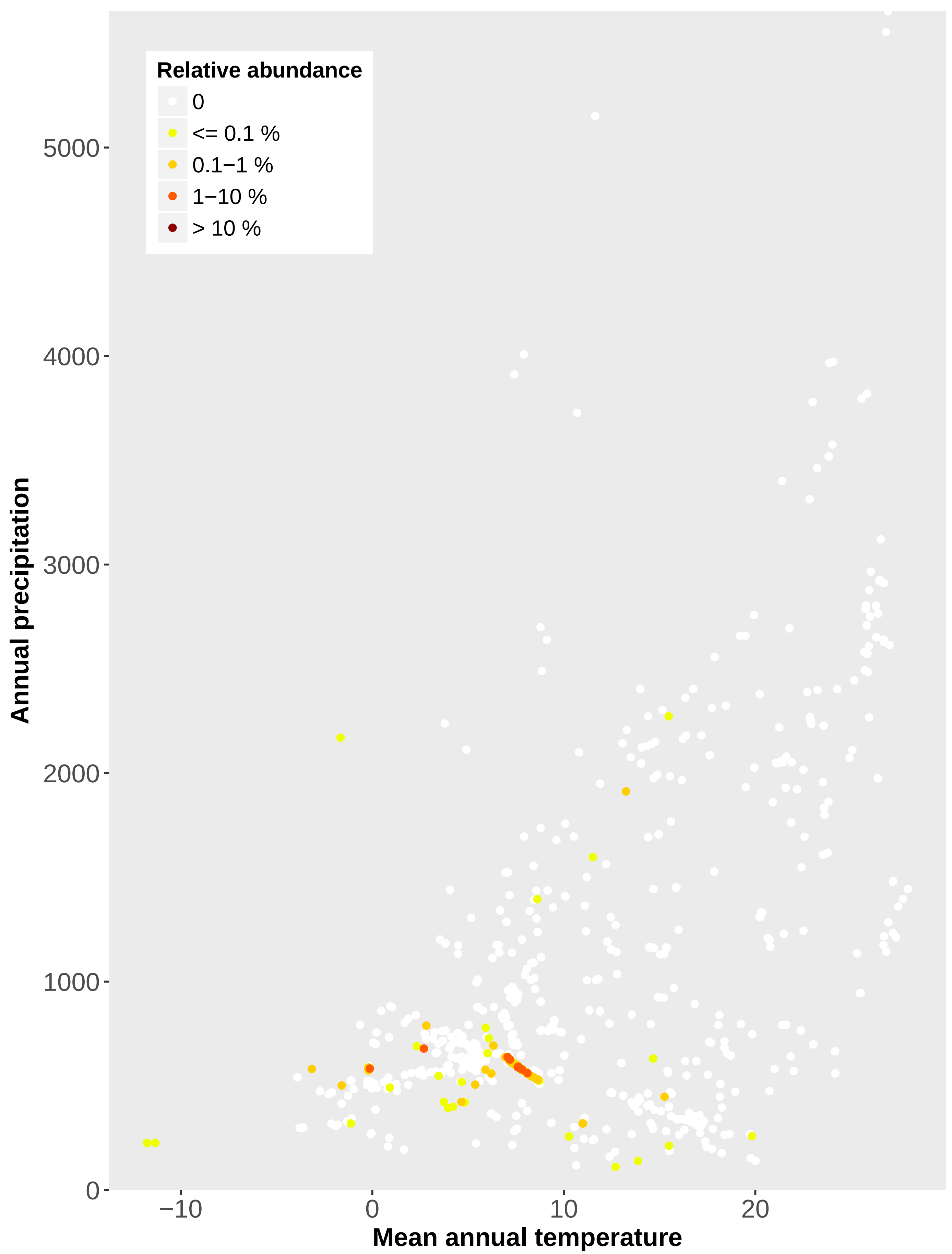

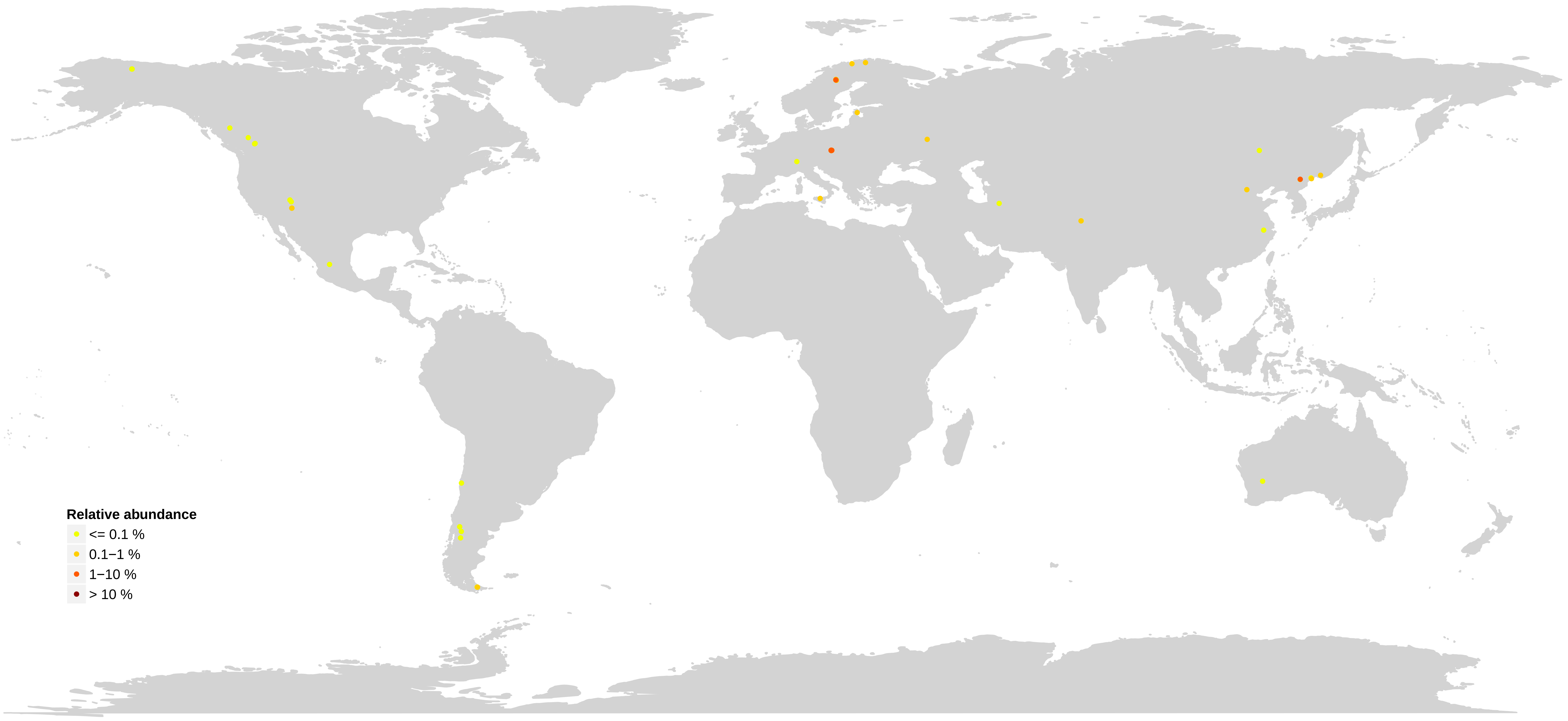

SH207075 Chaetothyriales sp

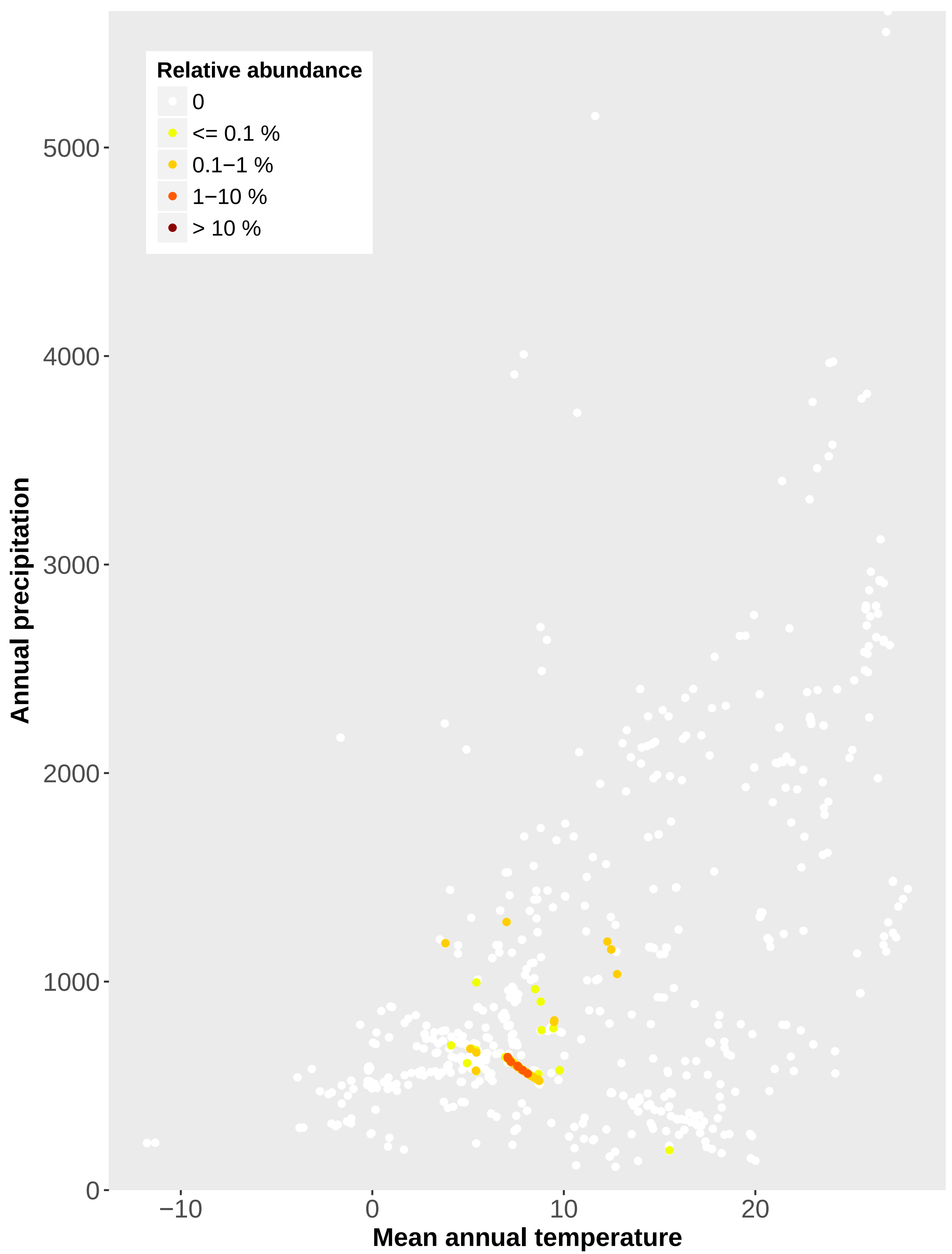

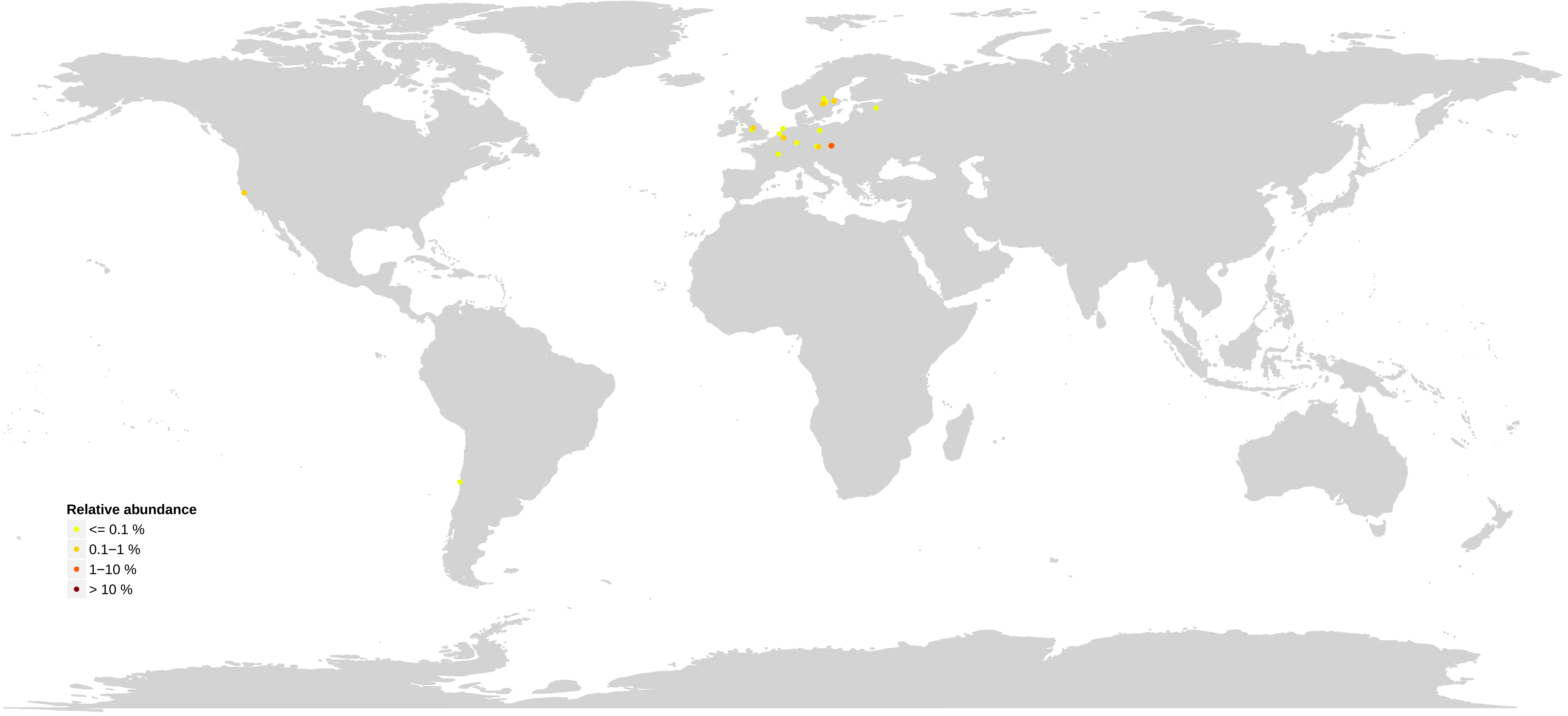

SH207620 Basidiomycota sp

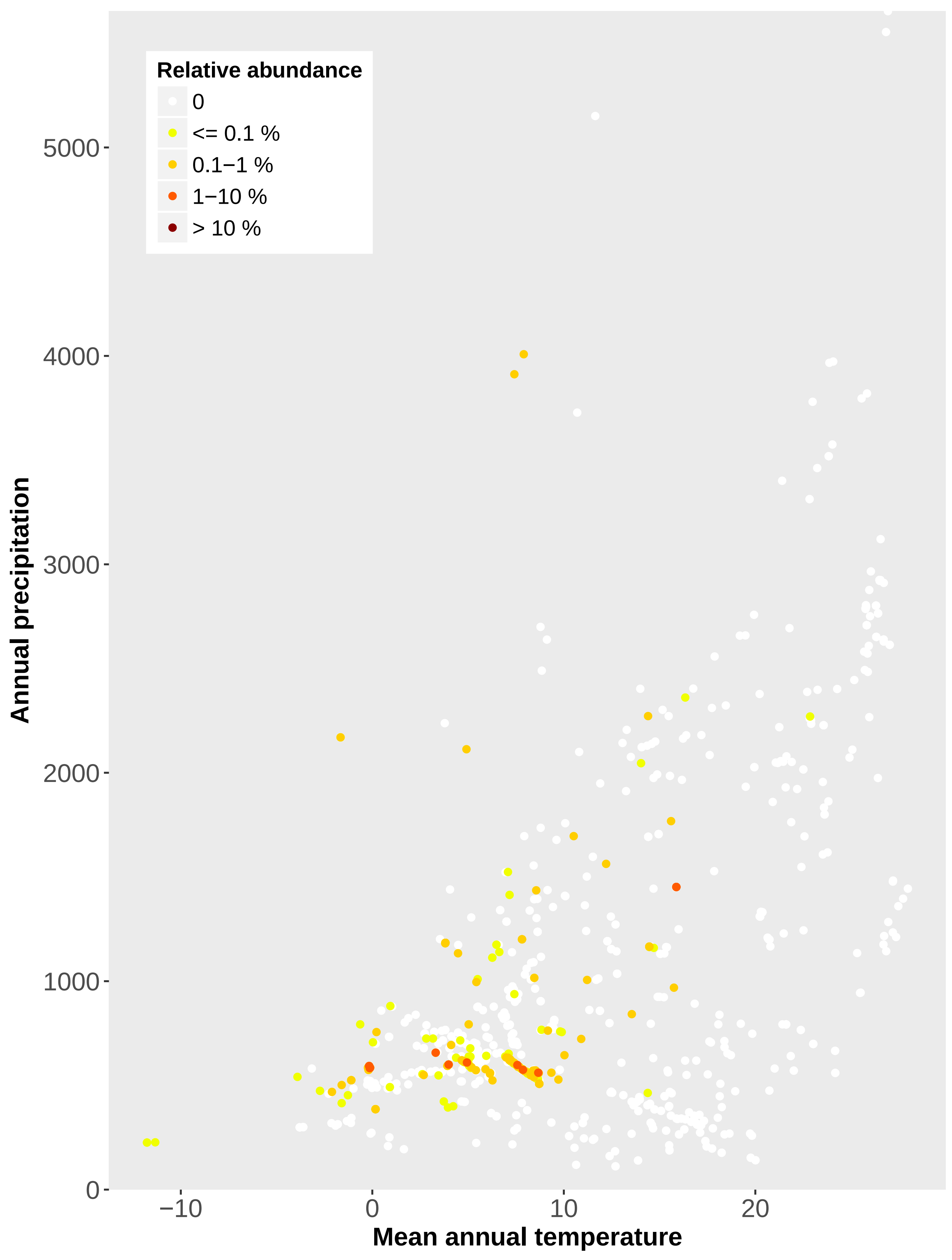

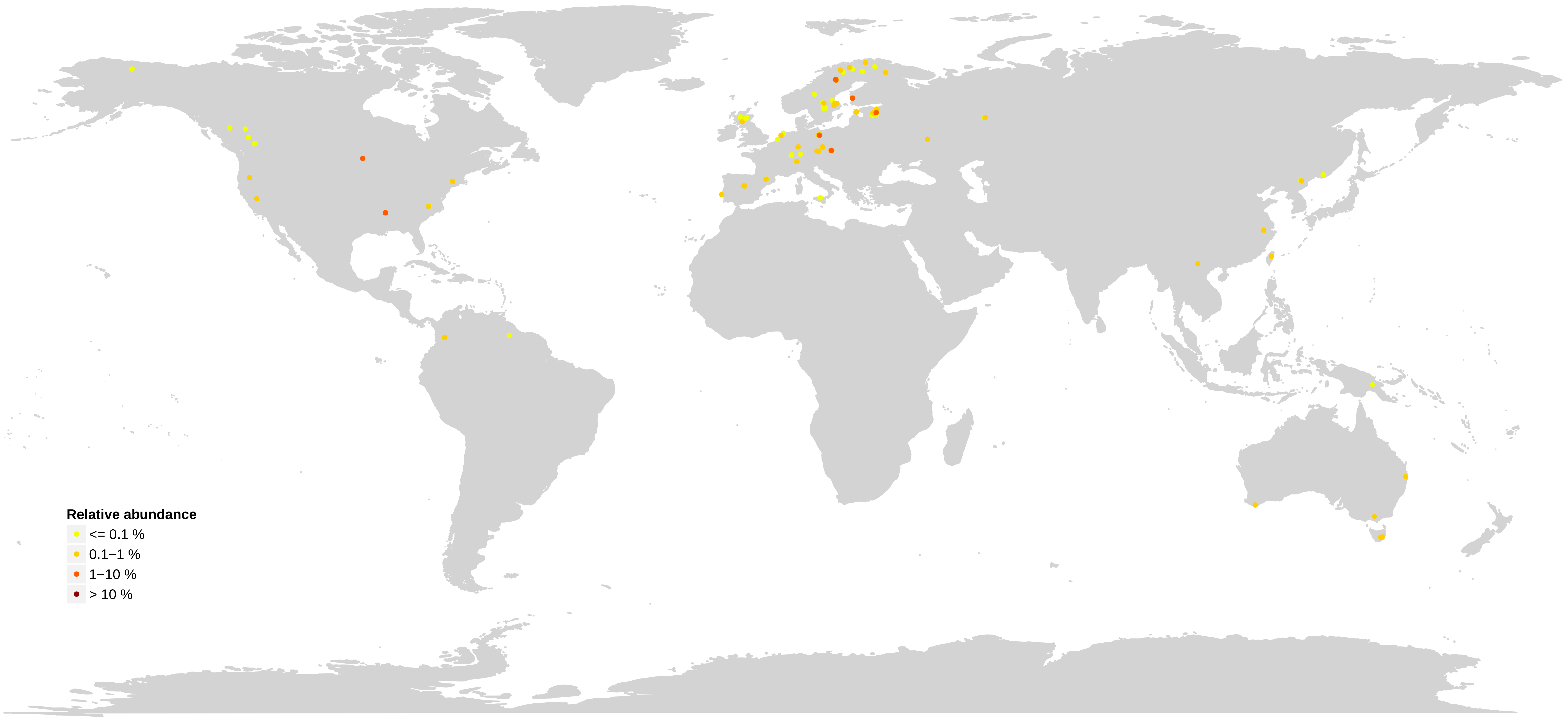

SH184926 Agaricales sp

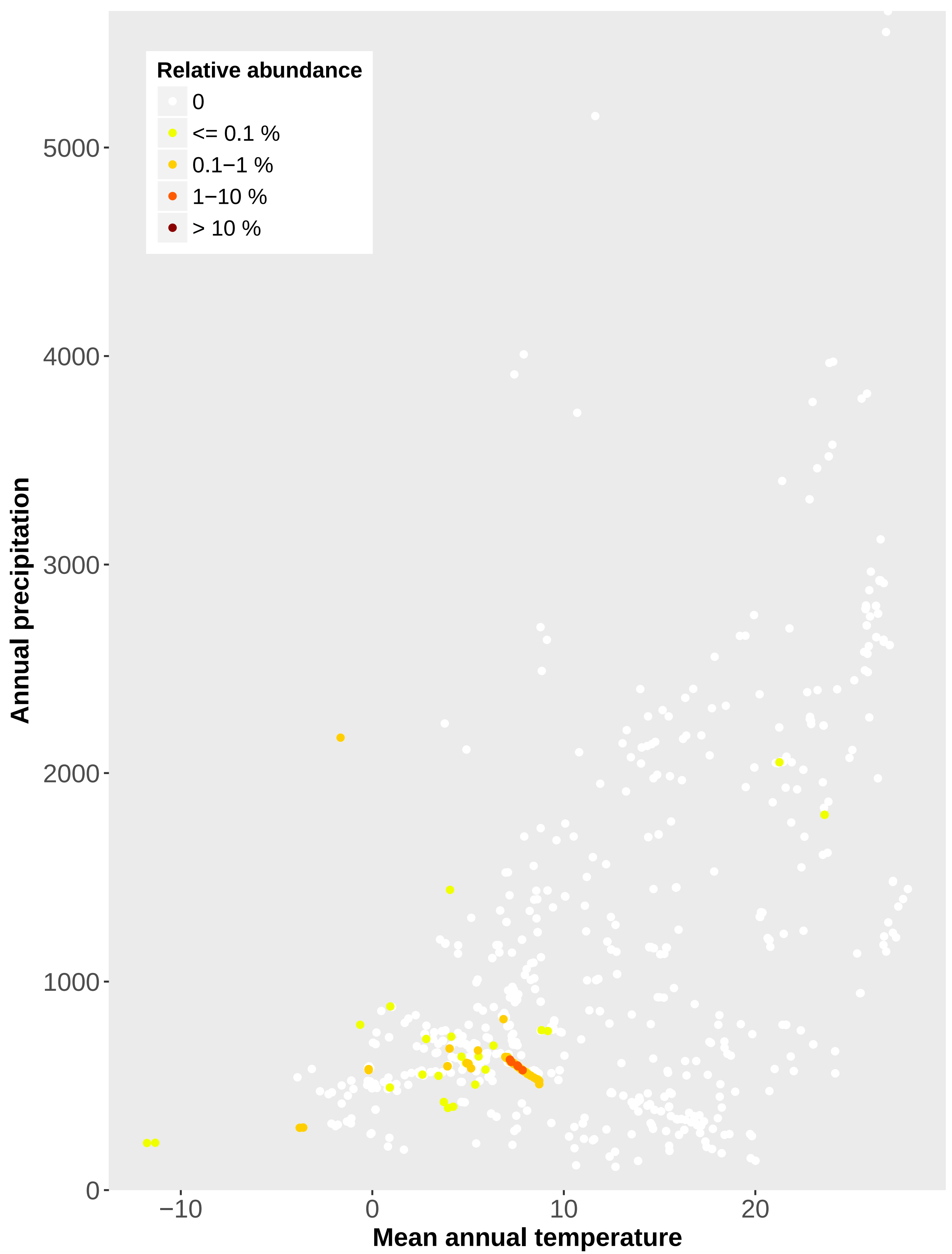

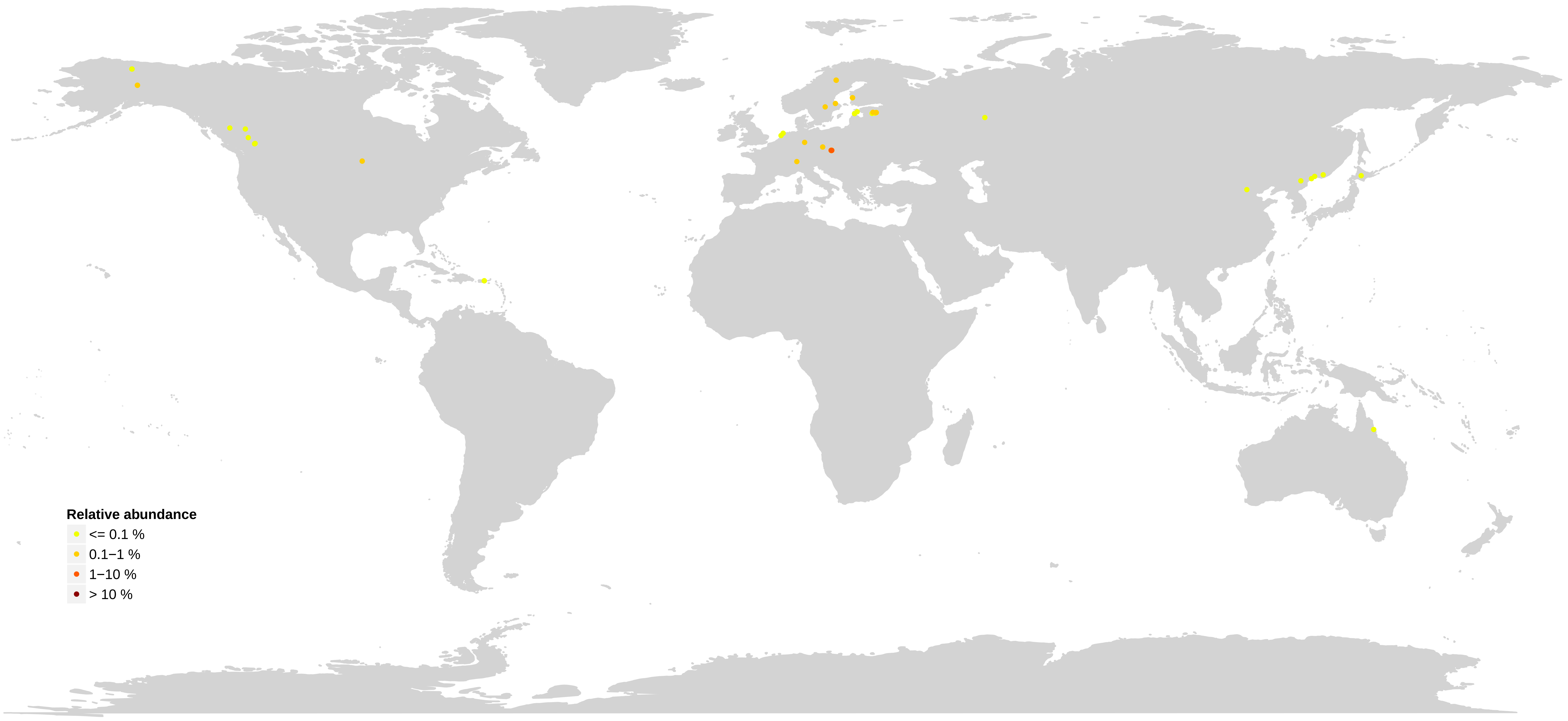

SH181342 Trichoderma viride

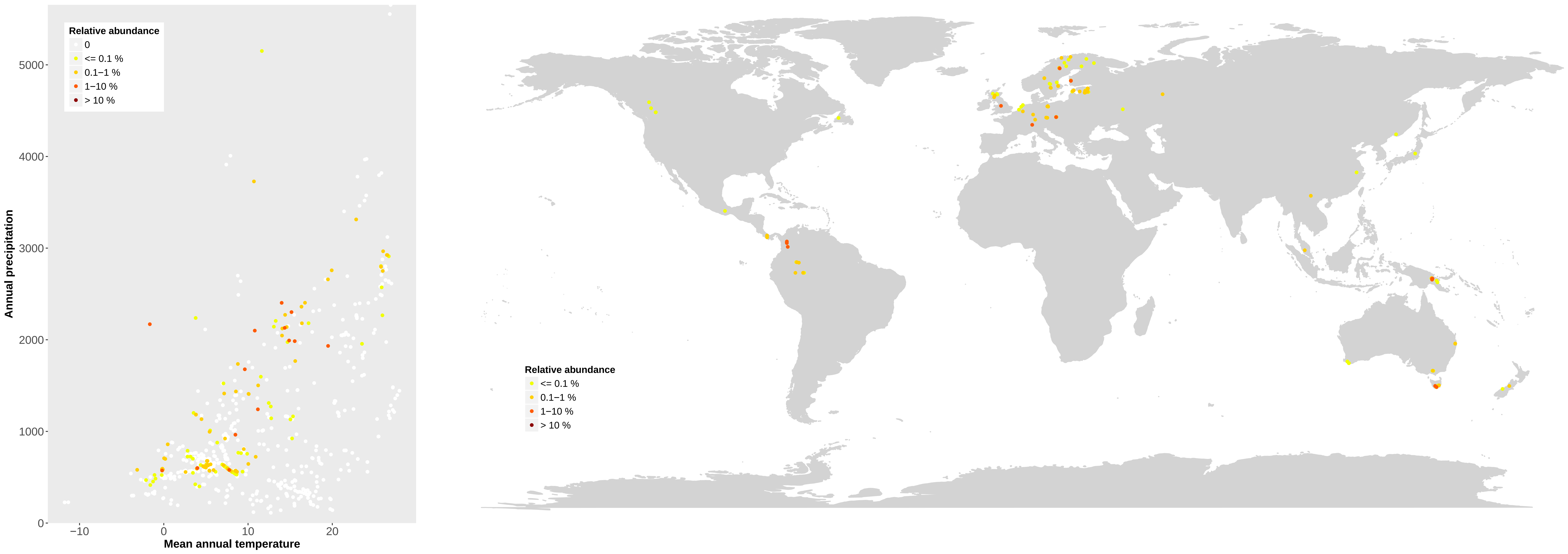

SH215110 Ascomycota sp

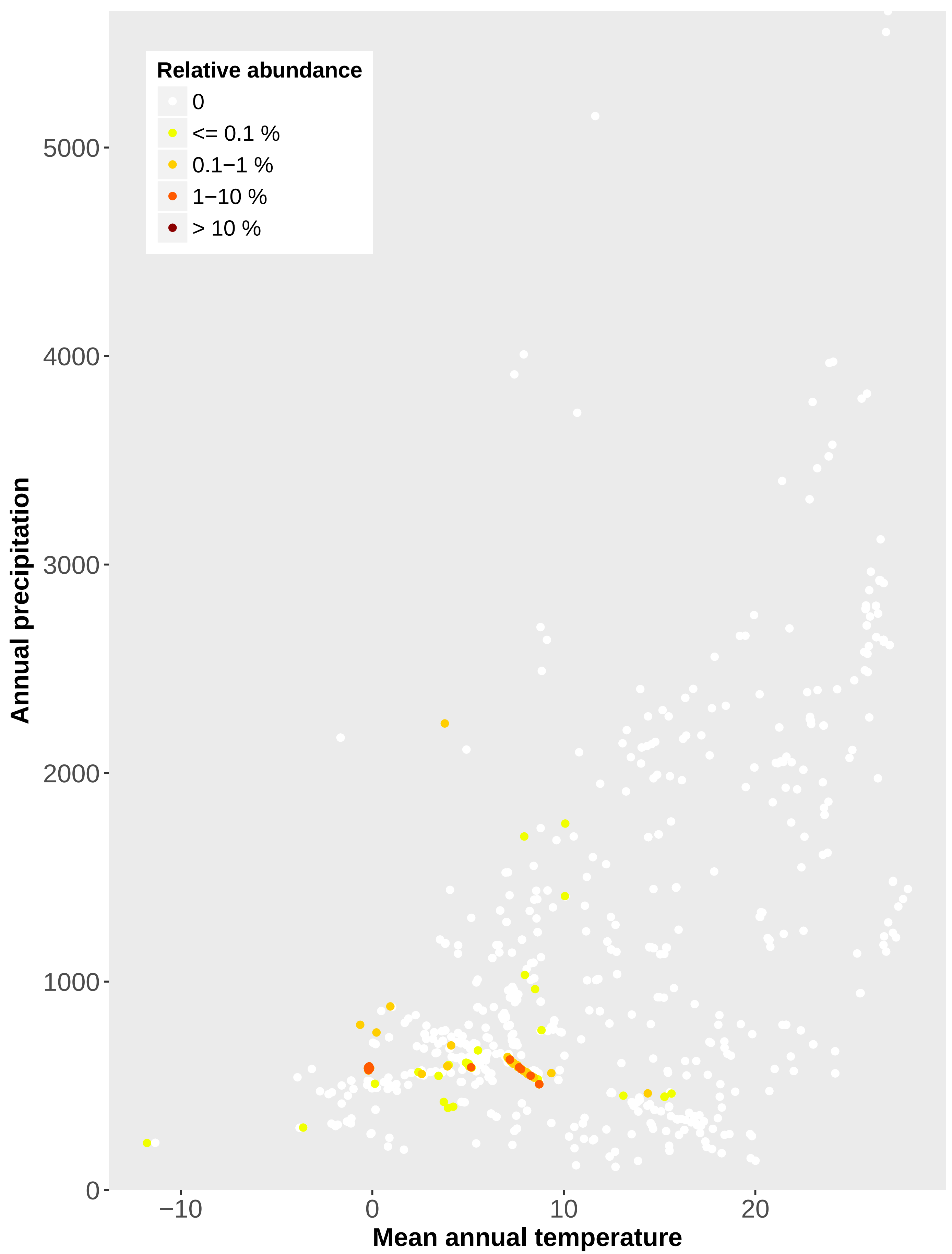

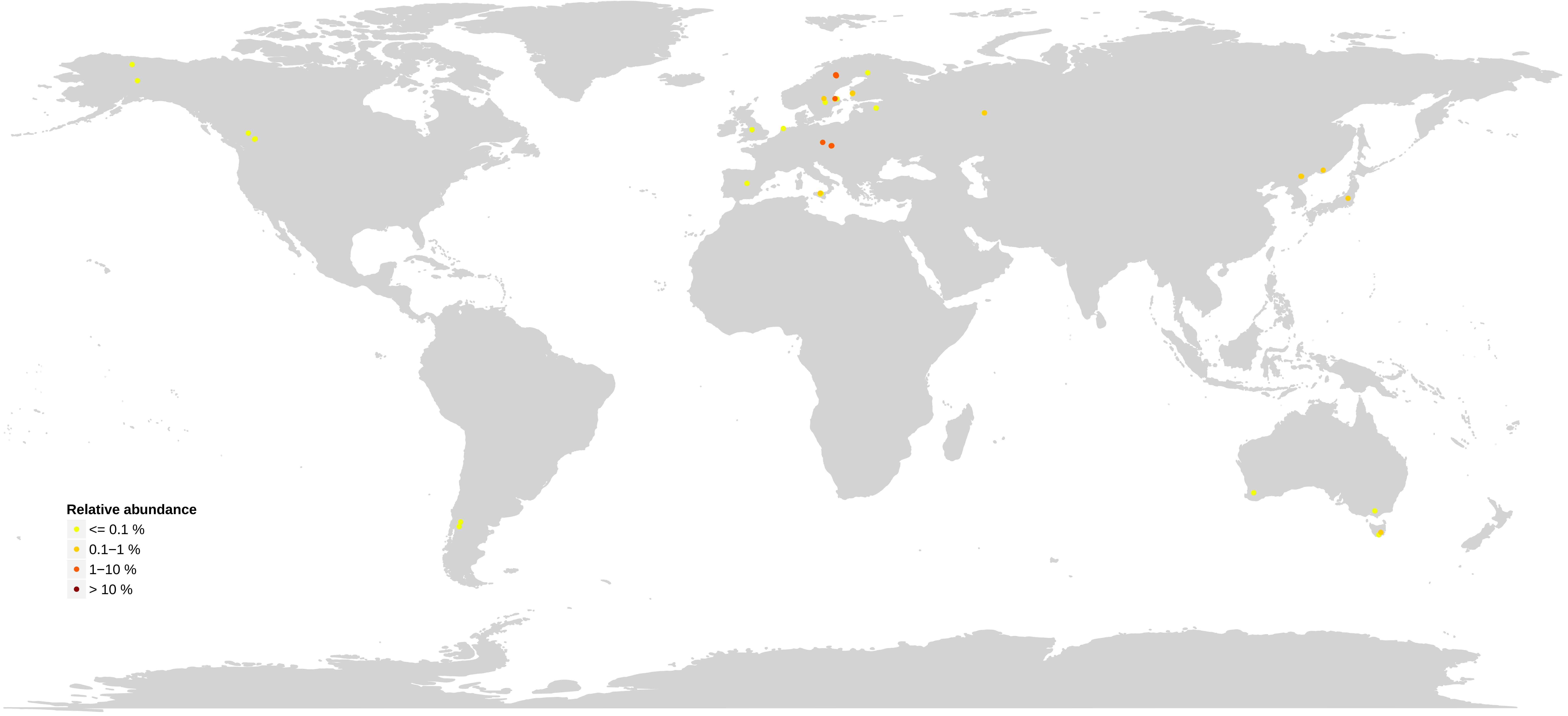

SH206392 Dothideomycetes sp

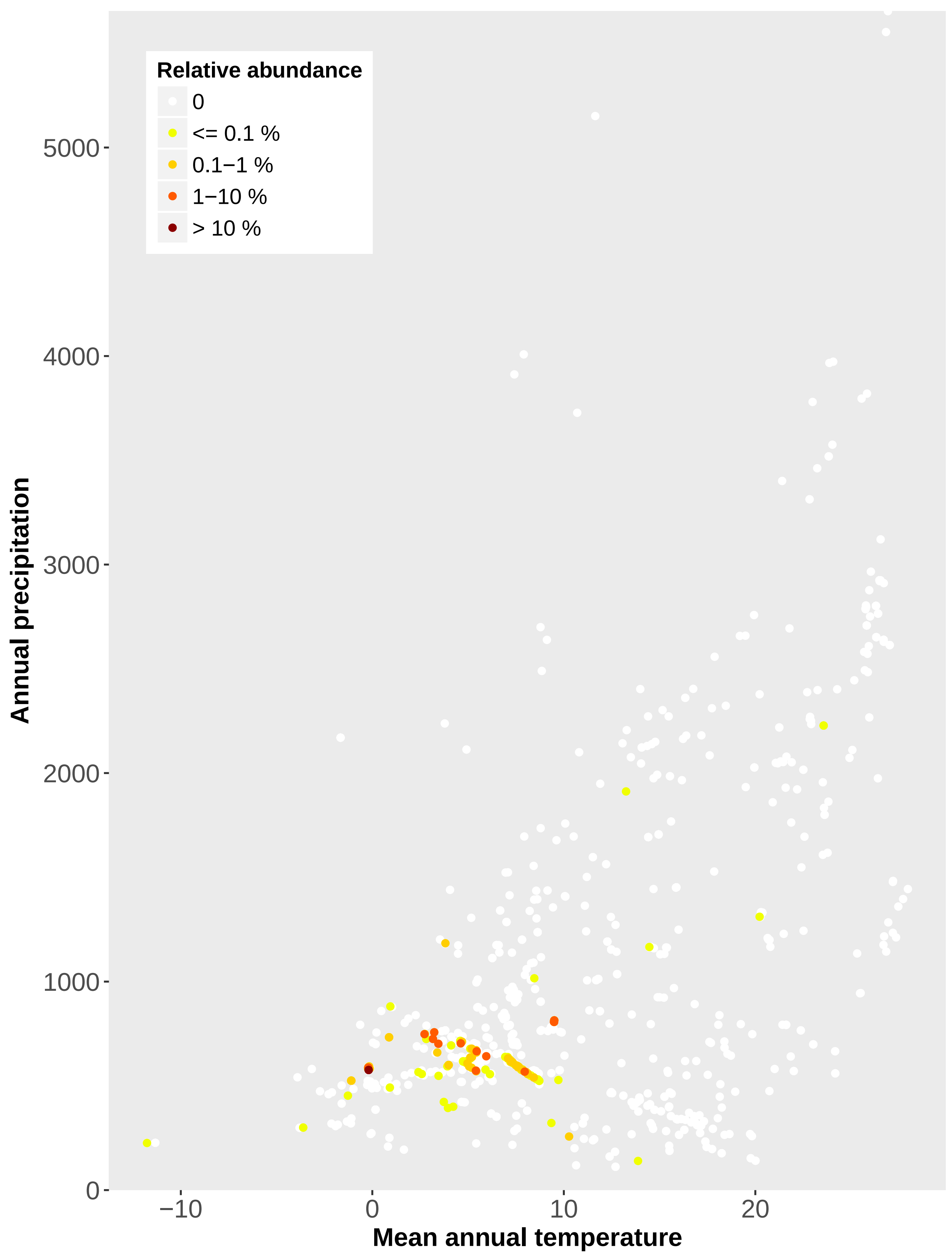

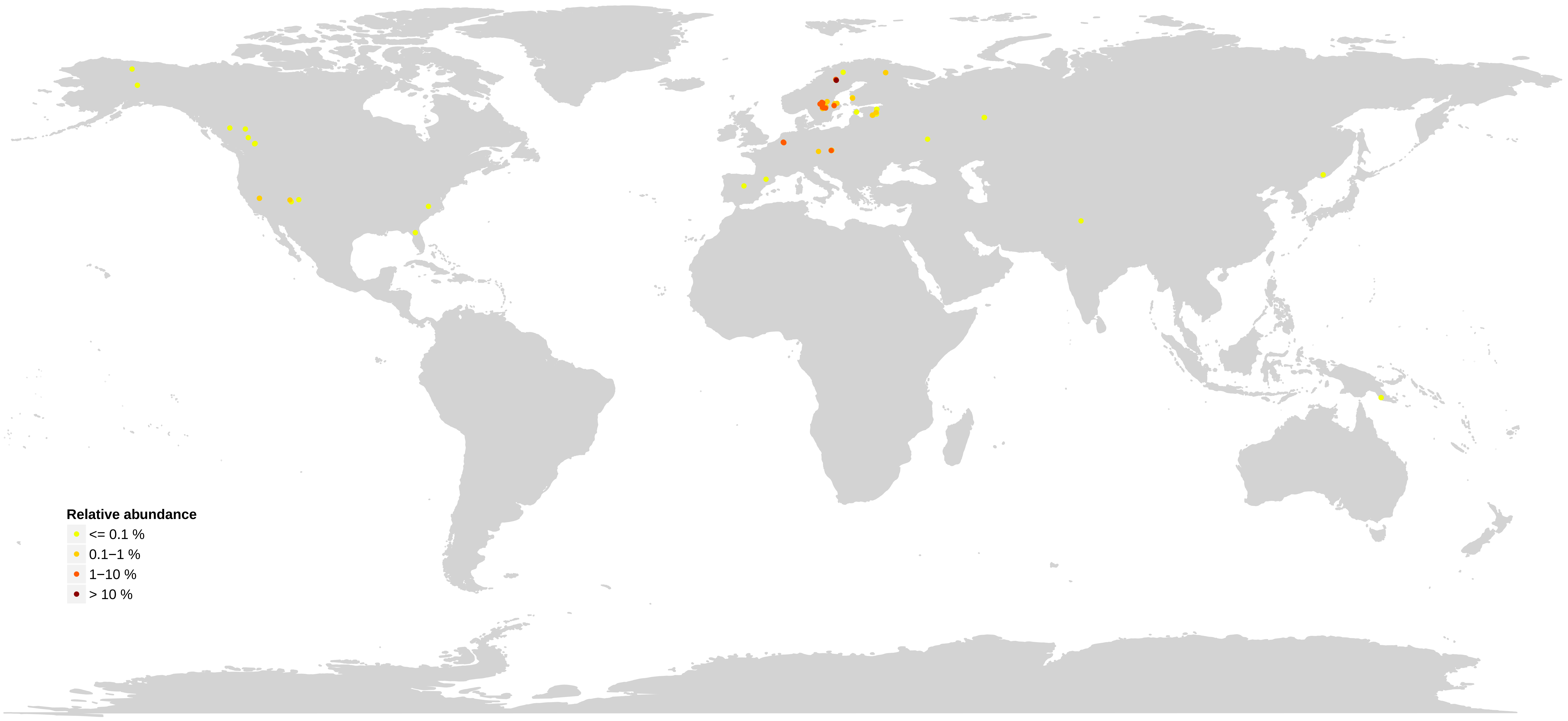

SH200451 Pleosporales sp

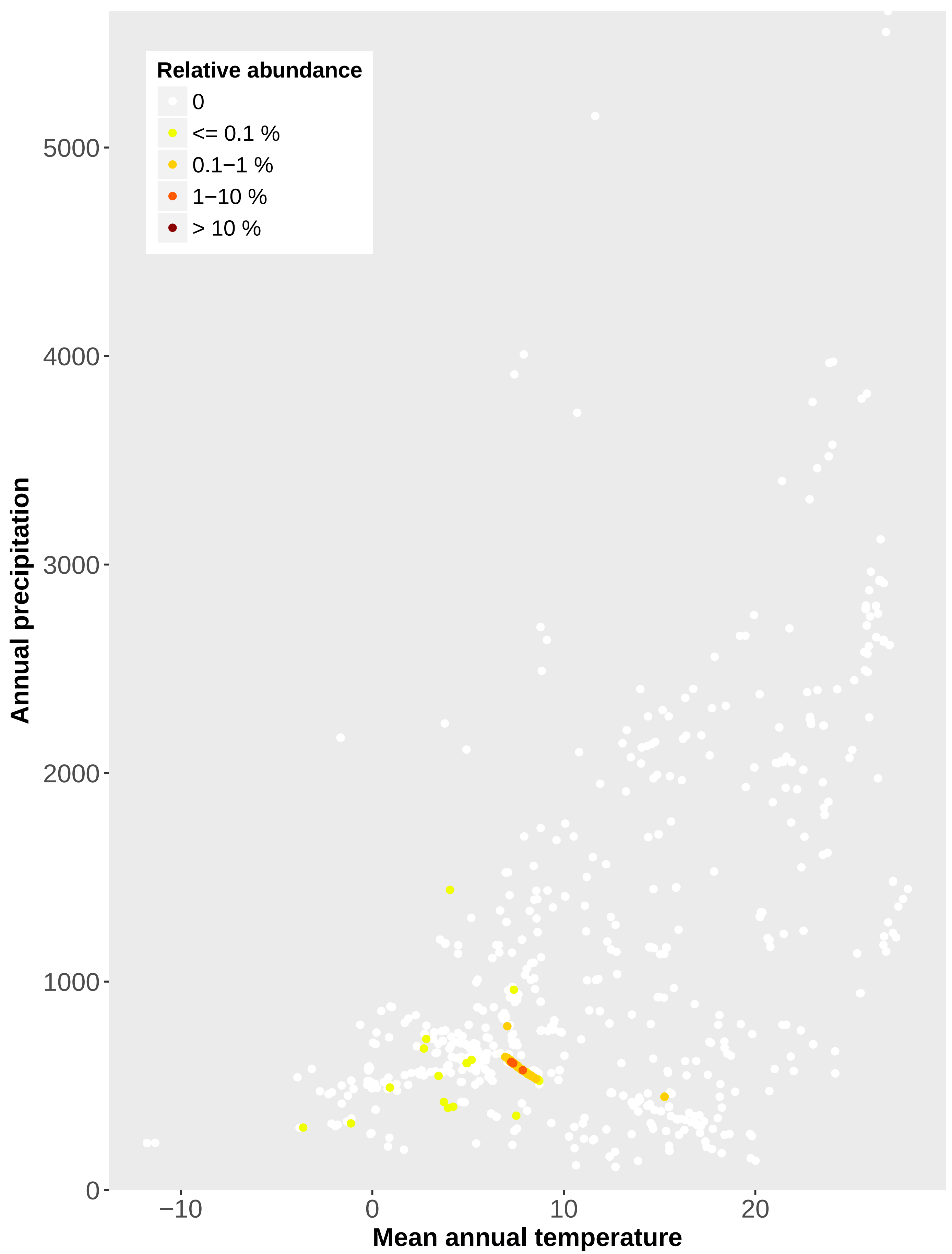

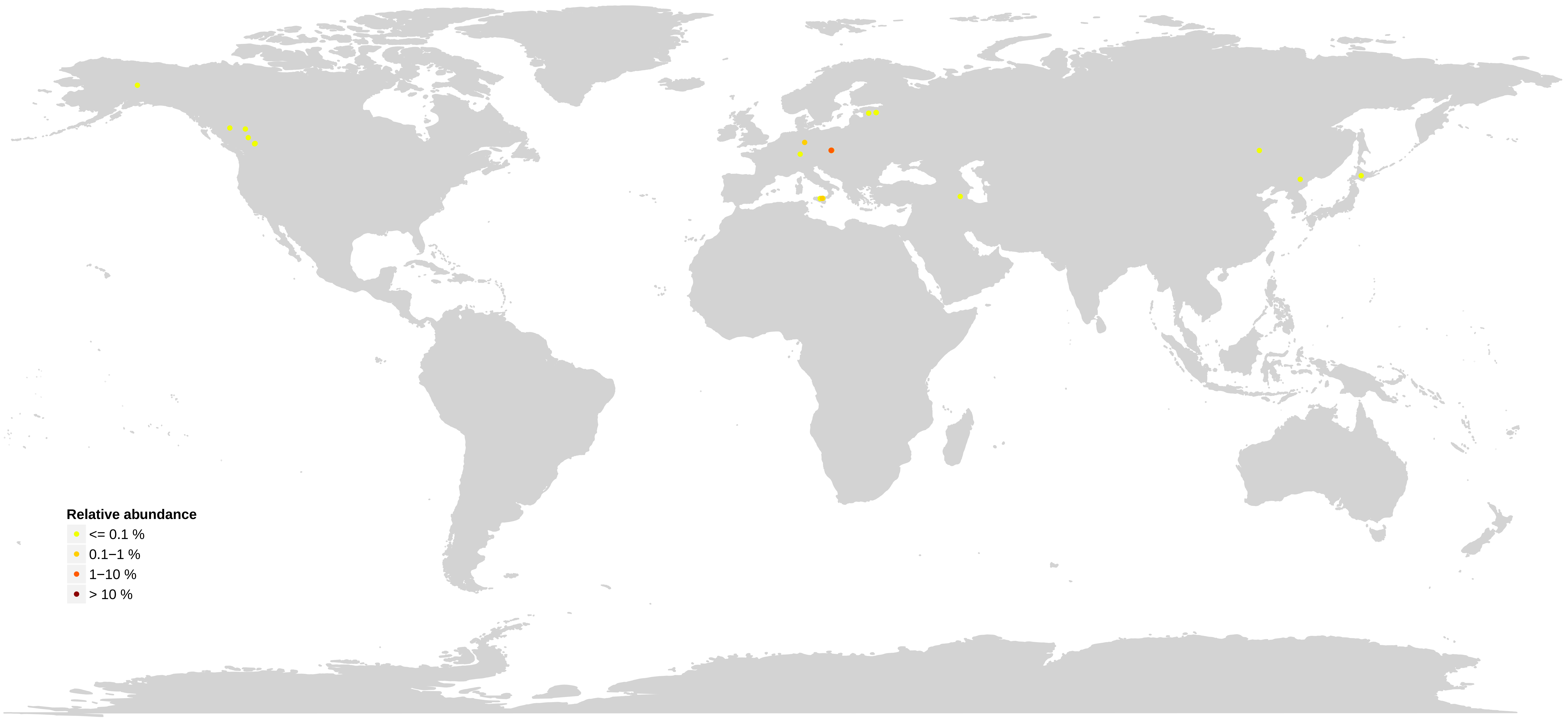

SH019336 Ilyonectria destructans

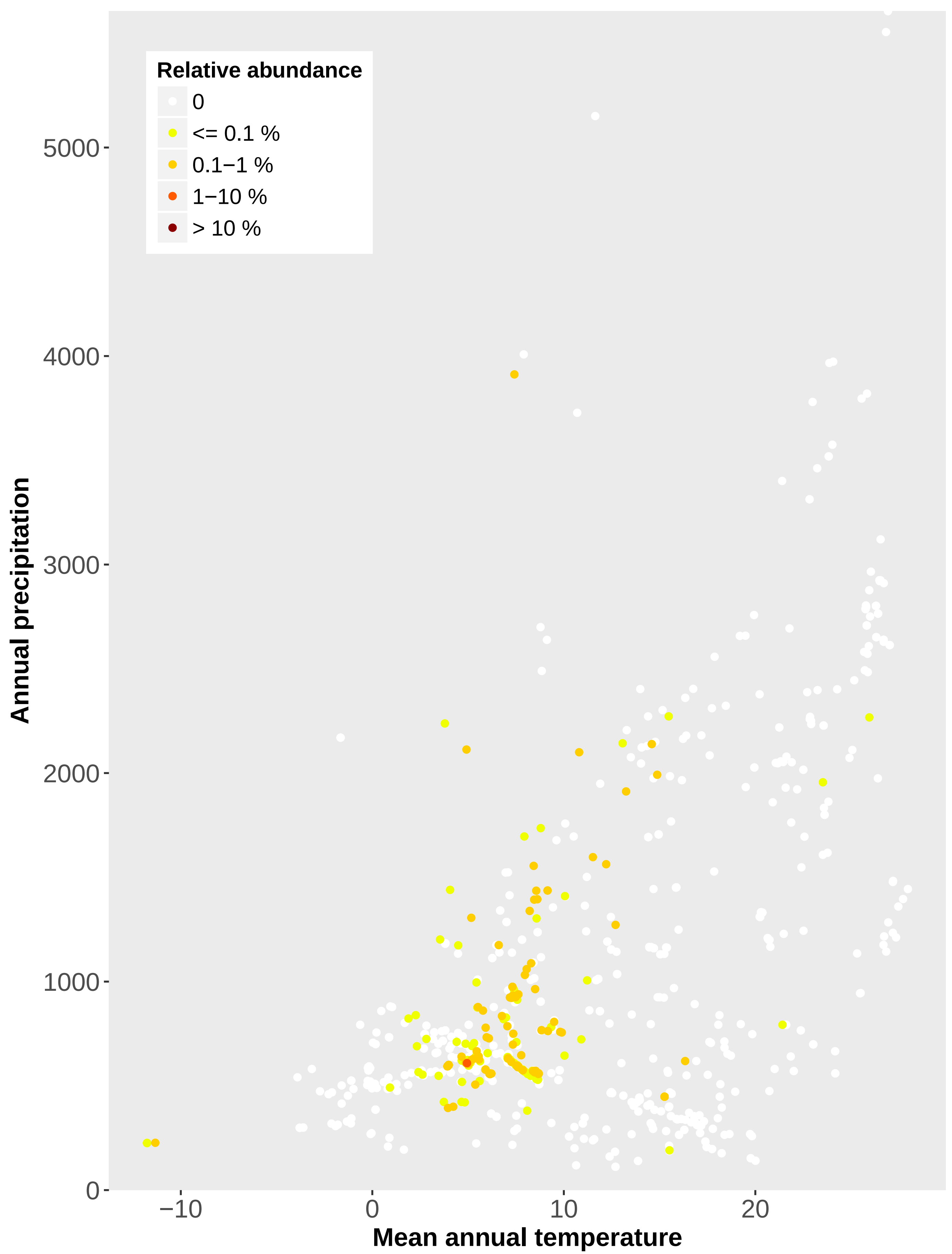

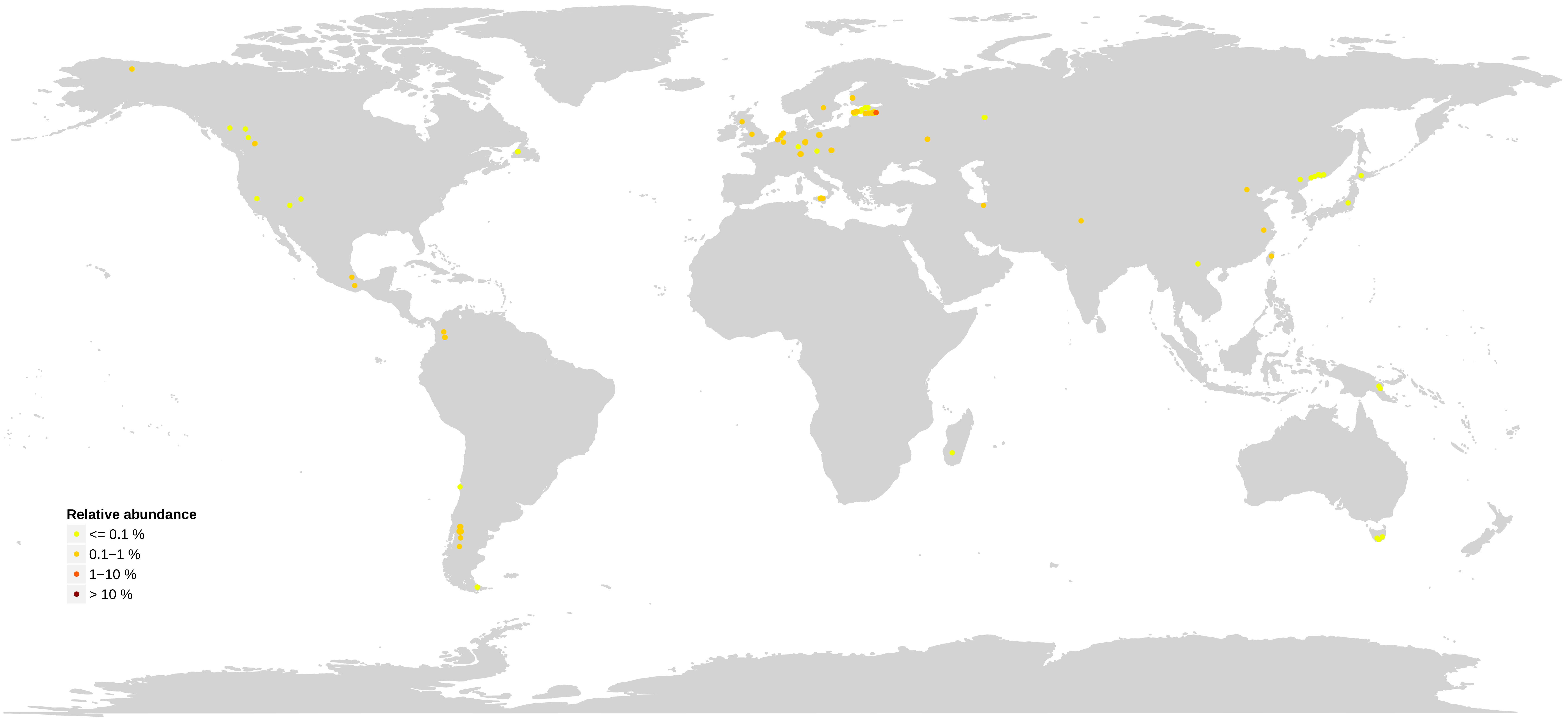

SH216097 Umbelopsis sp

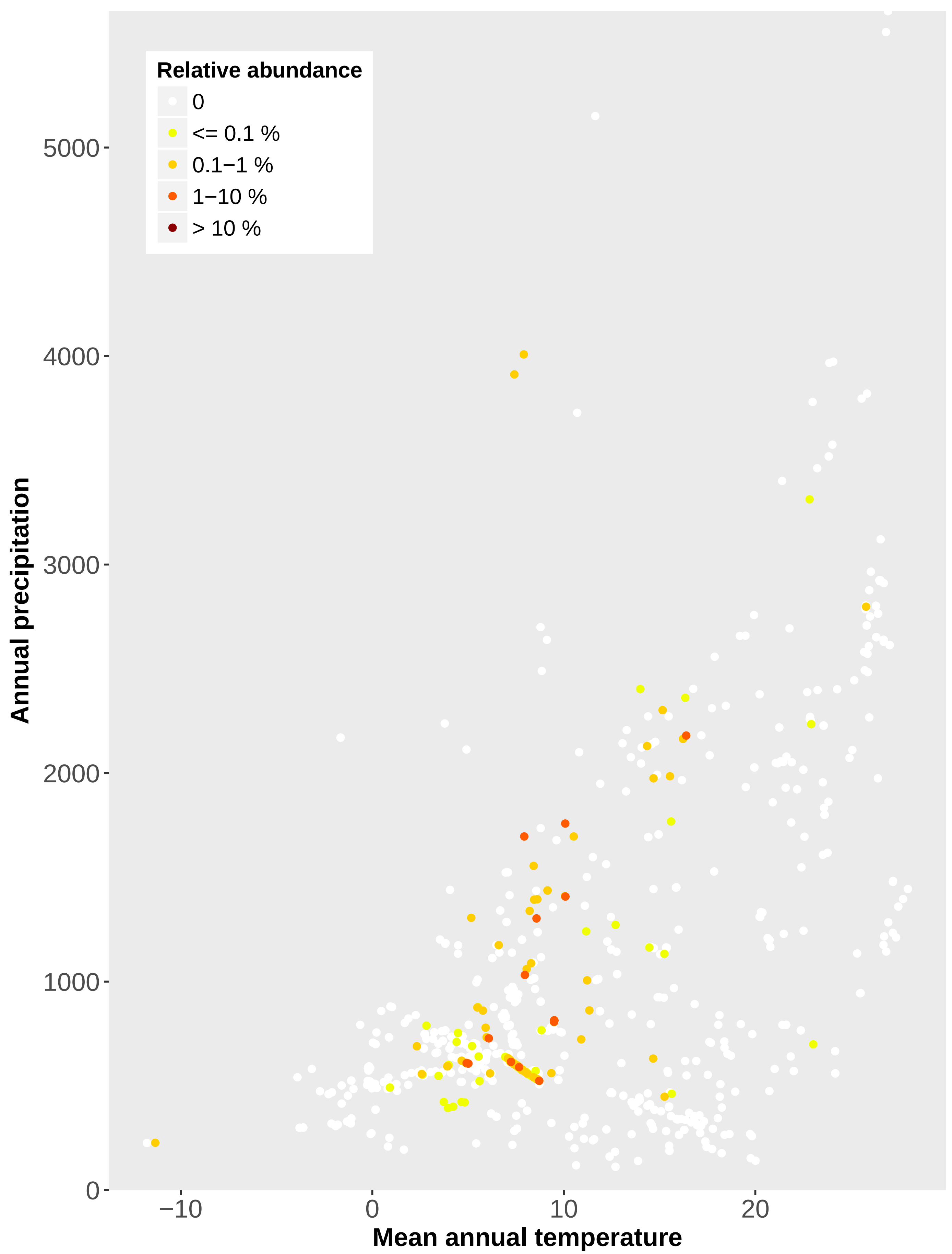

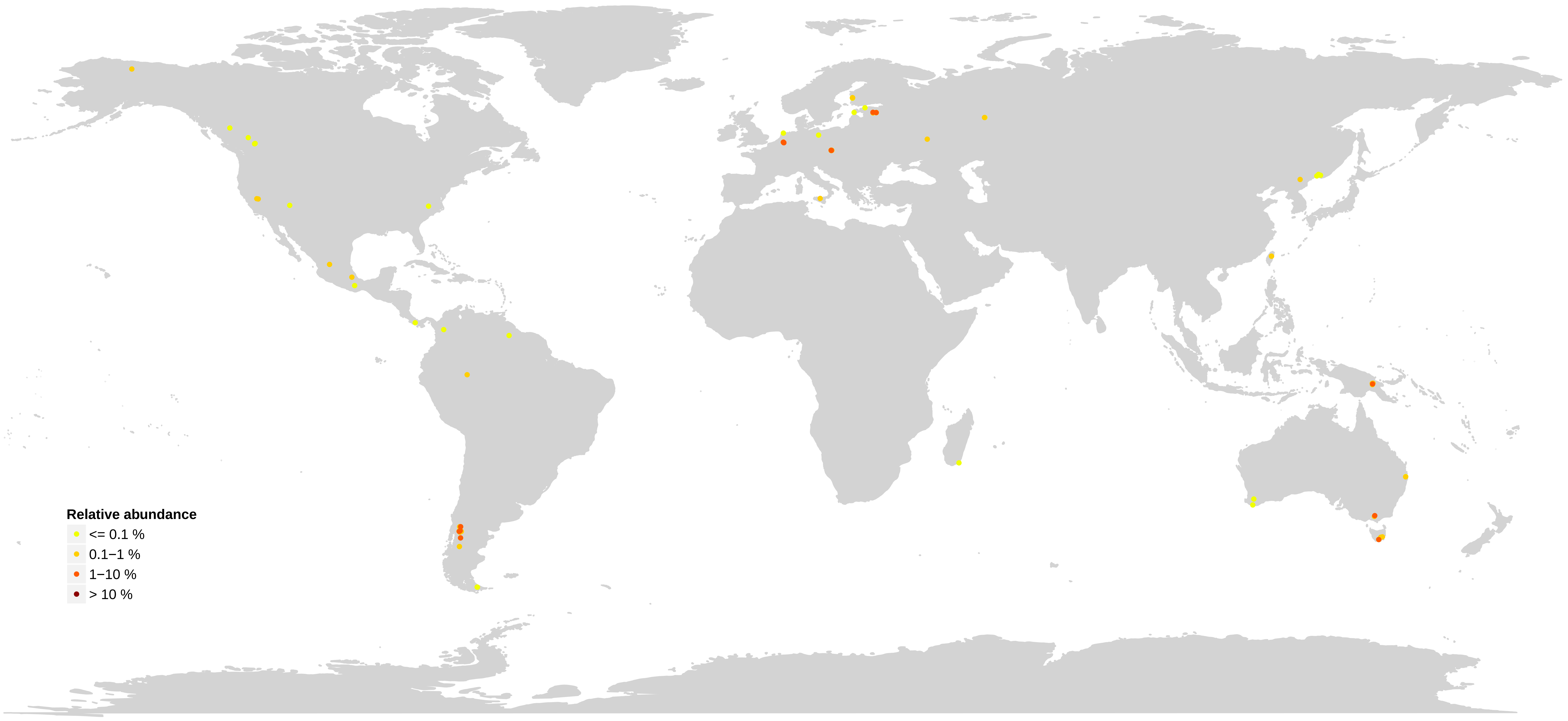

SH184964 Tolypocladium cylindrosporum

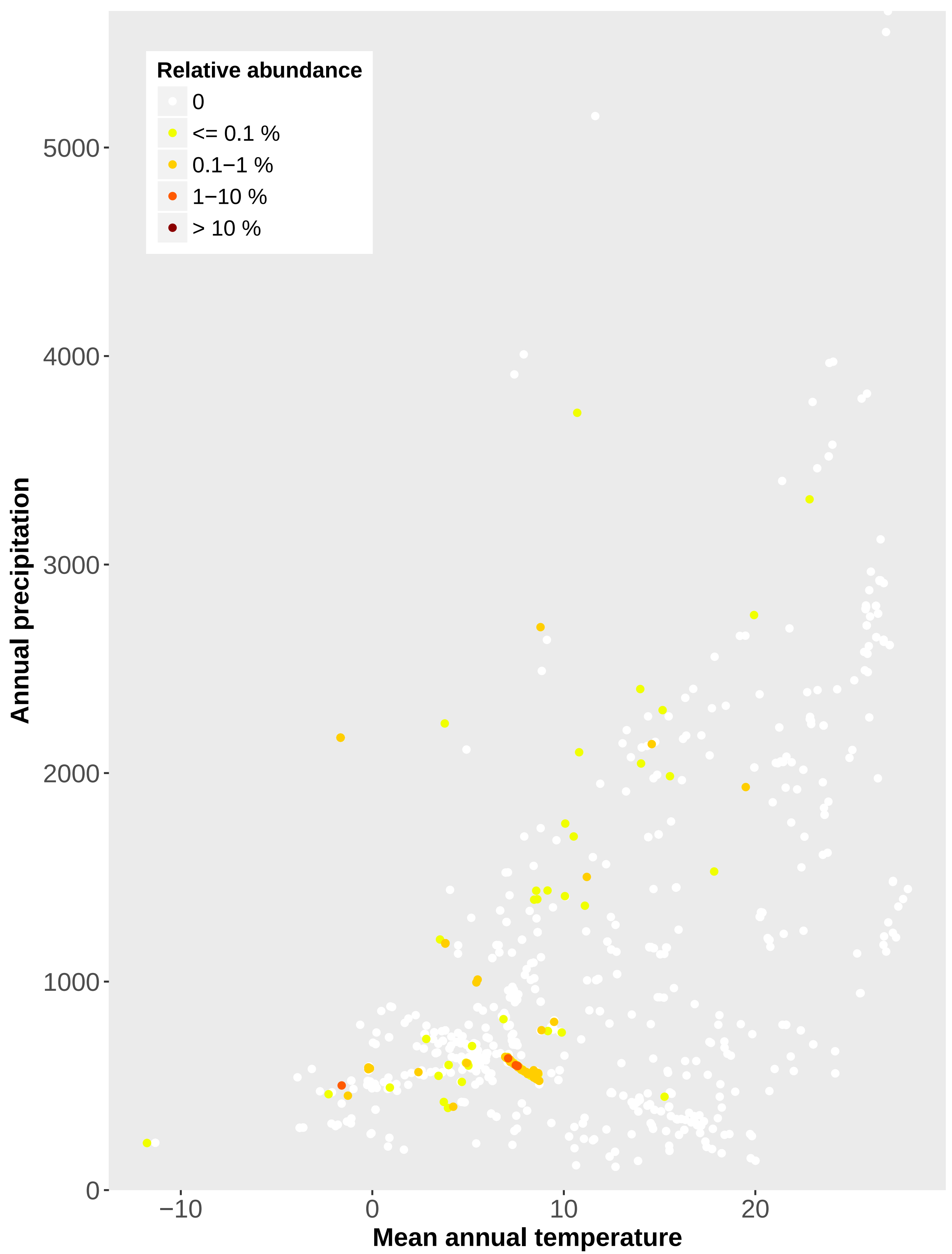

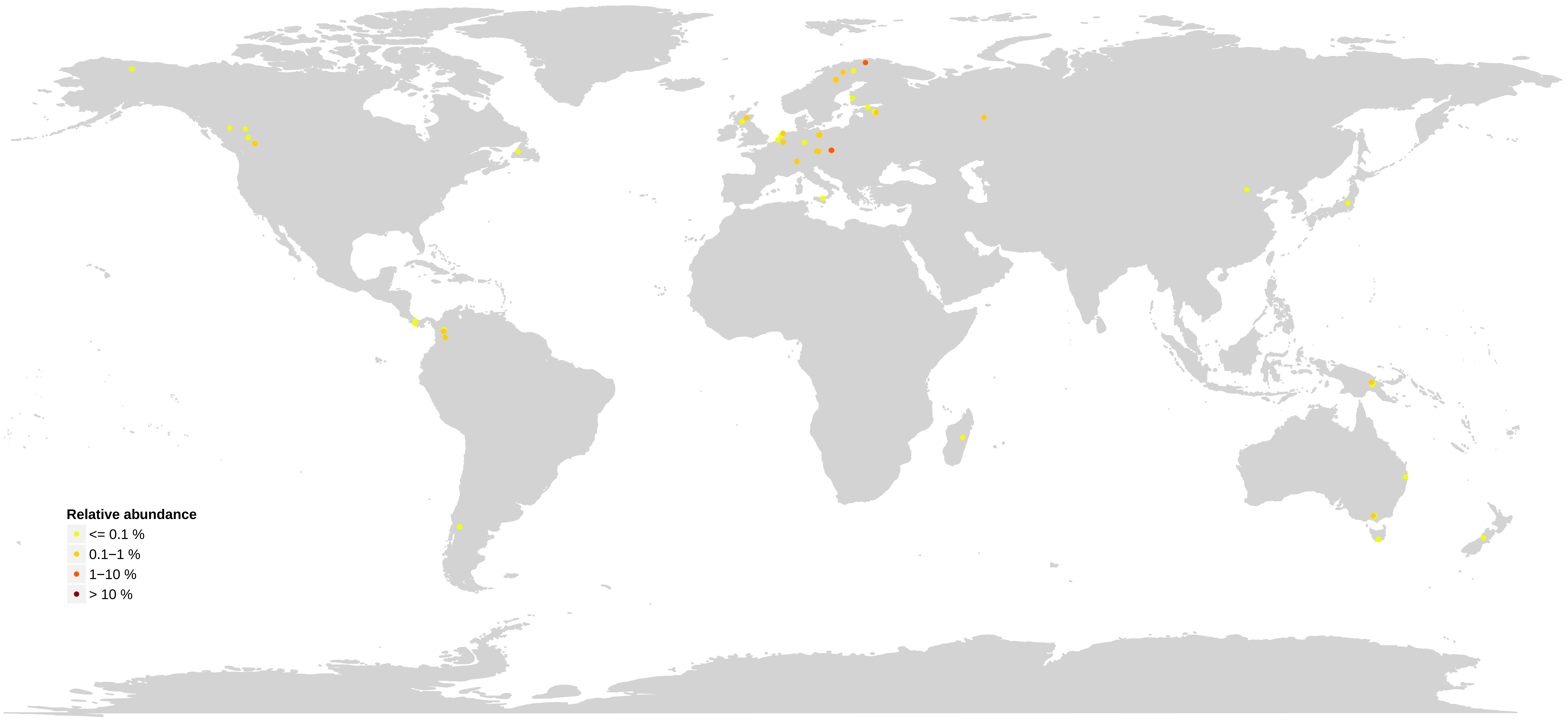

SH015851 Fungi sp

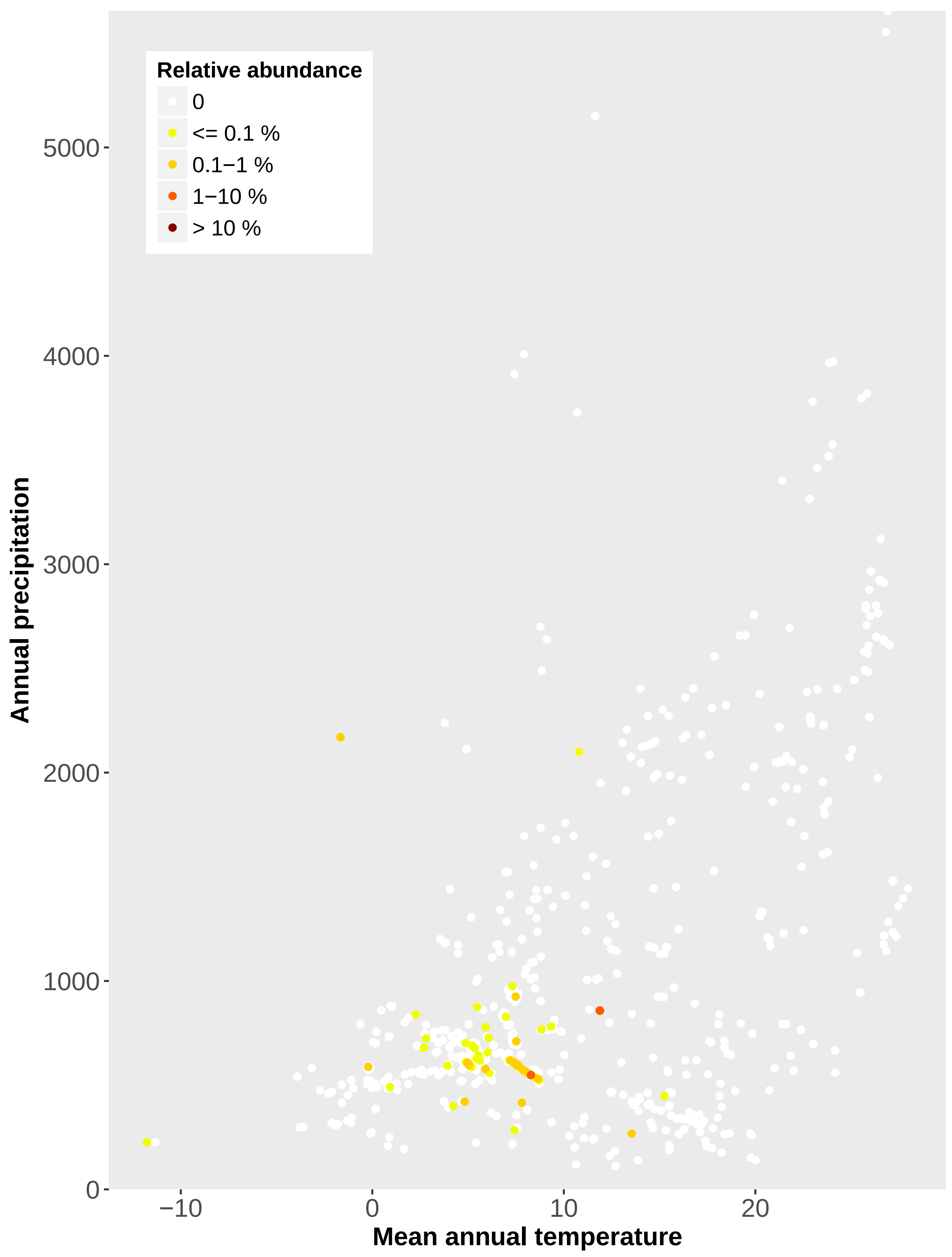

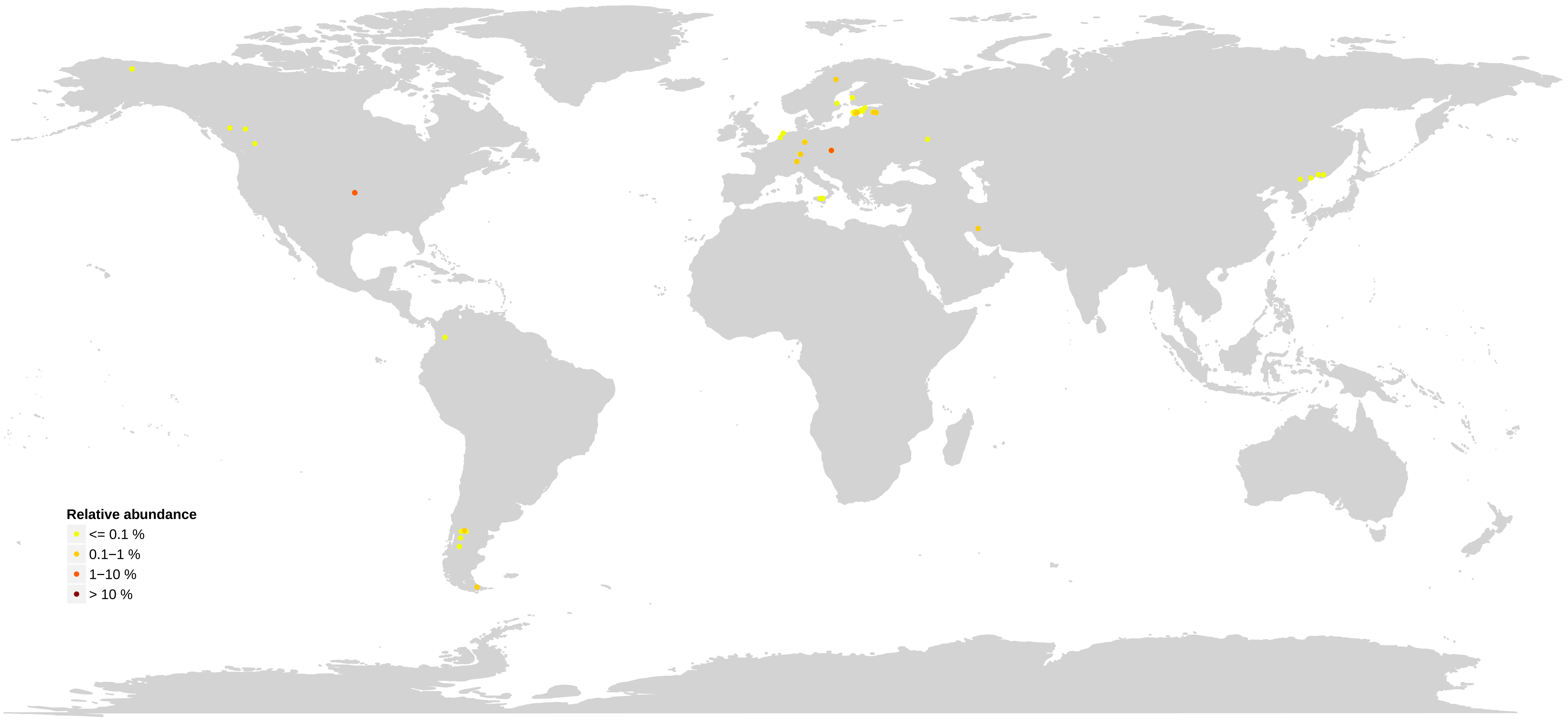

SH192574 Pochonia bulbillosa

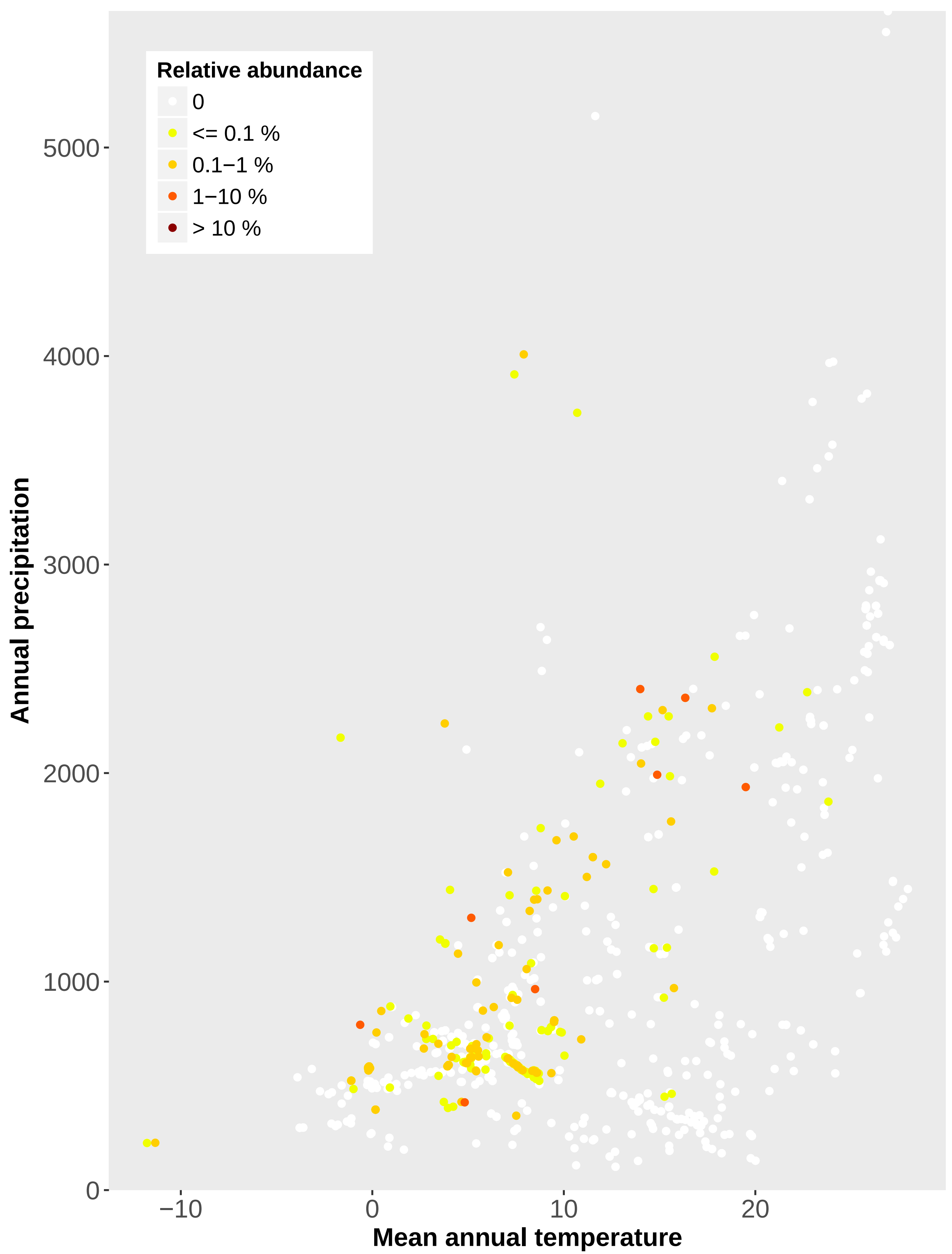

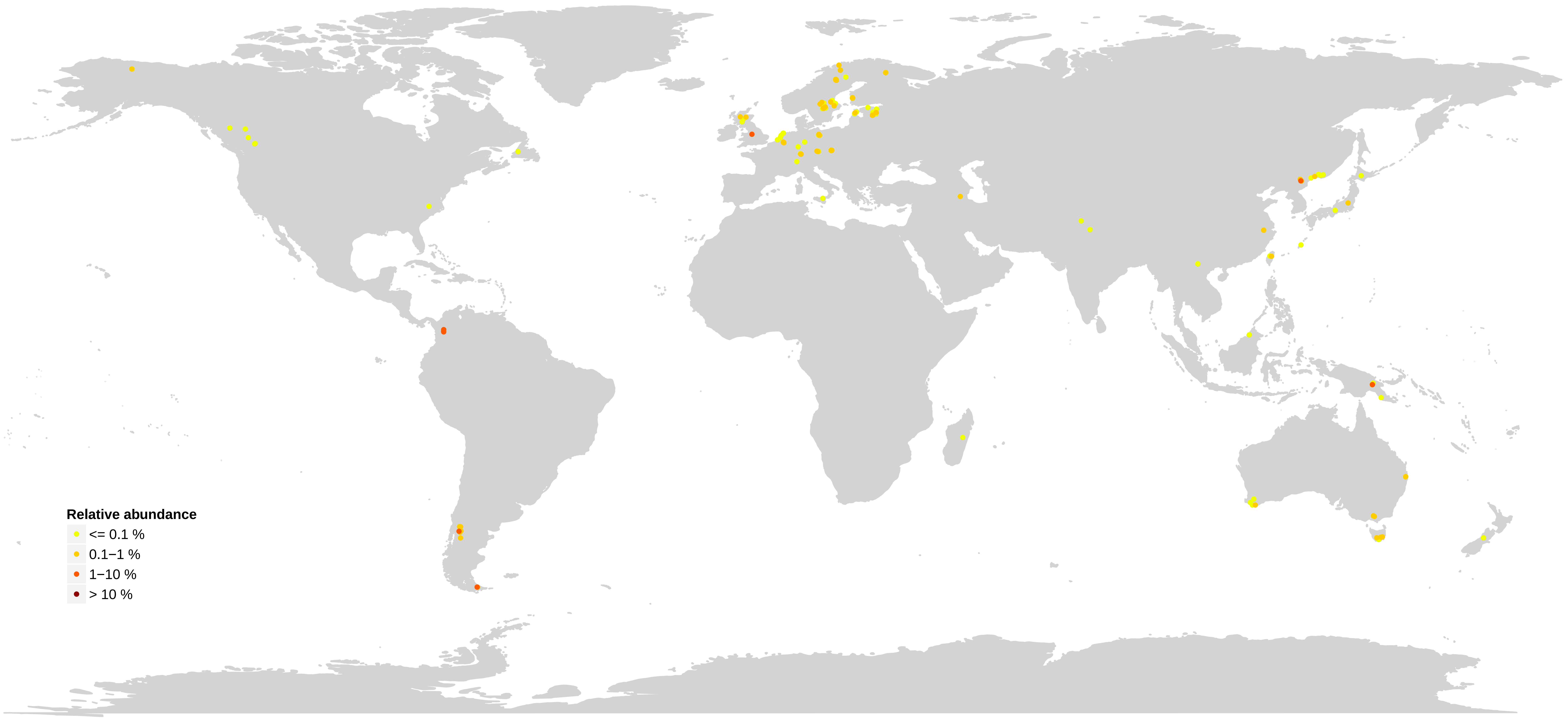

SH181579 Chaetosphaeriaceae sp

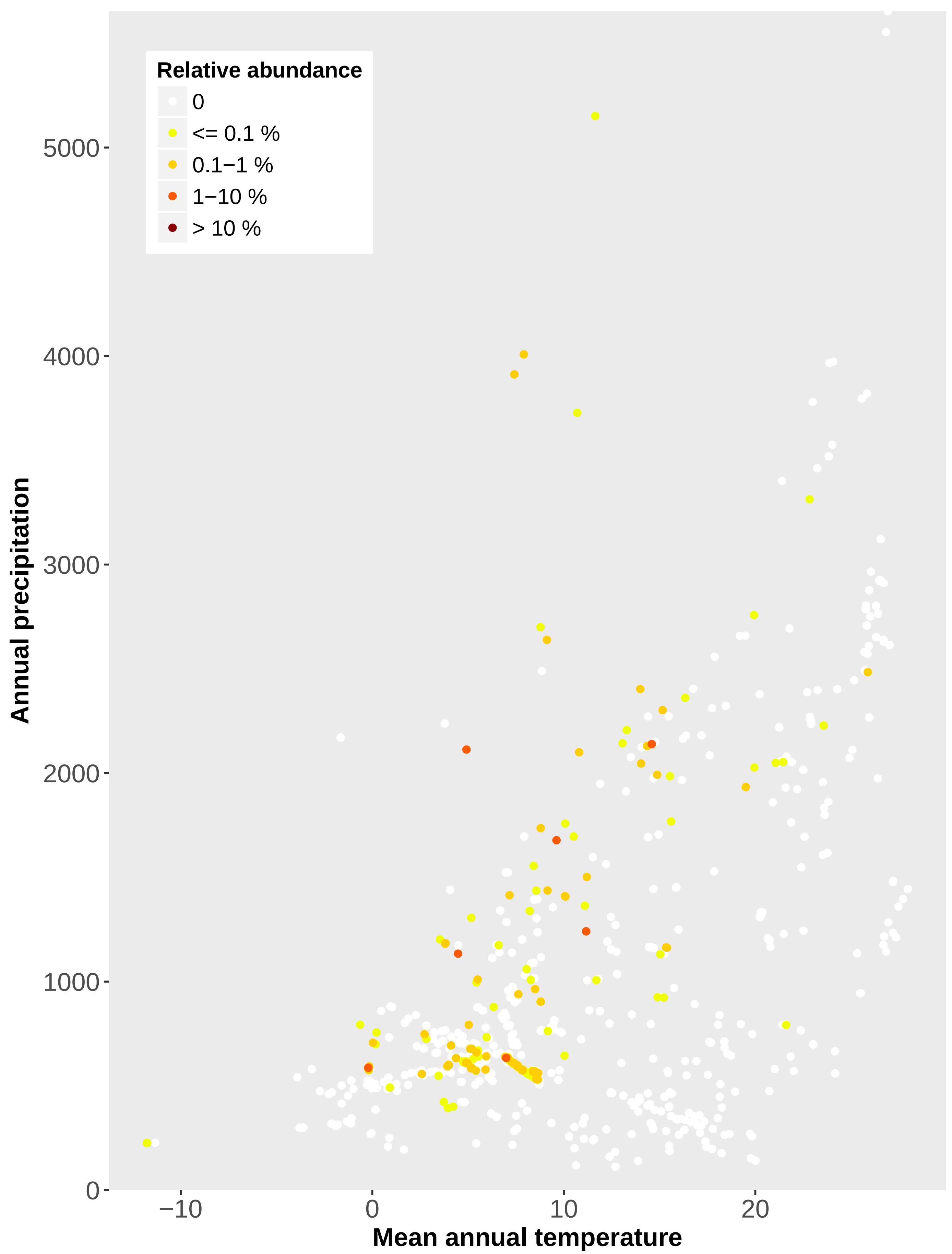

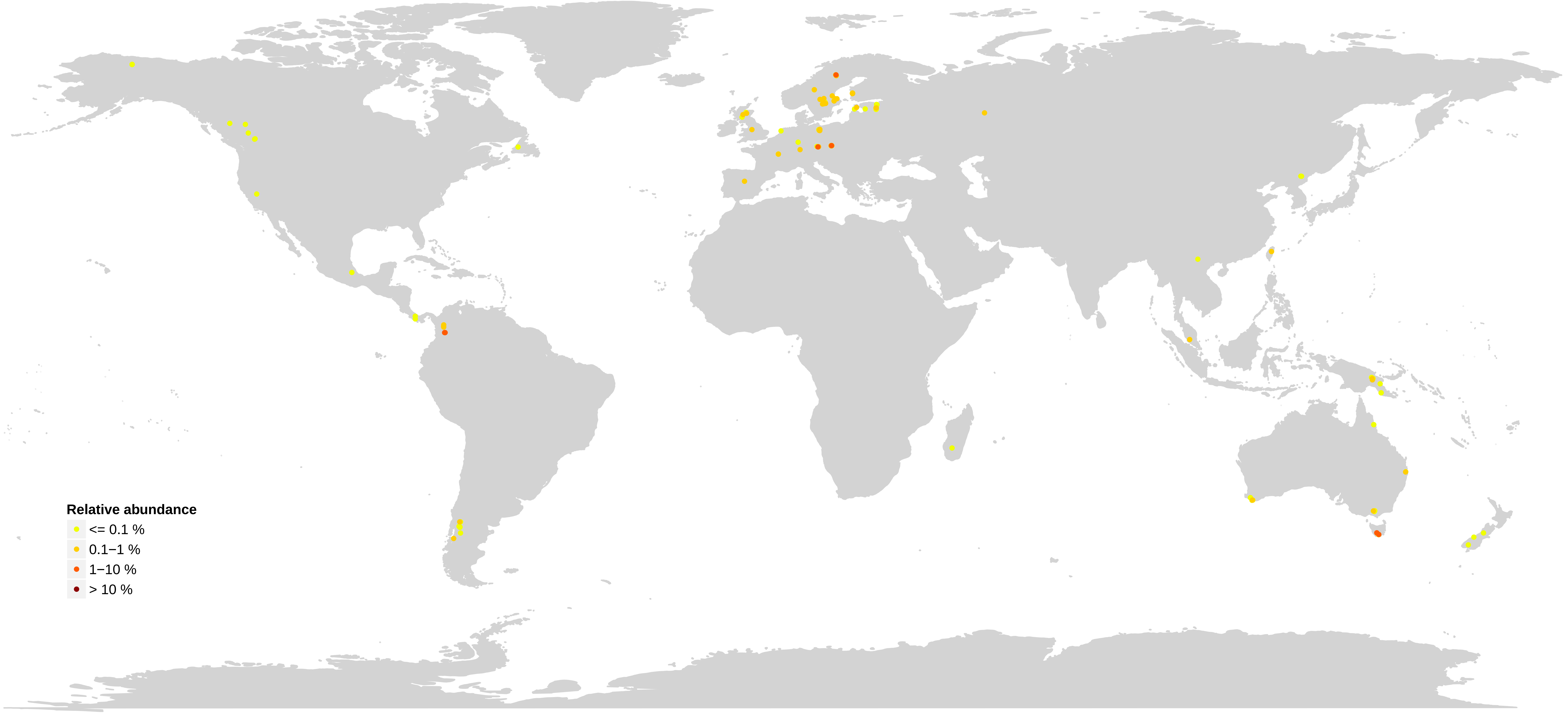

SH206474 Alternaria arborescens

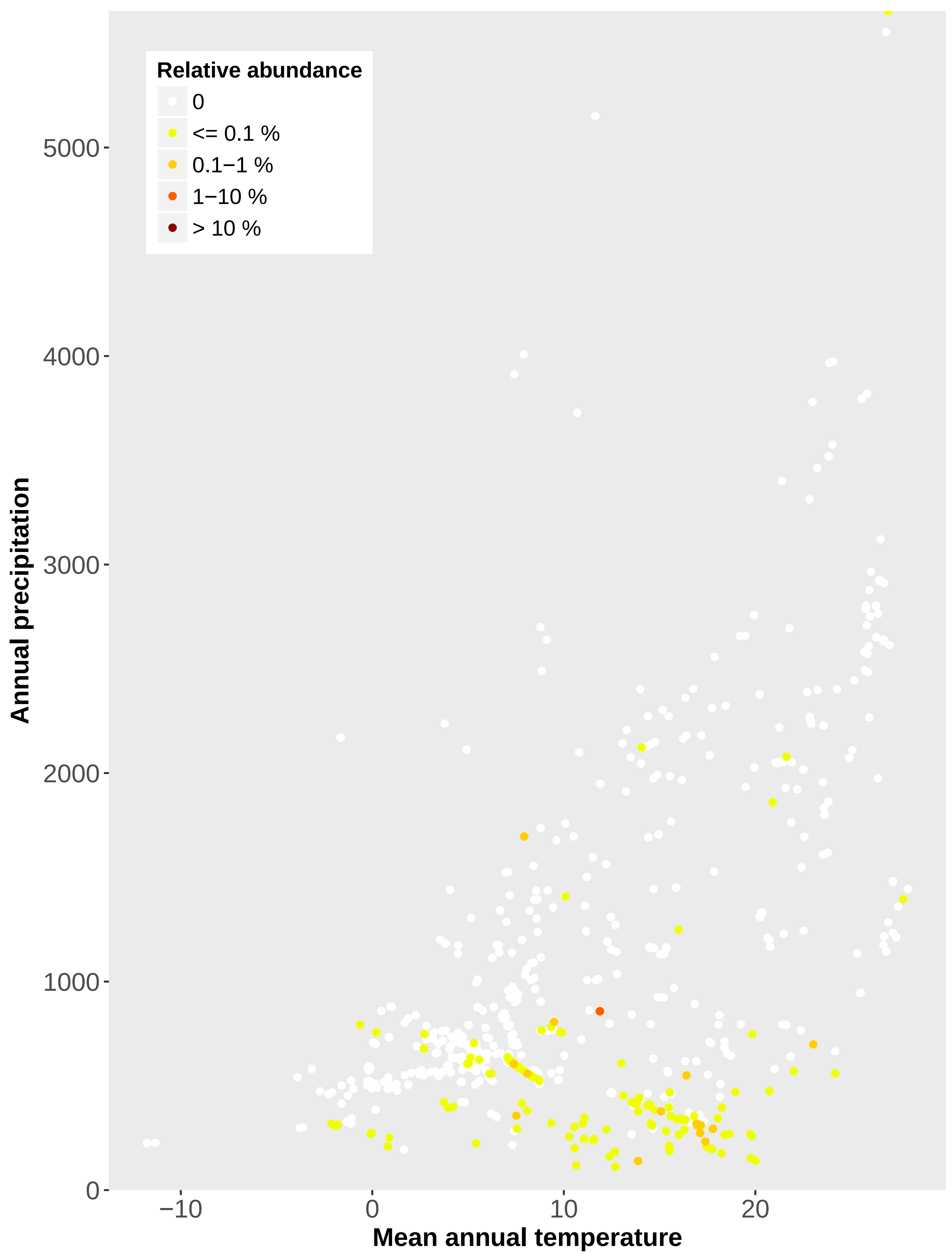

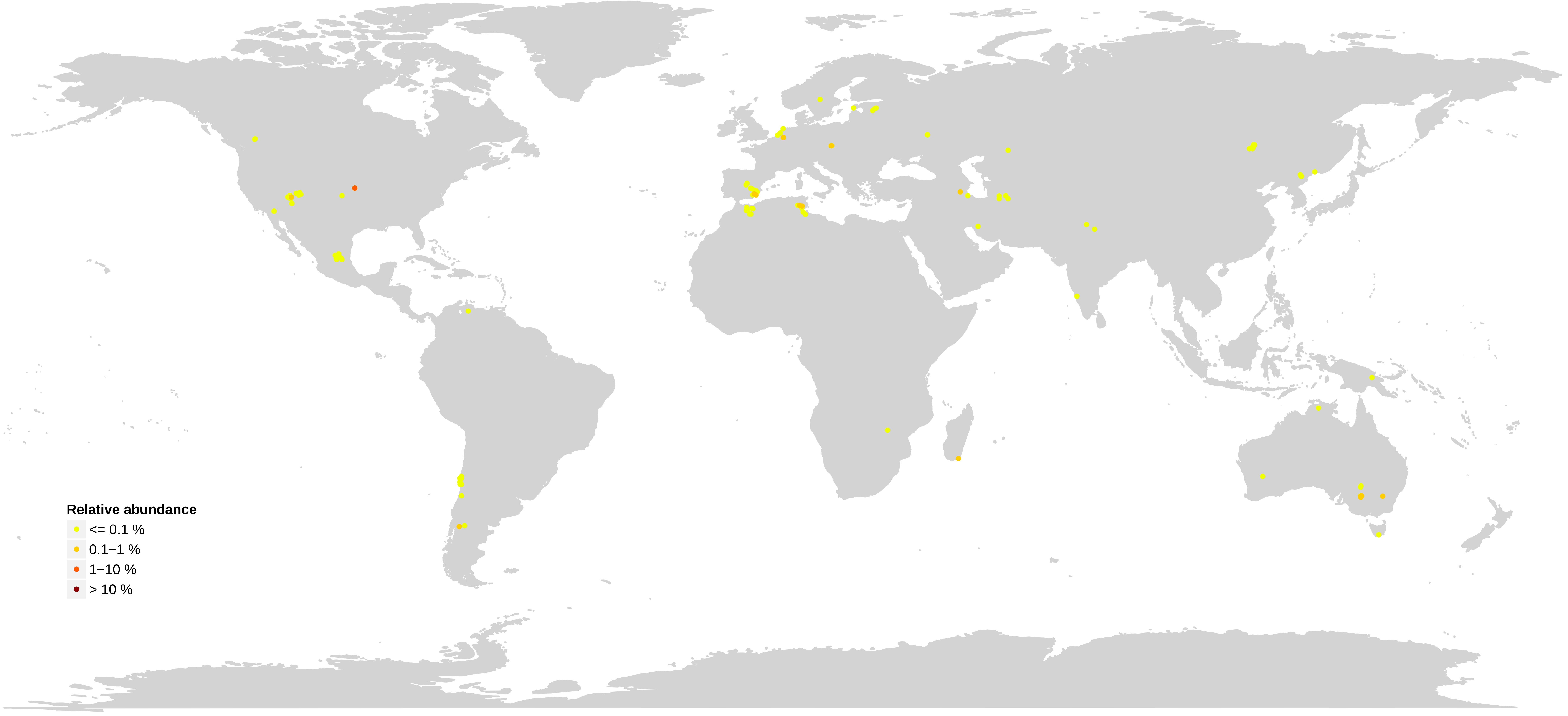

SH187220 Polyporales sp

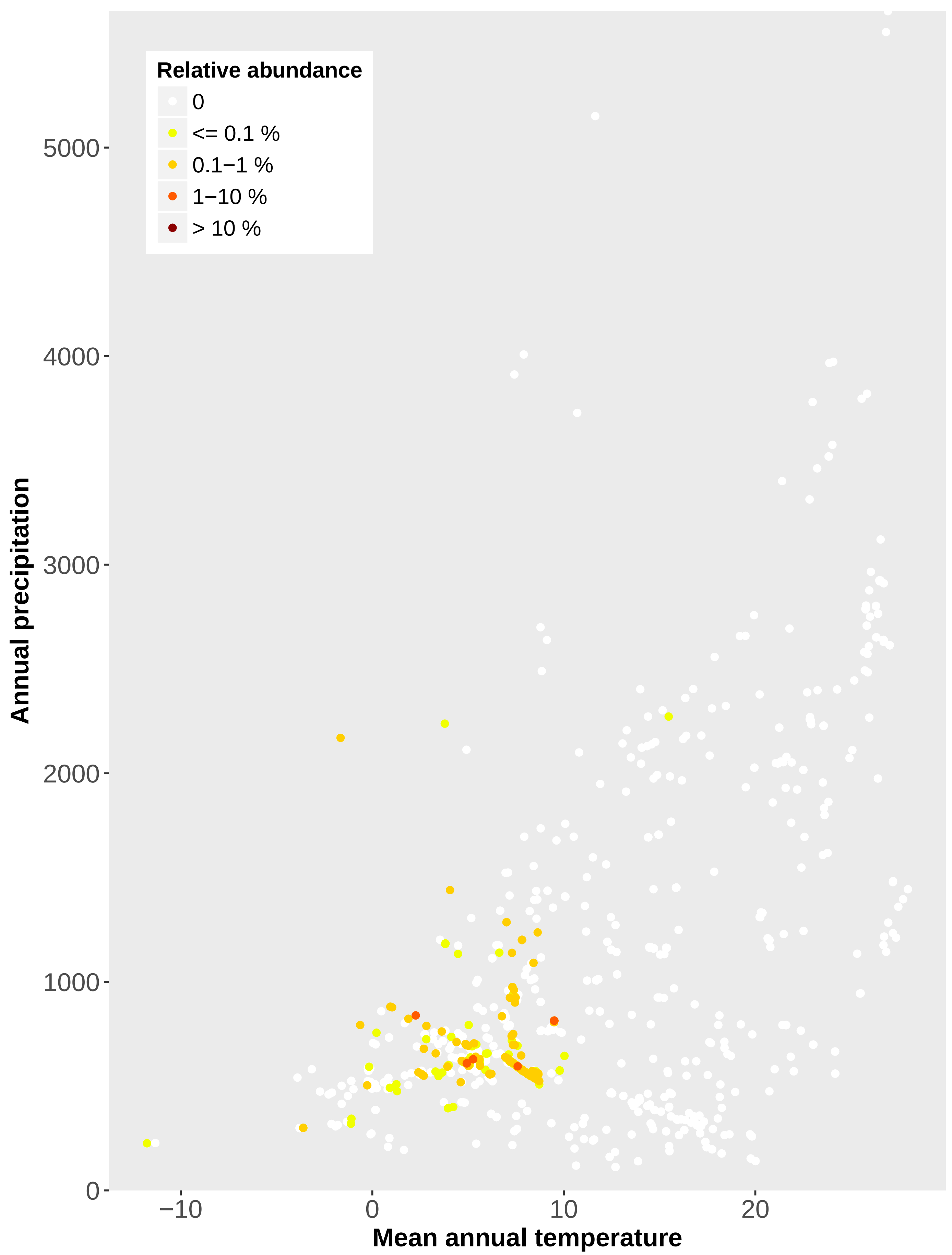

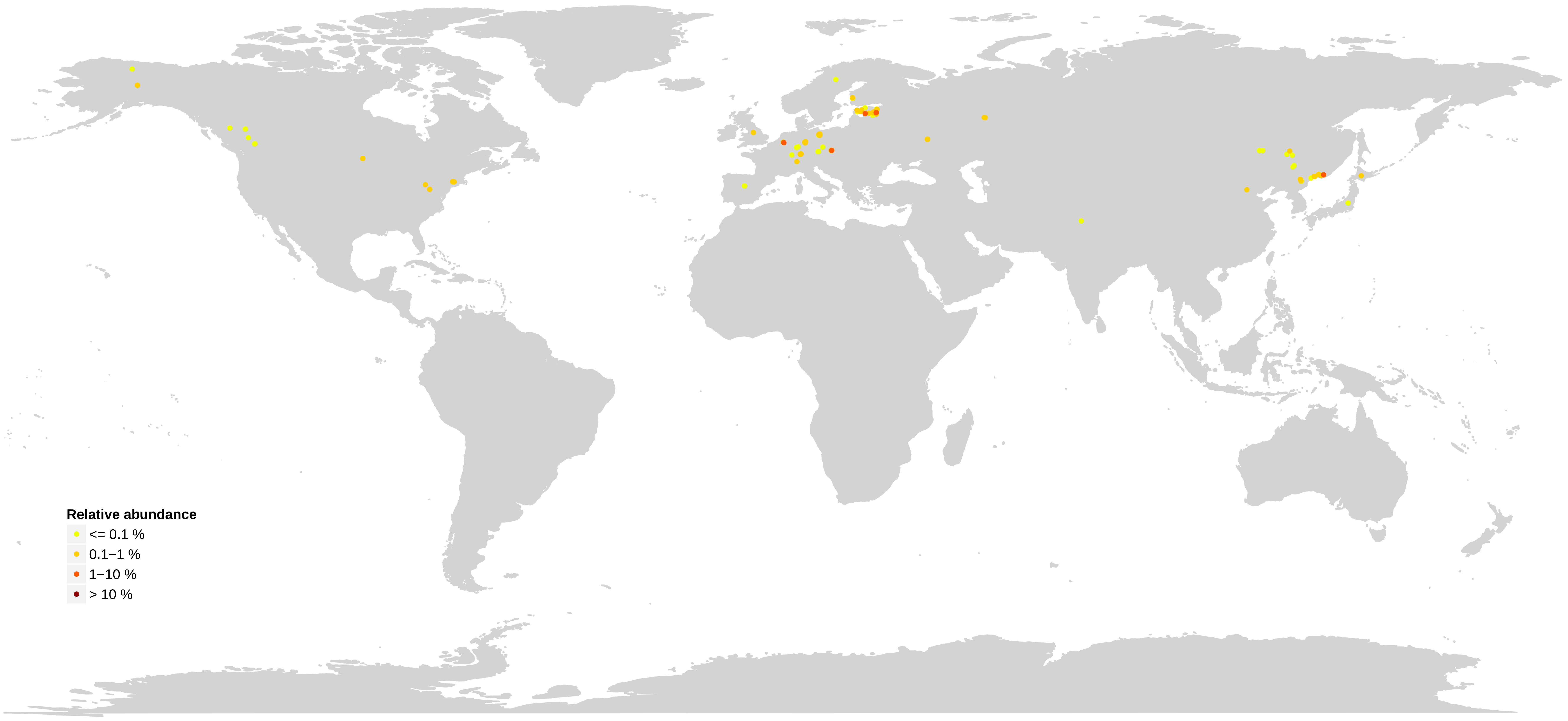

SH204986 Phialocephala fortinii

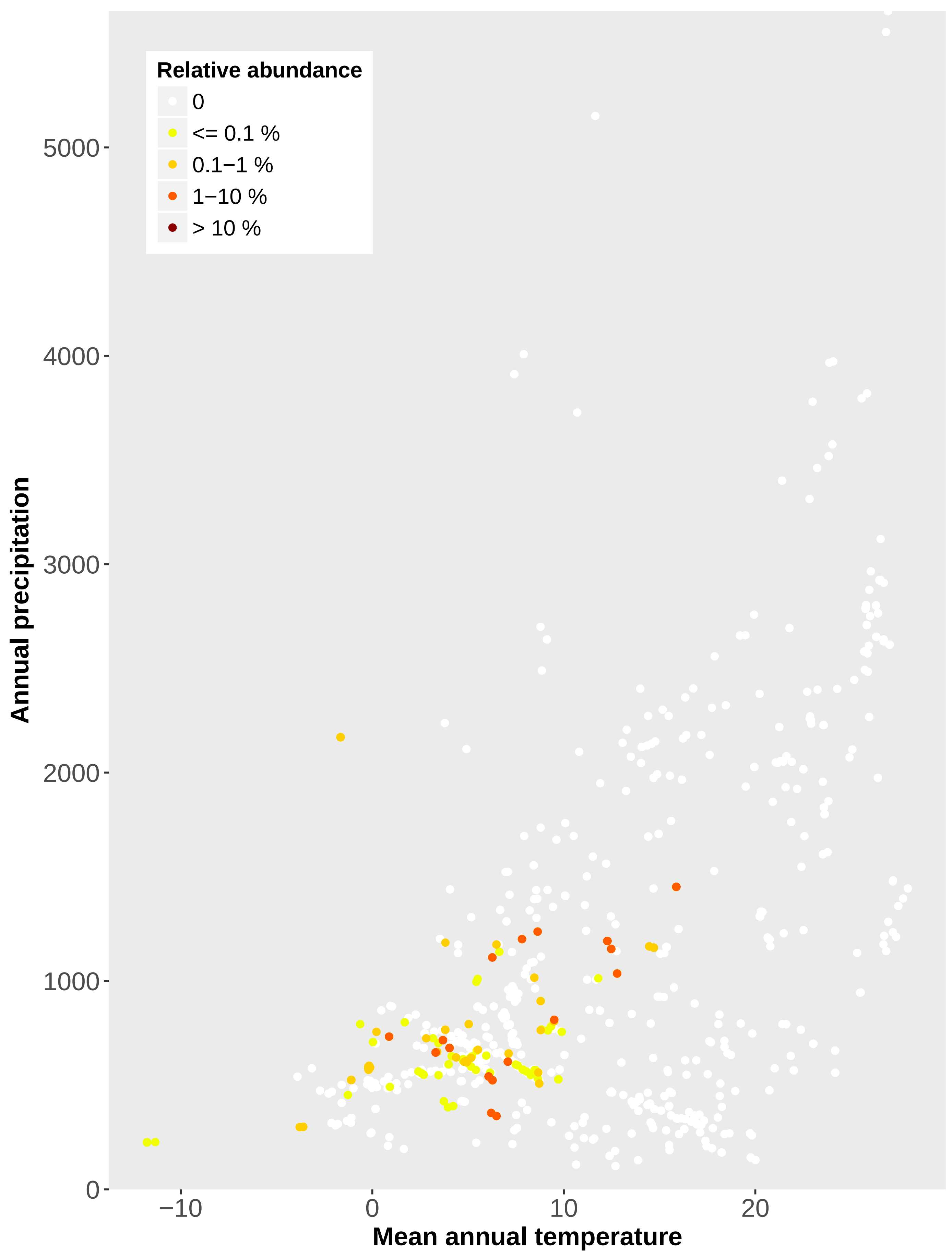

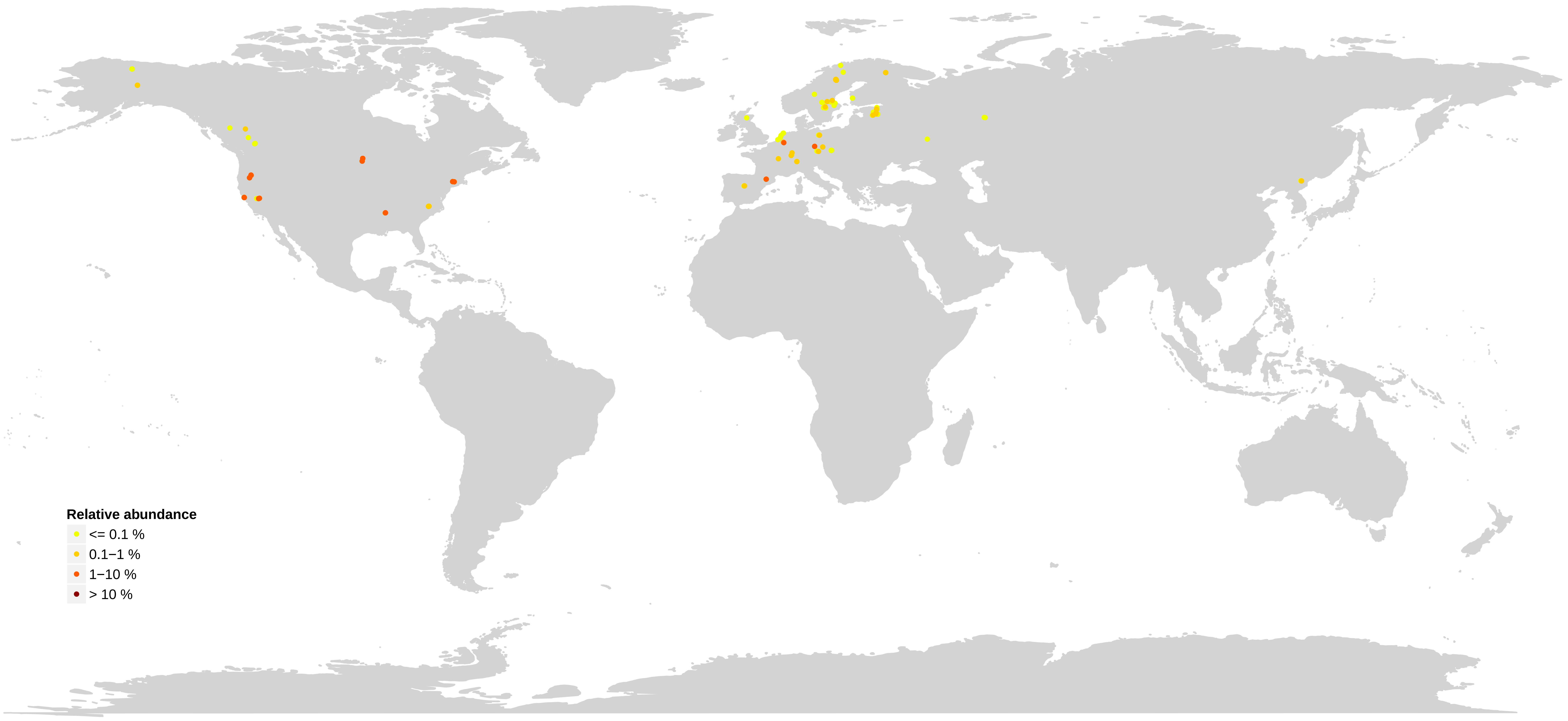

SH521508 Ascomycota sp

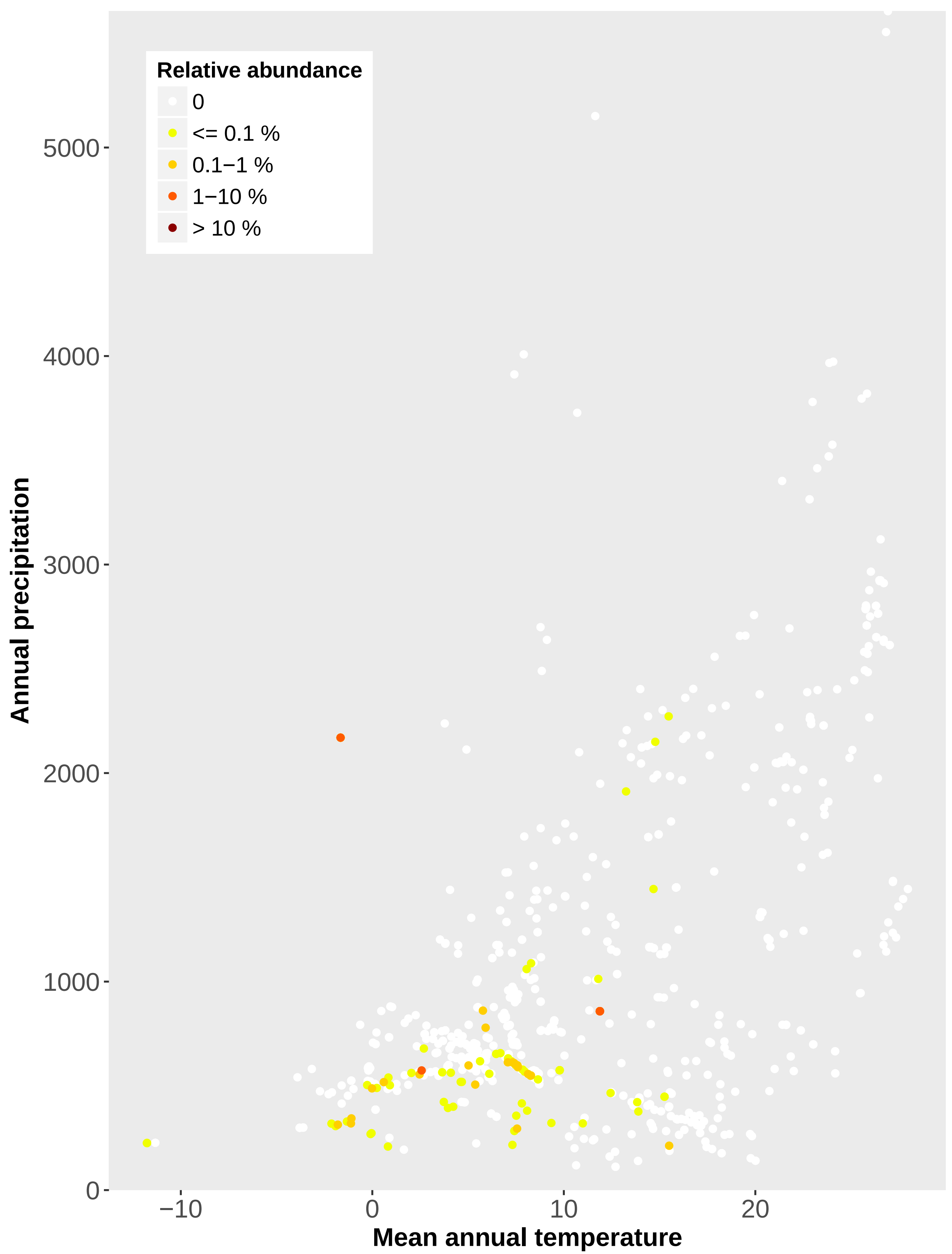

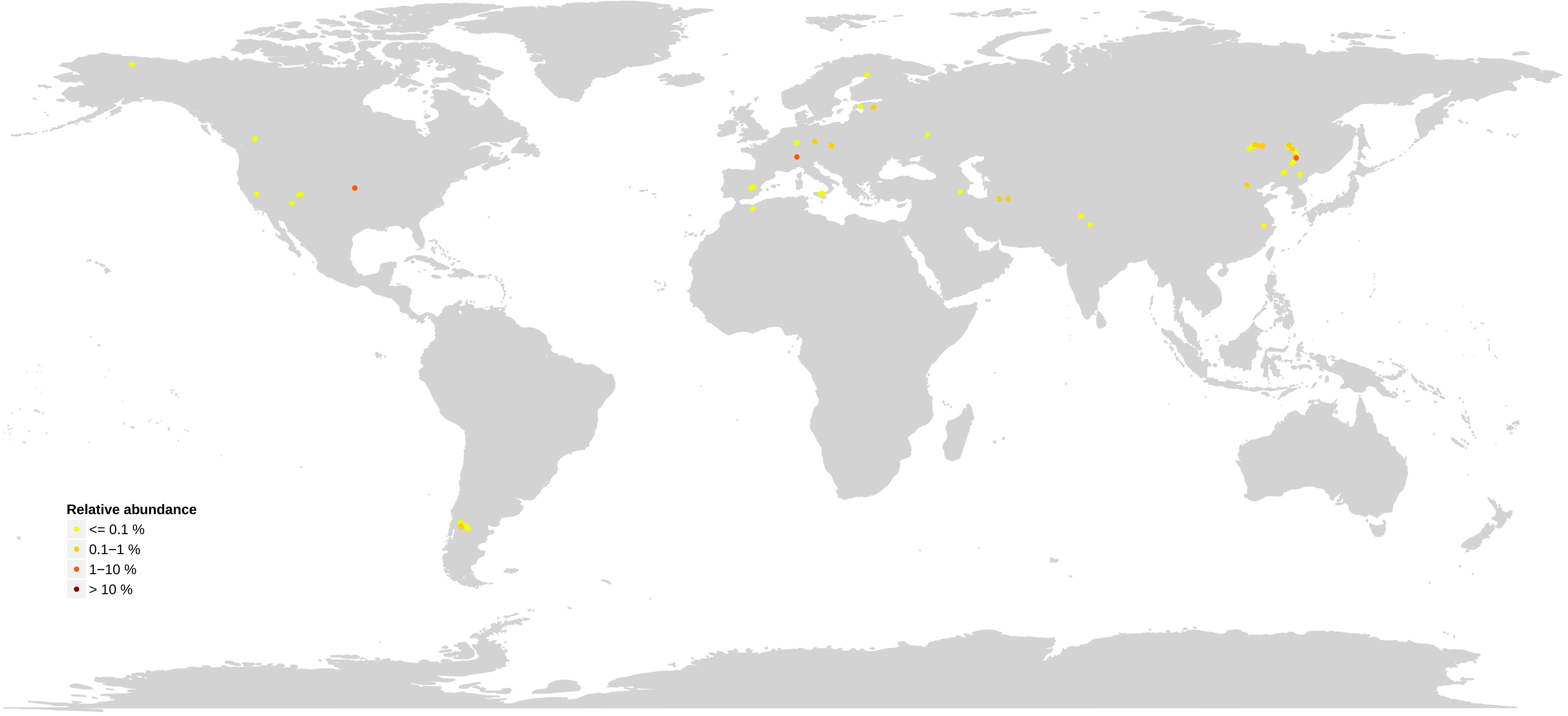

SH199403 Penicillium soppii

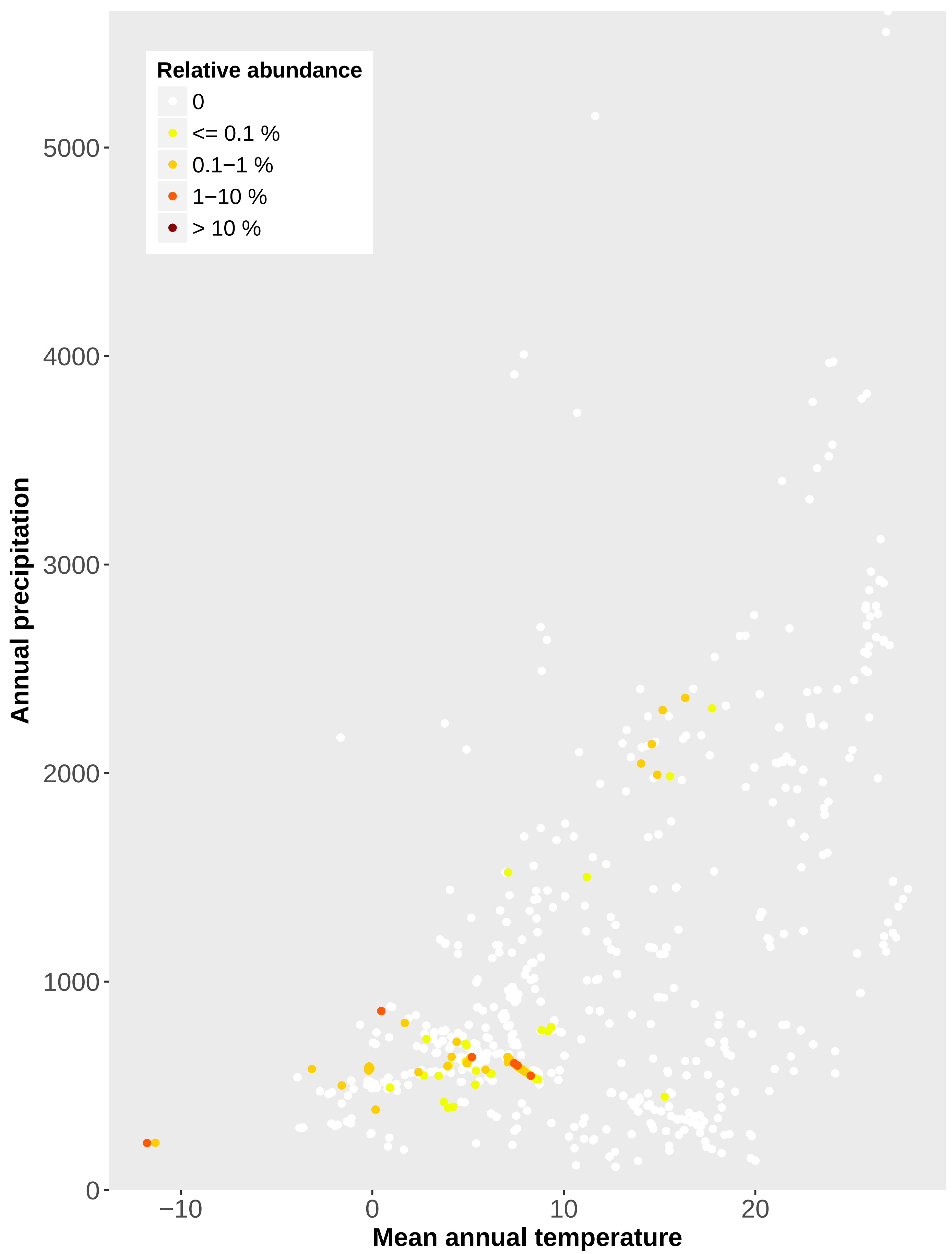

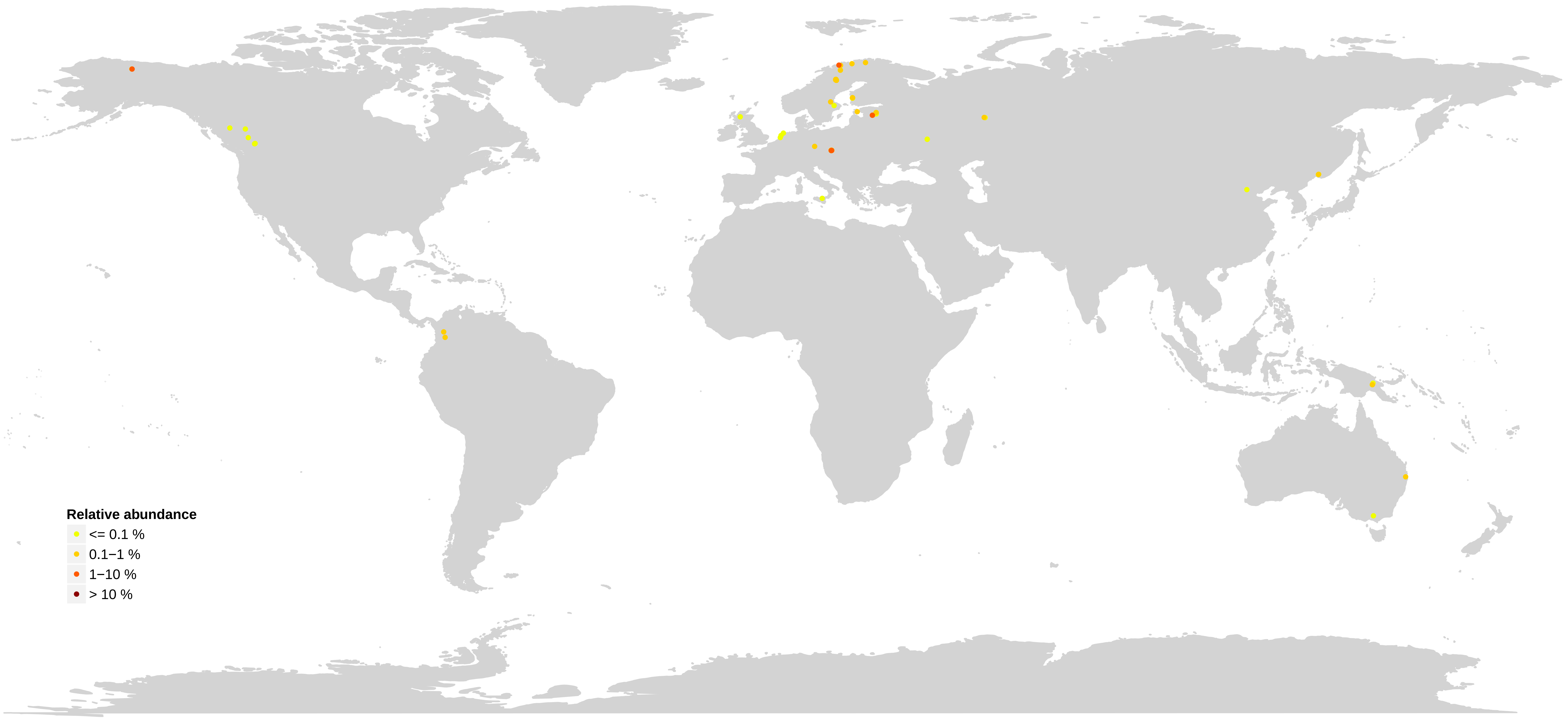

SH183634 Mortierella amoeboidea

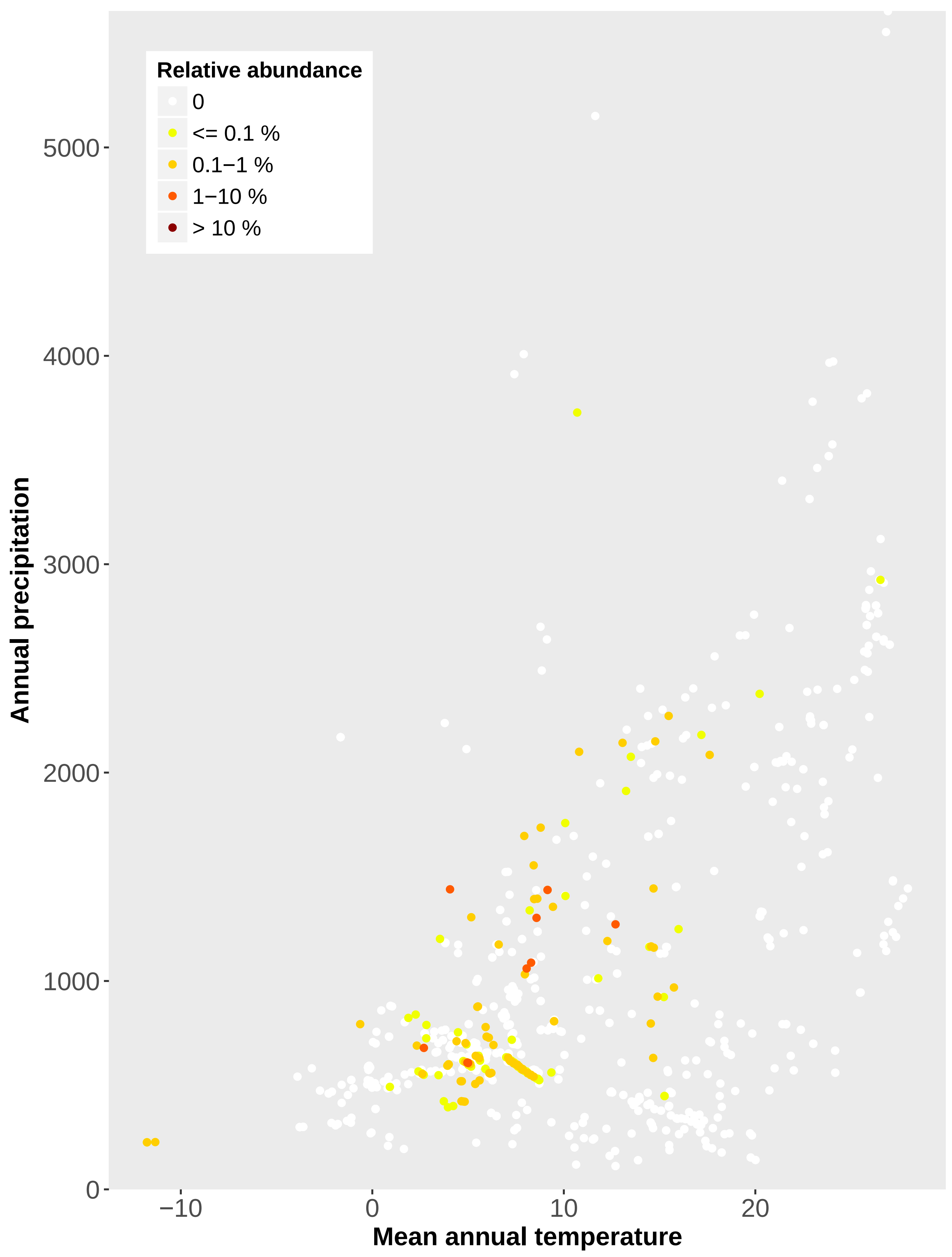

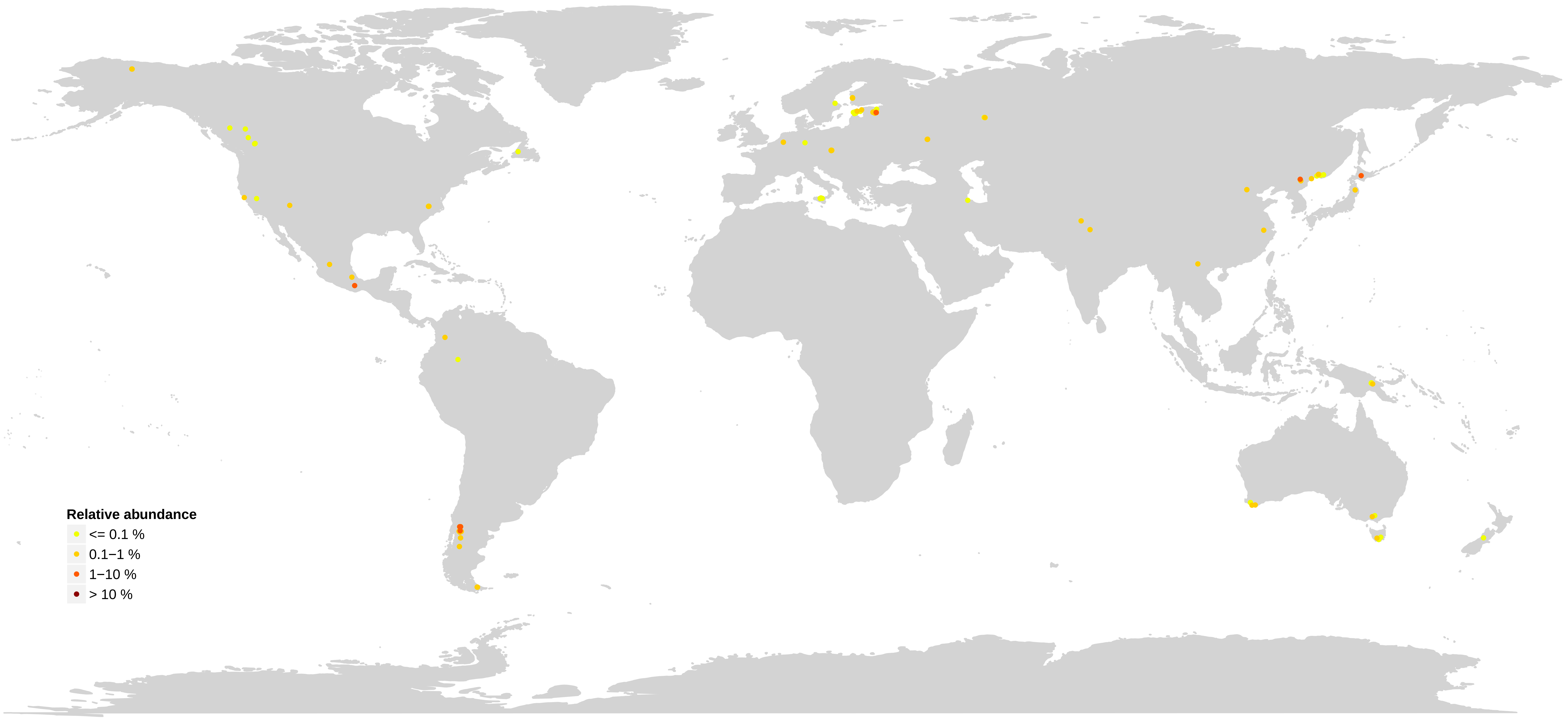

SH202693 Xenopolyscytalum pinea

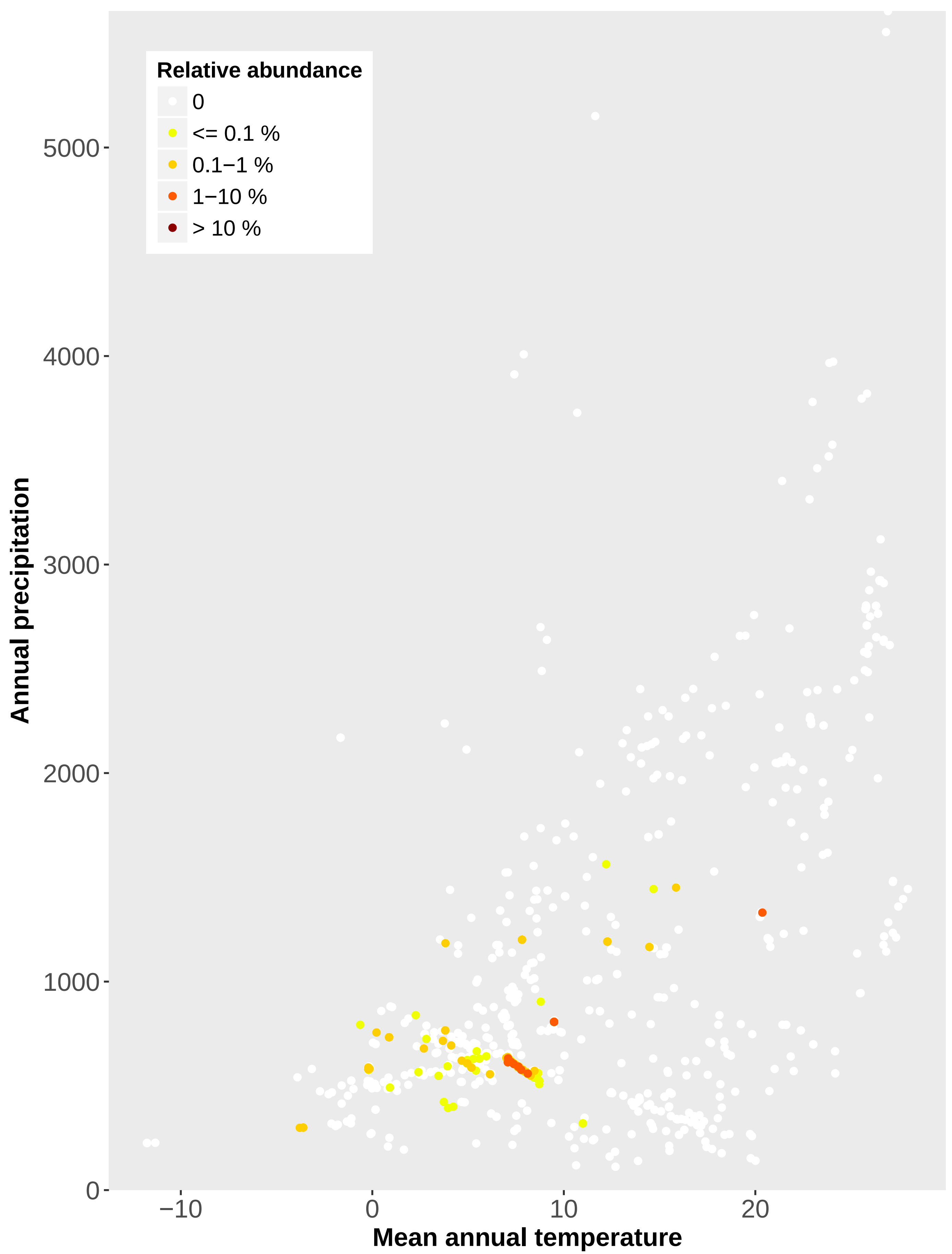

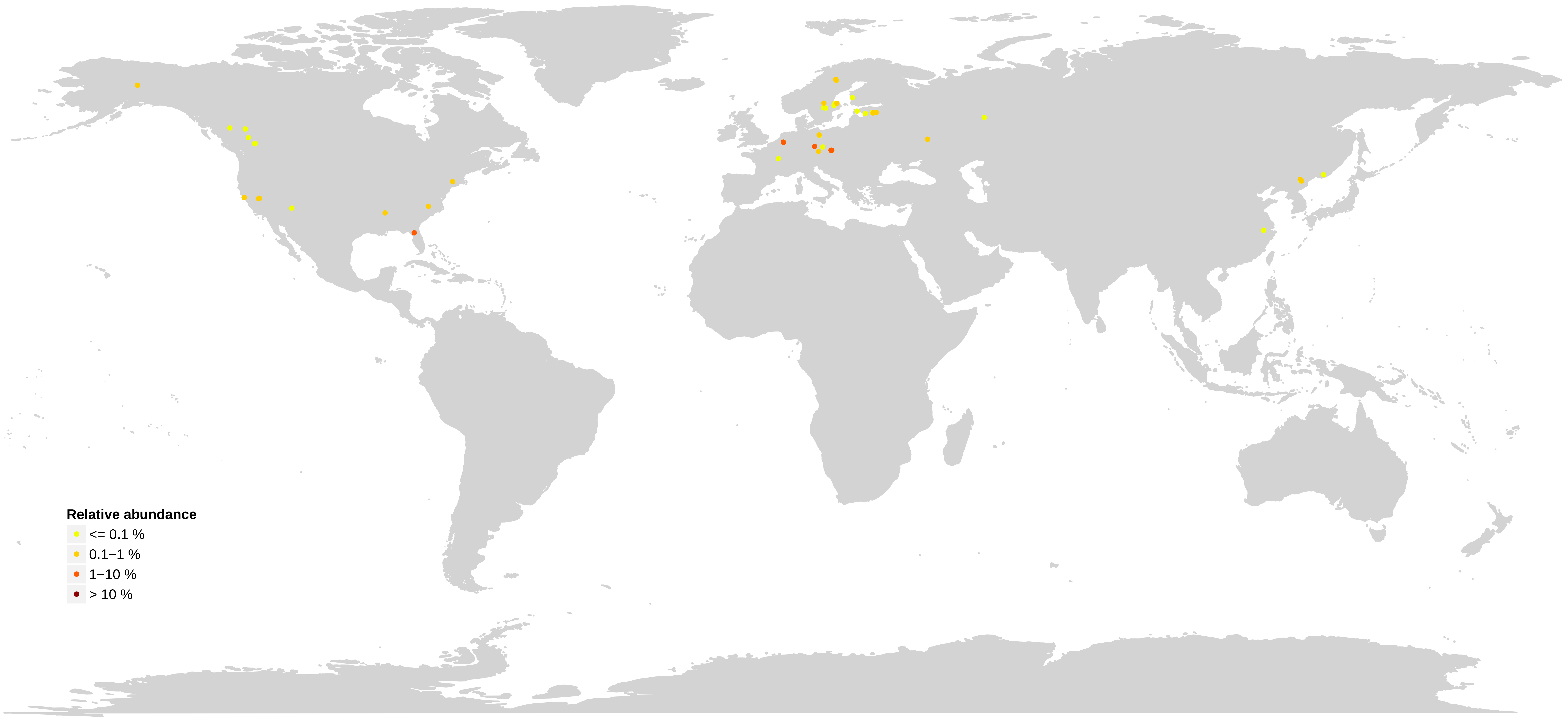

SH202969 Nectria ramulariae

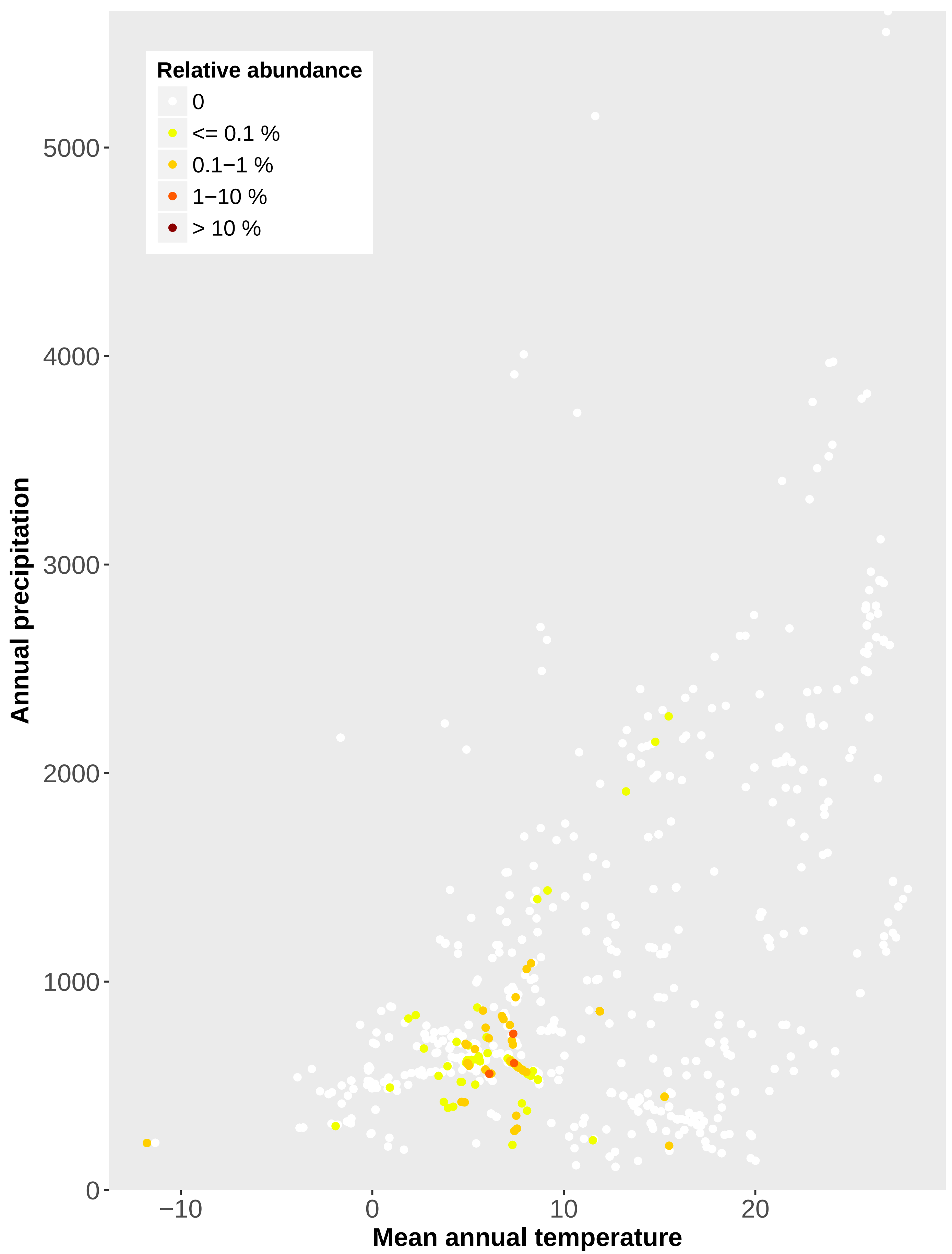

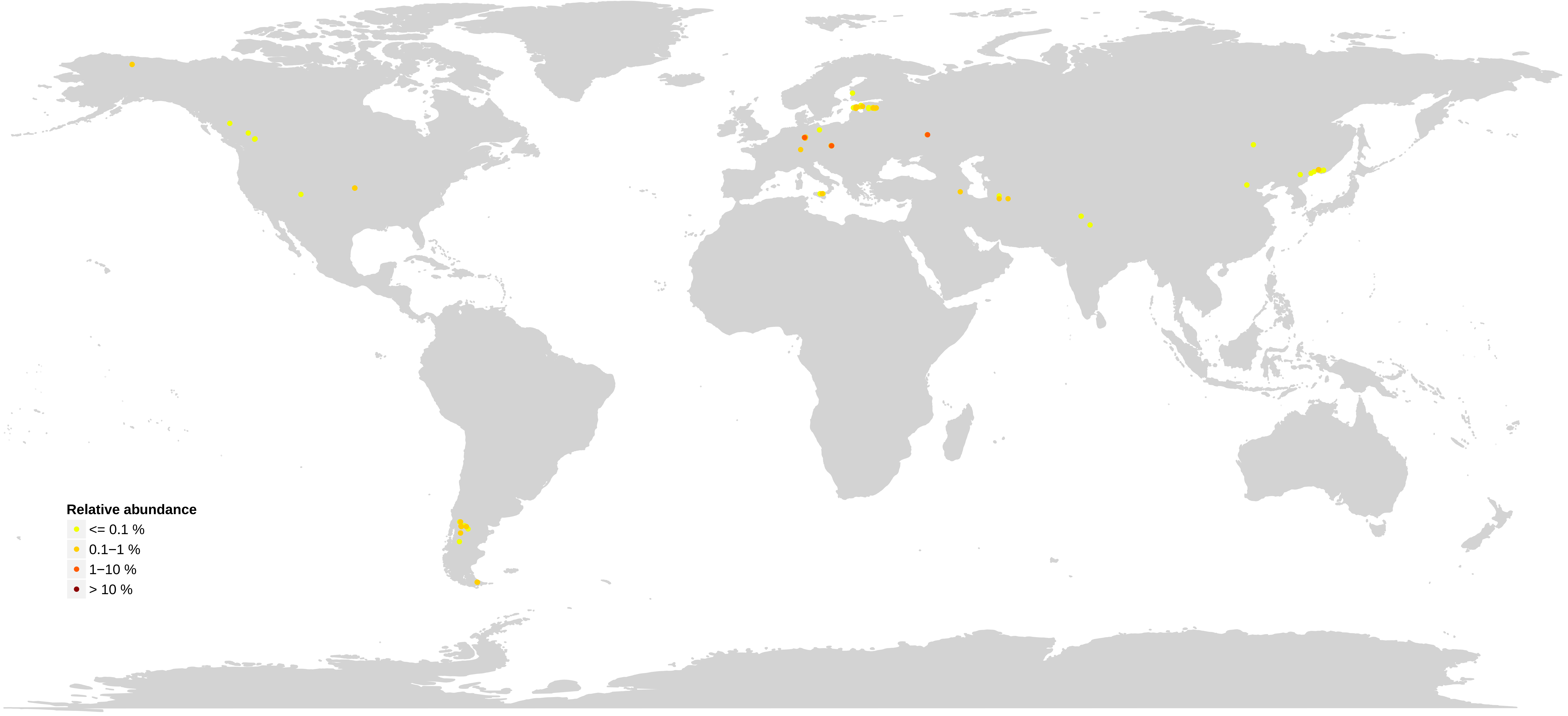

SH407681 Penicillium chrysogenum

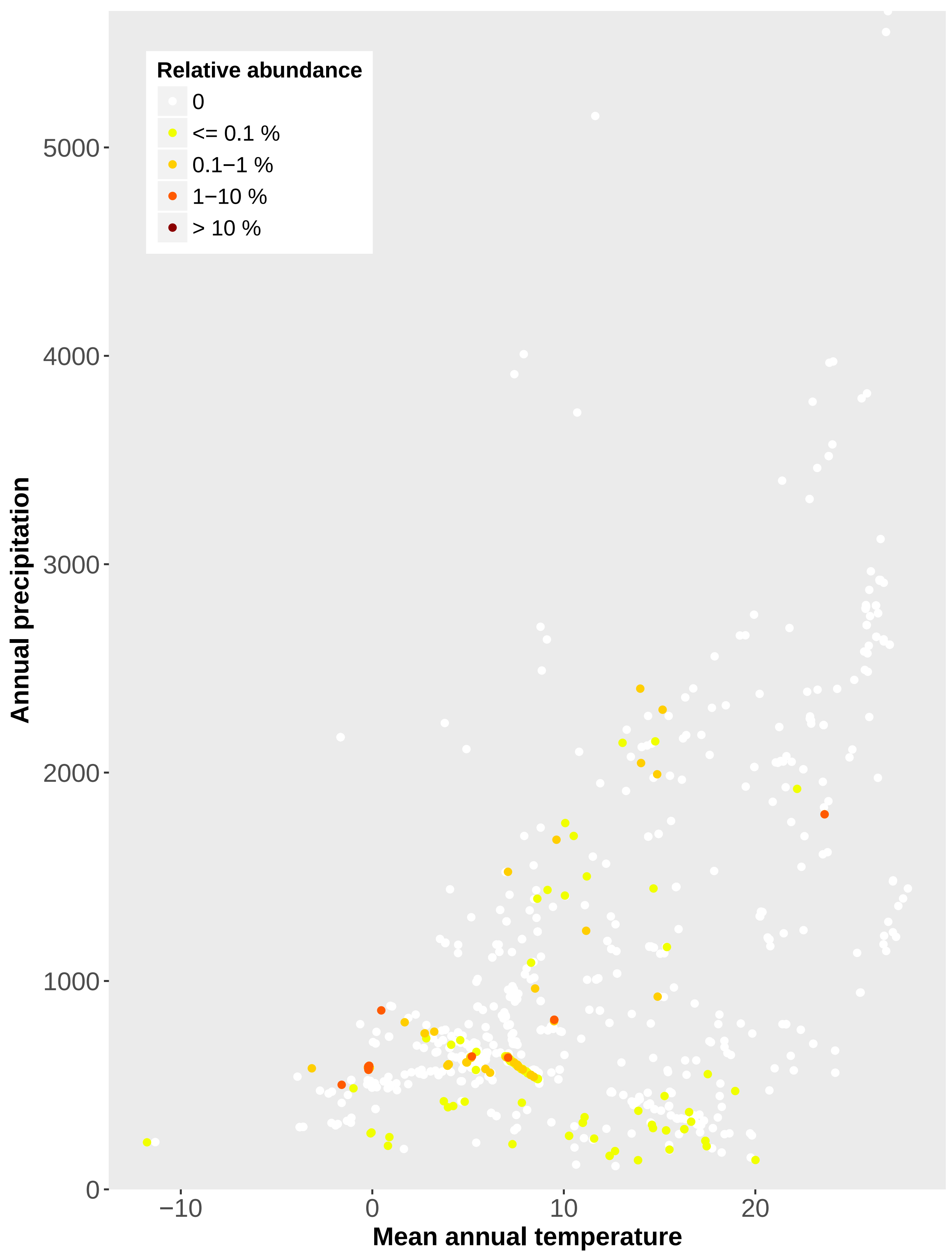

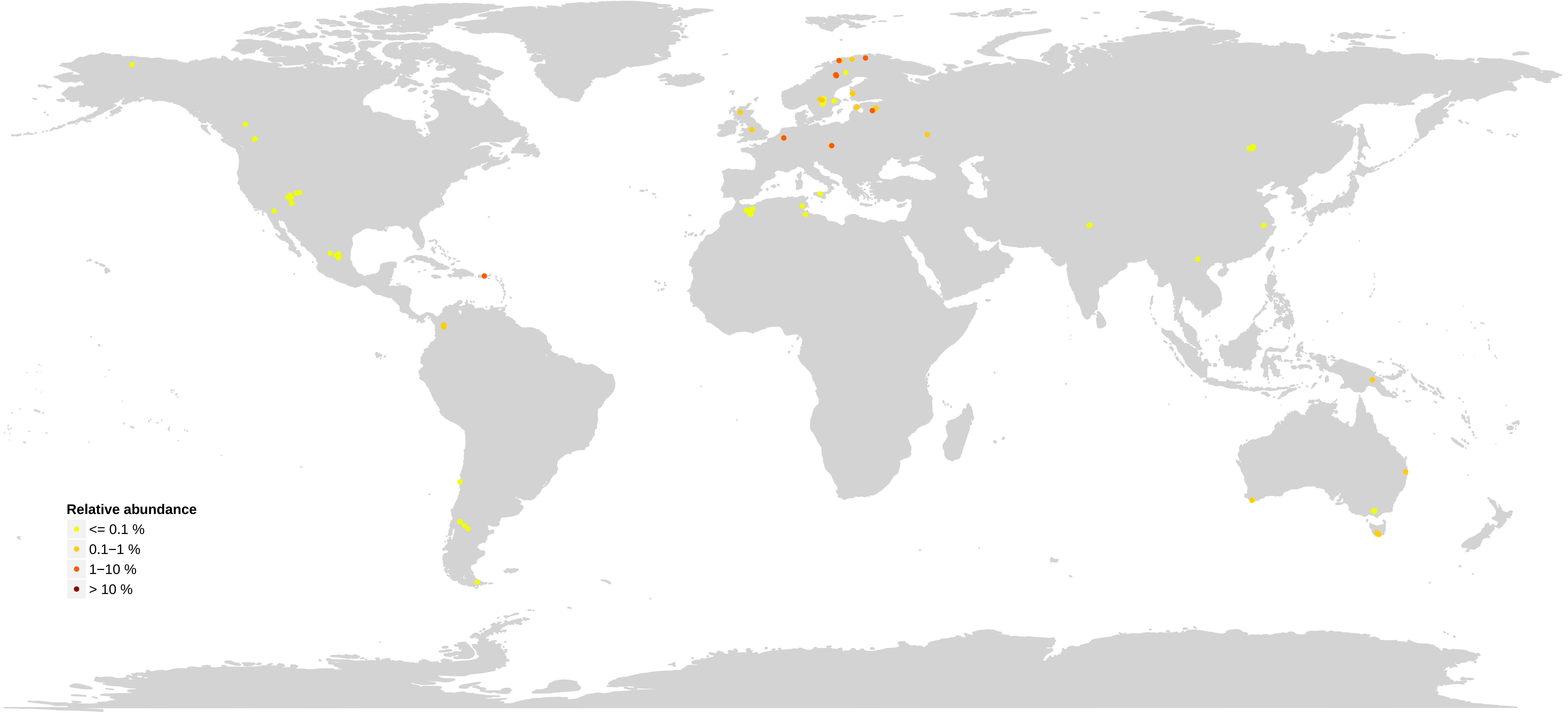

SH357830 Cryptococcus fuscescens

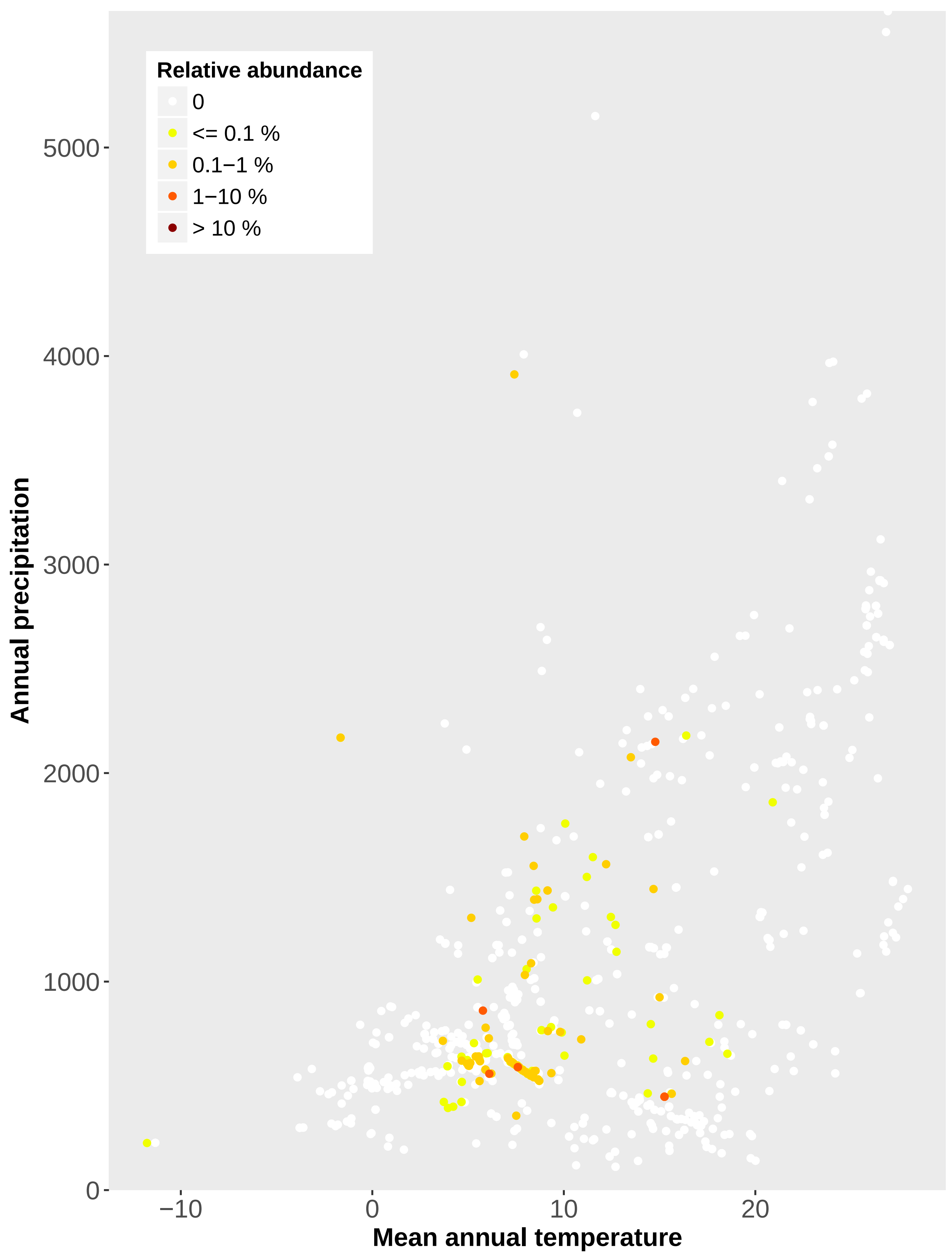

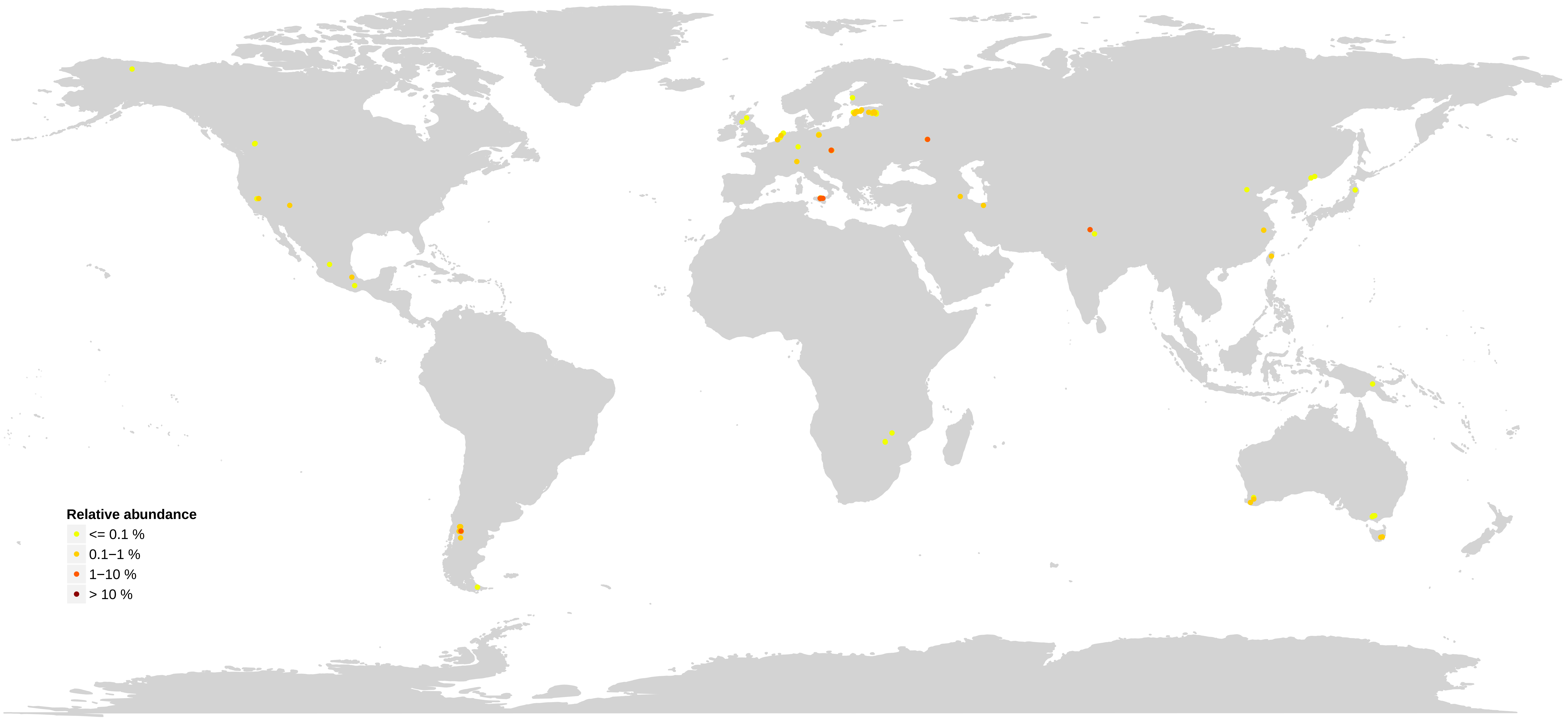

SH214294 Helotiales sp

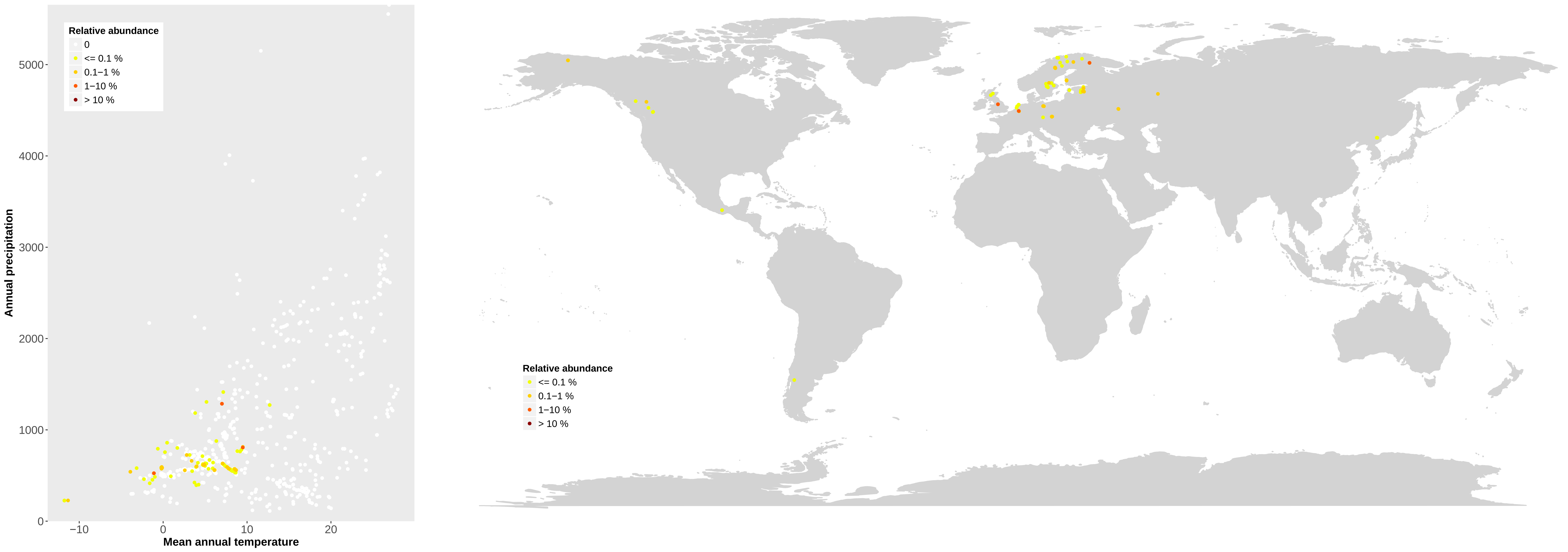

SH182913 Phaeosphaeriaceae sp

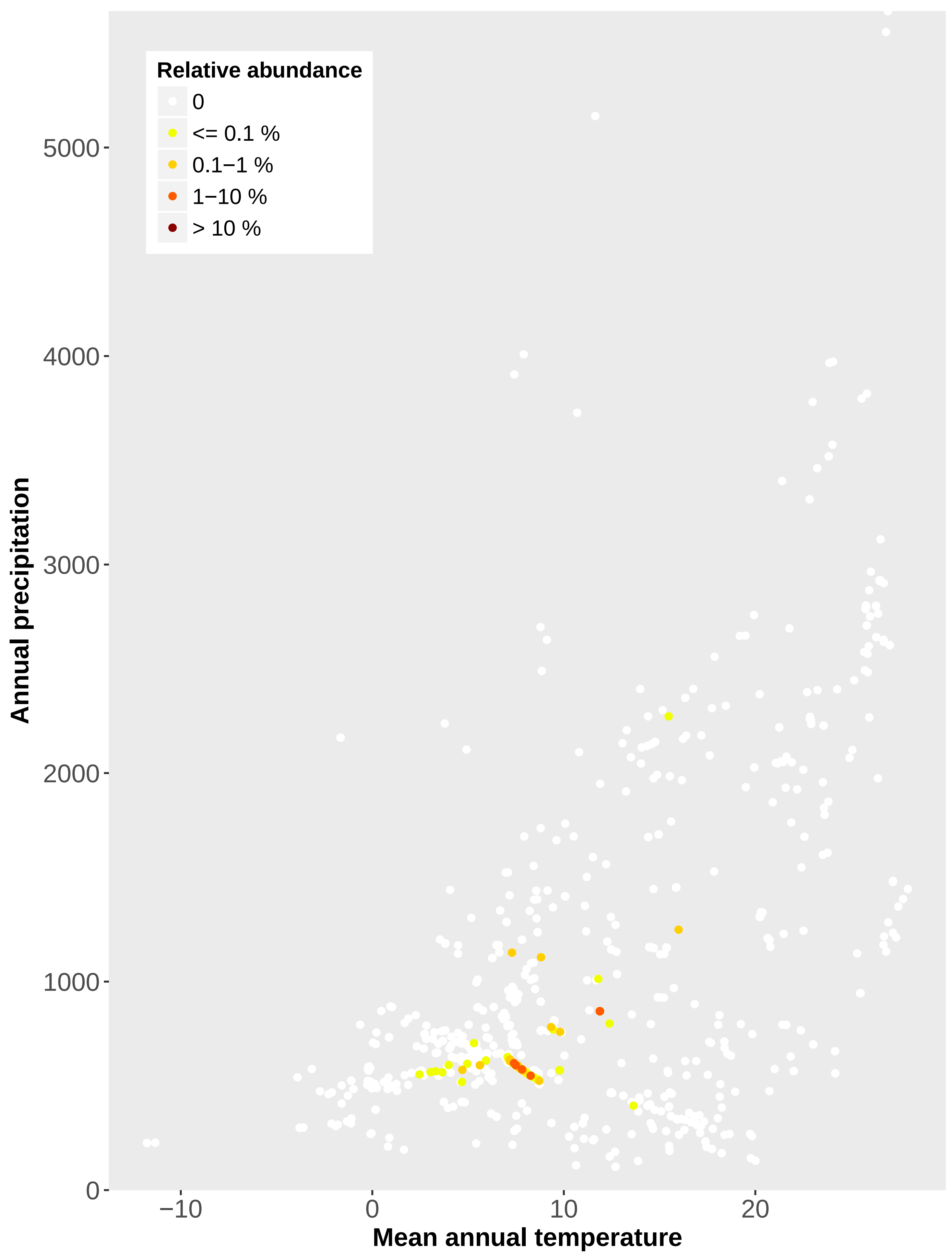

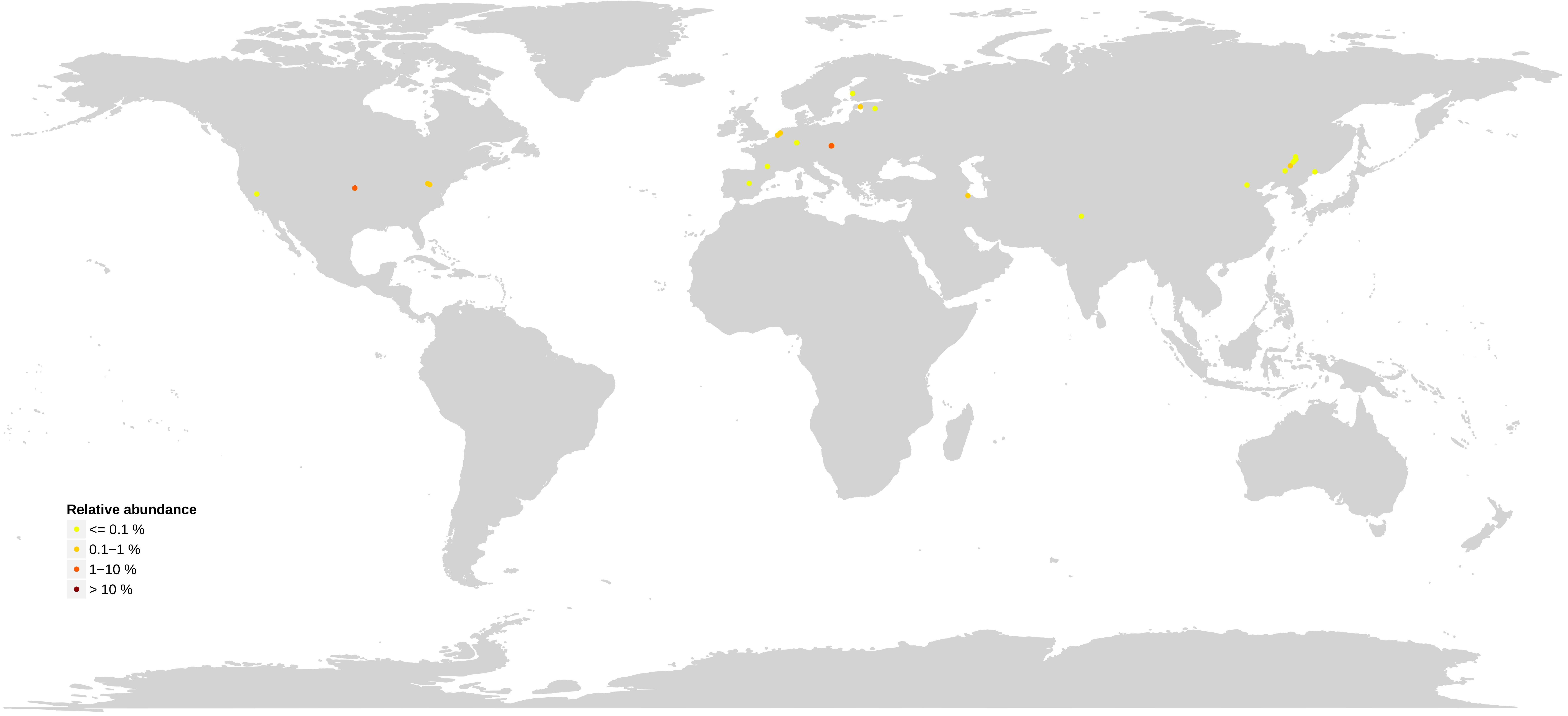

SH493946 Leptosphaeriaceae sp

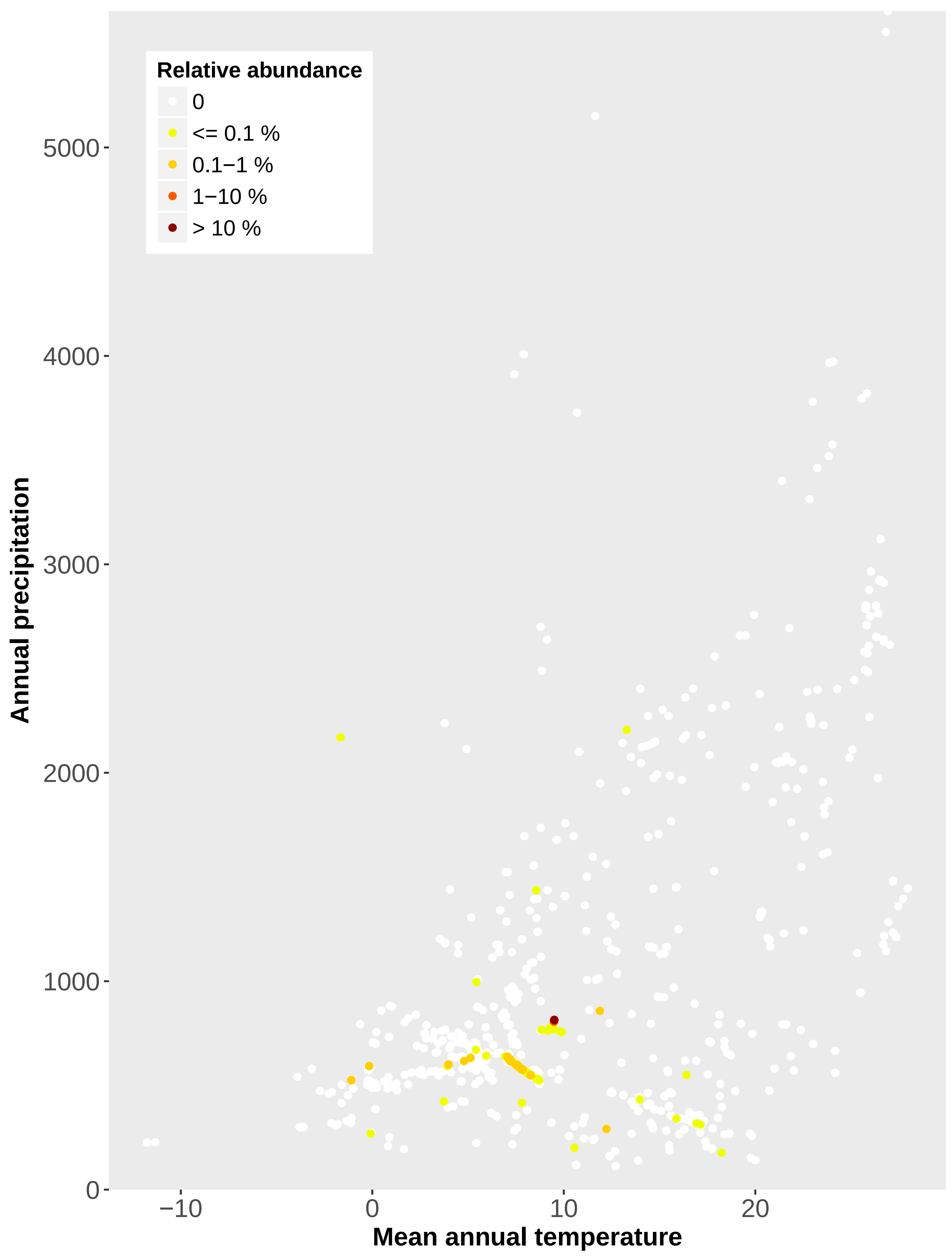

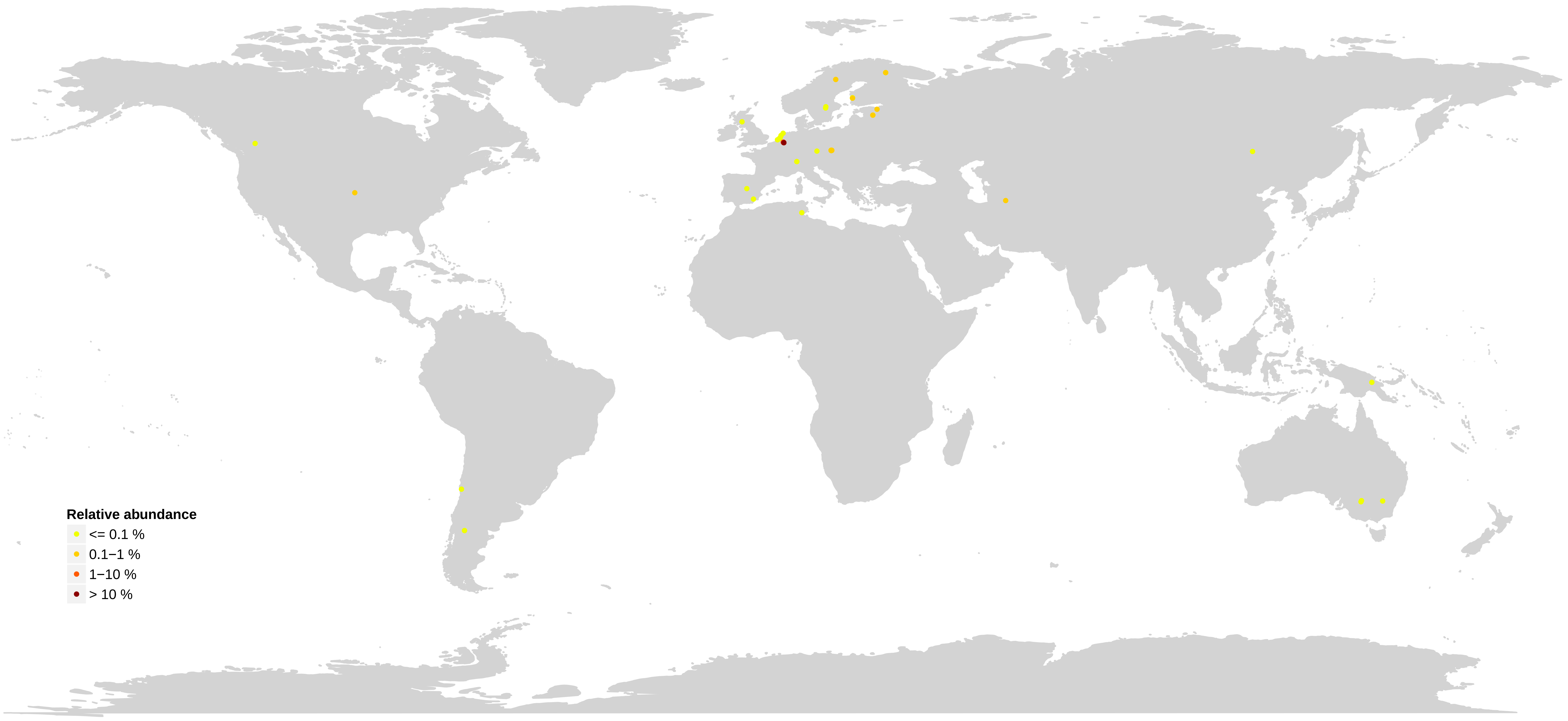

SH217842 Chaetothyriales sp

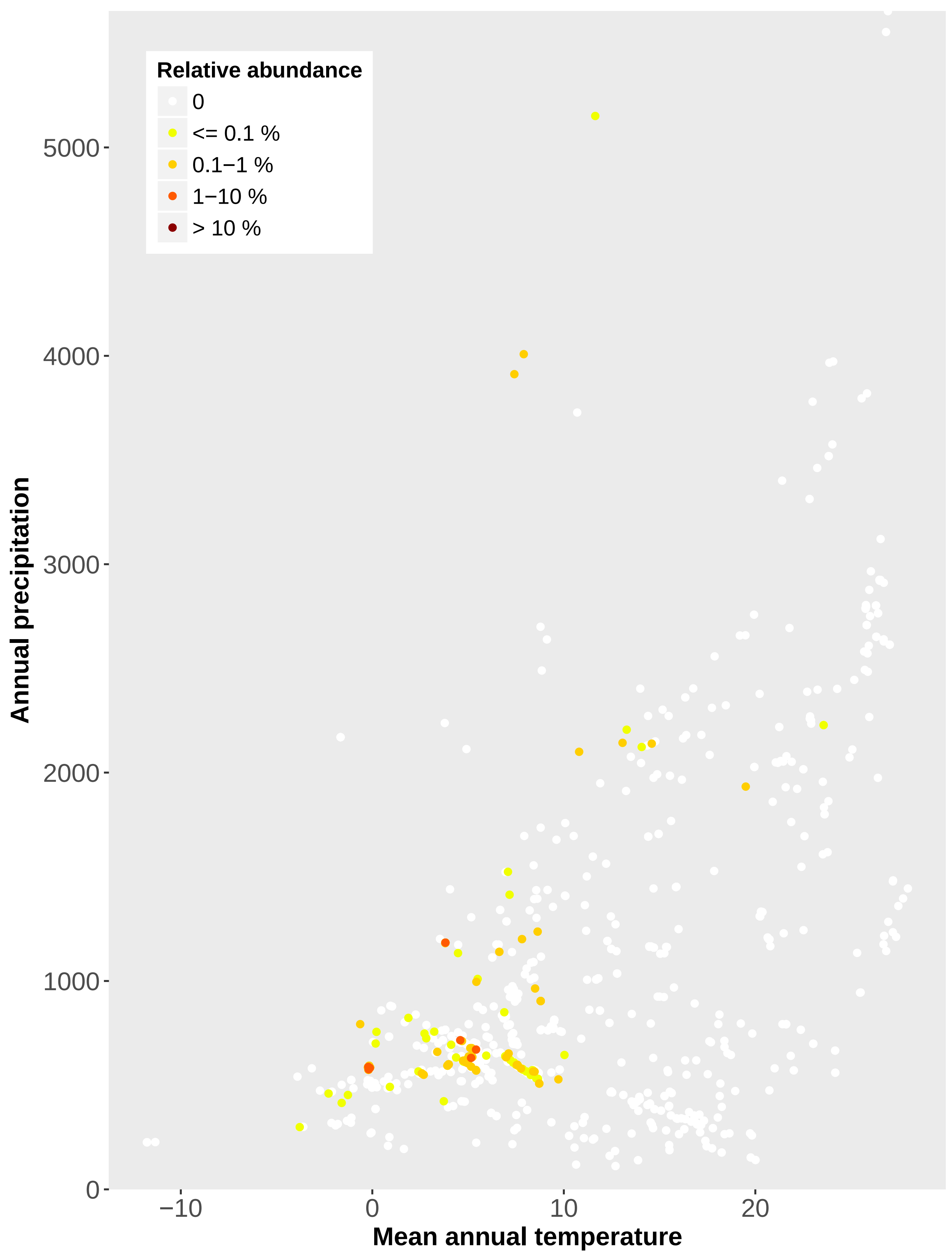

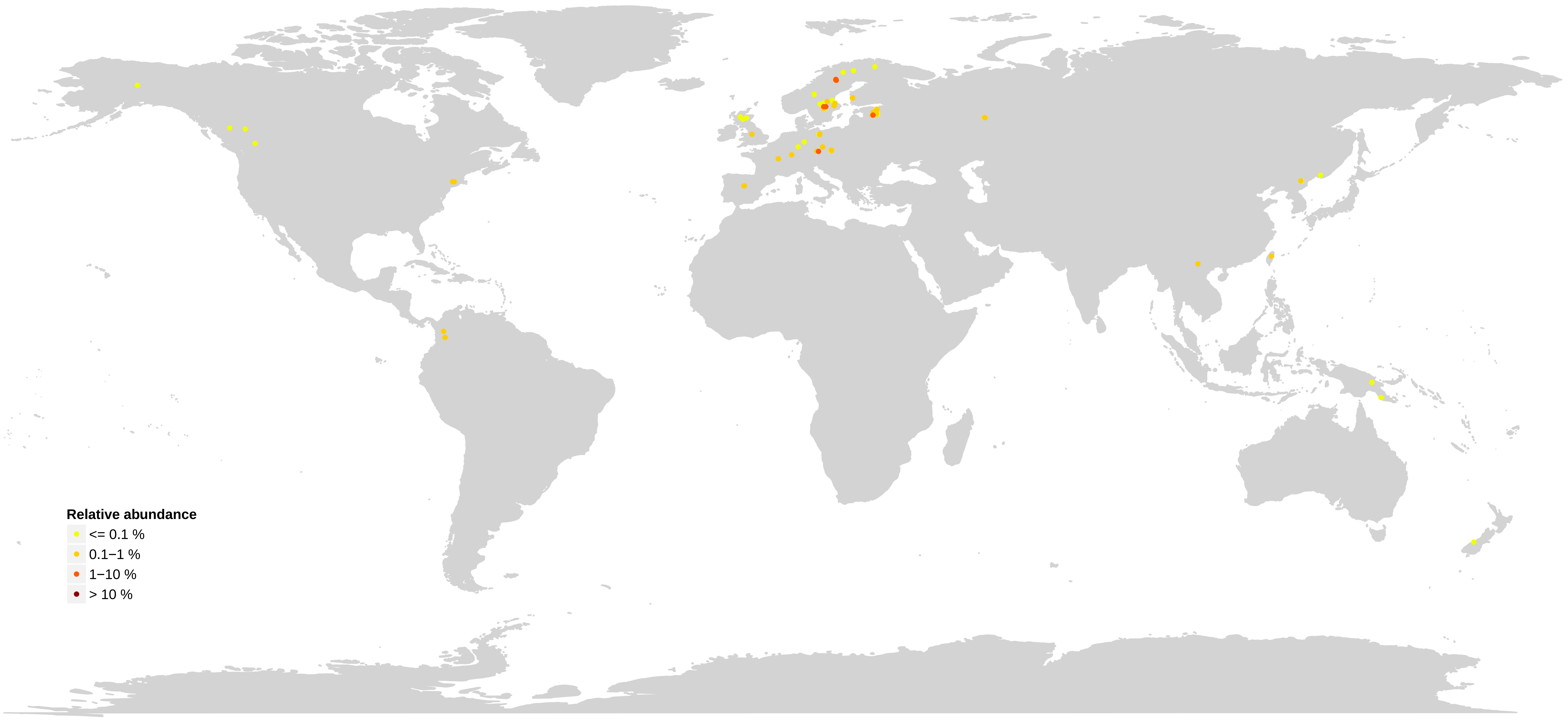

SH199633 Mortierella horticola

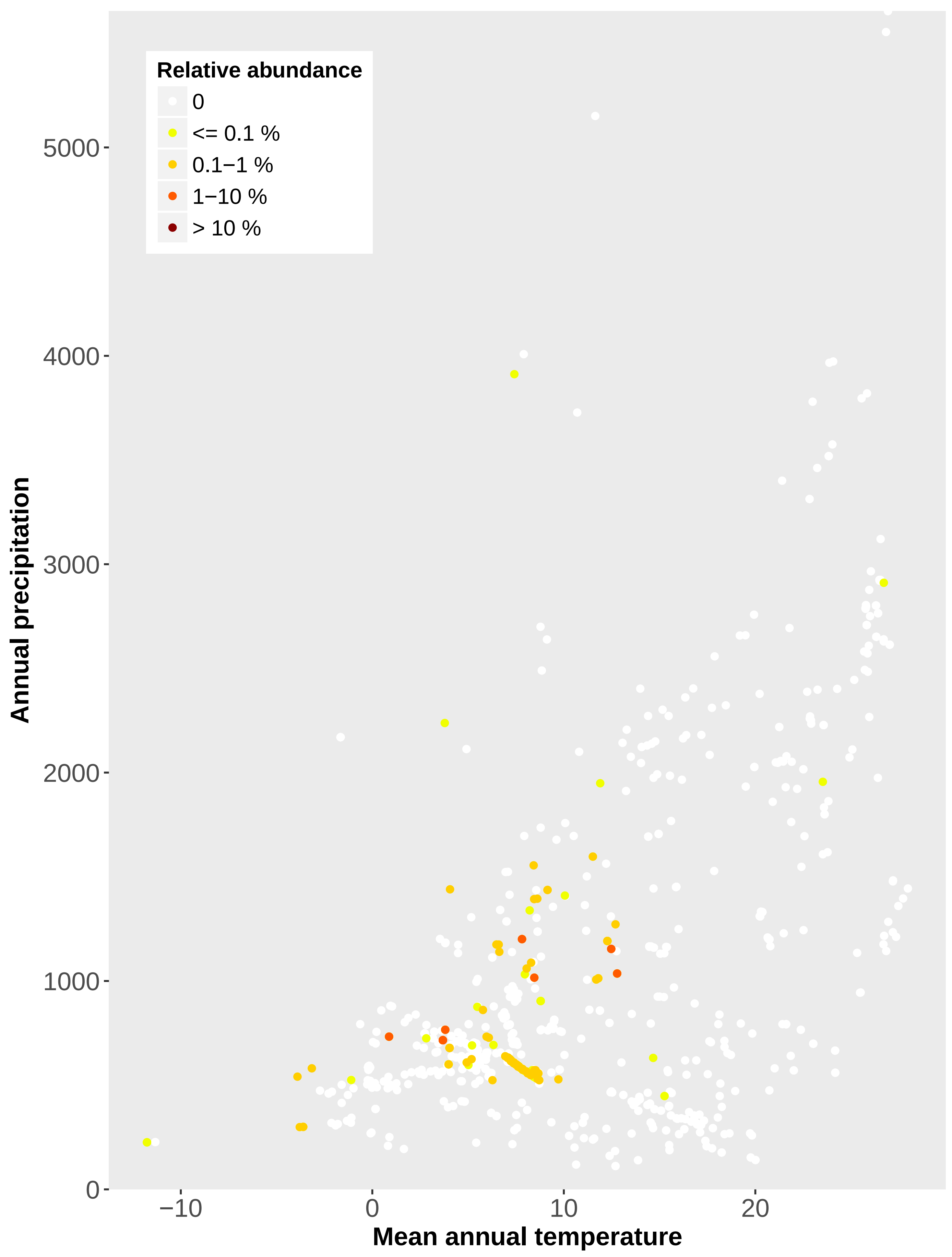

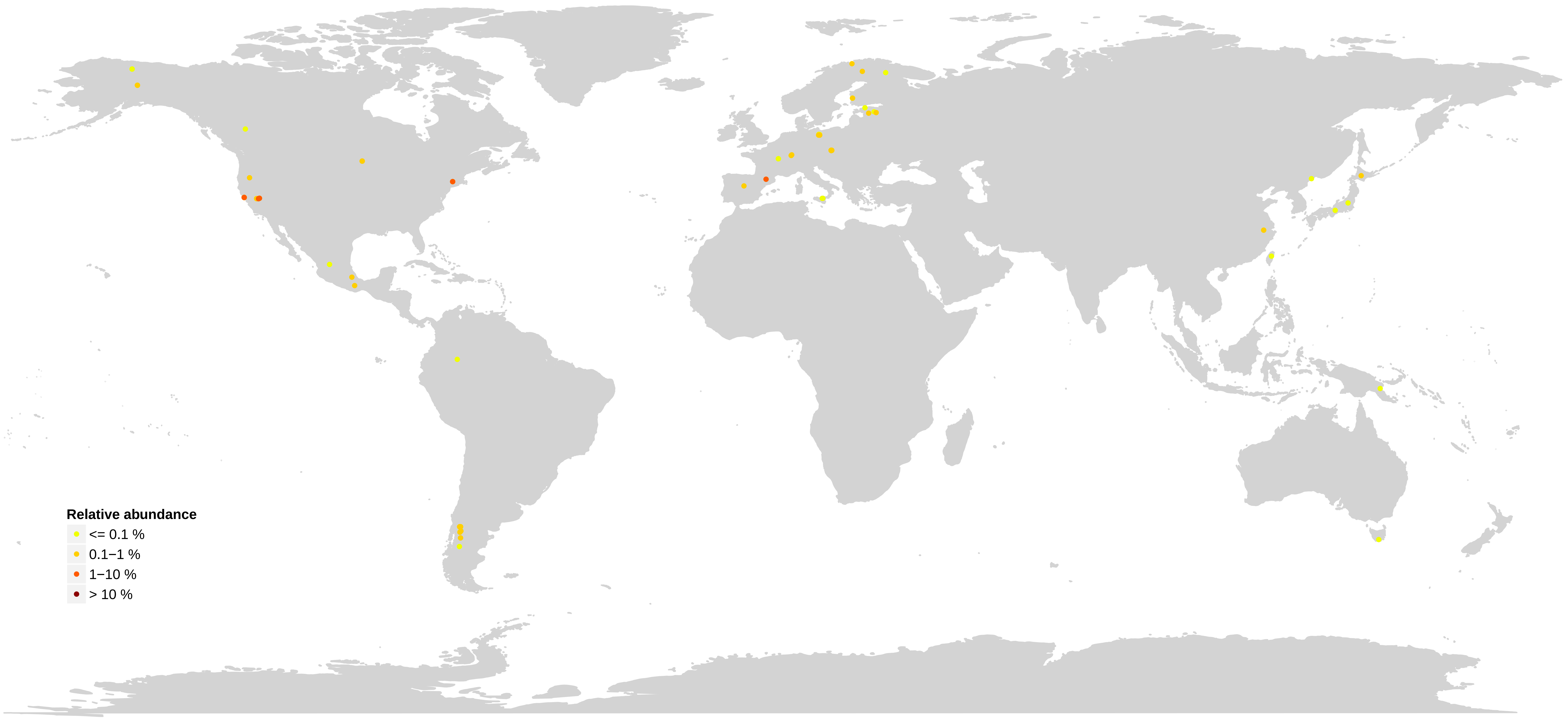

SH275913 Acephala applanata

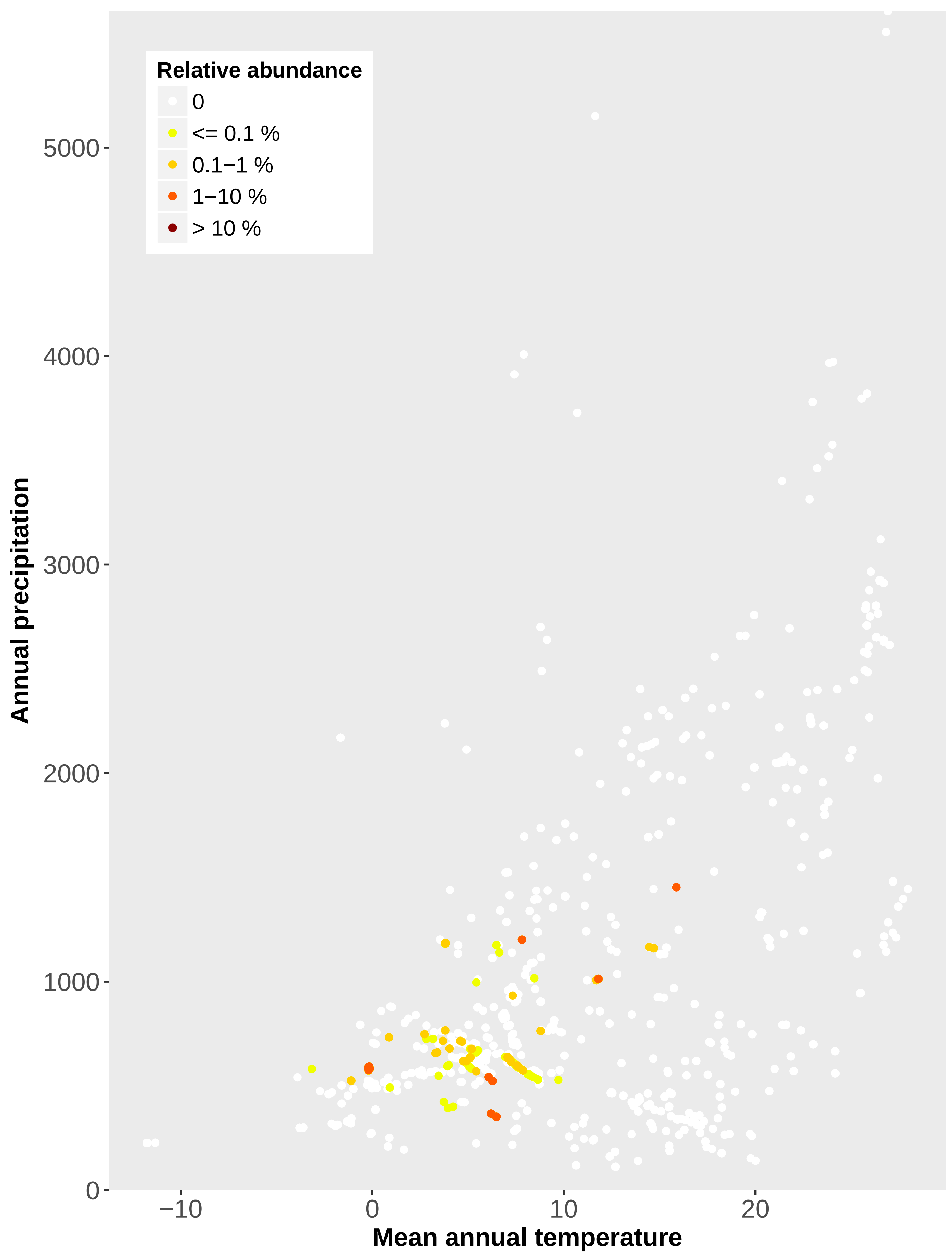

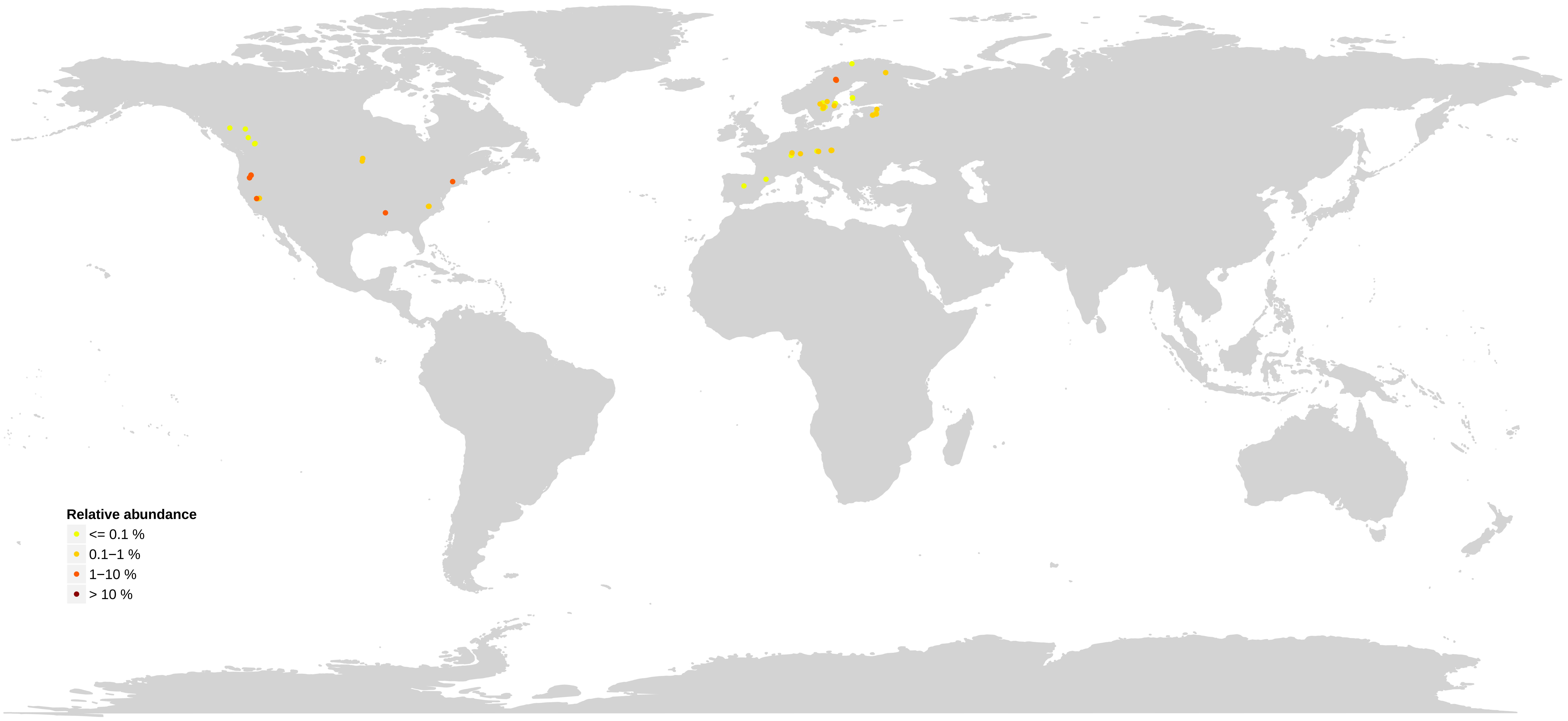

SH181078 Meliniomyces variabilis

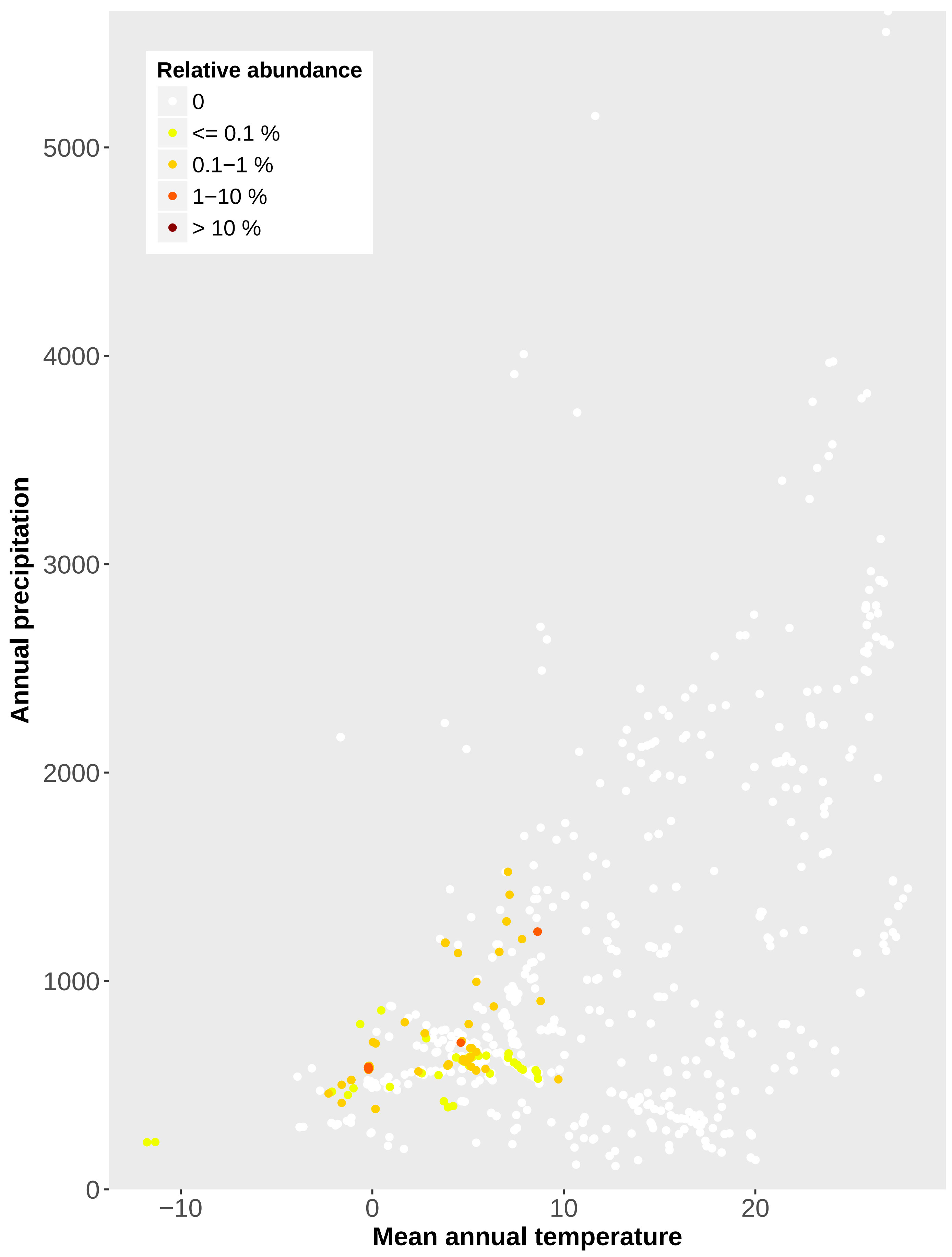

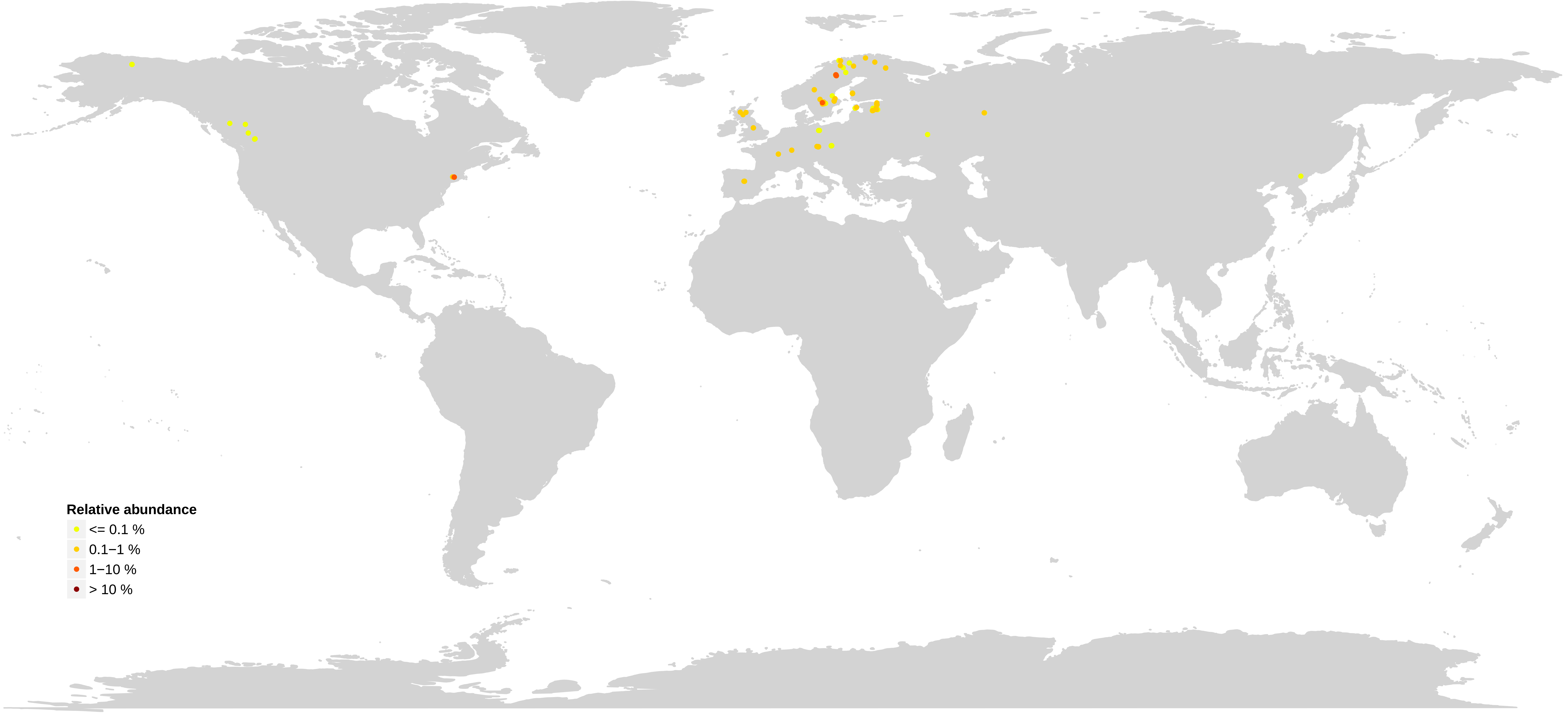

SH229381 Trichoderma minutisporum

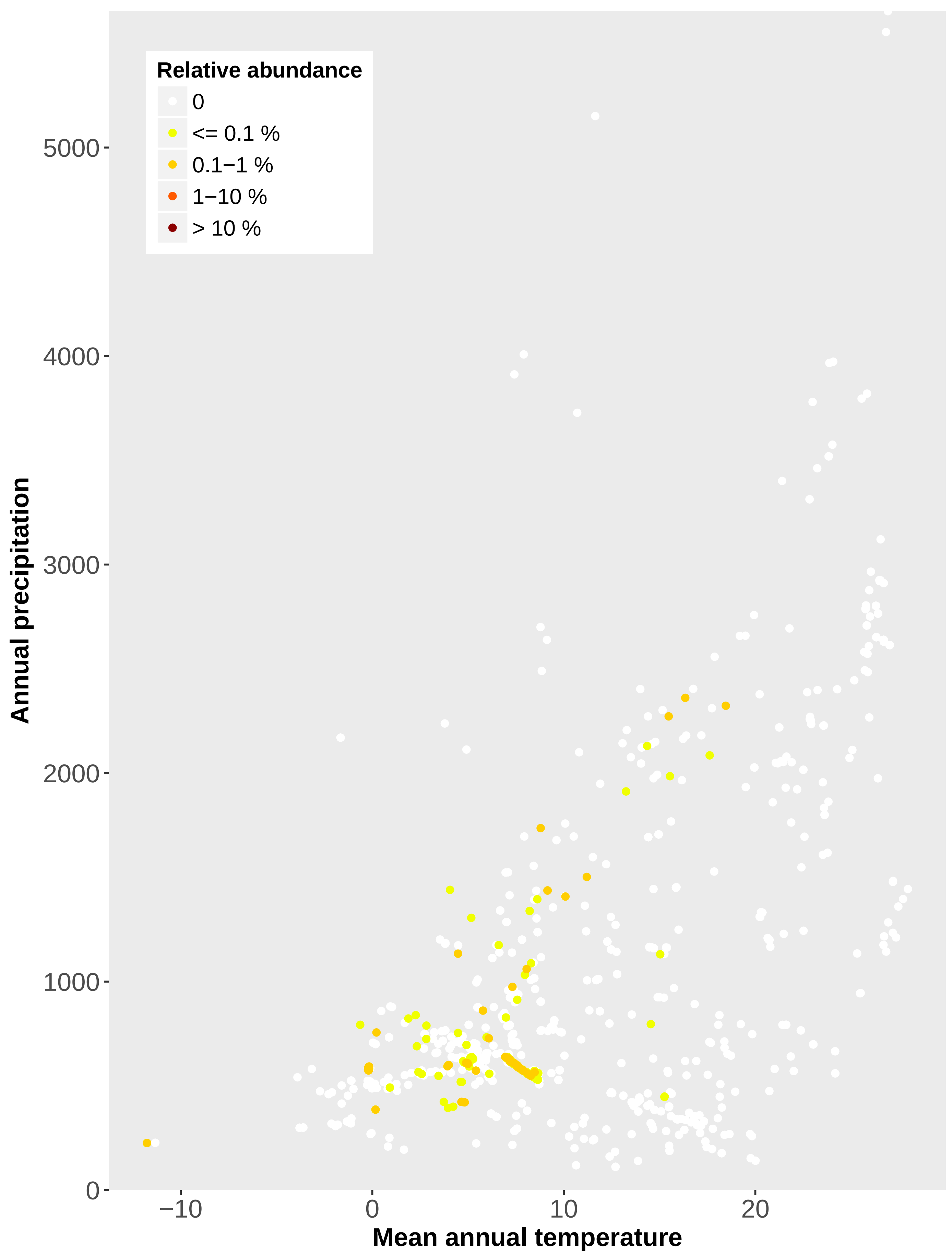

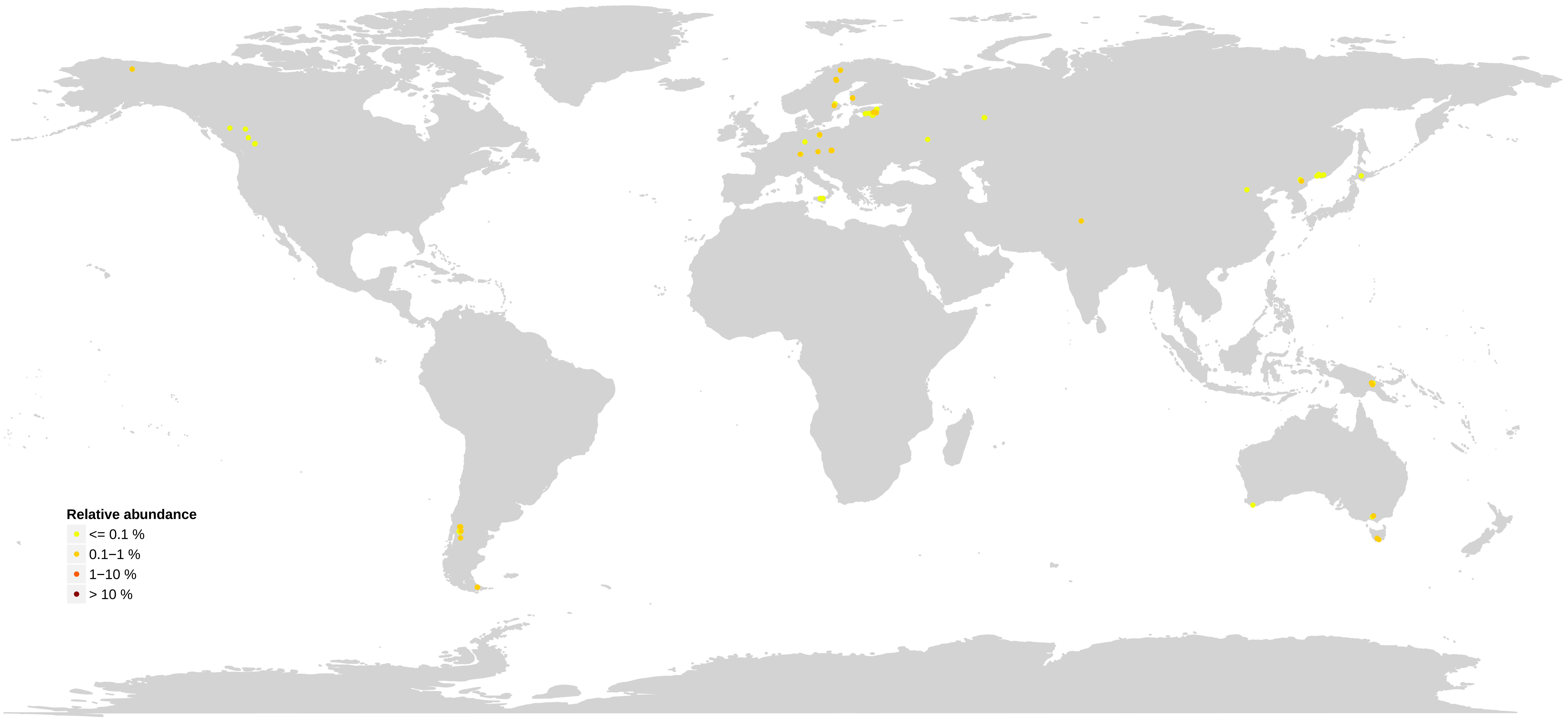

SH193247 Cladophialophora sylvestris

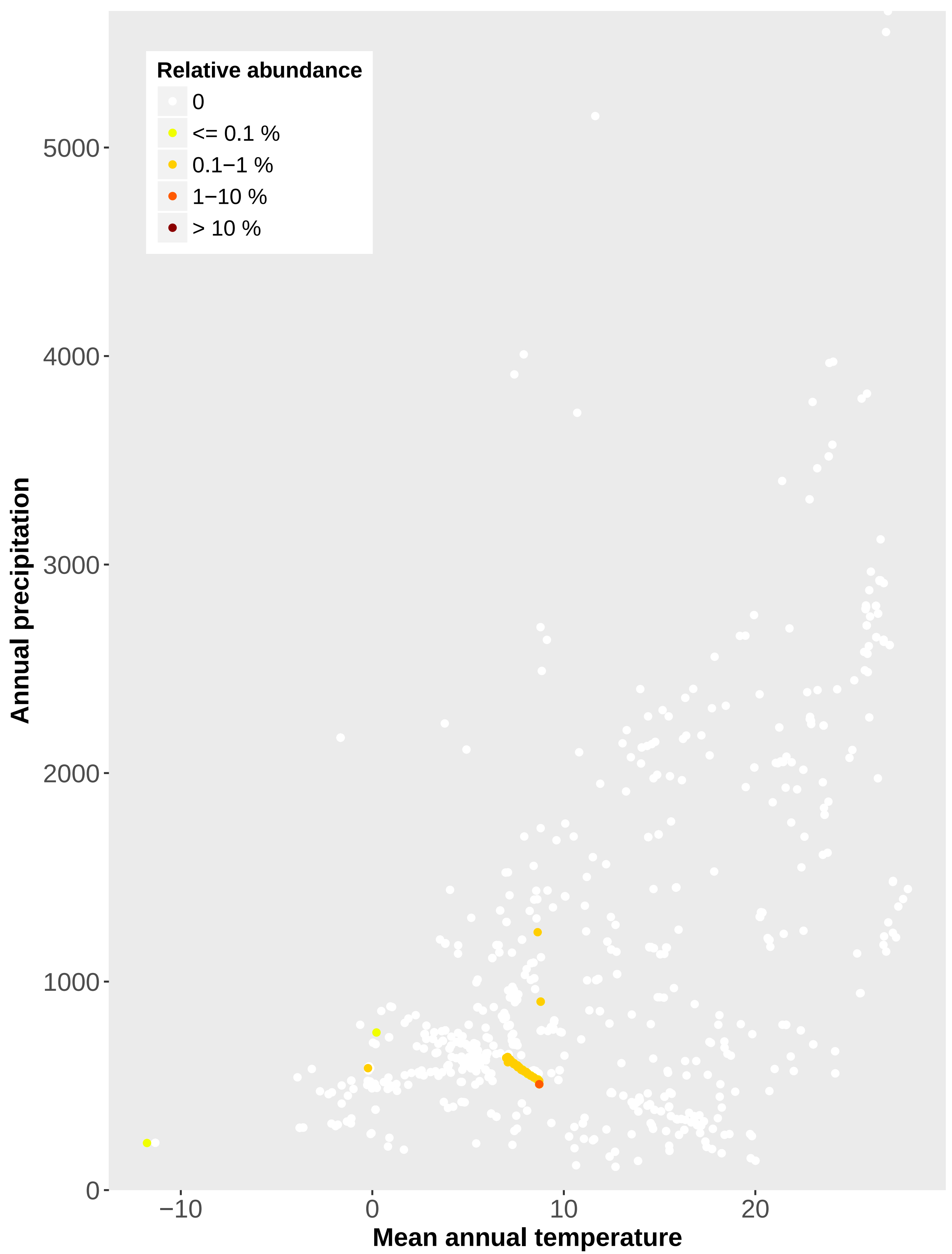

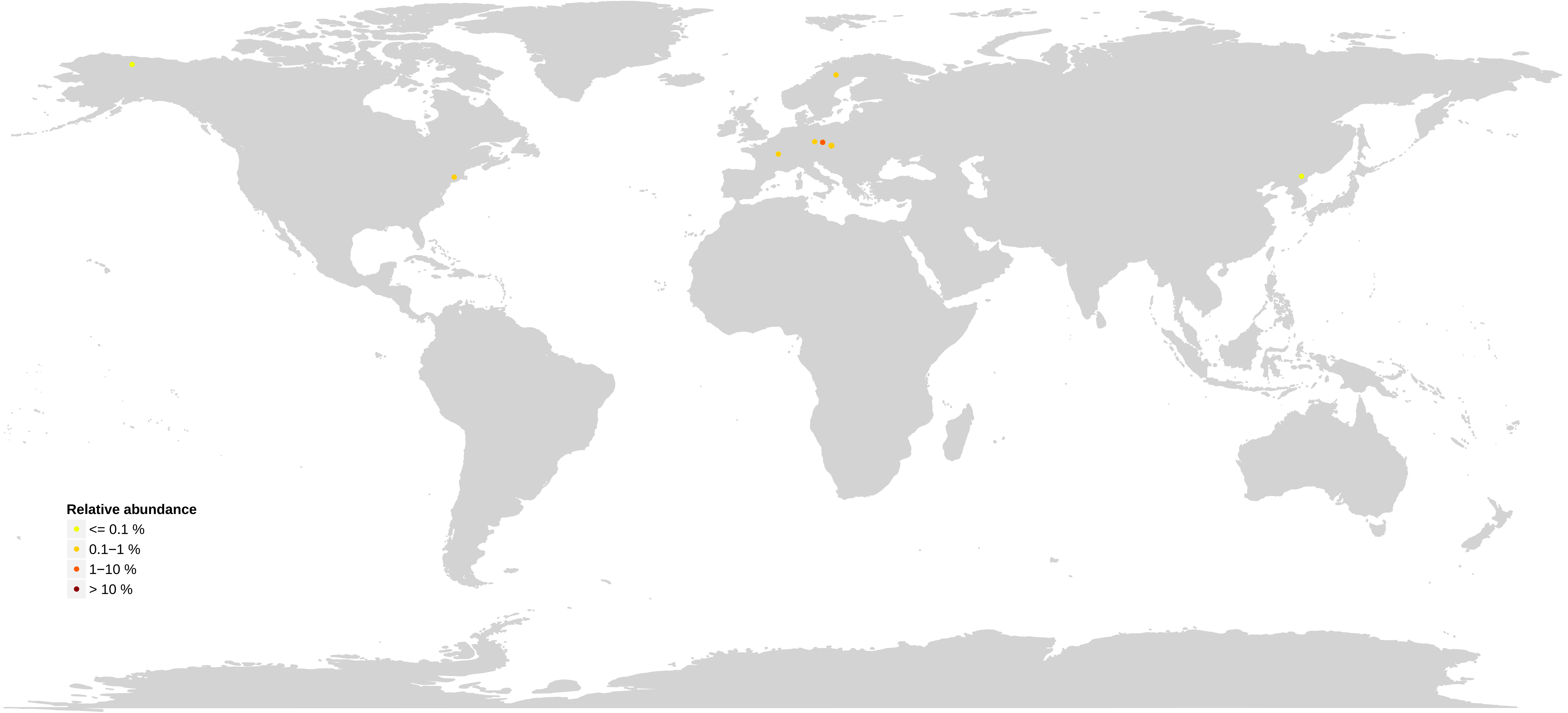

SH202967 Ilyonectria mors−panacis

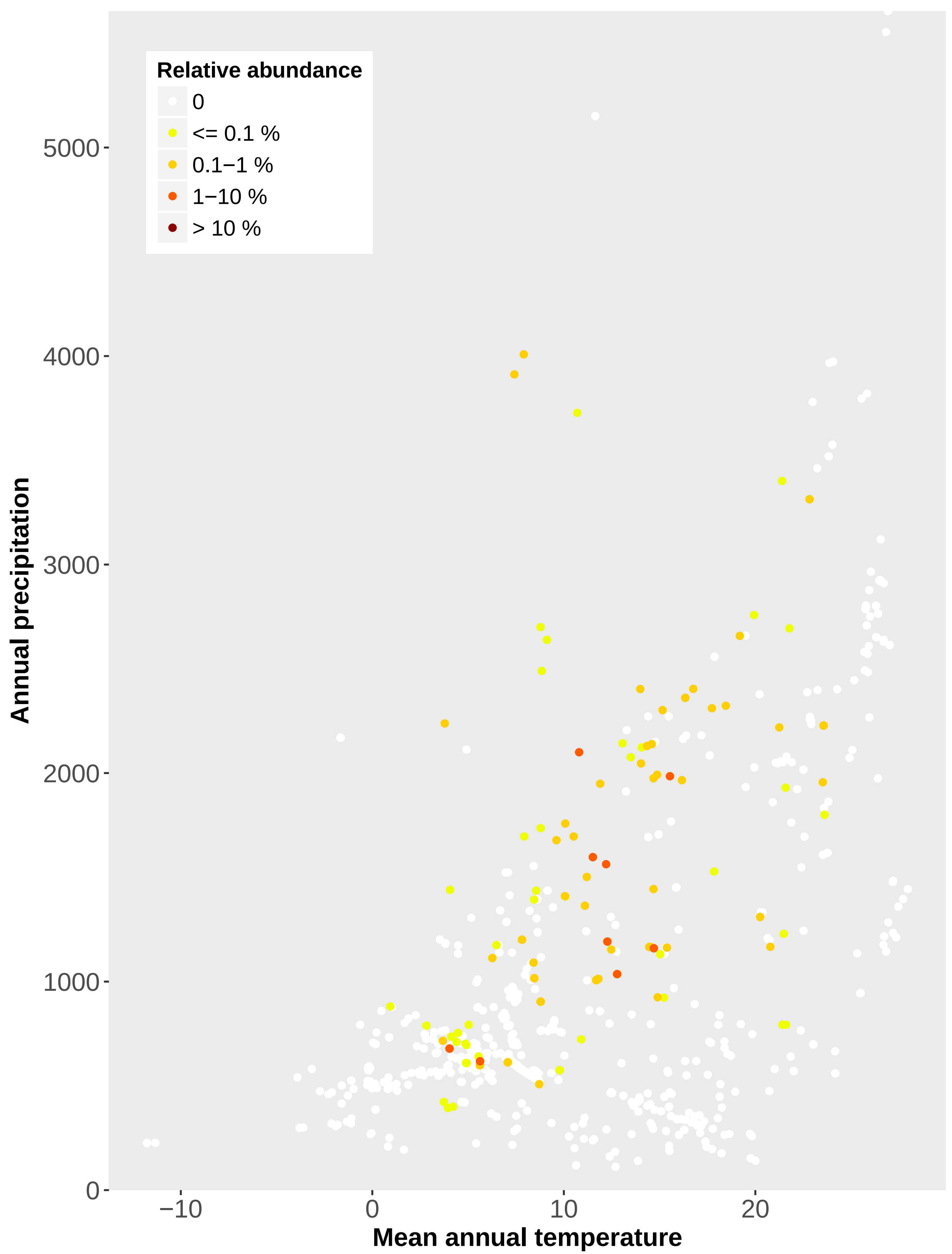

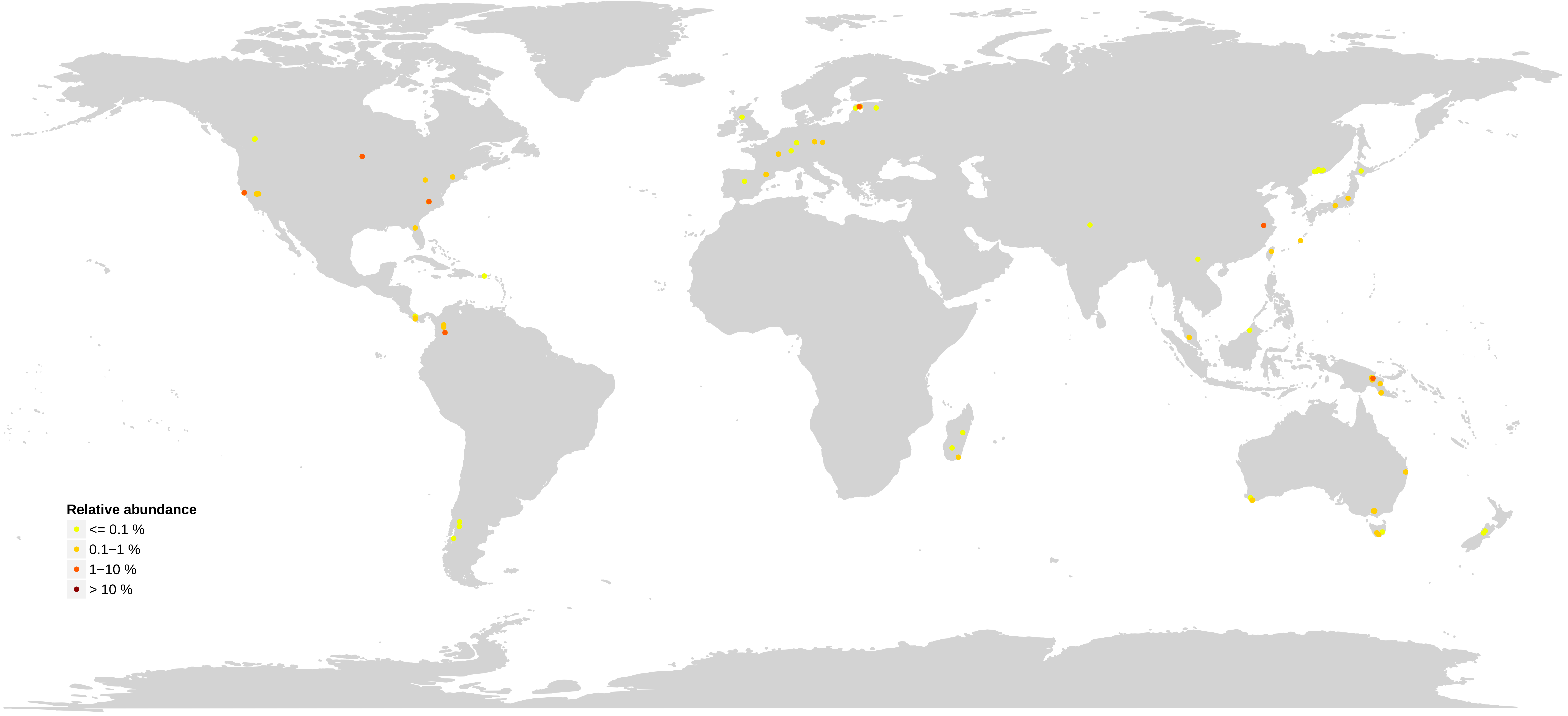

SH207011 Pleosporales sp

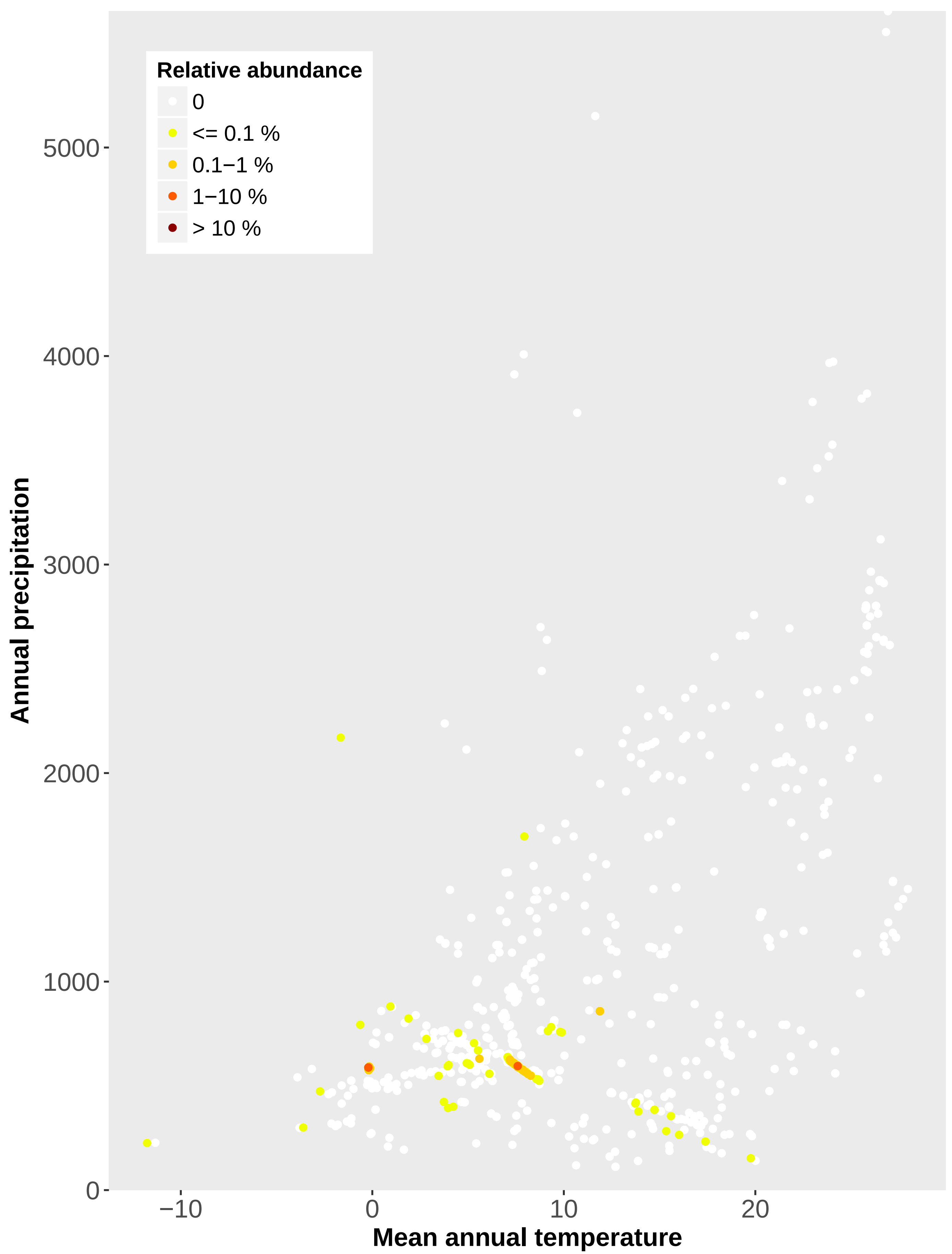

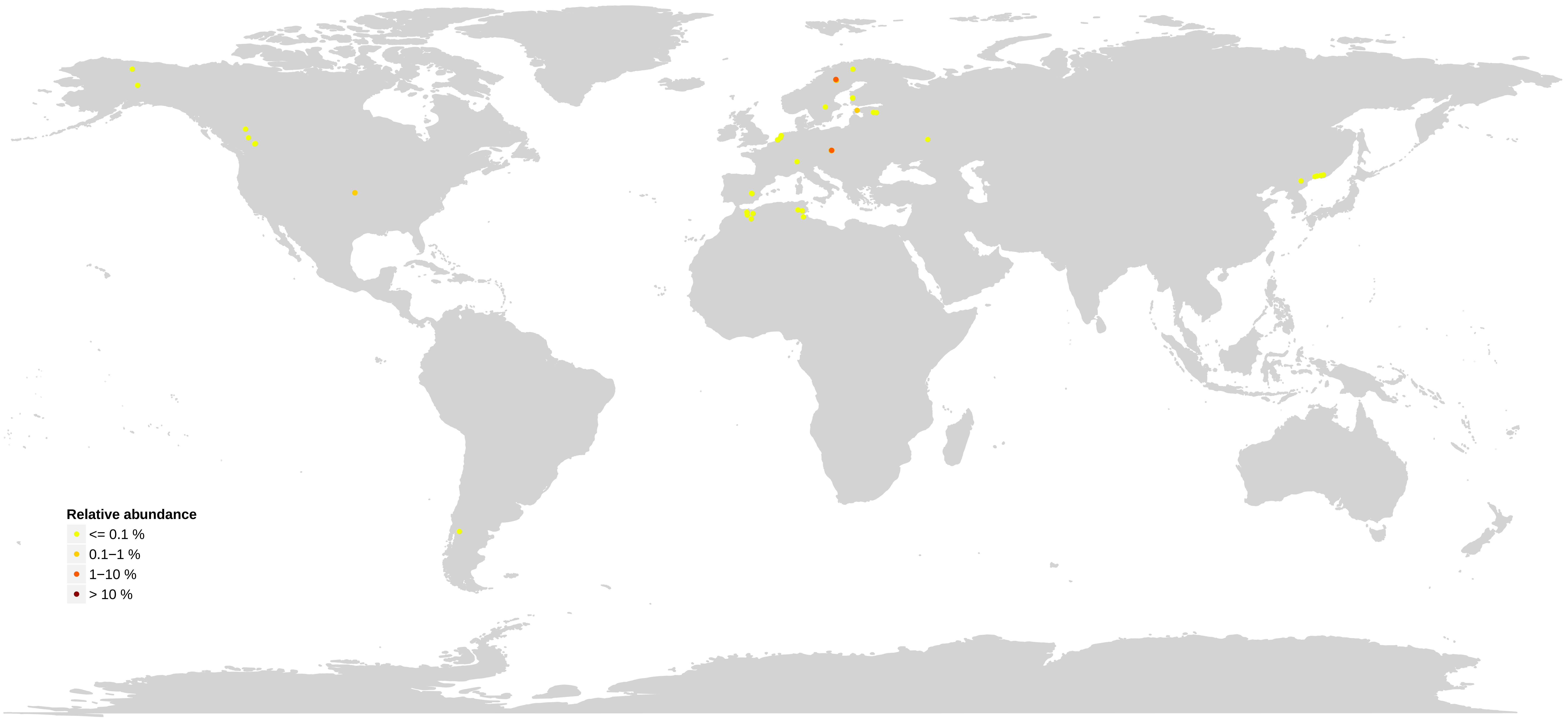

SH005081 Tremellomycetes sp

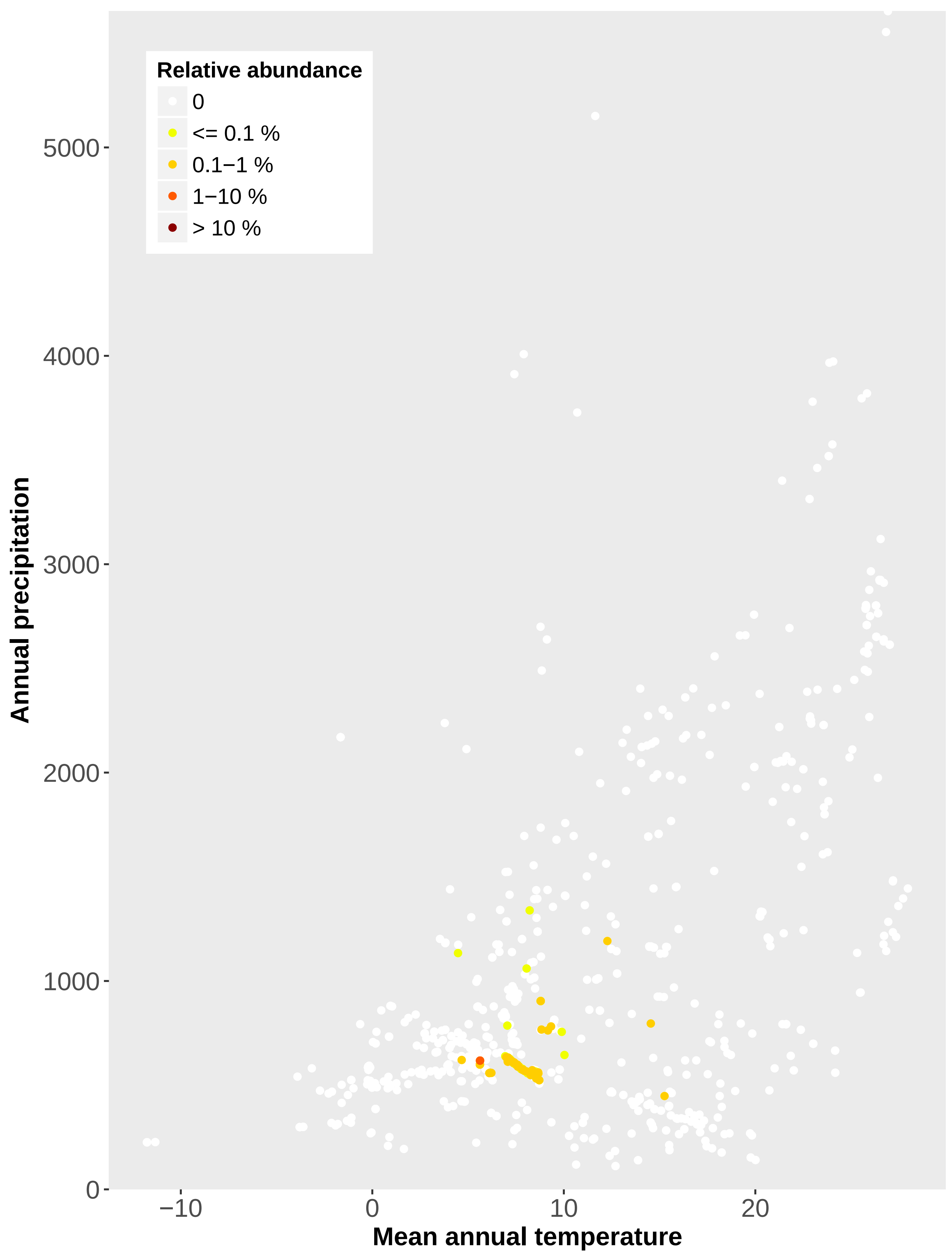

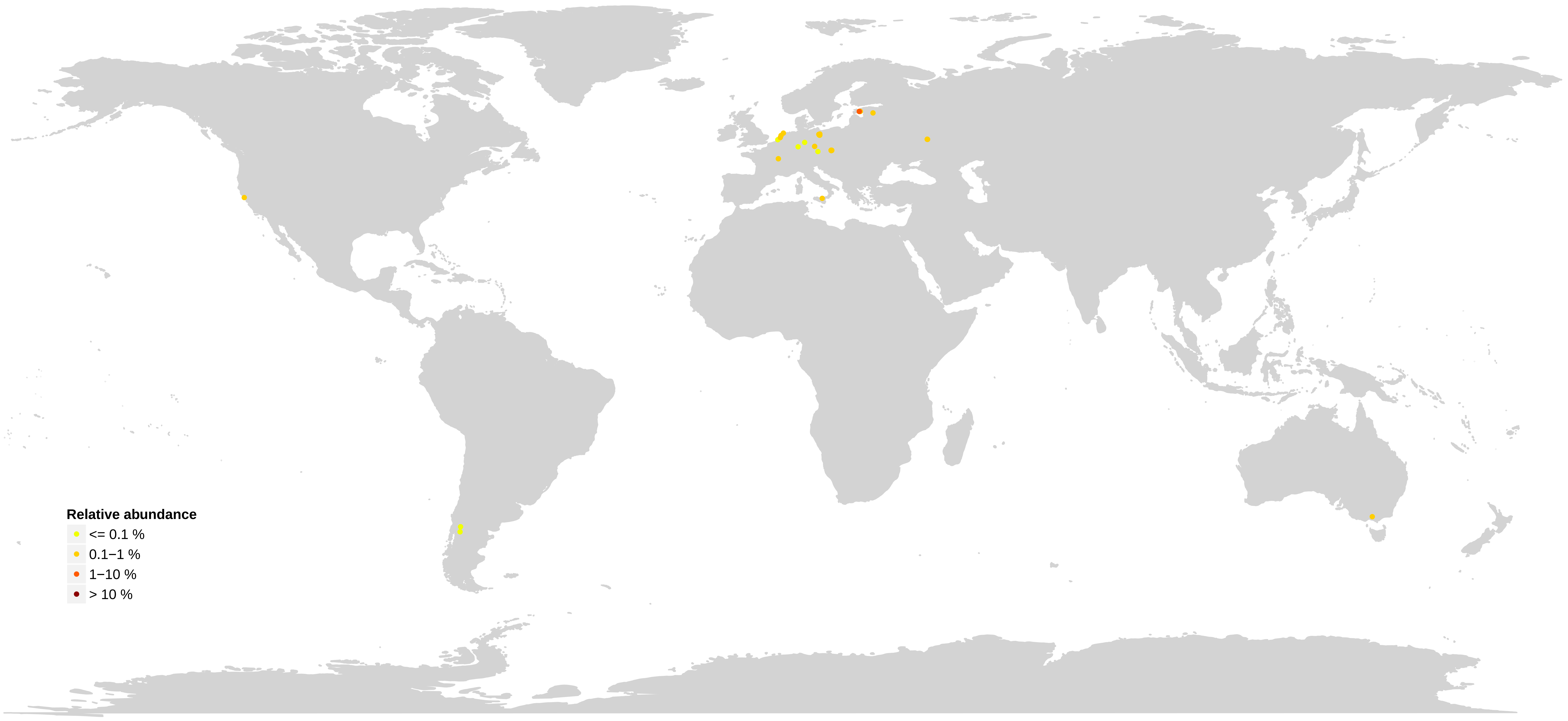

SH014364 Trichoderma semiorbis

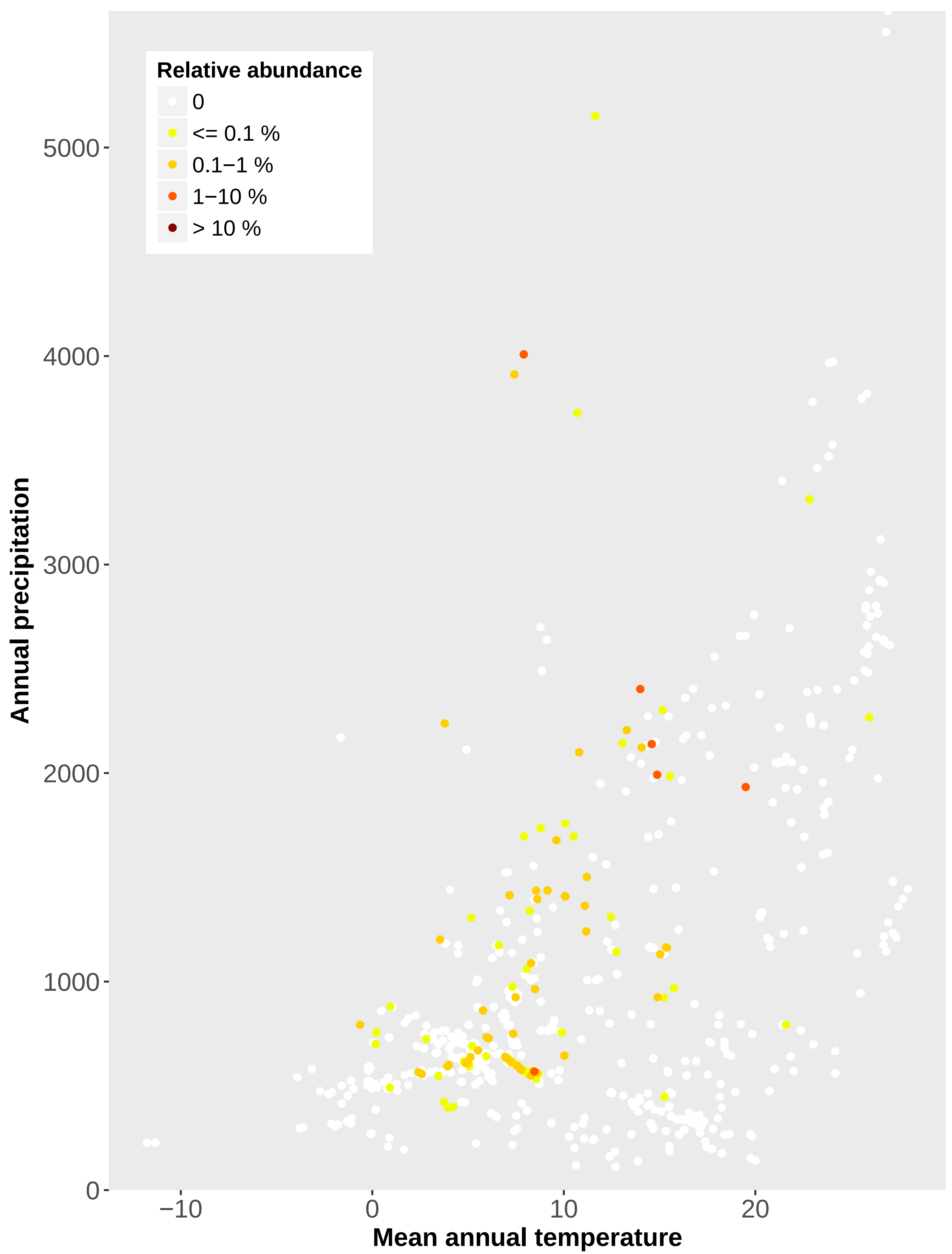

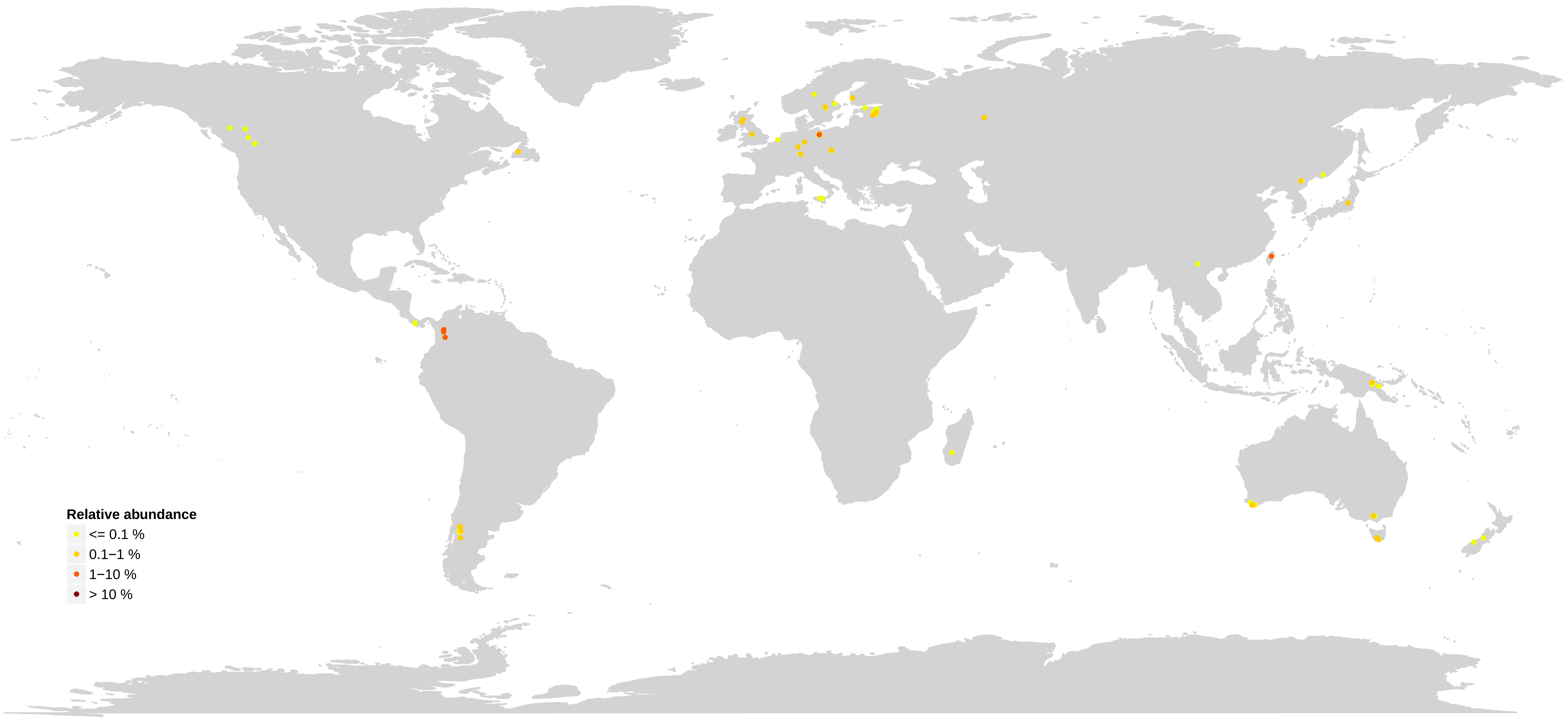

SH180116 Mortierella sclerotiella

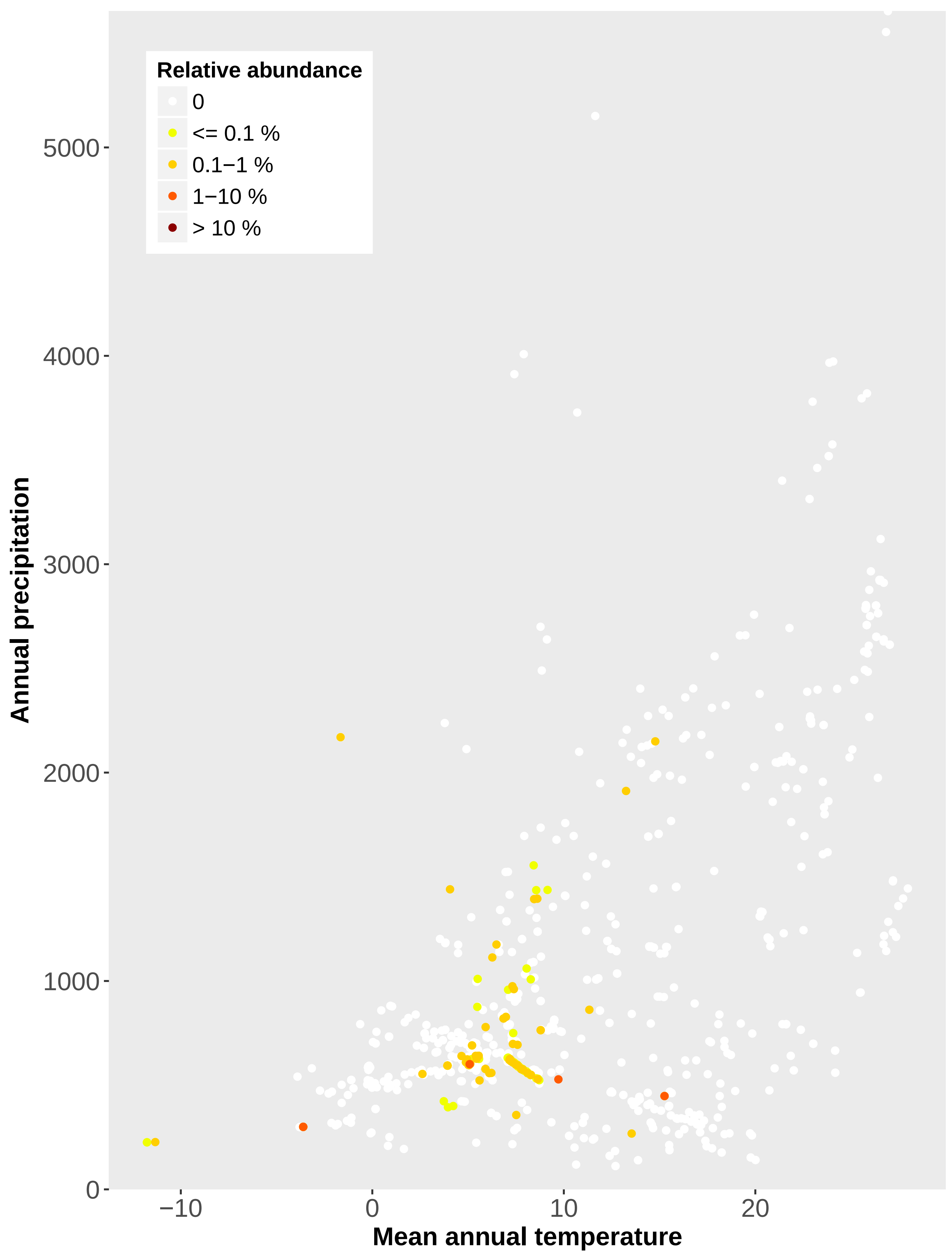

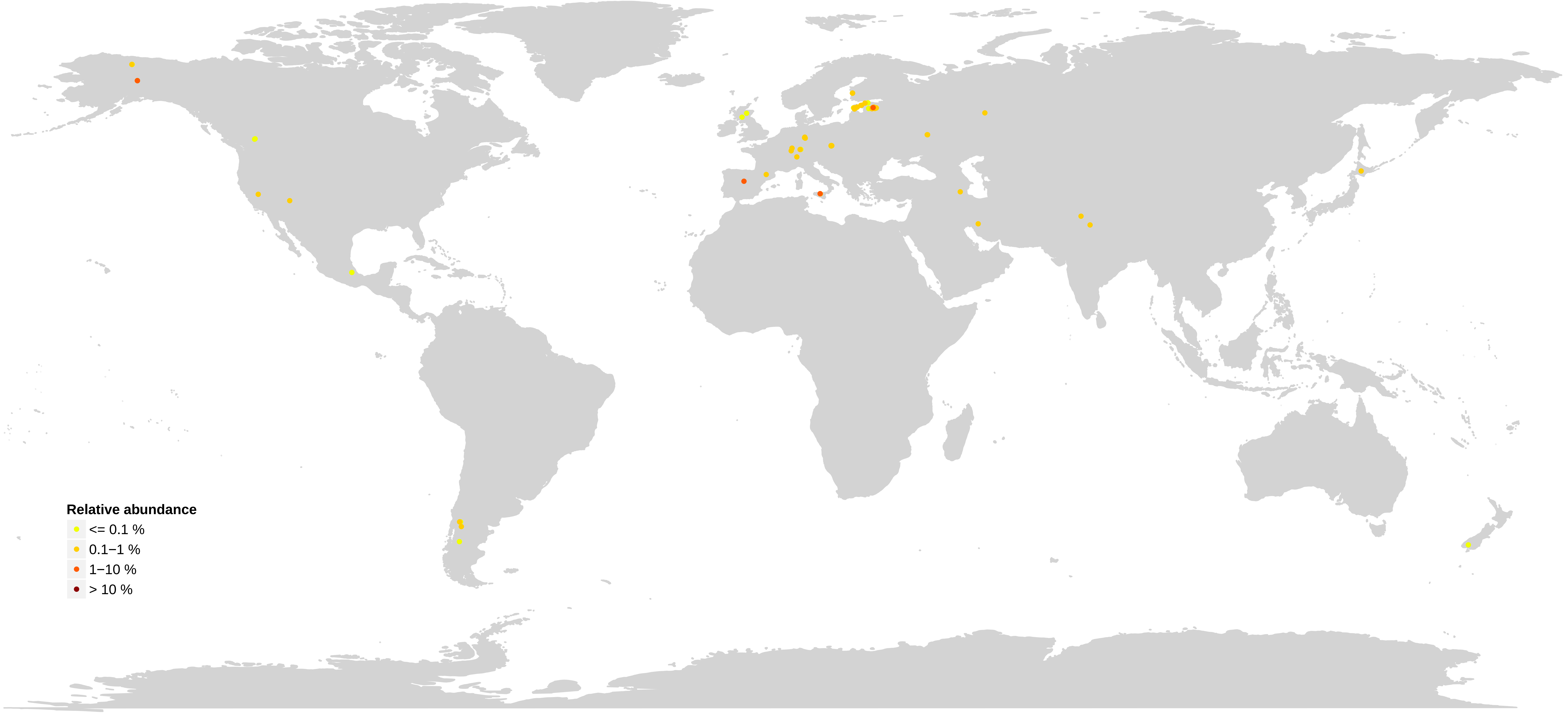

SH229375 Trichoderma stellatum

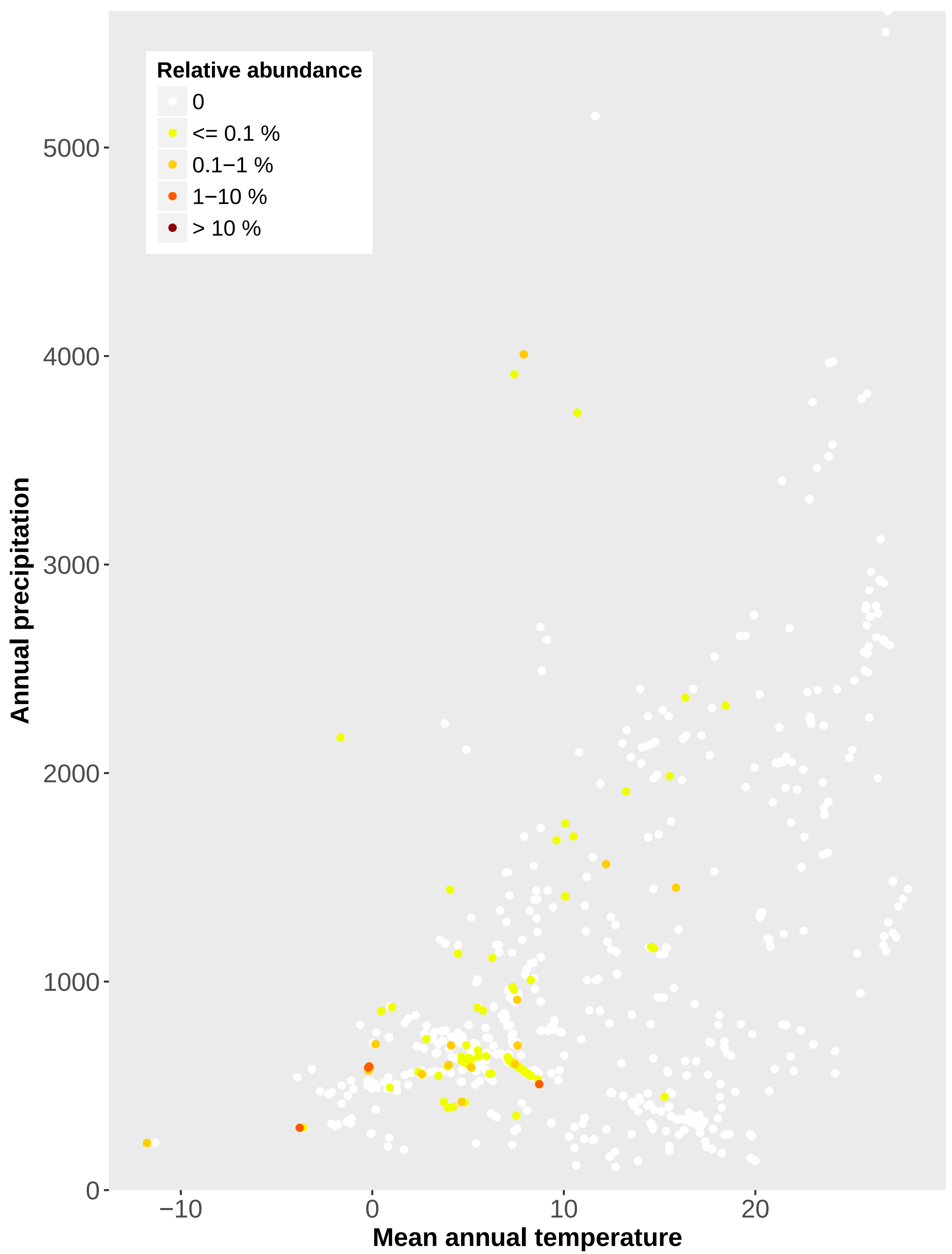

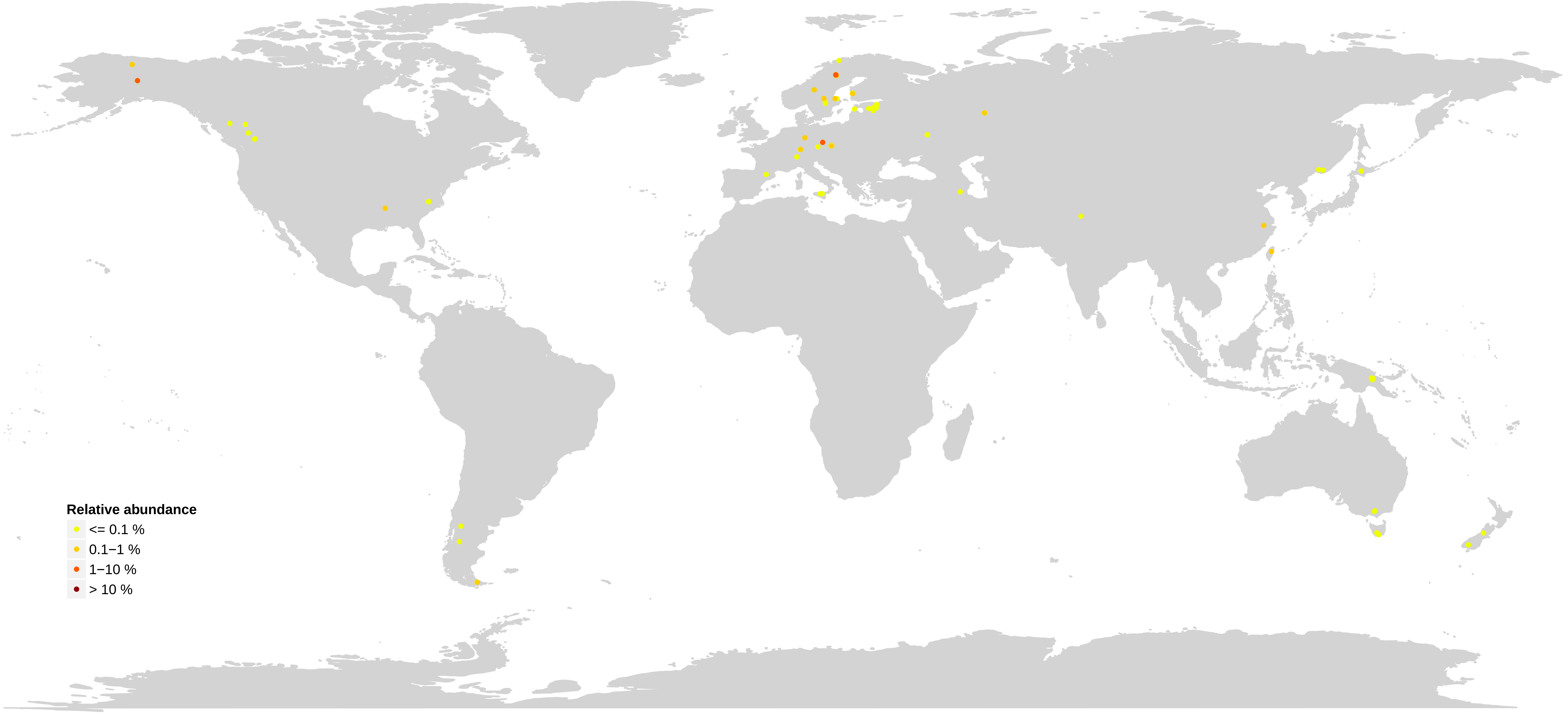

SH211202 Bionectriaceae sp

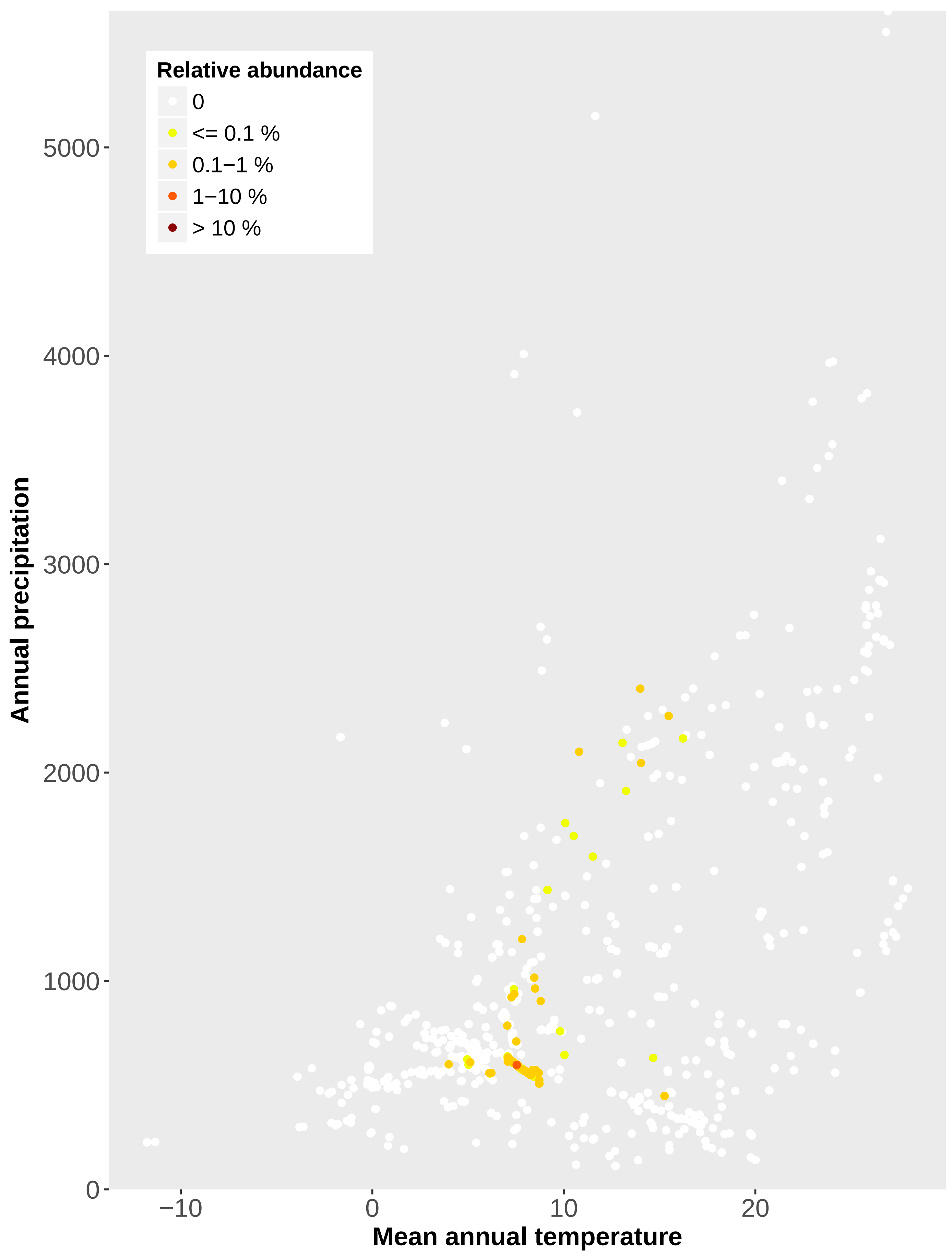

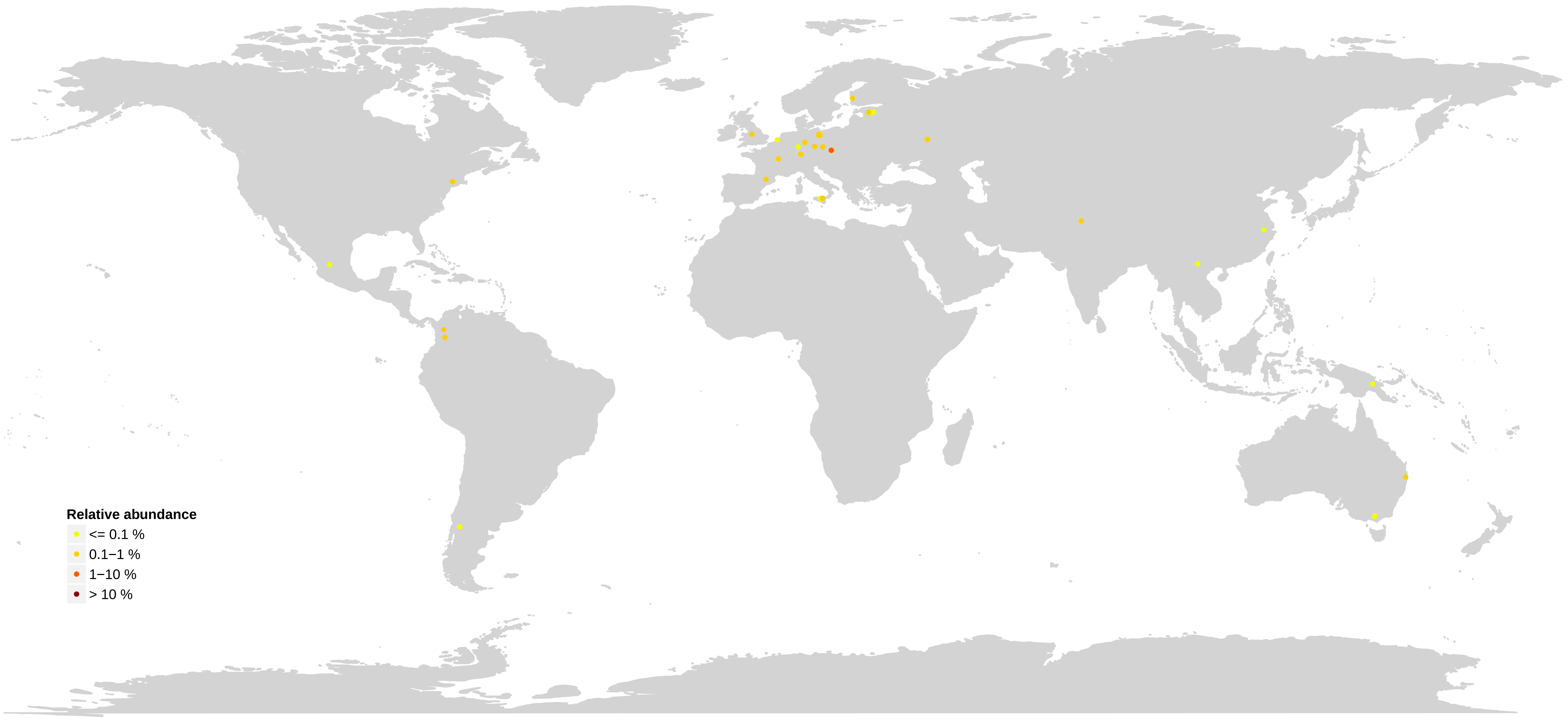

SH213394 Mortierellales sp

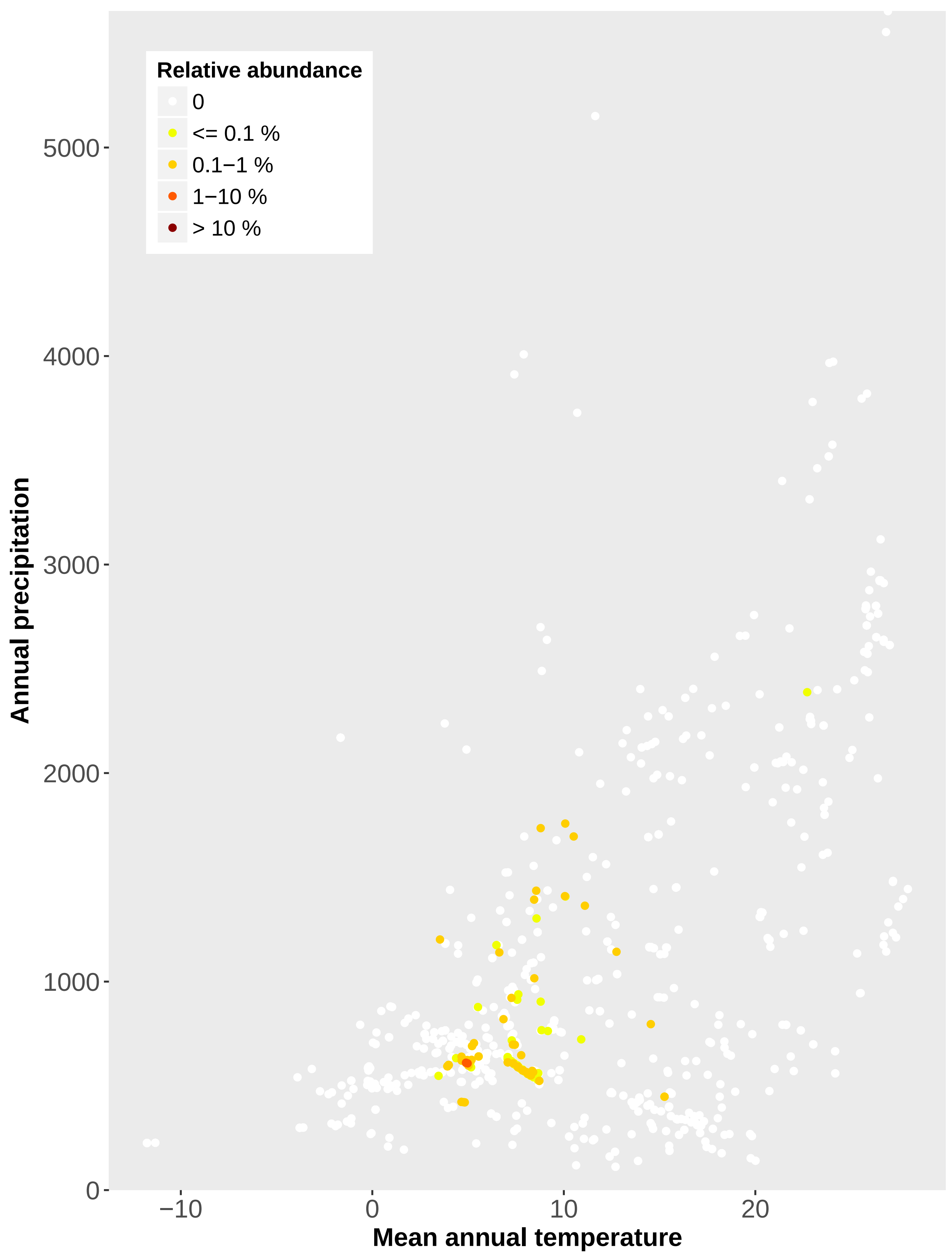

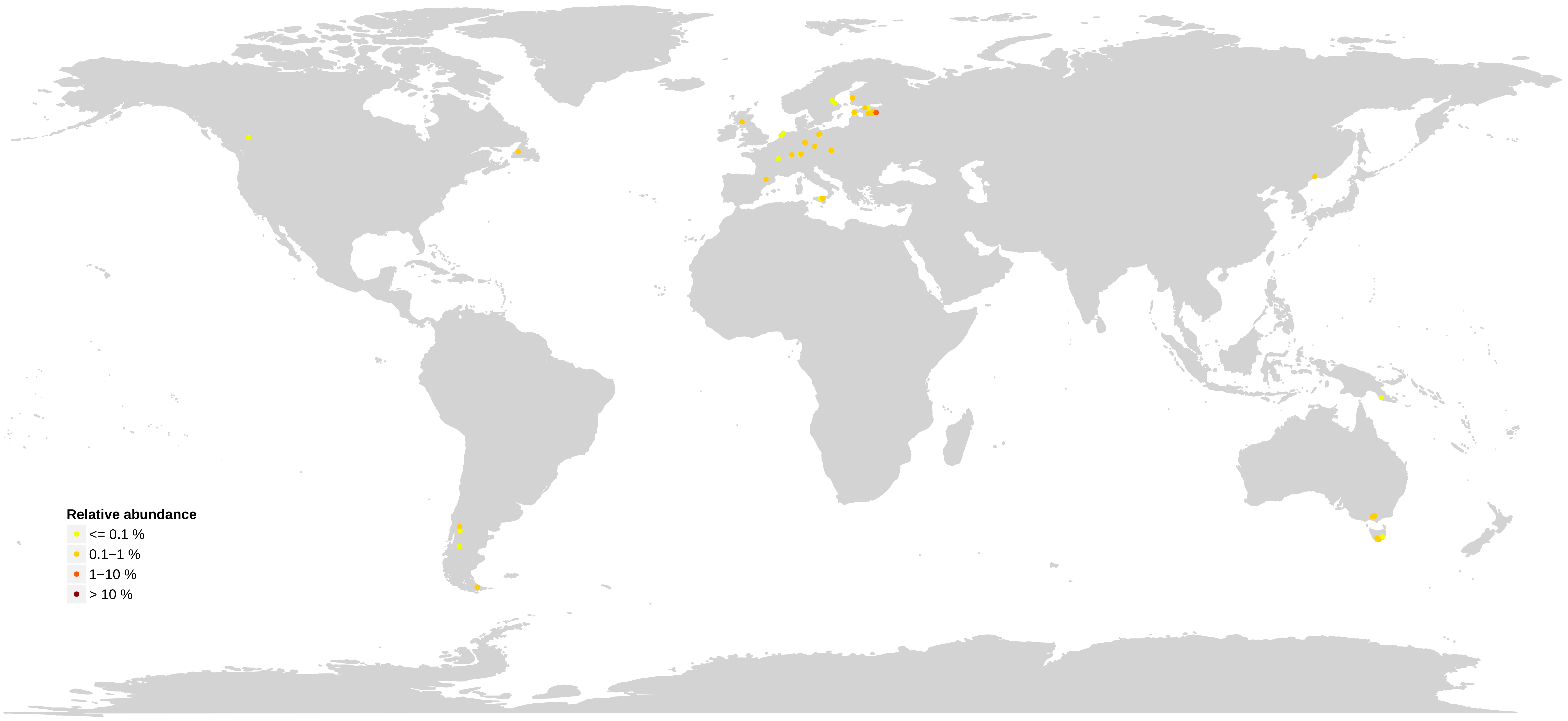

SH196643 Trichosporon moniliiforme

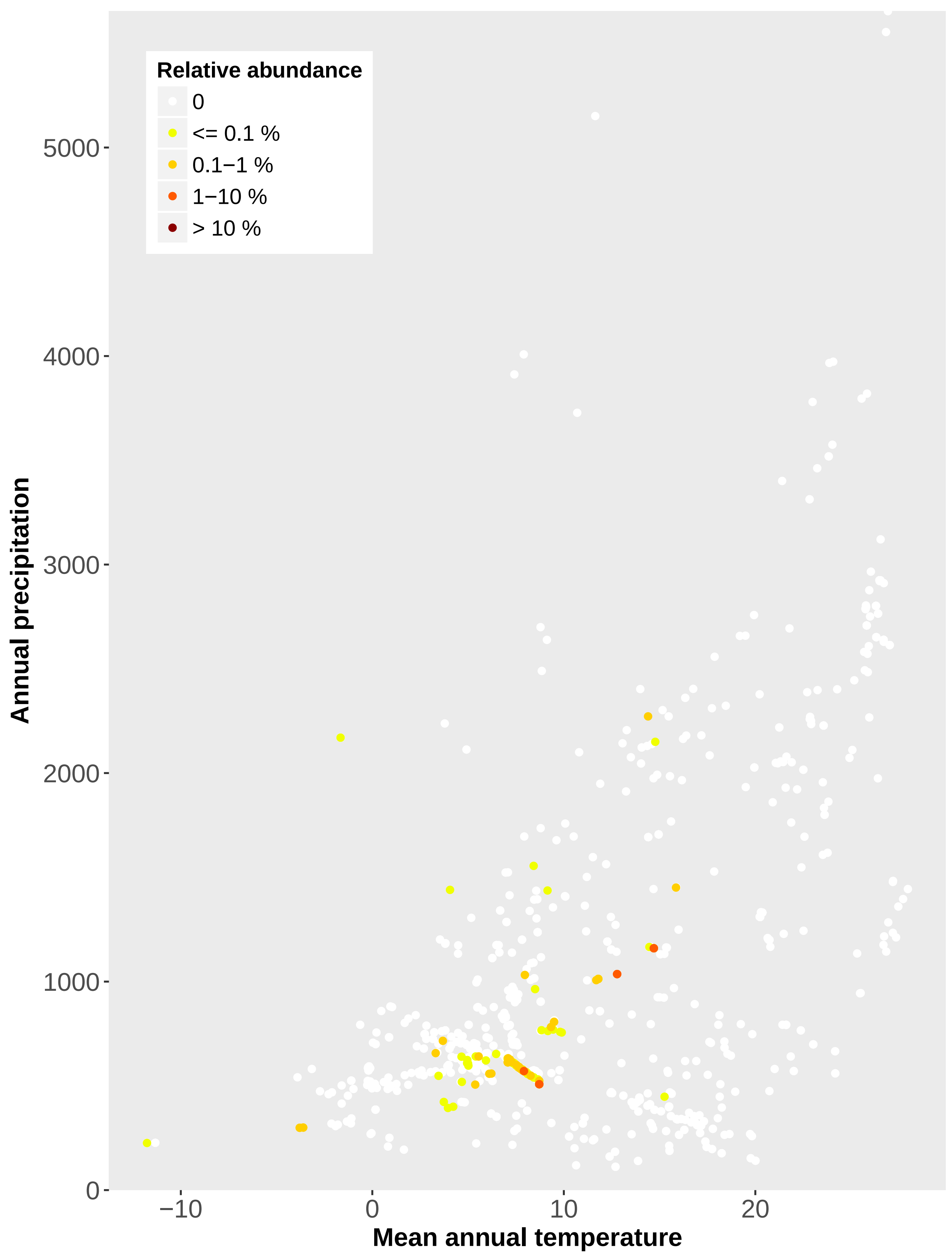

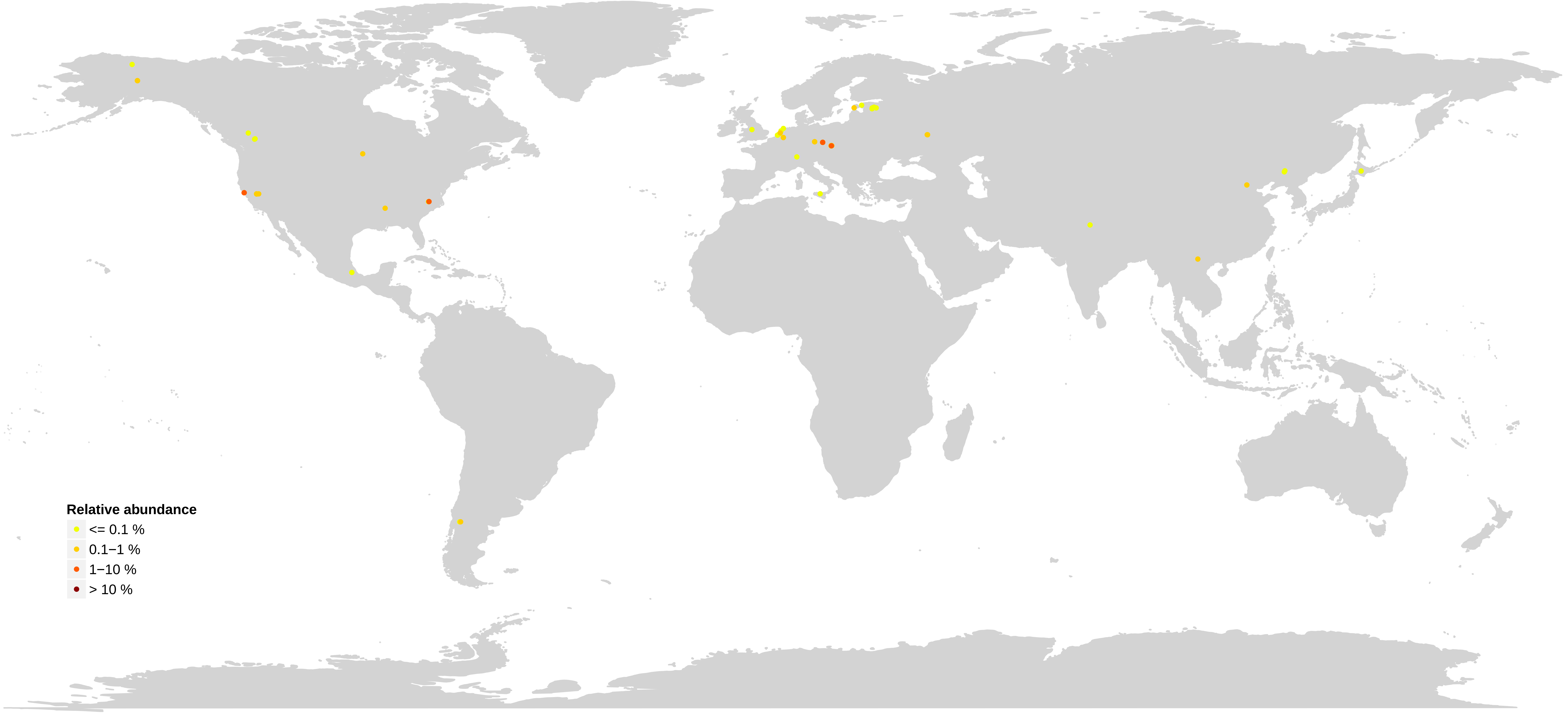

SH195345 Humicola sp

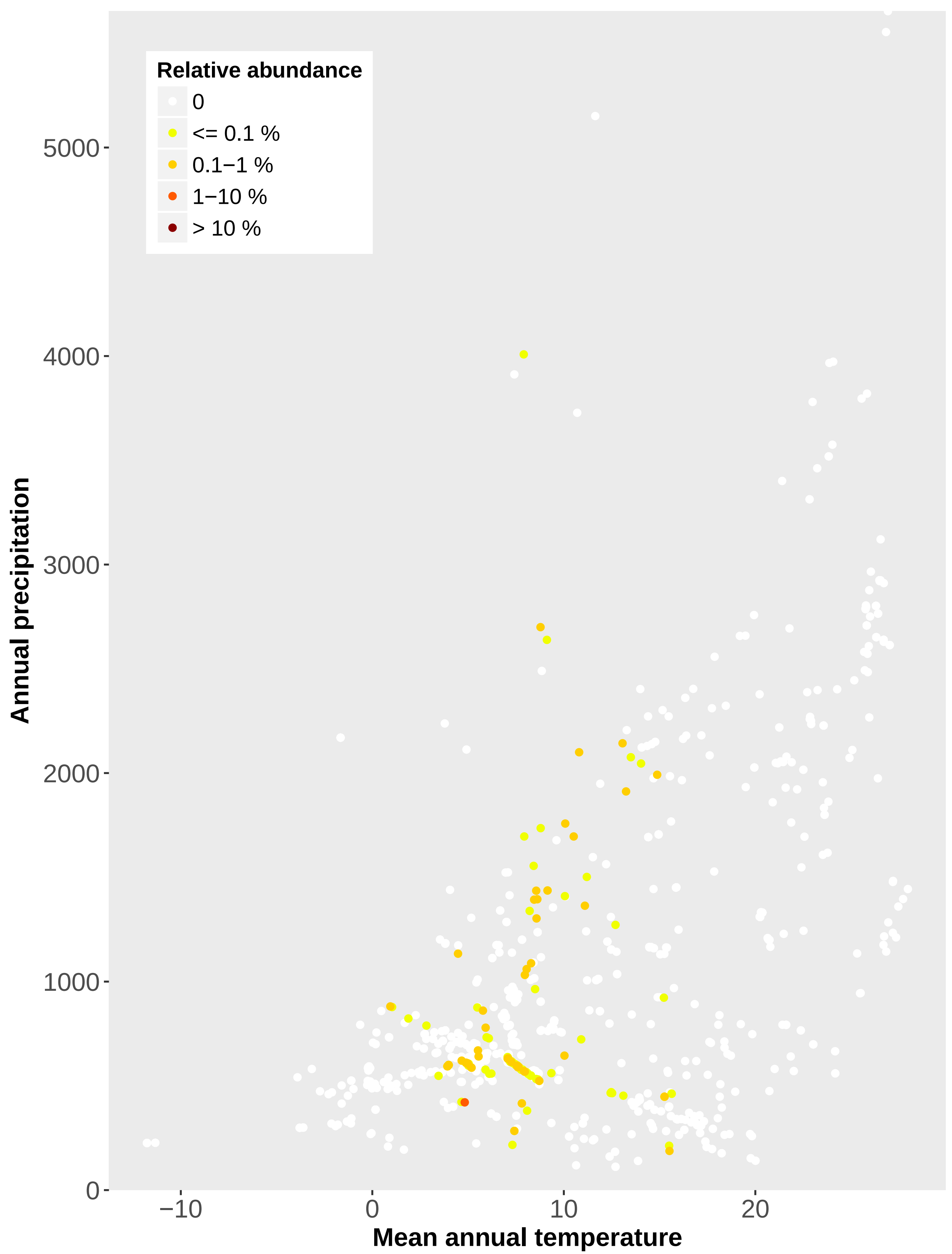

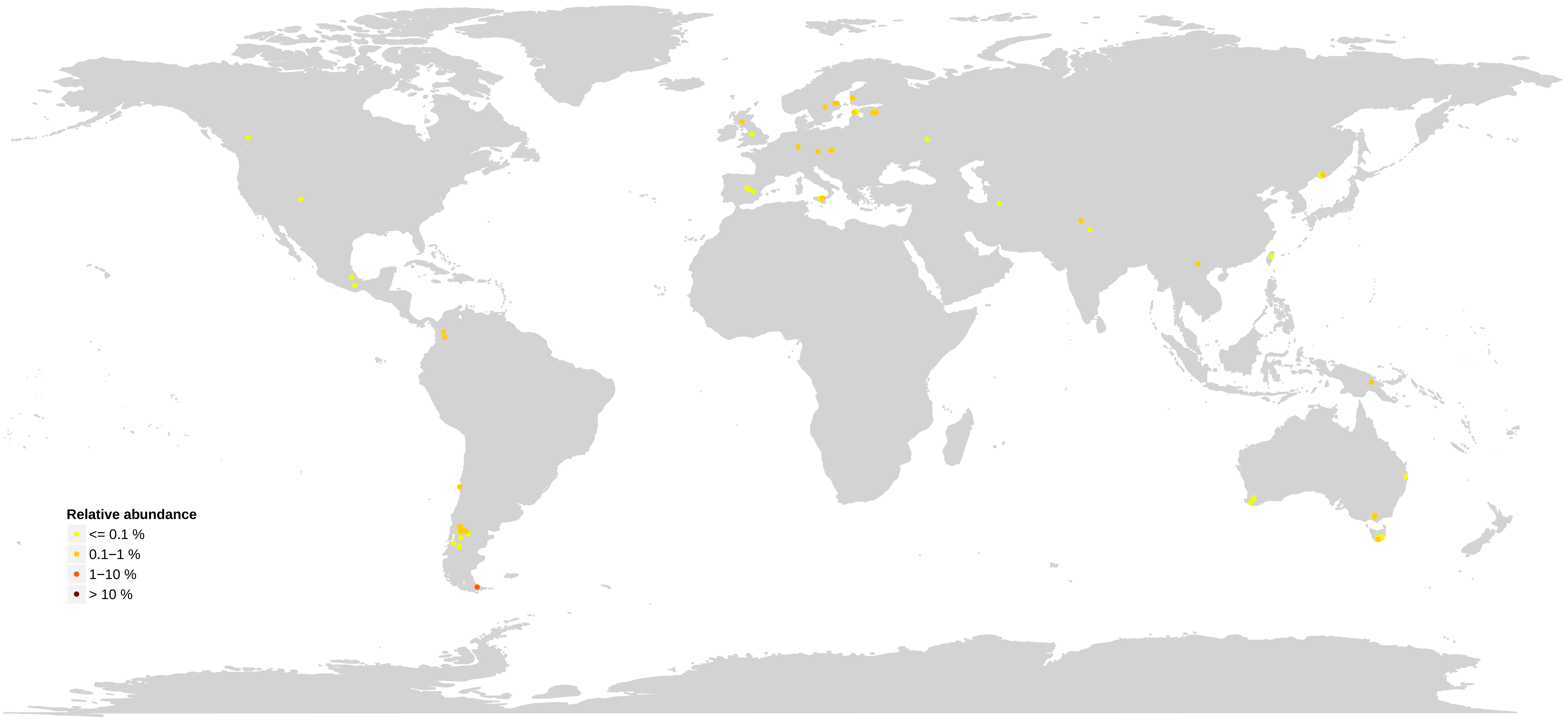

SH203497 Ascomycota sp

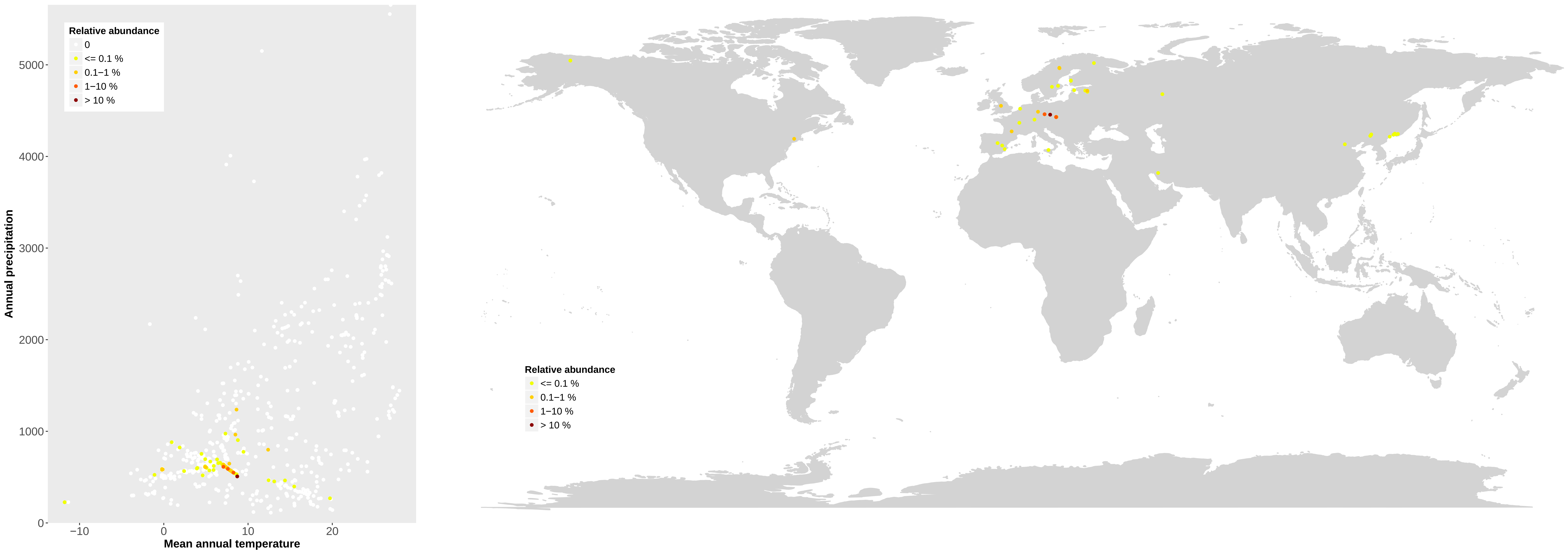

SH181585 Chaetosphaeria sp

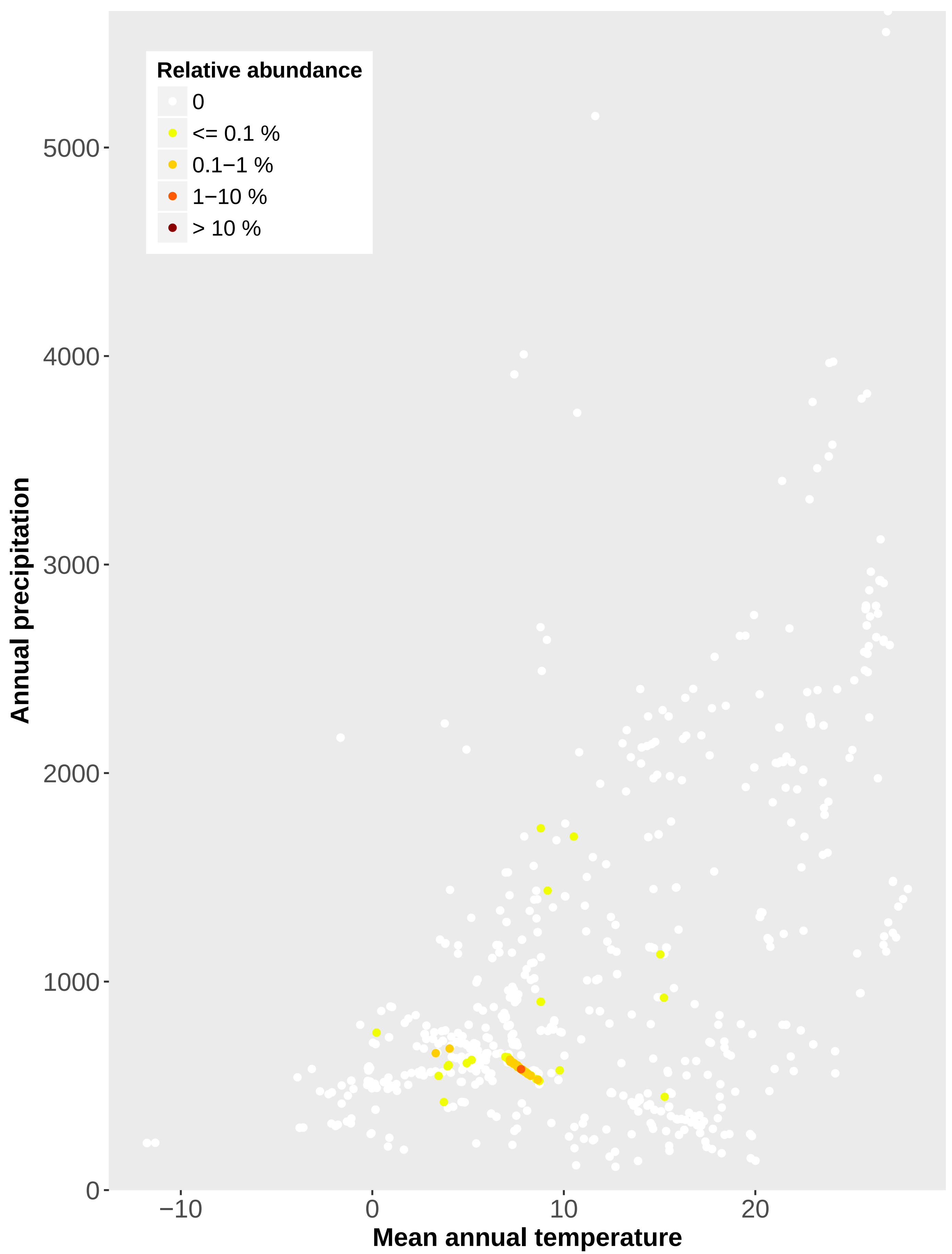

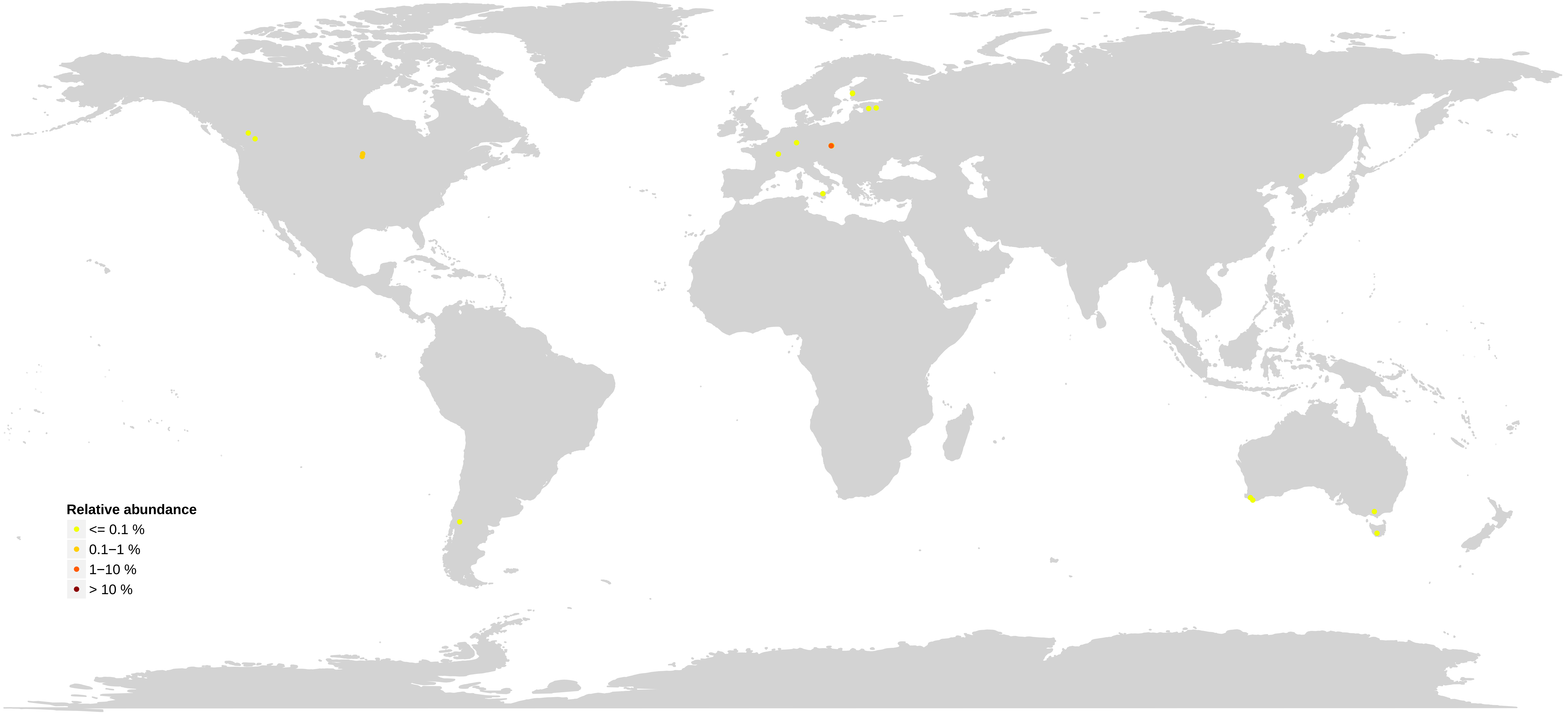

SH180664 Microbotryomycetes sp

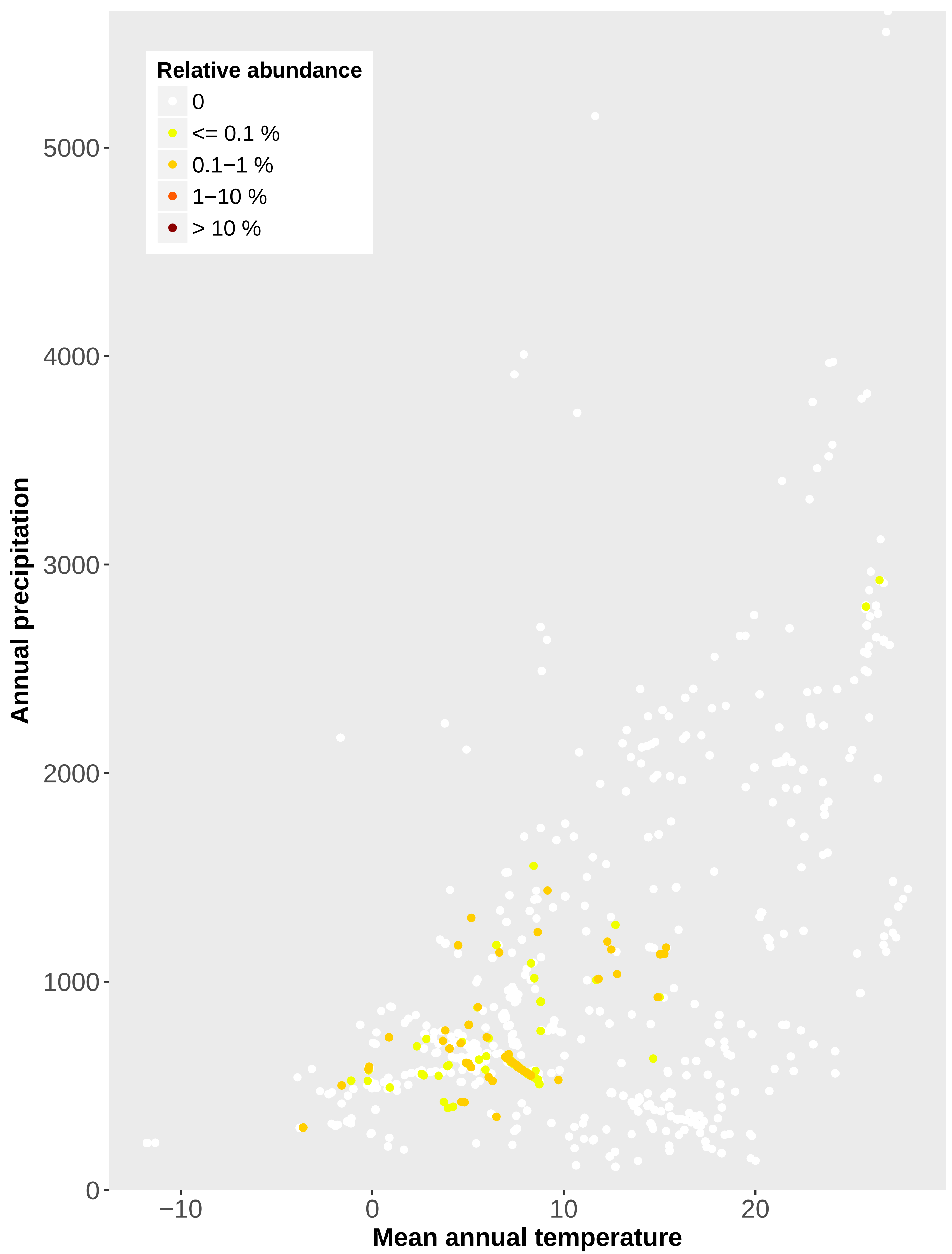

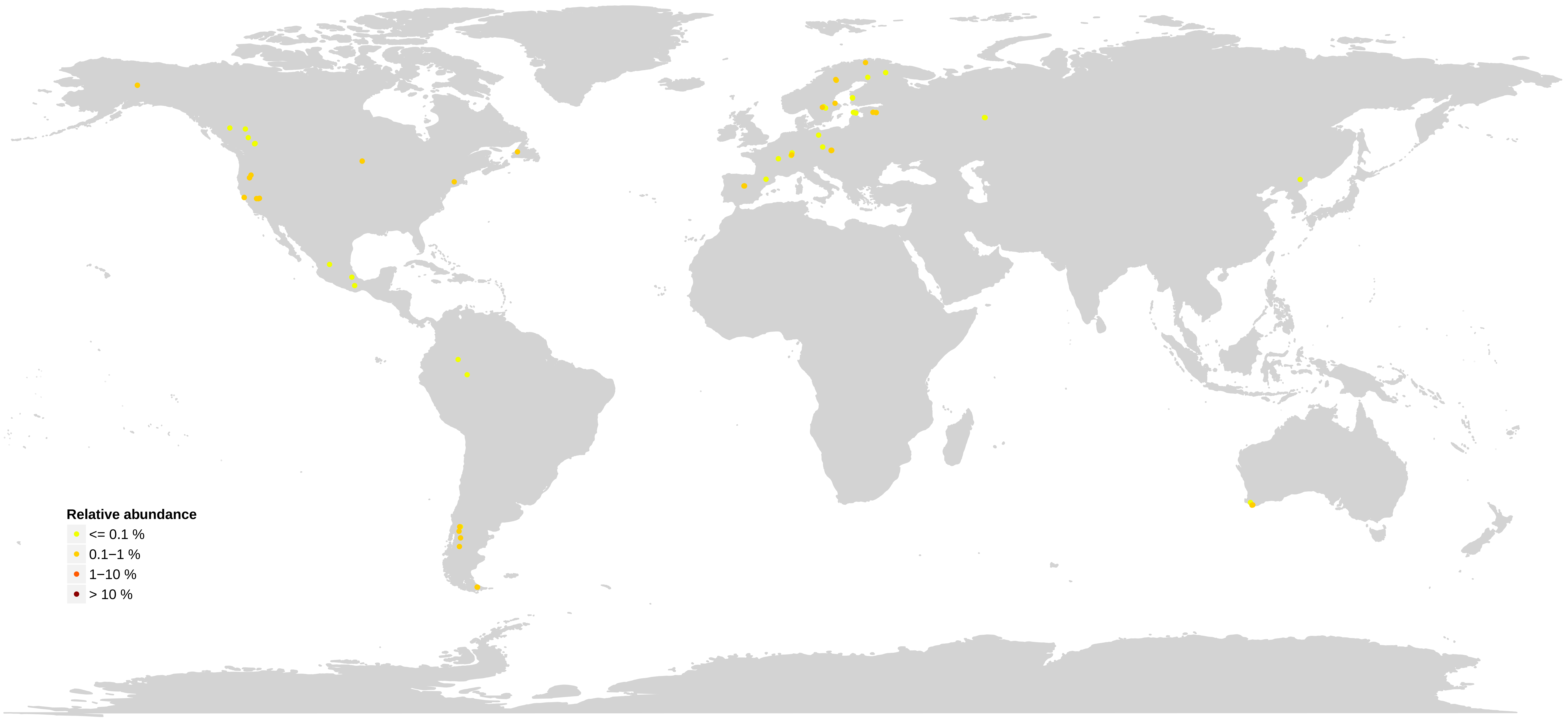

SH012122 Pochonia cordycepisociata

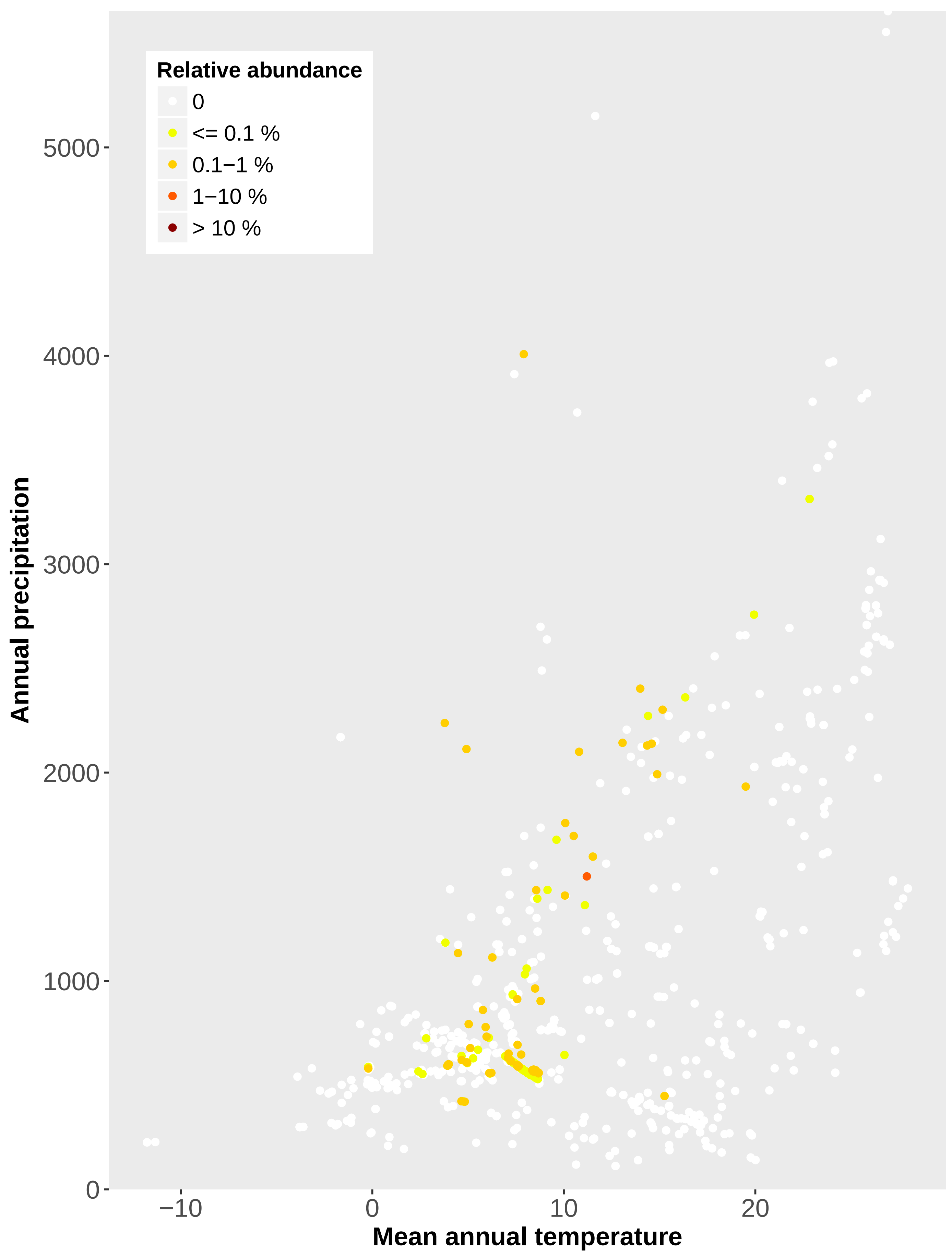

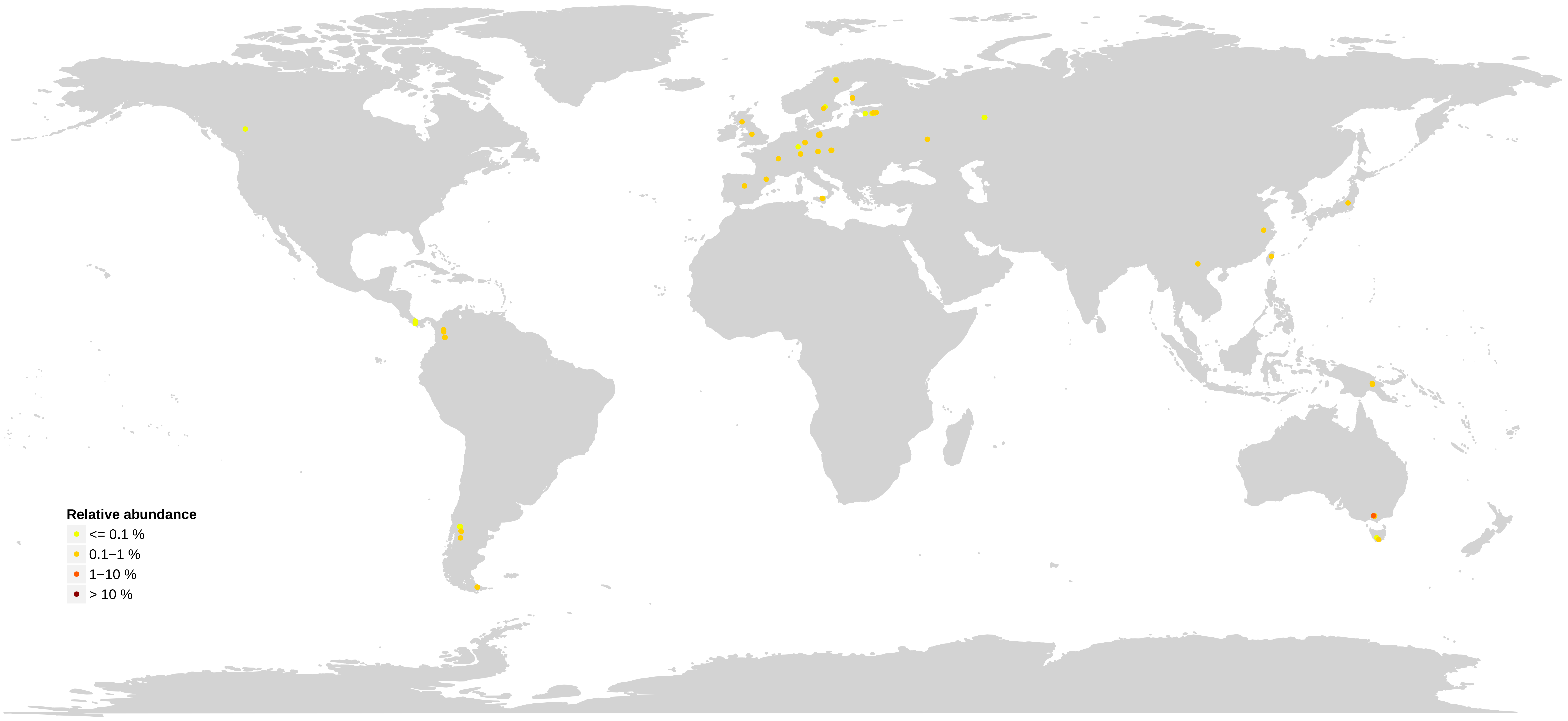

SH187072 Fungi sp

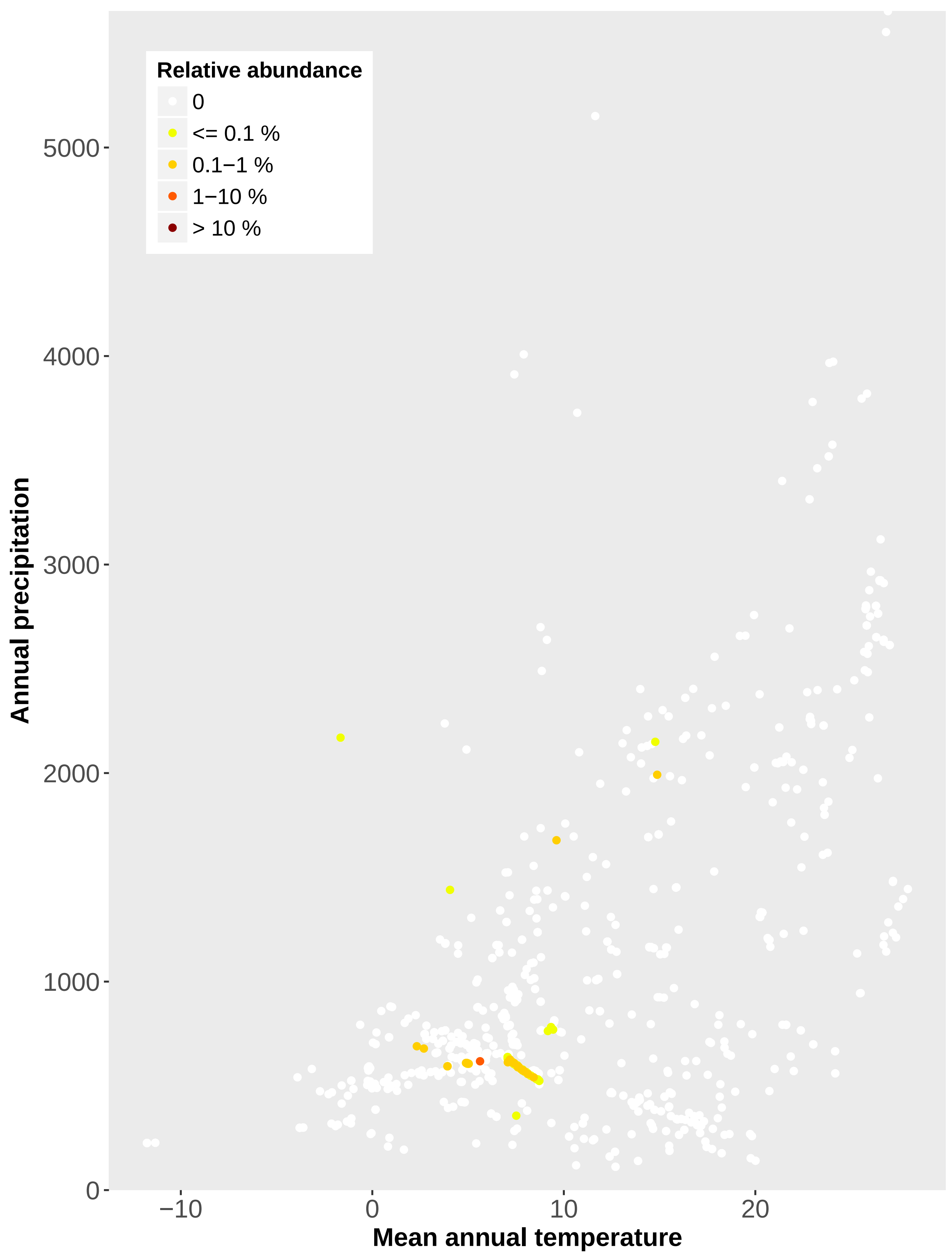

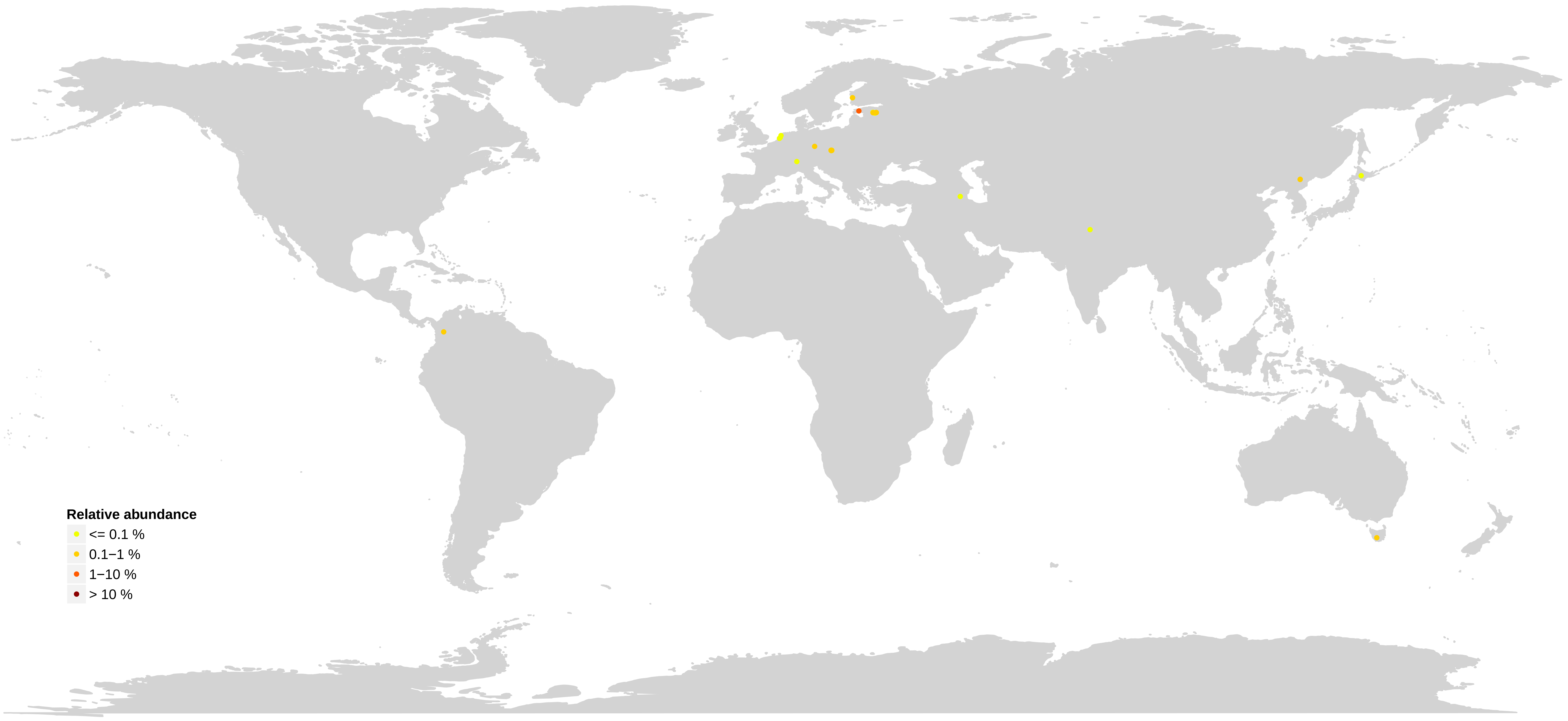

SH195328 Chaetomiaceae sp

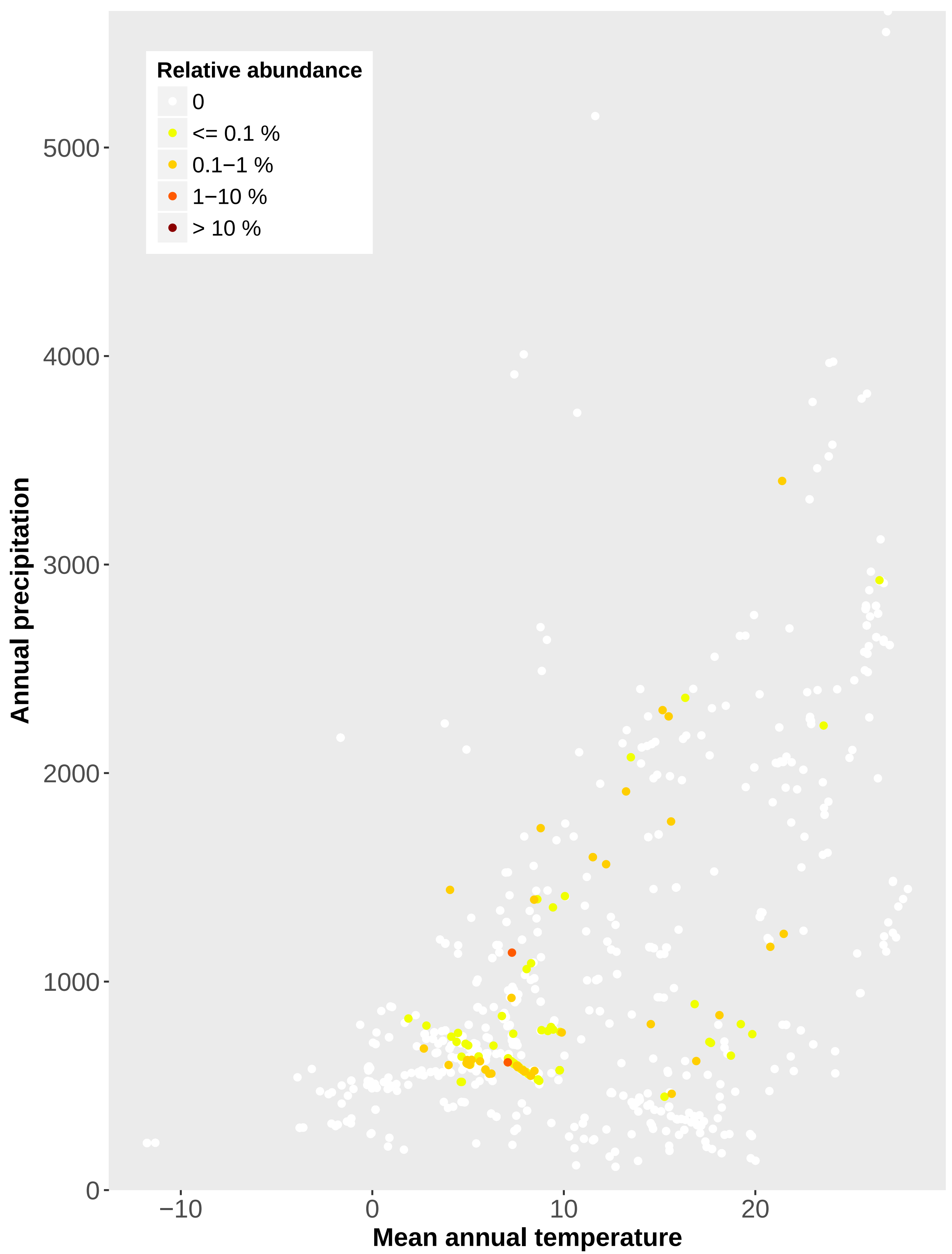

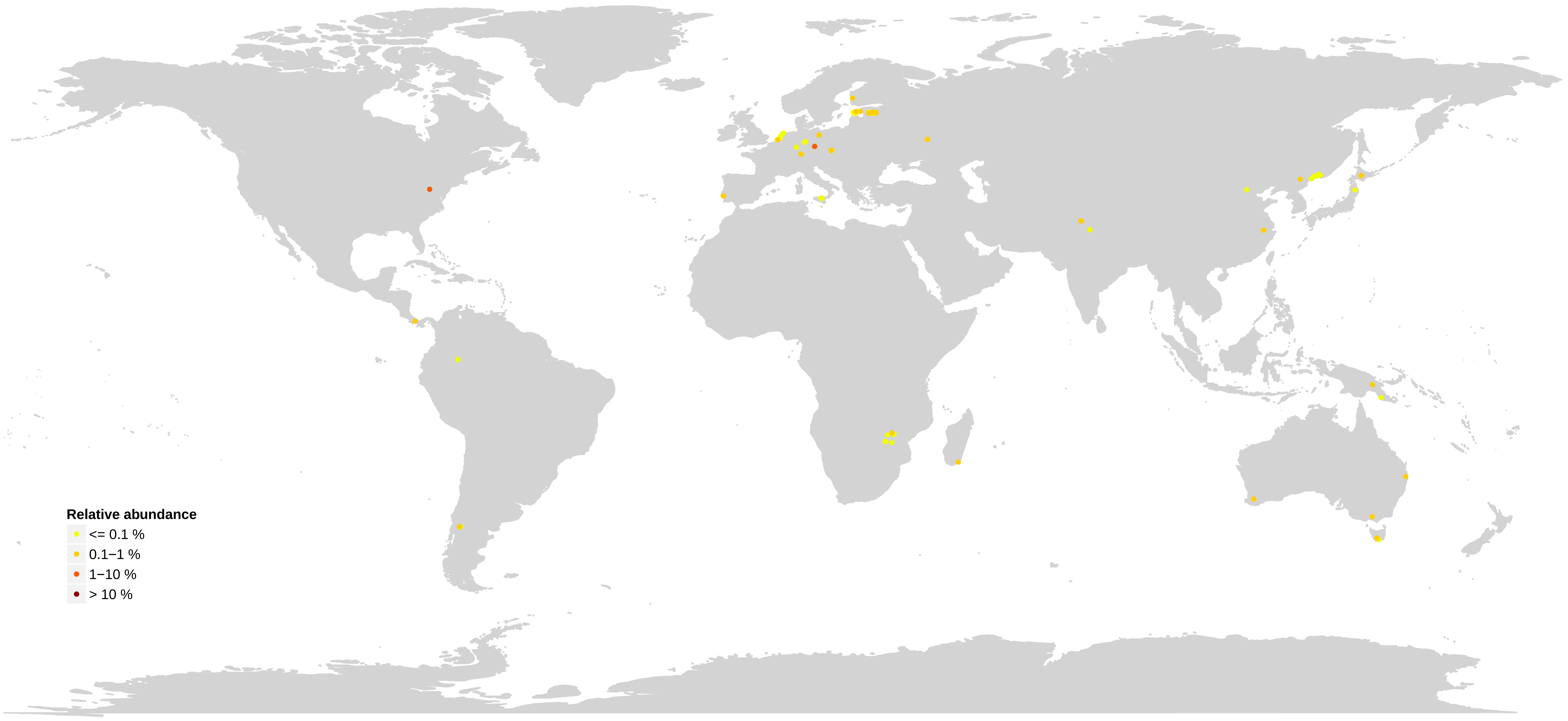

SH199199 Exophiala moniliae

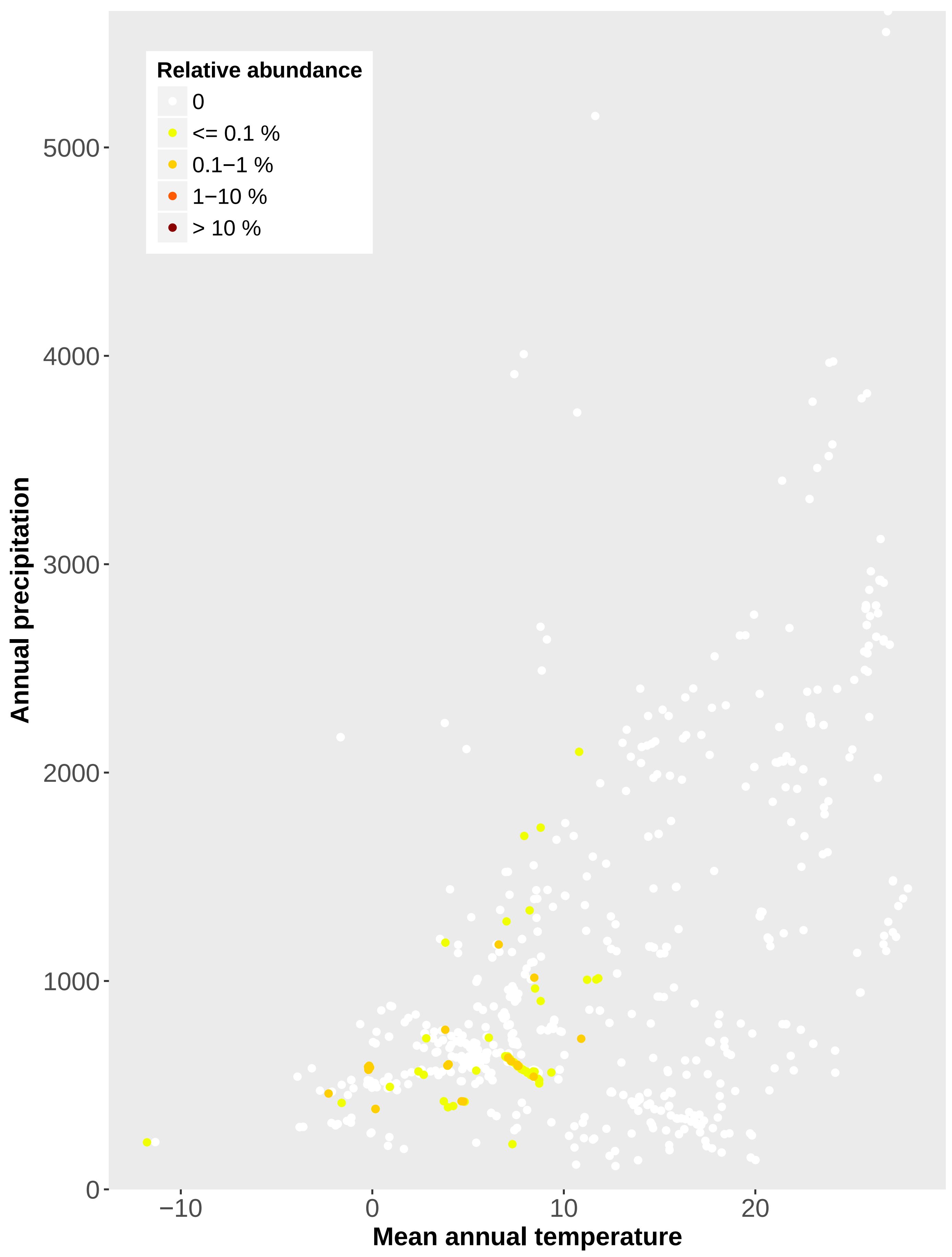

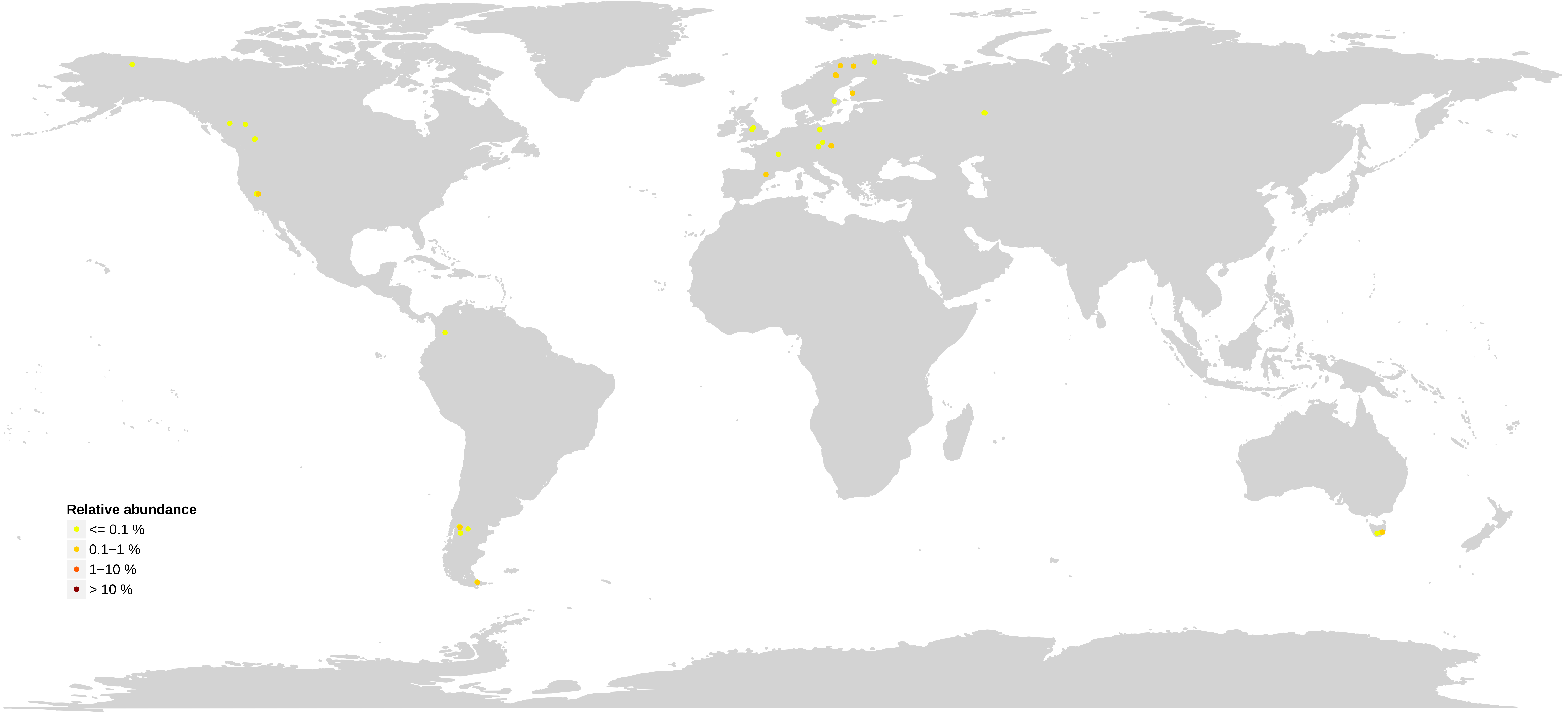

Supplementary Figure 5: Geographic distribution and sequence abundances of the most common fungal taxa with mean annual temperatures/annual precipitation for the corresponding samples.

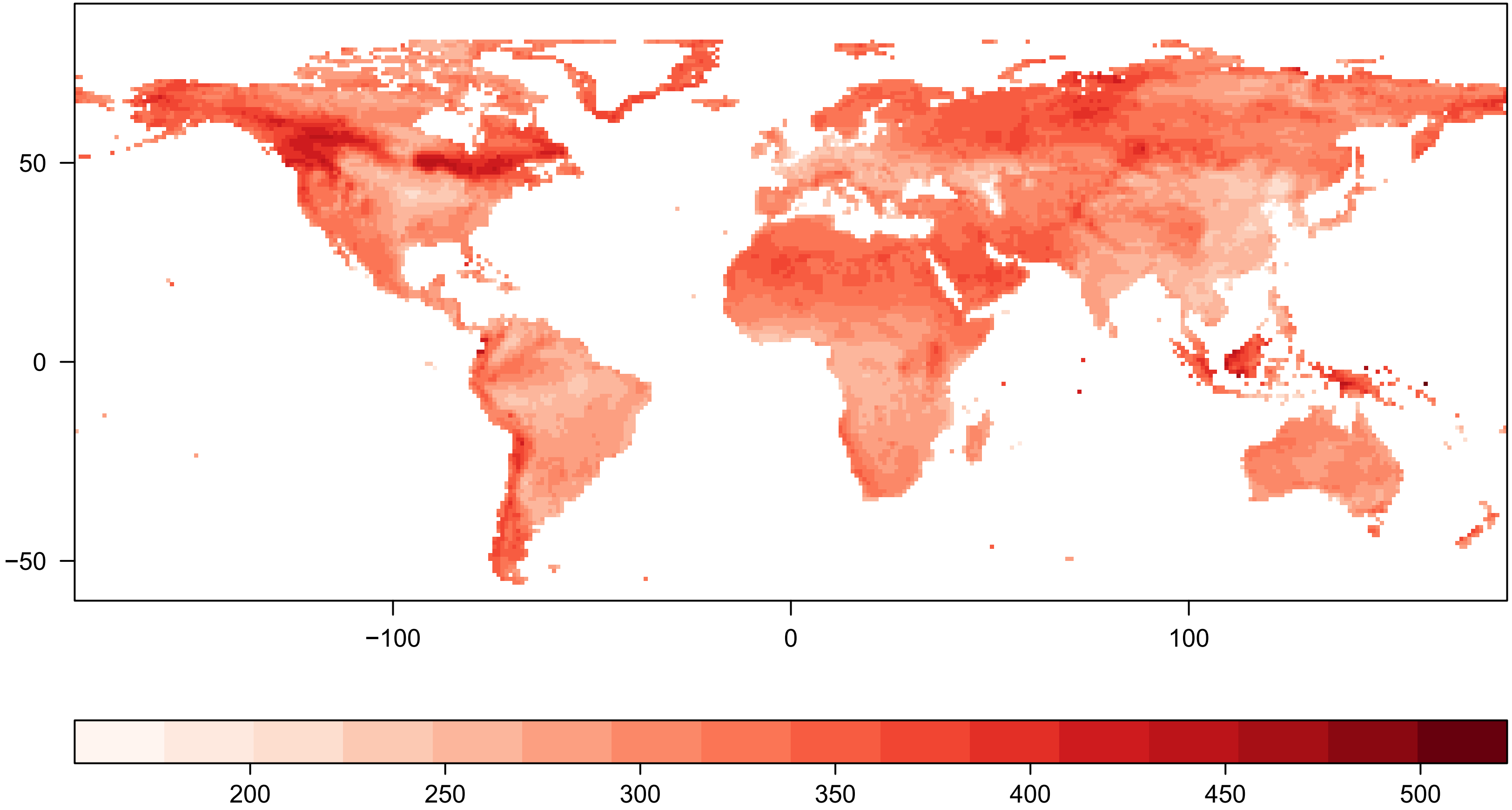

50
0
−50
−100
0
100
200
250
300
350
400
450
500

Supplementary Figure 6: Inferred patterns of fungal species diversity (OTU richness) predicted by the best-subset GLM (model R^2 = 14.8%).

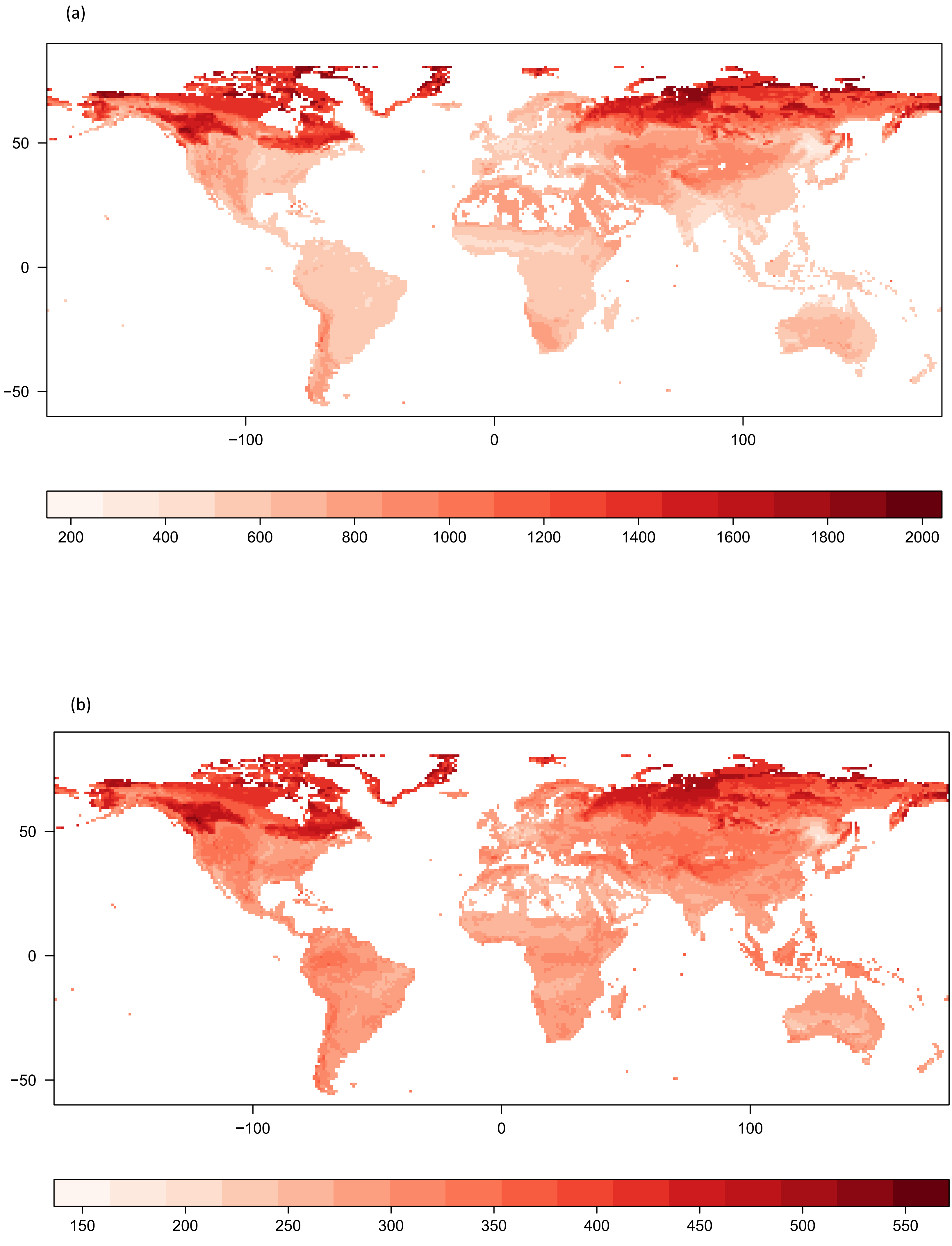
(a)
50
0
−50
−100
0
100
200
400
600
800
1000
1200
1400
1600
1800
2000
(b)
50
0
−50
−100
0
100
150
200
250
300
350
400
450
500
550

Supplementary Figure 7: Inferred patterns of fungal species diversity predicted by the random forest. **a,** The Chao index of the OTU diversity projection (model R^2 = 39.46%); **b,** OTU richness projection (model R^2 = 29.55%).

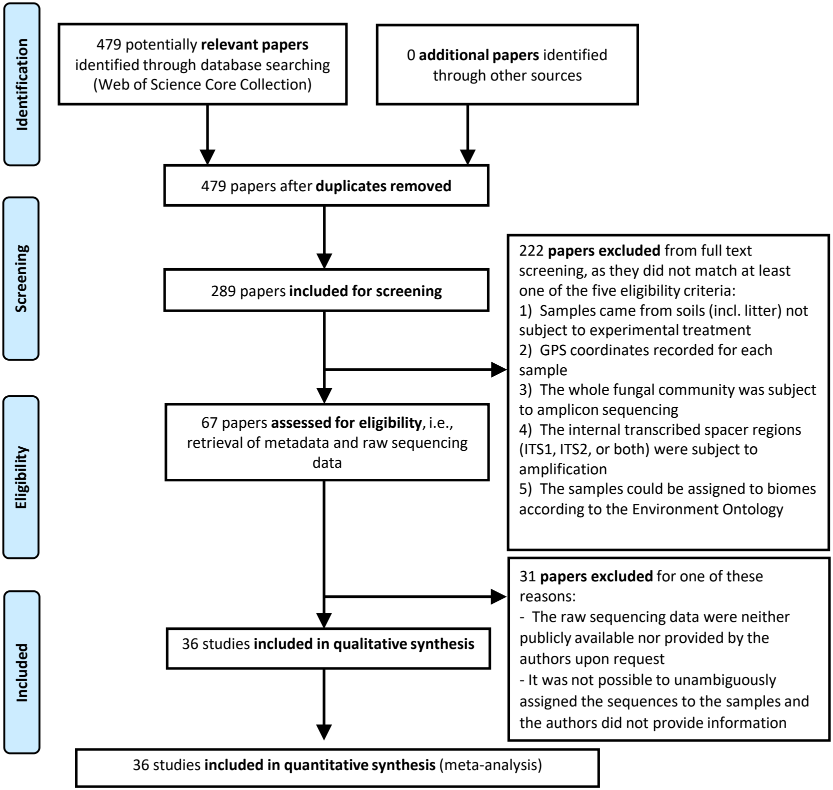
Identification
479 potentially **relevant papers** identified through database searching (Web of Science Core Collection)
0 **additional papers** identified through other sources
479 papers after **duplicates removed**
Screening
289 papers **included for screening**
222 **papers excluded** from full text screening, as they did not match at least one of the five eligibility criteria:
1) Samples came from soils (incl. litter) not subject to experimental treatment
2) GPS coordinates recorded for each sample
3) The whole fungal community was subject to amplicon sequencing
4) The internal transcribed spacer regions (ITS1, ITS2, or both) were subject to amplification
5) The samples could be assigned to biomes according to the Environment Ontology
Eligibility
67 papers **assessed for eligibility**, i.e., retrieval of metadata and raw sequencing data
31 **papers excluded** for one of these reasons:
- The raw sequencing data were neither publicly available nor provided by the authors upon request
- It was not possible to unambiguously assigned the sequences to the samples and the authors did not provide information
Included
36 studies **included in qualitative synthesis**
36 studies **included in quantitative synthesis** (meta-analysis)

Supplementary Figure 8: PRISMA Flow Diagram reporting literature search and selection process.

Supplementary Table 1: List of included studies.

Source	Sequence data availability	Raw sequences	Samples
Baldrian 2012	MG RAST 4783710.3	11702	8
Buéé 2009	www.biomed.cas.cz/mbu/lbwrf/metastudy_datasets/Buee_2009_C0E2.zip	180213	6
Clemmensen 2015	NCBI SRA SRP016090	665639	466
Elliott 2015	NCBI SRA SRP048856	199855	18
Geml 2014	European Nucleotide Archive 0 ERP001713	596422	10
Geml 2015	Dryad Digital Repository DOI https://doi.org/10.5061/dryad.2fc32	2133897	10
Goldmann 2015	NCBI SRA SRP049544	149079	48
Hartmann 2012	Supplementary Data	2224307	6
Chaput 2015	NCBI BioProject PRJNA229802	30969	4
Ihrmark 2012	NCBI SRA SRA052087	493590	12
Kadowaki 2014	DNA Data Bank of Japan (DDBJ) Sequence Read Archive (DRA) DRA000937	67936	42
Leff 2015	NCBI SRA SRP052716	17233559	7
Liu 2015	NCBI SRA SRP041347	290152	26
Maestre 2015	NCBI BioProject PRJNA301533	20636752	155
Mašínová 2016	MG-RAST 0 4696490.3	3157547	264
McHugh 2014	www.biomed.cas.cz/mbu/lbwrf/metastudy_datasets/McHugh_2014_F229.zip	149335	22
McHugh 2015	MG-RAST 0 4563787.3 and 4563788.3	651992	20
Mello 2011	NCBI SRA SRP006078	14776	2
Oja 2015	UNITE repository (http://unite.ut.ee) DOI 10.15156/BIO/100002	166863	90
Op De Beeck 2014	NCBI SRA SRP026207	45182	79
Op De Beeck 2015	NCBI SRA SRP028404	239742	215
Pansu 2015	Dryad Digital Repository DOI http://dx.doi.org/10.5061/dryad.t8534	7697226	0
Peay 2013	www.biomed.cas.cz/mbu/lbwrf/metastudy_datasets/Peay_2013_74BB.zip	212064	62
Rime 2015	Dryad Digital Repository DOI https://doi.org/10.5061/dryad.gp302	244336	36
Rincón 2015	NCBI SRA SRP045166	225746	27
Schmidt 2013	European Nucleotide Archive 0 PRJEB3999	2613965	16
Sterkenburg 2015	NCBI SRA SRP035356	359030	56
Sun 2015	European Nucleotide Archive 0 PRJEB6382	321135	40
Talbot 2014	NCBI SRA SRP040314	1138403	598
Tedersoo 2014	NCBI SRA SRP043706	2122352	365
Tedersoo 2015	NCBI SRA SRP055957	894604	23
Tedersoo 2016	NCBI SRA SRP043706	900801	138
Urbanová 2015	MG-RAST 0 4620497.3 and 4620498.3	87761	50
Veach 2015	NCBI SRA SRX1129668–1129669	1371524	91
Voříšková 2014	MG-RAST 0 4524551.3	135830	48
Žifčáková 2016	MG-RAST 0 4544233.3	229318	24
