# Supplementary material for: A meta-analysis of global fungal distribution reveals climate-driven patterns
Source: Nat Commun. 2019 Nov 13;10:5142. doi: 10.1038/s41467-019-13164-8 (PMC6853883; doi:10.1038/s41467-019-13164-8)
Supplement: Supplementary file 2 — Description of Additional Supplementary Files [file 41467_2019_13164_MOESM2_ESM.pdf]

Description of Additional Supplementary Files

File Name: Supplementary Data 1

Description: Most frequently recorded fungal species hypotheses (SHs). SHs found in >5% of those environmental samples that contained at least 1000 sequences mapped to SHs.

File Name: Supplementary Software 1

Description: Supplementary Software 1 contains all R scripts used by the authors to analyze the drivers of distribution of fungal taxa and of fungal diversity.
