# Supplementary material for: A meta-analysis of global fungal distribution reveals climate-driven patterns
Source: Nat Commun. 2019 Nov 13;10:5142. doi: 10.1038/s41467-019-13164-8 (PMC6853883; doi:10.1038/s41467-019-13164-8)
Supplement: Supplementary file 5 — Reporting Summary [file 41467_2019_13164_MOESM5_ESM.pdf]

nature research

Corresponding author(s): Petr Baldrian

Last updated by author(s): Oct 10, 2019

Reporting Summary

Nature Research wishes to improve the reproducibility of the work that we publish. This form provides structure for consistency and transparency in reporting. For further information on Nature Research policies, see Authors & Referees and the Editorial Policy Checklist.

Statistics

For all statistical analyses, confirm that the following items are present in the figure legend, table legend, main text, or Methods section.

n/a	Confirmed	
☐	☒	The exact sample size (n) for each experimental group/condition, given as a discrete number and unit of measurement
☐	☒	A statement on whether measurements were taken from distinct samples or whether the same sample was measured repeatedly
☐	☒	The statistical test(s) used AND whether they are one- or two-sided<br>*Only common tests should be described solely by name; describe more complex techniques in the Methods section.*
☐	☒	A description of all covariates tested
☐	☒	A description of any assumptions or corrections, such as tests of normality and adjustment for multiple comparisons
☐	☒	A full description of the statistical parameters including central tendency (e.g. means) or other basic estimates (e.g. regression coefficient) AND variation (e.g. standard deviation) or associated estimates of uncertainty (e.g. confidence intervals)
☐	☒	For null hypothesis testing, the test statistic (e.g. F, t, r) with confidence intervals, effect sizes, degrees of freedom and P value noted<br>*Give P values as exact values whenever suitable.*
☐	☒	For Bayesian analysis, information on the choice of priors and Markov chain Monte Carlo settings
☒	☐	For hierarchical and complex designs, identification of the appropriate level for tests and full reporting of outcomes
☐	☒	Estimates of effect sizes (e.g. Cohen's d, Pearson's r), indicating how they were calculated

Our web collection on statistics for biologists contains articles on many of the points above.

Software and code

Policy information about availability of computer code

Data collection	Raw data preparation and quality filtering were performed using Seed pipeline v2.1 (http://www.biomed.cas.cz/mbu/lbwrf/seed/).<br>Full ITS1 or ITS2 fungal region was extracted using Perl script ITSx v1.0.11.<br>ITS sequence classification was performed using BLASTn, version 2.5.0.<br>Clustering into OTUs was performed using VSEARCH software, version 2.4.3.<br>All statistical analyses were performed using the R software, version 3.3.2.<br>(more details in Methods section)
Data analysis	Source code provided as Supplementary Software

For manuscripts utilizing custom algorithms or software that are central to the research but not yet described in published literature, software must be made available to editors/reviewers. We strongly encourage code deposition in a community repository (e.g. GitHub). See the Nature Research guidelines for submitting code & software for further information.

Data

Policy information about availability of data

All manuscripts must include a data availability statement. This statement should provide the following information, where applicable:

- Accession codes, unique identifiers, or web links for publicly available datasets
- A list of figures that have associated raw data
- A description of any restrictions on data availability

Data Availability statement provided.

Field-specific reporting

Please select the one below that is the best fit for your research. If you are not sure, read the appropriate sections before making your selection.

☒ Life sciences ☐ Behavioural & social sciences ☐ Ecological, evolutionary & environmental sciences

For a reference copy of the document with all sections, see nature.com/documents/nr-reporting-summary-flat.pdf

Life sciences study design

All studies must disclose on these points even when the disclosure is negative.

Sample size	We analyzed sequencing data and metadata for 3084 soil samples.
Data exclusions	We explored 479 papers with following selection criteria: (1) samples came from soils (including litter) that were not subject to experimental treatment; (2) the precise geographic location of each sample was recorded (GPS coordinates); (3) the whole fungal community was subject to amplicon sequencing (studies using group-specific primers were excluded); (4) the internal transcribed spacer regions (ITS1, ITS2, or both) were subject to amplification; (5) sequencing data were publicly available or provided by the authors of the study upon request, and the sequences were unambiguously assigned to samples; (6) the samples could be assigned to biomes according to the Environment Ontology. In total, 67 publications contained samples that matched our criteria however, for 31 of the publications, we were not able to obtain sequencing data (the data were neither public nor provided by the authors upon request).<br>Poorly preserved samples that were dominated by moulds (relative abundance of sequences belonging to >20% Mortierellaceae, >20% Mucoraceae or >5% Trichocomaceae) were removed as previously recommended since high mould dominance typically results from growth after sampling and does not correspond to the abundance of mould in soil. Finally we obtain 3084 soil samples.
Replication	Our study contains only biological replicates from individual studies.
Randomization	This research was based on observation from already published studies, the authors did not affect experimental design of individual studies.
Blinding	N/A

Reporting for specific materials, systems and methods

We require information from authors about some types of materials, experimental systems and methods used in many studies. Here, indicate whether each material, system or method listed is relevant to your study. If you are not sure if a list item applies to your research, read the appropriate section before selecting a response.

Materials & experimental systems

n/a	Involved in the study
☒	☐ Antibodies
☒	☐ Eukaryotic cell lines
☒	☐ Palaeontology
☒	☐ Animals and other organisms
☒	☐ Human research participants
☒	☐ Clinical data

Methods

n/a	Involved in the study
☒	☐ ChIP-seq
☒	☐ Flow cytometry
☒	☐ MRI-based neuroimaging
